# CO_2_ capture by fluorinated-imidazolium based ionic liquids: a multiple minima hypersurfaces analysis

**DOI:** 10.3389/fchem.2026.1820345

**Published:** 2026-05-28

**Authors:** Jennifer Cuellar, Osvaldo Yáñez, Sol M. Mejía

**Affiliations:** 1 Línea de Investigación en Química Computacional, Grupo de Investigación GIFUJ, Departamento de Química, Facultad de Ciencias, Pontificia Universidad Javeriana, Bogotá, Colombia; 2 Departamento de Ciencias Básicas y Modelado. Facultad de Ciencias Naturales e Ingeniería. Universidad Jorge Tadeo Lozano, Bogotá, Colombia; 3 Centro de Modelación Ambiental y Dinámica de Sistemas (CEMADIS), Facultad de Ingeniería y Negocios, Universidad de Las Américas, Santiago, Chile

**Keywords:** carbon dioxide capture, density functional theory, intermolecular interactions, ionic liquids, physisorption

## Abstract

Ionic liquids (ILs) are a promising alternative for CO_2_ capture, offering high stability, environmental friendliness, and scalability. The present theoretical study focused on the evaluation of 12 ILs for the capture of up to 5 CO_2_ molecules. The 12 ILs are based on imidazole and fluorine, [C_8_H_4_F_13_mim]^+^[BF_4_]^−^, [C_8_H_4_F_13_mim]^+^[TFO]^−^, [Dmim]^+^[BF_4_]^−^, [Dmim]^+^[TFO]^−^, [Hmim]^+^[FAP]^−^, [Dbim]^+^[FAP]^−^, [Hmim]^+^[methide]^−^, [Dbim]^+^[methide]^−^, [Hmim]^+^[(PFOc)SO_3_]^−^, [Omim]^+^[(PFOc)SO_3_]^−^, [Hmim]^+^[(PFBu)SO_3_]^−^, and [Omim]^+^[(PFBu)SO_3_]^−^. A stochastic dynamic search algorithm was used to generate the structure of the nCO_2_-IL clusters, with n = 1 to 5. The molecular clusters were then optimized using Density Functional Theory (DFT) calculations to describe the nature of the interactions between the 12 ILs and CO_2_. Results show that CO_2_ physisorps and preferentially interacts with anions, also fluorine substitution increasing CO_2_ affinity. Cluster formation is exothermic and favored at lower temperatures. The physisorption is driven by weak van der Waals interactions. The [Dbim]^+^[FAP]^-^, [C_8_H_4_F_13_mim]^+^[BF_4_]^−^ and [C_8_H_4_F_13_mim]^+^[TFO]^−^ ILs demonstrated the highest CO_2_ binding capacity but at the simulated conditions the process is not spontaneous. These findings are useful for designing efficient CO_2_ capture materials based on ILs.

## 1 Introduction

Carbon dioxide capture demands a significant amount of energy, requiring substantial costs primarily obtained from carbon taxes and tax incentives. This is why, over the last few years, the development of new technologies that are efficient and economically sustainable has been actively pursued. A large number of techniques have been developed at both industrial and laboratory scales, including processes such as molecular sieving, carbamation, physical adsorption, dry amine washing, mineral carbonation, and physisorption/chemisorption processes (Hasib-ur-Rahman et al., 2010; Zeng et al., 2017; Zhao et al., 2025). Currently, the most widely used capture technique is amine scrubbing, as it is the most cost-effective technology available to date and one of the most developed processes at the industrial level. This technique is based on the reaction of CO_2_ with amines, forming carbamate, which allows for its removal from the atmosphere. However, the conditions of combustion gases, such as the low partial pressure of CO_2_ and the high temperatures they can reach, lead to losses through evaporation and degradation of the amines. Furthermore, this process can produce toxic SO_x_ and NO_x_ gases. It has also been found that amines are corrosive and difficult to recycle, making this process less environmentally friendly than expected. Although amine scrubbing is the cheapest technology developed thus far, it is still a relatively expensive process due to the numerous steps involved in treating and disposing of the resulting compounds (Dutcher et al., 2015) Consequently, research continues to explore new capture techniques and improve existing ones.

Ionic liquids (ILs) have attracted attention for CO_2_ capture owing to their adjustable properties, wide capture range, thermal and chemical stability, negligible vapor pressure, and high solubility–attributes that can provide a solution to the issues associated with amines (Hasib-ur-Rahman et al., 2010; Ramdin et al., 2012). A brief explanation for why ILs exhibits these properties lies in their chemical structure, as they are compounds formed by generally organic cations and inorganic/organic anions that exist as liquids at room temperature (Hurley and Wier, 1951). This characteristic confers on them the aforementioned properties and also renders them highly recyclable, enabling the development of cleaner and more sustainable chemical processes (Brandt et al., 2011). Moreover, ILs possess the capability to dissolve organic, inorganic, and even polymeric materials (Real Academia de Ciencias Exactasy Naturales, F., 2008). Similarly, these types of compounds demonstrate good solubility of gases such as H_2_, O_2_, CO, and CO_2_, exhibit both hydrophobicity and hydrophilicity, have low toxicity, do not induce corrosion, and due to their thermal stability, experience minimal losses during their utilization and recirculation (Hasib-ur-Rahman et al., 2010).

There are two primary types of ILs employed for CO_2_ capture: functionalized and non-functionalized ILs. The key difference lies in the addition of reactive functional groups, such as amines, to either the cation or anion, typically on the carbon chains of the cation. This modification aims to facilitate CO_2_ capture through the formation of carbamates, characteristic of chemical absorption methods (Mumford et al., 2017). However, chemical absorption can also occur in ILs with basic anions, particularly those formed by strong Lewis bases and imidazolium-based cations. This is because the basic anion can abstract a proton from the carbon chain of the cation, forming a carbene to which the CO_2_ molecules can add, thus forming stable bonds (Blath et al., 2012; Cadena et al., 2004; Zalewski et al., 2021). In contrast, non-functionalized ILs relies on the physical solubility of CO_2_, where the anion plays a more significant role, while the cation exerts a lesser influence (Sistla and Sridhar, 2021). The existence of these two types of ILs offers an additional advantage over traditional solvents, as the type of sorption can be tailored by selecting appropriate cations, anions, and substituents. This versatility highlights the adaptability of ionic liquids for CO_2_ capture.

A disadvantage associated with the use of these compounds is their high viscosity, which is regulated by the type of cation and anion that make up the IL. In the case of the anion, the weaker the van der Waals interactions or hydrogen bonds it can form, the higher the viscosity. Conversely, for cations, the length of the alkyl chain, as well as the degree of branching and the nature of substituents, influence this property. It is important to consider that high viscosity directly affects the solubility of gases, as higher viscosity impedes the transfer of matter and the dispersion of phases, directly affecting gas diffusion in this type of compound, thus conditioning their capture kinetics (Rogers et al., 2012). Nevertheless, this issue can be mitigated by selecting a suitable cation and anion according to the type of capture to be performed. The challenge resides in the choice of anions and cations from the vast array of existing compounds of this type. Consequently, an in-depth study of the types of anions and cations, their structures, functional groups, and their respective applications should be undertaken. Not only is viscosity affected by the type of anion and cation present in the IL, but other properties, such as electrical conductance, melting point, and relative energy released, are also influenced by the structure and functional groups present in the respective ions (Rogers et al., 2012). Another drawback to consider is that the weight percentages of CO_2_ absorption by these compounds have not reached the levels of other technologies. Reported CO_2_ uptake capacities for ionic liquids (ILs) vary widely depending on their structure and experimental conditions (Faisal Elmobarak et al., 2023; Fu et al., 2022; Kang et al., 2020; Luo et al., 2016; Sarmad et al., 2017; Sohaib et al., 2020; Zeng et al., 2017; Zhu et al., 2017), see representative examples of these experimental values in Supplementary Table S1 of the Supplementary Material (SM).

Since adjusting the ionic components can alter the IL’s properties, this presents both an advantage and a disadvantage when studying CO_2_ capture with this type of compound. It opens a vast opportunity for studying different cation-anion combinations, yet simultaneously poses a challenge, as understanding the CO_2_ solubility process at the molecular level is necessary. This includes comprehending the interactions between CO_2_ and ionic liquids and how these interactions determine the process’s execution. Such understanding is crucial for determining the ideal combination characteristics for this purpose and proposing changes that allow obtaining ILs with high capture capacities.

It has been found that most cations possess an aromatic nature with nitrogen atoms in the ring, while anions can be formed by different chemical elements. However, the ILs of interest in this study are focused on those that have demonstrated an efficient rate of absorption and decomposition of CO_2_ at an experimental level, among which are cations composed of an imidazole ring and anions containing fluorinated compounds (Babamohammadi et al., 2015). Considering the vast amount of possible cation-anion combinations that can be performed and meet the aforementioned characteristics, it is necessary to develop systematic studies focused on analyzing these combinations at the molecular level, where intramolecular interactions (anion-cation) and intermolecular interactions (ILs-CO_2_) are characterized. These interactions explain the physicochemical properties of these compounds, and from them, it is possible to determine the nature of the CO_2_ capture process. To this end, theoretical methods are highly useful, as they can be employed to determine the physical and chemical properties of this type of compound, reducing the experimental efforts and costs associated with the synthesis of ILs on the laboratory (Jensen, 2017; Sistla and Sridhar, 2021).

Computational chemistry presents an alternative for studying the capture process, where Density Functional Theory (DFT) can be employed to determine the structural characteristics of ILs, their physicochemical properties, and their interactions with other molecules. Regarding investigations conducted to ascertain how the nature of interactions in these compounds influences the capture process, several studies have been undertaken. One such study by Maxiem Mercy et al. characterized the interactions of superbasic ILs comprising phosphonium cations combined with imidazole and azolide anions. It was found that the cation’s influence was significant in determining the overall binding strength, and there existed no relationship between pairing energy and adsorption capacity (Mercy et al., 2015). In our previous work explored the capture mechanism of four non-functionalized ILs ([Bmim]^+^[PF_6_]^−^, [Emim]^+^[PF_6_]^−^, [Bmim]^+^[TFSI]^−^, [Bmim]^+^[TFSI]^−^, [Bmim]^+^[TFSI]^−^), theoretically corroborating that the CO_2_ capture process with ILs occurs through physisorption, with CO_2_ tending to aggregate between the anion and cation but interacting more with the anion (Alvarez Becerra et al., 2022). A study conducted by Sudha Sistla and Vignesh Sridhar investigated ion-ion and IL-CO_2_ interactions, considering cations with imidazole and pyridine, while for anions, compounds with fluorine, bromine, chlorine, and sulfur were chosen. This study highlighted the importance of anion-CO_2_, cation-CO_2_, IL-CO_2_, and anion-cation interactions. It was also determined that the cation-anion interaction energy increases CO_2_ solubility, as the lower this energy, the higher the physical absorption of CO_2_ (Sistla and Sridhar, 2021). There are also studies, such as those by Palomar et al. (2011) and Alvarez Becerra et al. (2022), that evaluated the influence of anions and cations on the CO_2_ capture process. Additionally, these studies compare values of capture parameters such as Henry’s law, densities, enthalpies, and entropies of dissolution for ILs calculated from a computational perspective with experimentally determined values. These investigations show that DFT calculations yield approximate values very close to those obtained experimentally for these properties.

Although the outlook is favorable in terms of experimental research, it remains necessary to conduct systematic studies on numerous additional combinations of ions. Furthermore, studies should be undertaken to evaluate the effect of including more than one CO_2_ molecule on the surface of the ILs and how this increases conditions the capture and modifies the anion-cation and IL-CO_2_ interactions, since in this type of system, the properties of the clusters can change with the addition of molecules.

Based on this, the present study extends the analysis to a significantly expanded set of IL (twelve) while further exploring multiple CO_2_ loading conditions, providing new insights into adsorption behavior, particularly regarding cooperative effects and their implications for realistic CO_2_ capture scenarios.

## 2 Methods

### 2.1 Computational details

For more clarity, the detailed abbreviations and definitions used in the paper are listed in Table 1.

**TABLE 1:** List of abbreviations and acronyms used in the paper.

Abbreviation	Definition	Abbreviation	Definition
CO_2_	Carbon dioxide	[C_8_H_4_F_13_mim]^+^	1-(3,3,4,4,5,5,6,6,7,7,8,8,8-tridecafluorooctyl)-3-methylimidazolium
SO_x_	Sulfur oxides	[Dmim]^+^	1,3-Dimethylimidazolium
NO_x_	Nitrogen oxides	[Hmim]^+^	1-Hexyl-3-methylimidazolium
H_2_	Molecular hydrogen	[Dbim]^+^	1,3-Dibutylimidazolium
O_2_	Molecular oxygen	[Omim]^+^	1-Octyl-3-methylimidazolium
CO	Carbon monoxide	[Bmim]^+^	1-Butyl-3-methylimidazolium
IL	Ionic liquid	[Emim]^+^	1-Ethyl-3-methylimidazolium
ILs	Ionic liquids	[BF_4_]^−^	Tetrafluoroborate
DFT	Density functional theory	[TFO]^−^	Trifuoromethanesulfonate
PES	Potential energy surface	[FAP]^−^	Tris-(pentafluoroethyl)trifluorophosphate
M06-2X	Minnesota functional	[(PFBu)SO_3_] ^−^	Perfluorobutanesulfonate
6-31G(d,p)	Basis set	[(PFOc)SO_3_] ^−^	Heptadecafluorooctanesulfonate
Cc-pVTZ	Basis set	[PF_6_] ^−^	Hexafluorophosphate
SMD	Continuum solvation model based on the quantum mechanical charge density of a solute molecule	[TFSI]^−^	bis(trifluoromethylsulfonyl)imide
PM7	Parametric method seven	E	Energy
COSMO	Conductor-like screening model	H	Enthalpy
GM	Global mínima	G	Gibbs free energy
BCP	Bond critical points	S	Entropy
ρ(_rc_)	Electron density	ΔE	Formation energy
∇_2_ρ(_rc_)	Laplacian of the electron density	ΔH	Formation enthalpy
H(_rc_)	Electron energy density	ΔG	Formation gibbs free energy
lV(_rc_)l/G(_rc_)	Relation between the virial field and the kinetic energy density	ΔS	Formation entropy
lV(_rc_)l	Virial field	D3	Grimme dispersion correction
G(_rc_)	Kinetic energy density	EPMs	Electrostatic potential maps
I.E.,	Interaction energy	QTAIM	Quantum theory of atoms in molecules
NCI	Mon-covalent interaction index	BCP	Bond critical point

#### 2.1.1 Exploration of potential energy surface

**FIGURE 1:**
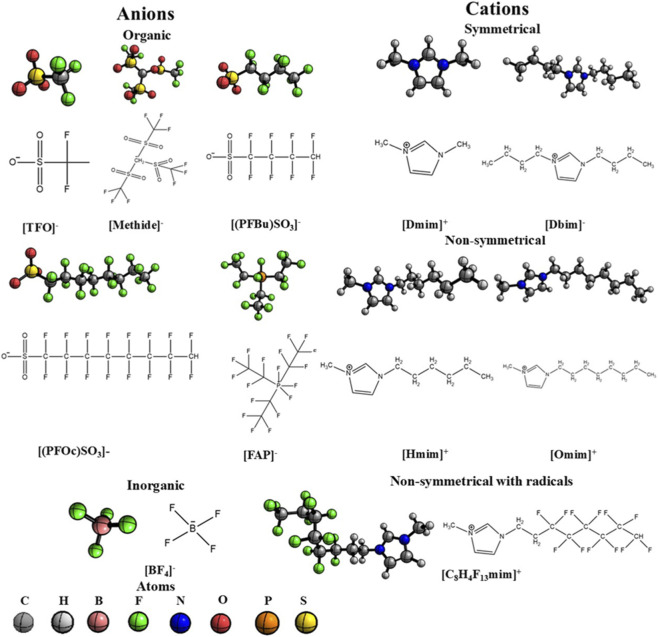
Classification of the anions and cations that make up the ILs studied in this research. Molecular formula: [TFO]^−^ = CF_3_O_3_S^−^, [Methide]^−^ = C_4_F_9_O_6_S_3_^−^, [(PFBu)SO_3_]^−^ = C_4_F_9_O_3_S^−^, [(PFOc)SO_3_]^−^ = C_8_F_17_O_3_S^−^, [BF_4_]^−^ = BF_4_^−^, [Dmim]^+^ = C_5_H_9_N_2_^+^, H), [Dbim]^+^ = C_11_H_21_N_2_^+^, [Hmim]^+^ = C_10_H_19_N_2_^+^, [Omim]^+^ = C_12_H_23_N_2_^+^, K) [C_8_H_4_F_13_mim]^+^ = C_12_H_10_F_13_ N_2_^+^. Refer the IUPAC nomenclature of these structures in Table 1.

In the course of this work, clusters containing ILs with organic cations were analyzed. These cations incorporate the imidazole ring within their structure, differing from each other in terms of the length of the carbon chains replacing the nitrogen atoms. Moreover, a distinction is drawn between symmetric chains, such as [Dmim]^+^ and [Dbim]^+^, and non-symmetric chains, such as [C_8_H_4_F_13_mim]^+^, [Hmim]^+^, and [Omim]^+^. Additionally, a cation containing fluorine atoms is present as radicals in one of the carbon chains, specifically [C_8_H_4_F_13_mim]^+^, as depicted in Figure 1. Regarding the anions, all of them contain fluorine atoms in their structures. However, the differences arise from the fact that four out of the five anions possess an organic nature, such as [TFO]^−^, [FAP]^−^, [Methide]^−^, [(PFOc)SO_3_]^−^, and [(PFBu)SO_3_]^−^, while the [BF_4_]^−^ anion is the only one with an inorganic nature. It is noteworthy that all the organic anions contain sulfur and oxygen atoms, in addition to fluorine, except for [FAP]^−^, which contains only phosphorus atoms along with fluorine. It is also crucial to mention that the anions containing sulfur and oxygen are sulfonates, as they are anions that have the sulfuric anhydride functional group SO_3_^−^ and their general formula is RSO_3_^−^. Varies among them is the R, which in the case of the studied anions is a carbon chain with fluorine substituents that varies in length depending on the anion, except for the [Methide]^−^ anion, where the R is a chain of a single carbon substituted with two additional sulfonates, as depicted in Figure 1.

Already with some context about the nature of both the cations and anions studied during this research, we proceeded to form each of the clusters. The structures of all compounds (cations, anions, and CO_2_) were obtained from databases such as PubChem (Kim et al., 2025) and ChemSpider (Royal Society of Chemistry, 2023). The Potential Energy Surface (PES) of the clusters nCO_2_[C_8_H_4_F_13_mim]^+^[BF_4_]^−^, nCO_2_[C_8_H_4_F_13_mim]^+^[TFO]^−^, nCO_2_[Dmim]^+^[BF_4_]^−^, nCO_2_[Dmim]^+^[TFO]^−^, nCO_2_[Hmim]^+^[FAP]^−^, nCO_2_[Dbim]^+^[FAP]^−^, nCO_2_[Hmim]^+^[Methide]^−^, nCO_2_[Dbim]^+^[Methide]^−^, nCO_2_[Hmim]^+^[(PFOc)SO_3_]^−^, nCO_2_[Omim]^+^[(PFOc)SO_3_]^−^, nCO_2_[Hmim]^+^[(PFBu)SO_3_]^−^, and nCO_2_[Omim]^+^[(PFBu)SO_3_]^−^ (where “n” represents the number of CO_2_ molecules in the cluster, varying from 1 to 5) was systematically explored by stochastic search of the molecular fragments. It is important to clarify that the number of five CO_2_ molecules per IL cluster analyzed in this study does not correspond to a saturation limit. Rather, it represents a computationally tractable range where interaction trends could be systematically studied.

This stochastic search of the molecular fragments was carried out using the SnipperKick algorithm (Yañez et al., 2019; 2021), an extension of the methodology developed by Saunders (2004) and Addicoat and Metha (2009). This algorithm is designed to find the putative global minimum in molecular systems composed of both isolated systems and clusters. In this process, molecular fragments are defined by their Cartesian coordinates and placed in a defined box. Each fragment is rotated at a random angle, and then the coordinates of the fragments are translated across the box. Once this process is completed, the resulting structures are locally optimized to determine the cluster energy (Addicoat and Metha, 2009; Saunders, 2004; Yañez et al., 2021).

The exploration of the PES was performed with a neutral global charge, carrying out approximately 10,000 energy calculations for each cluster. The local optimization of the structures was performed by the PM7 semi-empirical method (Stewart, 2013), utilizing the COSMO implicit solvent model (Klamt and Schüürmann, 1993) from the MOPAC2016 package (J.J.P, S, 2016; Stewart and Seiler, 1990). The algorithm was instructed to provide at most the 35 most stable structures in each cluster. Subsequently, the reoptimization and calculation of the frequencies of the obtained structures were conducted using the M06-2X functional (Grimme et al., 2011; Zhao et al., 2006), the 6-31G(d,p) basis set, the Grimme dispersion correction (D3), and the implicit solvent using the SMD parameterization of the IEF-PCM from the Gaussian16 package (Frisch et al., 2016). This approach was employed due to its good performance observed in the description of charged systems (Seeger and Izgorodina, 2020). It was ensured that all the delivered structures were highly stable, as none of them exhibited imaginary frequencies. Finally, the lowest energy structure of each cluster was re-optimized again using the M06-2X functional and the cc-pVTZ basis set. This methodology was previously presented and validated in our study “Exploring the potential energy surface of nCO_2_ (n = 1–5) capture by imidazole- and fluorine-based ionic liquids: A DFT study” (Alvarez Becerra et al., 2022), where we explored the PES of nCO_2_ (n = 1–5) capture by imidazole- and fluorine-based ILs using DFT calculations. We evaluated multiple basis sets (6-31G(d,p), 6–311++G(d,p), and cc-pVTZ) and found that the cc-pVTZ basis set provided the most accurate energy descriptions. Our results also indicate that the most stable structures correspond to the lowest spin multiplicities states.

#### 2.1.2 Thermodynamic properties

The calculation of the thermodynamic properties was performed considering the global minima (GM) found in the exploration of PES (M06-2X-D3/cc-pVTZ and SMD implicit solvent). Five calculations were carried out for each cluster, i.e. 60 in total. These calculations were performed using the supermolecule approximation (Equation 1).

$$\Delta X=X_{cluster}-\sum X_{monomeros}$$ (1)

where “X” represents energy (E), enthalpy (H), Gibbs free energy (G), and entropy (S), depending on the property being evaluated. Essentially, these calculations involve subtracting the property (E, H, G, or S) of the cluster (nCO_2_[Cation]^+^[Anion]^−^ with n = 1–5) from the properties of the involved monomers. In this case, the considered monomers are CO_2_ and ILs, totaling 13 monomers. The properties of these monomers were obtained through optimization and frequency calculations utilizing the M06-2X-D3/cc-pVTZ and SMD implicit solvent.

It is important to note that the results for the enthalpy of formation (ΔH), Gibbs free energy of formation (ΔG) and entropy of formation (ΔS) provided by this approximation are at a temperature of 298.15 K, while the cluster formation energy (ΔE) is found to be calculated at 0 K Furthermore, the thermodynamic properties E and S were calculated from partition functions resulting from the translational, rotational, and vibrational motions of the electrons (Ochterski, 2000), while G and H were calculated traditionally based on thermodynamic principles. These calculations were performed using the Gaussian 16 package (Frisch et al., 2016).

#### 2.1.3 Topological and weak interactions analysis

To analyze the weak interactions, present in the formation of the nCO_2_[Cation]^+^[Anion]^−^ clusters of the GM found in the exploration of PES (M06-2X-D3/cc-pVTZ), the Quantum Theory of Atoms In Molecules (QTAIM) developed by Bader (1990), which is implemented in the AIMAll Professional software version 19.10.12, was employed (Todd, 2019). Through this methodology, it became possible to estimate a number of key topological properties, such as the electron density (ρ), the electron density Laplacian (∇^2^ρ), the total electron energy density (H), the |V|/G parameter, and the interaction energy (I.E.,). These properties were evaluated at the Bond Critical Points (BCPs). Furthermore, the abundances of the non-covalent interactions present in each of the clusters were computed. The amounts of CO_2_-ILs, [Cation]^+^-[Anion]^−^, and CO_2_-CO_2_ interactions were analyzed. As part of the characterization process, visual representations of the molecular graphs were also generated, enabling a better comprehension of the arrangement and nature of the interactions. A complementary study to the QTAIM was developed, which involved the analysis of the Non-Covalent Interaction index (NCI) through the Multiwfn program (Analyzer and Lu, 2019). This study enabled the visualization and description of the non-covalent interactions present in terms of their spatial location and interaction type (such as hydrogen bonds, van der Waals interactions, and steric clashes) (Contreras-García et al., 2011). Furthermore, a color-coding system was utilized to represent the strength of these interactions. For the visualization of the isosurfaces generated in the NCI analysis, the VMD viewer was used (Humphrey et al., 1996). Electrostatic Potential Maps (EPMs) were prepared to analyze the reactivity of the molecular clusters. The primary goal was to identify areas with an excess or deficiency of electron density in anions and cations, and to understand how these areas affect the physisorption of CO_2_ molecules. Creating these maps necessitated calculating the electron density and electrostatic potential. This was accomplished using the “cubegen” tool from the Gaussian16 package, and the resulting maps were visualized through the VMD program.

## 3 Results and discussion

### 3.1 Exploration of the potential energy surface

To determine the CO_2_ sorption mechanism, potential energy surfaces (PES) were extensively explored by allowing the anion, cation, and each CO_2_ molecule to move freely. Up to 10,000 configurations were generated, which, after being optimized with PM7 in the MOPAC program, should provide a maximum of 35 different clusters. Supplementary Table S2 of the SM shows the number of stable structures obtained for each cluster studied (nCO_2_-IL, n = 1–5) during this first PES exploration stage. The PM7 semiempirical optimization yielded 1840 different structures. For most systems, a maximum of 34 different structures were obtained. However, for the ILs [C_8_H_4_F_13_mim]^+^[BF_4_]^−^, [C_8_H_4_F_13_mim]^+^[TFO]^−^, [Dmim]^+^[BF_4_]^−^, and [Dmim]^+^[TFO]^−^, only 24 structures were obtained, regardless of the CO_2_ molecule number. Once these structures were generated as starting points, reoptimization and frequency calculations were performed with the M06-2X-D3/6-31G(d,p) approach. Through these calculations, 74 structures were discarded due to imaginary frequencies or lack of convergence, resulting in 1766 different and stable cluster geometries (nCO_2_-IL, n = 1–5). In other words, less than 5% of the PM7-obtained structures did not lead to stable DFT structures. Finally, the lowest-energy structures were reoptimized using the M06-2X/cc-pVTZ approach.

**FIGURE 2:**
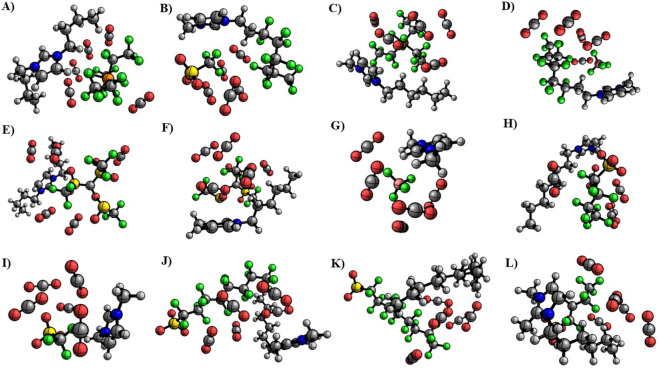
Putative global minimum for 5CO_2_-IL clusters. **(A)** [Dbim]^+^[FAP]^-^, **(B)** [C_8_H_4_F_13_mim]^+^[TFO]^-^, **(C)** [Hmim]^+^[FAP]^-^, **(D)** [C_8_H_4_F_13_mim]^+^[BF_4_]^-^, **(E)** [Dbim]^+^[Methide]^-^, **(F)** [Hmim]^+^[Methide]^-^, **(G)** [Dmim]^+^[BF_4_]^-^, **(H)** [Omim]^+^[(PFBu)SO_3_]^-^, **(I)** [Dmim]^+^[TFO]^-^, **(J)** [Hmim]^+^[(PFOc)SO_3_]^-^, **(K)** [Omim]^+^[(PFOc)SO_3_]^-^, and **(L)** [Hmim]^+^[(PFBu)SO_3_]^−^.

Considering the extensive exploration performed for all isomers in this research, this section presents only the geometries of the 12 putative global minima of the clusters with 5 CO_2_ molecules, as shown in Figure 2. The remaining putative global minima (with CO_2_ to 4CO_2_) can be found in Supplementary Tables S1–S3 of the SM, and additionally, the local minima can be consulted in Supplementary Tables S3–S62 of the SM.

#### 3.1.1 Structure-stability relationship

Generally, the optimized geometries of all clusters corresponding to the global minimum reveal that CO_2_ molecules are predominantly located between the cation and the anion, with a preference for the latter. As the number of CO_2_ molecules increases, they tend to surround the anions, exhibiting similar behavior, and in fact, more clearly as there is a larger number of CO_2_ molecules, as reported in the study by Soniya et al., where it was found that the formation of complexes between 2 ILs ([ETT]^+^[FEP]^−^, [Hmim]^+^[FEP]^−^) and up to 30 CO_2_ molecules occurs due to the anisotropic distribution generated by the fluorine atoms of the anion (Rao and Gejji, 2016). On the other hand, the side chains of the cations play a crucial role in determining the global minimum, as the CO_2_ molecules tend to be located in the areas where these chains are situated. Thus, in ILs with asymmetric chain cations, CO_2_ tends to be located on the longer chain, whereas in clusters with symmetric chains, CO_2_ tends to be located uniformly between the two chains.

Furthermore, in ILs containing fluorine atoms in either the cation and/or the anion, a preferential arrangement of CO_2_ molecules towards these zones is observed. This preference is most noticeable in the nCO_2_[Dbim]^+^[FAP]^−^ clusters, where there are the most fluorine atoms in the anion (18 atoms). These findings align with the results of previous studies that have demonstrated the influence of fluorine-containing substituents on the CO_2_ adsorption properties of ILs. For instance, a study by Almantariotis et al. (2010) found that the affinity of CO_2_ for ILs with fluorinated substituents is due, in part, to the larger free volume in the fluorinated compounds compared to their hydrogenated counterparts. They found stronger interactions of carbon dioxide with the fluorinated alkyl chains due to dispersion forces. Muldoon et al. (2007) showed that fluorine-halogenated ionic liquids exhibit higher CO_2_ adsorption capacity compared to other halogens such as Cl and Br.

Exceptions to the trend that CO_2_ is located close to the anion occur in the CO_2_[C_8_H_4_F_13_mim]^+^[BF_4_]^−^ cluster and in the nCO_2_[C_8_H_4_F_13_mim]^+^[TFO]^−^ clusters. In the second case, where the anion is a sulfonate featuring a one-carbon R-chain attached to the sulfuric anhydride, the CO_2_ is located in the imidazole ring region. As more CO_2_ molecules are added, it is observed that, although there is always a preference for interaction with the anion, after more than three CO_2_ molecules are added, they tend to disperse a little more over the cation. In the second case, after adding three CO_2_ molecules, it is observed that, although these still interact mainly with the R chain of the anion, the CO_2_ molecules disperse a little more along the cation near the longer chain and with fluorine substituents.

Finally, for the clusters nCO_2_[Hmim]^+^[(PFOc)SO_3_]^−^, nCO_2_[Omim]^+^[(PFOc)SO_3_]^−^, nCO_2_[Hmim]^+^[(PFBu)SO_3_]^−^, and nCO_2_[Omim]^+^[(PFBu)SO_3_]^−^, whose cations differ in that [Hmim]^+^ has its longest aliphatic chain of 6 carbons while in [Omim]^+^ it is 8 carbons, it is possible to note that, when CO_2_ molecules are added, they do not tend to be dispersed by either the anion or the cation, but are located more or less uniformly between the anions and the cations.

Based on these results, the CO_2_ capture mechanism for ILs studied in this work occurs through physisorption. This behavior is attributed to the selected cation–anion combinations, which are primarily composed of imidazolium cations substituted with carbon chains, while the anions contain fluorinated groups in their structure.

It has been experimentally observed that although a chemical reaction between the imidazolium ring nitrogen atoms and CO_2_ can occur-leading to carbamate formation-this process strongly depends on the nature of the IL’s anion. For instance, anions bearing carboxylic groups promote chemisorption, as they can abstract a hydrogen atom from the imidazolium nitrogen, thereby enabling nucleophilic attack on CO_2_ (Blath et al., 2012).

In contrast, fluorinated anions, such as those used in this study, generate electron-rich regions due to their high electronegativity. This enhances electrostatic attraction toward CO_2_ molecules, favoring non-covalent interactions. Additionally, the alkyl chains substituting the imidazolium ring on the cation side also play a key role in promoting physisorption. These chains increase the free volume and reduce the strength of cation–anion interactions.

As demonstrated in the study by Umecky et al. (2019), stronger anion–cation interactions tend to create environments that favor chemisorption. In our case, the use of cations with long alkyl chains led to weakened ion-pair interactions, as confirmed by the topological analysis of the electronic density (see Section 3.3), supporting a physisorption-driven mechanism.

### 3.2 Thermodynamic properties

**FIGURE 3:**
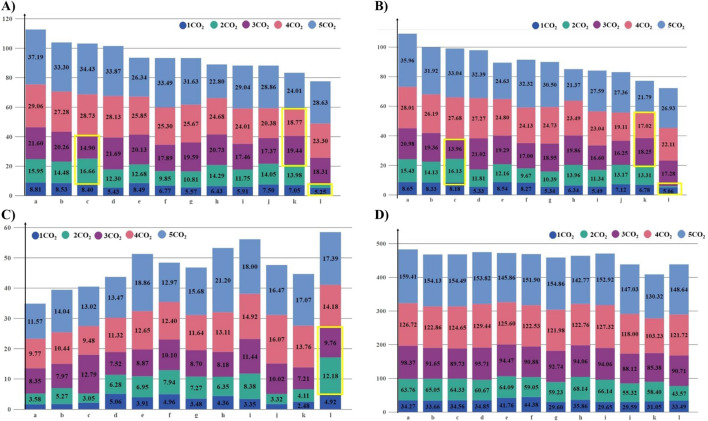
Thermodynamic parameters of nCO2-IL cluster formation; n = 1 to 5; IL = a: [Dbim]+[FAP]−, b: [C8H4F13mim]+[TFO]−, c: [Hmim]+[FAP]−, d: [C8H4F13mim]+[BF4]−, e: [Dbim]+[Methide]−, f: [Hmim]+[Methide]−, g: [Dmim]+[BF4]−, h: [Omim]+[(PFBu)SO3]−, i: [Dmim]+[TFO]−, j: [Hmim]+[(PFOc)SO3]−, k: [Omim]+[(PFOc)SO3]−, l: [Hmim]+[(PFBu)SO3]−). **(A)** Energy (ΔE, kcal/mol), **(B)** Enthalpy (ΔH, kcal/mol), **(C)** Gibbs free energy (ΔG, kcal/mol), D) Entropy (ΔS, cal mol−1K−1). Yellow boxes highlight the exceptions to the trend where thermodynamic values increase as the number of CO2 molecules increases.

The results of the different thermodynamic properties of formation (ΔE, ΔH, ΔG, ΔS) are presented in Figure 3 for the 60 molecular clusters studied, i.e., for the twelve salts and their combinations with 1, 2, 3, 4, or 5 CO_2_ molecules. Values of ΔE and ΔH up to −37.19 kcal/mol and −35.96 kcal/mol, respectively, are observed, indicating exothermic physisorption processes due to interactions as weak as the C-H---F interaction of 0.50 kcal/mol (see Section 3.3). Likewise, these processes are non-spontaneous (ΔG > 0), and being an agglomeration process, ΔS < 0 in all cases.

In general, as seen in Figure 3A, ΔE becomes more negative as the number of CO_2_ molecules increases, with three exceptions highlighted with yellow boxes. This behaviour is preserved when temperature effects are included (see ΔH in Figure 3B). However, no single energetic ordering of the clusters can be established, as it depends on the number of CO_2_ molecules considered (Supplementary Table S63). Notably, clusters with [Dbim]^+^[FAP]^-^ are the most stable in three out of 5 systems studied. Moreover, ΔE values are consistently more negative than ΔH values, with differences ranging from 0.16 kcal/mol (in CO_2_[Dbim]^+^[FAP]^-^) to 2.23 kcal/mol (in 5CO_2_[Omim]^+^[(PFOc)SO_3_]^-^). These results are in agreement with our previous findings for related ILs [Bmim]^+^[PF_6_]^-^, [Emim]^+^[PF_6_]^-^, [Bmim]^+^[TFSI]^-^ and [Emim]^+^[TFSI]^-^ and indicate that CO_2_ physisorption becomes less favorable increasing temperature, except for CO_2_[Hmim]^+^[Methide]^-^ (Supplementary Figure S4).

These results enable a more explicit quantitative analysis of CO_2_ adsorption as a function of loading. Overall, adsorption becomes more favorable (i.e., more negative ΔE) as the number of CO_2_ molecules increases; however, this trend is not strictly monotonic and depends on the ionic liquid structure. Importantly, the normalized adsorption energies (ΔE per CO_2_ molecule, Table 2) remain nearly constant across the series, indicating that the interaction strength per molecule does not significantly change with increasing CO_2_ loading. This suggests the absence of strong cooperative or anti-cooperative effects. This behavior is further supported by the fact that only minor deviations are observed in specific cases, such as 3CO_2_[Hmim]^+^[FAP]^-^ and 2CO_2_[Hmim]^+^[(PFBu)SO_3_]^-^, where slightly more favorable formation energies are obtained compared to other clusters. Overall, these confirm that CO_2_ capture in the studied ILs is governed by weak, additive interactions rather than cooperative effects, similar to those systems stabilized by weak π---π and CH---π interactions (Tsuzuki et al., 2002).

**TABLE 2:** Normalization of the formation energy (ΔE) per CO_2_ molecule (N°CO_2_), where ΔE in kcal/mol.

N° CO_2_	ΔE/N° CO_2_	ΔE/N° CO_2_	ΔE/N° CO_2_	ΔE/N° CO_2_	ΔE/N° CO_2_	ΔE/N° CO_2_
​	[C_8_H_4_F_13_mim]^+^ [BF_4_]^−^	[Dmim]^+^ [TFO]^−^	[C_8_H_4_F_13_mim]^+^ [TFO]^−^	[Dmim]^+^ [BF_4_]^−^	[Dbim]^+^ [FAP]^−^	[Dbim]^+^ [Methide]^−^
1	−5.43	−5.91	−8.53	−5.57	−8.81	−8.49
2	−6.15	−5.87	−7.24	−5.40	−7.97	−6.34
3	−7.23	−5.82	−6.75	−6.53	−7.20	−6.71
4	−7.03	−6.00	−6.82	−6.42	−7.26	−6.46
5	−6.77	−5.81	−6.66	−6.33	−7.44	−5.27

CO_2_ No.	[Hmim]^+^ [FAP]^−^	[Hmim]^+^ [Methide]^−^	[Hmim]^+^ [(PFOc)SO_3_]^−^	[Hmim]^+^ [(PFBu)SO_3_]^−^	[Omim]^+^ [(PFOc)SO_3_]^−^	[Omim]^+^ [(PFBu)SO_3_]^−^
1	−8.40	−6.77	−7.50	−5.25	−7.05	−6.43
2	−8.33	−4.93	−7.03	−1.01	−6.99	−7.15
3	−4.97	−5.96	−5.79	−6.10	−6.48	−6.91
4	−7.18	−6.32	−5.09	−5.82	−4.69	−6.17
5	−6.89	−6.70	−5.77	−5.73	−4.80	−4.56

The ΔG values increase as more CO_2_ molecules are added, showing only one exception for [Hmim]^+^[(PFBu)SO_3_]^−^ IL with 2 and 3 CO_2_, see Figure 3C. The clusters with the [Dbim]^+^[FAP]^−^ IL exhibit the smallest increase in ΔG with CO_2_ addition, while [Hmim]^+^[(PFBu)SO_3_]^−^ is the IL with the largest change in ΔG with CO_2_ addition. Therefore, although the cluster formation process is exothermic, the effect of entropy is significant and equally dependent on the number of CO_2_ molecules, rendering the process non-spontaneous due to the decrease in entropy, see Figure 3D. This was expected, as physisorption processes typically exhibit this behavior due to several factors. One factor is the limited adsorption surface area, which means that as more molecules are adsorbed, the available area decreases, and thus the movement and arrangement of adsorbed molecules become restricted, implying a decrease in entropy (Erkey, 2011). Another influencing factor is the formation of intermolecular interactions between the adsorbing compounds and the adsorbed molecules. Since the number of these interactions tends to increase as more and more molecules are adsorbed, this implies that the adsorbed material has lower mobility, and consequently, entropy decreases (Bronowska, 2011; Chang, 2008).

These behaviors will be detailed in the QTAIM analysis (Section 3.3), where it was generally observed that as CO_2_ molecules were added, the total number of ILs-CO_2_ interactions increased, explaining this entropy behavior. Finally, from this analysis, it can be observed in Figure 3D **)** that the cluster with the highest entropy loss is [Dbim]^+^[FAP]^−^, while the cluster with the lowest entropy loss is [Hmim]^+^[(PFBu)SO_3_]^−^. It can also be observed that, unlike ΔE, ΔH, and ΔG, the differences in ΔS from cluster to cluster are not as pronounced; in general, they are all decreasing at approximately the same rate, indicating that ΔS is affected more by the number of CO_2_ molecules absorbed than by the type of IL considered.

### 3.3 Topological analysis of the electron density and non-covalent interactions

A detailed understanding of the level of interactions that give rise to stable CO_2_ uptake by ILs plays an essential role in the design and optimization of ILs. As evidenced in studies such as those of Zhu et al. (2011), where it was observed that the formation of weak interactions is responsible for the stabilization and, thus, the adsorption of CO_2_ in this type of compound. Therefore, a systematic analysis of the abundance and nature of the interactions between the anions and cations of ILs, cations-CO_2_, anions-CO_2_, as well as CO_2_-CO_2_ interactions of each of the studied clusters was carried out. This analysis was performed based on the Quantum Theory of Atoms In Molecules (QTAIM) and the non-covalent interaction (NCI) index.

**FIGURE 4:**
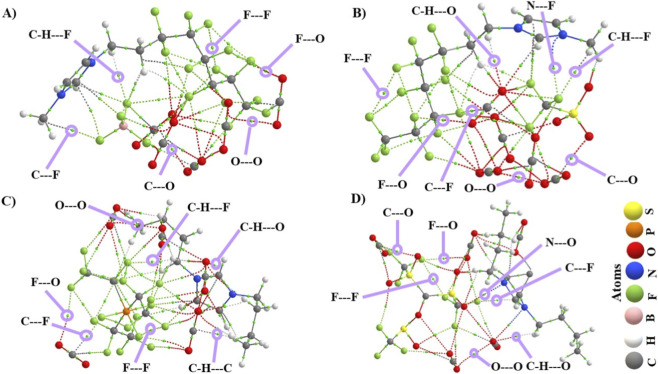
Molecular graphs of representative clusters. The dotted lines represent the binding paths for the different interactions, and their bond critical points (BCPs) are *highlighted* with purple circles: **(A)** 5CO_2_[C_8_H_4_F_13_mim]^+^[BF_4_]^−^, **(B)** 5CO_2_[C_8_H_4_F_13_mim]^+^[TFO]^−^, **(C)** 5CO_2_[Dbim]^+^[FAP]^−^ and **(D)** 5CO_2_[Dbim]^+^[Methide]^−^.

According to the molecular graphs (Figure 4; Supplementary Figures S3–S7 of the SM), it could be observed that in all clusters, fluorine interactions such as C---F, F---O, F---F, F---N, N---F, S---F, and C-H---F predominate, as expected due to the affinity of CO_2_ molecules for the areas containing this atom, as highlighted in the previous section and as observed in other studies such as that of Soniya et al. (Rao and Gejji, 2016). However, other types of interactions can be found in which fluorine is not involved, such as C---O, O---O, N---O, N---C, S---O, C-H---O, C-H---C, and C-H---S. These interactions occur depending on the IL and the number of CO_2_ molecules interacting on the cluster surface.

As for the anion-cation interactions, these vary depending on the cation and the anion that make up the IL. It can be noted that in most of the clusters, C---F, C---O, C-H---O, and C-H---F interactions are present. However, the clusters with the [Methide]^−^ anion that additionally present interactions with sulfur, such as S---F, S---O, and C-H---S, stand out. The ILs with the [C_8_H_4_F_13_mim]^+^ cation, which present F---F interactions, and the clusters with the [FAP]^−^ anion, which present C-H---C and F---F interactions, also stand out. Finally, in all the clusters, there are sporadic interactions with nitrogen, such as F---N and N---O interactions.

In the anion-CO_2_ interactions, all the studied clusters present C---F and F---O interactions, which was expected given the affinity of CO_2_ molecules for fluorine atoms. However, other types of interactions such as C---O, O---O, C-H---O, S---O, and N---O can be found, where the first three are mainly present in the clusters with [TFO]^−^ and [(PFBu)SO_3_]^−^ anions. The interaction with sulfur only occurs in the clusters with the [Methide]^−^ anion, and the interaction with nitrogen only occurs in the nCO_2_[C_8_H_4_F_13_mim]^+^[TFO]^−^, n = 2 and 3 clusters (see Supplementary Figures S5–S7 of the SM). The cation-CO_2_ interactions are mainly C---O, N---O, C-H---O, and C-H---C. Furthermore, in the clusters with the [C_8_H_4_F_13_mim]^+^ cation, C---F and C---O interactions are present, as well as C---C interactions in the clusters with the [(PFBu)SO_3_]^−^ and [(PFOc)SO_3_]^−^ anions. Finally, the CO_2_-CO_2_ interactions present in all clusters were O---O and C---O.

The nature of the interactions was determined through the values of five topological properties calculated over the BCPs: electron density ρ(r_cp_), Laplacian of the electron density ∇^2^ρ(r_cp_), electron energy density H(r_cp_), the ratio between the virial field |V(r_cp_)| and the kinetic energy density G(r_cp_) (|V(r_cp_)|/G(r_cp_)), and the interaction energy, I.E.,(r_cp_) which are tabulated in Supplementary Table S64 of the SM. These data show the average values per interaction type of the topological parameters analyzed, along with the abundance of each interaction type for all 60 clusters reported in this investigation.

Generally, it is observed that all interactions are of the van der Waals type, regardless of the type of interaction or the types of bonds formed, i.e., between anion and cation or between cation and CO_2_, etc. This is because, following the criterion of Rozas et al. (2000), it can be observed in Table 3 and Supplementary Table S64 of the SM that all values of ∇^2^ρ(r_cp_) > 0 and H(r_cp_) > 0 in anion-cation, anion-CO_2_, cation-CO_2_, and CO_2_-CO_2_ interactions. These values also show that the interactions are closed shell, since both ∇^2^ρ(r_cp_) and H(r_cp_) are positive, and the values of |V(r_cp_)|/G(r_cp_) are between 0 and 1 (Matta and Boyd, 2007).

**TABLE 3:** Average topological parameters of the different types of interactions. 1 = anion-cation, 2 = anion-CO_2_, 3 = cation-CO_2_, 4 = CO_2_-CO_2_. I = ∇^2^ρ(r_cp_) *10^−2^ a.u., II = H(r_cp_)*10^−3^ a.u., III = |V(r_cp_)|/G(r_cp_), formed in 5CO_2_-IL clusters.

IT	I	II	III	I	II	III	I	II	III	I	II	III
​	[Dbim]^+^ [FAP]^−^			[C_8_H_4_F_13_mim]^+^ [TFO]^−^			[Hmim]^+^ [FAP]^−^			[C_8_H_4_F_13_mim]^+^ [BF_4_]^−^		
1	4.72	2.20	0.78	3.71	1.72	0.77	4.30	1.97	0.77	3.66	1.57	0.79
2	3.08	1.33	0.78	3.13	1.39	0.78	3.40	1.42	0.79	4.76	1.68	0.84
3	2.18	1.07	0.75	2.91	1.37	0.76	2.13	1.01	0.75	2.61	1.24	0.76
4	4.13	1.87	0.78	3.29	1.54	0.76	2.08	1.05	0.74	3.32	1.56	0.76

IT	[Dbim]^+^ [Methide]^−^			[Hmim]^+^ [Methide]^−^			[Dmim]^+^ [BF_4_]^−^			[Omim]^+^ [(PFBu)SO_3_]^−^		
1	2.87	1.28	0.78	2.52	1.15	0.77	3.19	1.28	0.81	3.32	1.57	0.77
2	2.91	1.26	0.78	3.13	1.40	0.77	4.61	1.49	0.85	3.11	1.37	0.78
3	2.51	1.17	0.75	2.11	1.06	0.74	2.34	1.27	0.72	2.41	1.15	0.76
4	4.12	2.10	0.74	3.06	1.50	0.75	3.37	1.58	0.76	3.42	1.66	0.77

IT	[Dmim]^+^ [TFO]^−^			[Hmim]^+^ [(PFOc)SO_3_]^−^			[Omim]^+^ [(PFOc)SO_3_]^−^			[Hmim]^+^ [(PFBu)SO_3_]^−^		
1	3.47	1.57	0.78	3.18	1.48	0.77	4.23	2.05	0.77	3.32	1.52	0.78
2	3.48	1.37	0.81	2.80	1.28	0.77	2.57	1.17	0.77	2.77	1.28	0.77
3	2.35	1.26	0.73	2.26	1.13	0.75	2.29	1.15	0.74	1.79	0.93	0.73
4	3.2	1.65	0.74	2.86	1.36	0.76	3.19	1.49	0.76	3.99	1.79	0.78

When comparing individually, it can be observed that both ∇^2^ρ(r_cp_) and H(r_cp_) follow the same trends observed in ρ(r_cp_) in the order of all types of interactions. This was expected, since these two properties, together with the electron density, are very useful tools for determining the types and strengths of interactions. Furthermore, when studying the ratio |V(r_cp_)|/G(r_cp_), it can be noted that there are no notable variations in the values of this property for the different types of interactions. These generally remain between 0.7 a.u. and 0.8 a.u. All values are within the range described because this property does not determine the strength but describes the nature of the interactions (Bader, 1990).

**FIGURE 5:**
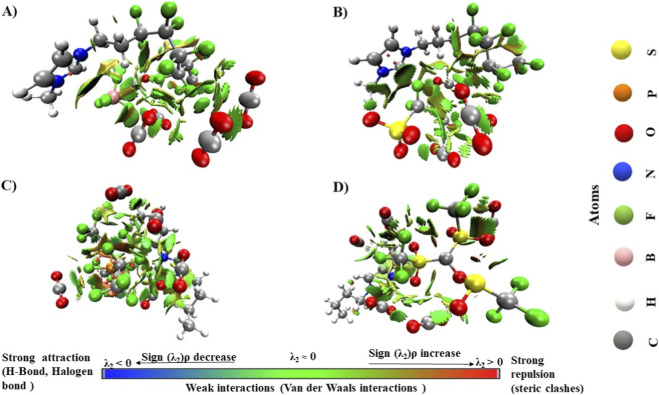
Representation of 3D isosurfaces of non-covalent interaction (NCI) analysis of representative molecular clusters. **(A)** 5CO_2_[C_8_H_4_F_13_mim]^+^[BF_4_]^−^, **(B)** 5CO_2_[C_8_H_4_F_13_mim]^+^[TFO]^−^, **(C)** 5CO_2_[Dbim]^+^[FAP]^−^, **(D)** 5CO_2_[Dbim]^+^[Methide]^−^.

The nature of the interactions can also be observed schematically through the NCI-3D plots, see Figure 5 and Supplementary Figures S8–S10 of the SM. For all the studied systems, attractive weak interactions are exhibited, as the isosurfaces are asymmetric and green, indicating that the stabilization of these types of systems is achieved solely via weak and van der Waals interactions.

Given the large number of structures and parameters analyzed, in this section, only the average values of electron density ρ(r_cp_) and interaction energy, I.E.,(r_cp_), together with the abundance of interactions of all the clusters studied with 5CO_2_ are shown graphically in Figures 6, 8. Additionally, for the four clusters with the strongest interactions with 5CO_2_, the average values of ρ(r_cp_) along with the abundance of interactions discriminating the different types of interactions are shown in Figure 7. For the smaller clusters, with CO_2_ to 4CO_2_, the same information can be found in Supplementary Figures S11–S47 of the SM.

**FIGURE 6:**
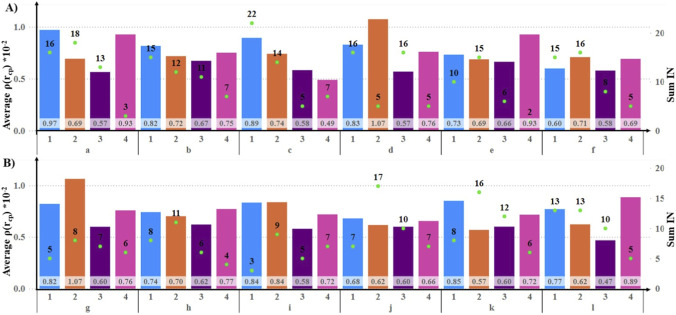
Average values of electron density ρ(rcp) and interactions number for the different types of interactions: 1 = anion-cation, 2 = anion-CO2, 3 = cation-CO2, 4 = CO2-CO2 for the clusters with 5CO2. Due to the graph’s size, it has been divided into two parts, **(A)** and **(B)**, where **(A)** includes clusters: a: [Dbim]+[FAP]−, b: [C8H4F13mim]+[TFO]−, c: [Hmim]+[FAP]−, d: [C8H4F13mim]+[BF4]−, e: [Dbim]+[Methide]−, f: [Hmim]+[Methide]− and B) continues with clusters: g: [Dmim]+[BF4]−, h: [Omim]+[(PFBu)SO3]−, i: [Dmim]+[TFO]−, j: [Hmim]+[(PFOc)SO3]−, k: [Omim]+[(PFOc)SO3]−, l: [Hmim]+[(PFBu)SO3]−.

**FIGURE 7:**
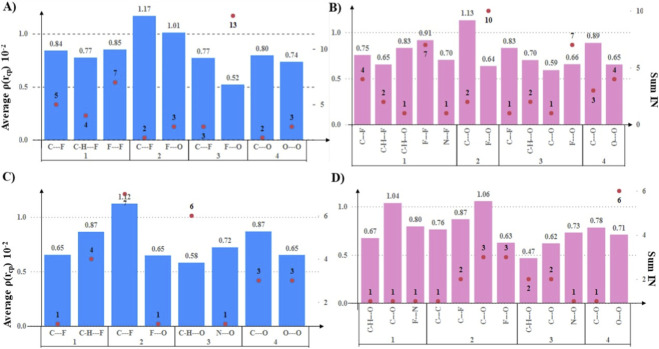
Mean values of the electron density ρ(r_cp_) and of the number of interactions (IN) for the different types of interactions: 1 = anion-cation, 2 = anion-CO_2_, 3 = cation-CO_2_, and 4 = CO_2_-CO_2_ per interaction class, where: **(A)** [C_8_H_4_F_13_mim]^+^[BF_4_]^−^, **(B)** [C_8_H_4_F_13_mim]^+^[TFO]^−^, **(C)** [Dmim]^+^[BF_4_]^−^, and **(D)** [Dmim]^+^[TFO]^−^.

The average values of the electron density ρ(r_cp_) for the different types of interactions, anion-cation, anion-CO_2_, cation-CO_2_, and CO_2_-CO_2_, do not allow for observing an established order in the strength of this type of interactions. Instead, the order varies depending on the cluster being analyzed, as shown in Figure 6 and Supplementary Figures S11–S14 of the SM. However, it is possible to establish that in most cases, the cation-CO_2_ interactions are the least strong, except for CO_2_[Dmim]^+^[TFO]^−^, CO_2_[Omim]^+^[(PFBu)SO_3_]^−^, 2CO_2_[Hmim]^+^[(PFOc)SO_3_]^−^, 2CO_2_[Hmim]^+^[(PFOc)SO_3_]^−^, 2CO_2_[Hmim]^+^[FAP]^−^, 2CO_2_[Omim]^+^[(PFBu)SO_3_]^−^, 3CO_2_[C_8_H_4_F_13_mim]^+^[BF_4_]_−_, 3CO_2_[Dbim]^+^[FAP]^−^, 3CO_2_[Hmim]^+^[(PFBu)SO_3_]^−^, 3CO_2_[Omim]^+^[(PFBu)SO_3_]^−^, 5CO_2_[Hmim]^+^[FAP]^−^, and 5CO_2_[Omim]^+^[(PFOc)SO_3_]^−^. This would indicate that, for most cases, anion-CO_2_ interactions are stronger than cation-CO_2_ interactions, which would explain the trend described in the potential energy surface (PES) scan regarding the location of CO_2_ preferentially over the anion sites of the different ILs.

If analyzed specifically, it is possible to determine that in the clusters with nCO_2_[C_8_H_4_F_13_mim]^+^[BF_4_]^−^, the strongest interactions are the anion-CO_2_, since they are the ones that present the highest average ρ(r_cp_), except for the cluster with 2CO_2_ see Supplementary Figure S12 of the SM. In general, it can be observed that the strength of the interactions for these clusters stabilizes in the following order: anion-CO_2_ > anion-cation > CO_2_-CO_2_ > cation-CO_2_. In the nCO_2_[Dmim]^+^[BF_4_]^−^ clusters, similar behavior is observed, these two being the only clusters in which the CO_2_ physisorption process anion-CO_2_ interactions are the strongest.

Another important point to highlight is that contrary to expectations, the ρ(r_cp_) values of the cation-CO_2_ interactions in clusters with [C_8_H_4_F_13_mim]^+^ have similar or even lower values than those presented in clusters without the addition of fluorine atoms to the cation chains. This would indicate that the addition of halogens to the cation structure does not contribute to the strengthening of ILs-CO_2_ interactions, especially cation-CO_2_ interactions. However, as demonstrated in studies by Faisal Elmobarak et al. (2023) and Kohno et al. (2022), the addition of such compounds to the cation chains enhances the permeability of these compounds, facilitating the formation of new interactions with both the anion and CO_2_ molecules. This is exemplified by the clusters [C_8_H_4_F_13_mim]^+^[TFO]^−^ and [C_8_H_4_F_13_mim]^+^[BF_4_]^−^, which exhibit improved CO_2_ solubility despite the lack of increased strength in ILs-CO_2_ interactions.

For the clusters nCO_2_[C_8_H_4_F_13_mim]^+^[TFO]^−^, nCO_2_[Dbim]^+^[FAP]^−^, and nCO_2_[Hmim]^+^[FAP]^−^, the order of strength stabilizes as anion-cation > CO_2_-CO_2_ > anion-CO_2_ > cation-CO_2_. This order is reached from the cluster with CO_2_ in nCO_2_[Dbim]^+^[FAP]^−^ and nCO_2_[Hmim]^+^[FAP]^−^ except for 4CO_2_[Hmim]^+^[FAP]^−^ (see Figure 6; Supplementary Figures S12–S14 of the SM), while for nCO_2_[C_8_H_4_F_13_mim]^+^[TFO]^−^, it is reached at 3CO_2_. In the clusters nCO_2_[Dbim]^+^[Methide]^−^, nCO_2_[Hmim]^+^[Methide]^−^, and nCO_2_[Hmim]^+^[(PFOc)SO_3_]^−^, the order of strength stabilizes as CO_2_-CO_2_ > anion-cation > anion-CO_2_ > cation-CO_2_. For nCO_2_[Dbim]^+^[Methide]^−^, this is reached at 3CO_2_; in nCO_2_[Hmim]^+^[Methide]^−^ with CO_2_ except for 5CO_2_[Hmim]^+^[Methide]^−^; and with 2CO_2_ in nCO_2_[Hmim]^+^[(PFOc)SO_3_]^−^. Finally, in nCO_2_[Hmim]^+^[(PFBu)SO_3_]^−^, the order stabilizes as CO_2_-CO_2_ > anion-CO_2_ > anion-cation > cation-CO_2_, and this order is reached with 2CO_2_. This shows that intramolecular interactions present greater strengths than IL-CO_2_ interactions in this case.

Finally, the clusters nCO_2_[Dmim]^+^[TFO]^−^, nCO_2_[Omim]^+^[(PFBu)SO_3_]^−^, and nCO_2_[Omim]^+^[(PFOc)SO_3_]^−^ are presenting high variation in the order of strength of the anion-cation interactions, CO_2_-CO_2_, and anion-CO_2_ as CO_2_ molecules are added (see Figure 6; Supplementary Figures S12–S14), which does not allow establishing a clear order as it has been established for the rest of the studied clusters. However, perhaps with the inclusion of more CO_2_ molecules, this order will stabilize. As for the value of the average electron densities, these vary from 0.23 × 10^−2^ a.u. to 1.58 × 10^−2^ a.u., with the C---F, C---O, F---F, and F---O interactions being the strongest ones (see Figure 7; Supplementary Figures S15–S28 of the SM), with values ranging from 1.0 × 10^−2^ a.u. to 1.58 × 10^−2^ a.u. F---F interactions are those that reach the maximum value of 1.58 × 10^−2^ a.u. in 5CO_2_[Hmim]^+^[(PFBu)SO_3_]^−^. It is important to note that these types of interactions occur mainly in the anion-cation and anion-CO_2_ types. However, it can be observed in Figure 7 and Supplementary Figures S15–S28 of the SM, that these types of interactions have the lowest abundance, while the lower strength interactions present a higher abundance. Thus, it can be concluded that the stabilization of these types of processes does not only depend on the strength of the interactions, but the abundance of the interactions also plays an important role.

Another result that could be deduced from Figure 6 and Supplementary Figures S12–S14 of the SM is that both the anion and cation that make up the IL, as well as their characteristics, have great relevance on the strengths and abundances of the interaction types. For example, it is observed that the average strength of the interactions in the different types (anion-cation, anion-CO_2_, cation-CO_2_, and CO_2_-CO_2_) is approximately equal in the clusters nCO_2_[C_8_H_4_F_13_mim]^+^[BF_4_]^−^, nCO_2_[C_8_H_4_F_13_mim]^+^[TFO]^−^, nCO_2_[Dmim]^+^[BF_4_]^−^, and nCO_2_[Dmim]^+^[TFO]^−^. However, there is a large difference in the abundance of interactions in the clusters, especially between anion-cation and cation-CO_2_ interactions, which are more abundant in clusters with [C_8_H_4_F_13_mim]^+^ cations than in clusters with the [Dmim]^+^ cation. This indicates that, although the addition of fluorine atoms on the [C_8_H_4_F_13_mim]^+^ cation increases the affinity of CO_2_ for the cation and thus increases the abundance of cation-CO_2_ interactions, the interactions generated are not as strong as the interactions with the anion and are comparable to those of clusters with smaller carbon chains and no fluorine substituents. However, this is not detrimental, as previously discussed, this increased affinity for both CO_2_ molecules and anions results in enhanced permeability and, consequently, solubility, as demonstrated in the study of Almantariotis et al. (2010), who showed that in their experimental study with [C_8_mim]^+^[NtF_2_]^−^, [C_10_mim]^+^[NtF_2_]^−^, [C_8_H_4_F_13_mim]^+^[NtF_2_]^−^, substitution with halogens on the alkyl chain of the imidazolium cation lead to a constant increase in gaseous uptake by IL.

Similarly, when comparing the clusters with [Dbim]^+^ and [Hmim]^+^ cations, in nCO_2_[Dbim]^+^[FAP]^−^ and nCO_2_[Hmim]^+^[FAP]^−^, the strengths of the interactions do not vary much between clusters. What differentiates them is the abundance of interactions, with the clusters with [Dbim]^+^[FAP]^−^ having the highest abundance, except at CO_2_. However, if the [Dbim]^+^[Methide]^−^ and [Hmim]^+^[Methide]^−^ clusters are compared, it can be noted that, in this case, the clusters with the [Hmim]^+^[Methide]^−^ IL present interactions with higher strengths and higher abundance than those presented with [Dbim]^+^[Methide]^−^. In both cases, the strongest interactions resulted in the clusters with asymmetric cations. Therefore, it can be concluded that the symmetry in the substituents influences the CO_2_ capture process because, although it causes the CO_2_ to be located uniformly over the vicinity of the chains that substitute the imidazole ring and, therefore, form a greater number of interactions, these interactions are not as strong. This would indicate that the strength of IL-CO_2_ interactions benefits when fewer of these molecules interact with the cation. However, as observed in these and previous cases, clusters tend to be more stable as the number of interactions increases.

In the clusters with the [Hmim]^+^ and [Omim]^+^ cations it can be noted that those with the [Omim]^+^ cation in their structures have the highest abundance and strength of interactions. From this, it can be concluded that although cations with asymmetric chains show the strongest interactions, as described above, the length of the chains also influences the abundance and strength of the interactions. Therefore, the longer one of the chains substituting the imidazole ring, the stronger the strength of the interactions.

On the other hand, when evaluating the anions, it could be determined that the inorganic [BF_4_]^−^ anion favors the strength of the anion-CO_2_ interactions (see Figure 6; Supplementary Figures S12–S14 of the SM), while in the organic anions, the strength of the anion-cation and CO_2_-CO_2_ interactions is favored. Considering organic anions with sulfonates, it can be observed that the longer and more fluorine-substituted the SO_3_^−^ substituted chains, the higher the abundance of the interactions, but the lower their strength. The addition of more sulfonates to the anion, as in the case of the clusters with [Methide]^−^, slightly improves the strength of the interactions compared to the clusters with the [(PFBu)SO_3_]^−^ (6 carbons) and [(PFOc)SO_3_]^−^ (8 carbons) anions, but does not make them greater than those present in the [TFO]^−^ anion, which is the one with the smaller SO_3_^−^ substituted chain (1 carbon). Of the clusters with organic anions, the one with the highest strength and abundance is the one with the [FAP]^−^ anion, which was to be expected, due to this anion having the highest amount of fluorine in its structure and, therefore, CO_2_ has a high affinity for these areas of the molecule. However, although it has the highest strength of interactions in organic clusters, the strength is no greater than that present with the inorganic anion.

In terms of saturation, i.e., the maximum amount of CO_2_ that can be absorbed by the ILs, the abundance and strength of the interactions can be considered to increase with the number of CO_2_ molecules. Regarding the abundance, it increases with the number of CO_2_ molecules for all types of interactions except anion-cation interactions and for all ILs studied. However, CO_2_-CO_2_ interactions have the lowest abundance of all interactions studied, while IL-CO_2_ interactions have the highest abundance, as is evident from Figures 6, 7 and Supplementary Figures S12–S14 of the SM. This suggests that saturation does not occur up to 5CO_2_, as CO_2_ still prefers to interact with either the anion or the cation of the ILs.

Saturation does not happen. However, the strength of the interactions can indicate some trends in how this process will occur. As demonstrated in the study by Sistla et al. (Sistla and Sridhar, 2021), the strength of the interactions determines the interstitial molecular spaces that allow CO_2_ molecules to be accommodated within the clusters. These spaces are smaller, and therefore, more CO_2_ molecules can be accommodated when the anion-cation interactions are weaker, and the IL-CO_2_ interactions are stronger. Among the clusters studied, as observed above, only two ILs ([C_8_H_4_F_13_mim]^+^[BF_4_]^−^ and [Dmim]^+^[BF_4_]^−^) exhibit stronger IL-CO_2_ interactions (specifically anion-CO_2_ interactions). Consequently, if this trend continues, it is estimated that these clusters have a greater chance of absorbing a higher number of CO_2_ molecules compared to the following three ILs: [C_8_H_4_F_13_mim]^+^[TFO]^−^, [Dbim]^+^[FAP]^−^, and [Hmim]^+^[FAP]^−^, for which anion-cation interactions are presented as the strongest interactions. Therefore, it is estimated that these clusters will reach saturation with a lower number of CO_2_ molecules.

**FIGURE 8:**
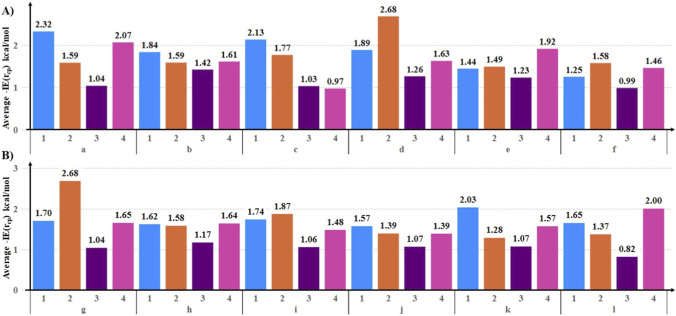
Interaction energy values IE(rcp) for the different types of interactions: 1 = anion-cation, 2 = anion-CO2, 3 = cation-CO2, 4 = CO2-CO2 for the clusters with 5CO2. Due to the graph’s size, it has been divided into two parts, **(A)** and **(B)**, where **(A)** includes clusters: a: [Dbim]+[FAP]−,b: [C8H4F13mim]+[TFO]−,c: [Hmim]+[FAP]−, d: [C8H4F13mim]+[BF4]−, e: [Dbim]+[Methide]−, f: [Hmim]+[Methide]− and B) continues with clusters: g: [Dmim]+[BF4]−, h: [Omim]+[(PFBu)SO3]−, i: [Dmim]+[TFO]−, j: [Hmim]+[(PFOc)SO3]−, k: [Omim]+[(PFOc)SO3]−, l: [Hmim]+[(PFBu)SO3]−.

The interaction energy, I.E.,(r_cp_), obtained from the Espinosa-Molins-Lecomte approximation (Espinosa et al., 1998), follows the same trends as ρ(r_cp_) (see Figures 6–8). This is positive because it confirms the conclusions regarding the orders of strength of the interaction types in the different clusters, as determined with ρ(r_cp_). Another remarkable aspect found in the analysis of this property is that most of the interactions have an energy in the range of 0.2–2.0 kcal/mol, except for the strongest interactions (C---F, C---O, F---F, and F---O), which can exceed 3.0 kcal/mol (see Figure 8; Supplementary Figures S29–S47 of the SM). This energy sequence confirms, as observed in the 3D isosurfaces of the non-covalent interaction (NCI) analysis, that the interactions are weak and primarily of the van der Waals type, with a highly dispersive character. It is, therefore, concluded that CO_2_ absorption by ILs occurs through physisorption.

Based on the previous results, it can be concluded that by analyzing the strength and frequency of the interactions, the most suitable combinations of anions and cations that would favor the CO_2_ capture process are those in which both the frequency and strength of the IL-interactions are prioritized, but most importantly, the abundance of interactions should be prioritized.

### 3.4 Electrostatic potential maps

**FIGURE 9:**
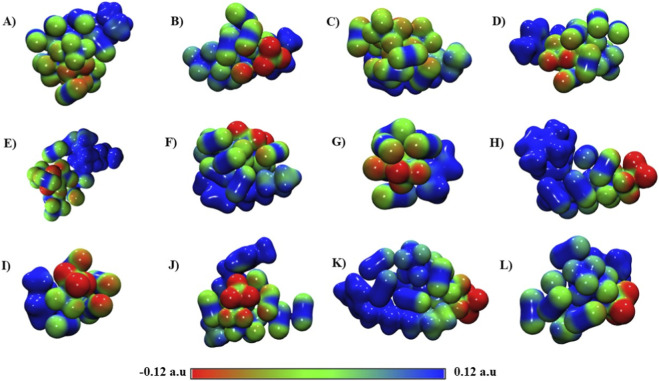
Electrostatic potential maps for 5CO_2_-IL. **(A)** [Dbim]^+^[FAP]^−^, **(B)** [C_8_H_4_F_13_mim]^+^[TFO]^−^, **(C)** [Hmim]^+^[FAP]^−^, **(D)** [C_8_H_4_F_13_mim]^+^[BF_4_]^−^, **(E)** [Dbim]^+^[Methide]^−^, **(F)** [Hmim]^+^[Methide]^−^, **(G)** [Dmim]^+^[BF_4_]^−^, **(H)** [Omim]^+^[(PFBu)SO_3_]^−^, **(I)** [Dmim]^+^[TFO]^−^, **(J)** [Hmim]^+^[(PFOc)SO_3_]^−^, **(K)** [Omim]^+^[(PFOc)SO_3_]^−^, L) [Hmim]^+^[(PFBu)SO_3_]^−^. All isosurfaces were generated at 0.01 a.u.

To characterize the charge distribution of different systems, a mapping method known as Electrostatic Potential Mapping (EPM) is used, in which a color scale can be used to determine the areas in which the systems have a deficiency or excess of electron density. The red areas correspond to the maximum negative potentials, while the blue areas correspond to the maximum positive potentials (Fernández Vega et al., 2018; Murray et al., 1994; VEGA RODRÍGUEZ, 2022). The MPEs of the clusters with 5CO_2_ are shown in Figure 9, while the maps of the clusters with CO_2_ to 4CO_2_ can be found in Supplementary Figures S48–S50 of the SM.

The EPMs provide information about the intermolecular interactions between ILs and CO_2_ molecules, we are analyzing clusters where physisorption processes take place. The surface charge values are represented by different colors and the potentials increase in the following order: red < orange < green < blue. In all clusters, as expected, the negative potentials are mainly associated with the anions of the ILs, centered on the oxygen and fluorine atoms, and partly with the oxygens of the CO_2_ molecules. On the other hand, positive potentials are mainly concentrated on the cations, on the carbon atoms and partly on the carbon atoms of the CO_2_ molecules. This potential explains the tendency of the CO_2_ molecules to be in the anion regions, since these regions have a higher reactivity. It also explains why, since CO_2_, which has both positive and negative potentials, is attracted by both the anions and cations of the different ILs, but mainly by the anions, the most stable clusters obtained from the PES are those in which the CO_2_ molecules are located between the anion and the cation.

The negative potentials, concentrated mainly on the fluorine and oxygen atoms of the anions, also explain why in most clusters C---F and C---O interactions form between the anion and the CO_2_ and why these interactions are the strongest. This also explains why C---O interactions are important in the types of interactions that form between the cation and CO_2_.

It is important to note that in the [C_8_H_4_F_13_mim]^+^ cation clusters, the addition of fluorine to the cation substituent chain turns the potential over the fluorine atoms from blue to green, increasing the reactivity of the cation and its affinity for CO_2_. However, since this addition does not make the electrostatic potential negative, the QTAIM analysis confirms the observation that the number of cation-CO_2_ interactions is increased, but the strengths of these interactions are almost equal to or sometimes lower than those in clusters without substitution. Furthermore, when the other cations are analyzed, the longer the chains substituting the imidazole ring, the more negative the potential on the cations become.

It should also be noted that although the negative potentials are mainly concentrated on the anions, the intensity of the color of these surfaces varies according to the type of anion forming the cluster. The clusters with inorganic anions have the surfaces with the most negative potential, which is concentrated more or less uniformly on the whole molecule, especially on the fluorine atoms. On the contrary, in the clusters with organic anions, which have sulphonates in their structure, the negative potential is concentrated mainly on the oxygen atoms of SO_3_^−^. In the flouroalkane chains that replace SO_3_^−^, the potential is seen to be between orange and green. Finally, for the [FAP]^−^ anion, the potential is evenly distributed between orange and green throughout the molecule and the orange surfaces are mainly concentrated on the fluorine atoms. These results confirm what was observed in the QTAIM analysis, that the clusters with inorganic anions have the strongest anion-CO_2_ interactions compared to the organic clusters, and also confirm why the anion-CO_2_ interactions in the clusters with organic anions are stronger in those where the chains replacing the SO_3_^−^ are shorter, since they do not suffer as much from the influence of the positive potential appearing on the carbon atoms, and the longer this chain is, the greater the influence of this positive potential will be.

### 3.5 Comparison with previous studies on one-to-one IL–CO_2_ complexes

Our study on nCO_2_ (n = 1–5) capture by imidazole and fluorine-based ionic liquids significantly extends previous research by systematically exploring multiple CO_2_ molecule interactions (Alvarez Becerra et al., 2022; Mercy et al., 2016; Torkzadeh and Moosavi, 2022). While numerous studies have focused on single CO_2_ molecule interactions with ionic liquids, our work provides a comprehensive analysis of how increasing CO_2_ molecules affects the capture mechanism and energetics. Previous single-molecule studies, such as those using DFT calculations with IEFPCM-SMD solvation models for [EMIM][BF4], have shown that CO_2_ is primarily stabilized through interactions with anions, with binding energies typically ranging from −2 to −4 kcal/mol (Awoke, 2024; Dhar, 2019). Our findings align with these observations but further demonstrate that as more CO_2_ molecules are added, the interaction patterns evolve significantly (Torkzadeh and Moosavi, 2022). The literature shows that for single CO_2_ molecules, van der Waals interactions dominate the physisorption process, with C---F and C---O interactions being particularly important (Ahsan et al., 2024; Torkzadeh and Moosavi, 2024). Our work confirms these findings while revealing that with multiple CO_2_ molecules, the abundance and distribution of these interactions change substantially. Studies on dicationic ionic liquids have shown that symmetry and alkyl chain length in cations significantly impact CO_2_ capture performance even at the single-molecule level (Torkzadeh and Moosavi, 2022; 2024). Our research extends this understanding by demonstrating how these structural features influence the capture of multiple CO_2_ molecules. By comparing our results with previous single-molecule studies, we provide valuable insights into the cooperative effects and saturation behavior that cannot be observed in simpler models. This comprehensive approach bridges the gap between fundamental single-molecule interactions and more realistic multi-molecule capture scenarios, offering a more complete picture of CO_2_ capture mechanisms by ionic liquids.

## 4 Conclusion

The nature of the interactions between 12 ionic liquids and CO_2_, as well as the change in these interactions as more CO_2_ molecules are added to the clusters, has been studied theoretically using semi-empirical and DFT calculations. By scanning the potential energy surface, it was found that CO_2_ molecules tend to localize between the anion and cation of the ionic liquids, with a preference for the anion. The side chains replacing the imidazole of the cation influence this localization. The addition of fluorine atoms increases the affinity of the sites to which CO_2_ molecules are added. Cluster formation is exothermic and non-spontaneous and becomes more exothermic and less spontaneous with the addition of CO_2_ molecules. It was found that none of the clusters show saturation up to a certain point, but it is suggested that some will eventually reach saturation with a lower number of CO_2_ molecules than others. Cluster stability is dependent on the number of CO_2_ molecules being evaluated; however, in 3 out of the 5 cases studied, the [Dbim]^+^[FAP]^−^ cluster was found to be the most stable. Additionally, it was observed that the formation energies of the clusters were neither enhanced nor weakened by the number of CO_2_ molecules present. The clusters are stabilized by weak van der Waals interactions, with fluorine interactions predominating. As shown by analyzing the 3D isosurfaces produced by the Non-Covalent Interactions index. Fluorine interactions predominate, such as C---F, F---O, F---F, F---N, N---F, S---F and C-H---F. There are also other types of interactions that do not involve fluorine such as C---O, O---O, N---O, N---C, S---O, C-H---O, C-H---C and C-H---S. The nature of the interactions varies according to the cluster studied, with cation-CO_2_ interactions generally being of lower strength. Asymmetric cations present the strongest interactions, while inorganic anions favor the strength of anion-CO_2_ interactions. It is observed that the abundance of interactions plays a fundamental role in the stabilization of clusters and, consequently, in the solubility of CO_2_ in ILs. Finally, the QTAIM analysis suggests that the most suitable combinations of anions and cations for CO_2_ capture are those that priorities both the abundance and strength of IL-CO_2_ interactions. Therefore, [Dbim]^+^[FAP]^−^, [C_8_H_4_F_13_mim]^+^[BF_4_]^−^ and [C_8_H_4_F_13_mim]^+^[TFO]^−^ ILs are suggested as the most promising for CO_2_ capture.

Finally, beyond their capacity for reversible CO_2_ physisorption, some of the ILs studied, especially those containing basic or nucleophilic groups, may also serve as organocatalysts for the fixation of CO_2_ into industrially relevant products such as cyclic carbonates, polycarbonates, or isocyanate-free polyurethanes. Although this catalytic potential was not explored in the present work, it represents a natural and promising extension of our findings. Further studies combining mechanistic modeling and reaction energetics will be necessary to evaluate such catalytic activity in detail.

## Data Availability

The original contributions presented in the study are included in the article/Supplementary Material, further inquiries can be directed to the corresponding authors.
