# Supplementary material for: CO2 capture by fluorinated-imidazolium based ionic liquids: a multiple minima hypersurfaces analysis
Source: Front Chem. 2026 May 28;14:1820345. doi: 10.3389/fchem.2026.1820345 (PMC13254104; doi:10.3389/fchem.2026.1820345)
Supplement: Supplementary file 1 [file DataSheet1.pdf]

Supplementary Material

CO_2 Capture by Fluorinated-Imidazolium Based Ionic Liquids: A Multiple Minima Hypersurfaces Analysis

Jennifer Cuellar[1,2], Osvaldo Yáñez[3,*], and Sol M. Mejía[1,*]

[1] Línea de Investigación en Química Computacional, Grupo de Investigación GIFUJ, Departamento de Química, Facultad de Ciencias, Pontificia Universidad Javeriana, Bogotá 110231, Colombia. E-mail: sol.mejia@javeriana.edu.co

[2] Departamento de Ciencias Básicas y Modelado. Facultad de Ciencias Naturales e ingeniería. Universidad Jorge Tadeo Lozano.

[3] Centro de Modelación Ambiental y Dinámica de Sistemas (CEMADIS), Facultad de Ingeniería y Negocios, Universidad de Las Américas, Santiago 7500975, Chile. E-mail: oyanez@udla.cl

* Authors to whom correspondence should be addressed.

Description

This Supplementary Material includes all optimized geometries for CO_2–ionic liquid clusters (n = 1–5), detailed interaction and formation energies, additional PES analyses, QTAIM and NCI plots, extended tables, and supporting figures. These resources complement the main text by providing full computational data and ensuring reproducibility of the results.

Content

TABLES

Table S1. Experimental CO_2 uptake capacities of representative ionic liquids.

Table S2. Number of structures obtained in the PES analysis by optimization with the PM7 semi-empirical method and reoptimization with the M06-2X-D3/6-31G(d,p) approach.

Table S3. Representations of lowest energy forms for $1CO_2[C_8H_4F_{13}mim]^+[BF_4]^-$ at the M06-2X-D3/6-31G(d,p) level with an implicit solvent model PCM. Relative energies are listed in kcal/mol.

Table S4. Representations of lowest energy forms for $2CO_2[C_8H_4F_{13}mim]^+[BF_4]^-$ at the M06-2X-D3/6-31G(d,p) level with an implicit solvent model PCM. Relative energies are listed in kcal/mol.

Table S5. Representations of lowest energy forms for $3CO_2[C_8H_4F_{13}mim]^+[BF_4]^-$ at the M06-2X-D3/6-31G(d,p) level with an implicit solvent model PCM. Relative energies are listed in kcal/mol.

Table S6. Representations of lowest energy forms for $4CO_2[C_8H_4F_{13}mim]^+[BF_4]^-$ at the M06-2X-D3/6-31G(d,p) level with an implicit solvent model PCM. Relative energies are listed in kcal/mol.

Table S7. Representations of lowest energy forms for $5CO_2[C_8H_4F_{13}mim]^+[BF_4]^-$ at the M06-2X-D3/6-31G(d,p) level with an implicit solvent model PCM. Relative energies are listed in kcal/mol.

Table S8. Representations of lowest energy forms for $1CO_2[C_8H_4F_{13}mim]^+[TFO]^-$ at the M06-2X-D3/6-31G(d,p) level with an implicit solvent model PCM. Relative energies are listed in kcal/mol.

Table S9. Representations of lowest energy forms for $2CO_2[C_8H_4F_{13}mim]^+[TFO]^-$ at the M06-2X-D3/6-31G(d,p) level with an implicit solvent model PCM. Relative energies are listed in kcal/mol.

Table S10. Representations of lowest energy forms for $3CO_2[C_8H_4F_{13}mim]^+[TFO]^-$ at the M06-2X-D3/6-31G(d,p) level with an implicit solvent model PCM. Relative energies are listed in kcal/mol.

Table S11. Representations of lowest energy forms for $4CO_2[C_8H_4F_{13}mim]^+[TFO]^-$ at the M06-2X-D3/6-31G(d,p) level with an implicit solvent model PCM. Relative energies are listed in kcal/mol.

Table S12. Representations of lowest energy forms for $5CO_2[C_8H_4F_{13}mim]^+[TFO]^-$ at the M06-2X-D3/6-31G(d,p) level with an implicit solvent model PCM. Relative energies are listed in kcal/mol.

Table S13. Representations of lowest energy forms for $1CO_2[Dmim]^+[BF_4]^-$ at the M06-2X-D3/6-31G(d,p) level with an implicit solvent model PCM. Relative energies are listed in kcal/mol.

Table S14. Representations of lowest energy forms for $2CO_2[Dmim]^+[BF_4]^-$ at the M06-2X-D3/6-31G(d,p) level with an implicit solvent model PCM. Relative energies are listed in kcal/mol.

Table S15. Representations of lowest energy forms for $3CO_2[Dmim]^+[BF_4]^-$ at the M06-2X-D3/6-31G(d,p) level with an implicit solvent model PCM. Relative energies are listed in kcal/mol.

Table S16. Representations of lowest energy forms for $4CO_2[Dmim]^+[BF_4]^-$ at the M06-2X-D3/6-31G(d,p) level with an implicit solvent model PCM. Relative energies are listed in kcal/mol.

Table S17. Representations of lowest energy forms for $5CO_2[Dmim]^+[BF_4]^-$ at the M06-2X-D3/6-31G(d,p) level with an implicit solvent model PCM. Relative energies are listed in kcal/mol.

Table S18. Representations of lowest energy forms for $1CO_2[Dmim]^+[TFO]^-$ at the M06-2X-D3/6-31G(d,p) level with an implicit solvent model PCM. Relative energies are listed in kcal/mol.

Table S19. Representations of lowest energy forms for $2CO_2[Dmim]^+[TFO]^-$ at the M06-2X-D3/6-31G(d,p) level with an implicit solvent model PCM. Relative energies are listed in kcal/mol.

Table S20. Representations of lowest energy forms for $3CO_2[Dmim]^+[TFO]^-$ at the M06-2X-D3/6-31G(d,p) level with an implicit solvent model PCM. Relative energies are listed in kcal/mol.

Table S21. Representations of lowest energy forms for $4CO_2[Dmim]^+[TFO]^-$ at the M06-2X-D3/6-31G(d,p) level with an implicit solvent model PCM. Relative energies are listed in kcal/mol.

Table S22. Representations of lowest energy forms for $5CO_2[Dmim]^+[TFO]^-$ at the M06-2X-D3/6-31G(d,p) level with an implicit solvent model PCM. Relative energies are listed in kcal/mol.

Table S23. Representations of lowest energy forms for $1CO_2[Dbim]^+[FAP]^-$ at the M06-2X-D3/6-31G(d,p) level with an implicit solvent model PCM. Relative energies are listed in kcal/mol.

Table S24. Representations of lowest energy forms for $2CO_2[Dbim]^+[FAP]^-$ at the M06-2X-D3/6-31G(d,p) level with an implicit solvent model PCM. Relative energies are listed in kcal/mol.

Table S25. Representations of lowest energy forms for $3CO_2[Dbim]^+[FAP]^-$ at the M06-2X-D3/6-31G(d,p) level with an implicit solvent model PCM. Relative energies are listed in kcal/mol.

Table S26. Representations of lowest energy forms for $4CO_2[Dbim]^+[FAP]^-$ at the M06-2X-D3/6-31G(d,p) level with an implicit solvent model PCM. Relative energies are listed in kcal/mol.

Table S27. Representations of lowest energy forms for $5CO_2[Dbim]^+[FAP]^-$ at the M06-2X-D3/6-31G(d,p) level with an implicit solvent model PCM. Relative energies are listed in kcal/mol.

Table S28. Representations of lowest energy forms for $1CO_2[Dbim]^+[Methide]^-$ at the M06-2X-D3/6-31G(d,p) level with an implicit solvent model PCM. Relative energies are listed in kcal/mol.

Table S29. Representations of lowest energy forms for $2CO_2[Dbim]^+[Methide]^-$ at the M06-2X-D3/6-31G(d,p) level with an implicit solvent model PCM. Relative energies are listed in kcal/mol.

Table S30. Representations of lowest energy forms for $3CO_2[Dbim]^+[Methide]^-$ at the M06-2X-D3/6-31G(d,p) level with an implicit solvent model PCM. Relative energies are listed in kcal/mol.

Table S31. Representations of lowest energy forms for $4CO_2[Dbim]^+[Methide]^-$ at the M06-2X-D3/6-31G(d,p) level with an implicit solvent model PCM. Relative energies are listed in kcal/mol.

Table S32. Representations of lowest energy forms for $5CO_2[Dbim]^+[Methide]^-$ at the M06-2X-D3/6-31G(d,p) level with an implicit solvent model PCM. Relative energies are listed in kcal/mol.

Table S33. Representations of lowest energy forms for $1CO_2[Hmim]^+[FAP]^-$ at the M06-2X-D3/6-31G(d,p) level with an implicit solvent model PCM. Relative energies are listed in kcal/mol.

Table S34. Representations of lowest energy forms for $2CO_2[Hmim]^+[FAP]^-$ at the M06-2X-D3/6-31G(d,p) level with an implicit solvent model PCM. Relative energies are listed in kcal/mol.

Table S35. Representations of lowest energy forms for $3CO_2[Hmim]^+[FAP]^-$ at the M06-2X-D3/6-31G(d,p) level with an implicit solvent model PCM. Relative energies are listed in kcal/mol.

Table S36. Representations of lowest energy forms for $4CO_2[Hmim]^+[FAP]^-$ at the M06-2X-D3/6-31G(d,p) level with an implicit solvent model PCM. Relative energies are listed in kcal/mol.

Table S37. Representations of lowest energy forms for $5CO_2[Hmim]^+[FAP]^-$ at the M06-2X-D3/6-31G(d,p) level with an implicit solvent model PCM. Relative energies are listed in kcal/mol.

Table S38. Representations of lowest energy forms for $1CO_2[Hmim]^+[Methide]^-$ at the M06-2X-D3/6-31G(d,p) level with an implicit solvent model PCM. Relative energies are listed in kcal/mol.

Table S39. Representations of lowest energy forms for $2CO_2[Hmim]^+[Methide]^-$ at the M06-2X-D3/6-31G(d,p) level with an implicit solvent model PCM. Relative energies are listed in kcal/mol.

Table S40. Representations of lowest energy forms for $3CO_2[Hmim]^+[Methide]^-$ at the M06-2X-D3/6-31G(d,p) level with an implicit solvent model PCM. Relative energies are listed in kcal/mol.

Table S41. Representations of lowest energy forms for $4CO_2[Hmim]^+[Methide]^-$ at the M06-2X-D3/6-31G(d,p) level with an implicit solvent model PCM. Relative energies are listed in kcal/mol.

Table S42. Representations of lowest energy forms for $5CO_2[Hmim]^+[Methide]^-$ at the M06-2X-D3/6-31G(d,p) level with an implicit solvent model PCM. Relative energies are listed in kcal/mol.

Table S43. Representations of lowest energy forms for $1CO_2[Hmim]^+[(PFOc)SO_3]^-$ at the M06-2X-D3/6-31G(d,p) level with an implicit solvent model PCM. Relative energies are listed in kcal/mol.

Table S44. Representations of lowest energy forms for $2CO_2[Hmim]^+[(PFOc)SO_3]^-$ at the M06-2X-D3/6-31G(d,p) level with an implicit solvent model PCM. Relative energies are listed in kcal/mol.

Table S45. Representations of lowest energy forms for $3CO_2[Hmim]^+[(PFOc)SO_3]^-$ at the M06-2X-D3/6-31G(d,p) level with an implicit solvent model PCM. Relative energies are listed in kcal/mol.

Table S46. Representations of lowest energy forms for $4CO_2[Hmim]^+[(PFOc)SO_3]^-$ at the M06-2X-D3/6-31G(d,p) level with an implicit solvent model PCM. Relative energies are listed in kcal/mol.

Table S47. Representations of lowest energy forms for $5CO_2[Hmim]^+[(PFOc)SO_3]^-$ at the M06-2X-D3/6-31G(d,p) level with an implicit solvent model PCM. Relative energies are listed in kcal/mol.

Table S48. Representations of lowest energy forms for $1CO_2[Omim]^+[(PFOc)SO_3]^-$ at the M06-2X-D3/6-31G(d,p) level with an implicit solvent model PCM. Relative energies are listed in kcal/mol.

Table S49. Representations of lowest energy forms for $2CO_2[Omim]^+[(PFOc)SO_3]^-$ at the M06-2X-D3/6-31G(d,p) level with an implicit solvent model PCM. Relative energies are listed in kcal/mol.

Table S50. Representations of lowest energy forms for $3CO_2[Omim]^+[(PFOc)SO_3]^-$ at the M06-2X-D3/6-31G(d,p) level with an implicit solvent model PCM. Relative energies are listed in kcal/mol.

Table S51. Representations of lowest energy forms for $4CO_2[Omim]^+[(PFOc)SO_3]^-$ at the M06-2X-D3/6-31G(d,p) level with an implicit solvent model PCM. Relative energies are listed in kcal/mol.

Table S52. Representations of lowest energy forms for $5CO_2[Omim]^+[(PFOc)SO_3]^-$at the M06-2X-D3/6-31G(d,p) level with an implicit solvent model PCM. Relative energies are listed in kcal/mol.

Table S53. Representations of lowest energy forms for $1CO_2[Hmim]^+[(PFBu)SO_3]^-$ at the M06-2X-D3/6-31G(d,p) level with an implicit solvent model PCM. Relative energies are listed in kcal/mol.

Table S54. Representations of lowest energy forms for $2CO_2[Hmim]^+[(PFBu)SO_3]^-$ at the M06-2X-D3/6-31G(d,p) level with an implicit solvent model PCM. Relative energies are listed in kcal/mol.

Table S55. Representations of lowest energy forms for $3CO_2[Hmim]^+[(PFBu)SO_3]^-$ at the M06-2X-D3/6-31G(d,p) level with an implicit solvent model PCM. Relative energies are listed in kcal/mol.

Table S56. Representations of lowest energy forms for $4CO_2[Hmim]^+[(PFBu)SO_3]^-$ at the M06-2X-D3/6-31G(d,p) level with an implicit solvent model PCM. Relative energies are listed in kcal/mol.

Table S57. Representations of lowest energy forms for $5CO_2[Hmim]^+[(PFBu)SO_3]^-$ at the M06-2X-D3/6-31G(d,p) level with an implicit solvent model PCM. Relative energies are listed in kcal/mol.

Table S58. Representations of lowest energy forms for $1CO_2[Omim]^+[(PFBu)SO_3]^-$at the M06-2X-D3/6-31G(d,p) level with an implicit solvent model PCM. Relative energies are listed in kcal/mol.

Table S59. Representations of lowest energy forms for $2CO_2[Omim]^+[(PFBu)SO_3]^-$at the M06-2X-D3/6-31G(d,p) level with an implicit solvent model PCM. Relative energies are listed in kcal/mol.

Table S60. Representations of lowest energy forms for $3CO_2[Omim]^+[(PFBu)SO_3]^-$ at the M06-2X-D3/6-31G(d,p) level with an implicit solvent model PCM. Relative energies are listed in kcal/mol.

Table S61. Representations of lowest energy forms for $4CO_2[Omim]^+[(PFBu)SO_3]^-$ at the M06-2X-D3/6-31G(d,p) level with an implicit solvent model PCM. Relative energies are listed in kcal/mol.

Table S62. Representations of lowest energy forms for $4CO_2[Omim]^+[(PFBu)SO_3]^-$ at the M06-2X-D3/6-31G(d,p) level with an implicit solvent model PCM. Relative energies are listed in kcal/mol.

Table S63. Stabilization order of the clusters by number of CO_2 molecules, where I represents ΔE and II represents ΔH

Table S64. Topological parameters, where BT = bond type, IN = interaction number, $\rho(r_{cp})$ and $\nabla^2\rho(r_{cp})$ in a.u..

FIGURES

Figure S1. Putative global minimum-energy structures for the $nCO_2[C_8H_4F_{13}mim]^+[BF_4]^-$, $nCO_2[C_8H_4F_{13}mim]^+[TFO]^-$, $nCO_2[Dmim]^+[BF_4]^-$, $nCO_2[Dmim]^+[TFO]^-$ molecular clusters. Atoms color scheme: carbon (grey), nitrogen (blue), sulphur (yellow), phosphorus (orange), fluorine (green), and hydrogen (white) at a level of theory M06-2X/cc-pVTZ (D3, SMD).

Figure S2. Putative global minimum-energy structures for the $nCO_2[Hmim]^+[FAP]^-$, $nCO_2[Dbim]^+[FAP]^-$, $nCO_2[Hmim]^+[Methide]^-$, $nCO_2[Dbim]^+[Methide]^-$ molecular clusters. Atoms color scheme: carbon (grey), nitrogen (blue), sulphur (yellow), phosphorus (orange), fluorine (green), and hydrogen (white) at a level of theory M06-2X/cc-pVTZ (D3, SMD).

Figure S3. Putative global minimum-energy structures for the $nCO_2[Hmim]^+[(PFOc)SO_3]^-$, $nCO_2[Omim]^+[(PFOc)SO_3]^-$, nCO_2 $[Hmim]^+[(PFBu)SO_3]^-$, $nCO_2[Omim]^+[(PFBu)SO_3]^-$molecular clusters. Atoms color scheme: carbon (grey), nitrogen (blue), sulphur (yellow), phosphorus (orange), fluorine (green), and hydrogen (white) at a level of theory M06-2X/cc-pVTZ (D3, SMD).

Figure S4. Difference between ΔH-ΔE of the clusters nCO_2IL; n = 1 a 5; IL = a: $[Dbim]^+[FAP]^-$, b: $[C_8H_4F_{13}mim]^+[TFO]^-$, c: $[Hmim]^+[FAP]^-$, d: $[C_8H_4F_{13}mim]^+[BF_4]^-$, e: $[Dbim]^+[Methide]^-$, f: $[Hmim]^+[Methide]^-$, g: $[Dmim]^+[BF_4]^-$, h: $[Omim]^+[(PFBu)SO_3]^-$, i: $[Dmim]^+[TFO]^-$, j: $[Hmim]^+[(PFOc)SO_3]^-$, k: $[Omim]^+[(PFOc)SO_3]^-$, l: $[Hmim]^+[(PFBu)SO_3]^-$. ΔE and ΔH in kcal/mol.

Figure S5. Molecular graphs and Bond critical points (BCP) for the $nCO_2[C_8H_4F_{13}mim]^+[BF4]^-$, $nCO_2[C_8H_4F_{13}mim]^+[TFO]^-$, $nCO_2[Dmim]^+[BF_4]^-$, $nCO_2[Dmim]^+[TFO]^-$ molecular clusters. Atoms color scheme: carbon (grey), nitrogen (blue), sulphur (yellow), phosphorus (orange), fluorine (green), and hydrogen (white). BCP are shown as green spheres. Bond paths are drawn as dashed lines paths at a level of theory M06-2X/cc-pVTZ (D3, SMD).

Figure S6. Molecular graphs and Bond critical points (BCP) for the $nCO_2[Hmim]^+[FAP]^-$, $nCO_2[Dbim]^+[FAP]^-$, $nCO_2[Hmim]^+[Methide]^-$, $nCO_2[Dbim]^+[Methide]^-$ molecular clusters. Atoms color scheme: carbon (grey), nitrogen (blue), sulphur (yellow), phosphorus (orange), fluorine (green), and hydrogen (white). BCP are shown as green spheres. Bond paths are drawn as dashed lines paths at a level of theory M06-2X/cc-pVTZ (D3, SMD).

Figure S7. Molecular graphs and Bond critical points (BCP) for the $nCO_2[Hmim]^+[(PFOc)SO_3]^-$, $nCO_2[Omim]^+[(PFOc)SO_3]^-$, nCO_2 $[Hmim]^+[(PFBu)SO_3]^-$, $nCO_2[Omim]^+[(PFBu)SO_3]^-$molecular clusters. Atoms color scheme: carbon (grey), nitrogen (blue), sulphur (yellow), phosphorus (orange), fluorine (green), and hydrogen (white). BCP are shown as green spheres. Bond paths are drawn as dashed lines paths at a level of theory M06-2X/cc-pVTZ (D3, SMD).

Figure S8. NCI plot isosurfaces (0.6 a.u) of the non-covalent interactions for $nCO_2[C_8H_4F_{13}mim]^+[BF4]^-$, $nCO_2[C_8H_4F_{13}mim]^+[TFO]^-$, $nCO_2[Dmim]^+[BF_4]^-$, $nCO_2[Dmim]^+[TFO]^-$ molecular clusters. Atoms color scheme: carbon (grey), nitrogen (blue), sulphur (yellow), phosphorus (orange), fluorine (green), and hydrogen (white). Green areas correspond to dispersive weak interactions.

Figure S9. NCI plot isosurfaces (0.6 a.u) of the non-covalent interactions for the $nCO_2[Hmim]^+[FAP]^-$, $nCO_2[Dbim]^+[FAP]^-$, $nCO_2[Hmim]^+[Methide]^-$, $nCO_2[Dbim]^+[Methide]^-$ molecular clusters. Atoms color scheme: carbon (grey), nitrogen (blue), sulphur (yellow), phosphorus (orange), fluorine (green areas), and hydrogen (white). Green areas correspond to dispersive weak interactions.

Figure S10 NCI plot isosurfaces (0.6 a.u) of the non-covalent interactions for the $nCO_2[Hmim]^+[(PFOc)SO_3]^-$, nCO_2 $[Hmim]^+[(PFBu)SO_3]^-$, $nCO_2[Omim]^+[(PFBu)SO_3]^-$, molecular clusters. Atoms color scheme: carbon (grey), nitrogen (blue), sulphur (yellow), phosphorus (orange), fluorine (green), and hydrogen (white). BCP are shown as green spheres. Bond paths are drawn as dashed lines paths at a level of theory M06-2X/cc-pVTZ (D3, SMD). Green areas correspond to dispersive weak interactions.

Figure S11. Average values of electron density $\rho(r_{cp})$ and number of interactions (IN) for the different types of interactions: 1 = Anion-Cation, 2 = Anion-CO_2, 3 = Cation-CO_2, 4 = CO_2-CO_2 for the clusters with $1CO_2$. (a) Plot for the clusters with IL a: $[Dbim]^+[FAP]^-$, b: $[C_8H_4F_{13}mim]^+[TFO]^-$, c: $[Hmim]^+[FAP]^-$, d: $[C_8H_4F_{13}mim]^+[BF_4]^-$, e: $[Dbim]^+[Methide]^-$, f: $[Hmim]^+[Methide]^-$. (b) Plot for the clusters with g: $[Dmim]^+[BF_4]^-$, h: $[Omim]^+[(PFBu)SO_3]^-$, i: $[Dmim]^+[TFO]^-$, j: $[Hmim]^+[(PFOc)SO_3]^-$, k: $[Omim]^+[(PFOc)SO_3]^-$, l: $[Hmim]^+[(PFBu)SO_3]^-$.

Figure S12. Average values of electron density $\rho(r_{cp})$ and number of interactions (IN) for the different types of interactions: 1 = Anion-Cation, 2 = Anion-CO_2, 3 = Cation-CO_2, 4 = CO_2-CO_2 for the clusters with 2CO_2. (a) Plot for the clusters with IL a: [Dbim]$^+$[FAP]$^-$, b: [$C_8H_4F_{13}$mim]$^+$[TFO]$^-$, c: [Hmim]$^+$[FAP]$^-$, d: [$C_8H_4F_{13}$mim]$^+$[BF_4]$^-$, e: [Dbim]$^+$[Methide]$^-$, f: [Hmim]$^+$[Methide]$^-$. (b) Plot for the clusters with IL g: [Dmim]$^+$[BF_4]$^-$, h: [Omim]$^+$[(PFBu)SO_3]$^-$, i: [Dmim]$^+$[TFO]$^-$, j: [Hmim]$^+$[(PFOc)SO_3]$^-$, k: [Omim]$^+$[(PFOc)SO_3]$^-$, l: [Hmim]$^+$[(PFBu)SO_3]$^-$.

Figure S13. Average values of electron density $\rho(r_{cp})$ and number of interactions (IN) for the different types of interactions: 1 = Anion-Cation, 2 = Anion-CO_2, 3 = Cation-CO_2, 4 = CO_2-CO_2 for the clusters with 3CO_2. (a) Plot for the clusters with IL a: [Dbim]$^+$[FAP]$^-$, b: [$C_8H_4F_{13}$mim]$^+$[TFO]$^-$, c: [Hmim]$^+$[FAP]$^-$, d: [$C_8H_4F_{13}$mim]$^+$[BF_4]$^-$, e: [Dbim]$^+$[Methide]$^-$, f: [Hmim]$^+$[Methide]$^-$. (b) Plot for the clusters with IL g: [Dmim]$^+$[BF_4]$^-$, h: [Omim]$^+$[(PFBu)SO_3]$^-$, i: [Dmim]$^+$[TFO]$^-$, j: [Hmim]$^+$[(PFOc)SO_3]$^-$, k: [Omim]$^+$[(PFOc)SO_3]$^-$, l: [Hmim]$^+$[(PFBu)SO_3]$^-$.

Figure S14. Average values of electron density $\rho(r_{cp})$ and number of interactions (IN) for the different types of interactions: 1 = Anion-Cation, 2 = Anion-CO_2, 3 = Cation-CO_2, 4 = CO_2-CO_2 for the clusters with 4CO_2. (a) Plot for the clusters with IL a: [Dbim]$^+$[FAP]$^-$, b: [$C_8H_4F_{13}$mim]$^+$[TFO]$^-$, c: [Hmim]$^+$[FAP]$^-$, d: [$C_8H_4F_{13}$mim]$^+$[BF_4]$^-$, e: [Dbim]$^+$[Methide]$^-$, f: [Hmim]$^+$[Methide]$^-$. (b) Plot for the clusters with IL g: [Dmim]$^+$[BF_4]$^-$, h: [Omim]$^+$[(PFBu)SO_3]$^-$, i: [Dmim]$^+$[TFO]$^-$, j: [Hmim]$^+$[(PFOc)SO_3]$^{-,}$ k: [Omim]$^+$[(PFOc)SO_3]$^-$, l: [Hmim]$^+$[(PFBu)SO_3]$^-$.

Figure S15. Average values of electron density ρ(rcp) and number of interactions (IN) for the different types of interactions: 1 = Anion-Cation, 2 = Anion-CO_2, 3 = Cation-CO_2, 4 = CO_2-CO_2 per interaction class for 5CO_2. a) [Dbim]$^+$[FAP]$^-$, b) [Dbim]$^+$[Methide]$^-$, c) [Hmim]$^+$[FAP]$^-$, d) [Hmim]$^+$[Methide]$^-$ at a level of theory M06-2X/cc-pVTZ (D3, SMD).

Figure S16. Average values of electron density ρ(rcp) and number of interactions (IN) for the different types of interactions: 1 = Anion-Cation, 2 = Anion-CO_2, 3 = Cation-CO_2, 4 = CO_2-CO_2 per interaction class for 5CO_2. a) [Hmim]$^+$[(PFOc)SO_3]$^-$, b) [Hmim]$^+$[(PFBu)SO_3]$^-$,, c) [Omim]$^+$[(PFOc)SO_3]$^-$, d) [Omim]$^+$[(PFBu)SO_3]$^-$ at a level of theory M06-2X/cc-pVTZ (D3, SMD).

Figure S17. Average values of electron density ρ(rcp) and number of interactions (IN) for the different types of interactions: 1 = Anion-Cation, 2 = Anion-CO_2, 3 = Cation-CO_2, 4 = CO_2-CO_2 per interaction class for 4CO_2. a) [$C_8H_4F_{13}$mim]$^+$[BF_4]$^-$, b) [$C_8H_4F_{13}$mim]$^+$ [TFO]$^-$, c) [Dmim]$^+$[BF_4]$^-$, d) [Dmim]$^+$[TFO]$^-$ at a level of theory M06-2X/cc-pVTZ (D3, SMD).

Figure S18. Average values of electron density ρ(rcp) and number of interactions (IN) for the different types of interactions: 1 = Anion-Cation, 2 = Anion-CO2, 3 = Cation-CO_2, 4 = CO_2-CO_2 per interaction class for 4CO_2. a) [Dbim]$^+$[FAP]$^-$, b) [Dbim]$^+$[Methide]$^-$, c) [Hmim]$^+$[FAP]$^-$, d) [Hmim]$^+$[Methide]$^-$ at a level of theory M06-2X/cc-pVTZ (D3, SMD).

Figure S19. Average values of electron density ρ(rcp) and number of interactions (IN) for the different types of interactions: 1 = Anion-Cation, 2 = Anion-CO_2, 3 = Cation-CO_2, 4 = CO_2-CO_2 per interaction class for 4CO_2. a) [Hmim]$^+$[(PFOc)SO_3]$^-$, b) [Hmim]$^+$[(PFBu)SO_3]$^-$, c) [Omim]$^+$[(PFOc)SO_3]$^-$, d) [Omim]$^+$[(PFBu)SO_3]$^-$ at a level of theory M06-2X/cc-pVTZ (D3, SMD).

Figure S20. Average values of electron density ρ(rcp) and number of interactions (IN) for the different types of interactions: 1 = Anion-Cation, 2 = Anion-CO_2, 3 = Cation-CO_2, 4 = CO_2-CO_2 per interaction class for 3CO_2. a) [$C_8H_4F_{13}$mim]$^+$[BF_4]$^-$, b) [$C_8H_4F_{13}$mim]$^+$ [TFO]$^-$, c) [Dmim]$^+$[BF_4]$^-$, d) [Dmim]$^+$[TFO]$^-$ at a level of theory M06-2X/cc-pVTZ (D3, SMD).

Figure S21. Average values of electron density ρ(rcp) and number of interactions (IN) for the different types of interactions: 1 = Anion-Cation, 2 = Anion-CO_2, 3 = Cation-CO_2, 4 = CO_2-CO_2 per interaction class for 3CO_2. a) [Dbim]$^+$[FAP]$^-$, b) [Dbim]$^+$[Methide]$^-$, c) [Hmim]$^+$[FAP]$^-$, d) [Hmim]$^+$[Methide]$^-$ at a level of theory M06-2X/cc-pVTZ (D3, SMD).

Figure S22. Average values of electron density ρ(rcp) and number of interactions (IN) for the different types of interactions: 1 = Anion-Cation, 2 = Anion-CO_2, 3 = Cation-CO_2, 4 = CO_2-CO_2 per interaction class for 3CO_2. a) [Hmim]$^+$[(PFOc)SO_3]$^-$, b) [Hmim]$^+$[(PFBu)SO_3]$^-$, c) [Omim]$^+$[(PFOc)SO_3]$^-$, d) [Omim]$^+$[(PFBu)SO_3]$^-$ at a level of theory M06-2X/cc-pVTZ (D3, SMD).

Figure S23 Average values of electron density ρ(rcp) and number of interactions (IN) for the different types of interactions: 1 = Anion-Cation, 2 = Anion-CO_2, 3 = Cation-CO_2, 4 = CO_2-CO_2 per interaction class for 2CO_2. a) [$C_8H_4F_{13}$mim]$^+$[BF_4]$^-$, b) [$C_8H_4F_{13}$mim]$^+$ [TFO]$^-$, c) [Dmim]$^+$[BF_4]$^-$, d) [Dmim]$^+$[TFO]$^-$ at a level of theory M06-2X/cc-pVTZ (D3, SMD).

Figure S24. Average values of electron density ρ(rcp) and number of interactions (IN) for the different types of interactions: 1 = Anion-Cation, 2 = Anion-CO_2, 3 = Cation-CO_2, 4 = CO_2-CO_2 per interaction class for 2CO_2. a) [Dbim]$^+$[FAP]$^-$, b) [Dbim]$^+$[Methide]$^-$, c) [Hmim]$^+$[FAP]$^-$, d) [Hmim]$^+$[Methide]$^-$ at a level of theory M06-2X/cc-pVTZ (D3, SMD).

Figure S25. Average values of electron density ρ(rcp) and number of interactions (IN) for the different types of interactions: 1 = Anion-Cation, 2 = Anion-CO_2, 3 = Cation-CO_2, 4 = CO_2-CO_2 per interaction class for 2CO_2. a) [Hmim]$^+$[(PFOc)SO_3]$^-$, b) [Hmim]$^+$[(PFBu)SO_3]$^-$, c) [Omim]$^+$[(PFOc)SO_3]$^-$, d) [Omim]$^+$[(PFBu)SO_3]$^-$ at a level of theory M06-2X/cc-pVTZ (D3, SMD).

Figure S26. Average values of electron density ρ(rcp) and number of interactions (IN) for the different types of interactions: 1 = Anion-Cation, 2 = Anion-CO_2, 3 = Cation-CO_2, 4 = CO_2-CO_2 per interaction class for 1CO_2. a) [$C_8H_4F_{13}$mim]$^+$[BF_4]$^-$, b) [$C_8H_4F_{13}$mim]$^+$ [TFO]$^-$, c) [Dmim]$^+$[BF_4]$^-$, d) [Dmim]$^+$[TFO]$^-$ at a level of theory M06-2X/cc-pVTZ (D3, SMD).

Figure S27. Average values of electron density ρ(rcp) and number of interactions (IN) for the different types of interactions: 1 = Anion-Cation, 2 = Anion-CO_2, 3 = Cation-CO_2, 4 = CO_2-CO_2 per interaction class for 1CO_2. a) [Dbim]$^+$[FAP]$^-$, b) [Dbim]$^+$[Methide]$^-$, c) [Hmim]$^+$[FAP]$^-$, d) [Hmim]$^+$[Methide]$^-$ at a level of theory M06-2X/cc-pVTZ (D3, SMD).

Figure S28. Average values of electron density ρ(rcp) and number of interactions (IN) for the different types of interactions: 1 = Anion-Cation, 2 = Anion-CO_2, 3 = Cation-CO_2, 4 = CO_2-CO_2 per interaction class for 1CO_2. a) [Hmim]$^+$[(PFOc)SO_3]$^-$, b) [Hmim]$^+$[(PFBu)SO_3]$^-$, c) [Omim]$^+$[(PFOc)SO_3]$^-$, d) [Omim]$^+$[(PFBu)SO_3]$^-$ at a level of theory M06-2X/cc-pVTZ (D3, SMD).

Figure S29. Interaction Energy values -IE(rcp) for the different types of interactions: 1 = Anion-Cation, 2 = Anion-CO_2, 3 = Cation-CO_2, 4 = CO_2-CO_2 for the clusters with 1CO_2. (a) Values for the clusters formed with ILs a to f. a: [Dbim]$^+$[FAP]$^-$, b: [$C_8H_4F_{13}$mim]$^+$[TFO]$^-$, c: [Hmim]$^+$[FAP]$^-$, d: [$C_8H_4F_{13}$mim]$^+$[BF_4]$^-$, e: [Dbim]$^+$[Methide]$^-$, f: [Hmim]$^+$[Methide]$^-$. (b) Values for clusters formed with ILs g to l. g: [Dmim]$^+$[BF_4]$^-$, h: [Omim]$^+$[(PFBu)SO_3]$^-$, i: [Dmim]$^+$[TFO]$^-$, j: [Hmim]$^+$[(PFOc)SO_3]$^-$, k: [Omim]$^+$[(PFOc)SO_3]$^-$, l: [Hmim]$^+$[(PFBu)SO_3]$^-$.

Figure S30. Interaction Energy values -IE(rcp) for the different types of interactions: 1 = Anion-Cation, 2 = Anion-CO_2, 3 = Cation-CO_2, 4 = CO_2-CO_2 for the clusters with 2CO2. (a) Values for the clusters formed with ILs a to f. a: [Dbim]$^+$[FAP]$^-$, b: [$C_8H_4F_{13}$mim]$^+$[TFO]$^-$, c: [Hmim]$^+$[FAP]$^-$, d: [$C_8H_4F_{13}$mim]$^+$[BF_4]$^-$, e: [Dbim]$^+$[Methide]$^-$, f: [Hmim]$^+$[Methide]$^-$. (b) Values for clusters formed with ILs g to l. g: [Dmim]$^+$[BF_4]$^-$, h: [Omim]$^+$[(PFBu)SO_3]$^-$, i: [Dmim]$^+$[TFO]$^-$, j: [Hmim]$^+$[(PFOc)SO_3]$^-$, k: [Omim]$^+$[(PFOc)SO_3]$^-$, l: [Hmim]$^+$[(PFBu)SO_3]$^-$.

Figure S31. Interaction Energy values -IE(rcp) for the different types of interactions: 1 = Anion-Cation, 2 = Anion-CO_2, 3 = Cation-CO_2, 4 = CO_2-CO_2 for the clusters with 3CO_2. (a) Values for the clusters formed with ILs a to f. a: [Dbim]$^+$[FAP]$^-$, b: [$C_8H_4F_{13}$mim]$^+$[TFO]$^-$, c: [Hmim]$^+$[FAP]$^-$, d: [$C_8H_4F_{13}$mim]$^+$[BF_4]$^-$, e: [Dbim]$^+$[Methide]$^-$, f: [Hmim]$^+$[Methide]$^-$. (b) Values for clusters formed with ILs g to l. g: [Dmim]$^+$[BF_4]$^-$, h: [Omim]$^+$[(PFBu)SO_3]$^-$, i: [Dmim]$^+$[TFO]$^-$, j: [Hmim]$^+$[(PFOc)SO_3]$^-$, k: [Omim]$^+$[(PFOc)SO_3]$^-$, l: [Hmim]$^+$[(PFBu)SO_3]$^-$.

Figure S32. Interaction Energy values -IE(rcp) for the different types of interactions: 1 = Anion-Cation, 2 = Anion-CO_2, 3 = Cation-CO_2, 4 = CO_2-CO_2 for the clusters with 4CO_2. (a) Values for the clusters formed with ILs a to f. a: [Dbim]$^+$[FAP]$^-$, b: [$C_8H_4F_{13}$mim]$^+$[TFO]$^-$, c: [Hmim]$^+$[FAP]$^-$, d: [$C_8H_4F_{13}$mim]$^+$[BF_4]$^-$, e: [Dbim]$^+$[Methide]$^-$, f: [Hmim]$^+$[Methide]$^-$. (b) Values for clusters formed with ILs g to l. g: [Dmim]$^+$[BF_4]$^-$, h: [Omim]$^+$[(PFBu)SO_3]$^-$, i: [Dmim]$^+$[TFO]$^-$, j: [Hmim]$^+$[(PFOc)SO_3]$^-$, k: [Omim]$^+$[(PFOc)SO_3]$^-$, l: [Hmim]$^+$[(PFBu)SO_3]$^-$.

Figure S33. Average values of Interaction Energy values -IE(rcp) for the different types of interactions: 1 = Anion-Cation, 2 = Anion-CO_2, 3 = Cation-CO_2, 4 = CO_2-CO_2 per interaction class for 5CO_2. a) [$C_8H_4F_{13}$mim]$^+$[BF_4]$^-$, b) [$C_8H_4F_{13}$mim]$^+$[TFO]$^-$, c) [Dmim]$^+$[BF_4]$^-$, d) [Dmim]$^+$[TFO]$^-$ at a level of theory M06-2X/cc-pVTZ (D3, SMD).

Figure S34. Average values of Interaction Energy values -IE(rcp) for the different types of interactions: 1 = Anion-Cation, 2 = Anion-CO_2, 3 = Cation-CO_2, 4 = CO_2-CO_2 per interaction class for 5CO_2. a) [Dbim]$^+$[FAP]$^-$, b) [Dbim]$^+$[Methide]$^-$, c) [Hmim]$^+$[FAP]$^-$, d) [Hmim]$^+$[Methide]$^-$ at a level of theory M06-2X/cc-pVTZ (D3, SMD).

Figure S35. Average values of Interaction Energy values -IE(rcp) for the different types of interactions: 1 = Anion-Cation, 2 = Anion-CO_2, 3 = Cation-CO_2, 4 = CO_2-CO_2 per interaction class for 5CO_2. a) [Hmim]$^+$[(PFOc)SO_3]$^-$, b) [Hmim]$^+$[(PFBu)SO_3]$^-$, c) [Omim]$^+$[(PFOc)SO_3]$^-$, d) [Omim]$^+$[(PFBu)SO_3]$^-$ at a level of theory M06-2X/cc-pVTZ (D3, SMD).

Figure S36. Average values of Interaction Energy values -IE(rcp) for the different types of interactions: 1 = Anion-Cation, 2 = Anion-CO_2, 3 = Cation-CO_2, 4 = CO_2-CO_2 per interaction class for 4CO_2. a) [$C_8H_4F_{13}$mim]$^+$[BF_4]$^-$, b) [$C_8H_4F_{13}$mim]$^+$[TFO]$^-$, c) [Dmim]$^+$[BF_4]$^-$, d) [Dmim]$^+$[TFO]$^-$ at a level of theory M06-2X/cc-pVTZ (D3, SMD).

Figure S37. Average values of Interaction Energy values -IE(rcp) for the different types of interactions: 1 = Anion-Cation, 2 = Anion-CO_2, 3 = Cation-CO_2, 4 = CO_2-CO_2 per interaction class for 4CO_2. a) $[Dbim]^+[FAP]^-$, b) $[Dbim]^+[Methide]^-$, c) $[Hmim]^+[FAP]^-$, d) $[Hmim]^+[Methide]^-$ at a level of theory M06-2X/cc-pVTZ (D3, SMD).

Figure S38. Average values of Interaction Energy values -IE(rcp) for the different types of interactions: 1 = Anion-Cation, 2 = Anion-CO_2, 3 = Cation-CO_2, 4 = CO_2-CO_2 per interaction class for 4CO_2. a) $[Hmim]^+[(PFOc)SO_3]^-$, b) $[Hmim]^+[(PFBu)SO_3]^-$, c) $[Omim]^+[(PFOc)SO_3]^-$, d) $[Omim]^+[(PFBu)SO_3]^-$ at a level of theory M06-2X/cc-pVTZ (D3, SMD).

Figure S39. Average values of Interaction Energy values -IE(rcp) for the different types of interactions: 1 = Anion-Cation, 2 = Anion-CO_2, 3 = Cation-CO_2, 4 = CO_2-CO_2 per interaction class for 3CO_2. a) $[C_8H_4F_{13}mim]^+[BF_4]^-$, b) $[C_8H_4F_{13}mim]^+[TFO]^-$, c) $[Dmim]^+[BF_4]^-$, d) $[Dmim]^+[TFO]^-$ at a level of theory M06-2X/cc-pVTZ (D3, SMD).

Figure S40. Average values of Interaction Energy values -IE(rcp) for the different types of interactions: 1 = Anion-Cation, 2 = Anion-CO_2, 3 = Cation-CO_2, 4 = CO_2-CO_2 per interaction class for 3CO_2. a) $[Dbim]^+[FAP]^-$, b) $[Dbim]^+[Methide]^-$, c) $[Hmim]^+[FAP]^-$, d) $[Hmim]^+[Methide]^-$ at a level of theory M06-2X/cc-pVTZ (D3, SMD).

Figure S41. Average values of Interaction Energy values -IE(rcp) for the different types of interactions: 1 = Anion-Cation, 2 = Anion-CO_2, 3 = Cation-CO_2, 4 = CO_2-CO_2 per interaction class for 3CO_2. a) $[Hmim]^+[(PFOc)SO_3]^-$, b) $[Hmim]^+[(PFBu)SO_3]^-$, c) $[Omim]^+[(PFOc)SO_3]^-$, d) $[Omim]^+[(PFBu)SO_3]^-$ at a level of theory M06-2X/cc-pVTZ (D3, SMD).

Figure S42. Average values of Interaction Energy values -IE(rcp) for the different types of interactions: 1 = Anion-Cation, 2 = Anion-CO_2, 3 = Cation-CO_2, 4 = CO_2-CO_2 per interaction class for 2CO_2. a) $[C_8H_4F_{13}mim]^+[BF_4]^-$, b) $[C_8H_4F_{13}mim]^+[TFO]^-$, c) $[Dmim]^+[BF_4]^-$, d) $[Dmim]^+[TFO]^-$ at a level of theory M06-2X/cc-pVTZ (D3, SMD).

Figure S43. Average values of Interaction Energy values -IE(rcp) for the different types of interactions: 1 = Anion-Cation, 2 = Anion-CO_2, 3 = Cation-CO_2, 4 = CO_2-CO_2 per interaction class for 2CO_2. a) $[Dbim]^+[FAP]^-$, b) $[Dbim]^+[Methide]^-$, c) $[Hmim]^+[FAP]^-$, d) $[Hmim]^+[Methide]^-$ at a level of theory M06-2X/cc-pVTZ (D3, SMD).

Figure S44. Average values of Interaction Energy values -IE(rcp) for the different types of interactions: 1 = Anion-Cation, 2 = Anion-CO_2, 3 = Cation-CO_2, 4 = CO_2-CO_2 per interaction class for 2CO_2. a) $[Hmim]^+[(PFOc)SO_3]^-$, b) $[Hmim]^+[(PFBu)SO_3]^-$, c) $[Omim]^+[(PFOc)SO_3]^-$, d) $[Omim]^+[(PFBu)SO_3]^-$ at a level of theory M06-2X/cc-pVTZ (D3, SMD).

Figure S45. Average values of Interaction Energy values -IE(rcp) for the different types of interactions: 1 = Anion-Cation, 2 = Anion-CO_2, 3 = Cation-CO_2, 4 = CO_2-CO_2 per interaction class for 1CO_2. a) $[C_8H_4F_{13}mim]^+[BF_4]^-$, b) $[C_8H_4F_{13}mim]^+[TFO]^-$, c) $[Dmim]^+[BF_4]^-$, d) $[Dmim]^+[TFO]^-$ at a level of theory M06-2X/cc-pVTZ (D3, SMD).

Figure S46. Average values of Interaction Energy values -IE(rcp) for the different types of interactions: 1 = Anion-Cation, 2 = Anion-CO_2, 3 = Cation-CO_2, 4 = CO_2-CO_2 per interaction class for 1CO_2. a) $[Dbim]^+[FAP]^-$, b) $[Dbim]^+[Methide]^-$, c) $[Hmim]^+[FAP]^-$, d) $[Hmim]^+[Methide]^-$ at a level of theory M06-2X/cc-pVTZ (D3, SMD).

Figure S47. Average values of Interaction Energy values -IE(rcp) for the different types of interactions: 1 = Anion-Cation, 2 = Anion-CO_2, 3 = Cation-CO_2, 4 = CO_2-CO_2 per interaction class for 1CO_2. a) $[Hmim]^+[(PFOc)SO_3]^-$, b) $[Hmim]^+[(PFBu)SO_3]^-$, c) $[Omim]^+[(PFOc)SO_3]^-$, d) $[Omim]^+[(PFBu)SO_3]^-$ at a level of theory M06-2X/cc-pVTZ (D3, SMD).

Figure S48. Electrostatic potential maps for $nCO_2[C_8H_4F_{13}mim]^+[BF4]^-$, $nCO_2[C_8H_4F_{13}mim]^+[TFO]^-$, $nCO_2[Dmim]^+[BF_4]^-$, $nCO_2[Dmim]^+[TFO]^-$ molecular clusters. All isosurfaces were generated at 0.01 a.u.

Figure S49. Electrostatic potential maps for $nCO_2[Dbim]^+[FAP]^-$, $nCO_2[Hmim]^+[FAP]^-$, $nCO_2[Dbim]^+[Methide]^-$, $nCO_2[Hmim]^+[Methide]^-$ molecular clusters with n = 1 a 5. All isosurfaces were generated at 0.01 a.u.

Figure S50. Electrostatic potential maps for $nCO_2[Hmim]^+[(PFOc)SO_3]^-$, nCO_2 $[Hmim]^+[(PFBu)SO_3]^-$, $nCO_2[Omim]^+[(PFBu)SO_3]^-$ molecular clusters with n = 1 a 5. All isosurfaces were generated at 0.01 a.u.

TABLES

Table S1. Experimental CO_2 Uptake Capacities of Representative Ionic Liquids

Experimental IL System / Functional Group	CO_2 Capacity	Experimental Method	Key Findings	Related IL in this study	Reference
ILs on mesoporous silica (Lys, Gly)	up to 0.61 mmol/g	TGA + desorption cycles	Fast kinetics, moderate chemisorption, reversible CO_2 capture	[emim][Lys], [emim][Gly]	Hiremath et al., 2016
Phosphonium ILs with H-bonding anions (Ac-PhO)	up to 1.2 mol/mol IL	Gravimetry volumetric + NMR, IR	Viscosity controlled by intramolecular H-bonding	Functionalized anion analogs	Luo et al., 2016
DBU-imidazole ILs	~1 mol/mol IL	Gravimetric analysis	Capture enhanced by anion substituents and temperature	DBU–Im-based ILs	Zhu et al., 2017
Fluorinated imidazolium ILs, phosphonium-based amino acid ILs	up to 0.90 mol/mol IL	High-pressure solubility (PVT), Gravimetric analysis	Free volume and fluorine enhance CO_2 affinity. Near 1:1 stoichiometry in anion-tethered amino acid ILs ([P_{66614}][Pro], [P_{66614}][Met])	[emim][Tf_2N], [bmim][FAP]	Zeng et al., 2017*
Blends: [emim][Ac] / [emim][TFA]	0.124 mol CO_2/mol IL	Gravimetric analysis	CO_2 solubility confirmed for [emim][Ac]/[TFA] blend; capacity lower than amine-functionalized TSILs	[emim][Ac] + [emim][TFA]	Zeng et al., 2017*
Task-specific ILs with amine groups (TSILs)	Varies (technique-dependent)	NMR and XRD	TSILs were capable of both chemical and physical Absorption. CO_2 Chemisorption through amino groups, higher affinity and selectivity	[C2MIM][Ac]	Sarmad et al., 2017*
Imidazolium ILs with amino acid anions (Lys, Ala)	up to 4.72 mol/mol IL	Gas bubbling, NDIR gas analyzer + NMR, FTIR	Dual-amino ILs outperform MEA in cyclic CO_2 capture	[emim][Lys], [emim][Ala]	Kang et al., 2020
Imidazolium ILs ([emim][MS], [emim][DCA]) in membrane systems	CO_2 removal efficiency >66%	Membrane contactors (absorption/desorption cycle)	High and sustained capture efficiency in practical separation systems	[emim][MS], [emim][DCA]	Sohaib et al., 2020

Imidazolium-based ILs with fluorinated or bulky anions	up to 0.755 mol CO_2/mol IL	Gravimetric and high-pressure solubility analysis	Fluorinated anions enhance CO_2 solubility; SCO_2 increases with number of fluorine atoms and anion size	[bmim][BF_4], [bmim][PF_6], [hmim][Methide], [hmim][FEP], [bmim][Methide], [eFAP], [bFAP]	Elmobarak et al., 2023*

Note: The experimental studies cited here validate the IL families and functional groups evaluated in our computational models, highlighting the complementarity of theoretical and empirical approaches. *These papers are reviews.

References

Elmobarak W. F., Almomani F., Tawalbeh M., AL-Othman A., Martis R., & Rasool K. (2023). Current status of CO_2 capture with ionic liquids: Development and progress. Fuel, 344, 128102.

Hiremath, V., Jadhav A.H., Lee H., Kwon S., & Seo J. G., (2016). Highly reversible CO_2 capture using amino acid functionalized ionic liquids immobilized on mesoporous silica. Chemical Engineering Journal, 287, 602–617.

Kang S., Kim, Chung Y. G., Kang J. H., & Song H. (2020). CO_2 absorption characteristics of amino group functionalized imidazolium-based amino acid ionic liquids. Journal of Molecular Liquids, 297, 111825.

Luo X. Y., Fan X., Shi G. L., Li H. R., & Wang C. M. (2016). Decreasing the viscosity in CO_2 capture by amino-functionalized ionic liquids through the formation of intramolecular hydrogen bond. The Journal of Physical Chemistry B, 120 (10), 2807–2813.

Sarmad S., Xie Y., Mikkola J. P., & Ji X. (2017). Carbon dioxide capture with ionic liquids and deep eutectic solvents: A new generation of sorbents. ChemSusChem, 10 (2), 324–352.

Sohaib Q., Vadillo J. M., Gómez-Coma L., Albo J., Druon-Bocquet S., Irabien A., Sanchez-Marcano J. (2020). CO_2 capture with room temperature ionic liquids; coupled absorption/desorption and single module absorption in membrane contactor. Chemical Engineering Science, 223, 115719.

Zeng, S., Zhang, X., Bai, L., Zhang, X., Wang, H., Wang, J., ... & Zhang, S. (2017). Ionic-liquid-based CO_2 capture systems: Structure, interaction and process. Chemical Reviews, 117 (14), 9625–9673.

Zhu X., Song M., & Xu Y. (2017). DBU-based protic ionic liquids for CO_2 capture. ACS Sustainable Chemistry & Engineering, 5(9), 8192–8198.

Table S2. Number of structures obtained in the PES analysis by optimization with the PM7 semi-empirical method and reoptimization with the M06-2X-D3/6-31G(d,p) approach.

N° CO_2	PM7	DFT	PM7	DFT	PM7	DFT	PM7	DFT	PM7	DFT	PM7	DFT
	$[C_8H_4F_{13}mim]^+$ $[BF_4]^-$		$[Dmim]^+$ $[TFO]^-$		$[C_8H_4F_{13}mim]^+$ $[TFO]^-$		$[Dmim]^+$ $[BF_4]^-$		$[Dbim]^+$ $[FAP]^-$		$[Dbim]^+$ $[Methide]^-$	
1	24	24	24	24	24	24	24	24	34	34	34	32
2	24	24	24	24	24	24	24	24	34	34	34	33
3	24	24	24	24	24	24	24	24	34	34	34	34
4	24	24	24	24	24	24	24	24	34	34	34	34
5	24	24	24	24	24	24	24	24	34	34	34	34
	$[Hmim]^+$ $[FAP]^-$		$[Hmim]^+$ $[Methide]^-$		$[Hmim]^+$ $[(PFOc)SO_3]^-$		$[Hmim]^+$ $[(PFBu)SO_3]^-$		$[Omim]^+$ $[(PFOc)SO_3]^-$		$[Omim]^+$ $[(PFBu)SO_3]^-$	
1	34	34	34	31	34	34	34	30	34	33	34	31
2	34	34	34	32	34	32	34	16	34	34	34	34
3	34	33	34	34	34	32	34	33	34	33	34	33
4	34	34	34	34	34	34	34	33	34	16	34	33
5	34	33	34	34	34	29	34	30	34	32	34	34

Table S3. Representations of lowest energy forms for $1CO_2[C_8H_4F_{13}mim]^+[BF_4]^-$ at the M06-2X-D3/6-31G(d,p) level with an implicit solvent model PCM. Relative energies are listed in kcal/mol.

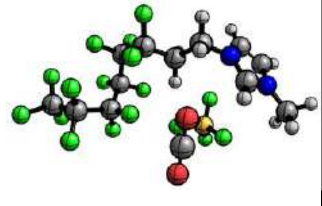	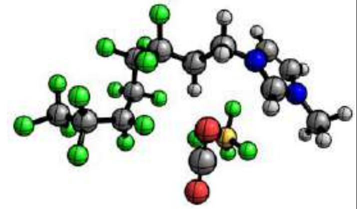	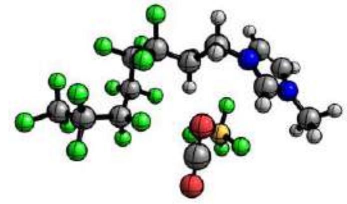	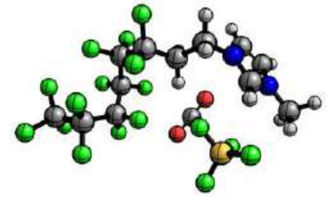	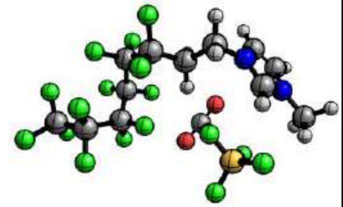
0.00	0.003	0.006	0.064	0.066

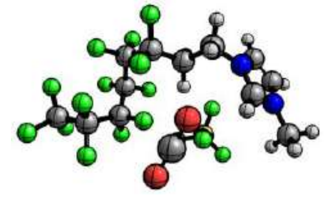	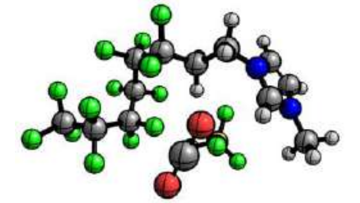	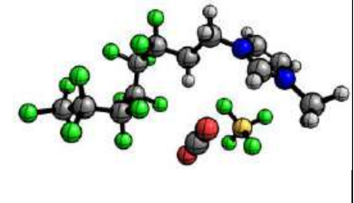	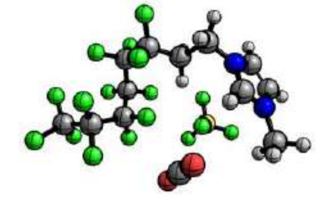	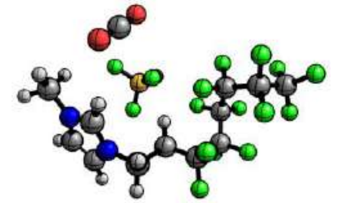
1.21	1.22	1.23	2.12	2.14
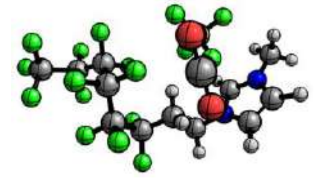	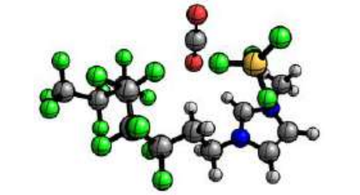	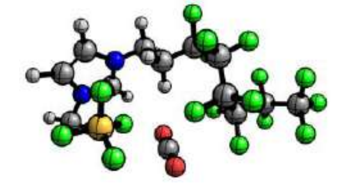	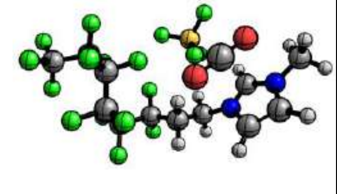	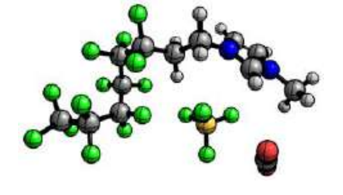
2.32	2.33	2.39	2.41	2.42
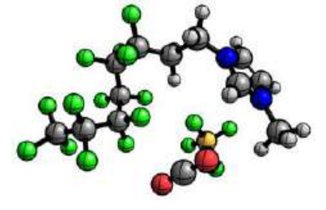	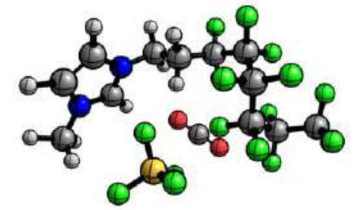	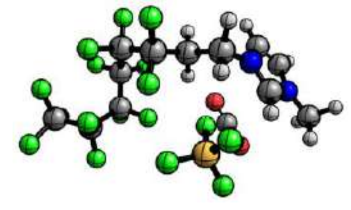	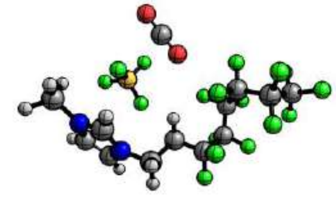	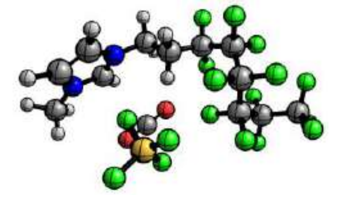
2.45	2.59	2.63	2.68	2.92
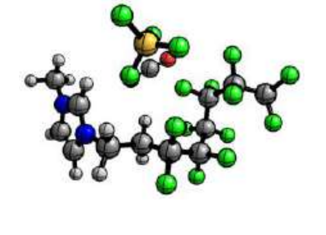	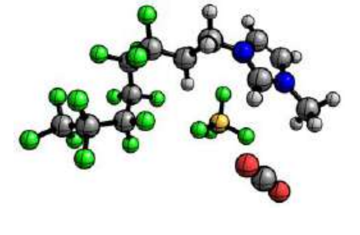	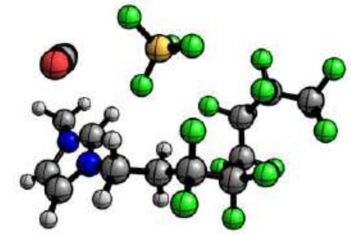	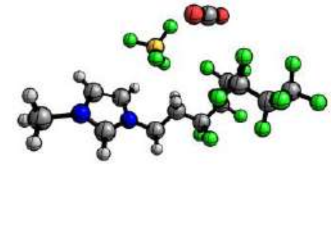	
3.49	3.98	4.56	5.70	

Table S4. Representations of lowest energy forms for $2CO_2[C_8H_4F_{13}mim]^+[BF_4]^-$ at the M06-2X-D3/6-31G(d,p) level with an implicit solvent model PCM. Relative energies are listed in kcal/mol.

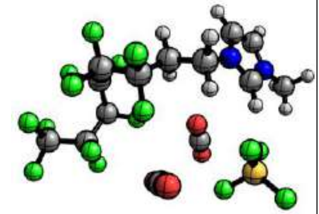	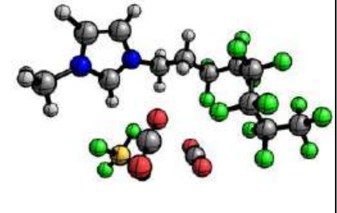	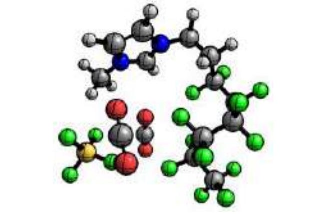	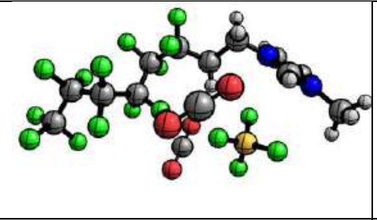	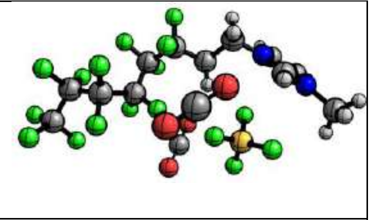
0.00	0.002	0.21	0.37	0.42
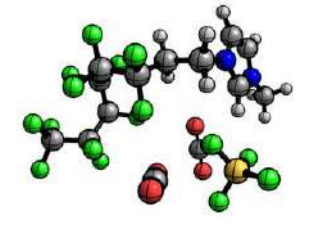	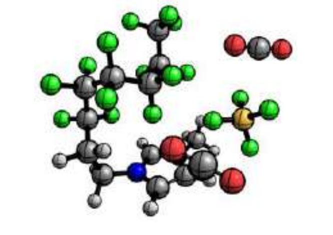	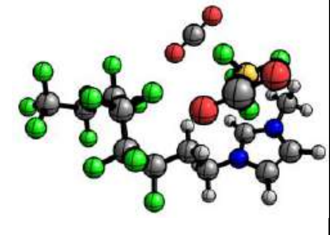	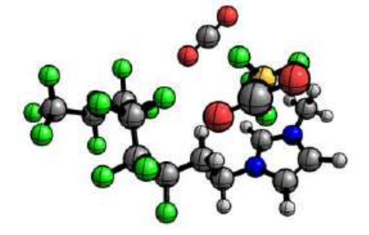	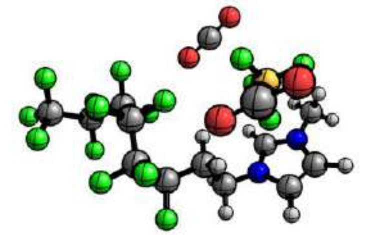
0.44	0.89	1.18	1.190	1.191
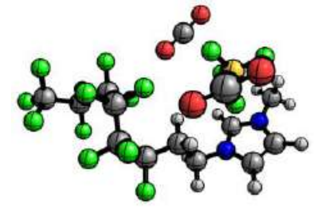	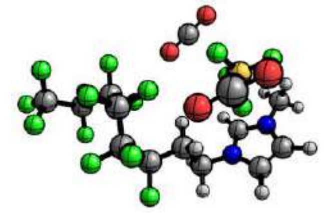	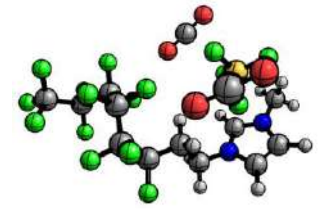	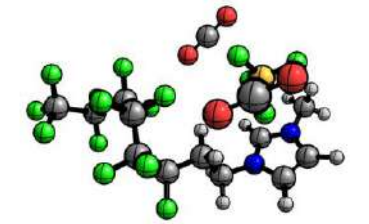	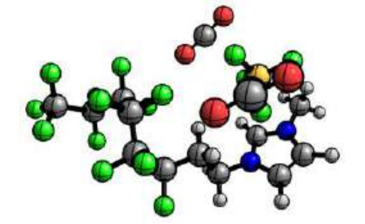
1.192	1.193	1.193	1.194	1.196
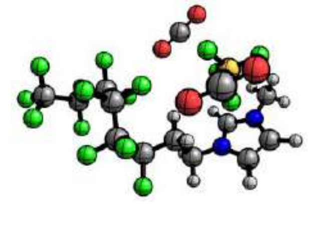	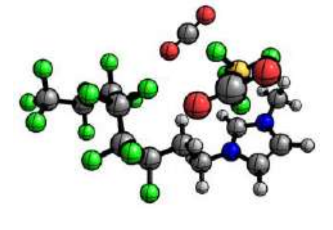	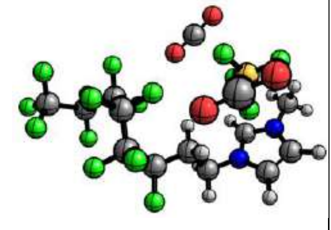	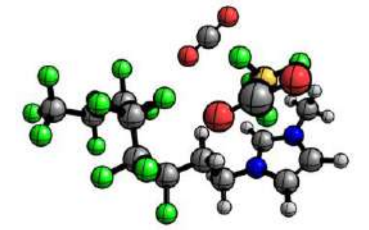	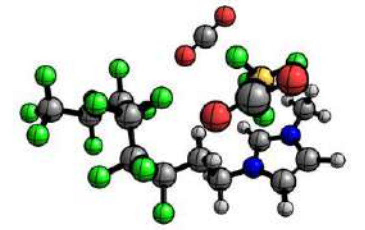
1.197	1.198	1.20	1.203	1.206

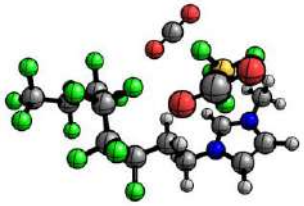	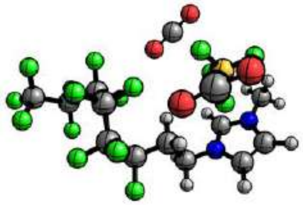	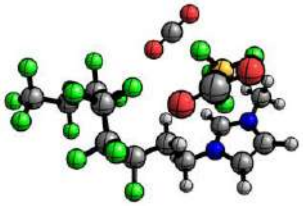	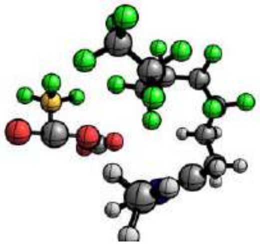	
1.207	1.207	1.208	2.090	

Table S5. Representations of lowest energy forms for $3CO_2[C_8H_4F_{13}mim]^+[BF_4]^-$ at the M06-2X-D3/6-31G(d,p) level with an implicit solvent model PCM. Relative energies are listed in kcal/mol.

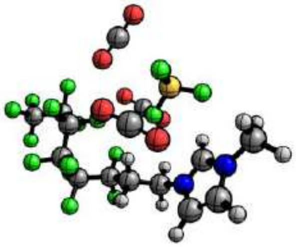	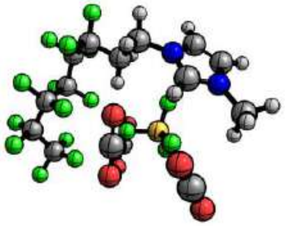	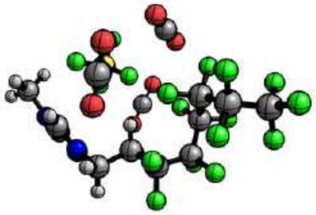	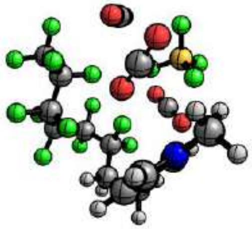	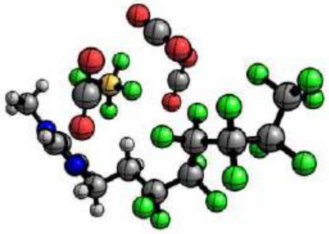
0.00	0.69	1.11	2.12	2.51
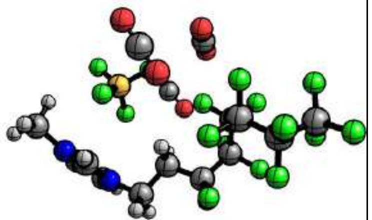	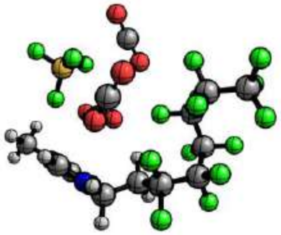	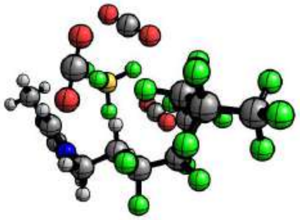	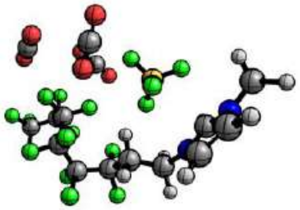	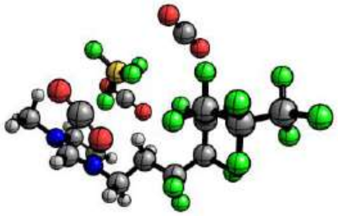
3.55	3.62	3.89	4.03	4.57

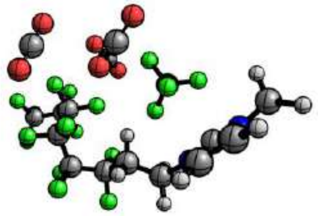	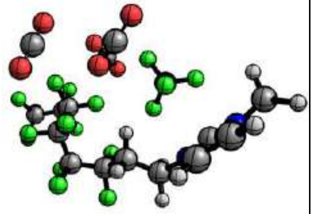	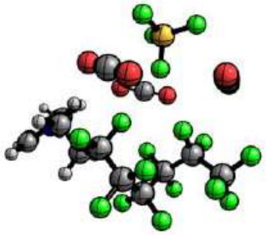	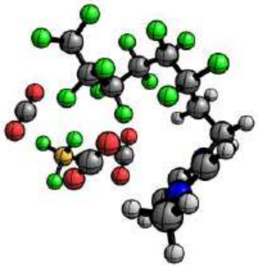	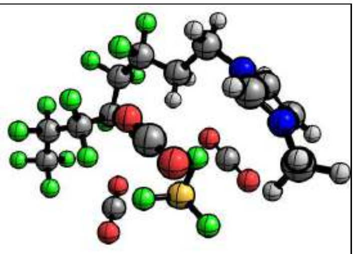
4.82	4.83	5.26	5.38	5.75
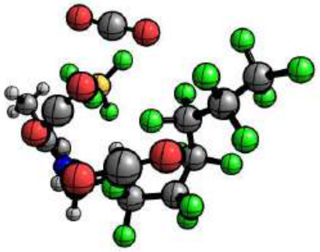	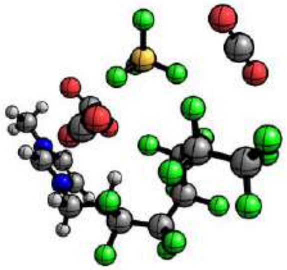	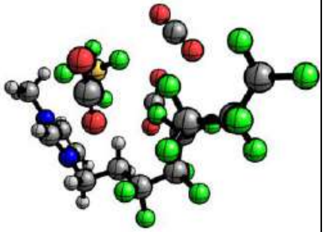	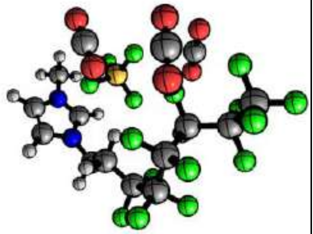	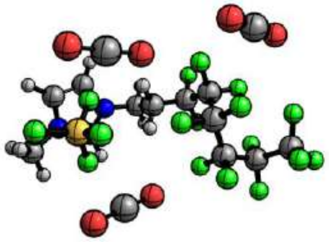
5.76	6.11	6.51	7.73	8.04
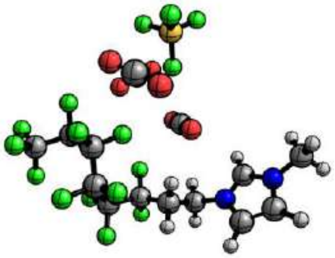	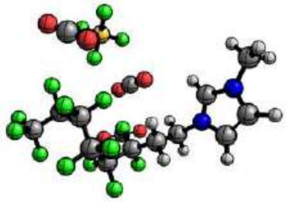	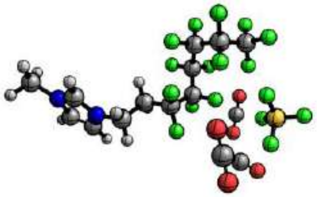	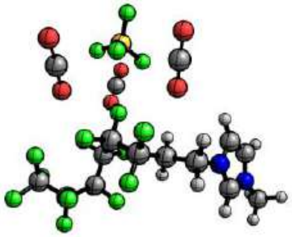	
10.09	10.16	11.24	13.18	

Table S6. Representations of lowest energy forms for $4CO_2[C_8H_4F_{13}mim]^+[BF_4]^-$ at the M06-2X-D3/6-31G(d,p) level with an implicit solvent model PCM. Relative energies are listed in kcal/mol.

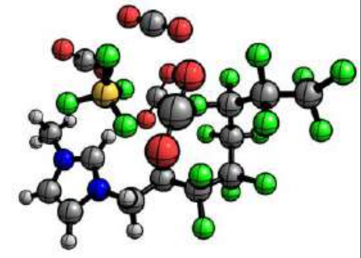	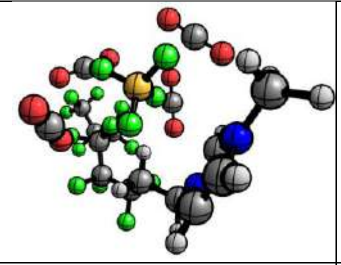	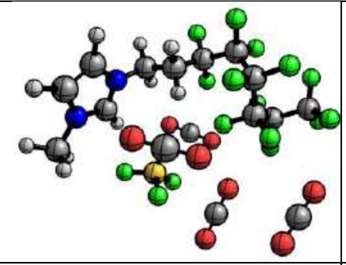	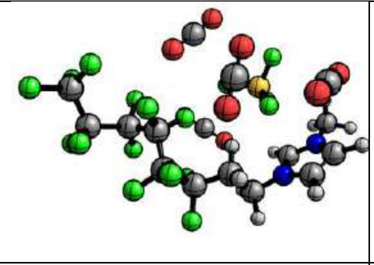	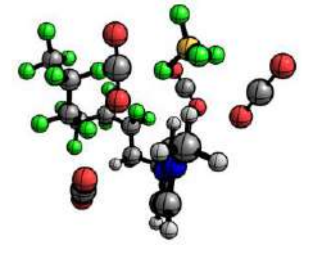
0.00	0.78	1.11	1.18	2.04
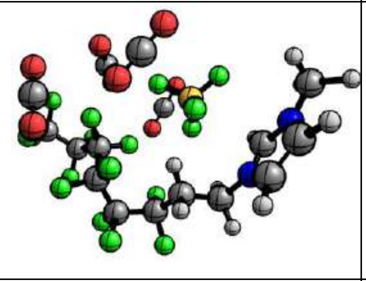	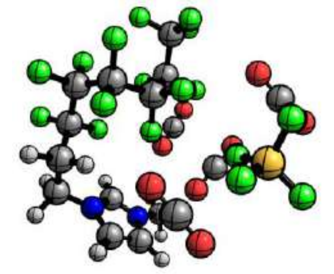	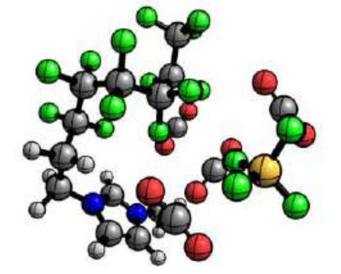	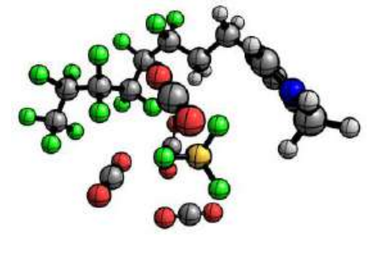	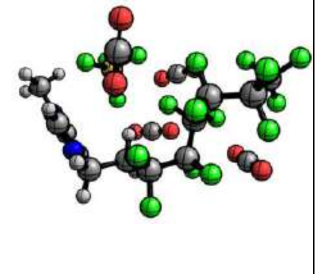
3.74	5.02	5.07	5.45	6.00
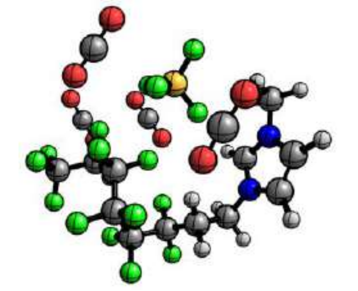	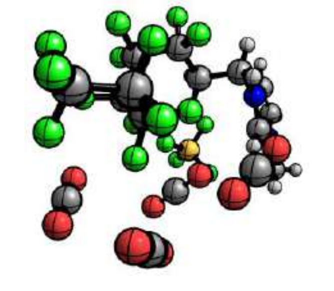	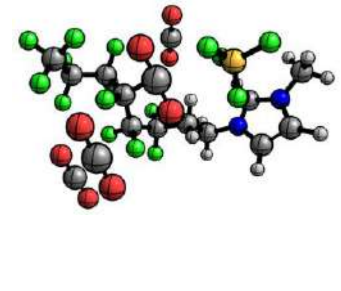	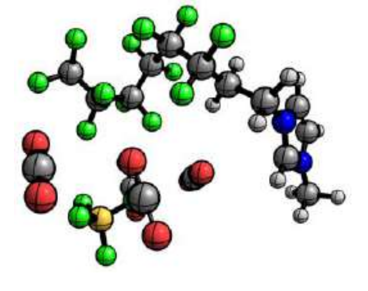	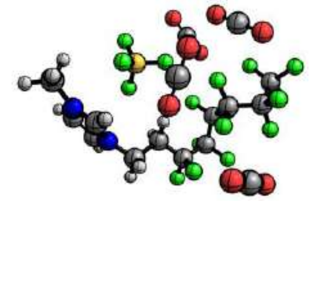
6.35	7.20	7.44	8.59	9.23

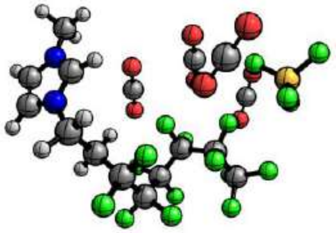	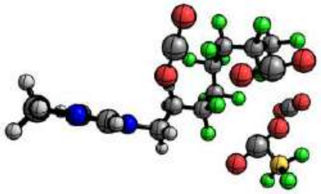	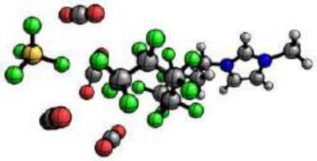	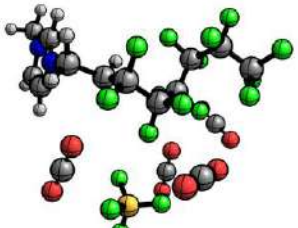	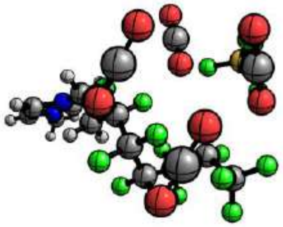
9.95	10.30	10.33	11.72	11.97
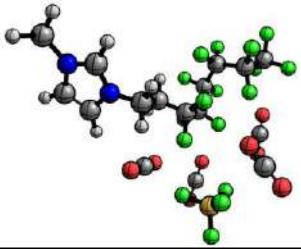	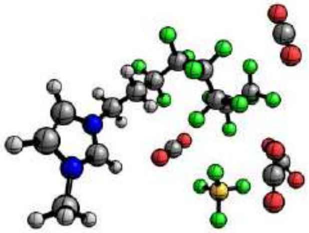	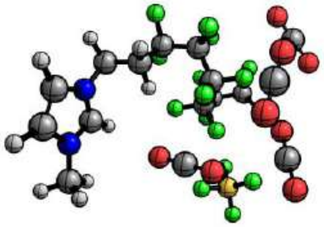	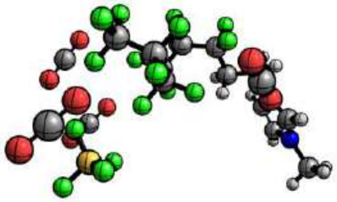	
13.32	13.42	14.45	14.80	

Table S7. Representations of lowest energy forms for $5CO_2[C_8H_4F_{13}mim]^+[BF_4]^-$ at the M06-2X-D3/6-31G(d,p) level with an implicit solvent model PCM. Relative energies are listed in kcal/mol.

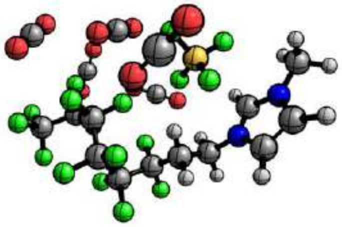	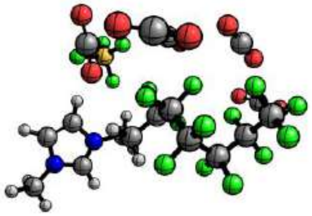	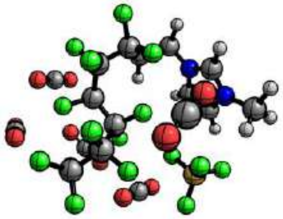	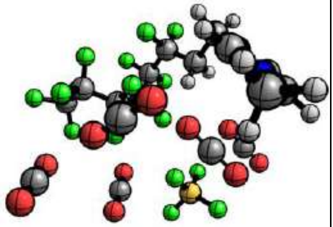	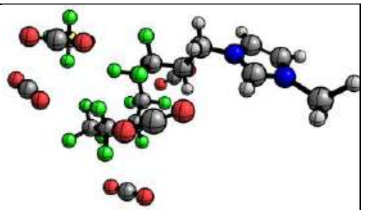
0.00	0.27	1.15	1.65	1.73

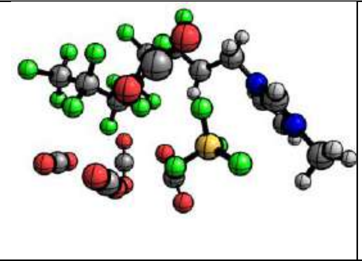	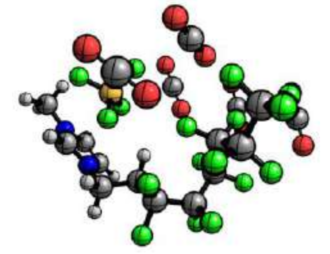	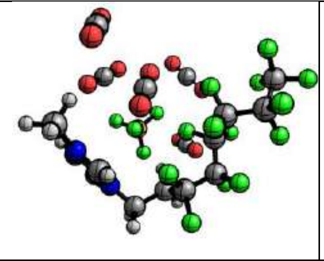	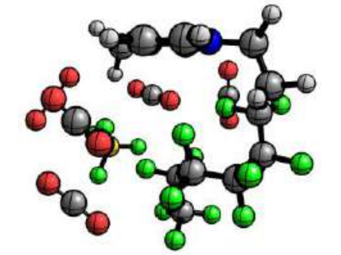	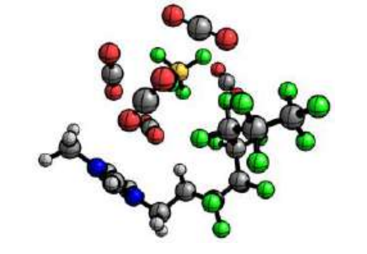
2.13	2.18	3.51	4.55	4.56
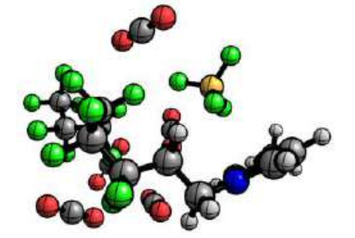	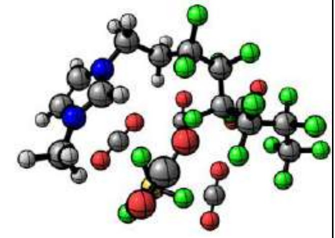	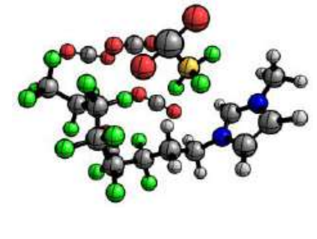	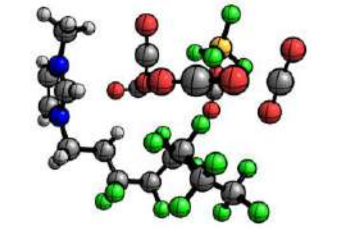	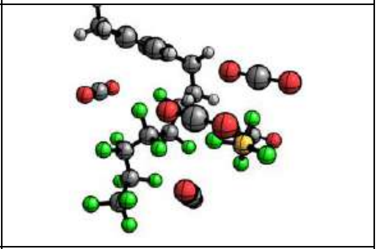
4.66	5.79	5.85	7.52	7.73
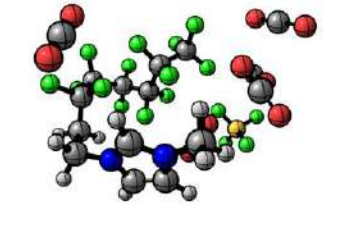	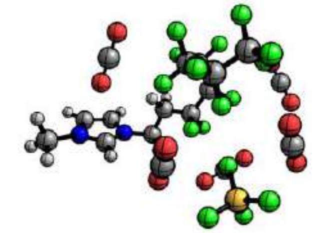	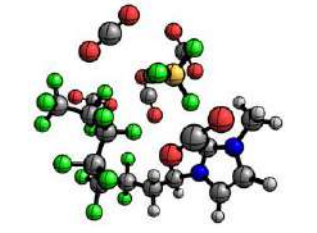	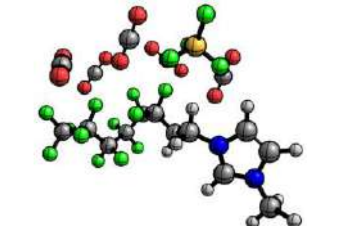	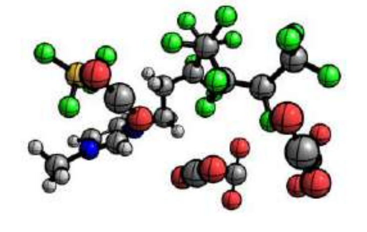
7.91	7.93	8.08	8.49	9.48
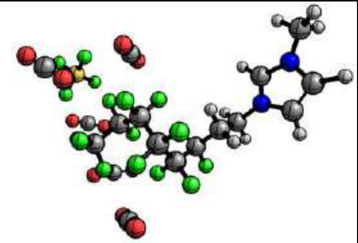	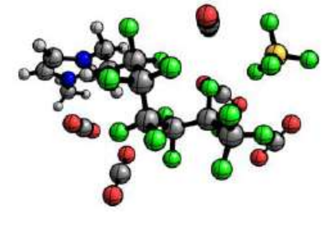	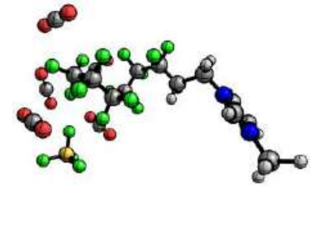	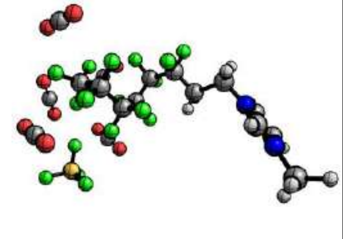	
12.94	14.50	15.04	17.64	

Table S8. Representations of lowest energy forms for $1CO_2[C_8H_4F_{13}mim]^+[TFO]^-$ at the M06-2X-D3/6-31G(d,p) level with an implicit solvent model PCM. Relative energies are listed in kcal/mol.

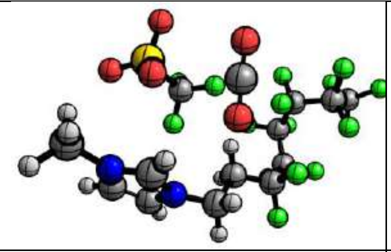	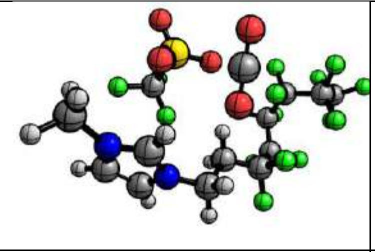	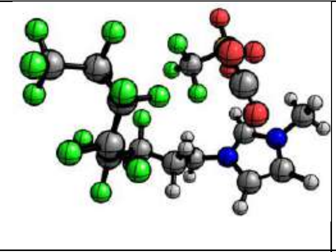	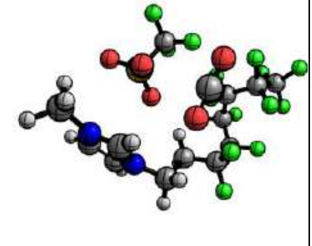	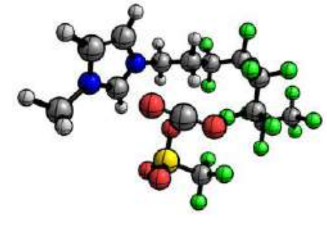
0.00	0.18	0.44	0.57	1.03
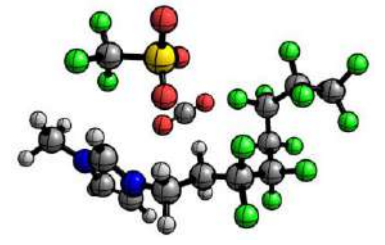	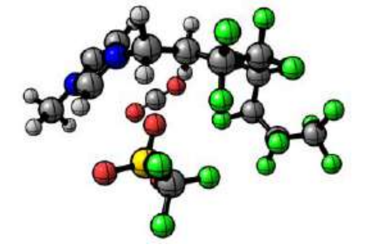	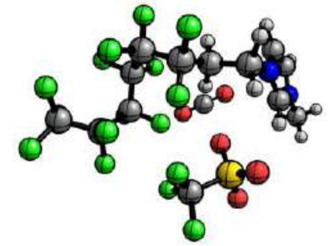	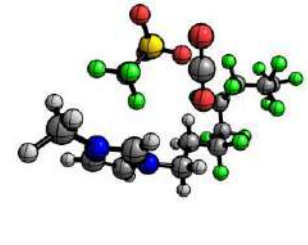	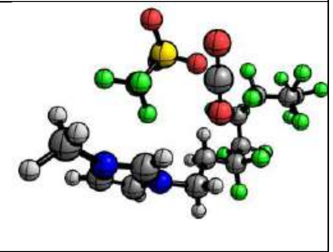
1.07	1.14	1.49	1.50	1.52
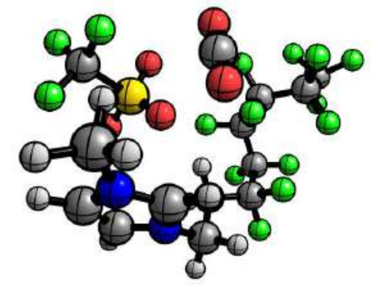	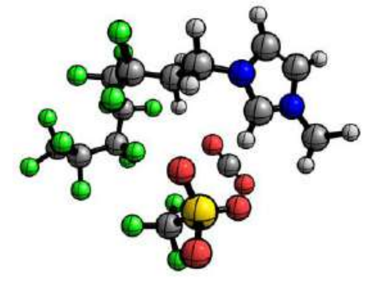	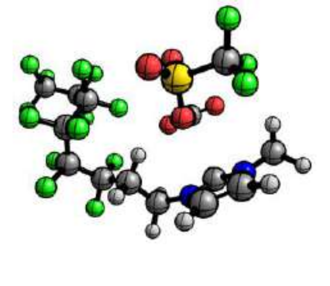	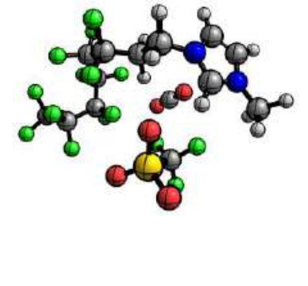	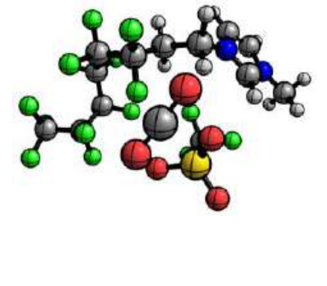
1.62	1.64	2.27	2.38	2.47

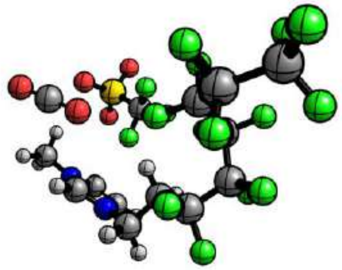	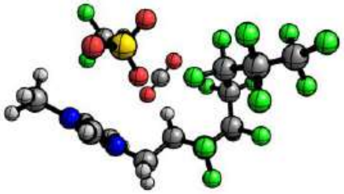	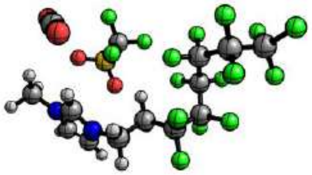	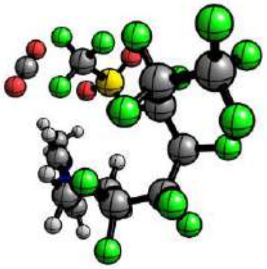	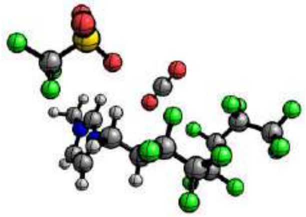
2.88	2.93	3.48	3.50	3.51
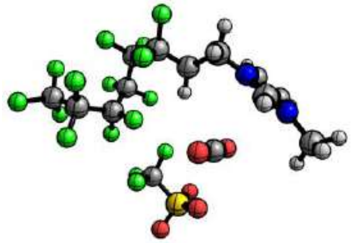	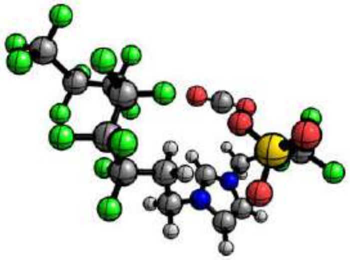	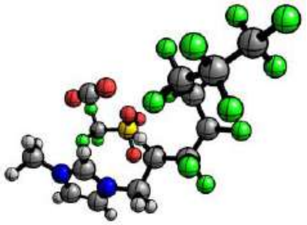	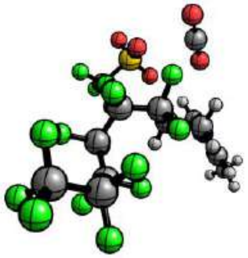	
3.58	3.80	4.21	6.73	

Table S9. Representations of lowest energy forms for $2CO_2[C_8H_4F_{13}mim]^+[TFO]^-$ at the M06-2X-D3/6-31G(d,p) level with an implicit solvent model PCM. Relative energies are listed in kcal/mol.

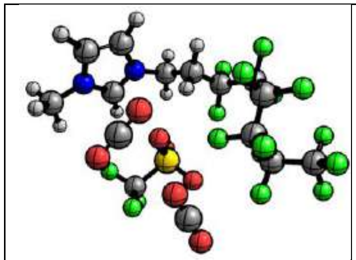	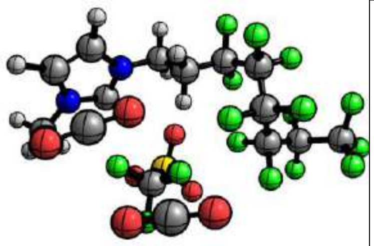	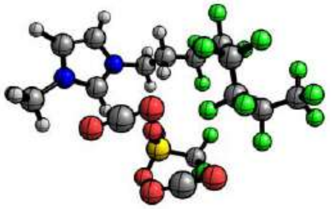	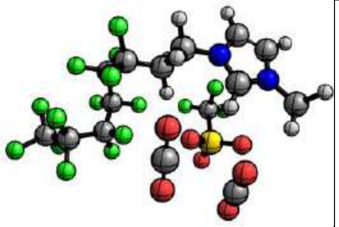	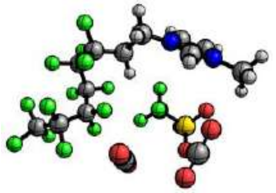
0.00	0.32	0.81	1.00	1.53

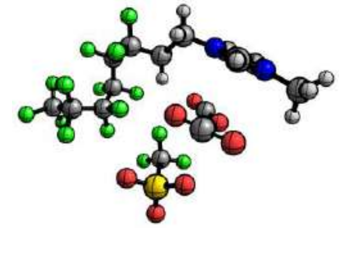	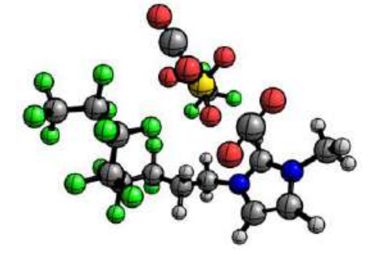	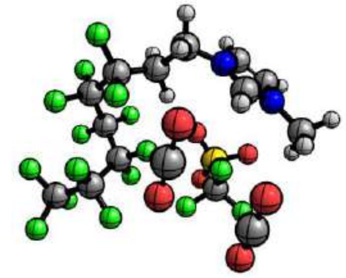	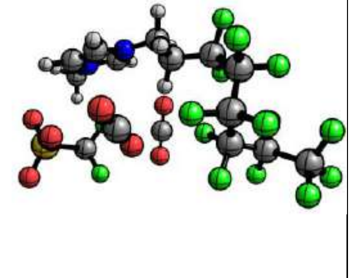	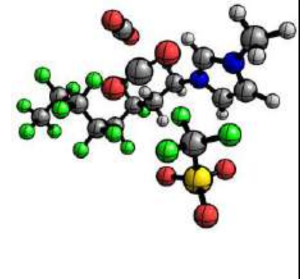
1.57	1.60	1.72	1.89	2.57
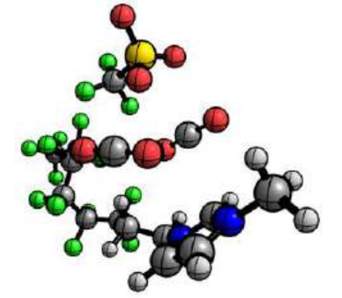	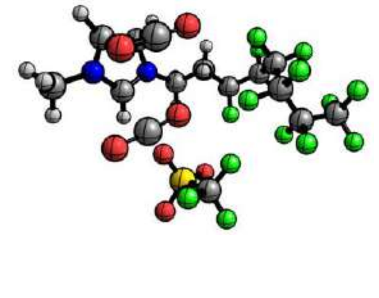	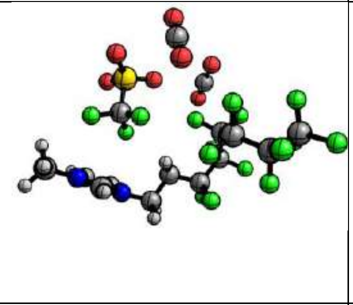	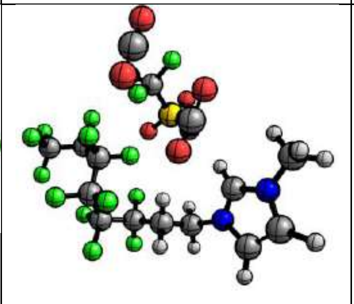	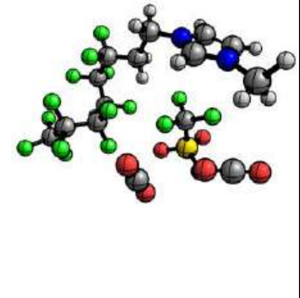
2.58	2.64	2.74	2.95	2.96
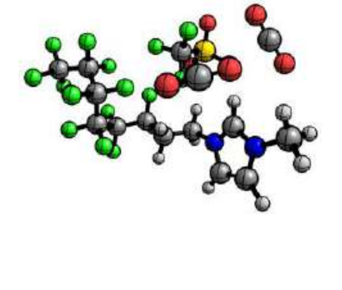	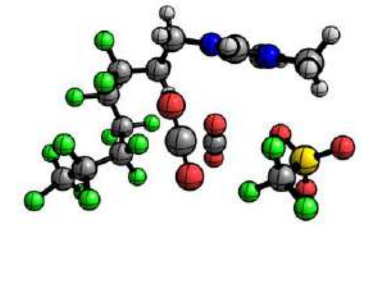	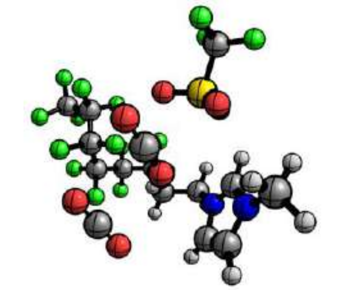	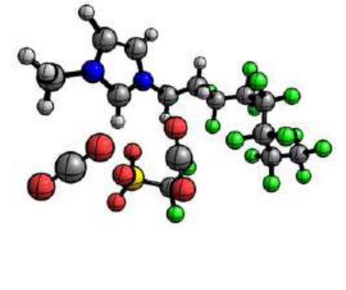	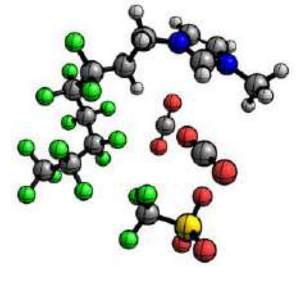
3.06	3.61	3.67	4.41	4.42

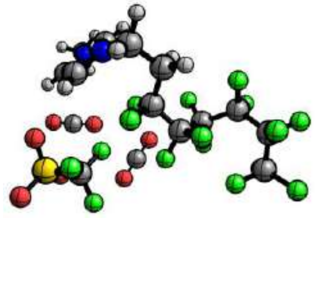	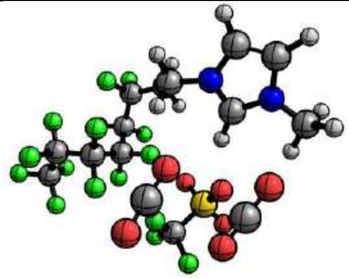	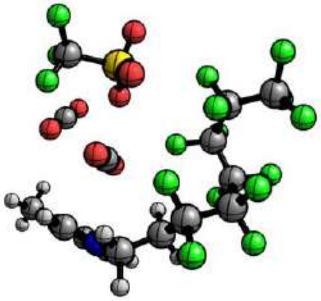	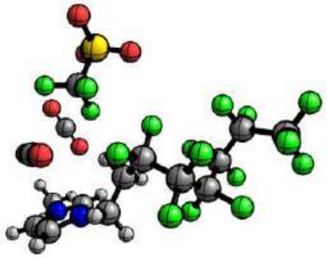	
4.80	5.59	6.25	7.64	

Table S10. Representations of lowest energy forms for $3CO_2[C_8H_4F_{13}mim]^+[TFO]^-$ at the M06-2X-D3/6-31G(d,p) level with an implicit solvent model PCM. Relative energies are listed in kcal/mol.

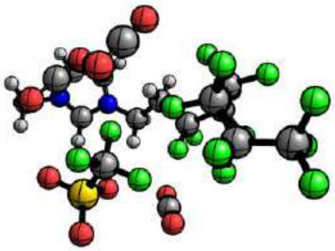	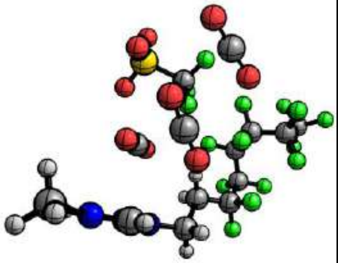	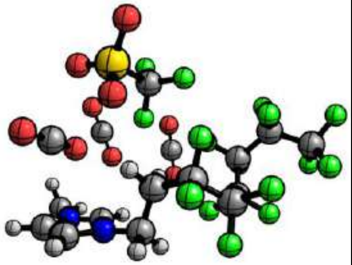	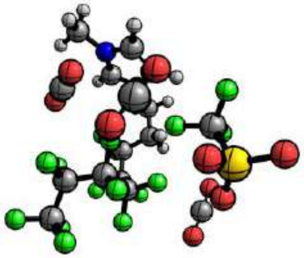	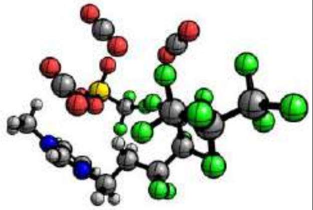
0.00	1.15	1.64	1.75	2.04
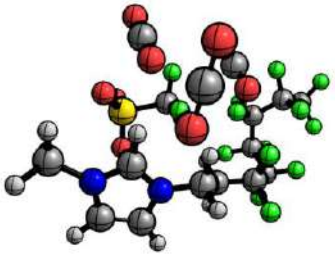	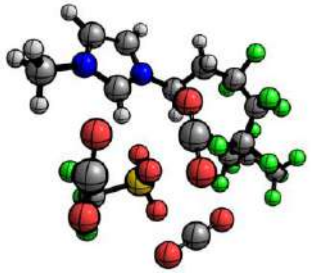	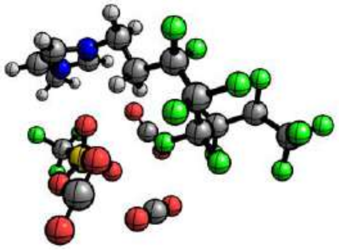	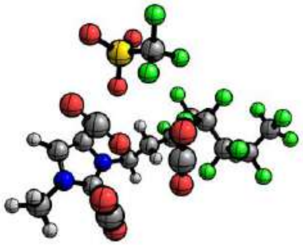	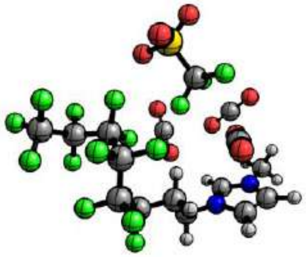
2.56	2.61	2.76	2.92	3.45

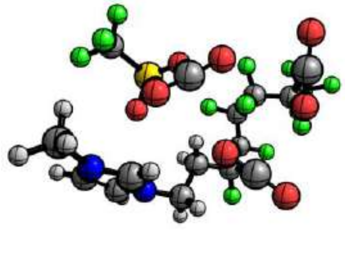	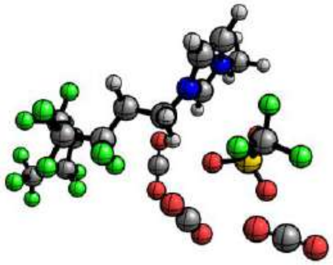	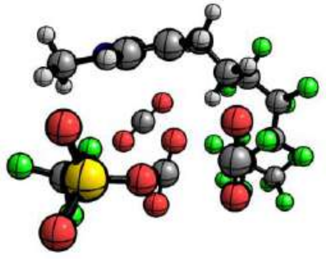	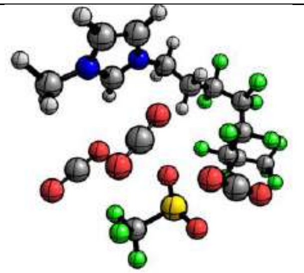	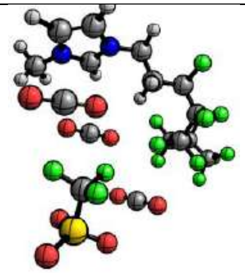
4.09	4.25	4.44	4.63	4.71
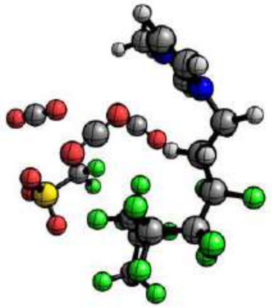	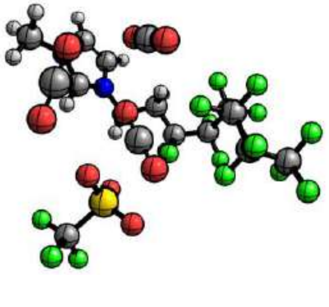	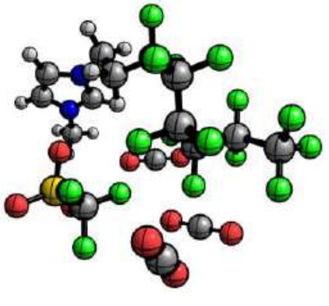	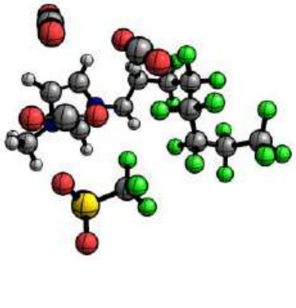	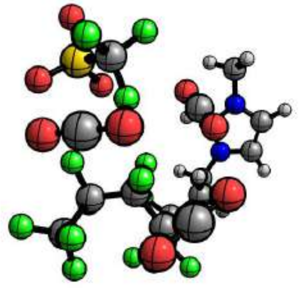
4.89	4.99	5.36	5.96	6.37
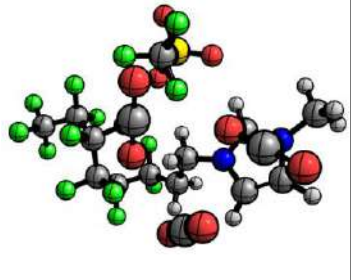	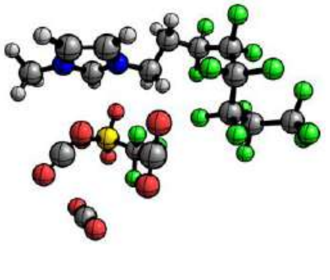	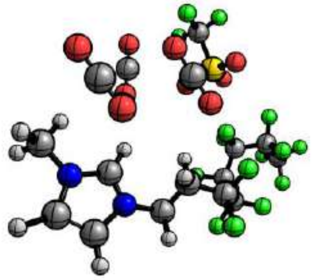		
6.64	7.73	7.73		

Table S11. Representations of lowest energy forms for $4CO_2[C_8H_4F_{13}mim]^+[TFO]^-$ at the M06-2X-D3/6-31G(d,p) level with an implicit solvent model PCM. Relative energies are listed in kcal/mol.

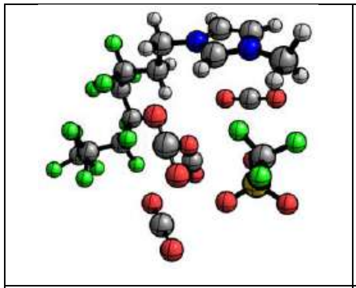	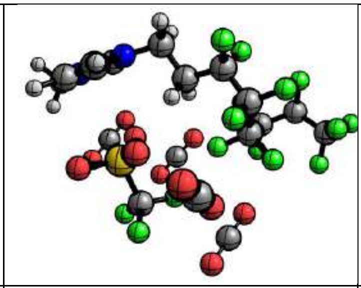	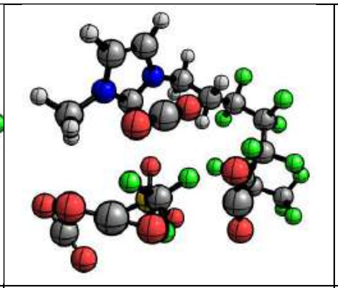	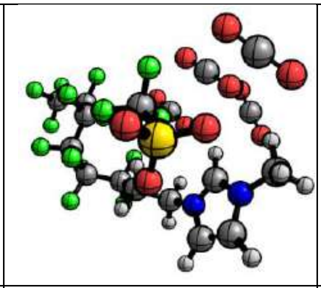	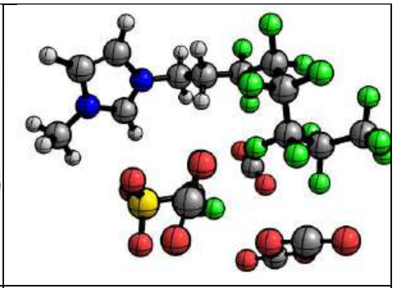
0.00	1.07	1.25	1.82	1.97
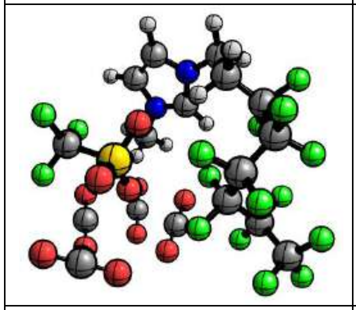	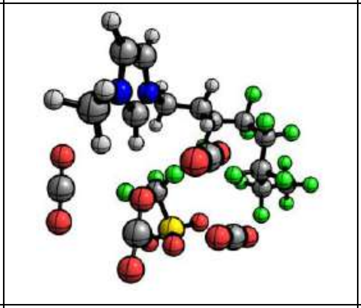	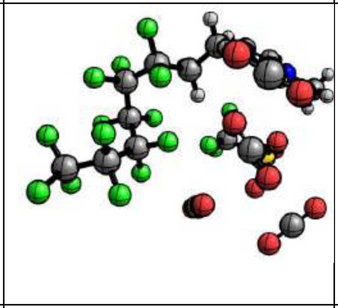	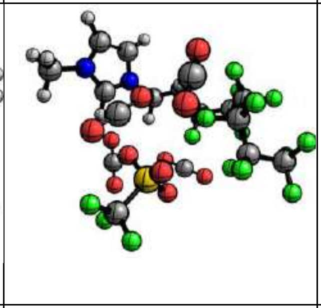	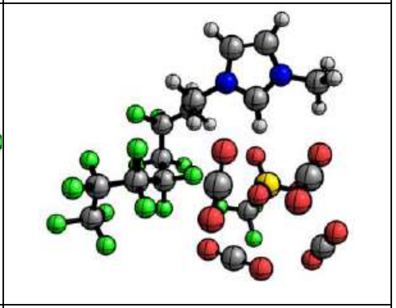
2.73	2.86	2.90	3.05	3.37
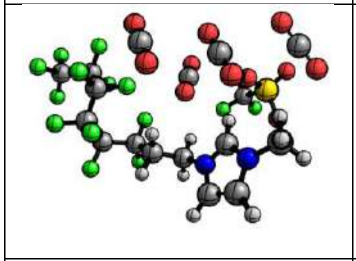	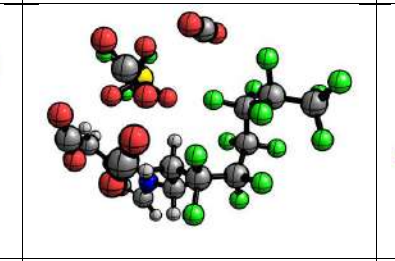	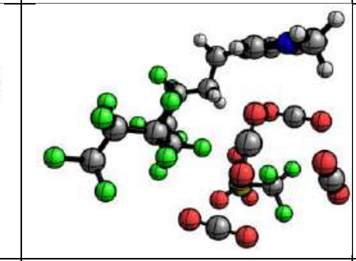	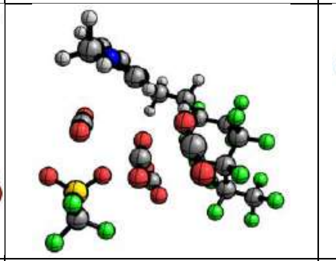	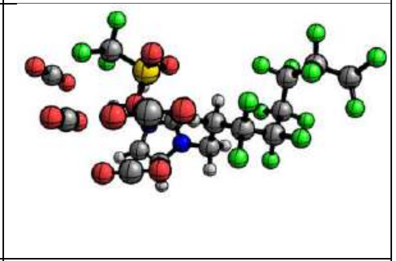
3.56	3.81	4.28	4.55	4.56

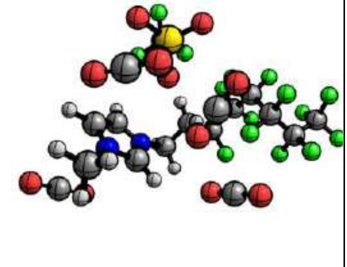	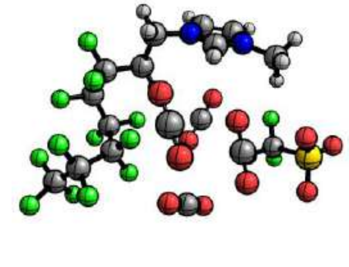	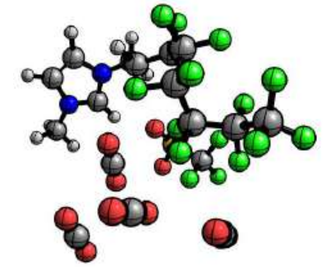	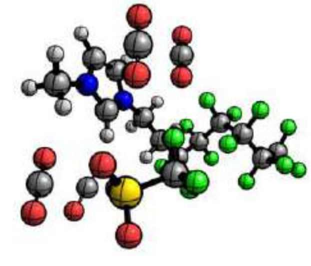	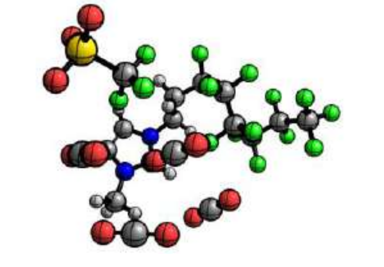
4.67	4.75	5.39	5.95	6.26
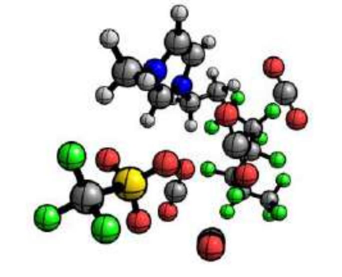	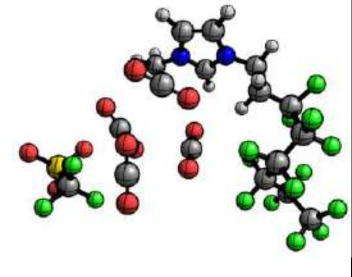	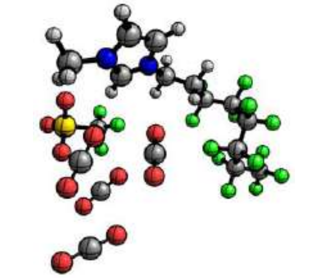	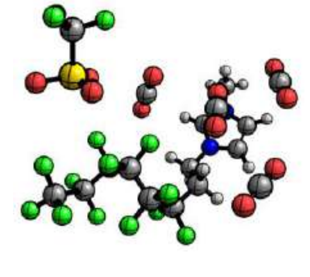	
6.41	8.48	9.38	14.23	

Table S12. Representations of lowest energy forms for $5CO_2[C_8H_4F_{13}mim]^+[TFO]^-$ at the M06-2X-D3/6-31G(d,p) level with an implicit solvent model PCM. Relative energies are listed in kcal/mol.

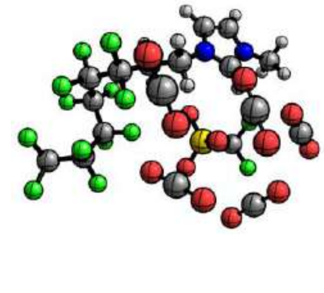	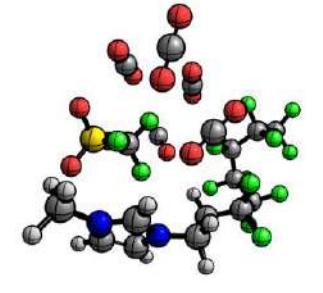	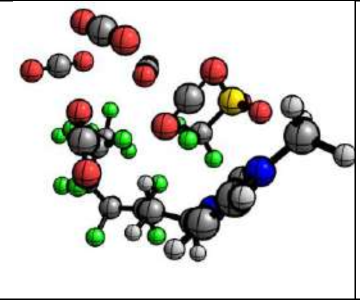	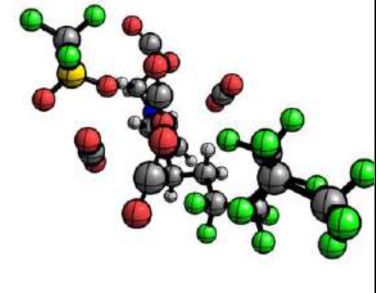	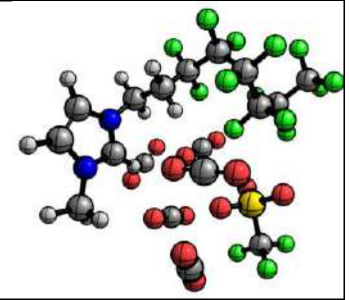
0.00	0.053	0.43	1.88	2.14

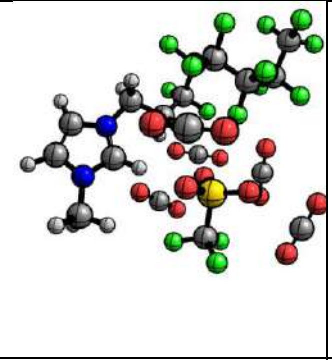	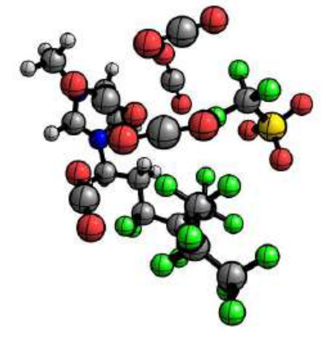	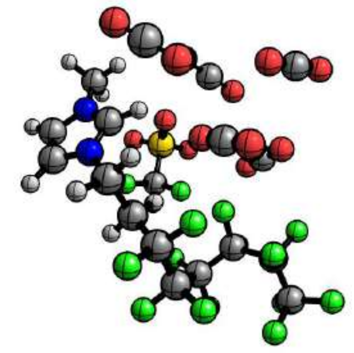	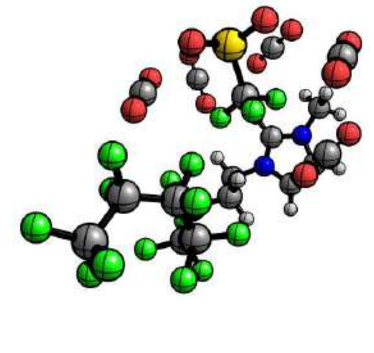	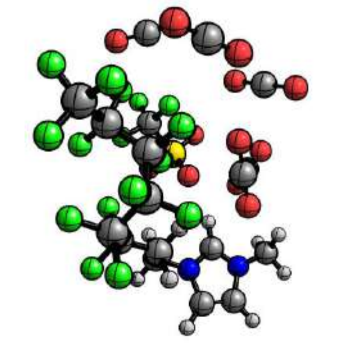
2.33	2.92	3.18	3.52	4.54
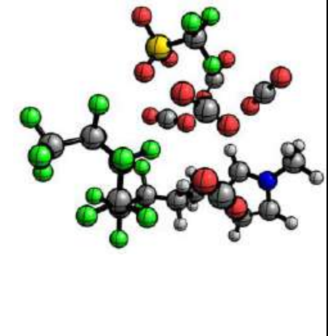	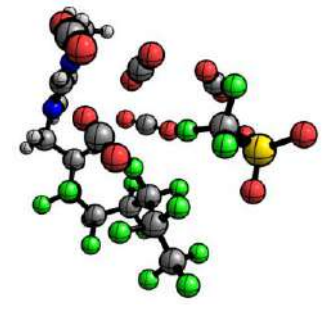	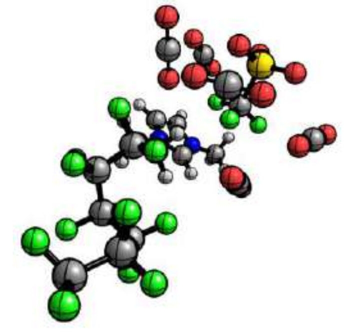	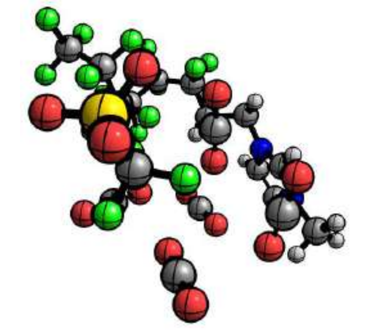	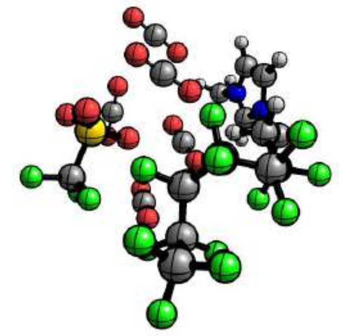
4.59	4.65	4.98	5.00	5.01
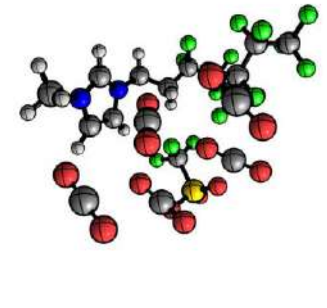	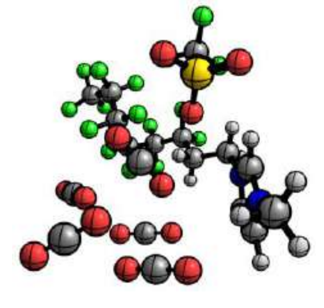	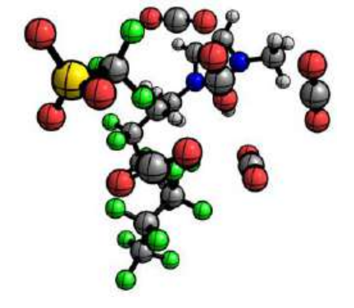	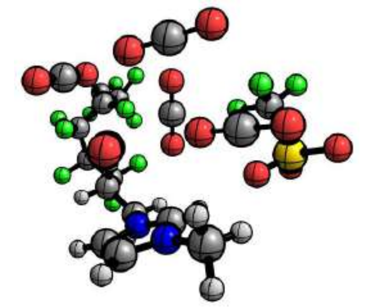	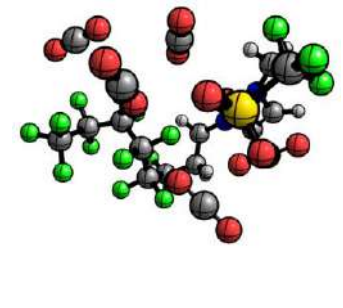
5.04	5.28	5.29	5.38	5.56

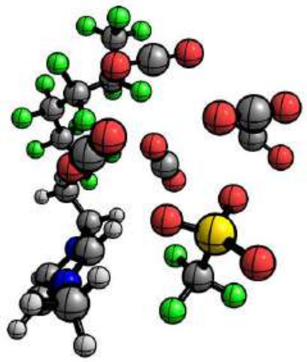	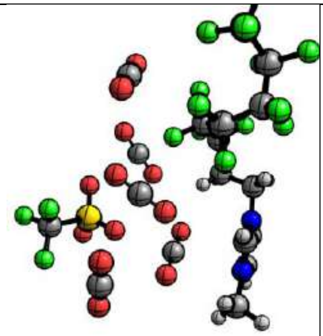	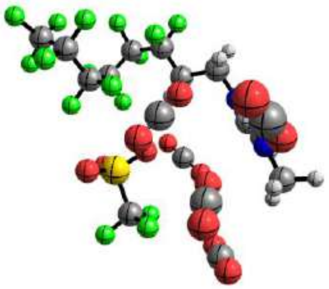	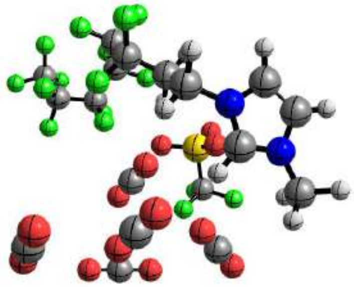	
5.59	5.81	8.54	11.08	

Table S13. Representations of lowest energy forms for $1CO_2[Dmim]^+[BF_4]^-$ at the M06-2X-D3/6-31G(d,p) level with an implicit solvent model PCM. Relative energies are listed in kcal/mol.

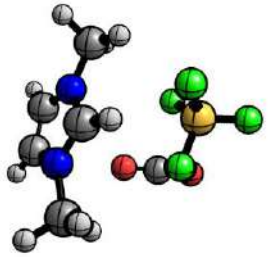	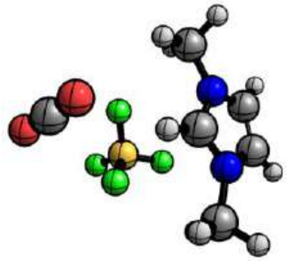	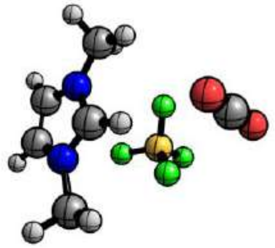	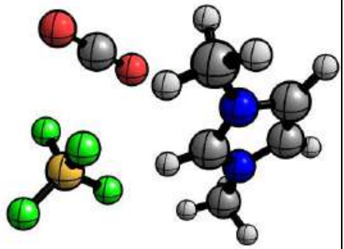	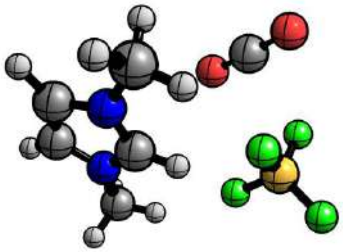
0.00	0.50	0.60	0.66	0.67

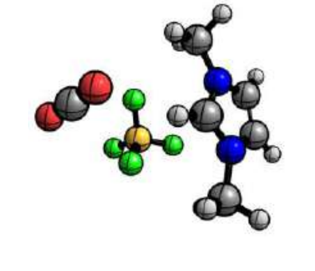	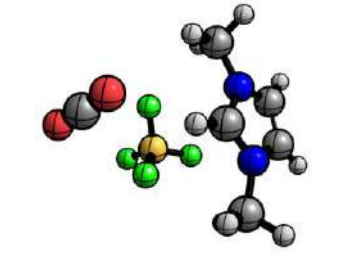	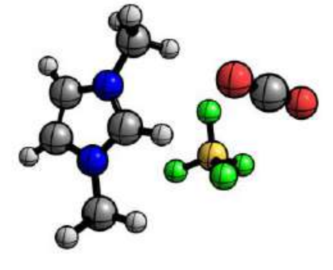	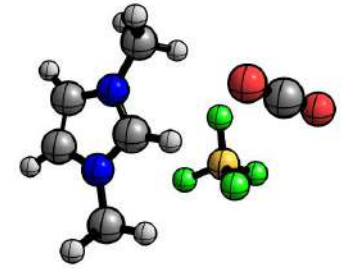	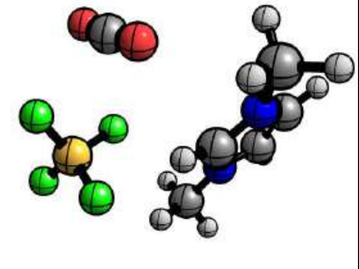
0.67	0.68	0.86	1.00	1.05
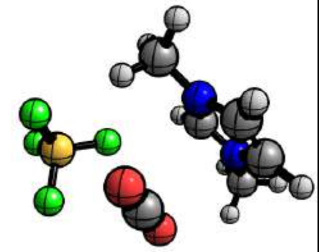	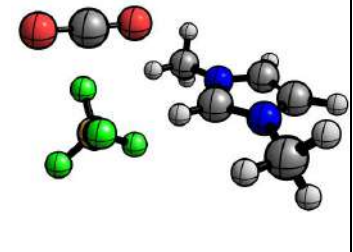	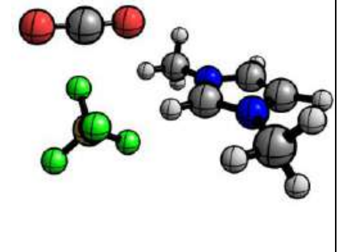	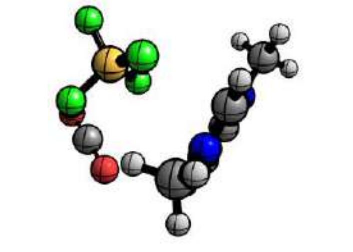	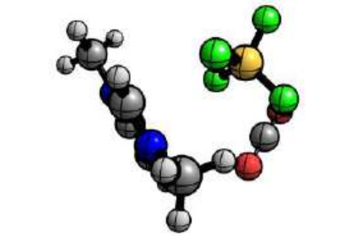
1.12	1.31	1.37	1.44	1.45
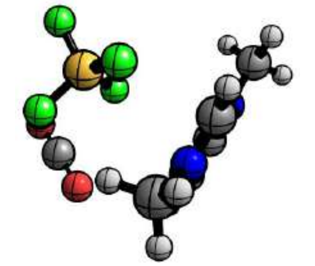	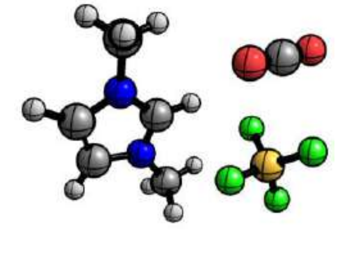	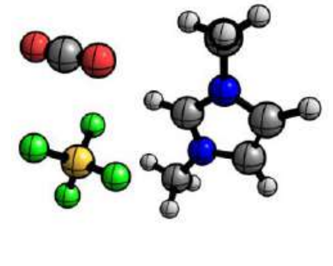	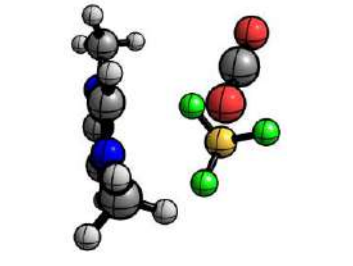	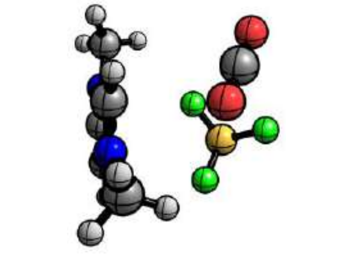
1.46	1.67	1.68	1.69	1.70

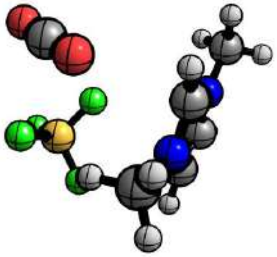	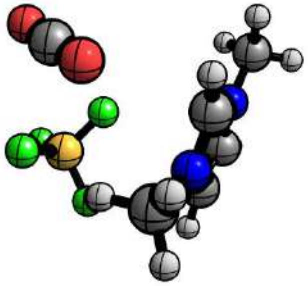	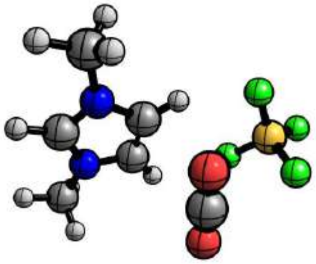	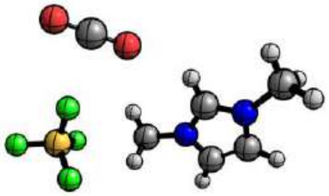	
1.98	1.99	3.05	7.52	

Table S14. Representations of lowest energy forms for $2CO_2[Dmim]^+[BF_4]^-$ at the M06-2X-D3/6-31G(d,p) level with an implicit solvent model PCM. Relative energies are listed in kcal/mol.

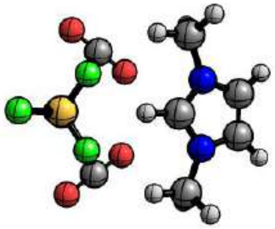	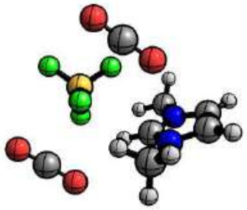	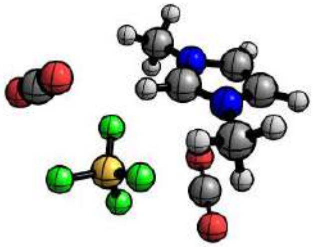	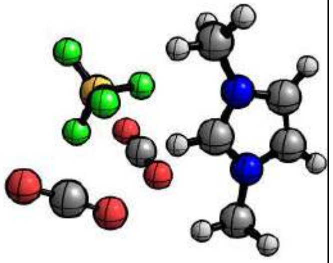	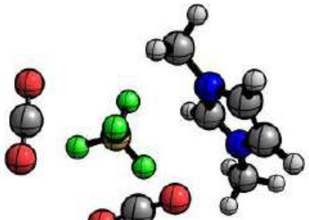
0.00	0.41	0.82	0.92	0.96
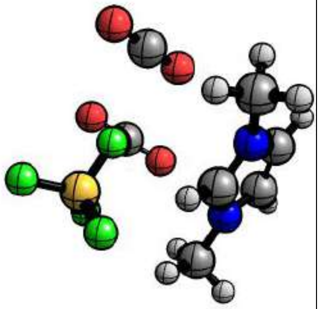	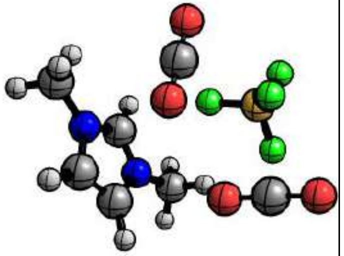	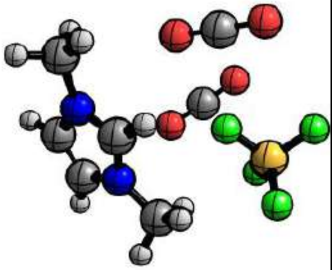	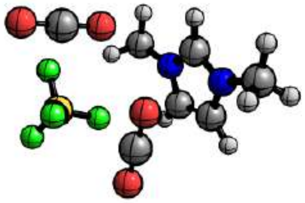	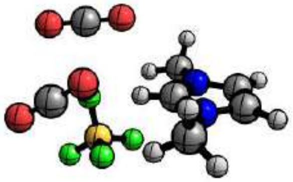
1.15	1.18	1.22	1.26	1.36

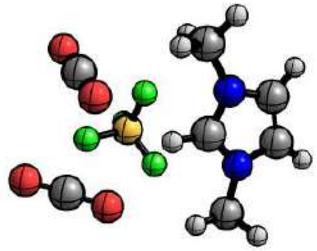	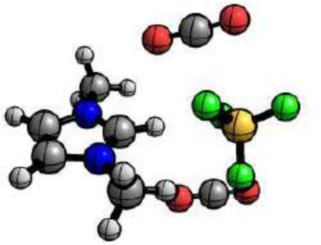	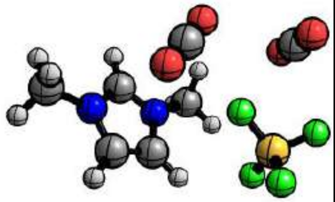	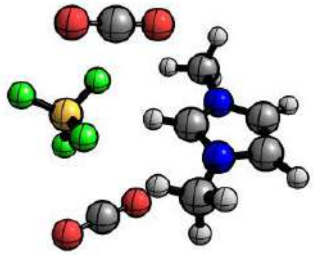	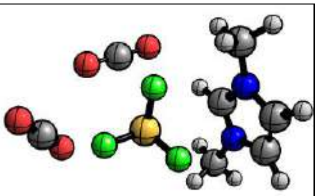
1.67	1.72	2.01	2.05	2.17
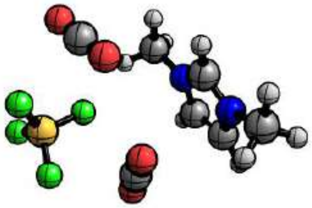	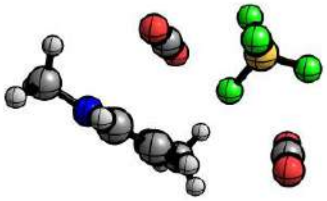	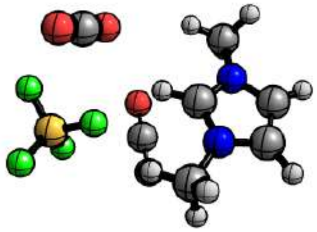	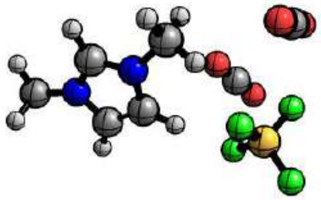	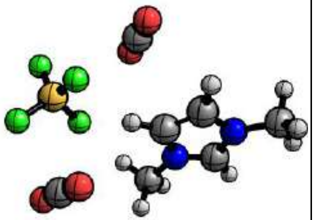
2.36	2.71	2.90	3.57	3.93
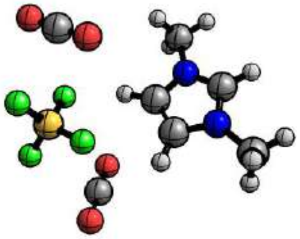	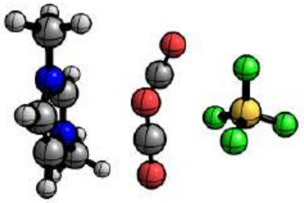	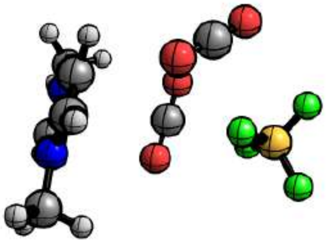	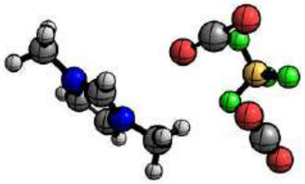	
4.20	4.21	5.47	6.06	

Table S15. Representations of lowest energy forms for $3CO_2[Dmim]^+[BF_4]^-$ at the M06-2X-D3/6-31G(d,p) level with an implicit solvent model PCM. Relative energies are listed in kcal/mol.

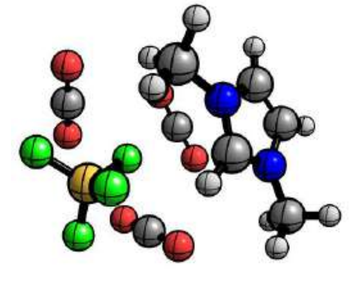	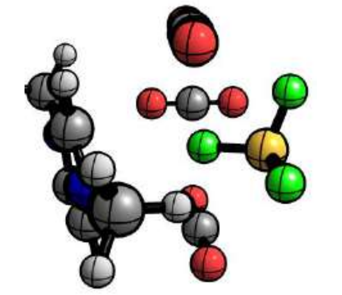	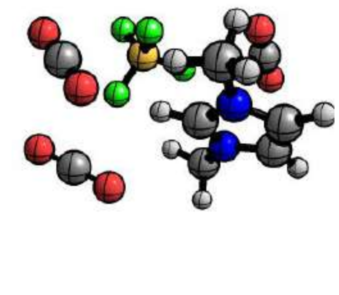	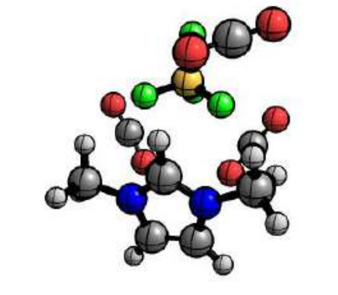	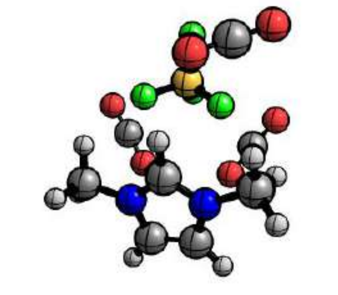
0.00	1.85	2.42	2.78	2.89
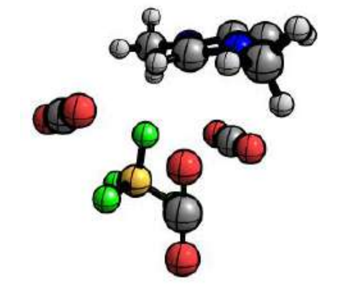	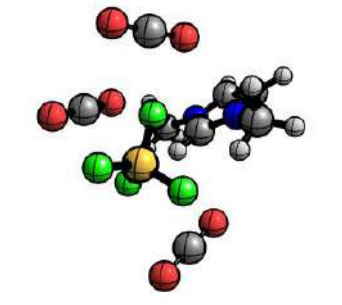	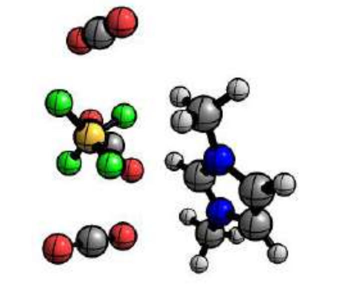	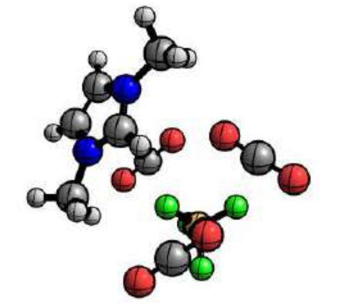	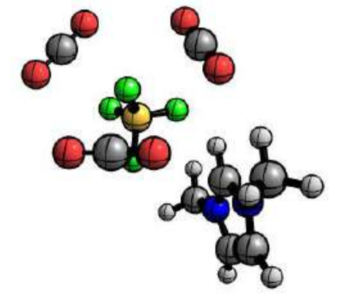
2.92	3.31	3.40	3.78	3.85
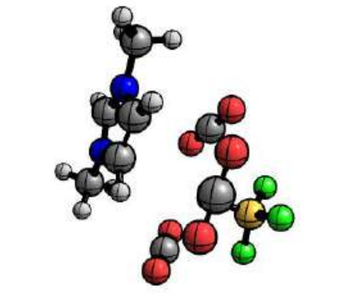	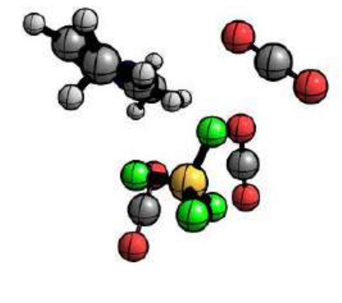	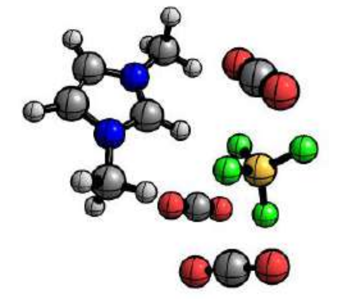	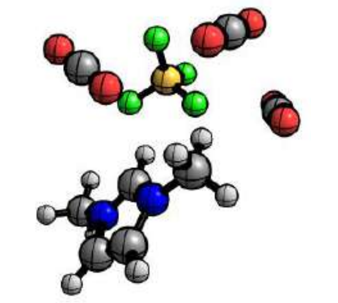	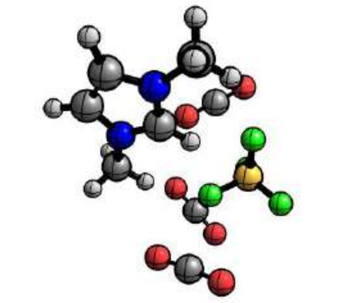
3.91	3.93	4.01	4.22	4.23

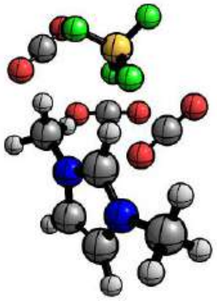	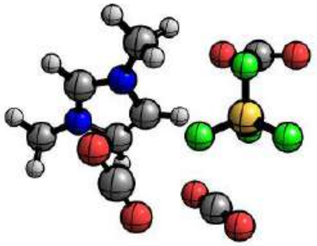	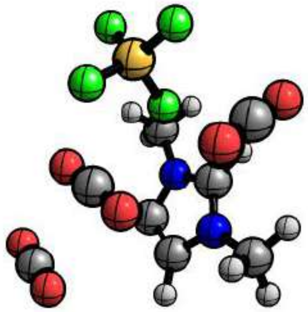	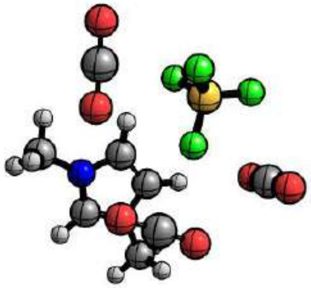	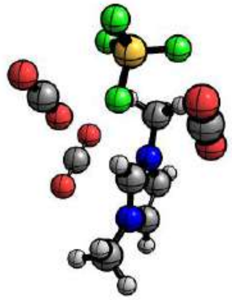
4.23	4.42	5.05	5.45	6.04
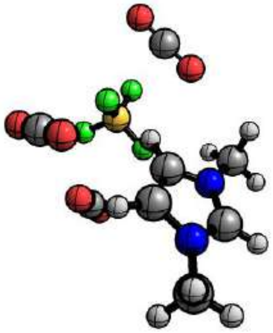	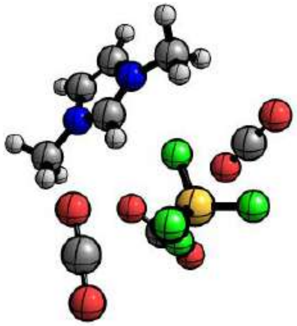	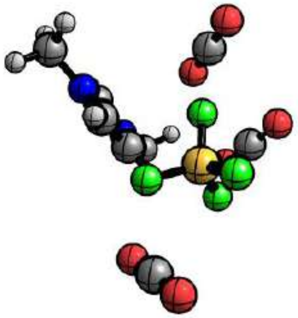	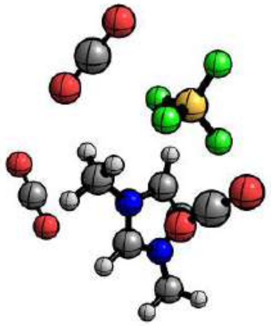	
6.23	6.39	6.52	10.45	

Table S16. Representations of lowest energy forms for $4CO_2[Dmim]^+[BF_4]^-$ at the M06-2X-D3/6-31G(d,p) level with an implicit solvent model PCM. Relative energies are listed in kcal/mol.

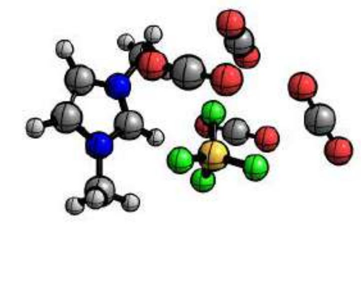	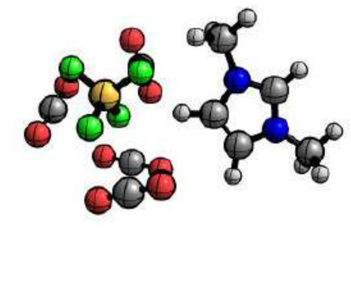	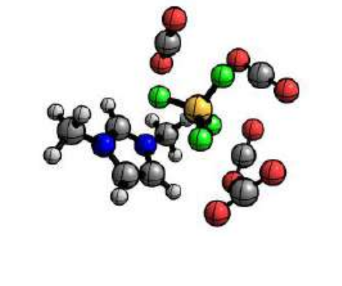	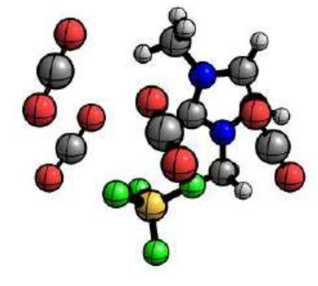	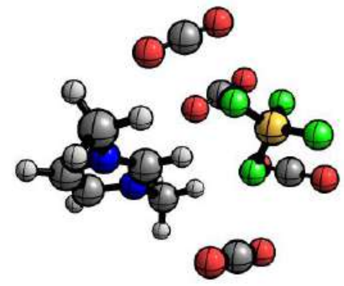
0.00	2.02	2.07	2.08	2.64
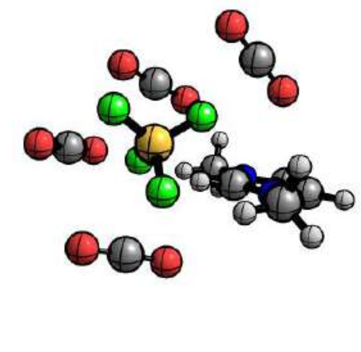	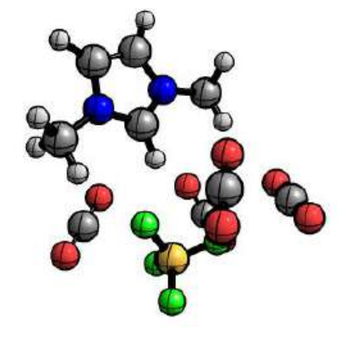	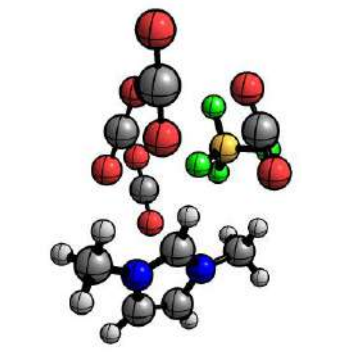	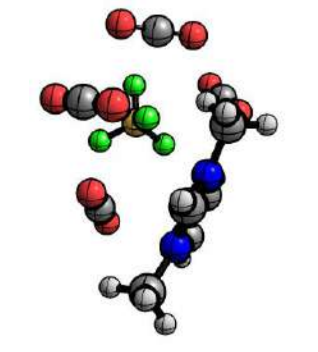	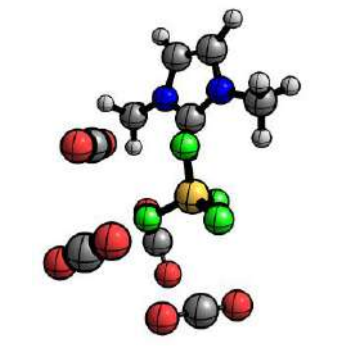
2.97	2.99	3.05	3.31	3.43
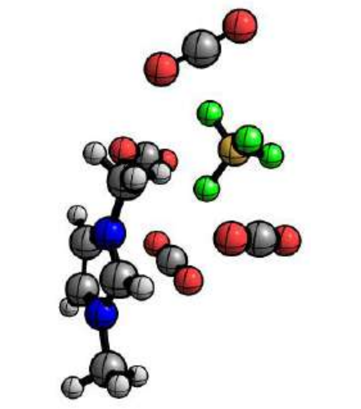	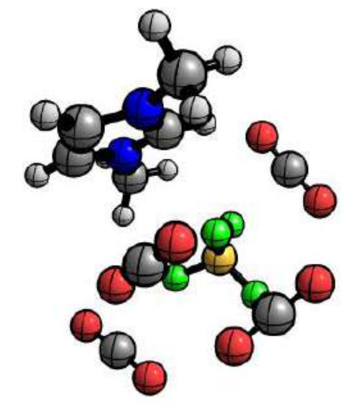	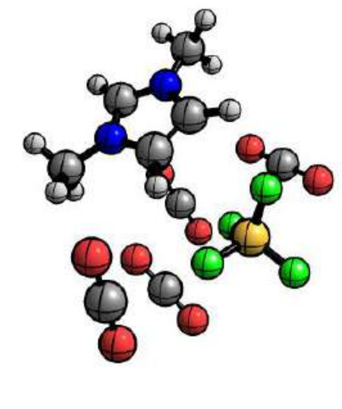	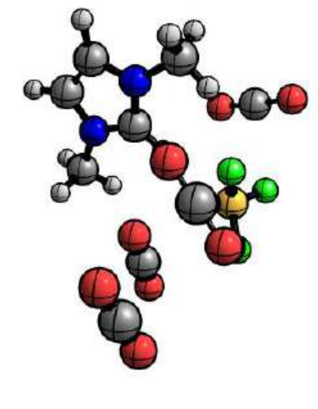	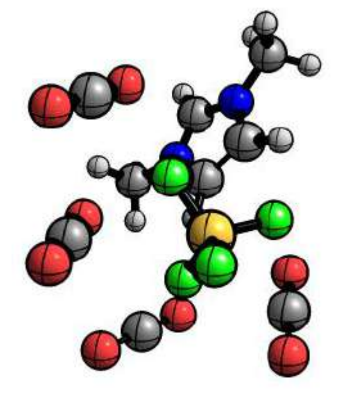
3.71	4.04	4.12	4.17	4.18

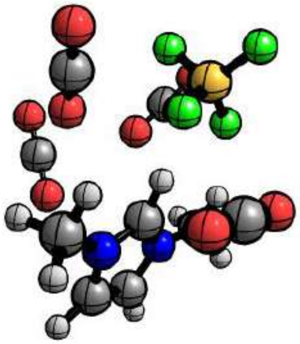	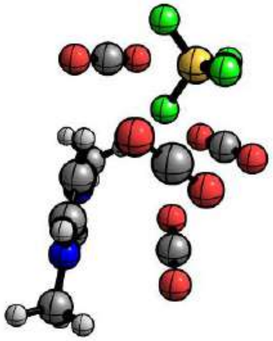	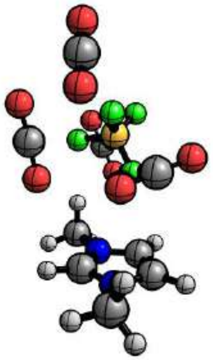	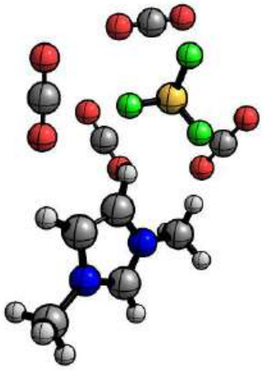	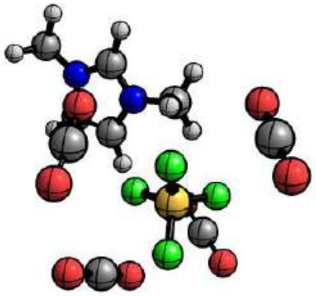
4.19	4.28	4.69	5.70	5.81
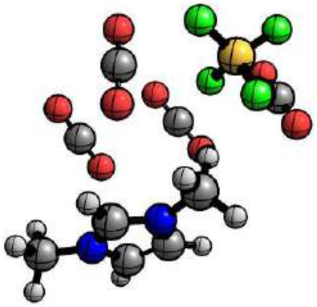	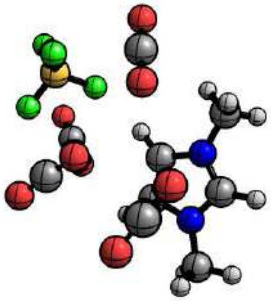	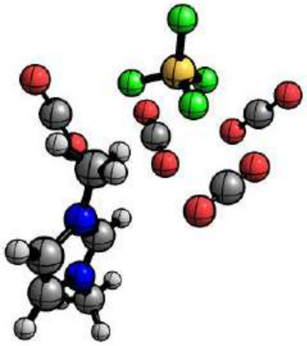	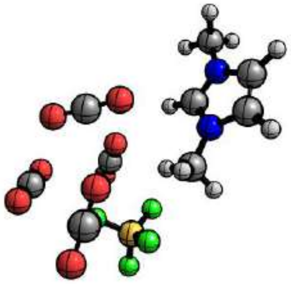	
5.84	6.25	6.34	6.43	

Table S17. Representations of lowest energy forms for $5CO_2[Dmim]^+[BF_4]^-$ at the M06-2X-D3/6-31G(d,p) level with an implicit solvent model PCM. Relative energies are listed in kcal/mol.

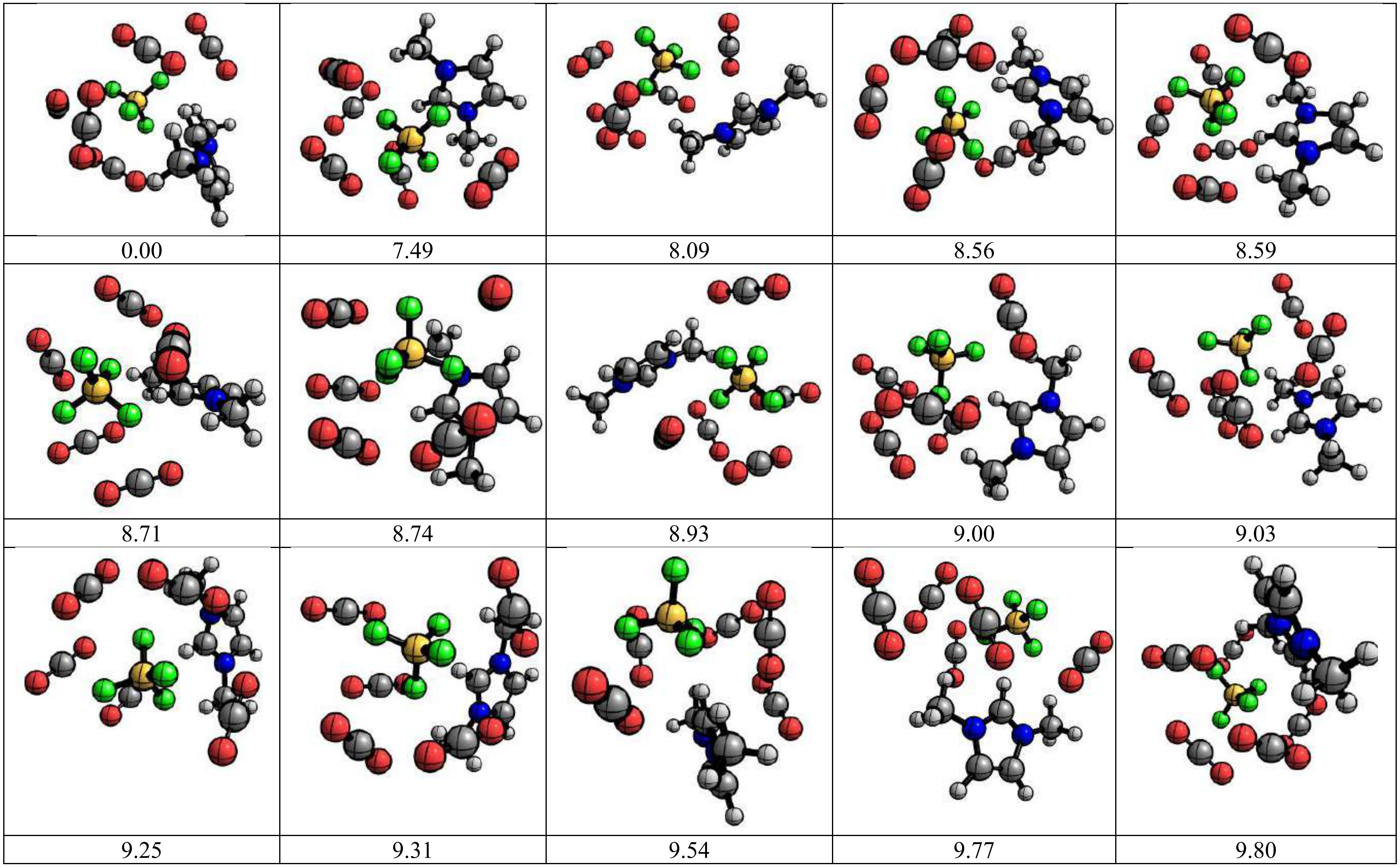
0.00
7.49
8.09
8.56
8.59
8.71
8.74
8.93
9.00
9.03
9.25
9.31
9.54
9.77
9.80

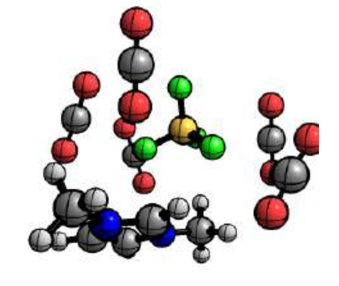	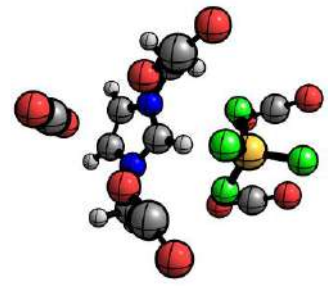	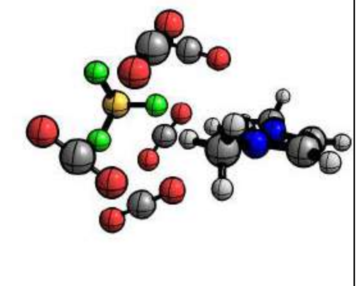	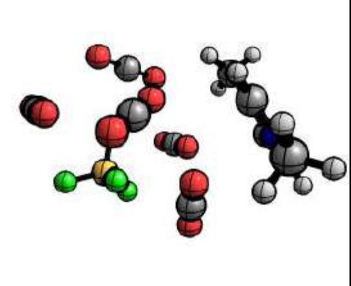	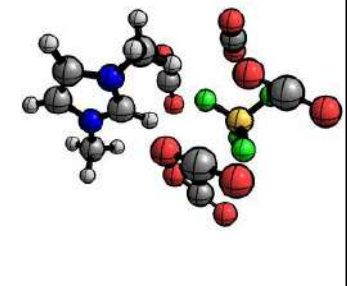
10.14	10.42	10.48	10.78	10.79
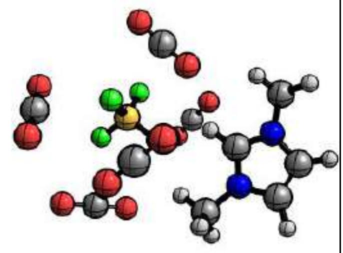	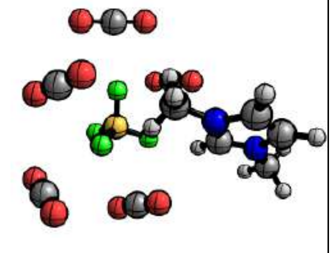	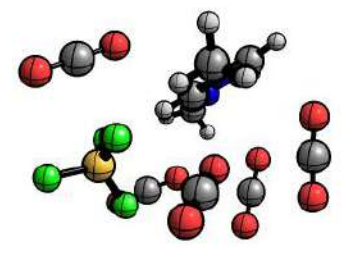	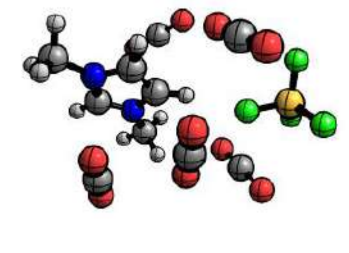	
11.91	11.97	12.13	16.80	

Table S18. Representations of lowest energy forms for $1CO_2[Dmim]^+[TFO]^-$ at the M06-2X-D3/6-31G(d,p) level with an implicit solvent model PCM. Relative energies are listed in kcal/mol.

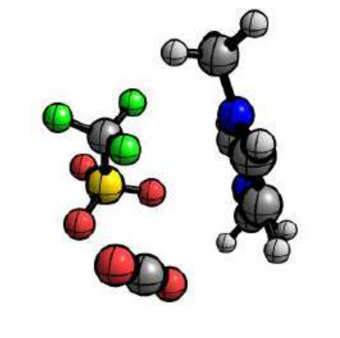	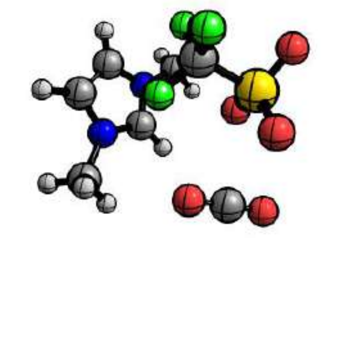	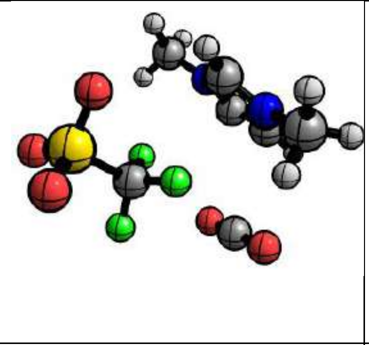	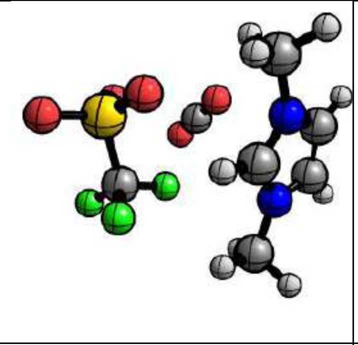	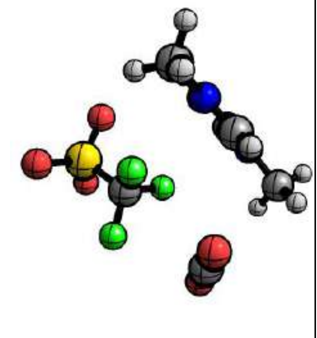
0.00	0.03	0.55	0.66	0.77

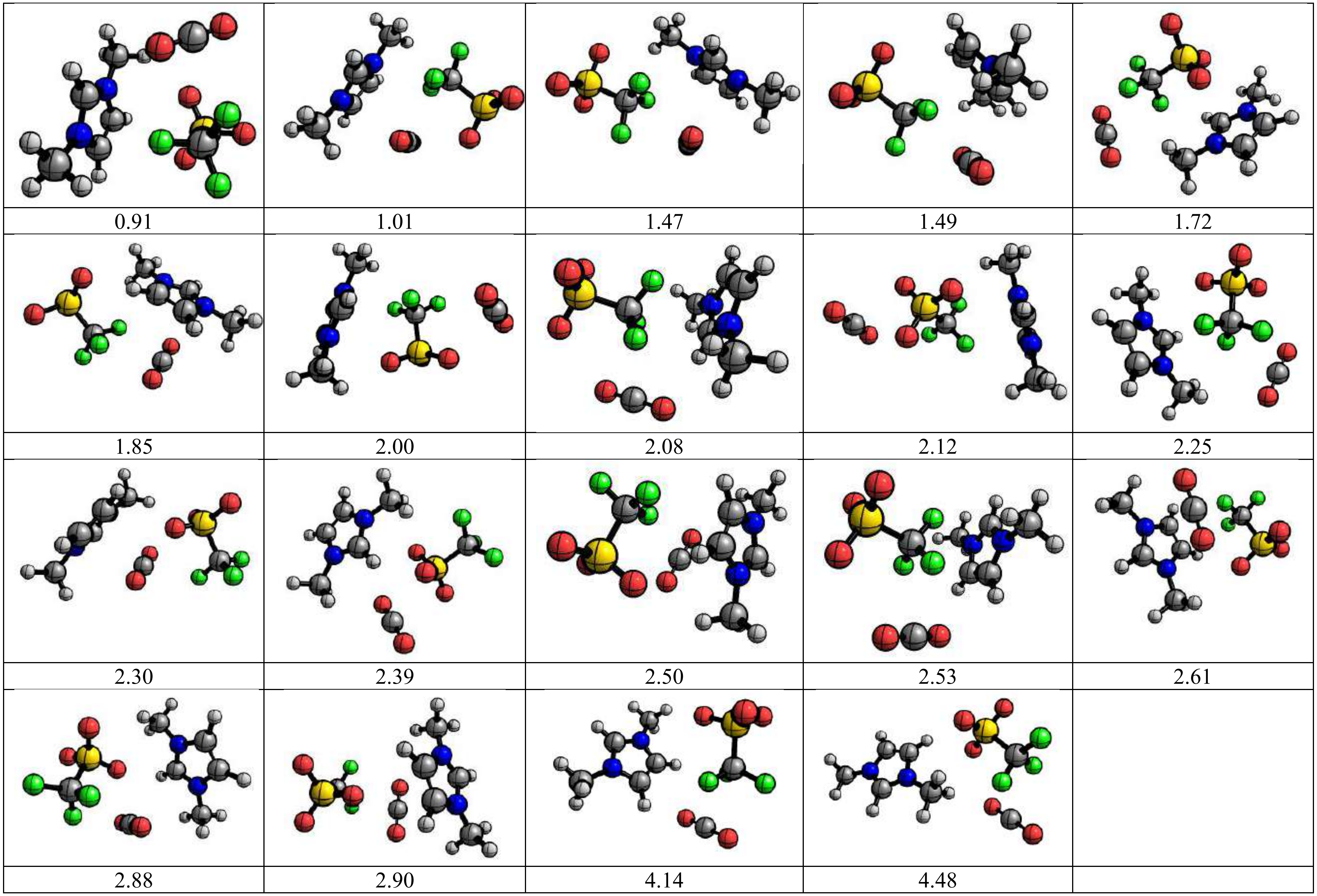
0.91
1.01
1.47
1.49
1.72
1.85
2.00
2.08
2.12
2.25
2.30
2.39
2.50
2.53
2.61
2.88
2.90
4.14
4.48

Table S19. Representations of lowest energy forms for $2CO_2[Dmim]^+[TFO]^-$ at the M06-2X-D3/6-31G(d,p) level with an implicit solvent model PCM. Relative energies are listed in kcal/mol.

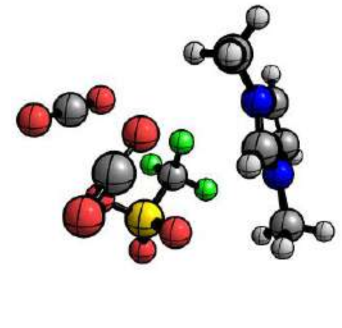	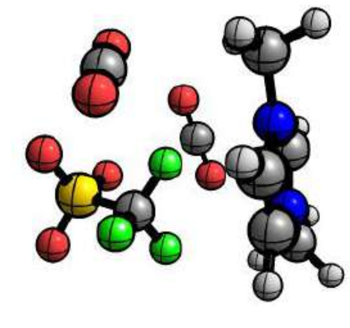	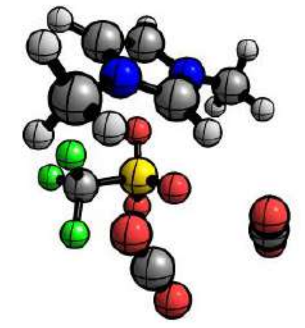	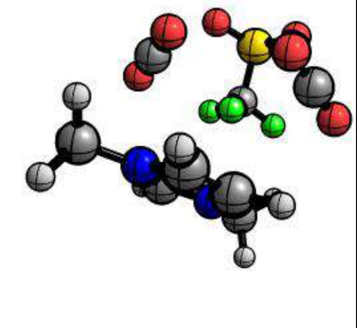	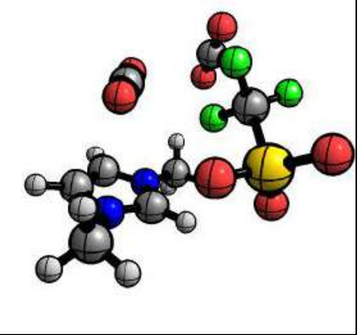
0.00	0.10	0.14	0.31	0.57
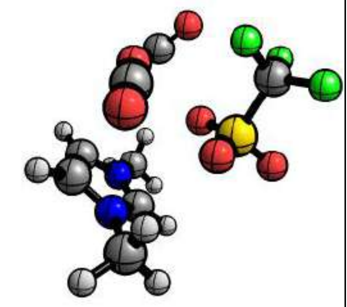	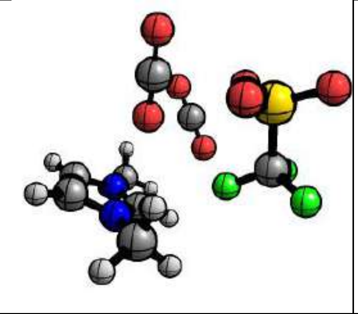	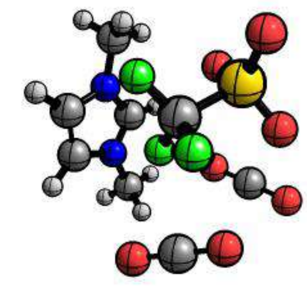	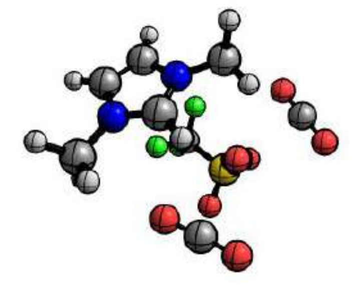	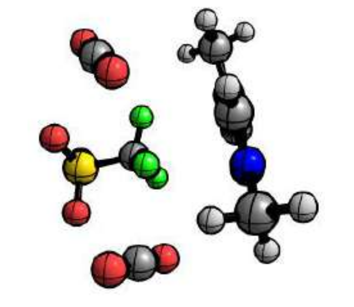
0.89	1.08	1.10	1.12	1.16
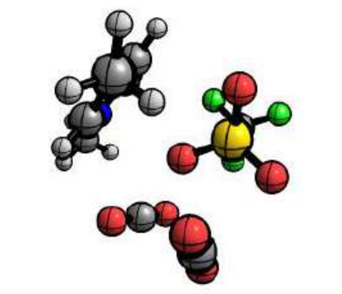	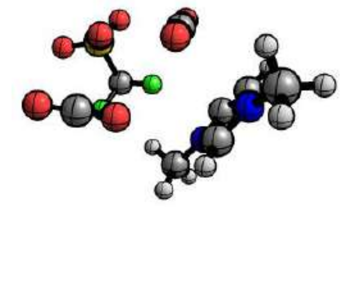	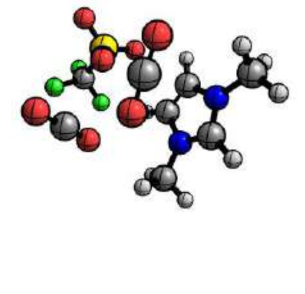	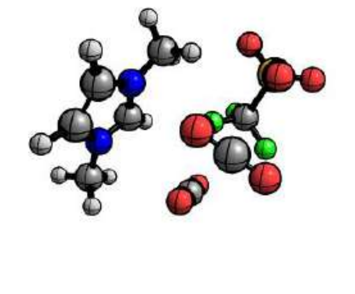	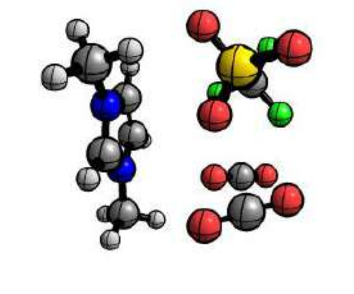

1.34	1.50	1.60	1.65	1.78
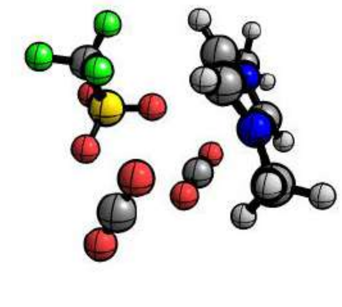	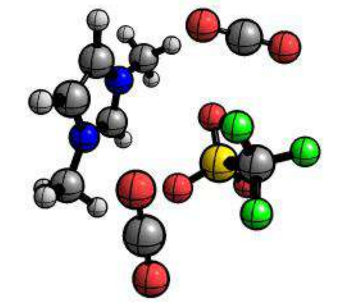	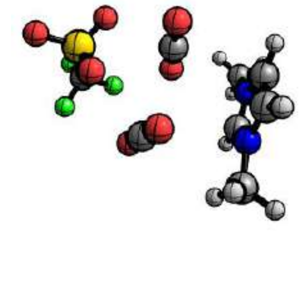	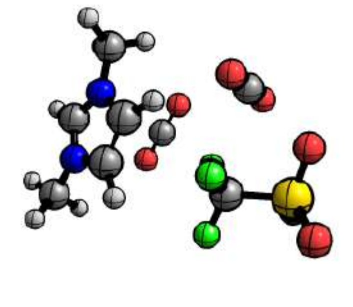	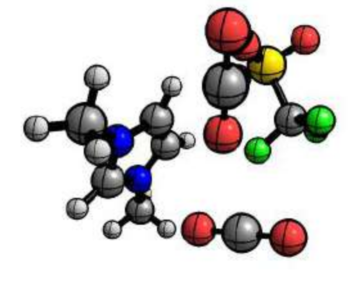
2.28	2.39	2.59	2.89	2.97
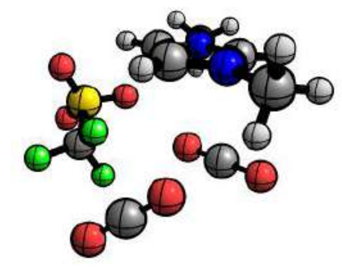	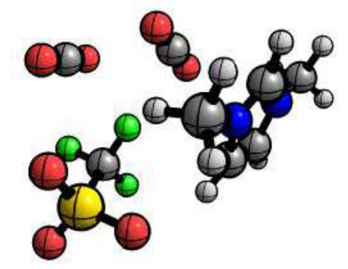	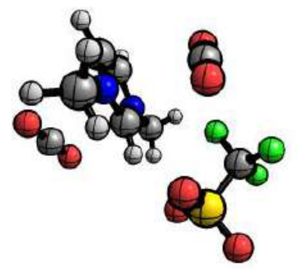	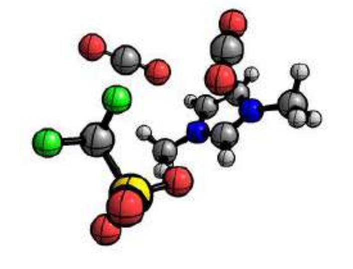	
3.05	3.36	4.14	4.37	

Table S20. Representations of lowest energy forms for $3CO_2[Dmim]^+[TFO]^-$ at the M06-2X-D3/6-31G(d,p) level with an implicit solvent model PCM. Relative energies are listed in kcal/mol.

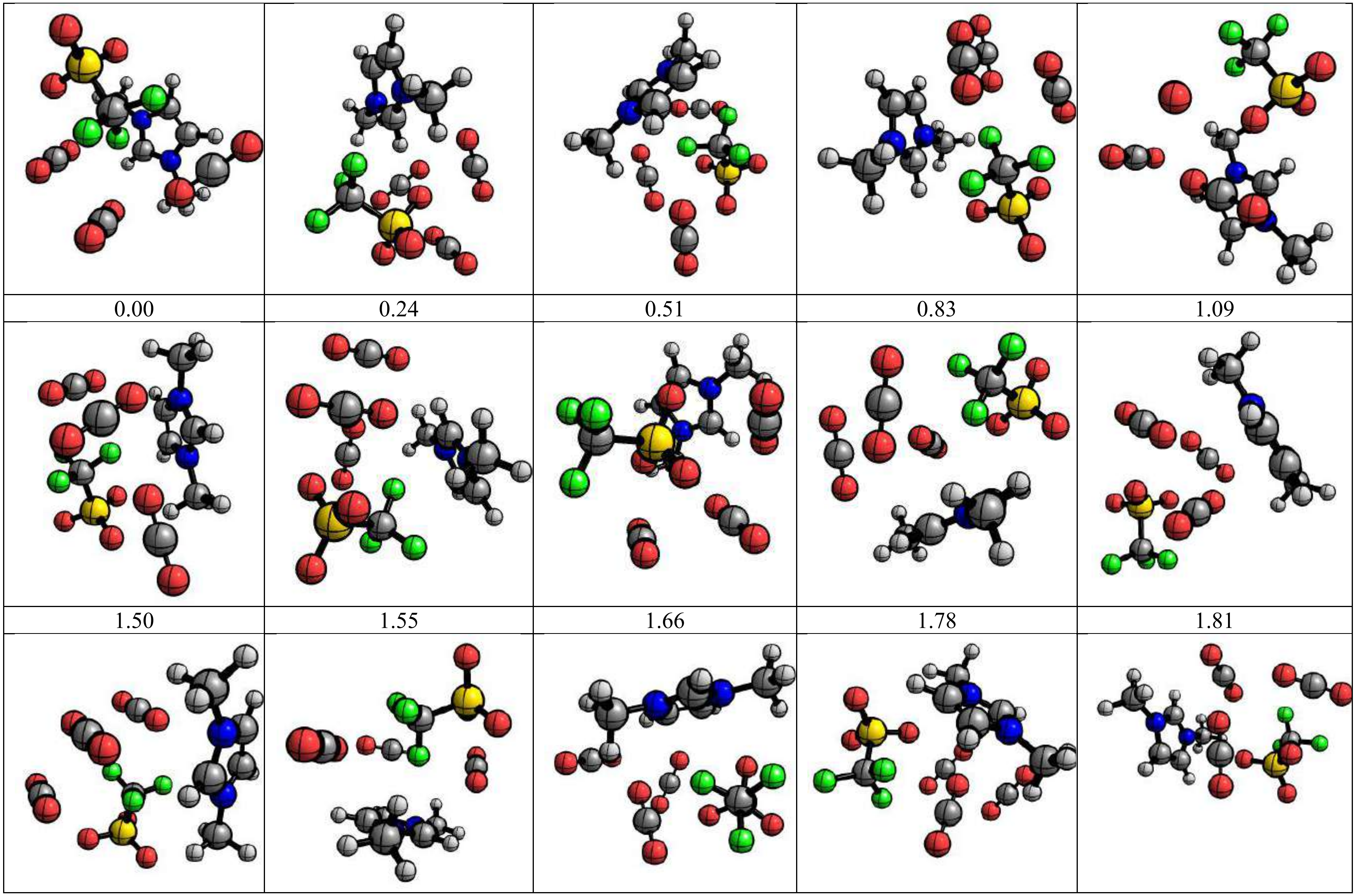
0.00
0.24
0.51
0.83
1.09
1.50
1.55
1.66
1.78
1.81

1.89	1.90	1.92	1.99	2.12
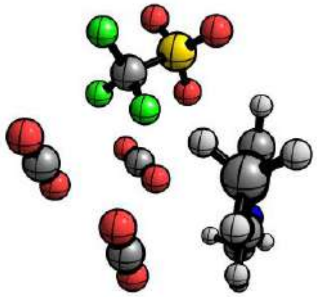	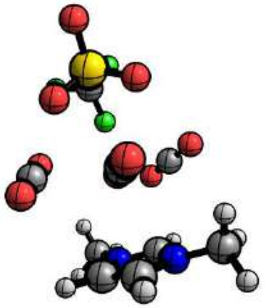	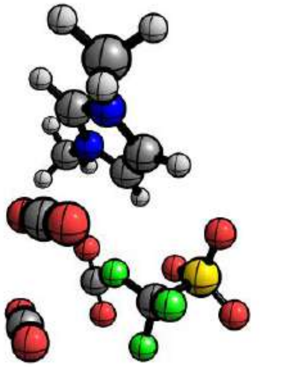	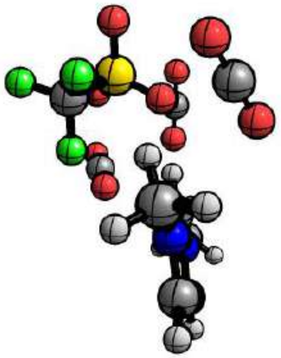	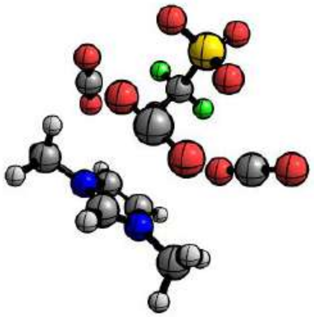
2.12	2.18	2.23	2.80	2.98
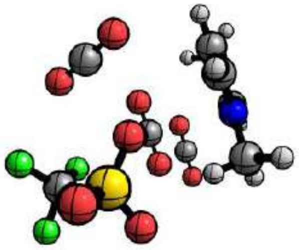	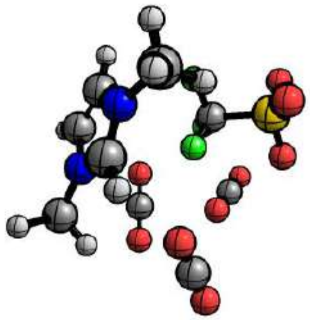	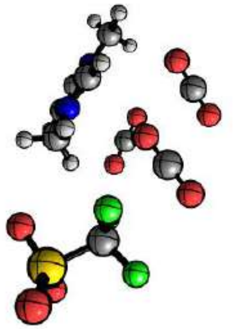	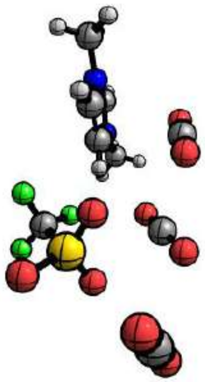	
3.25	3.85	4.06	5.10	

Table S21. Representations of lowest energy forms for $4CO_2[Dmim]^+[TFO]^-$ at the M06-2X-D3/6-31G(d,p) level with an implicit solvent model PCM. Relative energies are listed in kcal/mol.

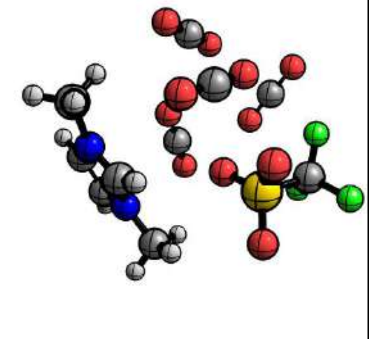	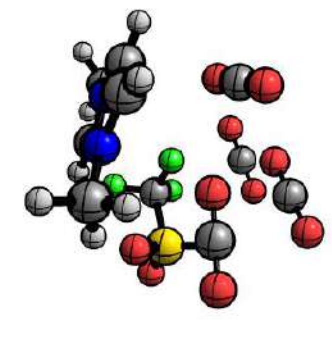	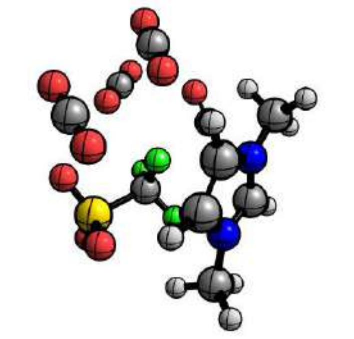	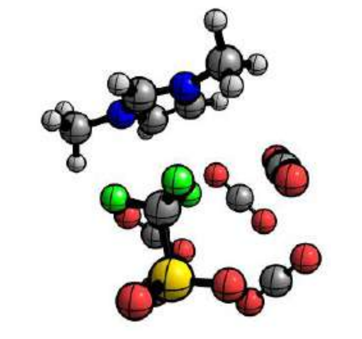	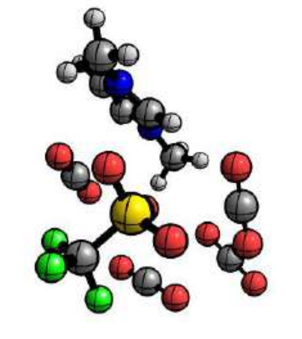
0.00	0.31	0.42	0.64	0.79
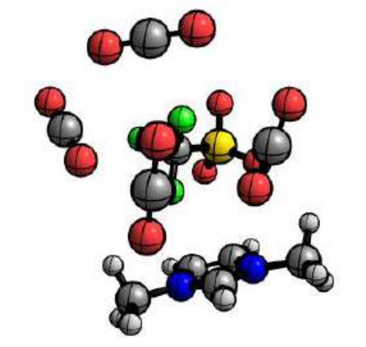	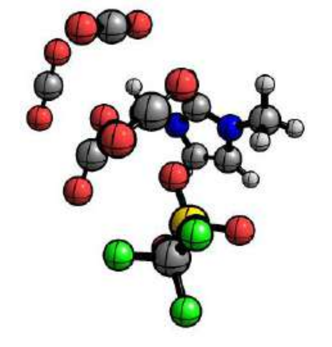	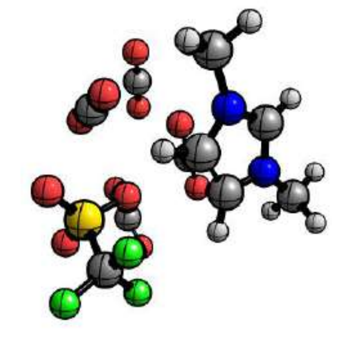	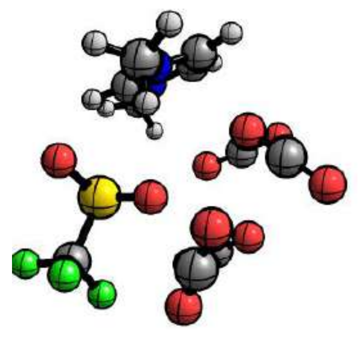	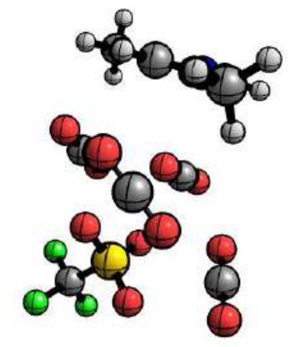
1.50	1.73	1.78	1.94	1.95
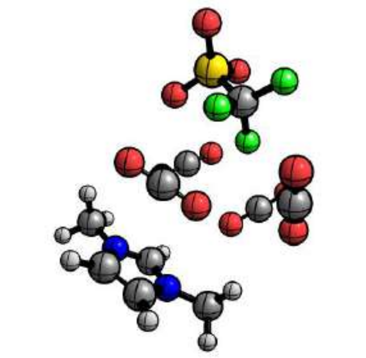	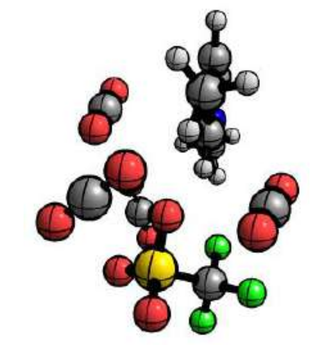	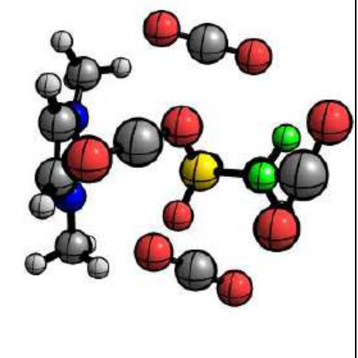	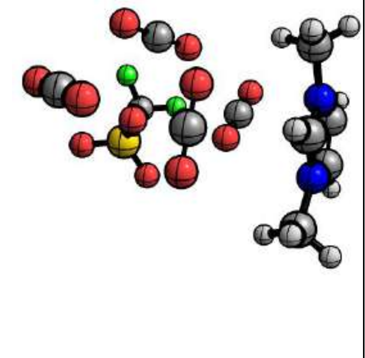	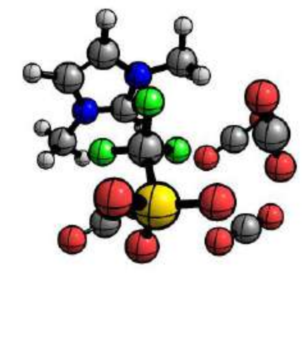
2.20	2.21	2.25	2.26	2.61

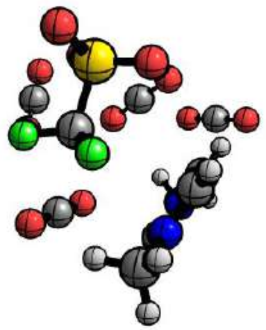	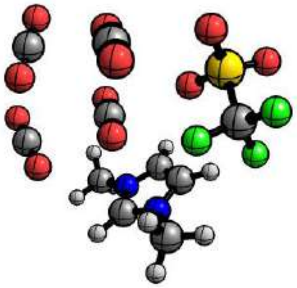	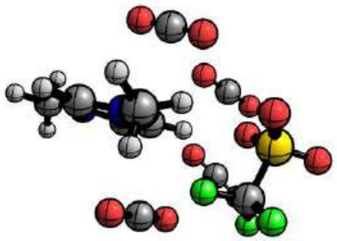	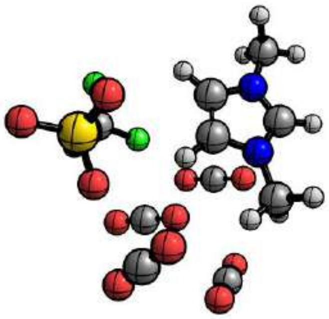	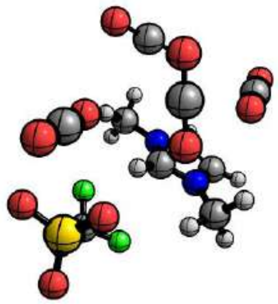
2.93	3.06	3.57	3.60	3.66
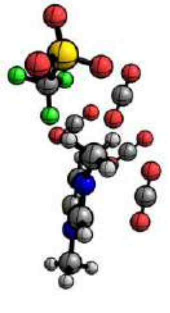	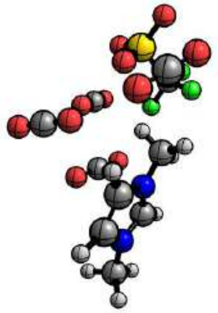	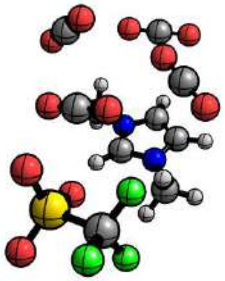	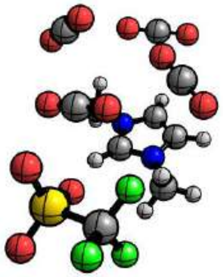	
3.89	4.64	5.24	5.32	

Table S22. Representations of lowest energy forms for $5CO_2[Dmim]^+[TFO]^-$ at the M06-2X-D3/6-31G(d,p) level with an implicit solvent model PCM. Relative energies are listed in kcal/mol.

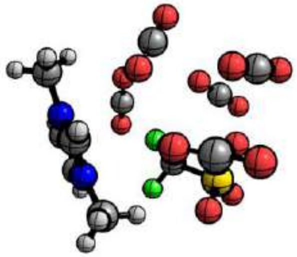	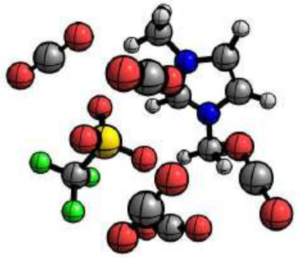	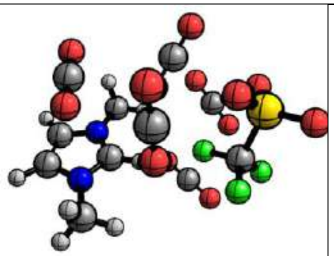	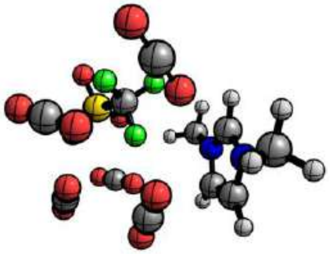	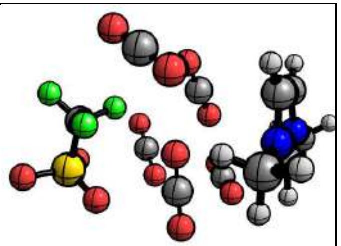
0.00	0.55	0.82	1.52	1.53

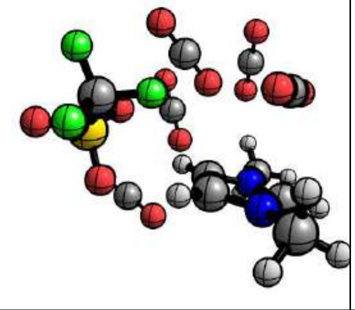	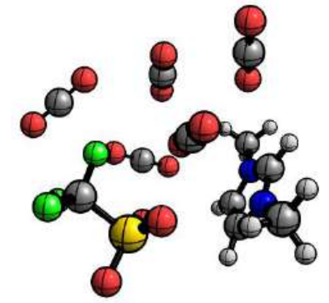	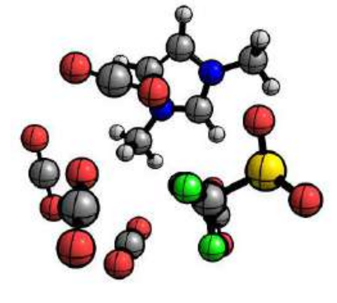	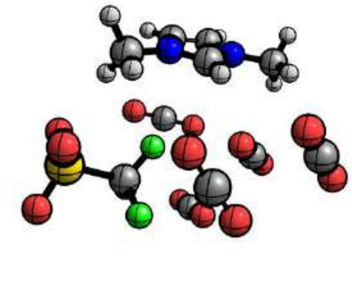	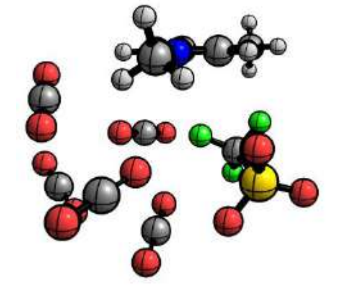
2.09	2.14	2.28	2.78	3.27
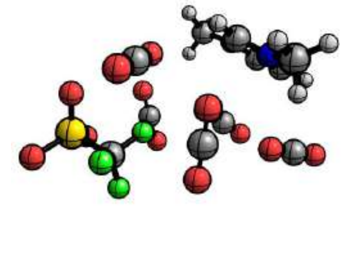	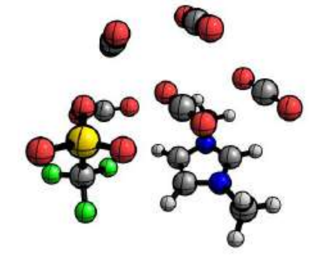	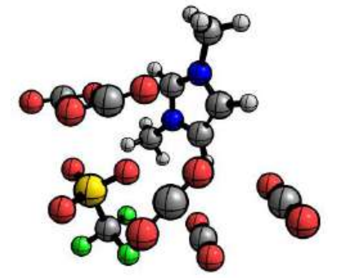	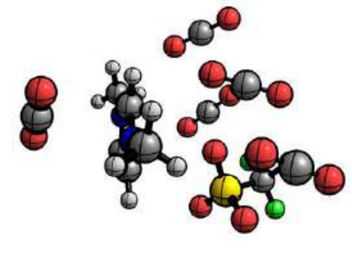	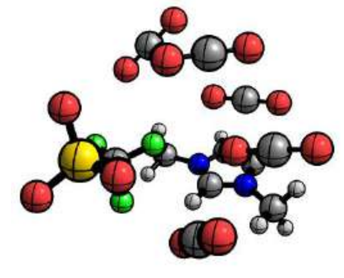
3.32	3.74	3.80	3.94	3.95
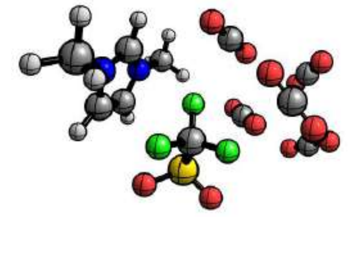	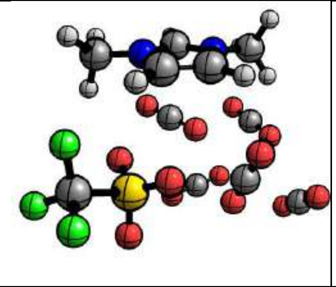	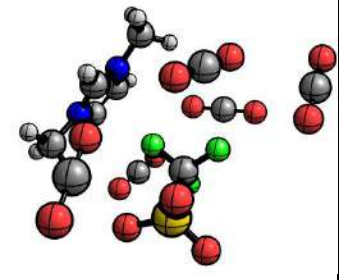	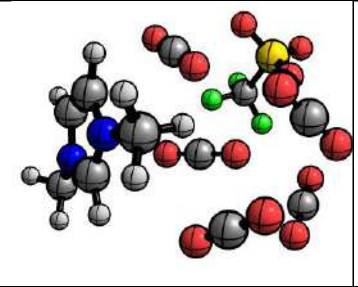	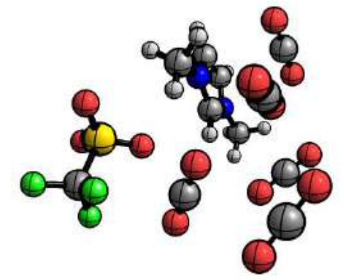
4.07	4.46	4.70	4.84	5.26

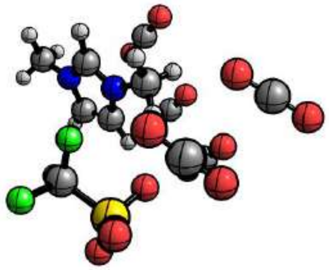	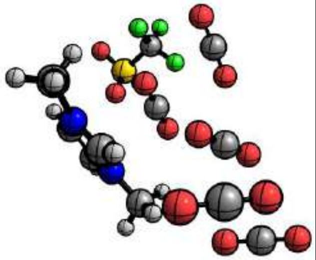	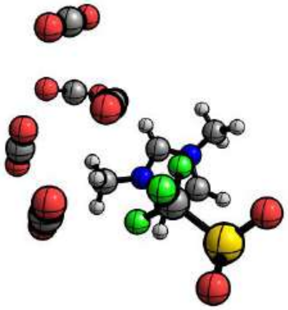		
6.43	7.56	7.62		

Table S23. Representations of lowest energy forms for $1CO_2[Dbim]^+[FAP]^-$ at the M06-2X-D3/6-31G(d,p) level with an implicit solvent model PCM. Relative energies are listed in kcal/mol.

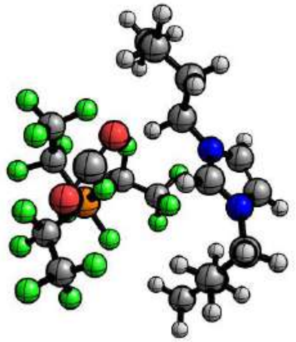	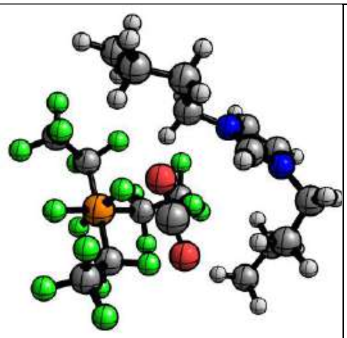	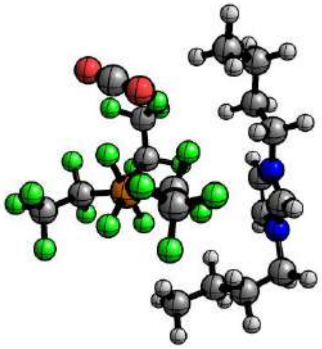	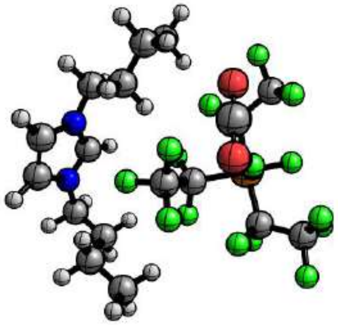	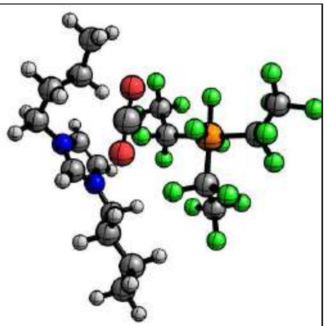
0.00	0.13	0.53	1.07	1.13

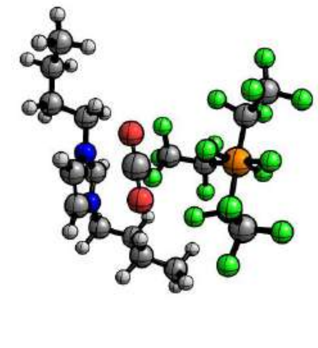	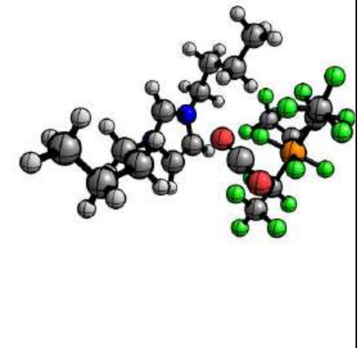	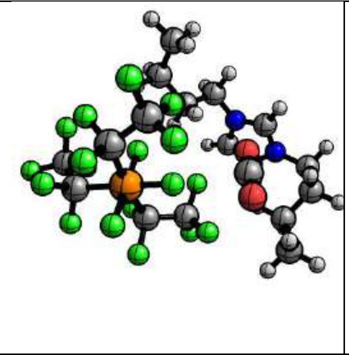	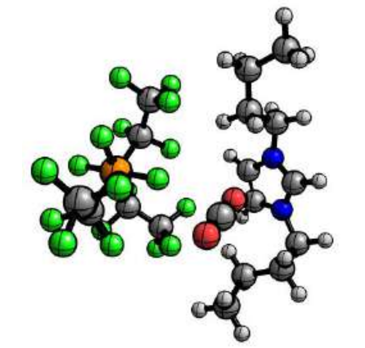	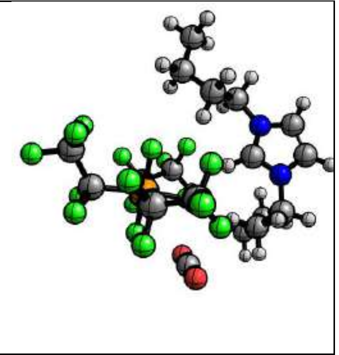
1.41	1.61	1.73	1.82	2.00
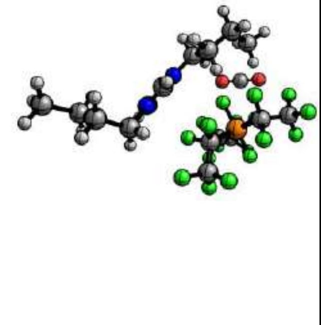	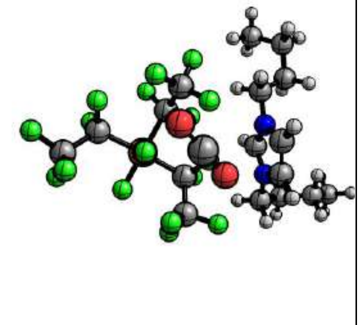	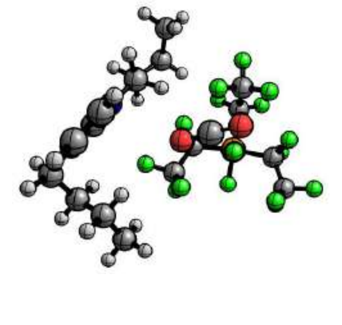	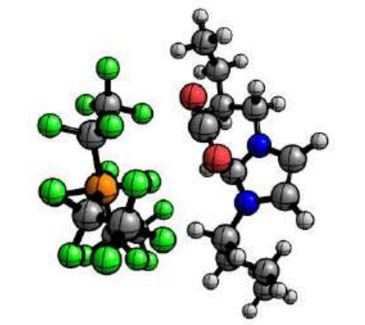	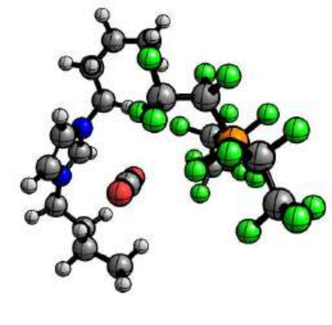
2.13	2.73	3.02	3.20	3.30
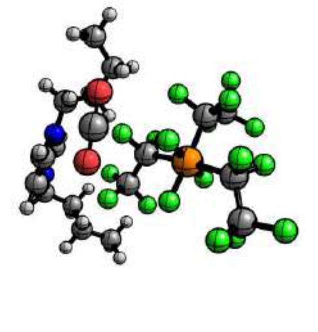	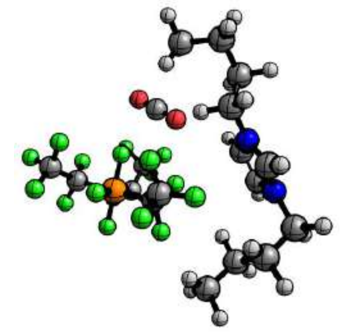	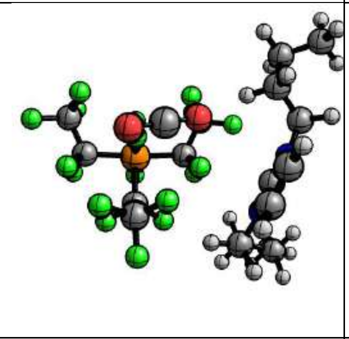	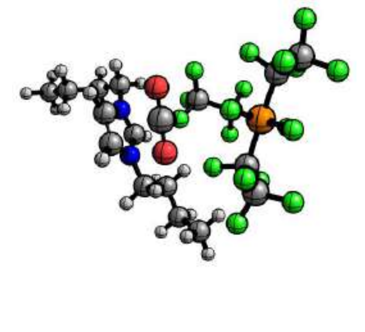	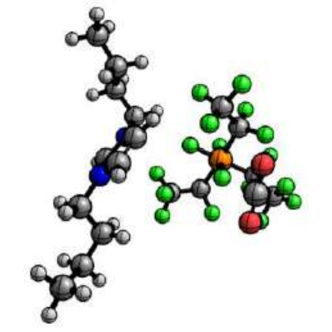
3.35	3.69	3.83	3.88	4.69

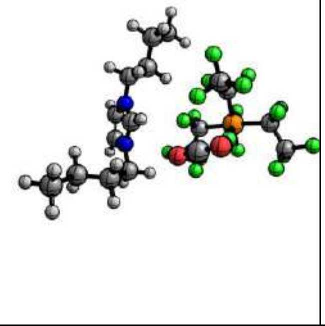	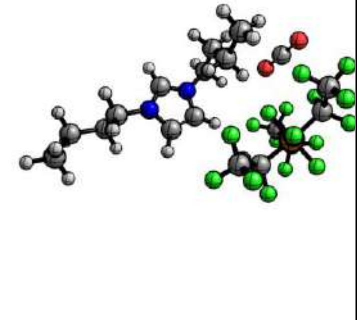	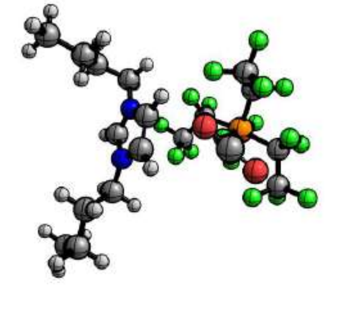	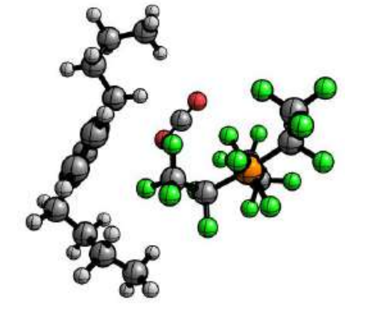	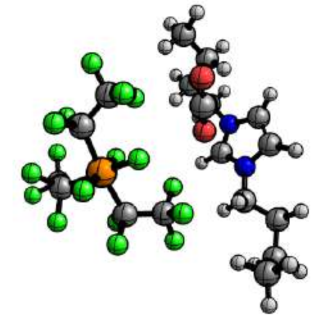
5.01	5.14	5.32	5.45	5.96
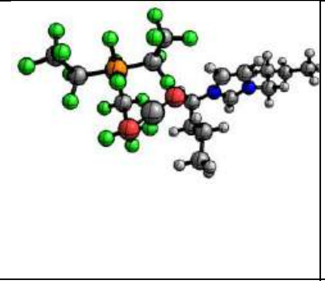	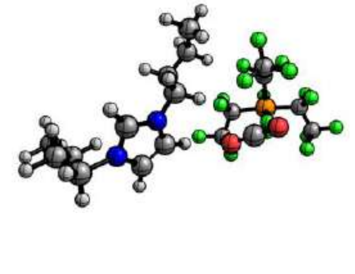	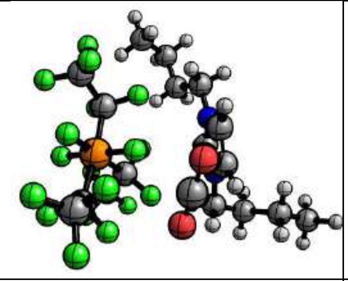	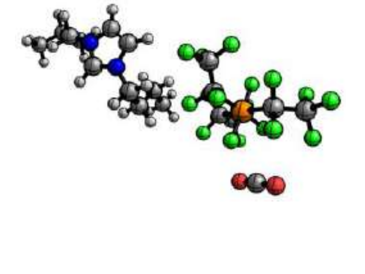	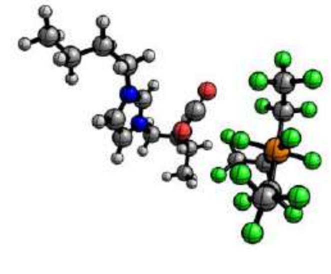
6.30	6.41	6.44	6.96	7.90
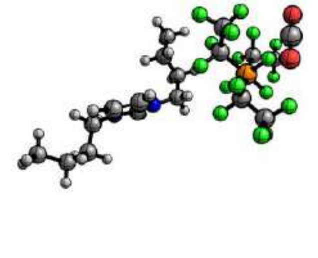	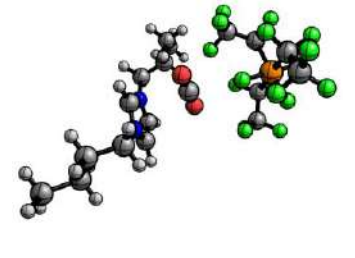	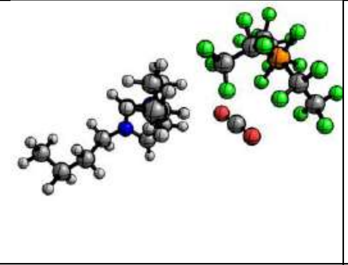	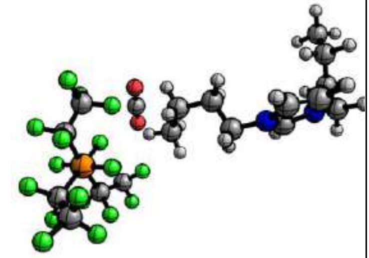	
8.04	8.58	9.41	12.70	

Table S24. Representations of lowest energy forms for $2CO_2[Dbim]^+[FAP]^-$ at the M06-2X-D3/6-31G(d,p) level with an implicit solvent model PCM. Relative energies are listed in kcal/mol.

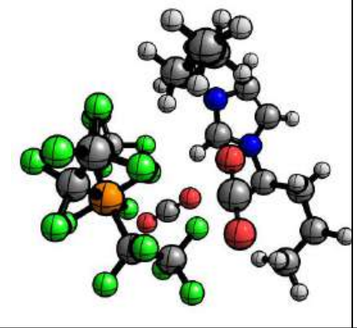	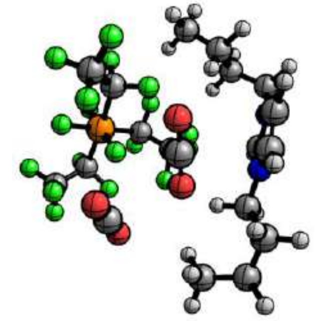	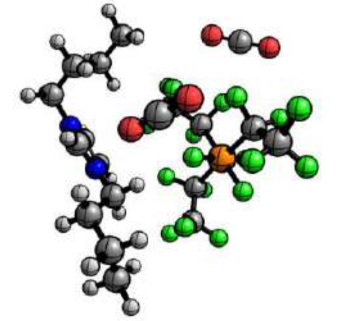	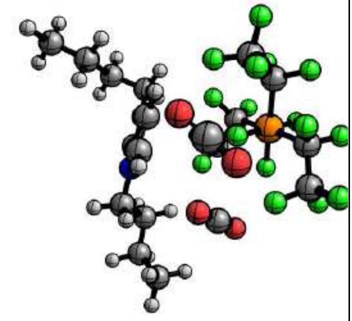	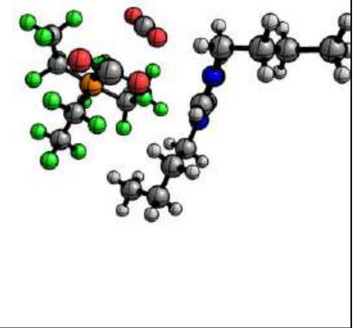
0.00	0.69	2.90	2.91	3.60
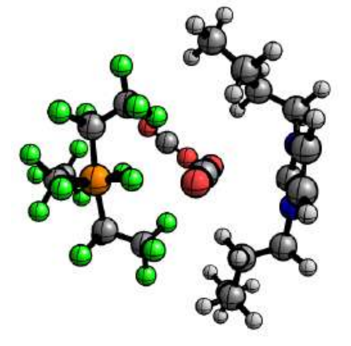	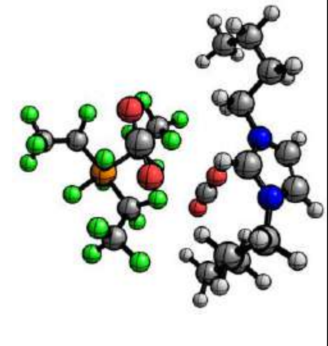	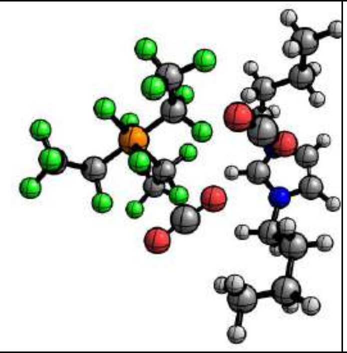	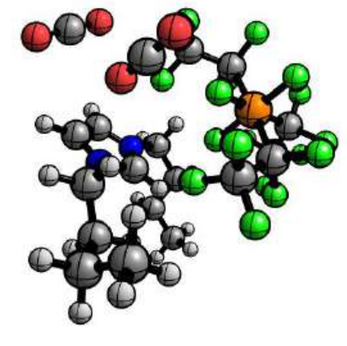	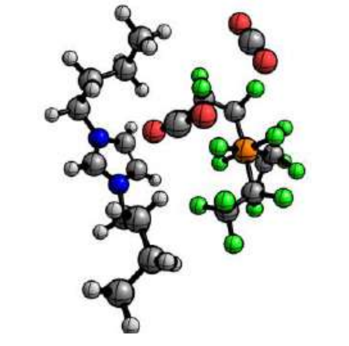
3.66	3.91	4.37	4.87	4.88
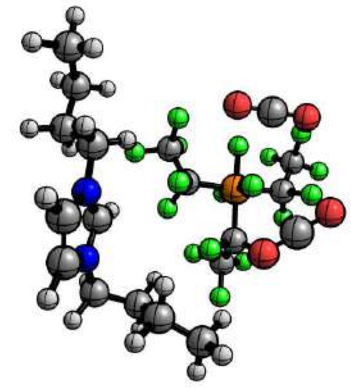	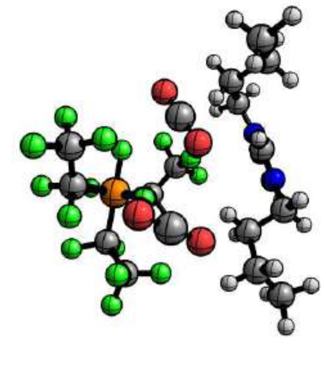	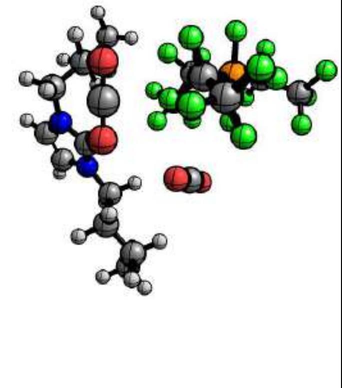	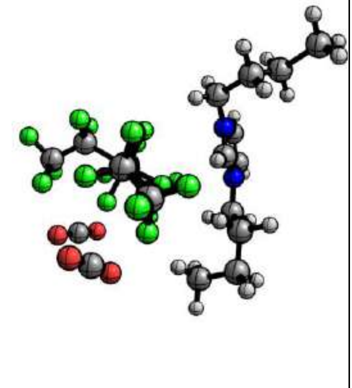	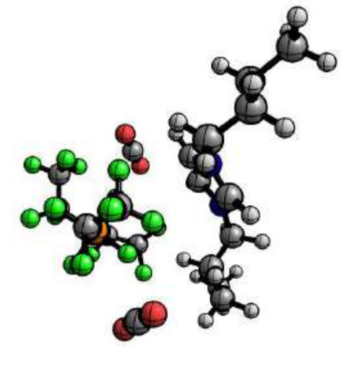

5.05	5.13	5.23	5.24	5.25
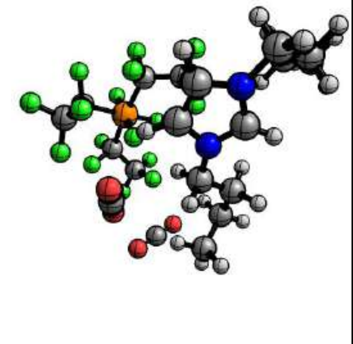	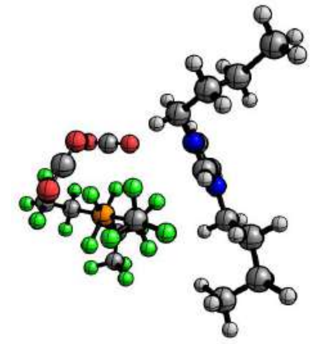	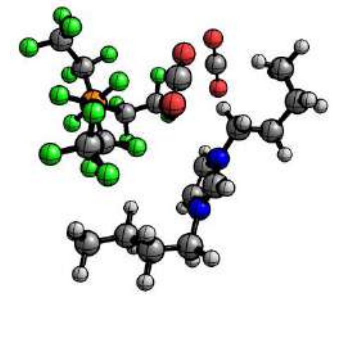	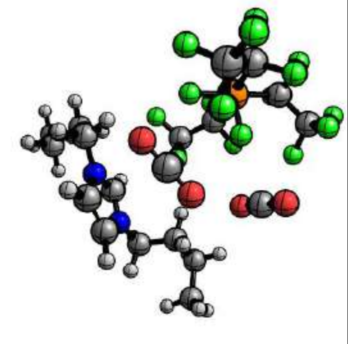	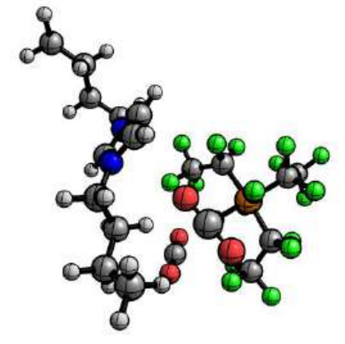
5.35	5.46	5.71	6.22	6.30
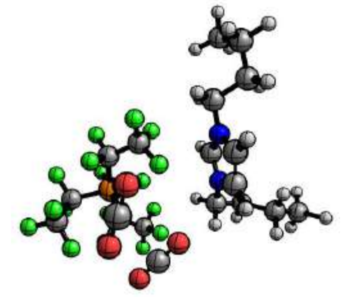	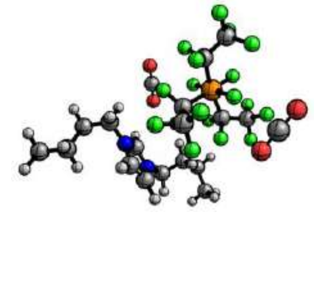	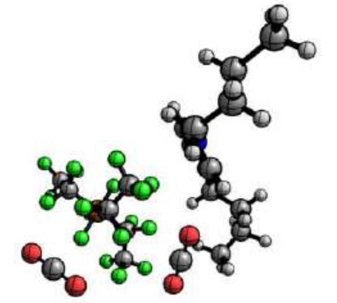	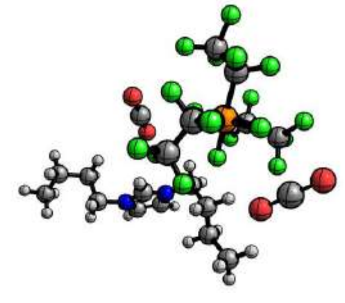	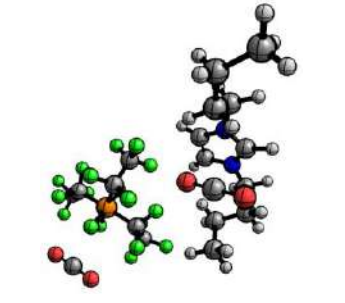
6.36	7.27	7.81	8.37	8.41
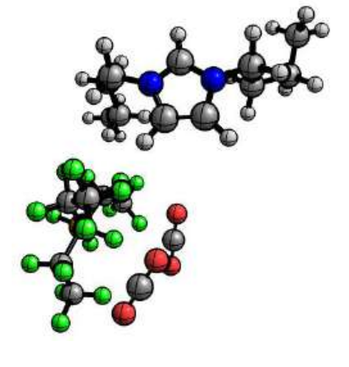	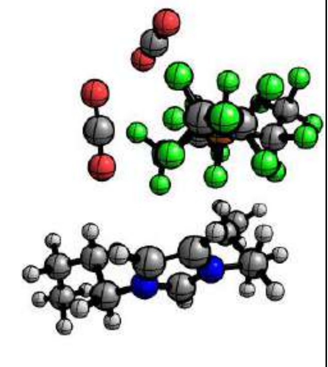	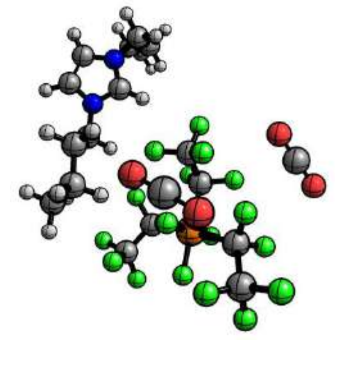	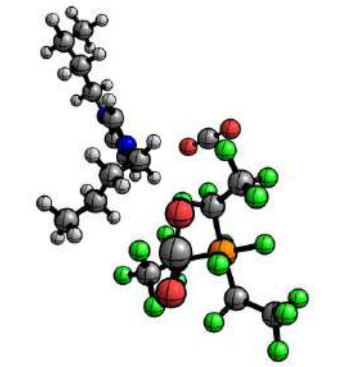	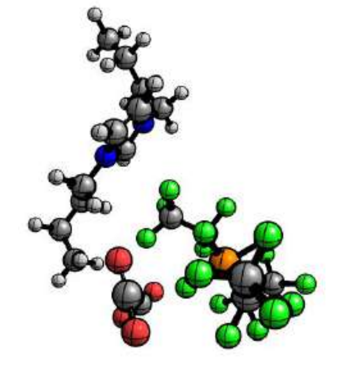
8.59	8.72	9.14	10.79	11.40

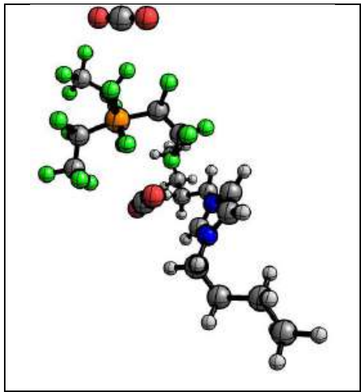	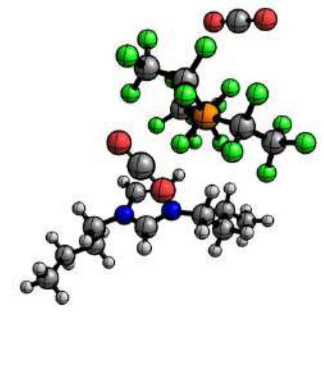	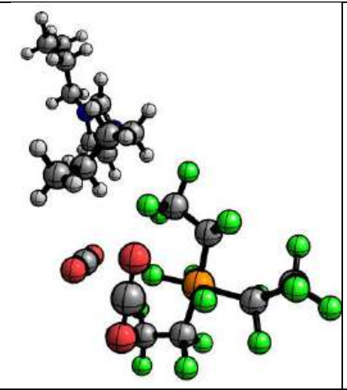	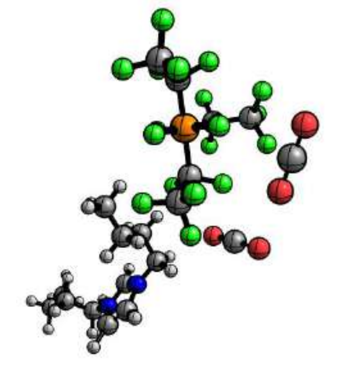	
11.40	11.53	12.79	13.31	

Table S25. Representations of lowest energy forms for $3CO_2[Dbim]^+[FAP]^-$ at the M06-2X-D3/6-31G(d,p) level with an implicit solvent model PCM. Relative energies are listed in kcal/mol.

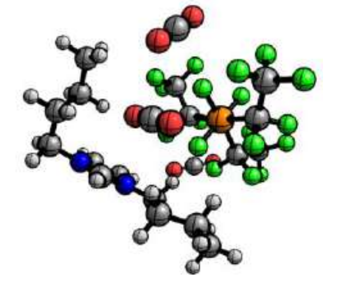	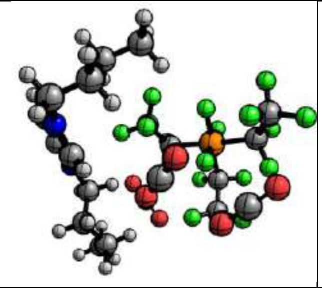	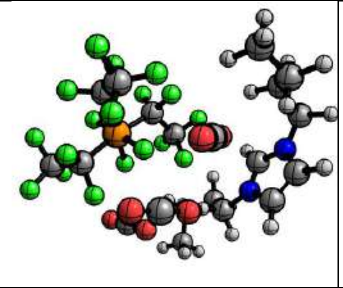	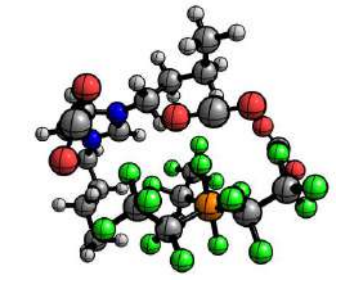	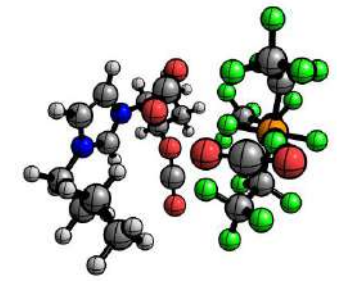
0.00	0.26	0.80	0.85	1.31

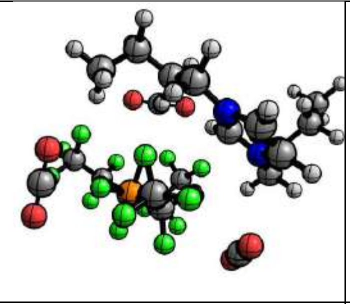	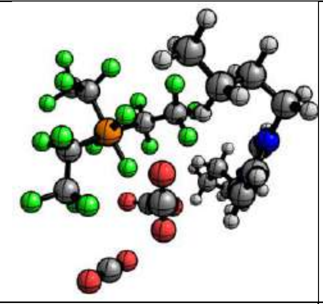	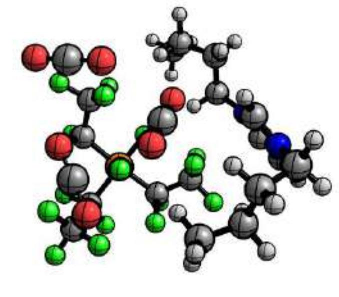	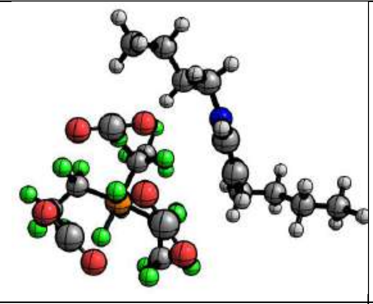	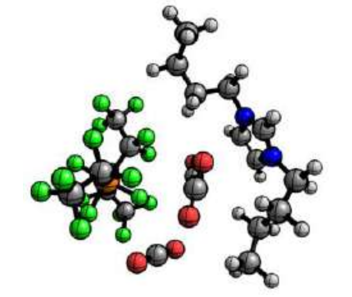
1.55	1.56	1.65	1.66	2.67
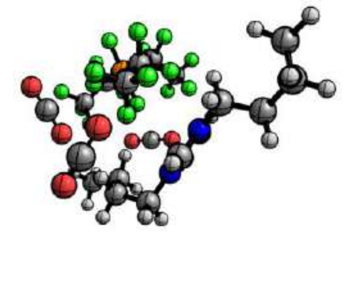	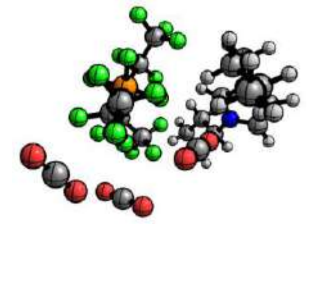	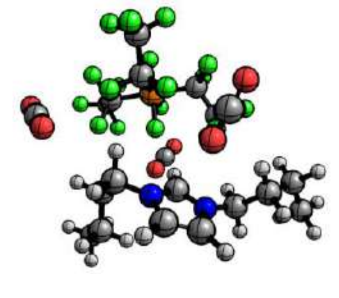	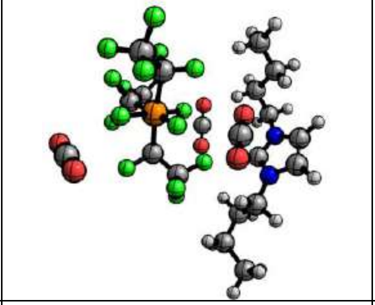	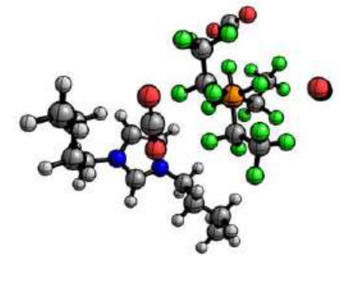
2.93	3.16	3.65	3.84	3.87
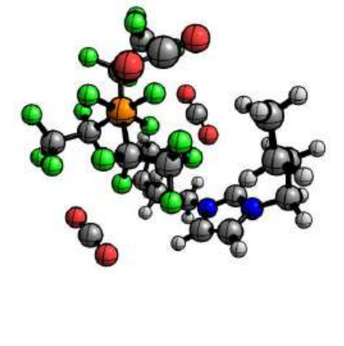	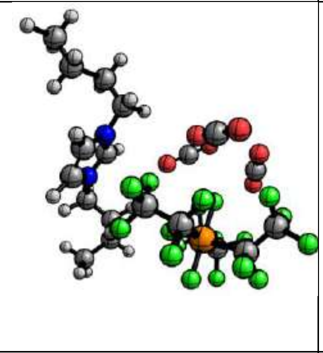	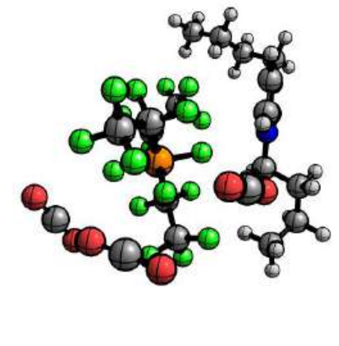	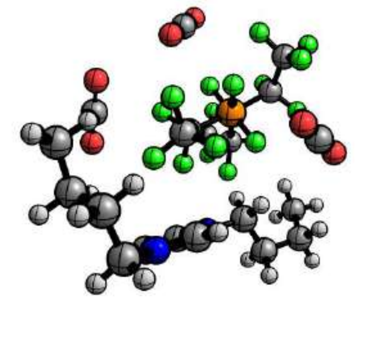	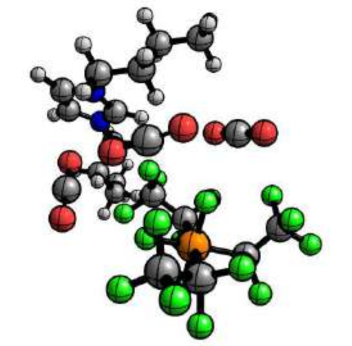
4.98	5.07	5.19	5.33	5.43

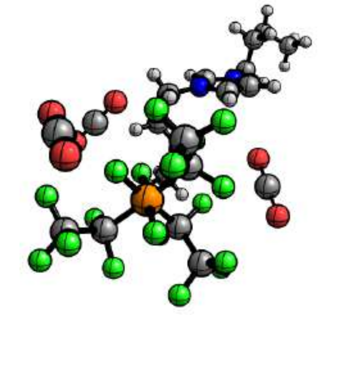	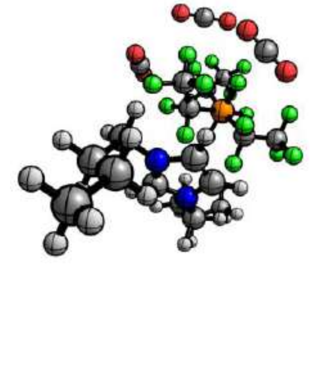	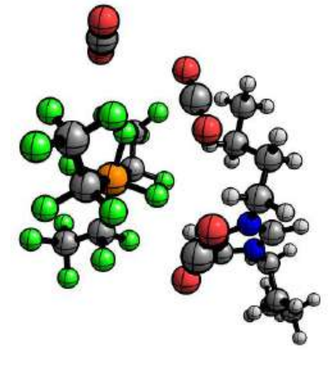	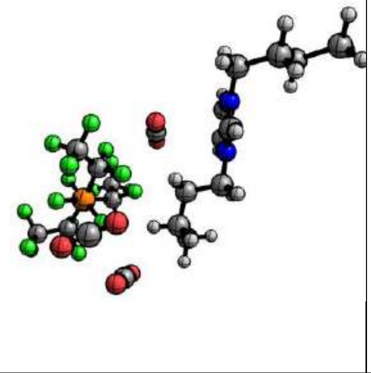	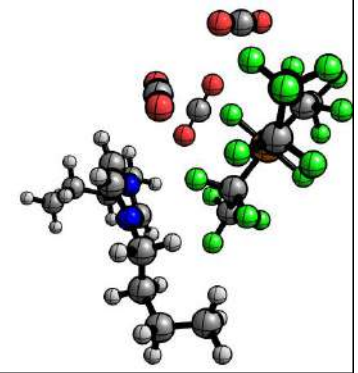
5.53	5.54	5.73	5.90	6.04
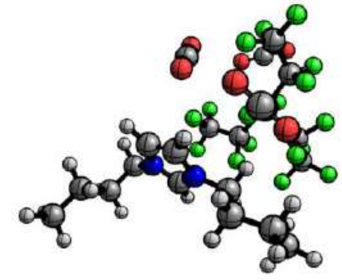	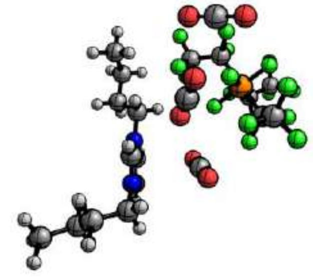	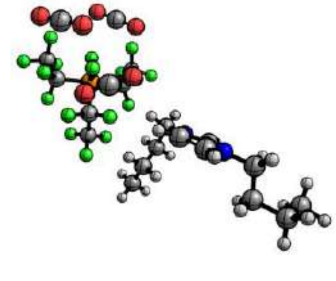	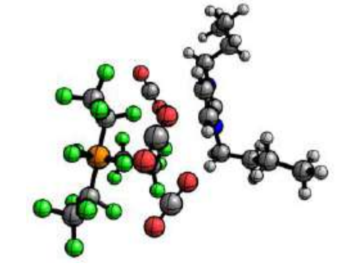	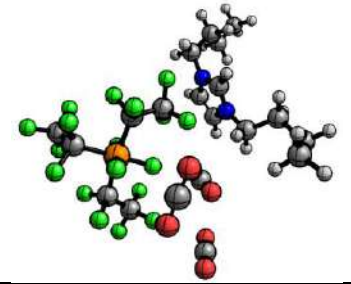
6.10	6.17	6.63	6.64	8.38
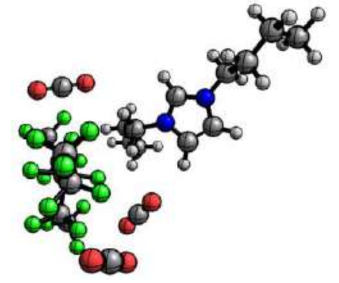	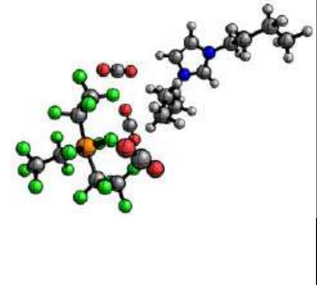	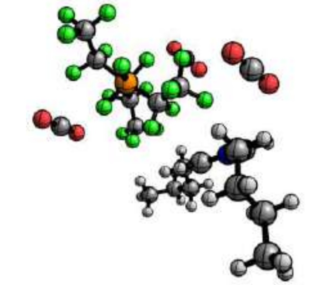	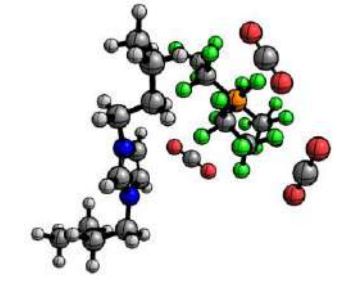	
9.96	11.11	13.51	14.68	

Table S26. Representations of lowest energy forms for $4CO_2[Dbim]^+[FAP]^-$ at the M06-2X-D3/6-31G(d,p) level with an implicit solvent model PCM. Relative energies are listed in kcal/mol.

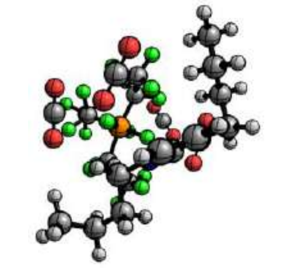	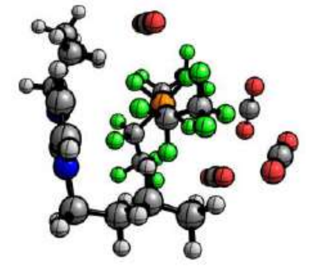	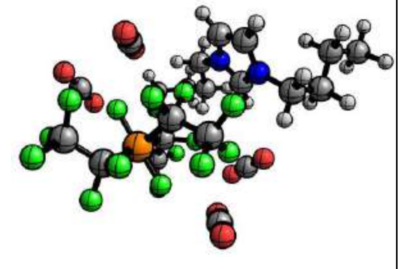	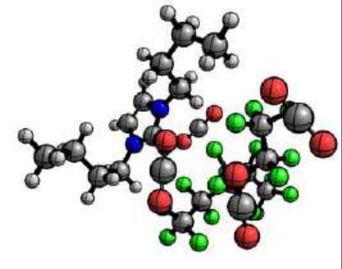	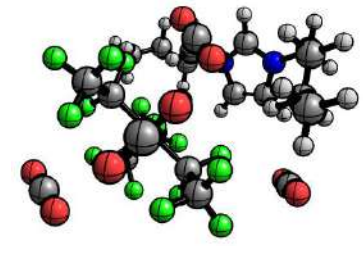
0.00	1.42	1.46	1.59	2.22
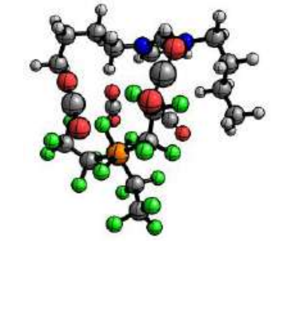	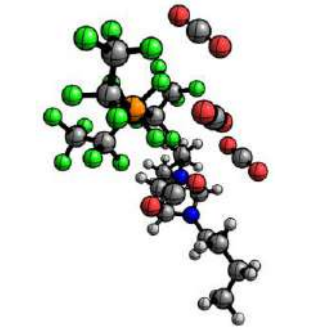	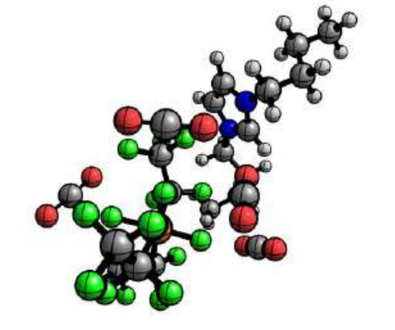	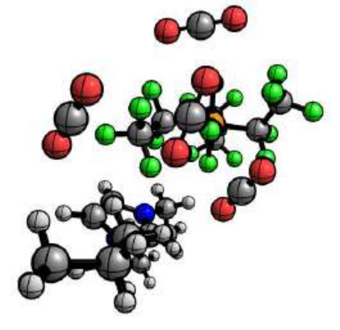	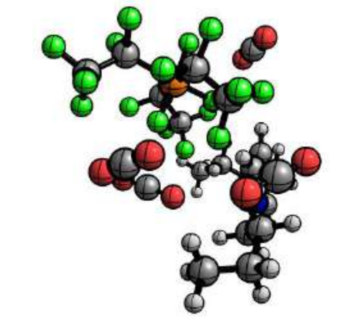
3.87	4.56	4.59	4.90	4.93
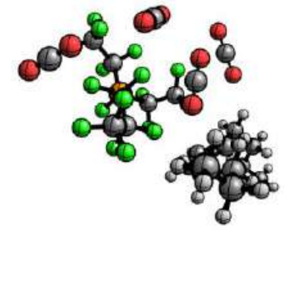	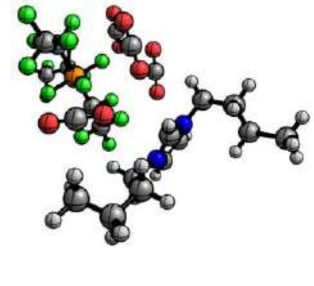	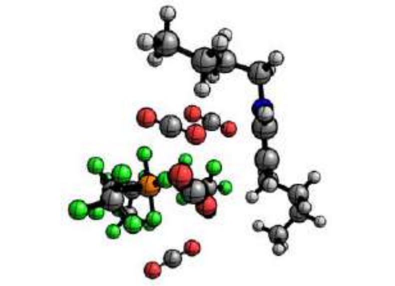	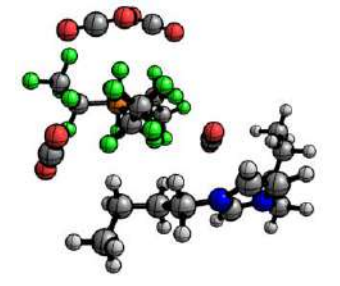	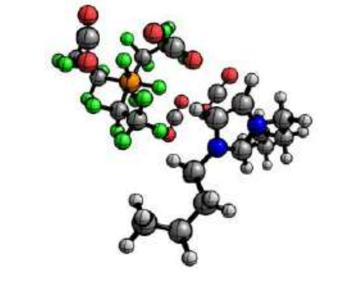
5.19	5.49	5.59	6.10	7.22

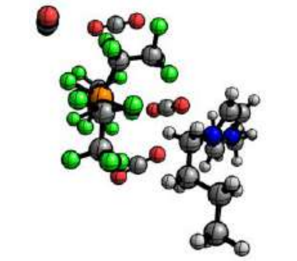	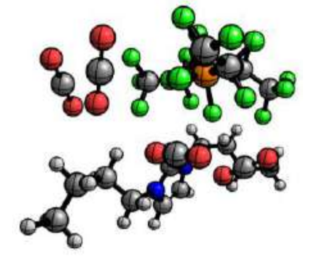	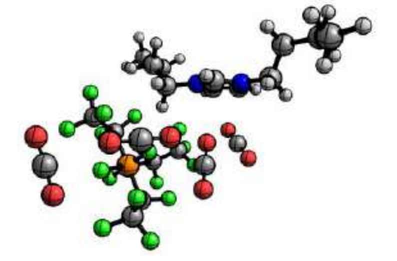	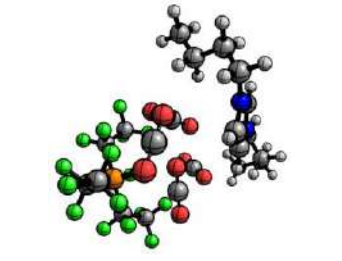	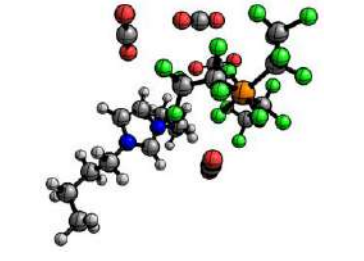
7.32	7.47	7.59	7.81	7.90
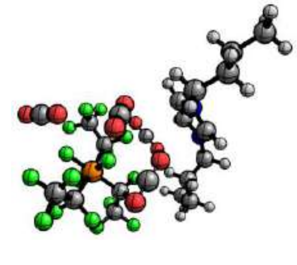	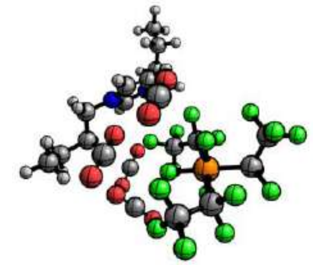	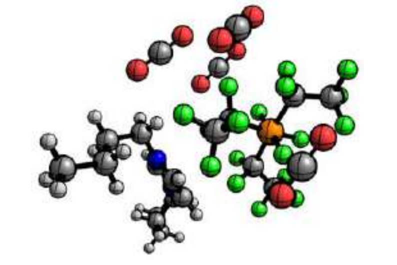	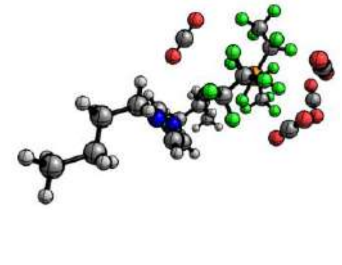	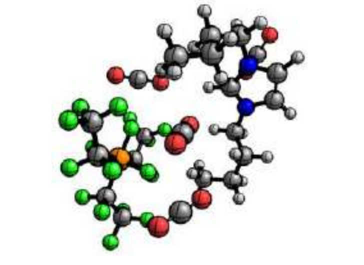
8.12	8.46	9.15	9.40	9.89
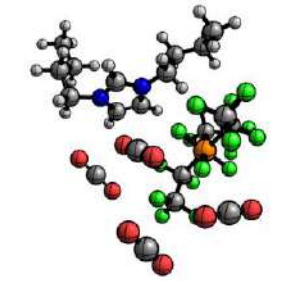	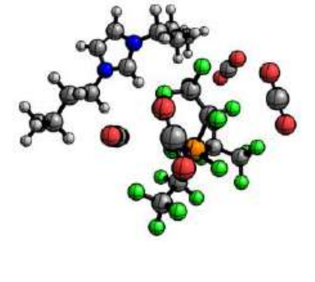	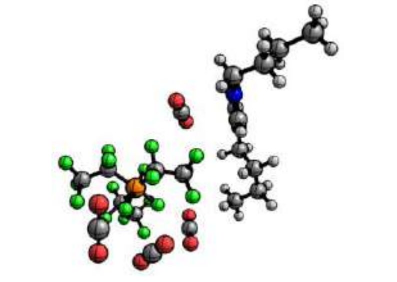	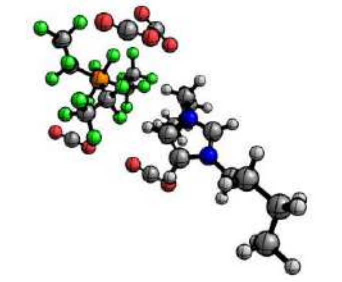	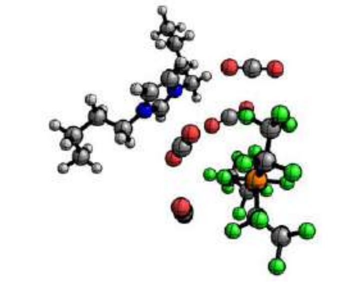
9.99	10.23	10.93	12.45	13.22

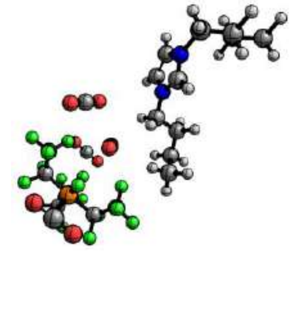	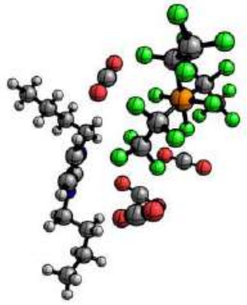	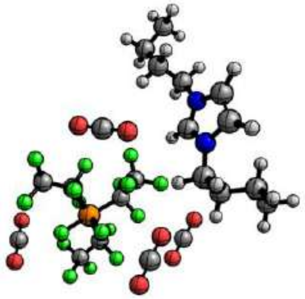	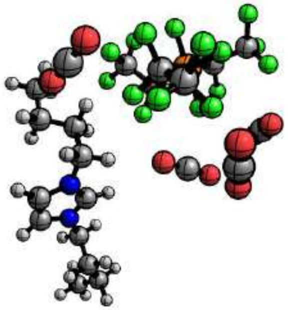	
13.55	13.66	16.62	16.63	

Table S27. Representations of lowest energy forms for $5CO_2[Dbim]^+[FAP]^-$ at the M06-2X-D3/6-31G(d,p) level with an implicit solvent model PCM. Relative energies are listed in kcal/mol.

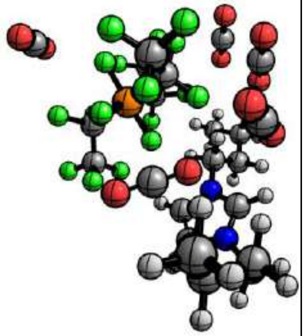	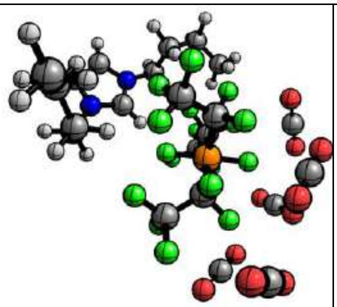	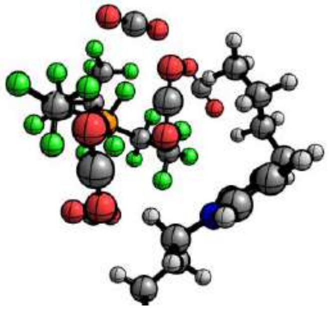	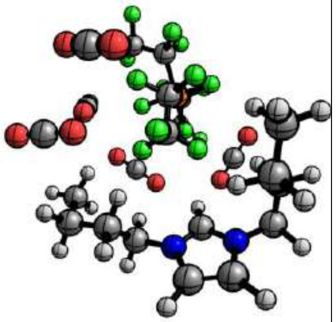	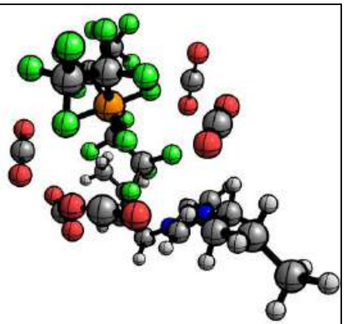
0.00	2.50	5.83	6.33	6.40

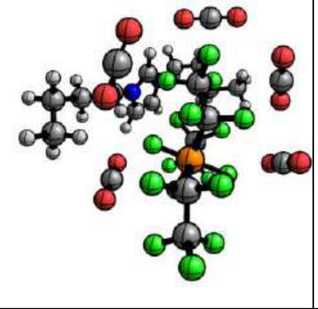	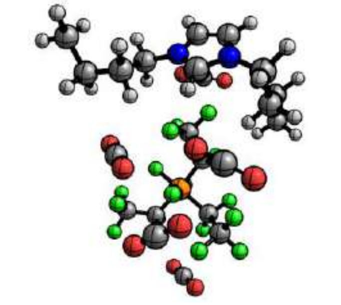	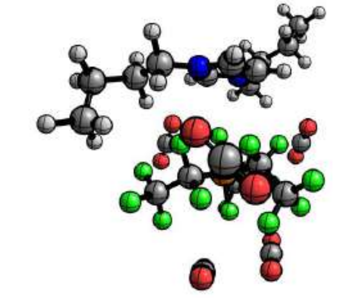	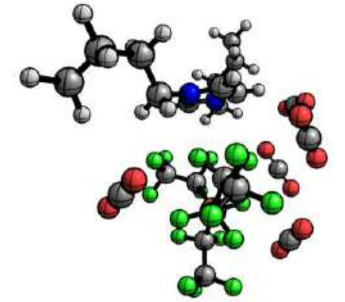	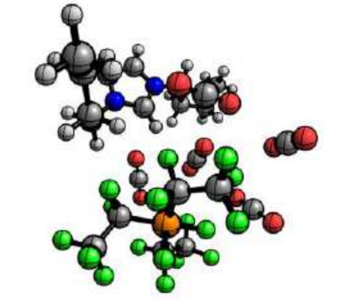
6.47	6.68	6.92	7.28	7.40
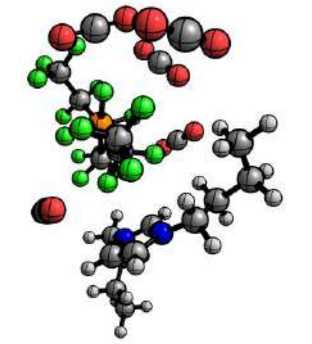	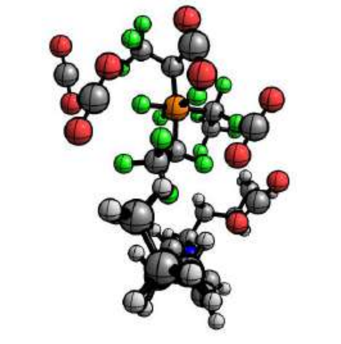	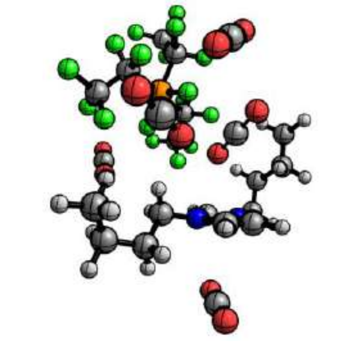	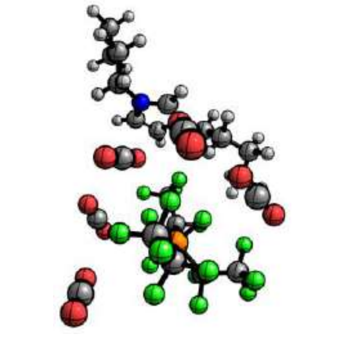	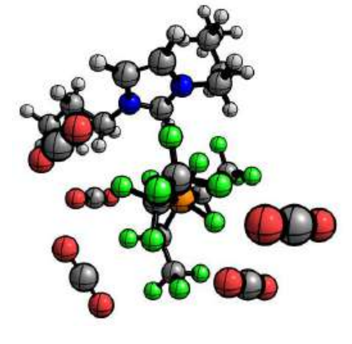
7.97	8.03	8.22	8.24	8.88
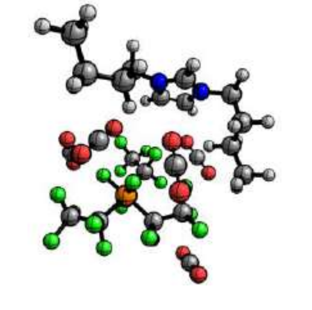	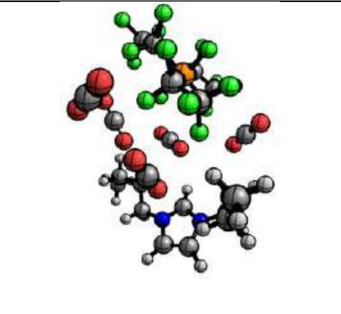	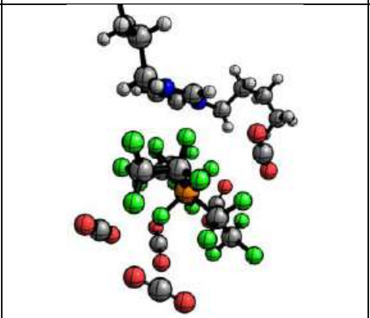	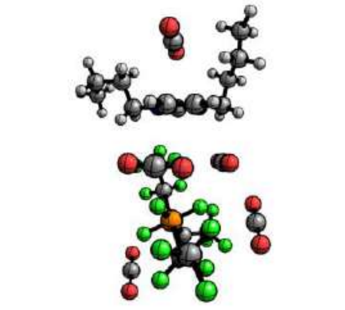	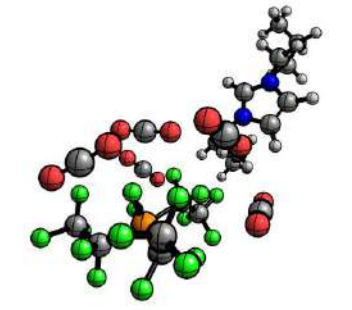
8.98	9.16	9.25	9.38	9.41

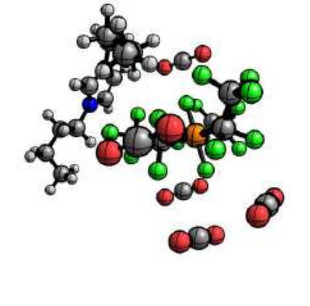	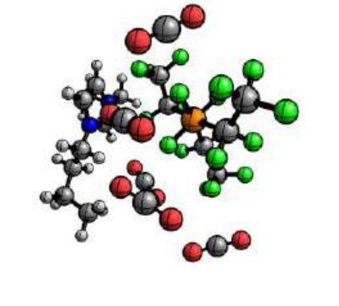	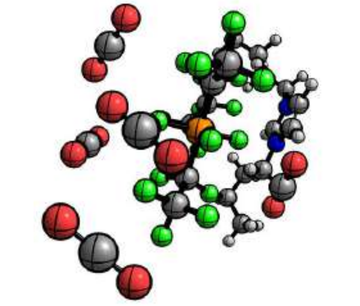	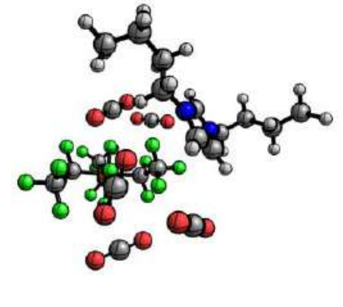	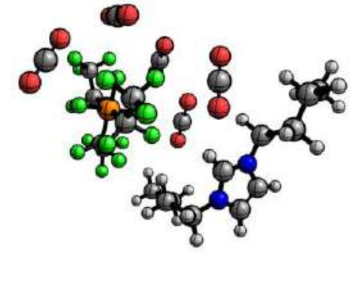
9.62	10.19	10.29	10.57	10.86
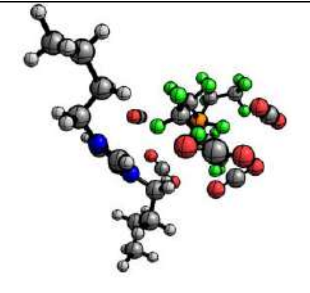	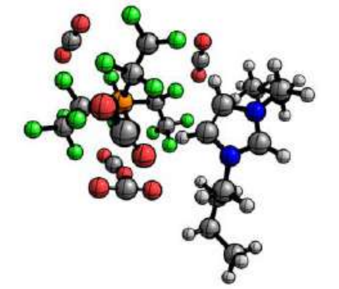	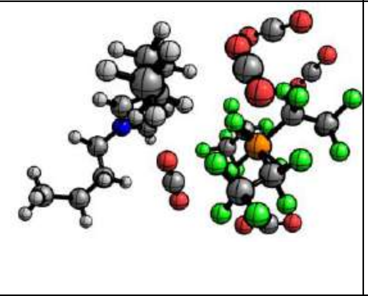	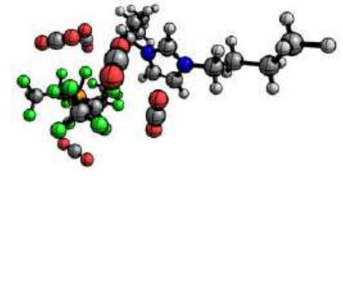	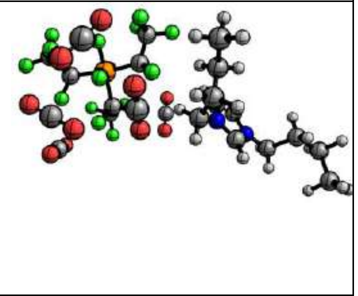
10.99	11.48	11.93	12.25	12.62
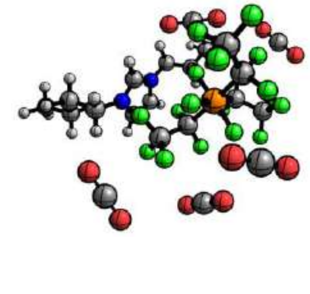	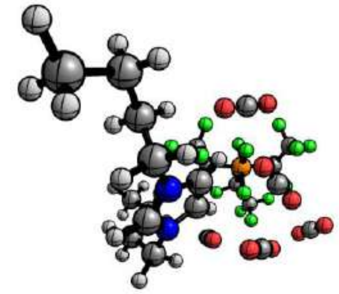	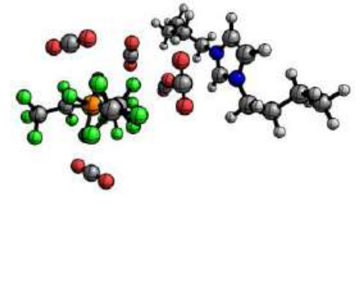	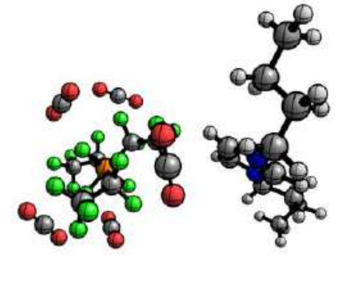	
13.19	14.82	16.12	21.00	

Table S28. Representations of lowest energy forms for $1CO_2[Dbim]^+[Methide]^-$ at the M06-2X-D3/6-31G(d,p) level with an implicit solvent model PCM. Relative energies are listed in kcal/mol.

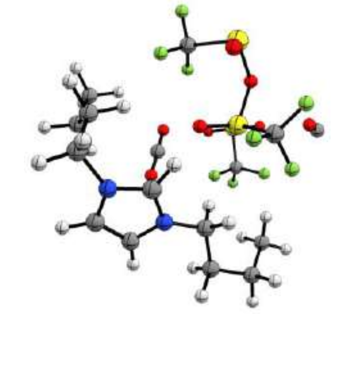	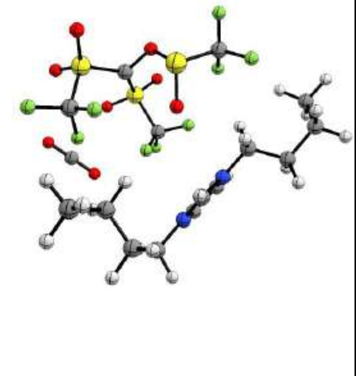	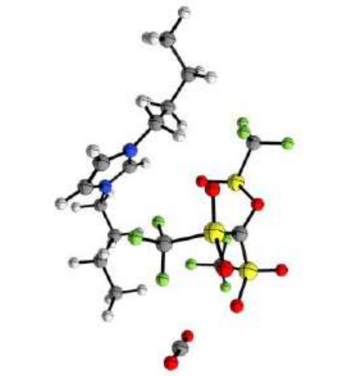	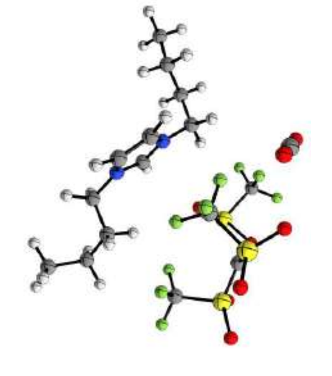	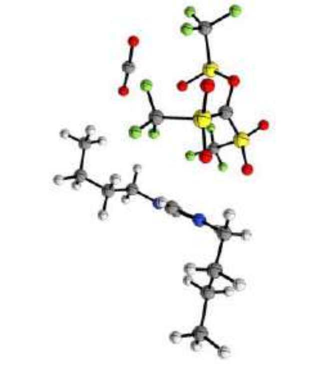
0.00	26.07	26.60	27.71	27.82
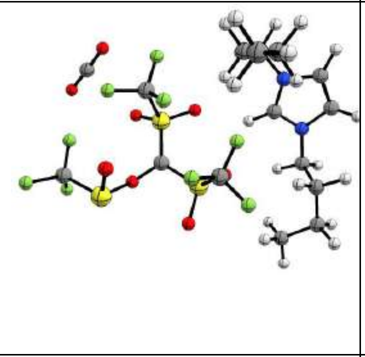	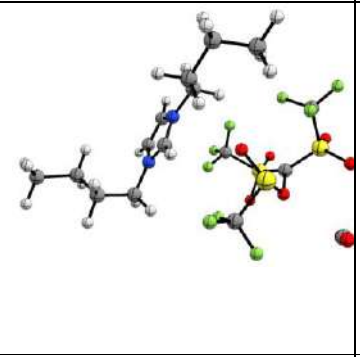	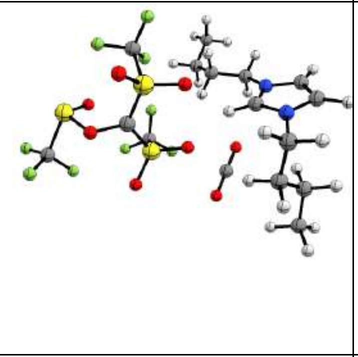	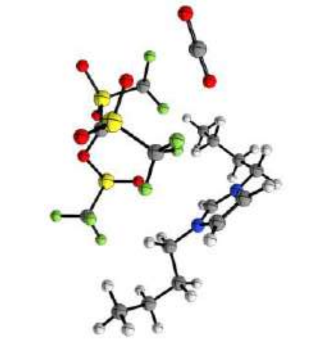	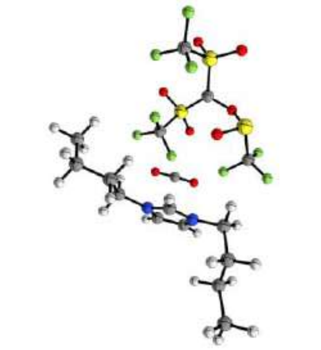
28.08	28.48	28.66	28.89	28.95

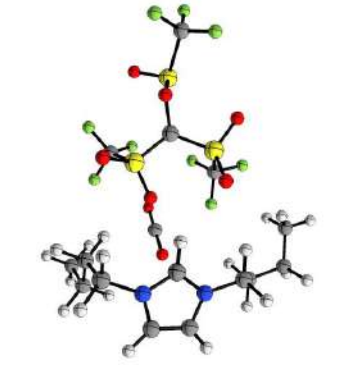	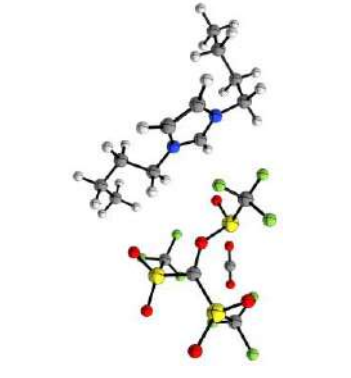	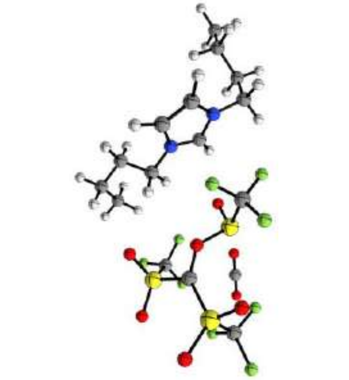	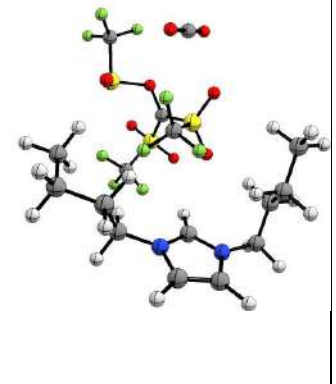	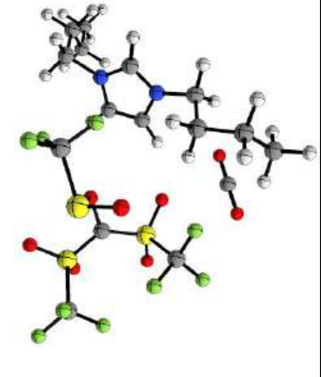
30.04	30.45	30.46	30.49	30.91
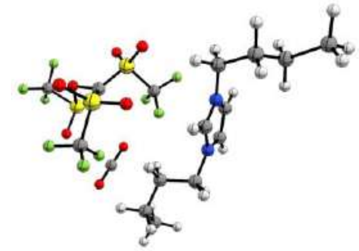	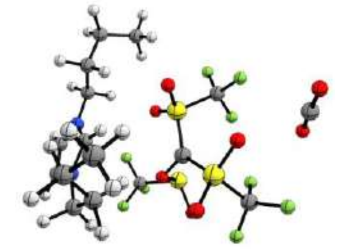	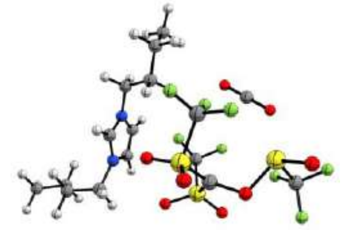	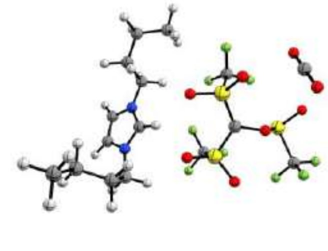	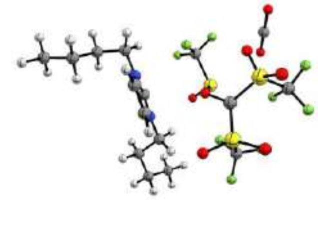
30.95	31.00	31.10	31.29	31.43
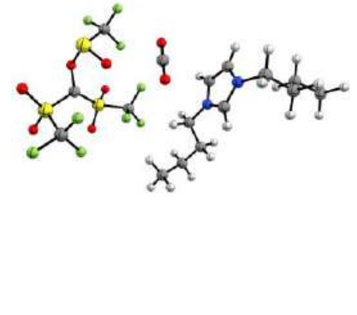	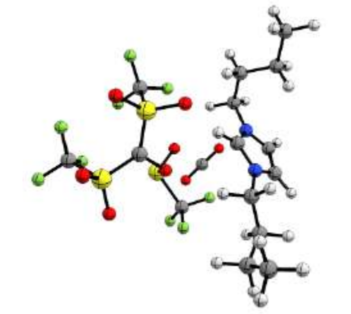	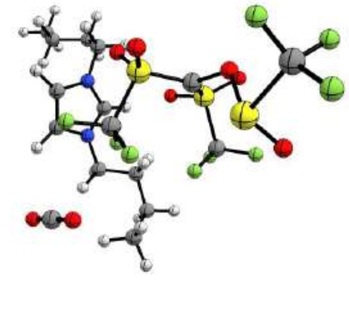	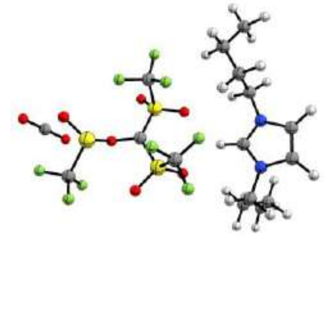	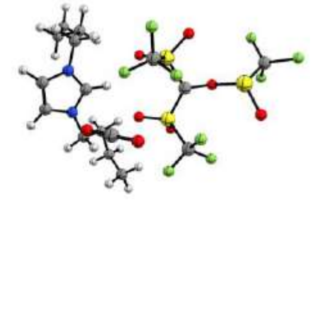
31.49	31.51	31.78	31.87	32.45

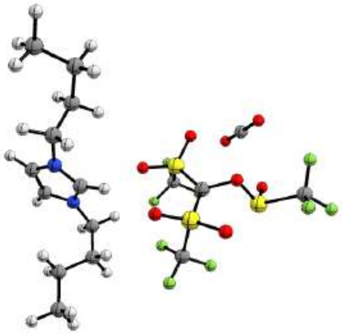	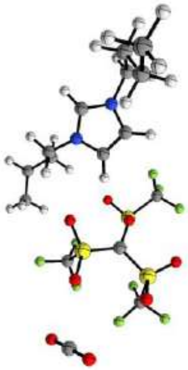	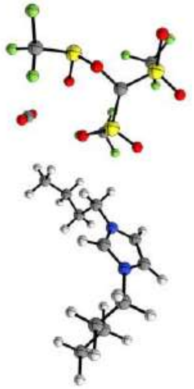		
32.97	33.74	35.42		

Table S29. Representations of lowest energy forms for $2CO_2[Dbim]^+[Methide]^-$ at the M06-2X-D3/6-31G(d,p) level with an implicit solvent model PCM. Relative energies are listed in kcal/mol.

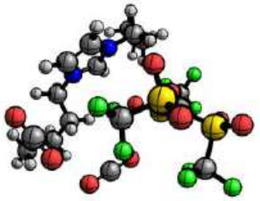	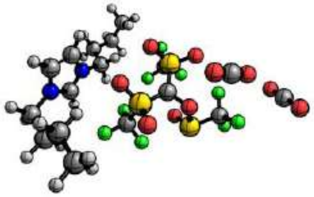	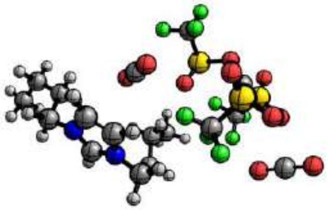	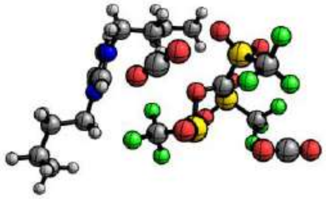	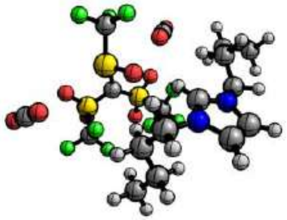
0.00	0.75	2.75	3.25	3.73
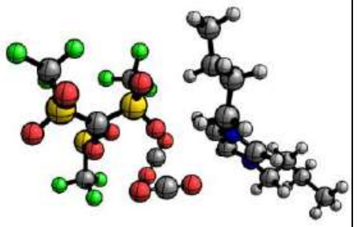	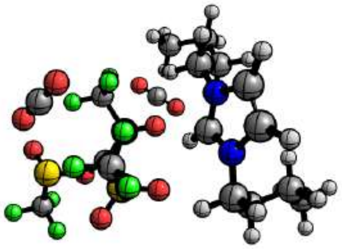	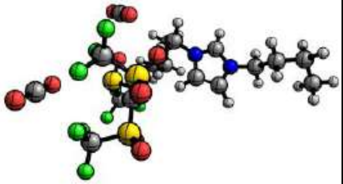	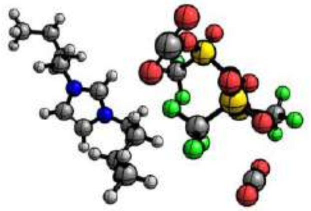	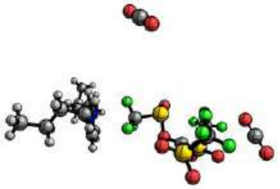
4.15	4.57	4.96	5.38	5.77

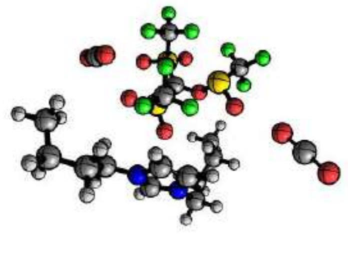	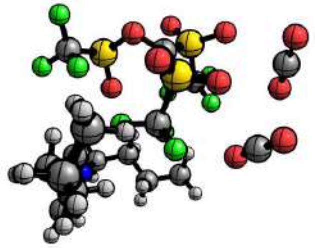	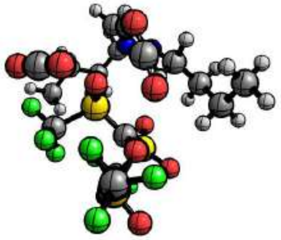	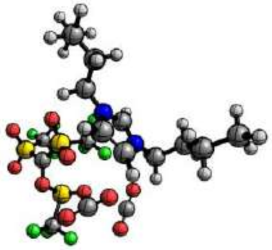	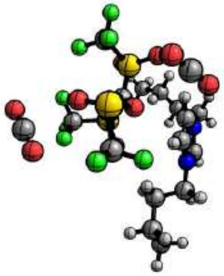
5.79	5.89	6.41	6.44	6.86
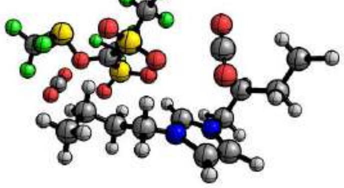	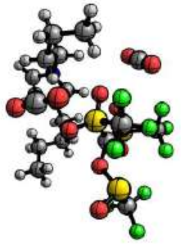	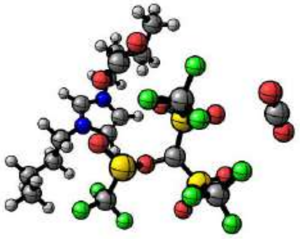	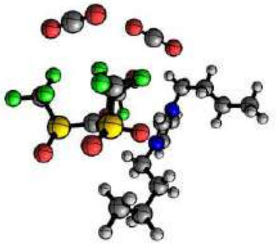	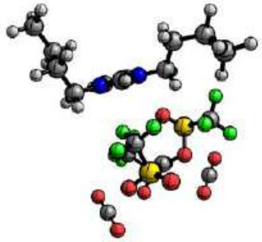
7.31	7.61	7.96	8.88	9.21
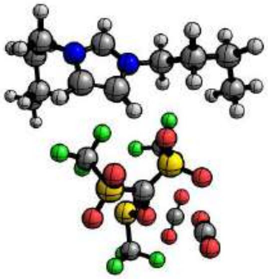				
20.88				

Table S30. Representations of lowest energy forms for $3CO_2[Dbim]^+[Methide]^-$ at the M06-2X-D3/6-31G(d,p) level with an implicit solvent model PCM. Relative energies are listed in kcal/mol.

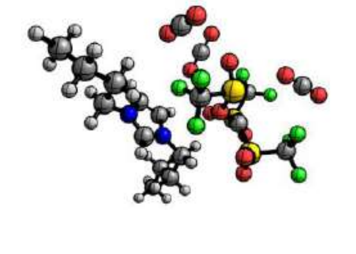	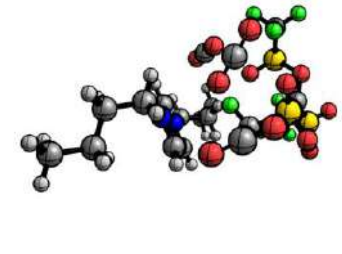	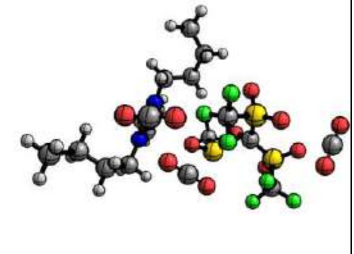	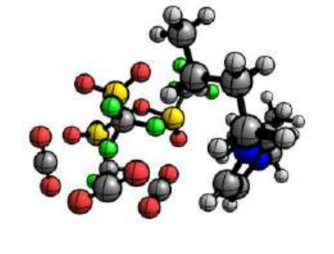	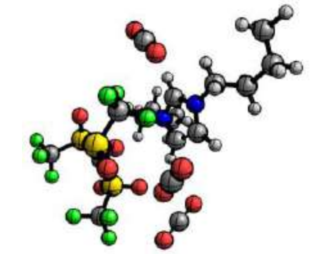
0.00	26.75	27.31	27.50	29.09
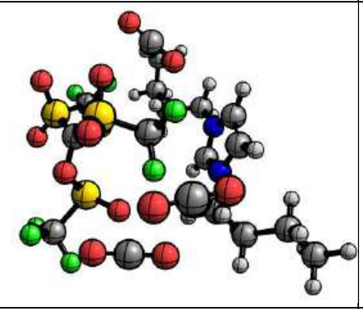	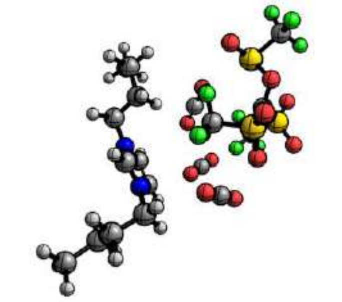	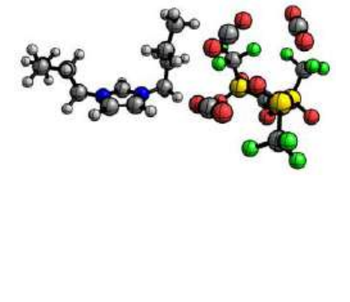	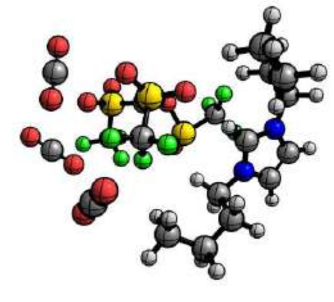	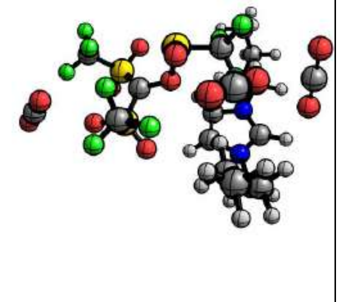
29.15	29.22	29.88	30.26	30.69
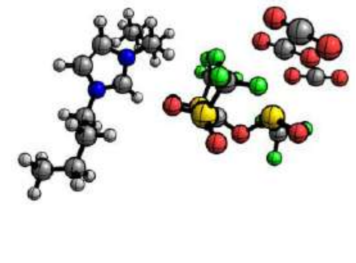	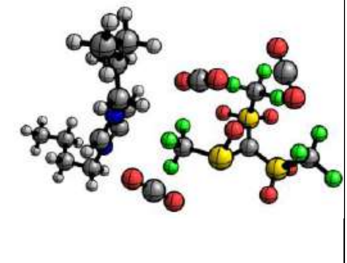	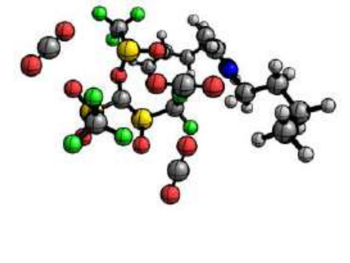	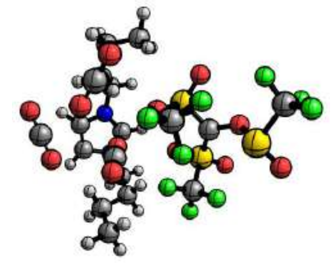	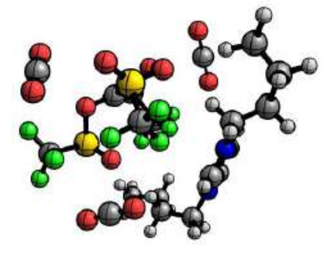
30.70	30.92	31.13	31.50	31.76

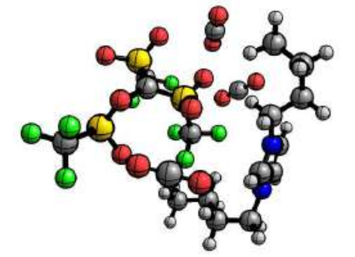	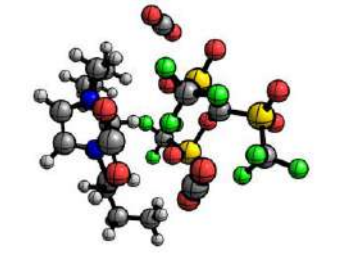	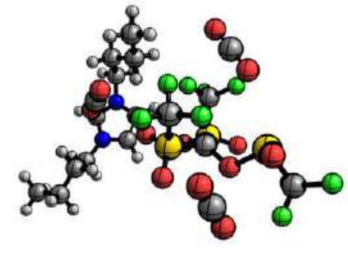	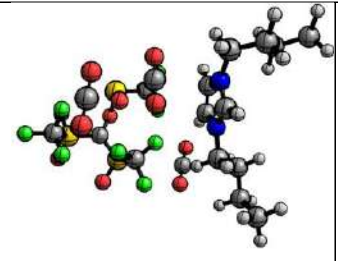	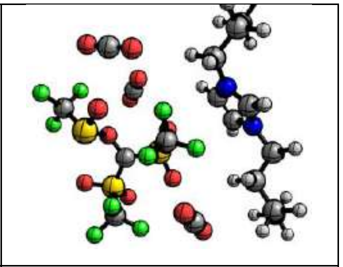
34.09	34.33	34.78	34.84	36.12
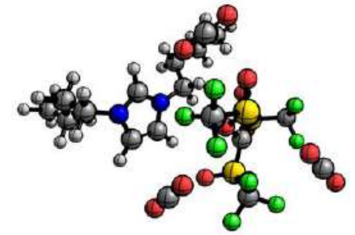				
39.29				

Table S31. Representations of lowest energy forms for $4CO_2[Dbim]^+[Methide]^-$ at the M06-2X-D3/6-31G(d,p) level with an implicit solvent model PCM. Relative energies are listed in kcal/mol.

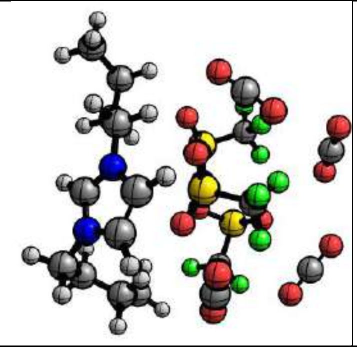	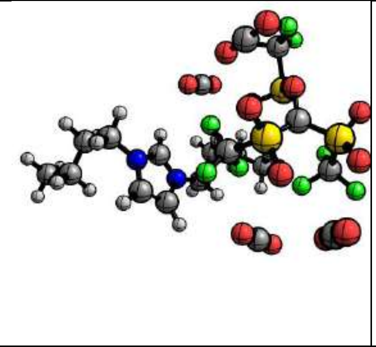	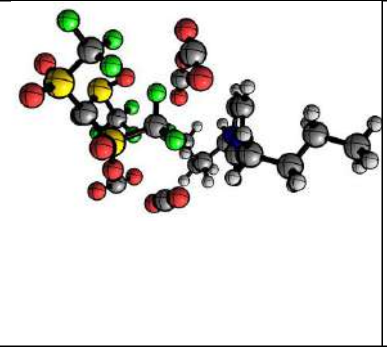	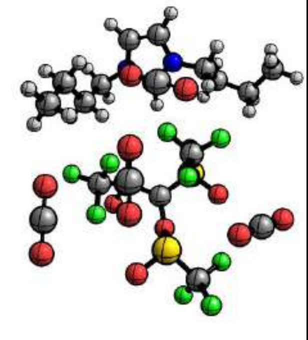	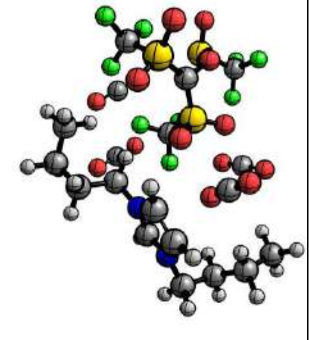
0.00	3.09	3.71	5.10	5.48

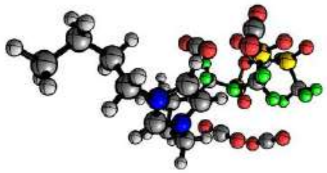	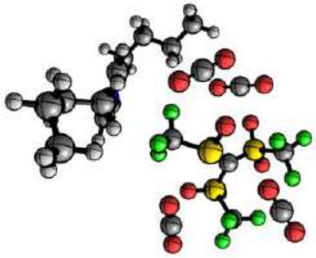	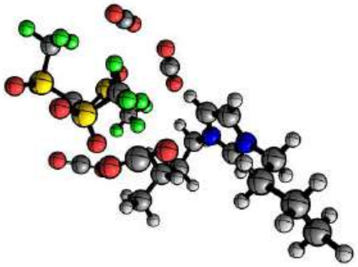	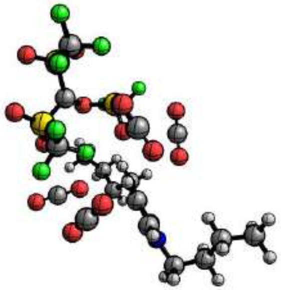	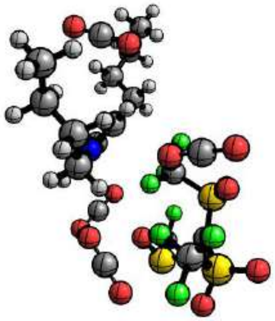
5.67	5.78	5.93	6.31	6.75
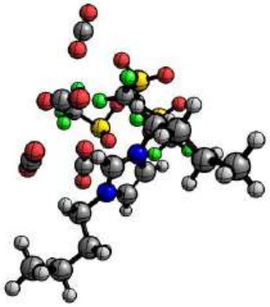	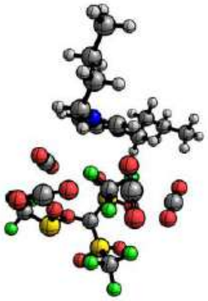	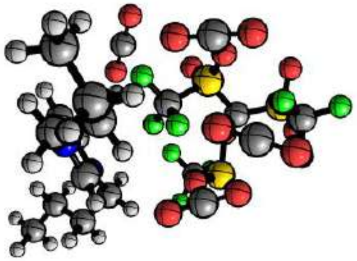	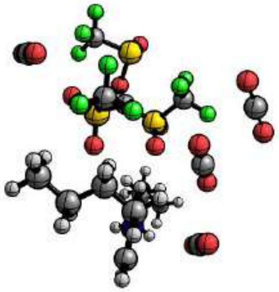	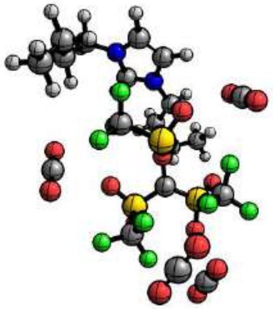
10.41	10.48	11.65	12.83	13.25

Table S32. Representations of lowest energy forms for $5CO_2[Dbim]^+[Methide]^-$ at the M06-2X-D3/6-31G(d,p) level with an implicit solvent model PCM. Relative energies are listed in kcal/mol.

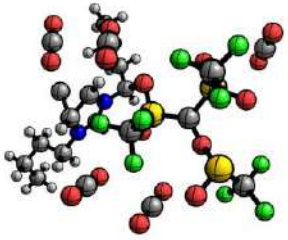	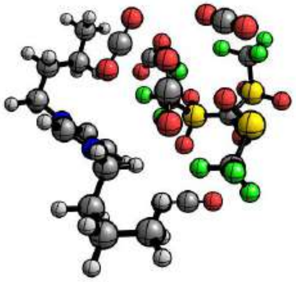	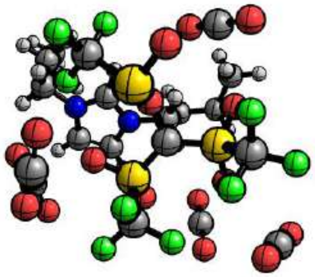	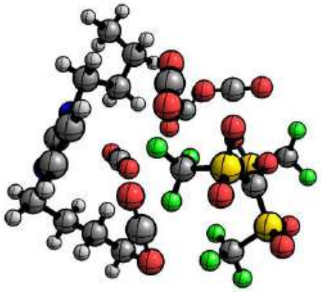	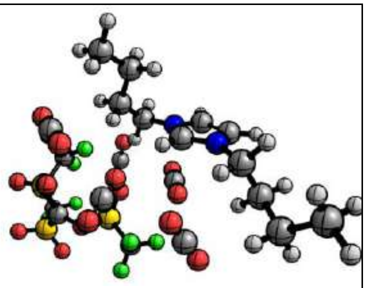

0.00	0.075	0.01	0.18	0.43
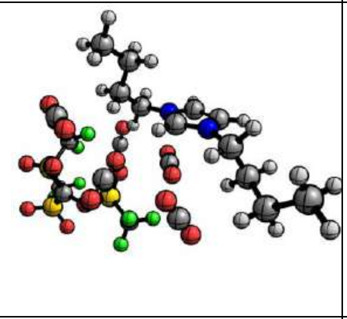	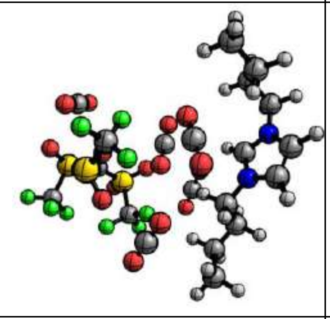	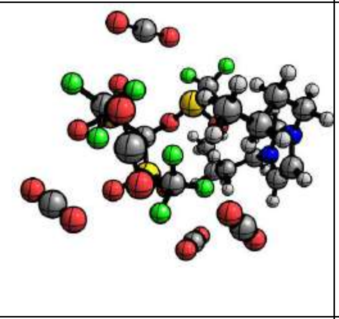	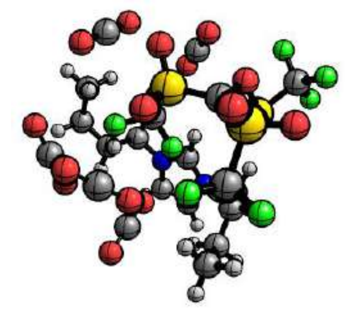	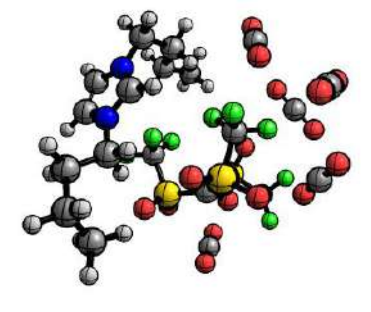
1.24	1.28	1.31	1.33	1.41
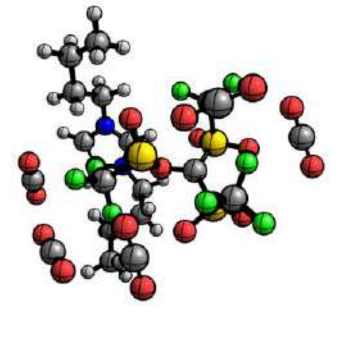	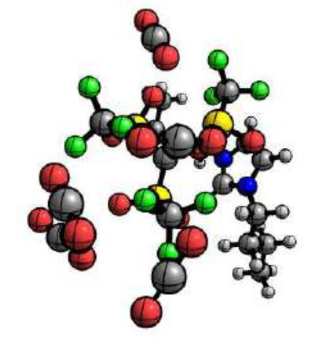	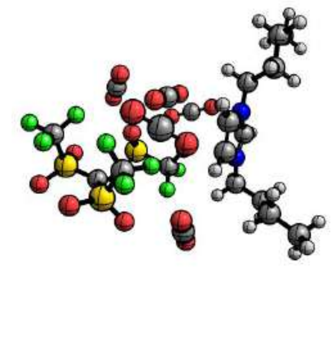	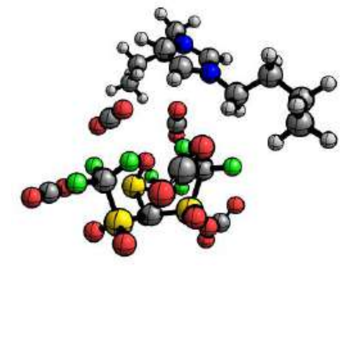	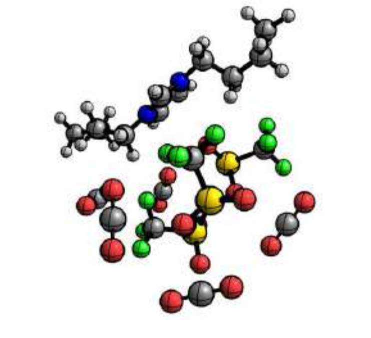
2.18	2.19	2.50	2.58	2.74
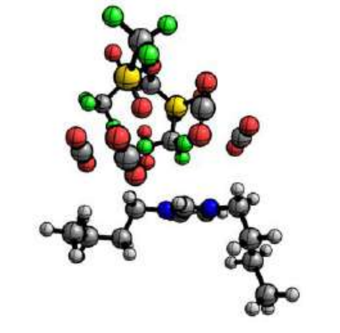	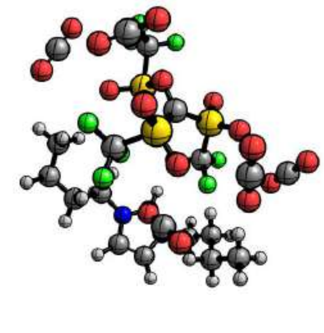	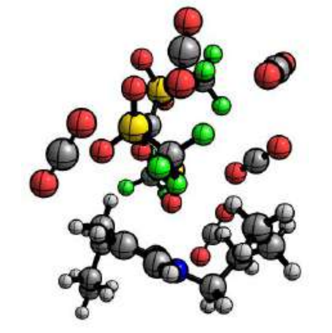	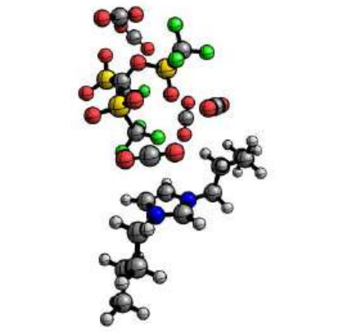	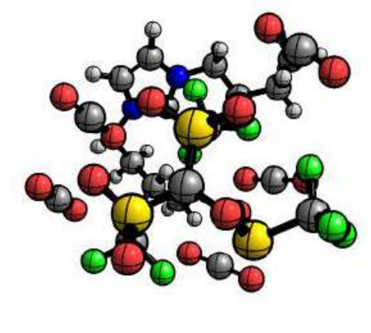
2.98	3.15	3.96	4.08	4.21

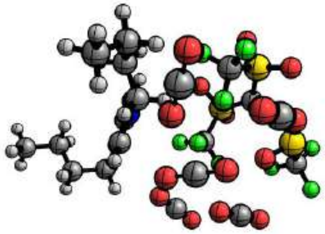	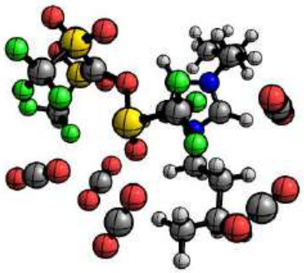	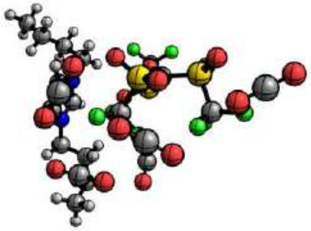	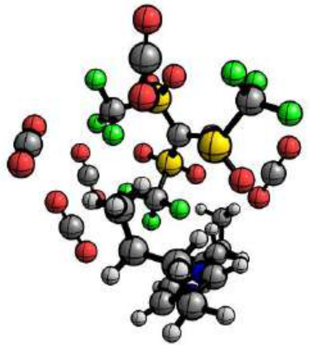	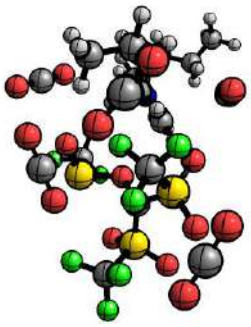
4.36	4.44	4.48	5.17	5.24
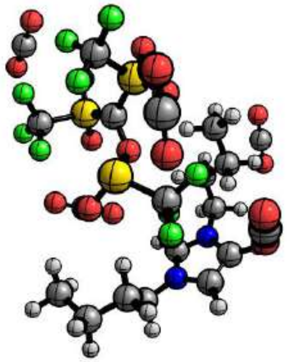	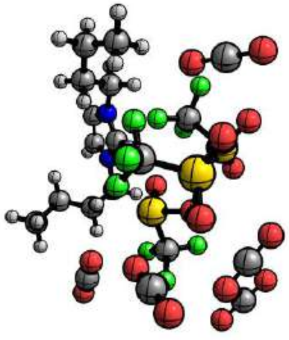	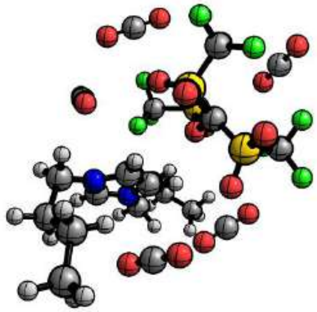	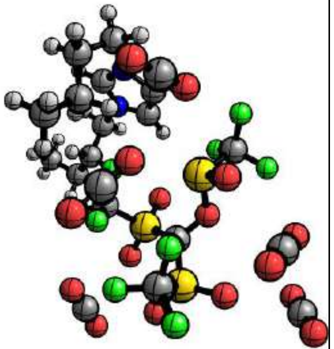	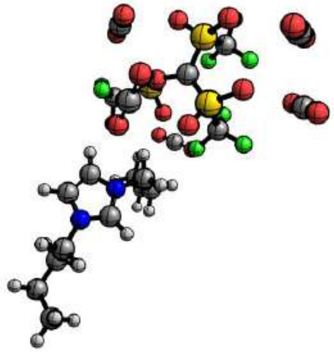
5.39	5.41	5.78	5.99	6.02

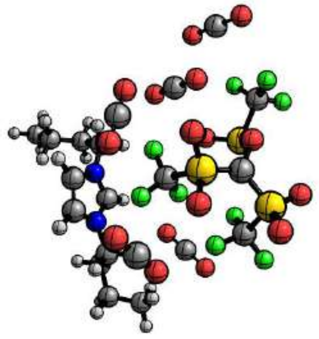	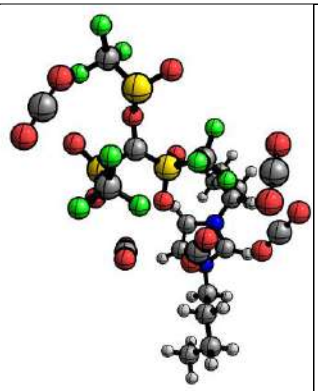	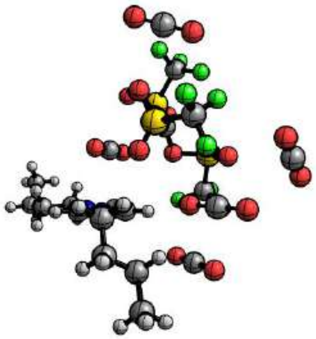		
6.54	6.88	7.28		

Table S33. Representations of lowest energy forms for $1CO_2[Hmim]^+[FAP]^-$ at the M06-2X-D3/6-31G(d,p) level with an implicit solvent model PCM. Relative energies are listed in kcal/mol.

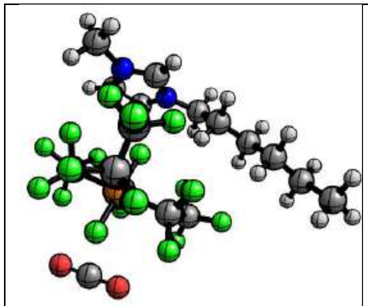	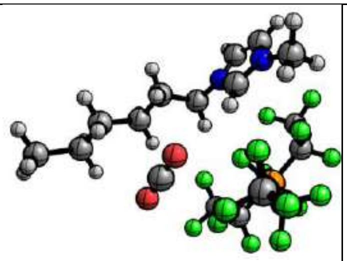	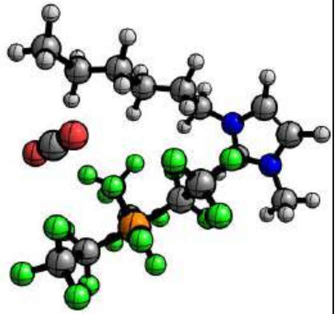	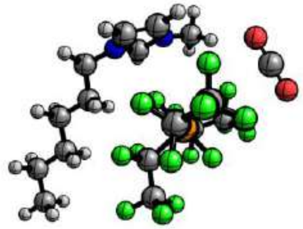	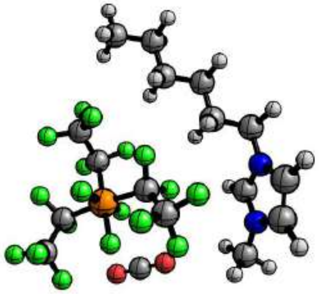
0.00	0.01	0.40	0.52	1.28

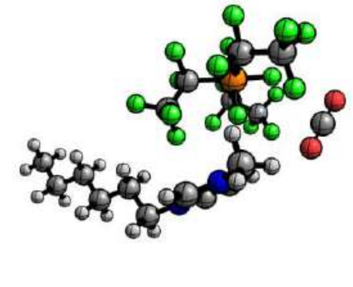	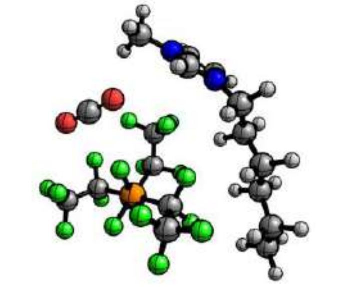	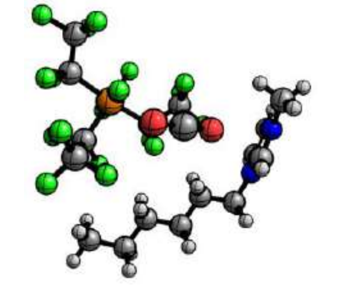	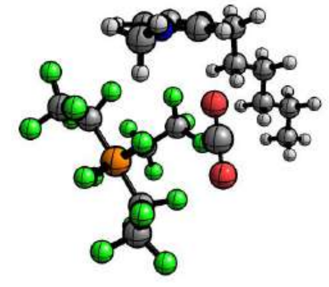	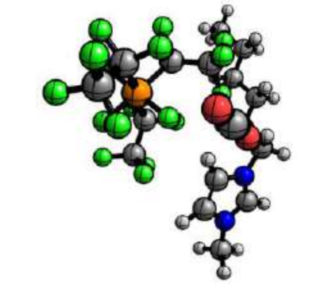
1.83	1.91	1.92	1.98	2.11
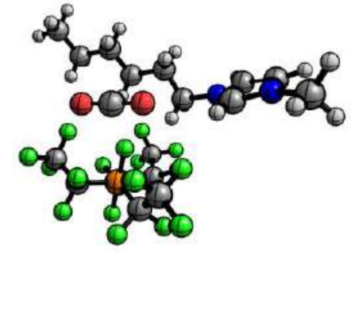	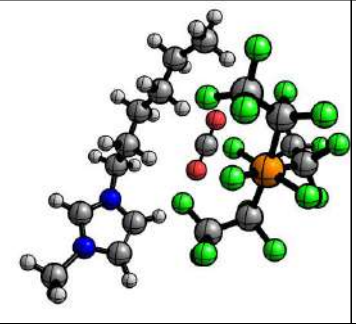	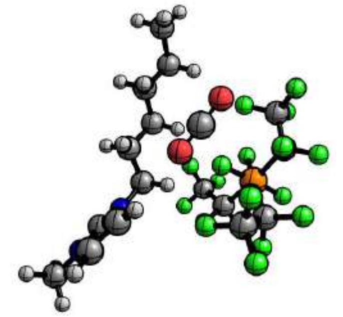	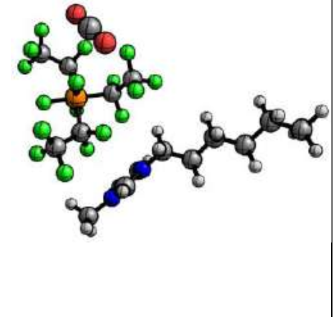	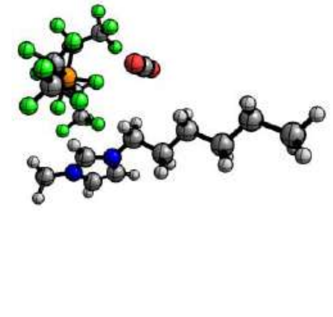
2.25	2.27	2.35	2.79	2.80
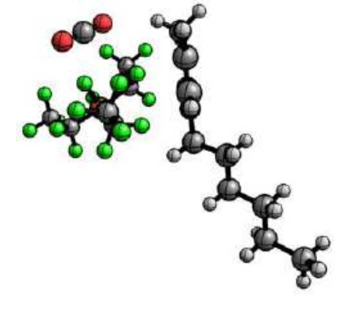	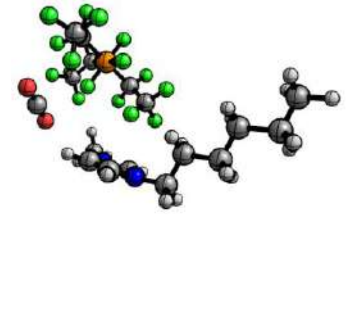	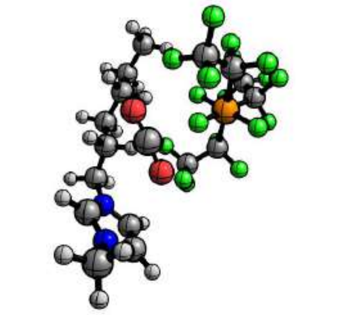	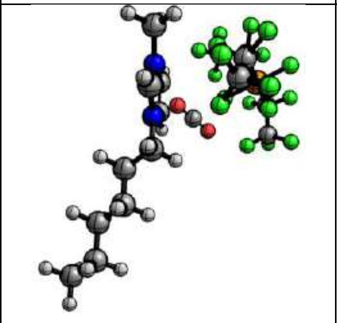	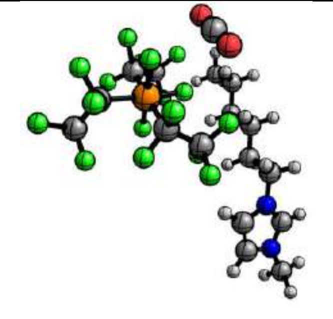
2.81	3.69	4.10	4.52	4.74

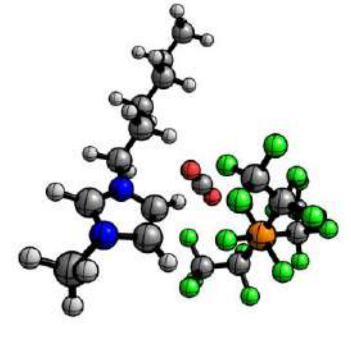	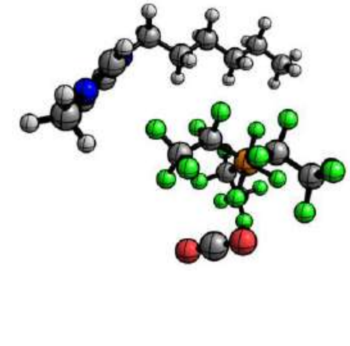	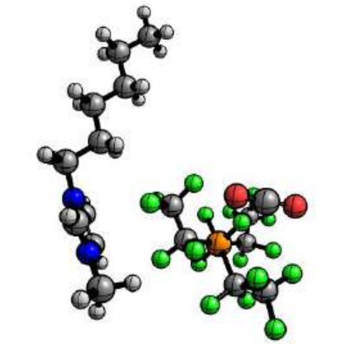	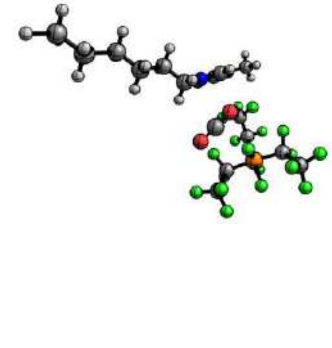	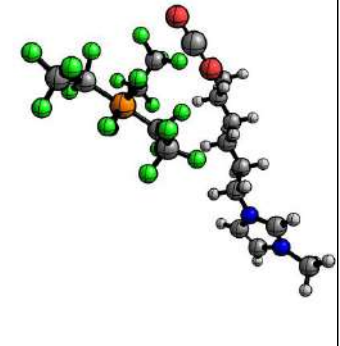
5.62	5.74	6.00	6.06	6.31
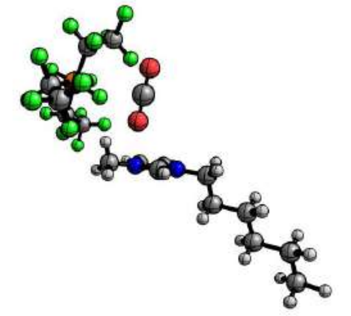	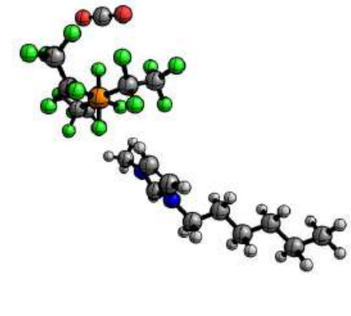	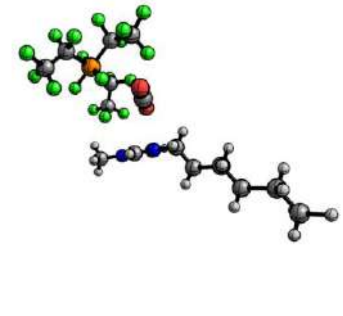	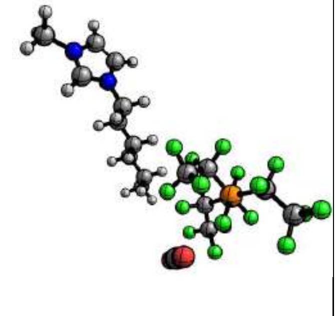	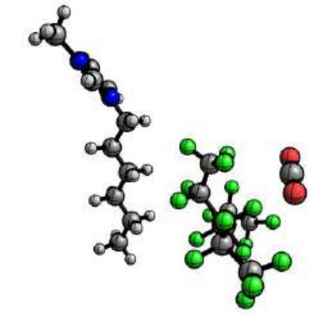
7.08	7.34	7.47	7.78	8.28
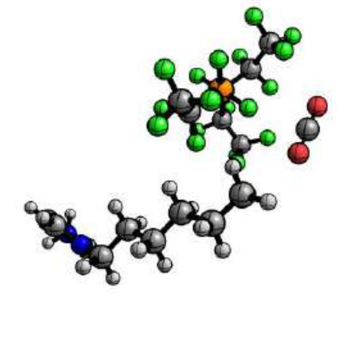	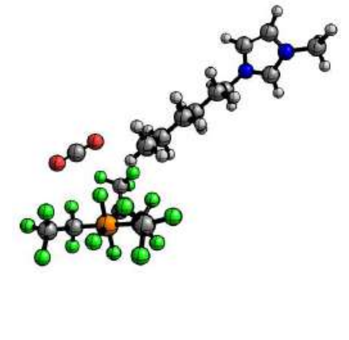	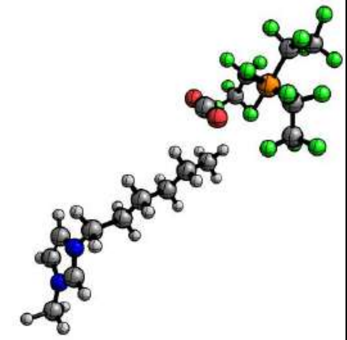	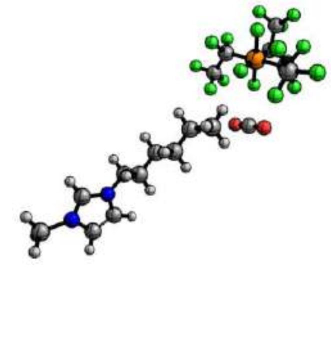	
8.91	8.99	10.27	10.44	

Table S34. Representations of lowest energy forms for $2CO_2[Hmim]^+[FAP]^-$ at the M06-2X-D3/6-31G(d,p) level with an implicit solvent model PCM. Relative energies are listed in kcal/mol.

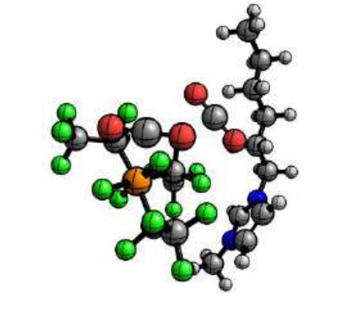	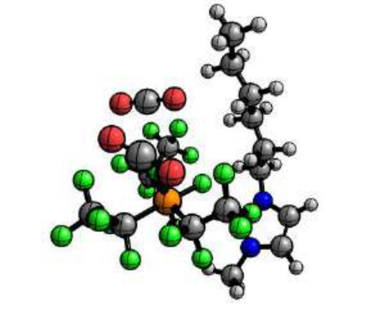	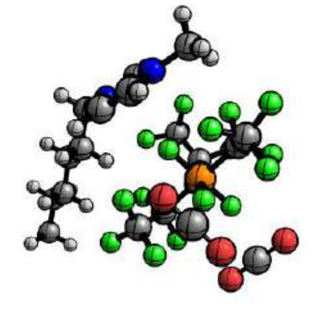	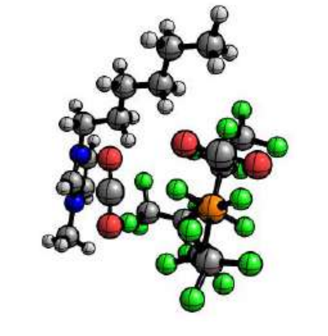	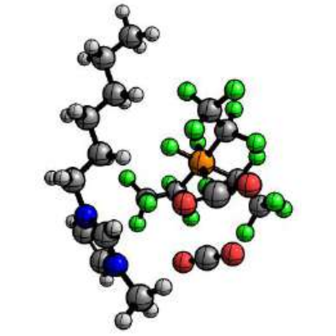
0.00	0.46	0.54	1.40	1.72
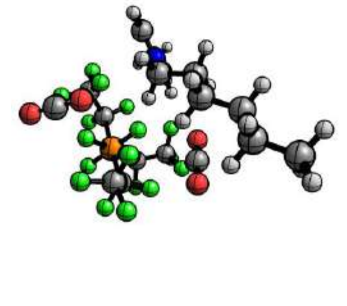	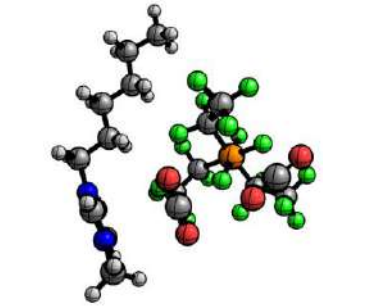	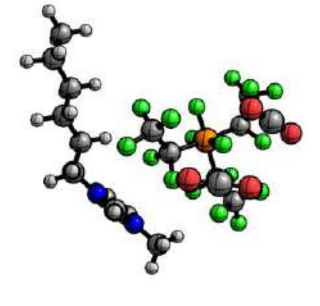	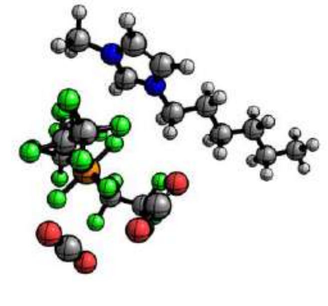	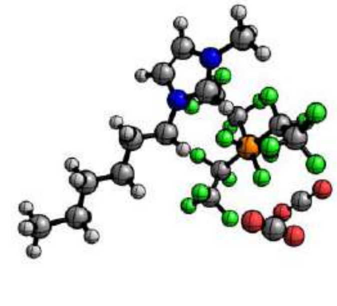
1.73	1.93	2.76	2.83	2.84
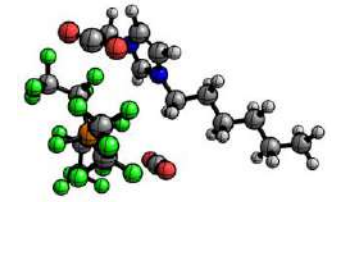	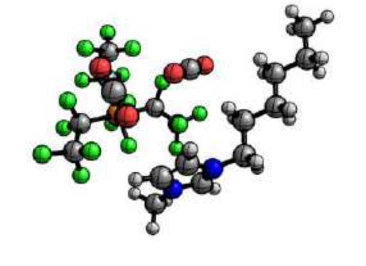	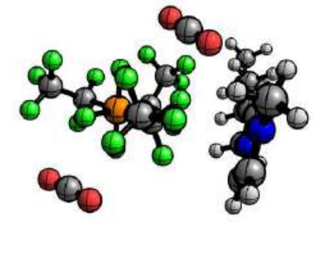	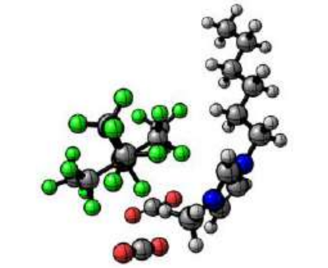	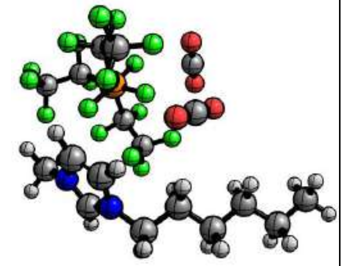
2.84	2.91	3.10	3.28	3.42

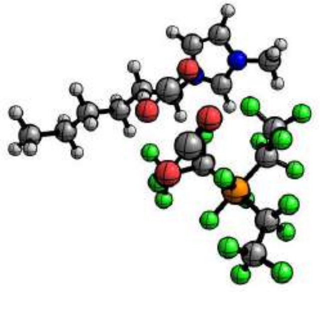	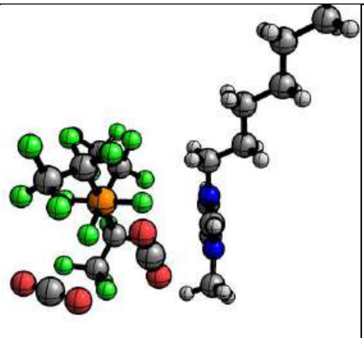	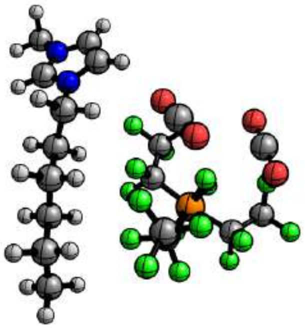	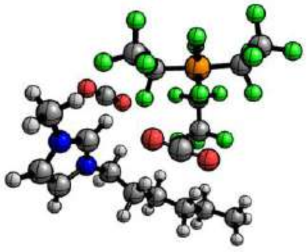	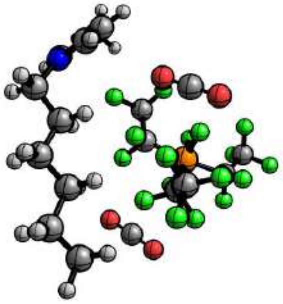
4.03	4.07	4.15	4.67	4.68
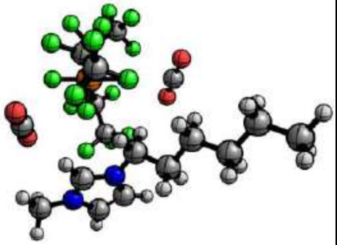	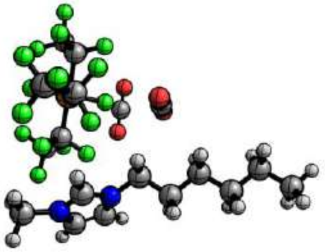	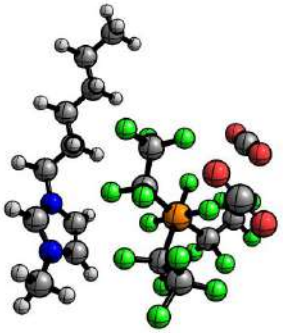	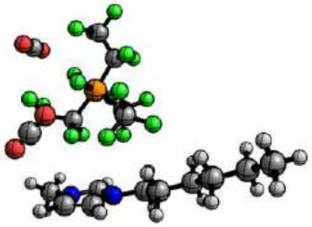	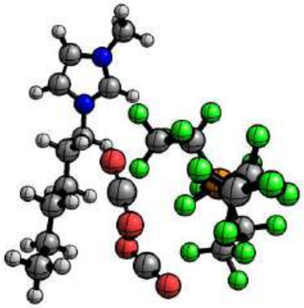
5.00	5.21	5.51	5.84	6.15
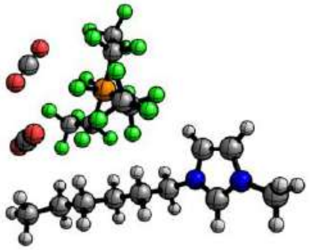	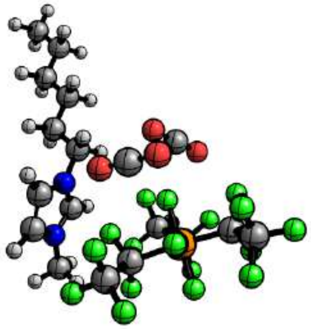	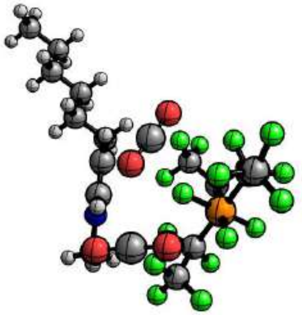	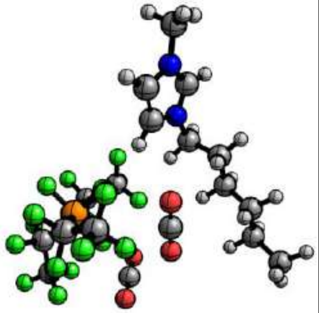	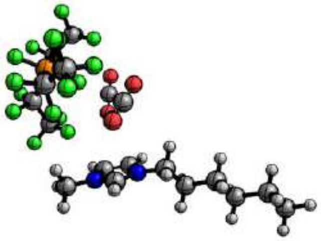
6.95	7.42	7.58	8.56	8.60

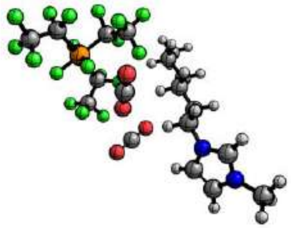	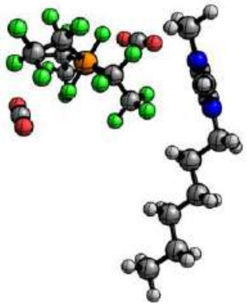	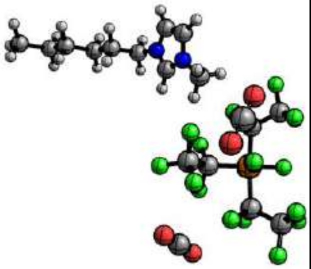	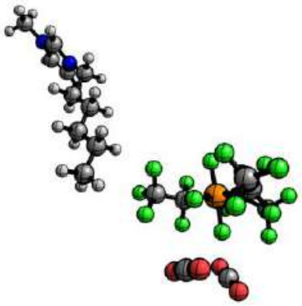	
8.76	10.12	13.19	16.04	

Table S35. Representations of lowest energy forms for $3CO_2[Hmim]^+[FAP]^-$ at the M06-2X-D3/6-31G(d,p) level with an implicit solvent model PCM. Relative energies are listed in kcal/mol.

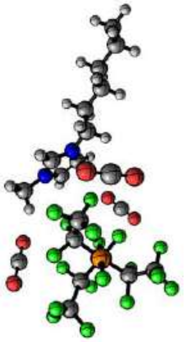	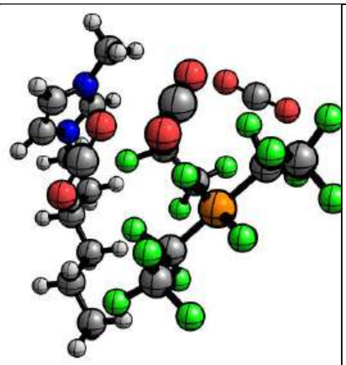	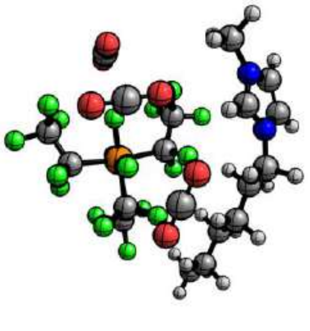	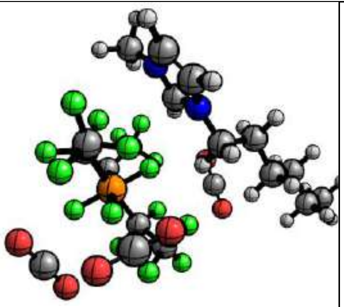	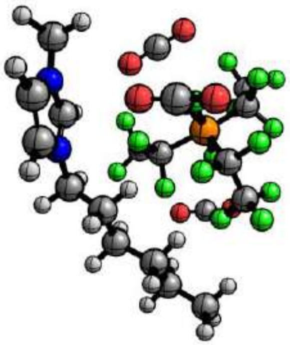
0.00	3.92	4.59	6.98	7.19

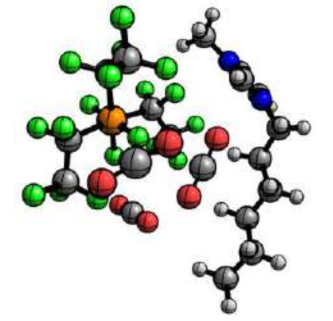	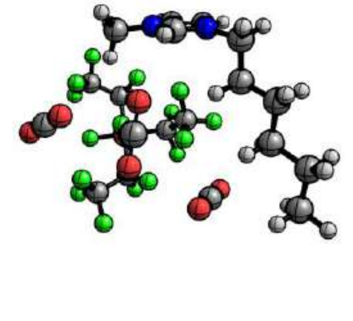	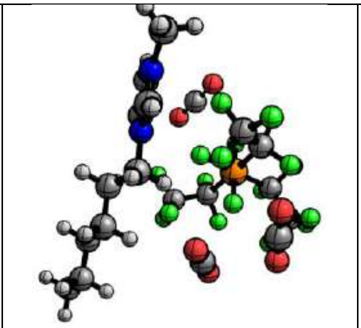	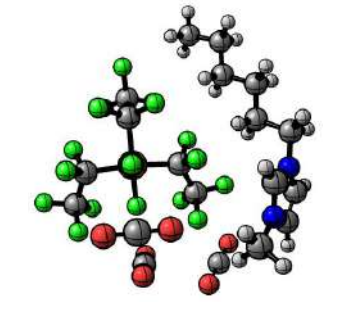	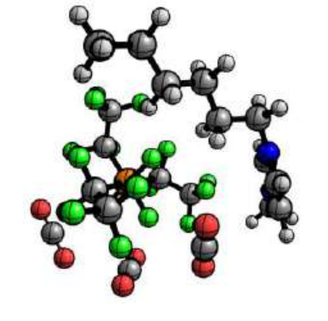
7.55	7.67	8.08	8.17	8.24
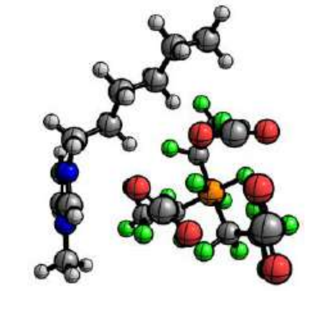	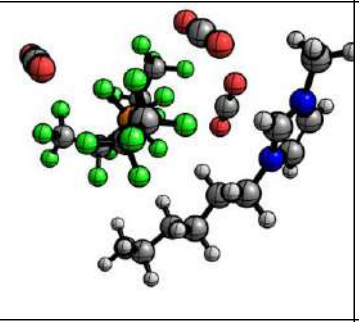	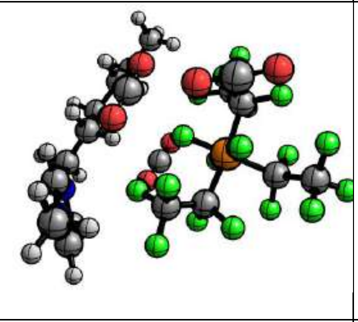	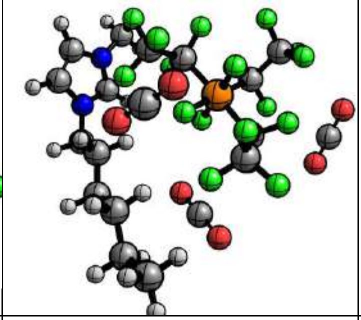	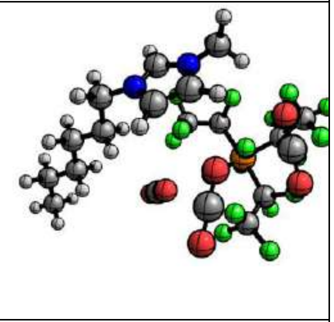
8.44	8.94	9.19	10.03	10.12
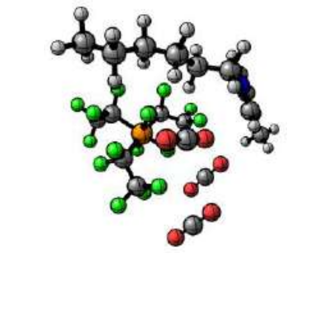	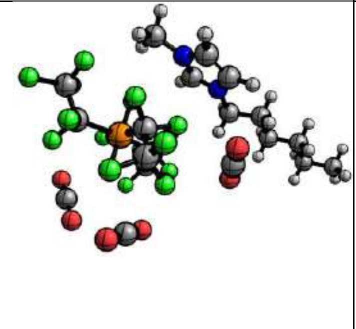	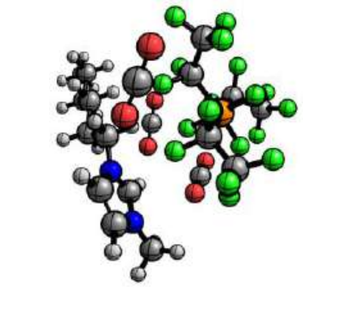	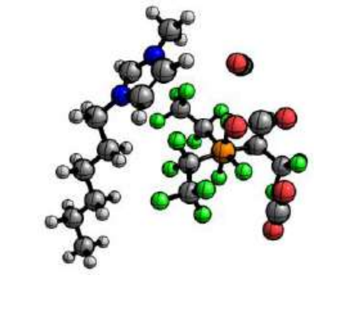	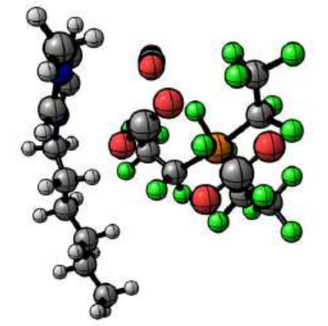
10.30	10.34	11.04	11.36	11.71

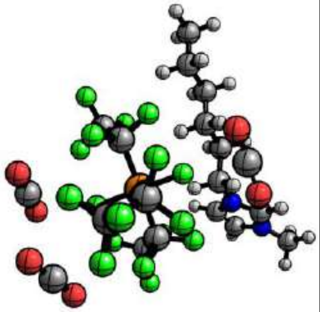	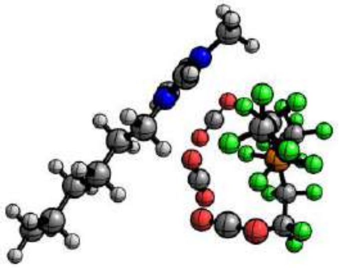	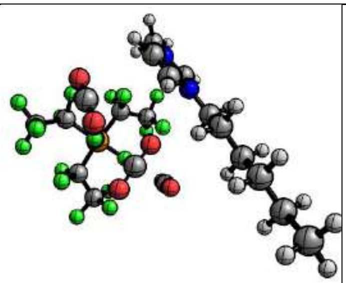	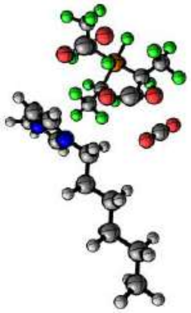	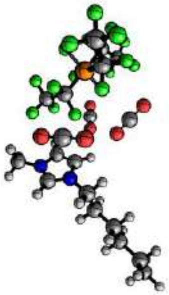
12.27	12.53	12.54	12.99	13.32
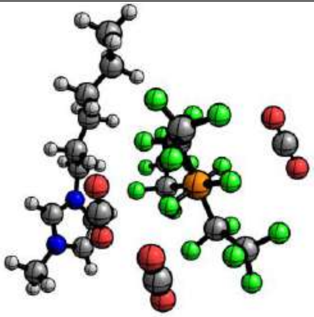	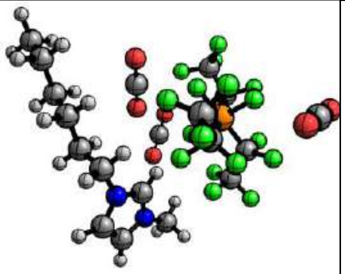	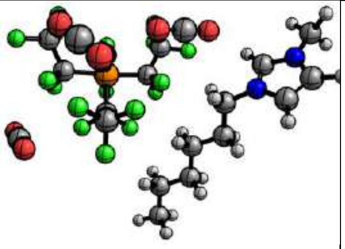	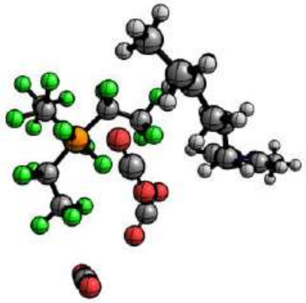	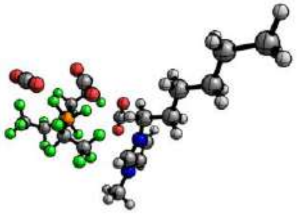
14.31	14.78	15.68	16.45	16.50
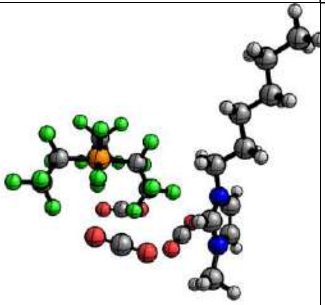	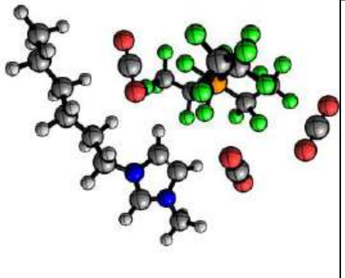	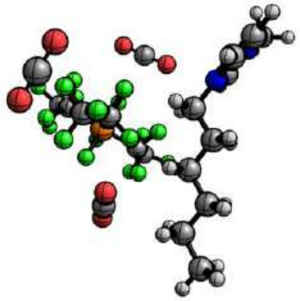		
17.01	17.59	21.92		

Table S36. Representations of lowest energy forms for $4CO_2[Hmim]^+[FAP]^-$ at the M06-2X-D3/6-31G(d,p) level with an implicit solvent model PCM. Relative energies are listed in kcal/mol.

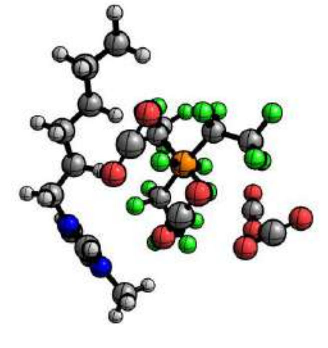	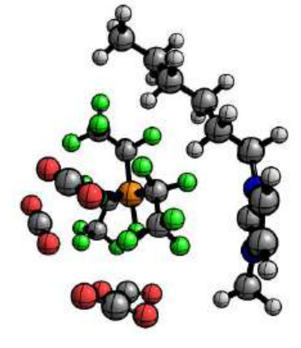	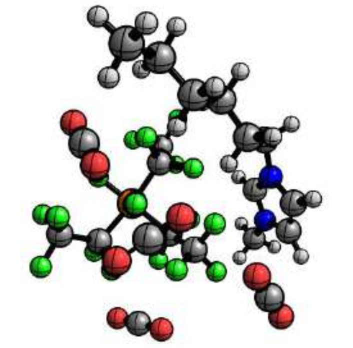	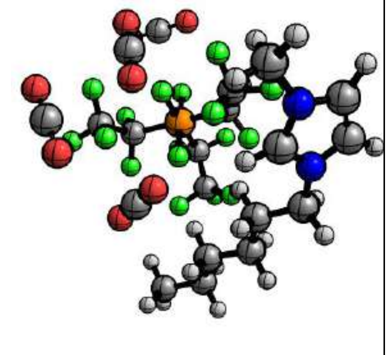	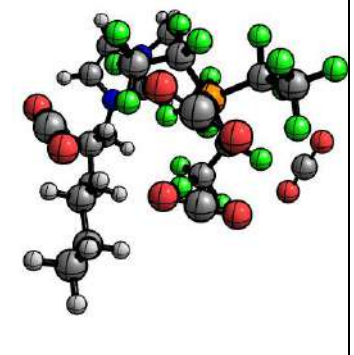
0.00	0.10	0.27	0.87	1.06
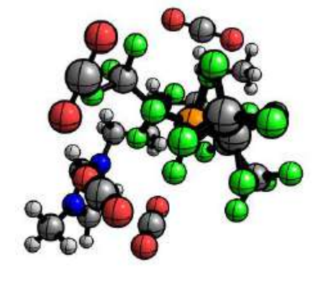	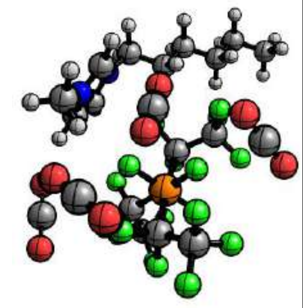	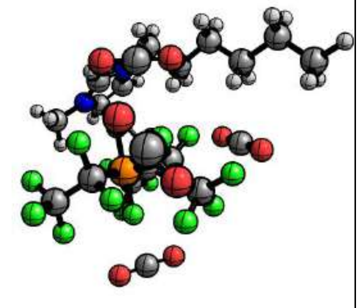	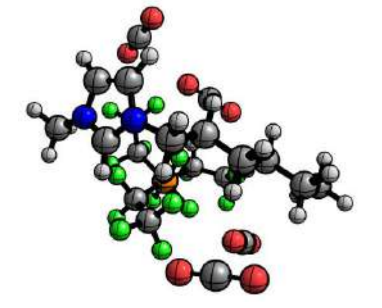	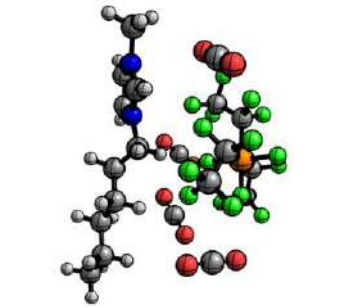
1.20	1.75	1.97	2.12	2.40
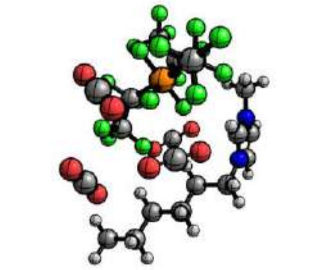	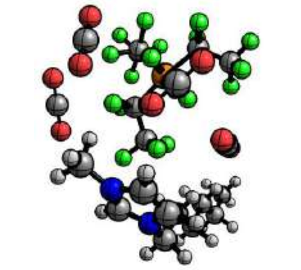	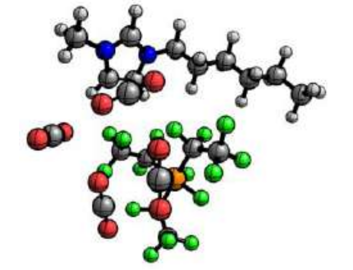	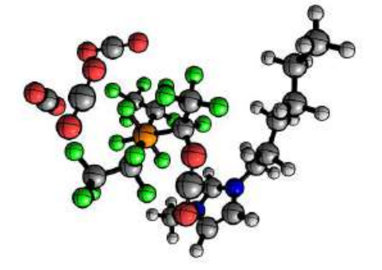	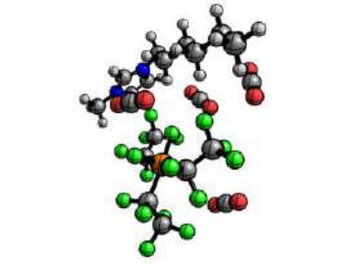
2.42	2.56	2.60	3.03	3.57

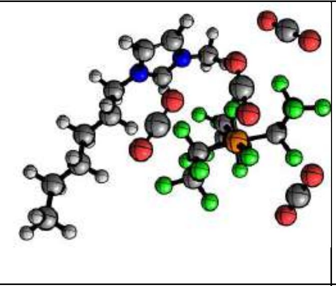	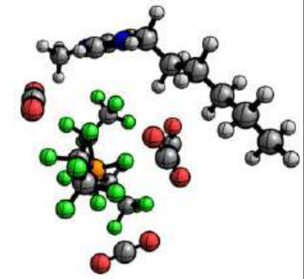	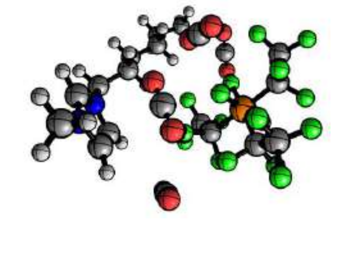	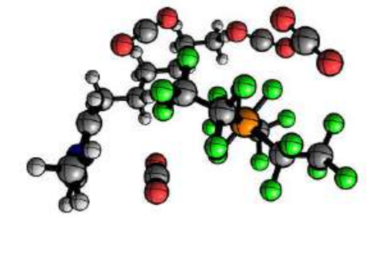	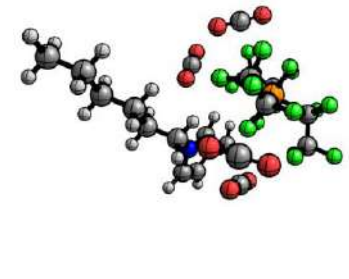
3.80	4.04	4.25	5.03	5.14
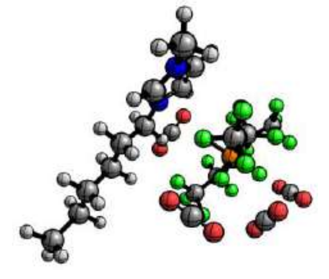	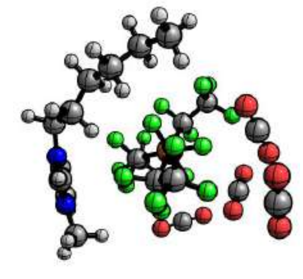	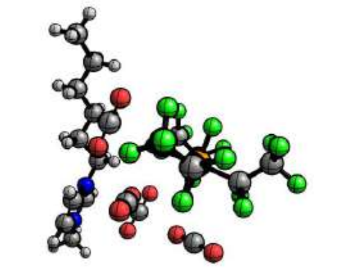	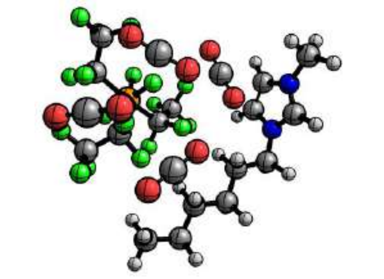	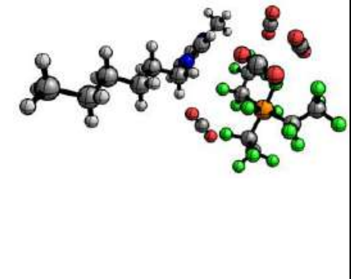
5.17	5.50	5.55	5.65	5.65
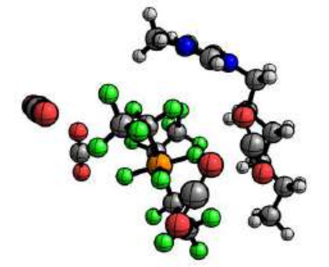	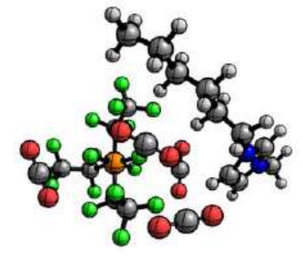	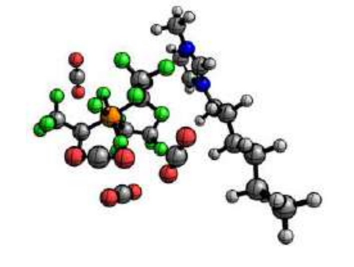	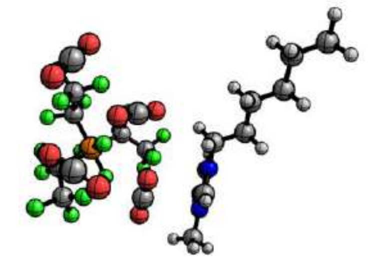	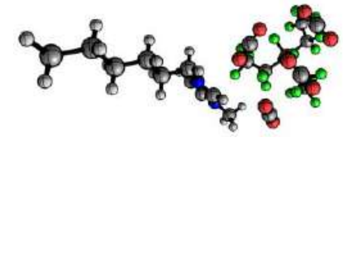
6.23	6.31	7.26	7.85	8.12

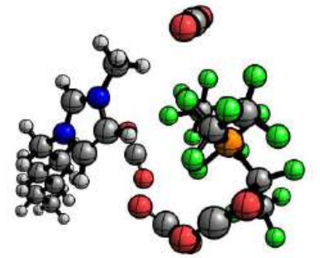	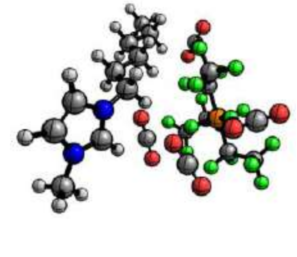	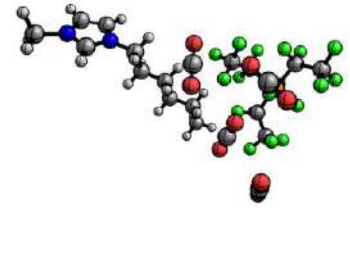	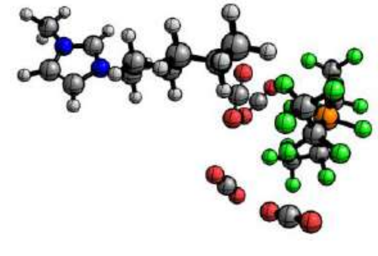	
8.73	9.38	9.90	11.49	

Table S37. Representations of lowest energy forms for $5CO_2[Hmim]^+[FAP]^-$ at the M06-2X-D3/6-31G(d,p) level with an implicit solvent model PCM. Relative energies are listed in kcal/mol.

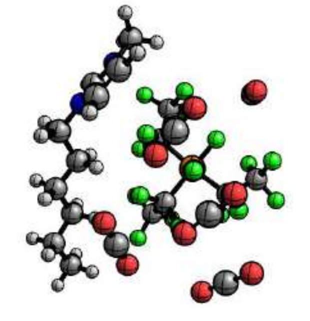	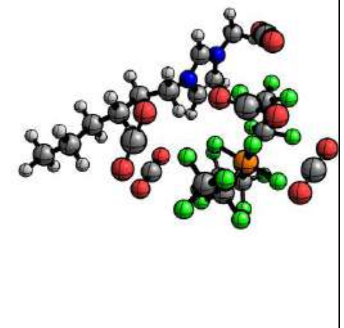	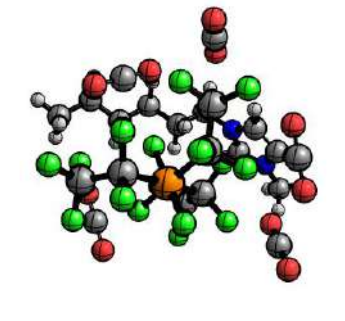	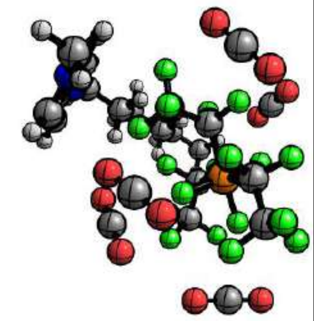	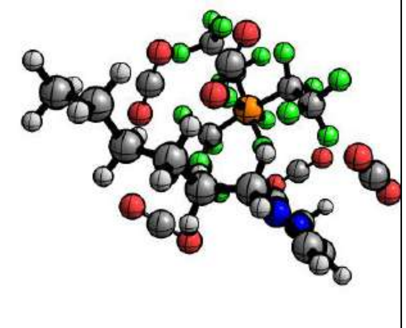
0.00	0.99	1.46	1.73	2.14
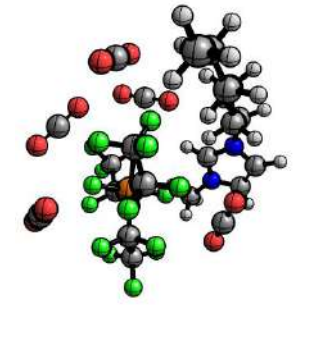	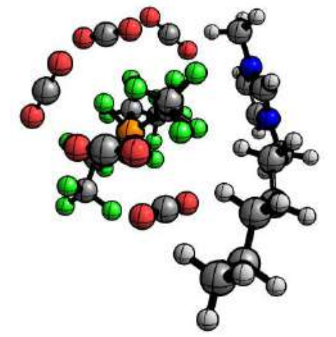	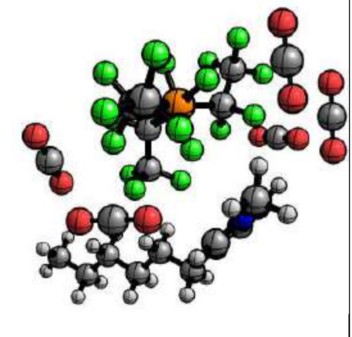	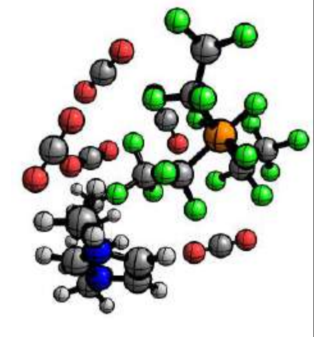	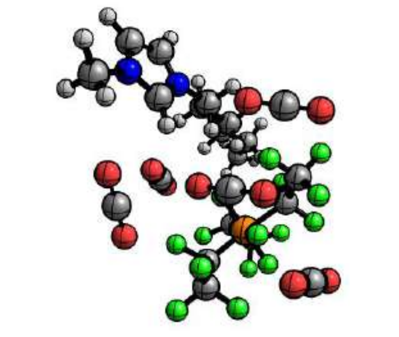

2.95	3.21	4.56	4.77	4.97
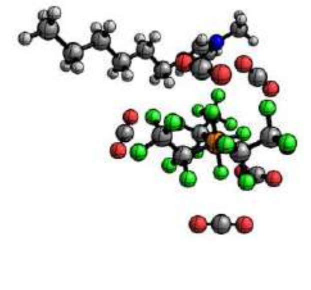	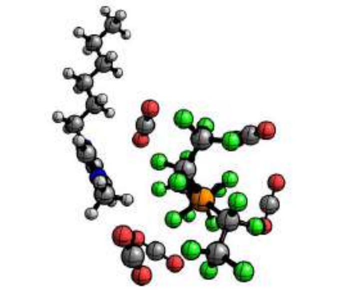	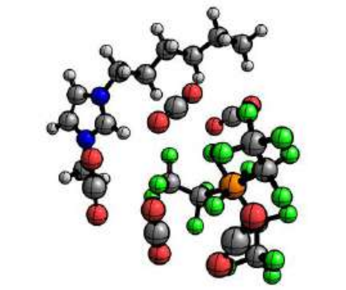	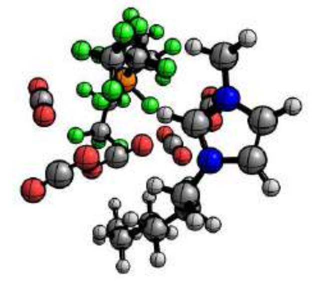	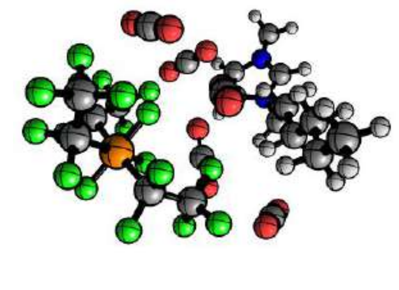
5.05	5.10	5.12	5.16	5.44
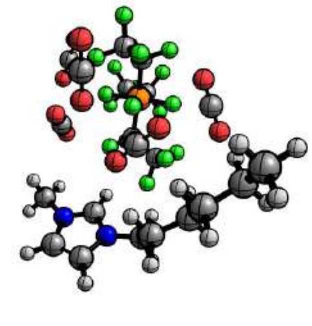	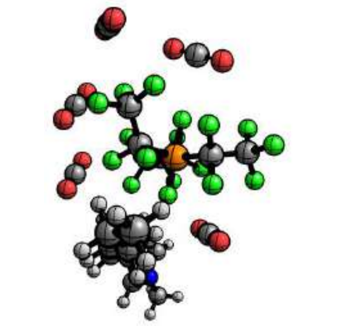	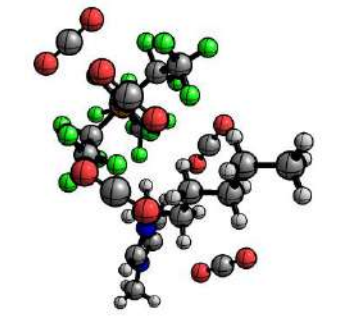	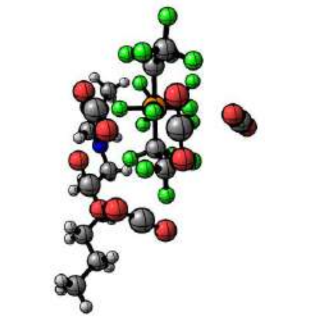	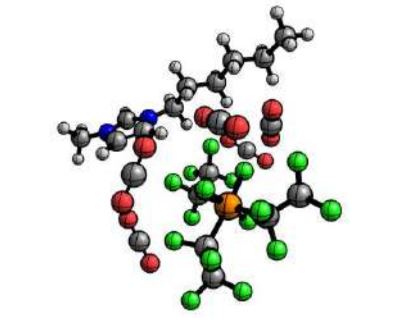
5.45	5.52	5.53	5.61	6.04
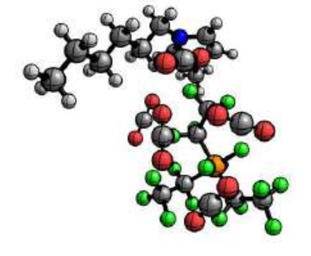	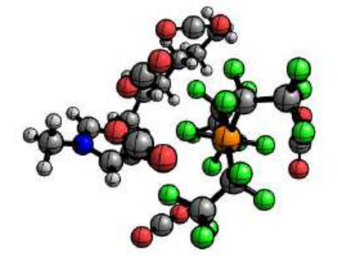	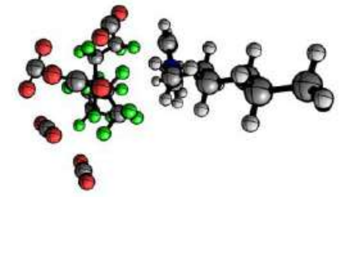	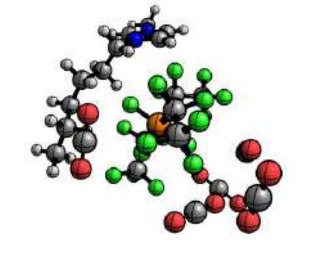	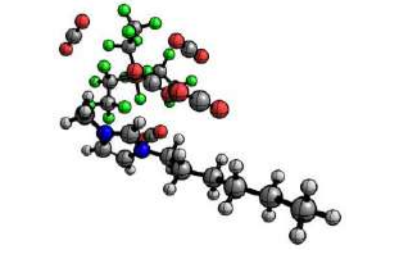
6.27	6.47	6.59	6.78	7.00

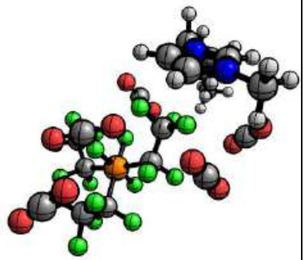	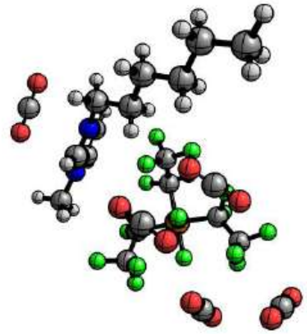	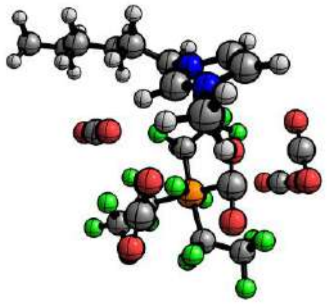	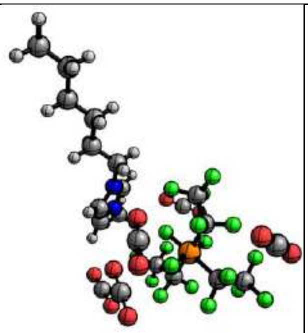	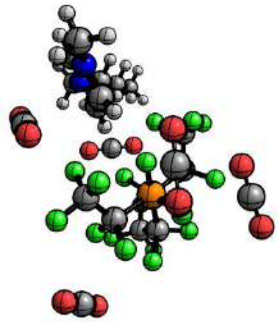
7.16	7.51	7.75	8.20	8.31
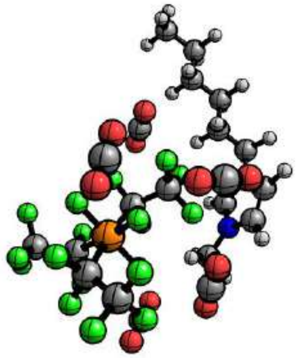	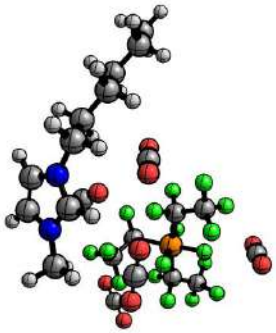	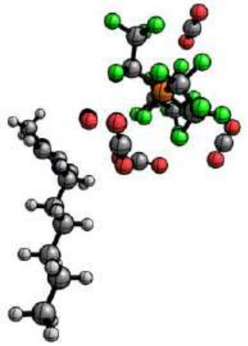		
8.85	11.40	11.49		

Table S38. Representations of lowest energy forms for $1CO_2[Hmim]^+[Methide]^-$ at the M06-2X-D3/6-31G(d,p) level with an implicit solvent model PCM. Relative energies are listed in kcal/mol.

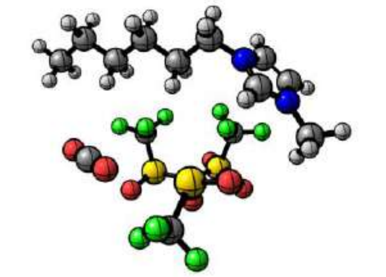	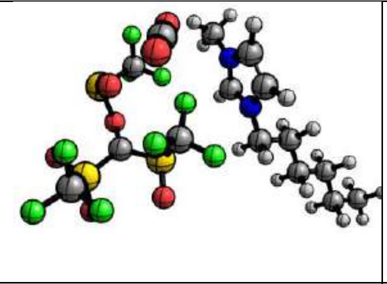	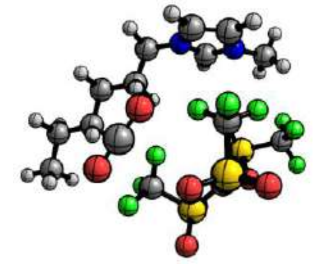	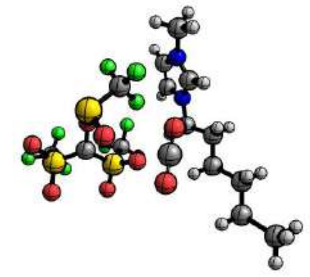	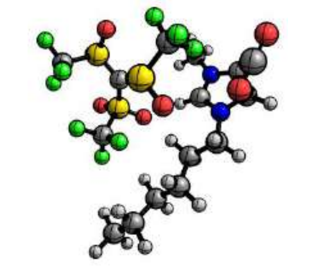
0.00	0.76	0.93	1.18	1.25
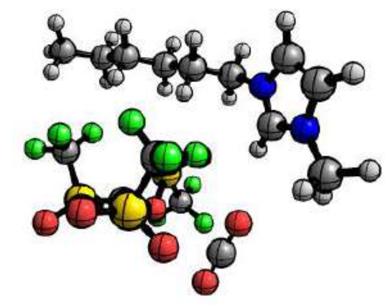	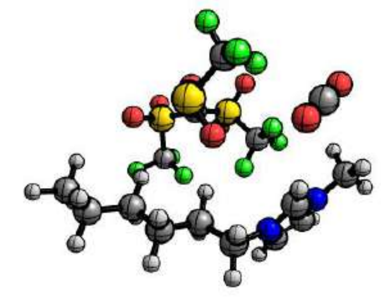	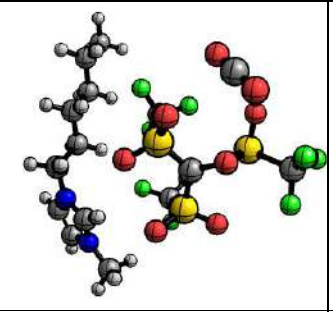	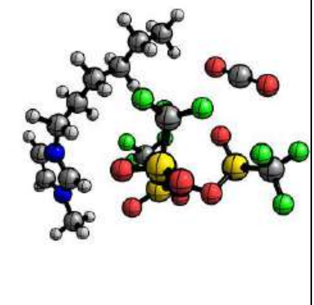	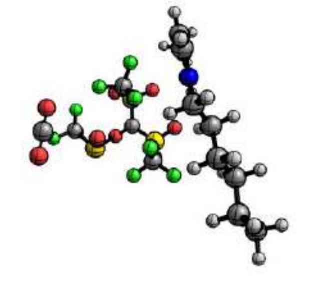
1.34	1.42	1.56	1.73	1.98
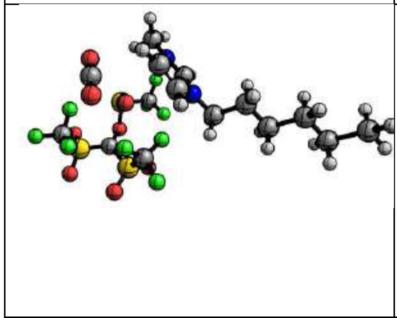	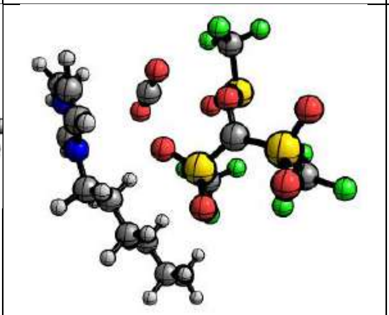	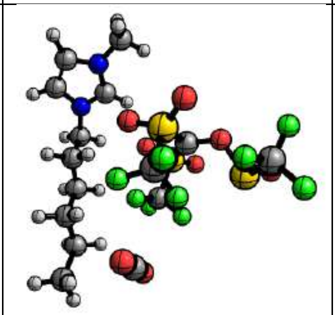	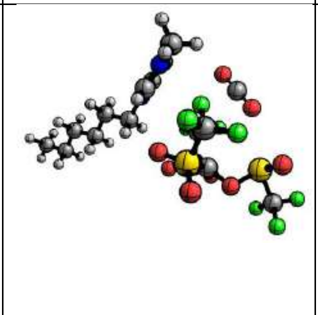	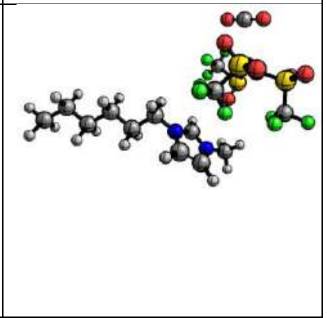
2.60	2.82	2.96	2.97	3.12

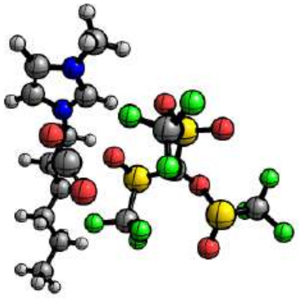	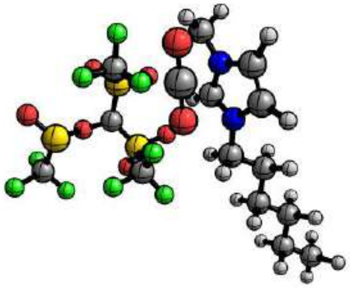	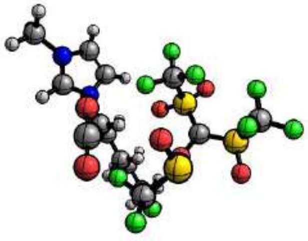	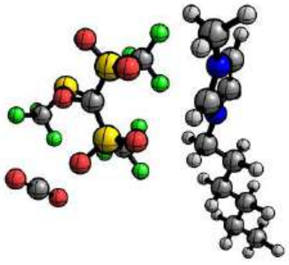	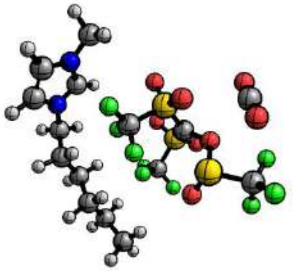
3.20	3.30	3.35	3.44	3.60
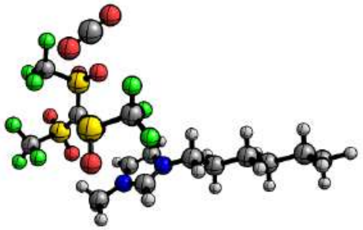	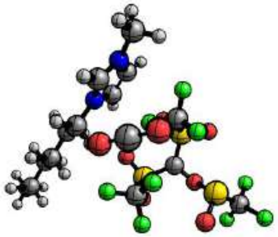	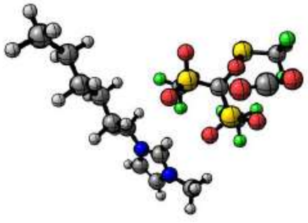	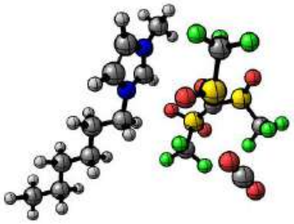	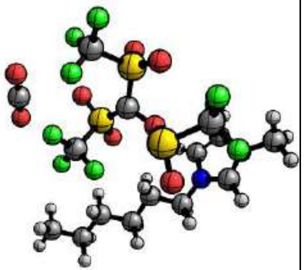
3.91	4.13	4.19	4.46	4.80
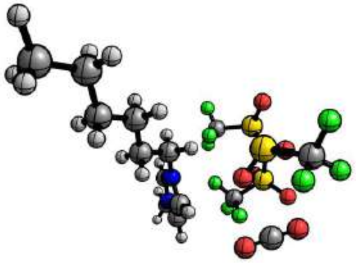	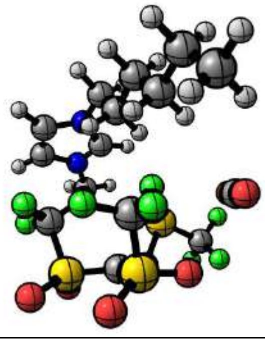	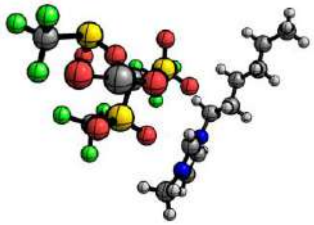	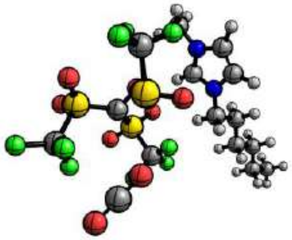	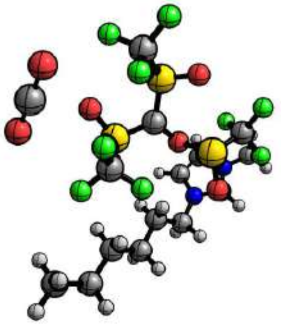
4.99	5.53	5.98	6.02	6.46

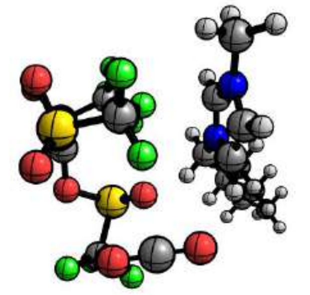				
6.52				

Table S39. Representations of lowest energy forms for $2CO_2$[Hmim]$^+$[Methide]$^-$ at the M06-2X-D3/6-31G(d,p) level with an implicit solvent model PCM. Relative energies are listed in kcal/mol.

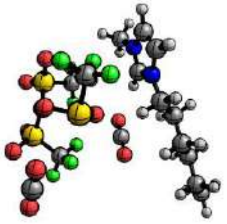	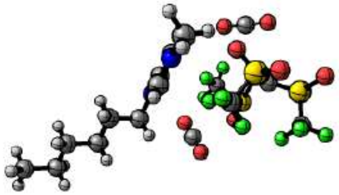	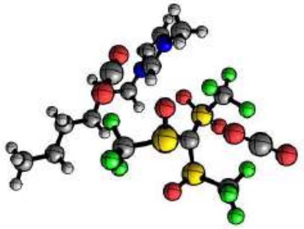	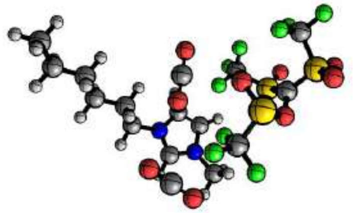	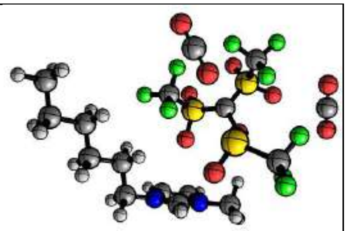
0.00	0.55	0.99	1.57	1.65
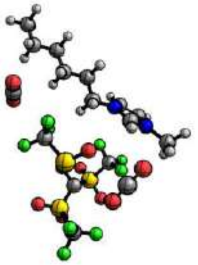	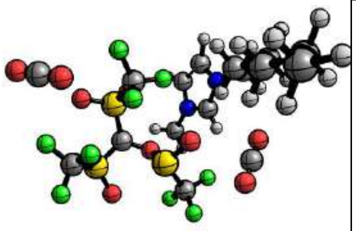	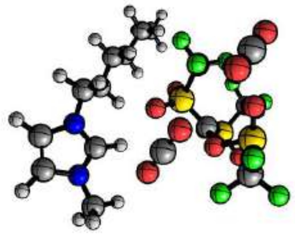	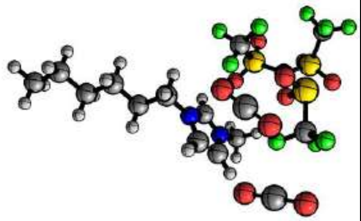	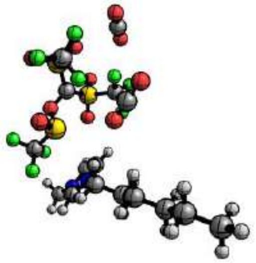
1.82	2.04	2.07	2.15	2.44

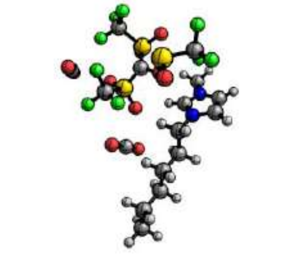	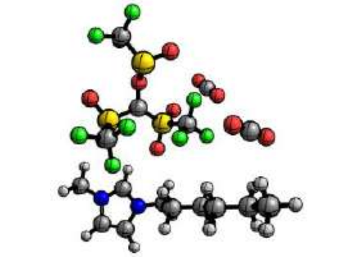	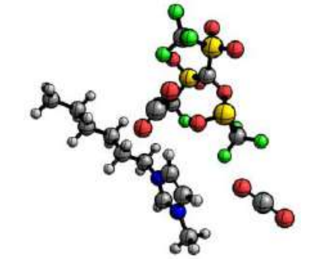	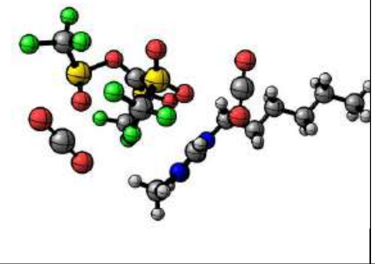	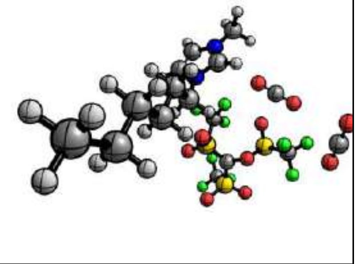
2.66	2.67	2.78	2.94	2.97
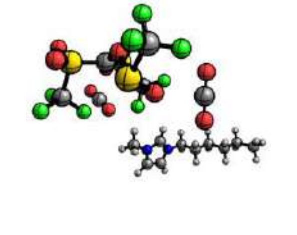	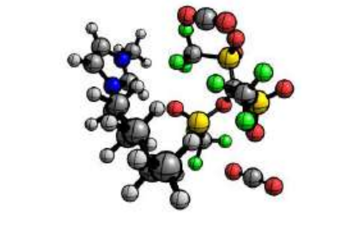	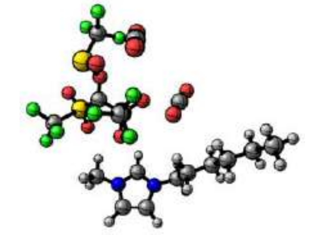	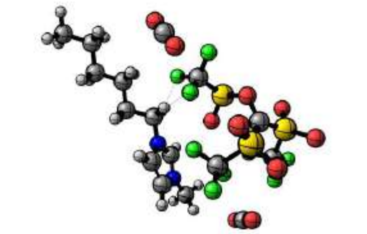	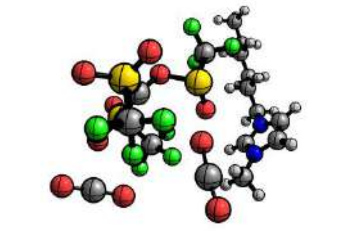
3.44	3.75	3.78	3.82	4.03
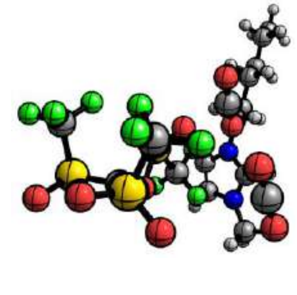	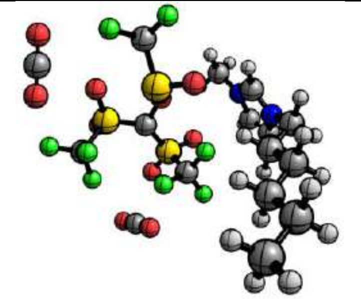	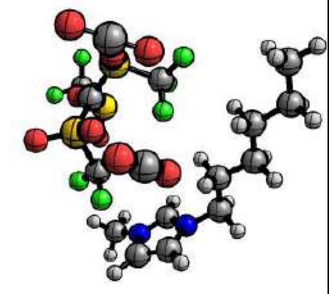	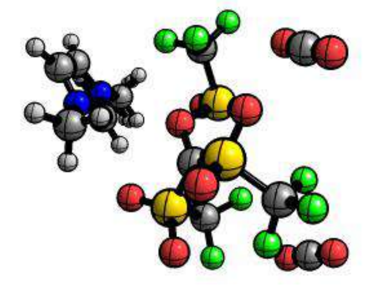	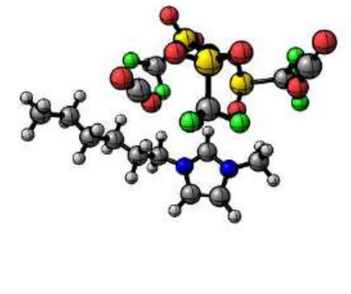
4.18	4.72	4.80	5.33	5.34

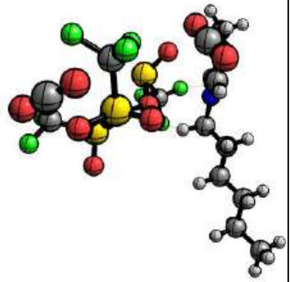	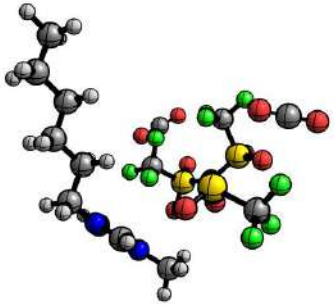	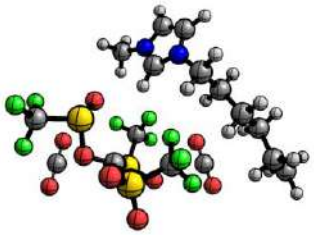	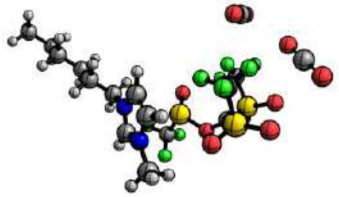	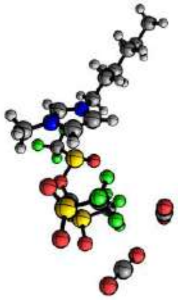
5.54	5.90	6.20	6.59	7.51
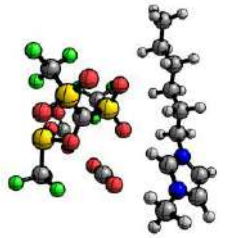				
7.78				

Table S40. Representations of lowest energy forms for $3CO_2[Hmim]^+[Methide]^-$ at the M06-2X-D3/6-31G(d,p) level with an implicit solvent model PCM. Relative energies are listed in kcal/mol.

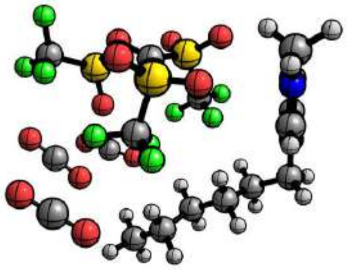	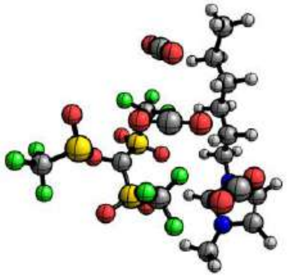	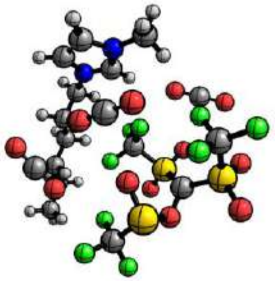	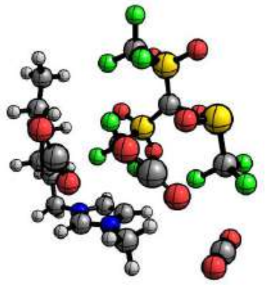	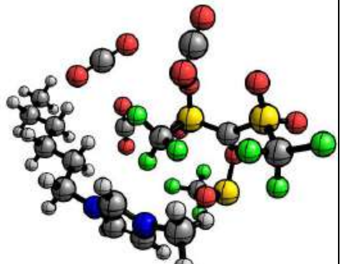
0.00	0.77	1.05	1.22	1.42

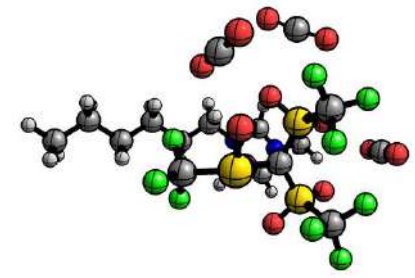	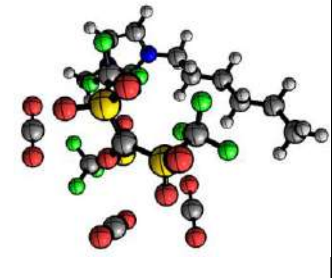	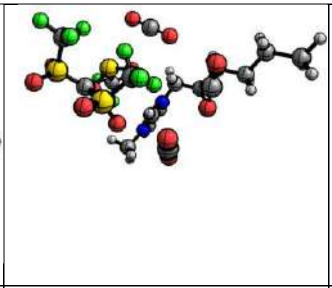	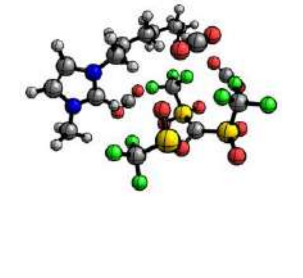	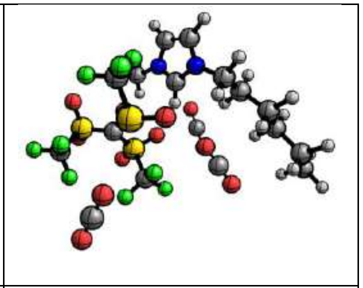
1.51	2.05	2.85	2.86	3.83
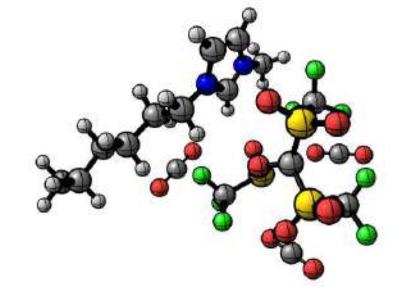	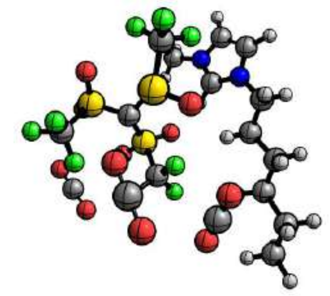	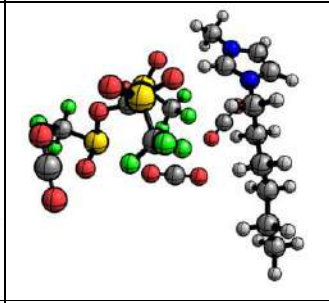	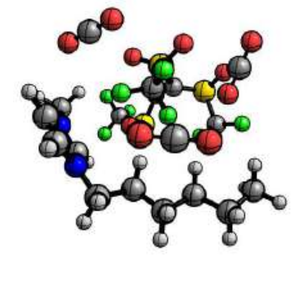	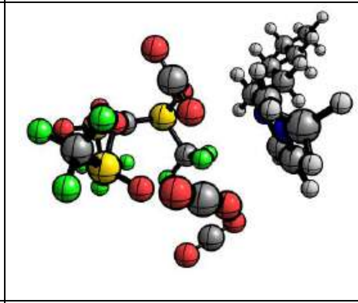
4.13	4.33	4.56	4.61	4.75
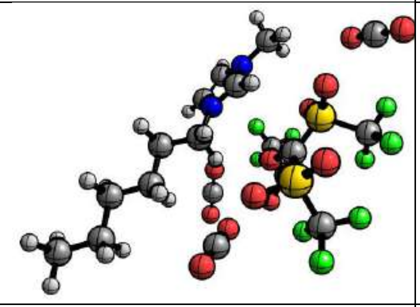	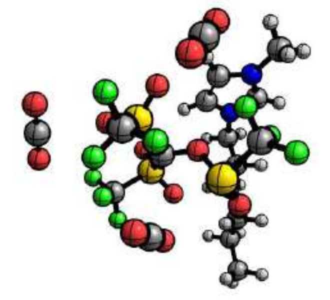	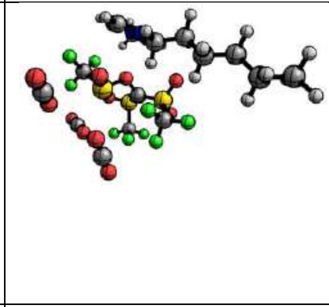	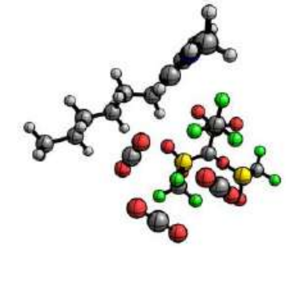	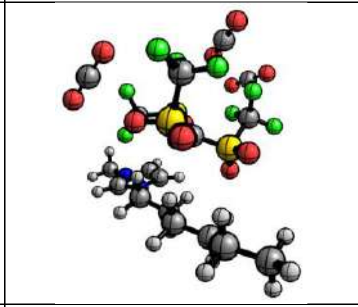
4.95	5.05	5.13	5.24	5.36

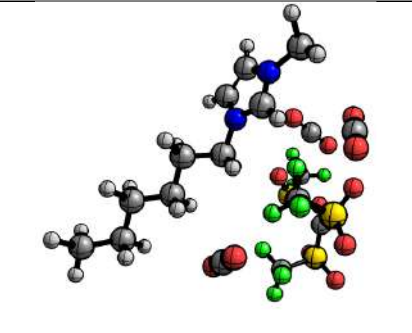	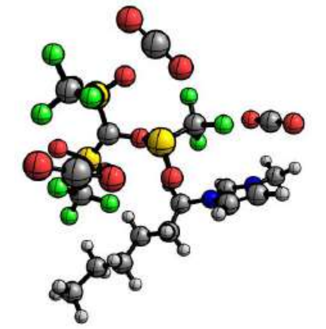	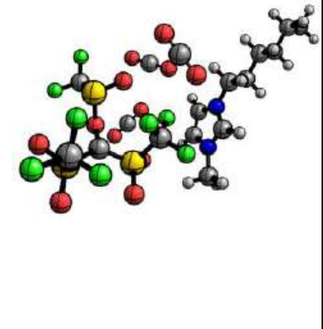	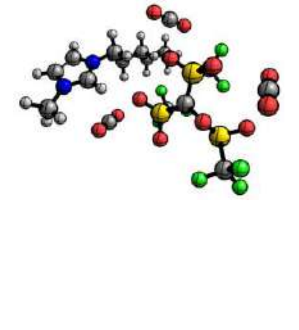	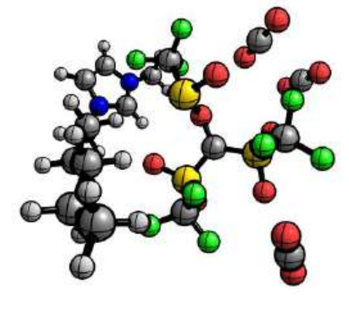
5.37	5.38	5.48	6.50	7.11
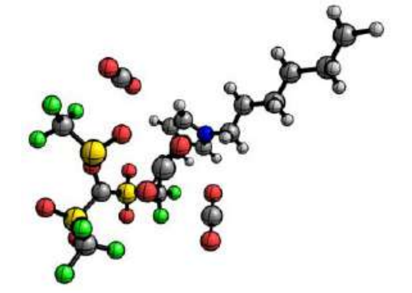	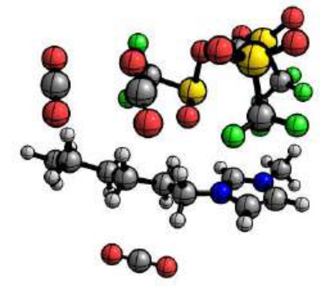	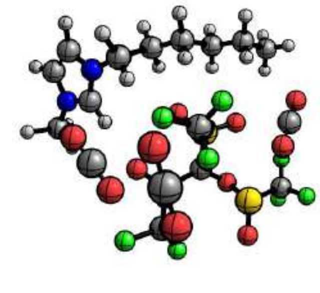	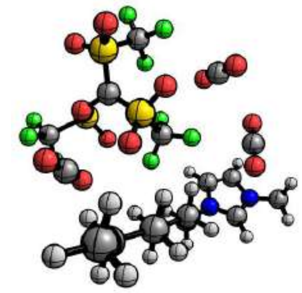	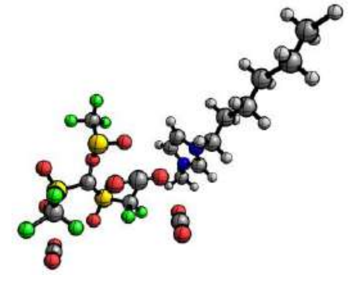
7.58	7.72	7.90	8.11	9.48
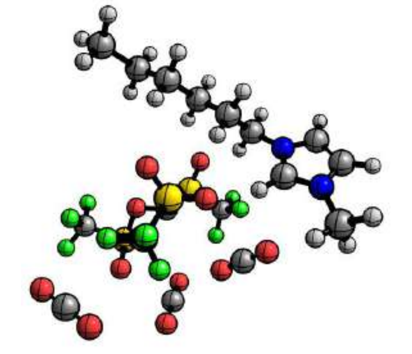	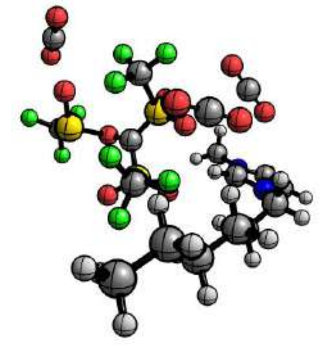	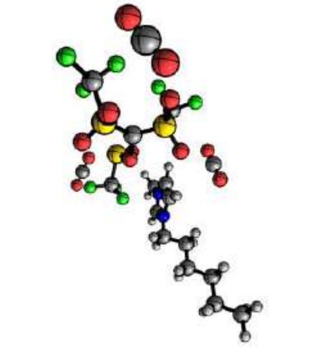	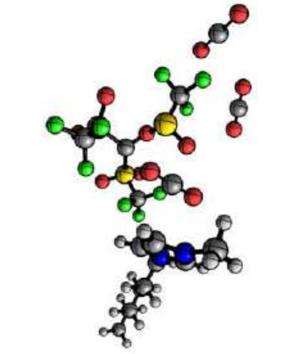	
9.87	9.89	12.38	12.86	

Table S41. Representations of lowest energy forms for $4CO_2[Hmim]^+[Methide]^-$ at the M06-2X-D3/6-31G(d,p) level with an implicit solvent model PCM. Relative energies are listed in kcal/mol.

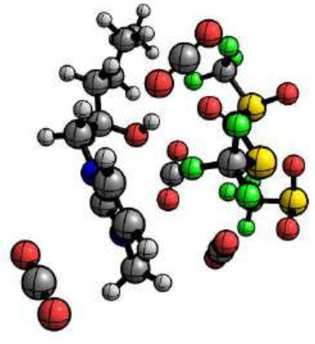	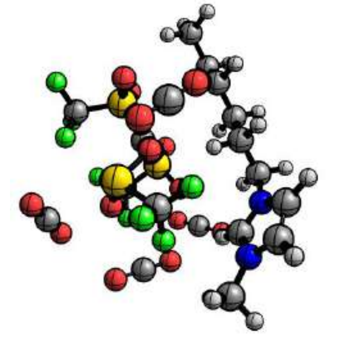	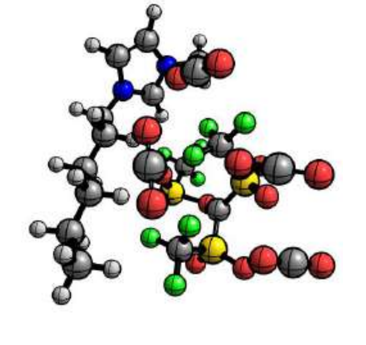	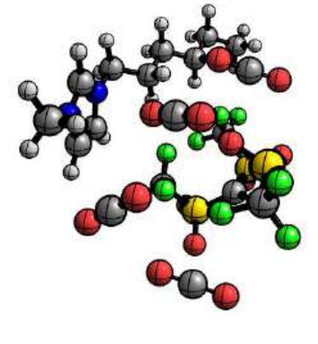	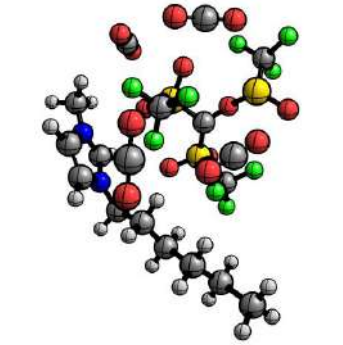
0.00	48.39	48.63	49.55	50.96
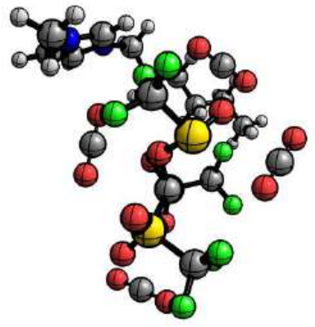	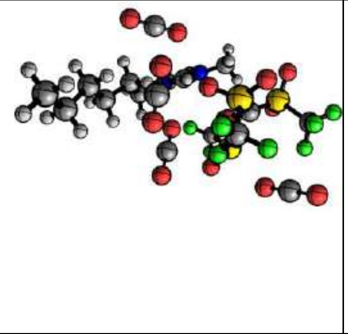	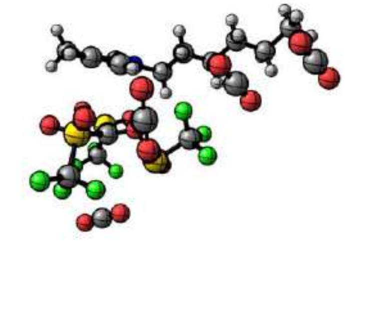	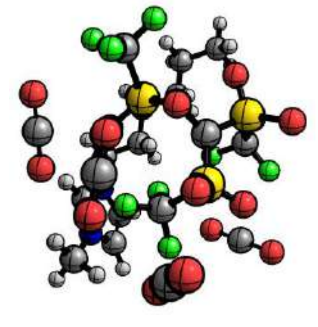	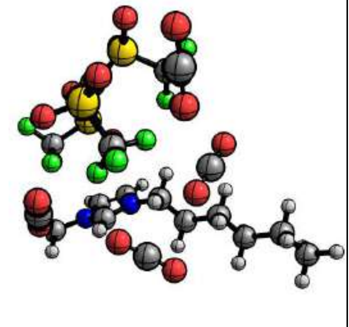
51.18	51.28	51.46	51.85	52.00
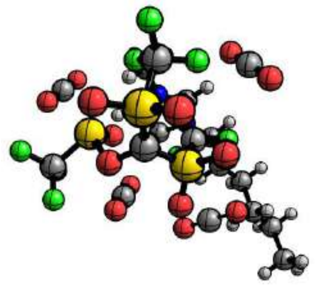	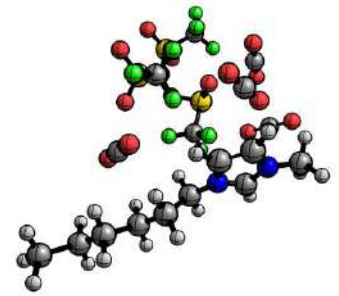	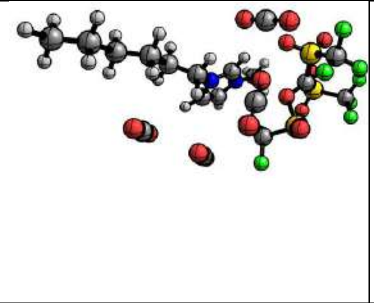	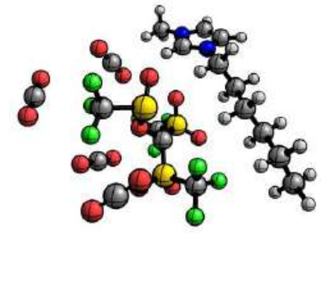	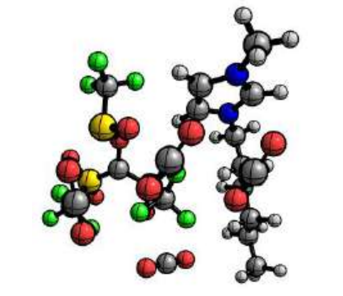
52.28	53.22	53.23	53.76	54.01

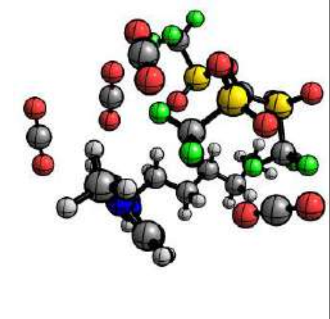	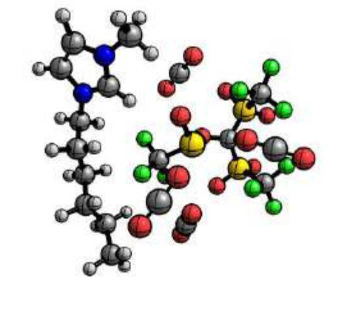	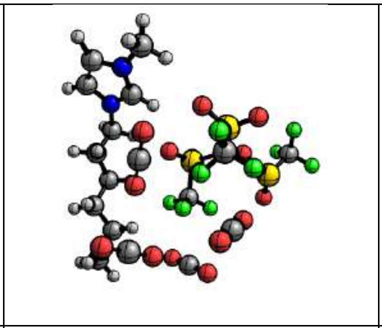	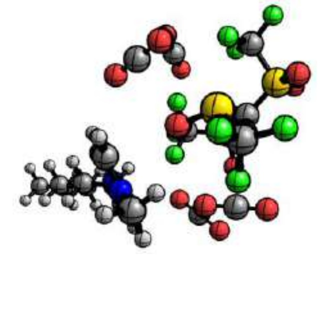	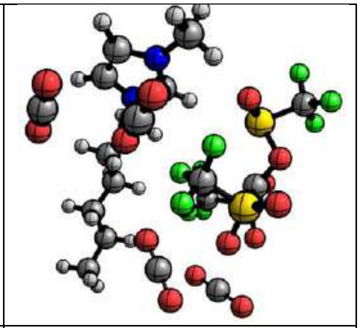
54.33	55.16	55.33	55.40	55.46
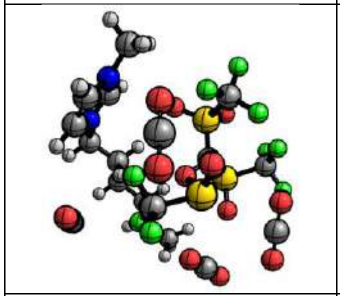	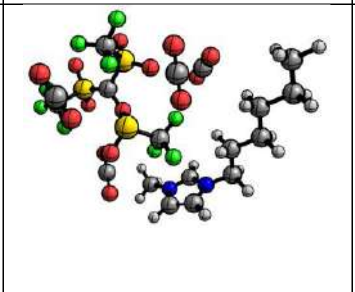	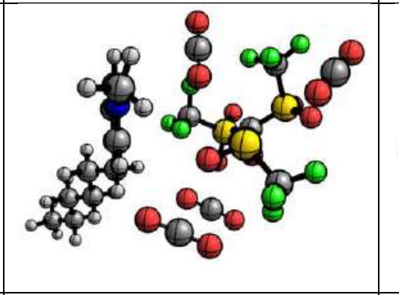	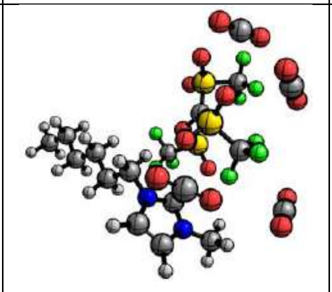	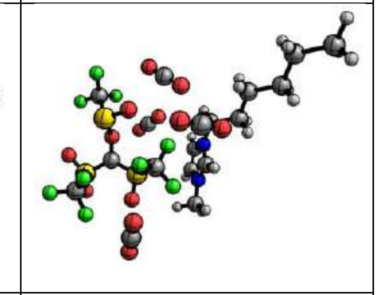
55.49	55.67	55.87	55.90	56.03
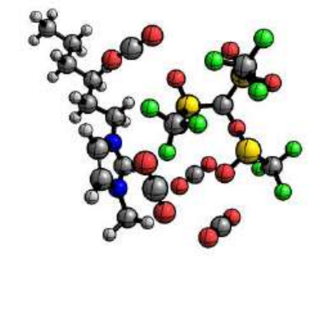	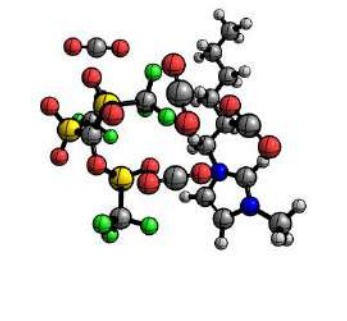	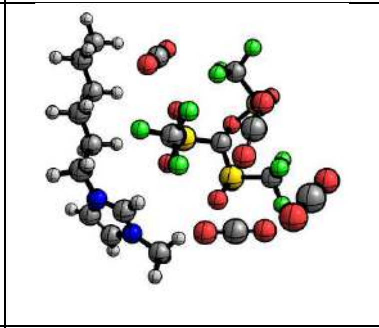	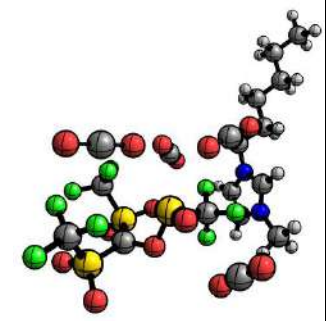	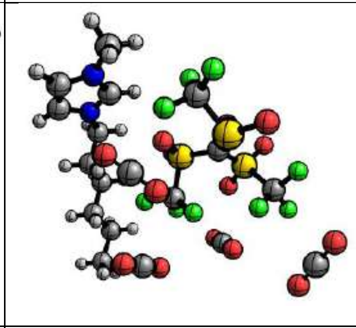
56.38	56.51	57.11	57.23	58.05

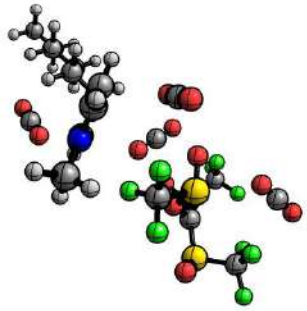	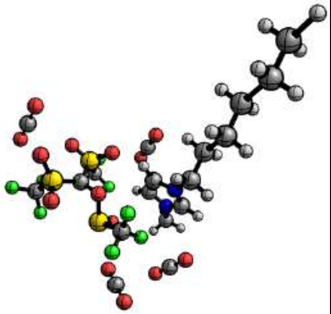	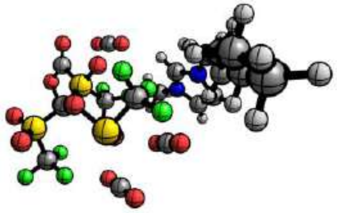		
58.16	59.56	63.33		

Table S42. Representations of lowest energy forms for $5CO_2[Hmim]^+[Methide]^-$ at the M06-2X-D3/6-31G(d,p) level with an implicit solvent model PCM. Relative energies are listed in kcal/mol.

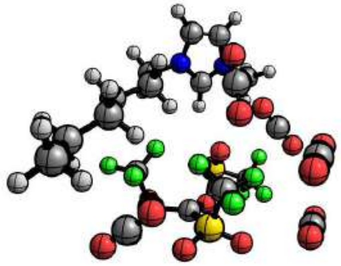	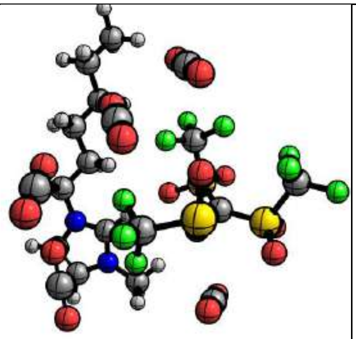	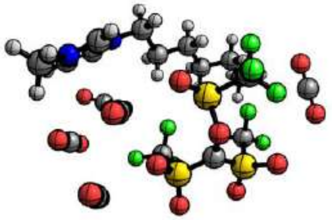	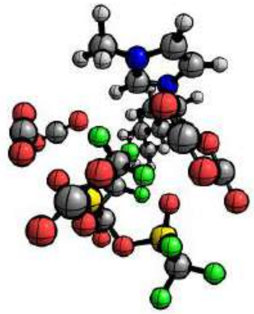	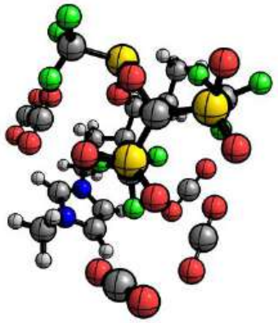
0.00	0.23	0.95	1.25	1.32

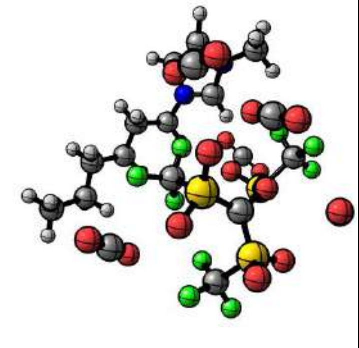	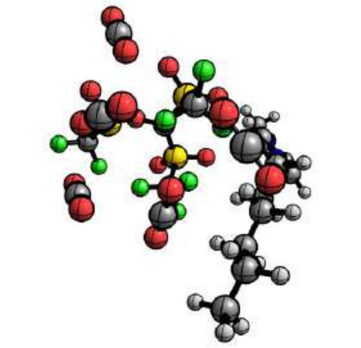	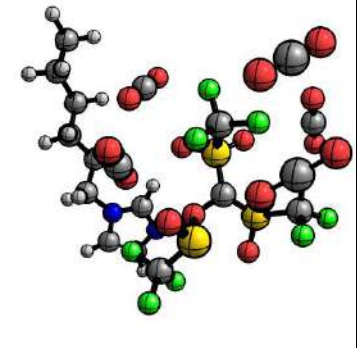	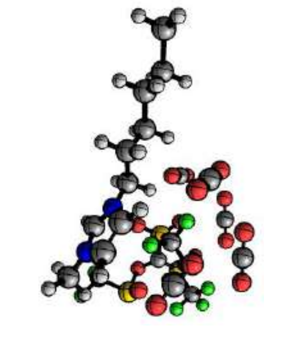	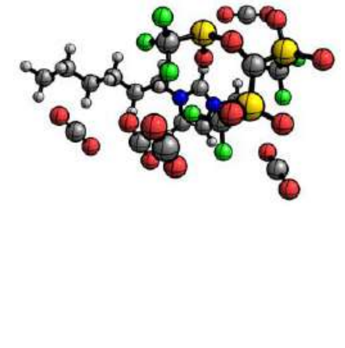
2.97	3.70	3.72	4.04	4.09
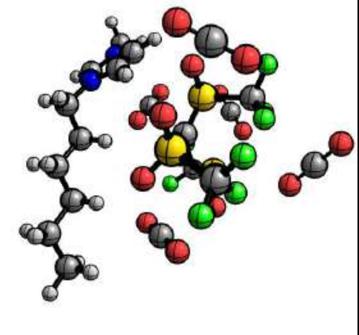	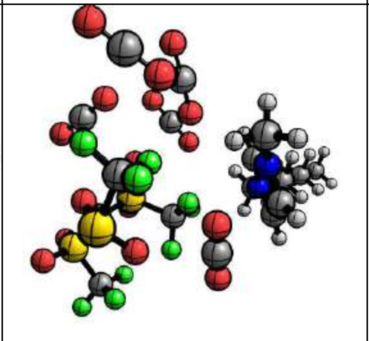	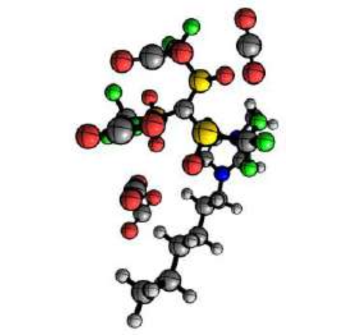	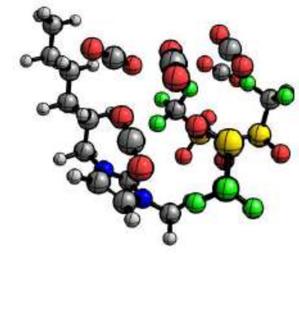	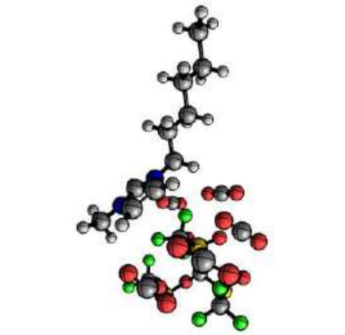
4.26	4.55	4.56	4.67	4.87
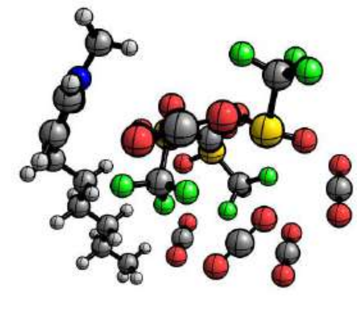	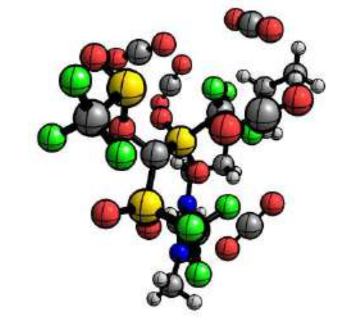	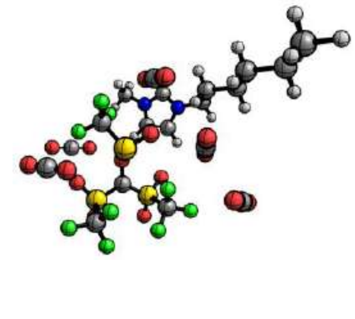	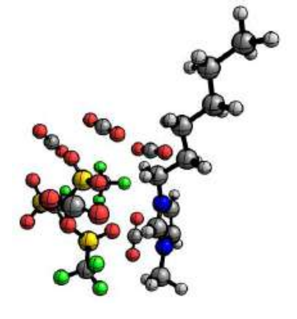	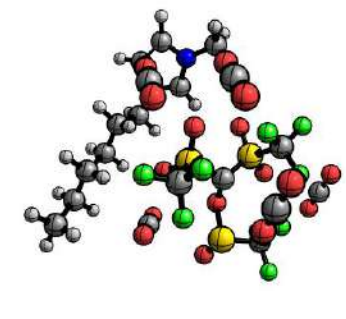
5.53	5.77	5.85	5.86	5.90

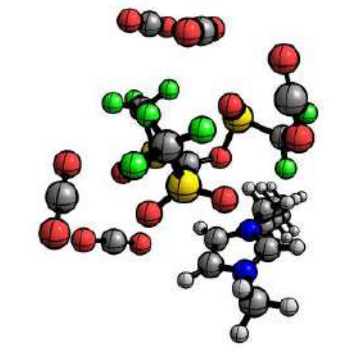	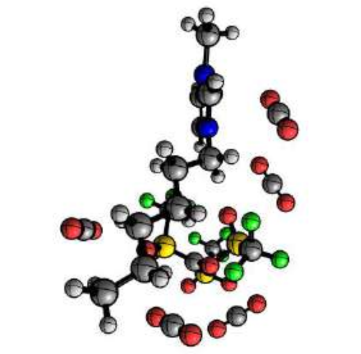	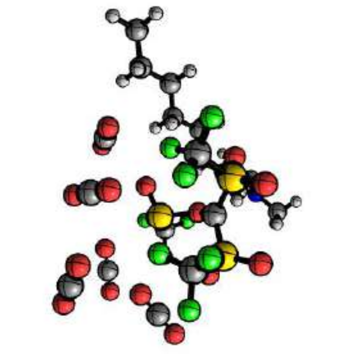	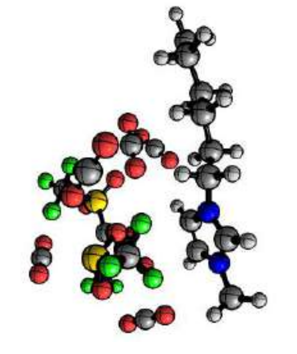	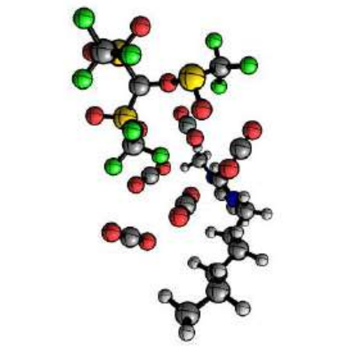
6.28	6.54	6.93	7.33	7.37
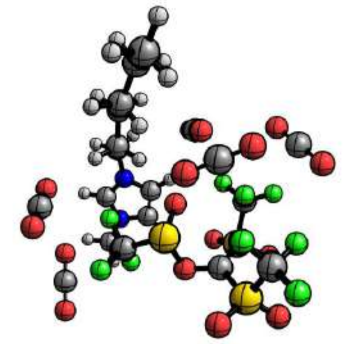	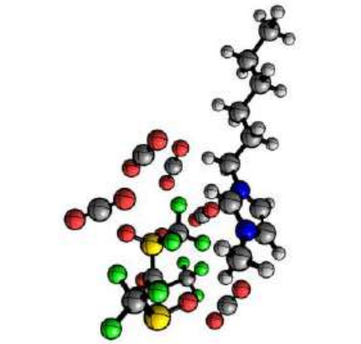	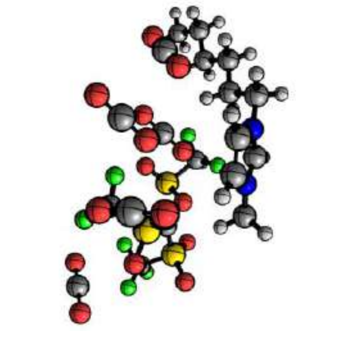	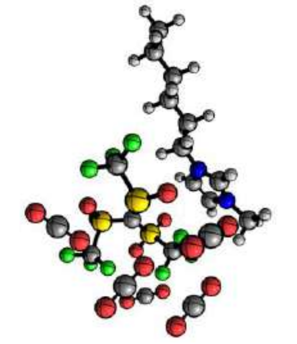	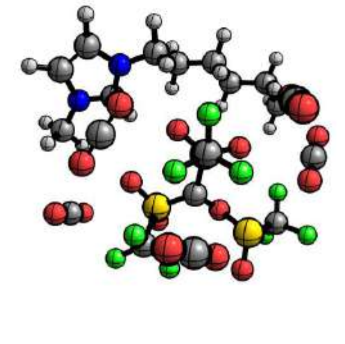
7.38	7.48	7.85	8.10	8.29
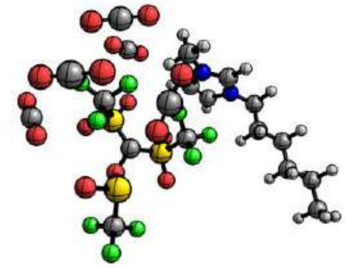	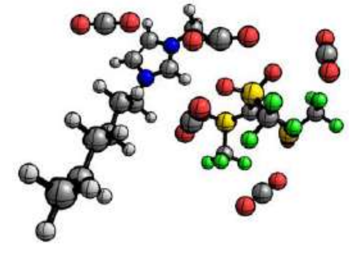	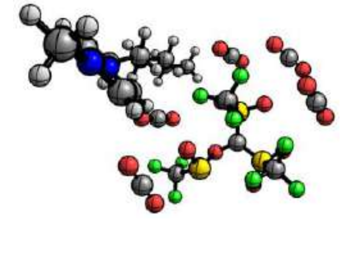	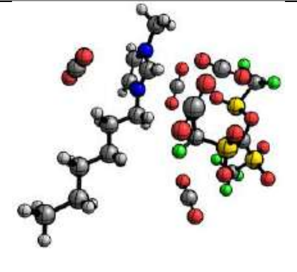	
9.19	9.89	10.23	11.08	

Table S43. Representations of lowest energy forms for $1CO_2[Hmim]^+[(PFOc)SO_3]^-$ at the M06-2X-D3/6-31G(d,p) level with an implicit solvent model PCM. Relative energies are listed in kcal/mol.

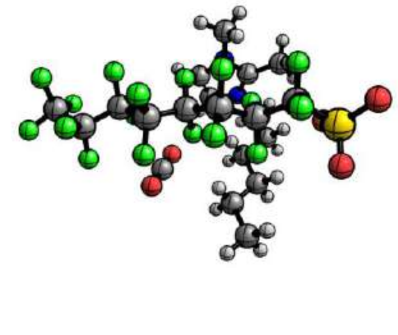	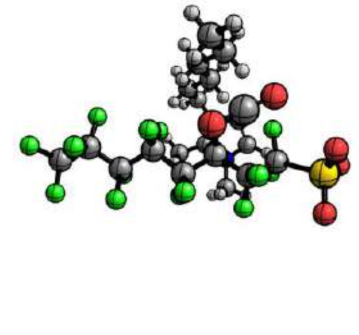	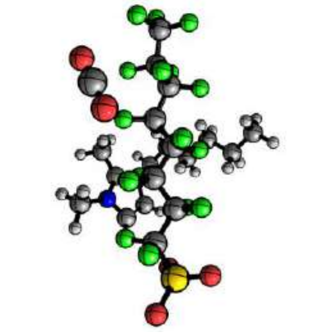	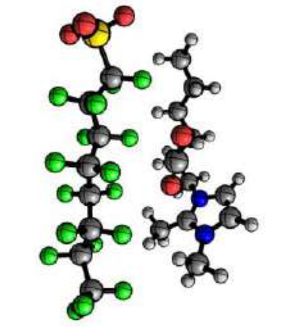	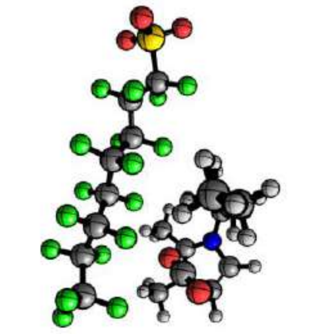
0.00	0.04	1.51	2.17	3.38
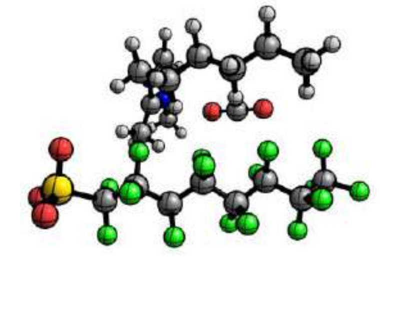	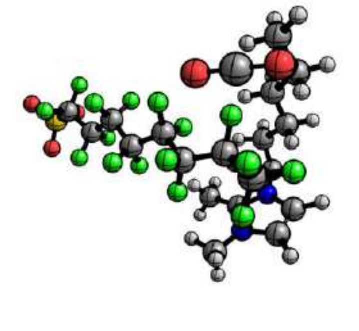	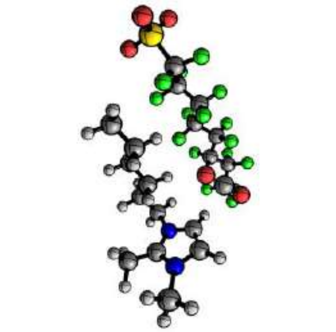	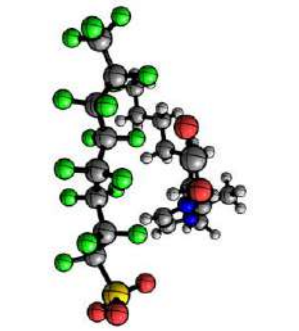	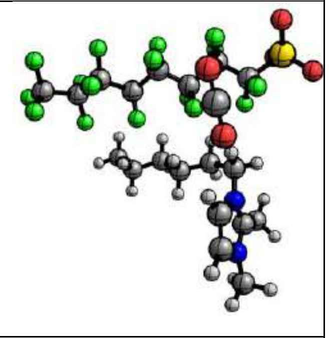
3.90	4.02	4.29	5.39	6.02
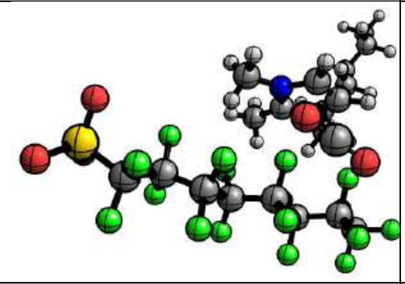	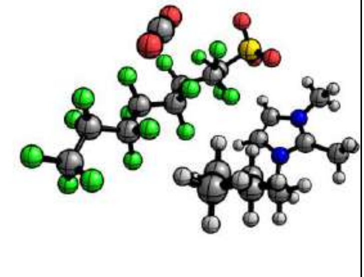	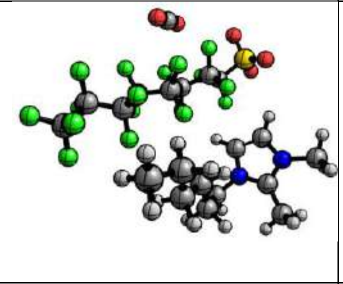	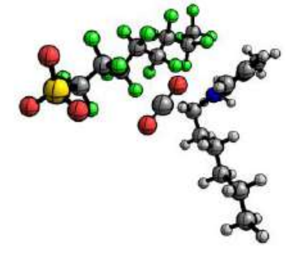	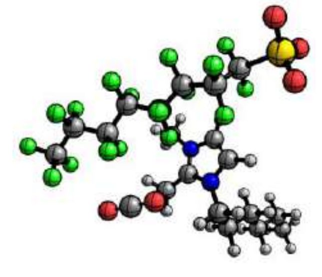
6.28	6.88	7.01	7.22	7.25

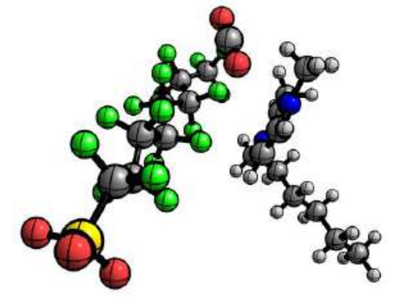	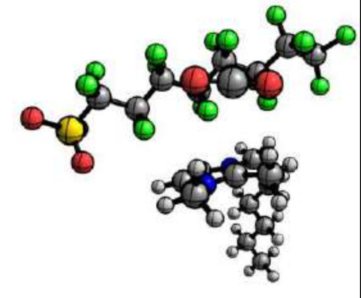	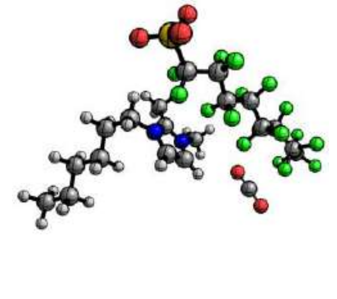	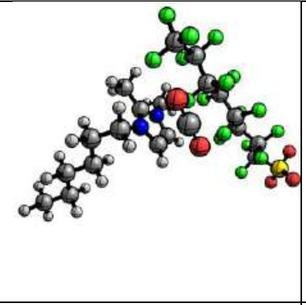	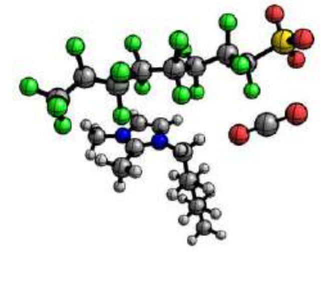
7.26	7.34	7.55	7.66	7.70
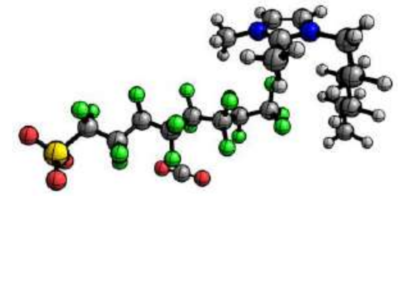	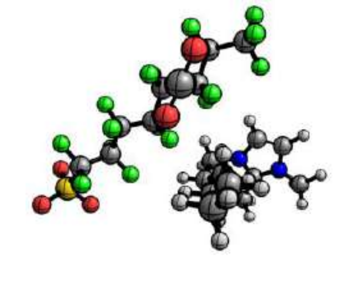	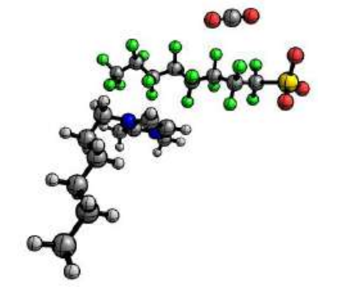	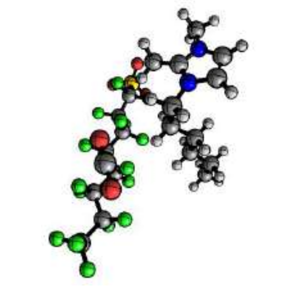	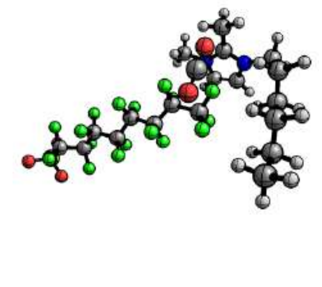
7.72	8.23	8.37	8.48	8.71
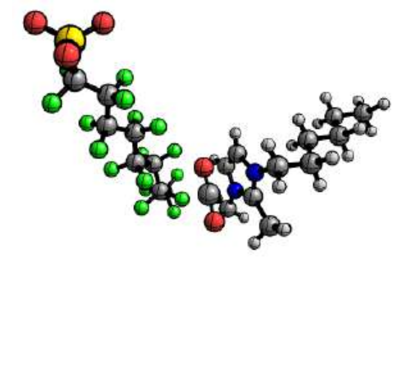	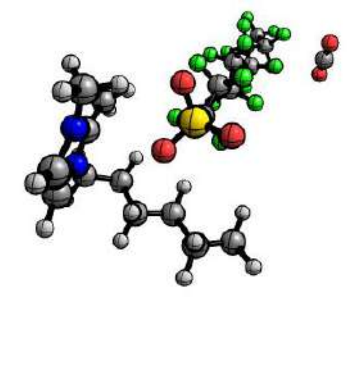	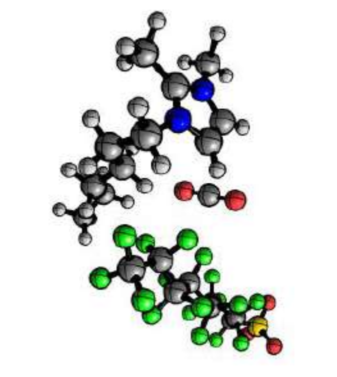	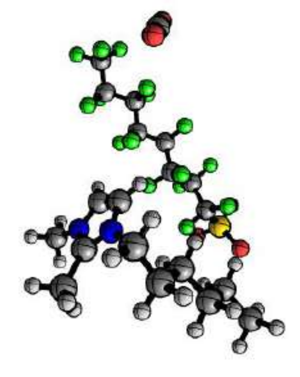	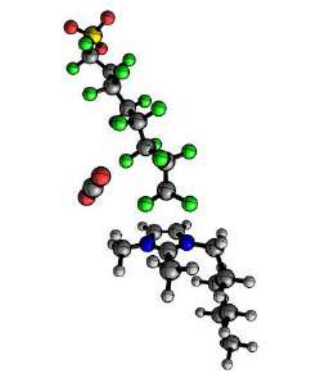
9.19	10.43	10.53	10.62	11.10

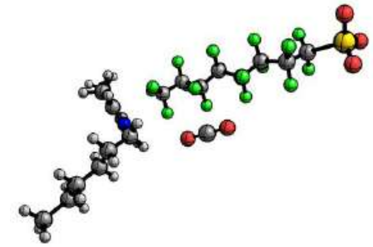	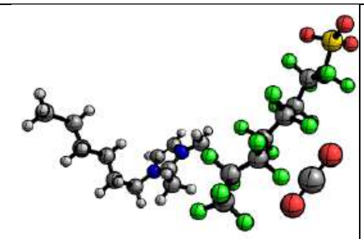	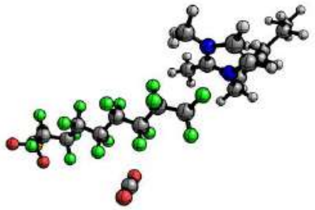		
12.99	13.68	14.05		

Table S44. Representations of lowest energy forms for $2CO_2[Hmim]^+[(PFOc)SO_3]^-$ at the M06-2X-D3/6-31G(d,p) level with an implicit solvent model PCM. Relative energies are listed in kcal/mol.

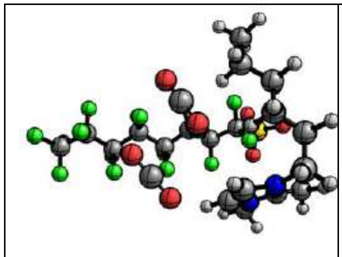	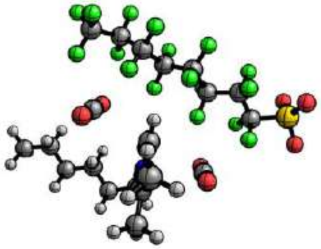	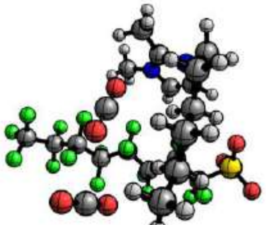	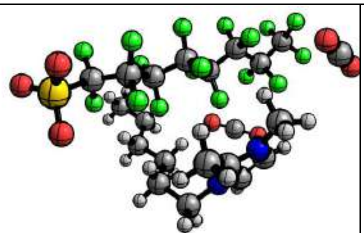	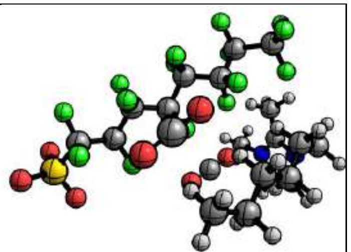
0.00	0.78	1.10	3.18	3.19
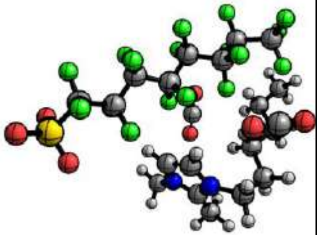	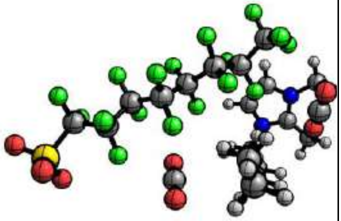	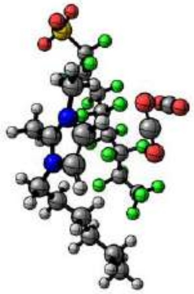	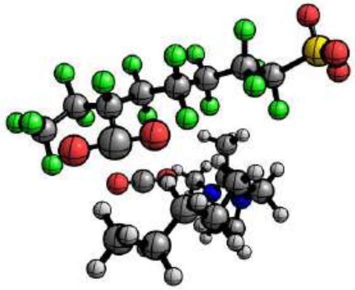	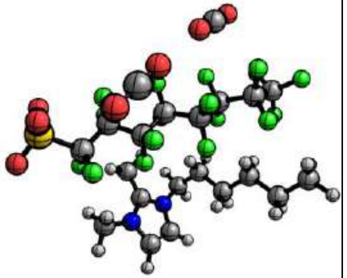
3.78	4.70	4.73	4.84	5.58

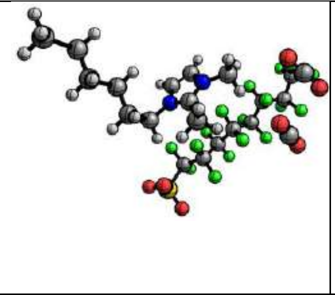	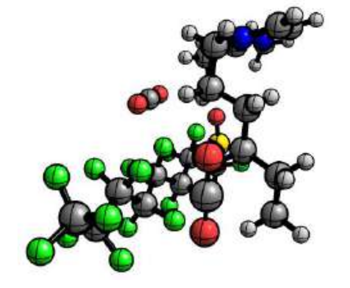	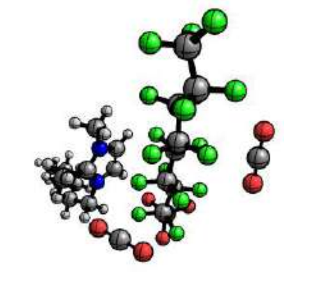	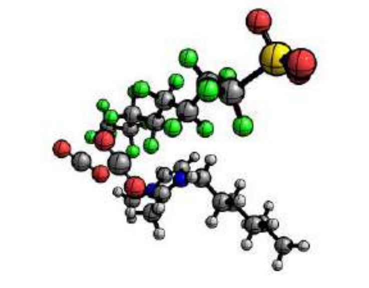	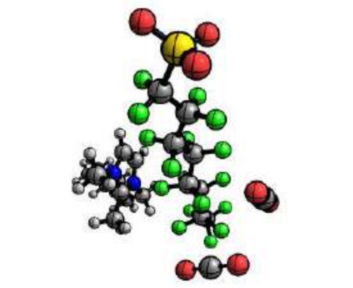
5.90	6.17	6.53	7.04	7.09
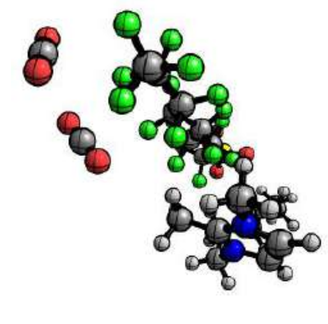	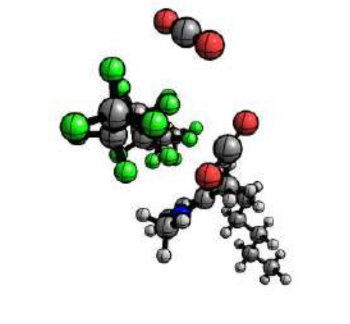	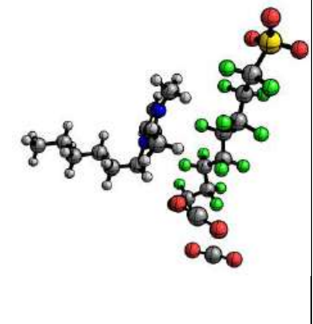	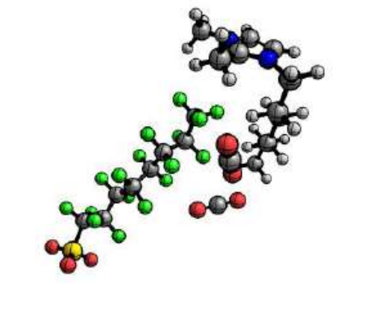	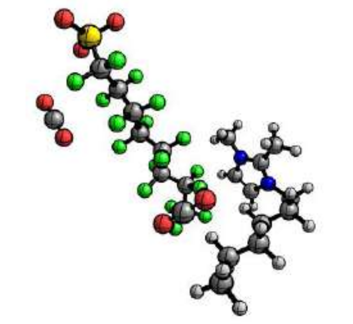
7.44	7.53	7.87	8.01	8.17
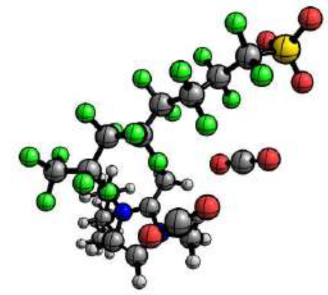	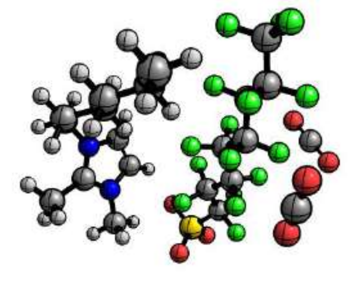	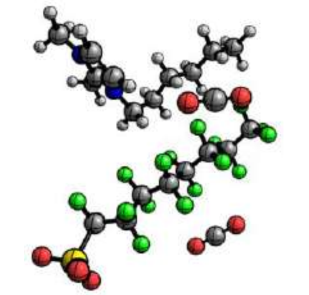	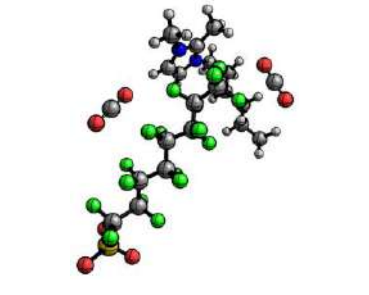	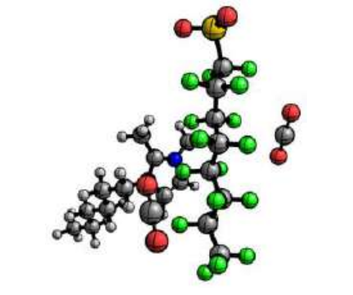
8.21	8.28	8.35	8.48	8.95

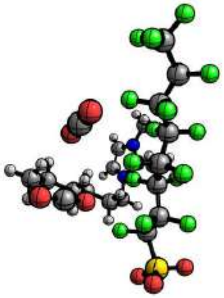	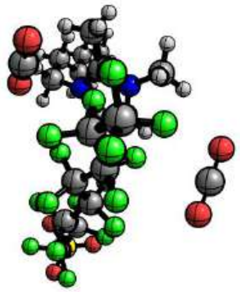	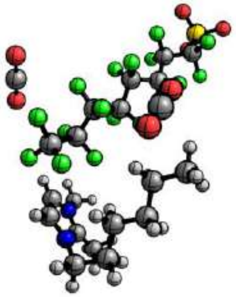	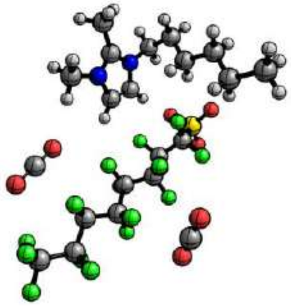	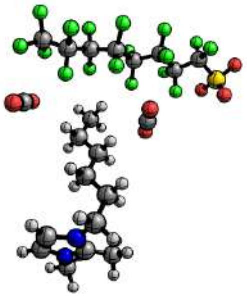
9.10	9.66	10.15	11.58	11.67
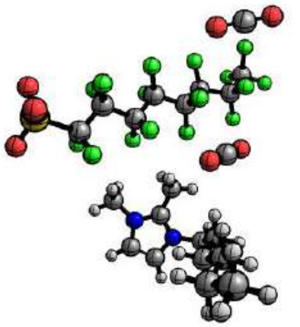				
13.10				

Table S45. Representations of lowest energy forms for $3CO_2[Hmim]^+[(PFOc)SO_3]^-$at the M06-2X-D3/6-31G(d,p) level with an implicit solvent model PCM. Relative energies are listed in kcal/mol.

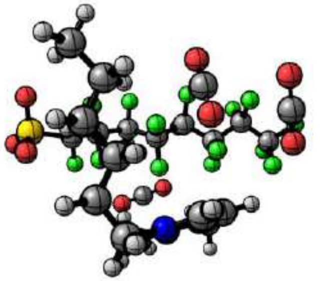	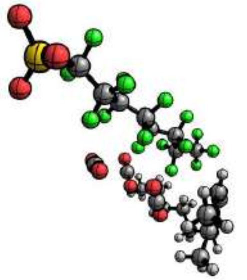	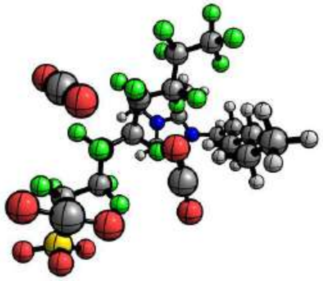	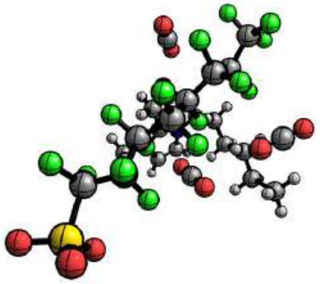	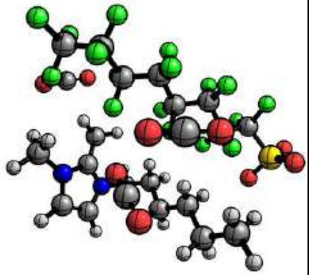

0.00	0.32	0.69	2.02	2.24
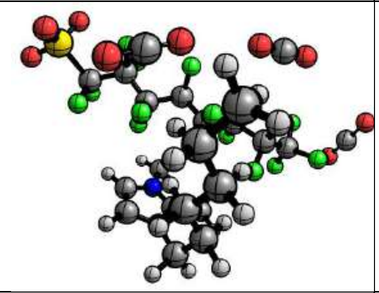	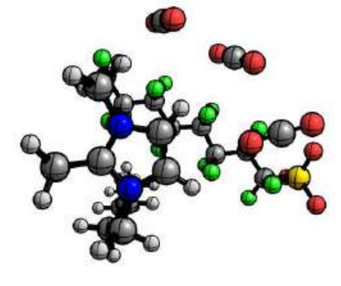	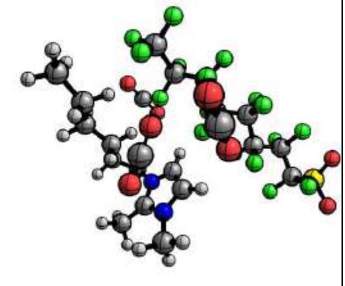	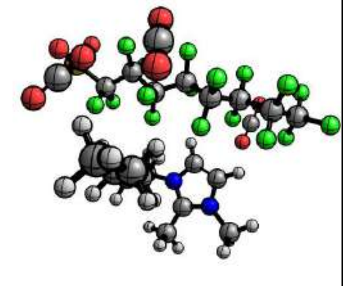	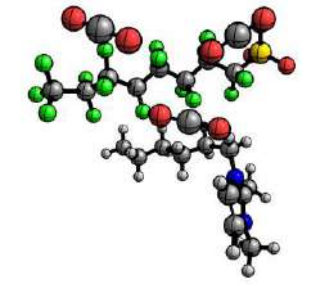
2.82	3.83	4.58	4.61	4.65
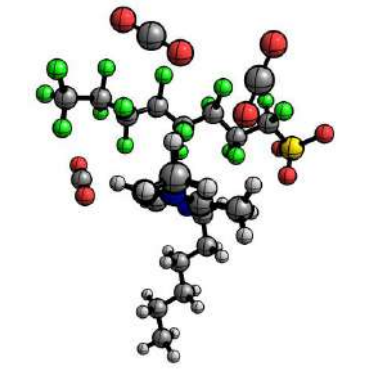	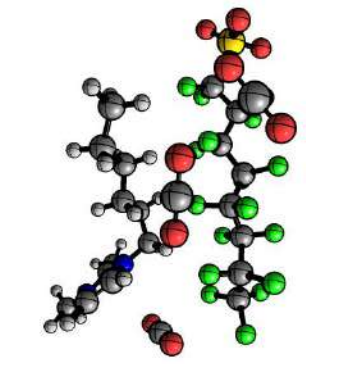	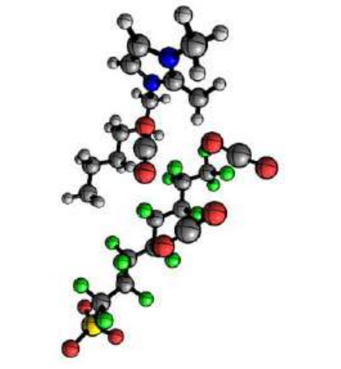	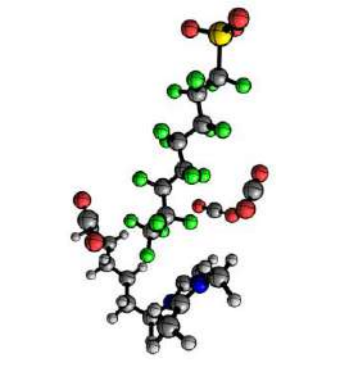	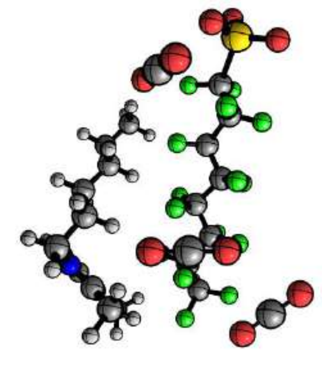
4.67	5.07	5.39	5.96	5.97
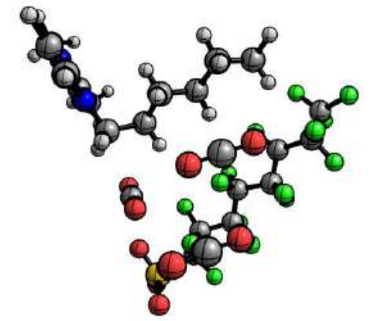	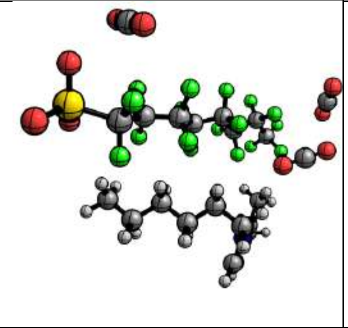	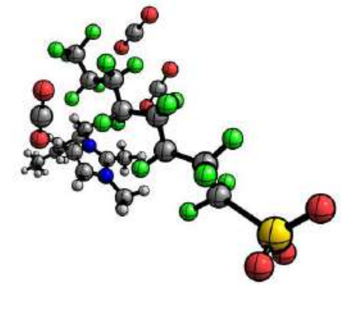	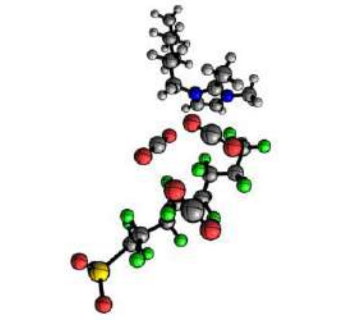	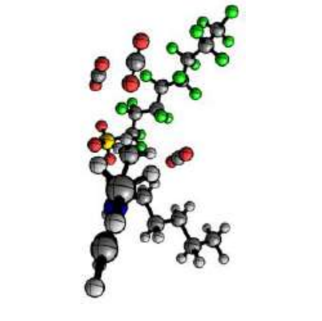
6.82	7.05	7.44	7.70	7.77

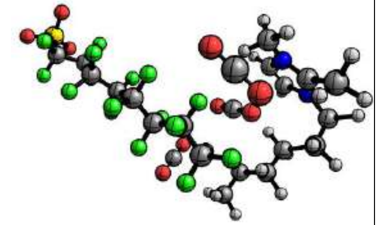	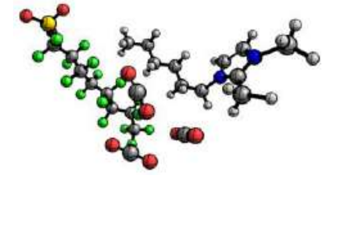	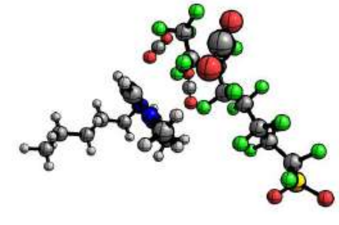	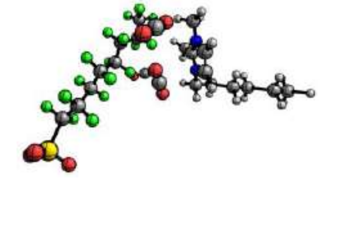	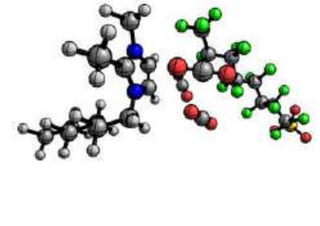
8.13	8.17	8.47	8.65	9.24
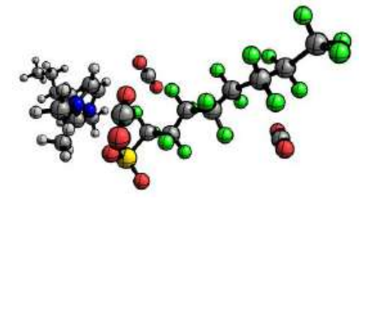	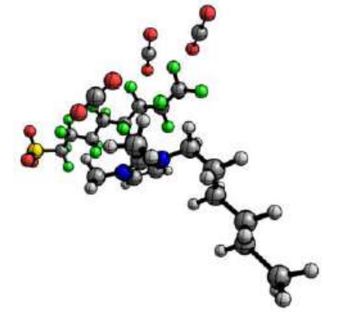	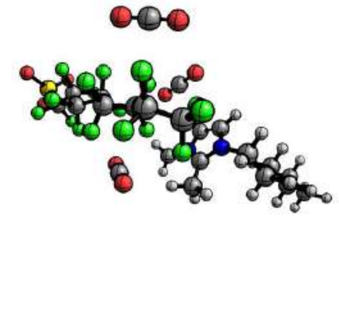	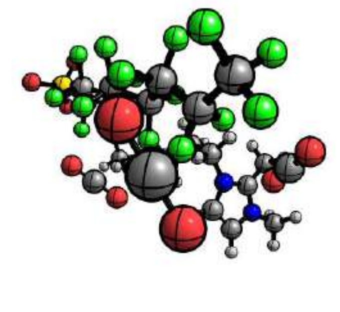	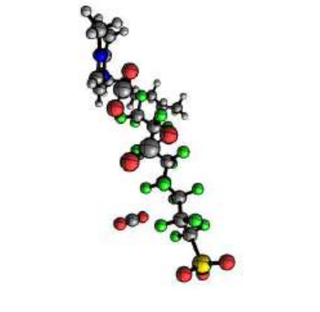
9.57	9.74	9.97	10.23	10.50
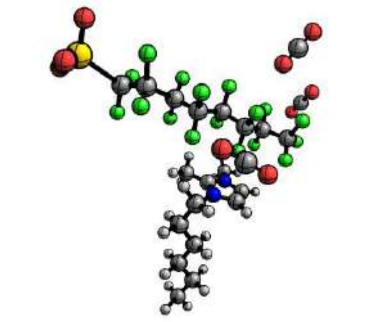	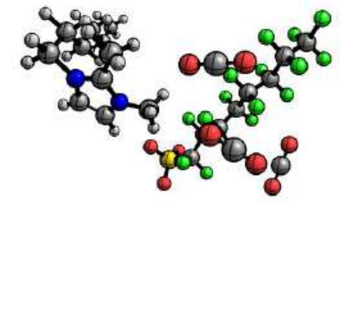			
10.88	12.59			

Table S46. Representations of lowest energy forms for $4CO_2[Hmim]^+[(PFOc)SO_3]^-$ at the M06-2X-D3/6-31G(d,p) level with an implicit solvent model PCM. Relative energies are listed in kcal/mol.

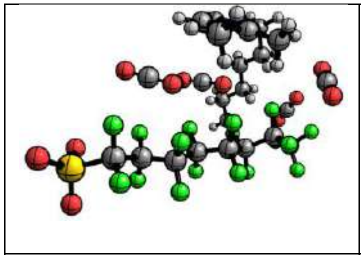	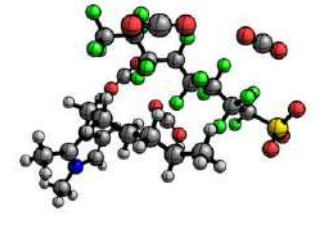	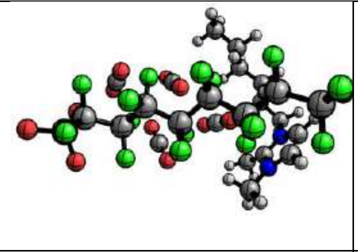	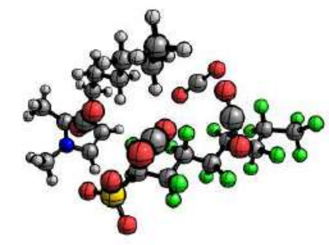	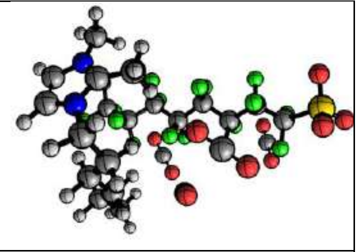
0.00	1.17	2.03	2.88	3.01
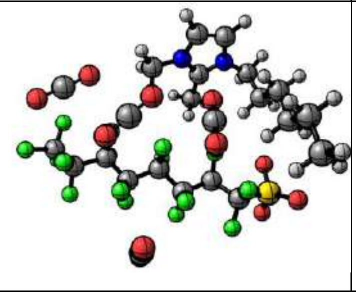	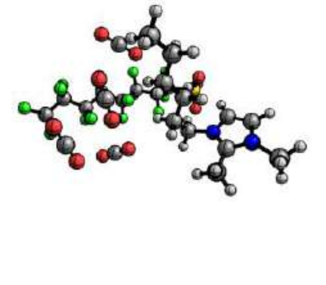	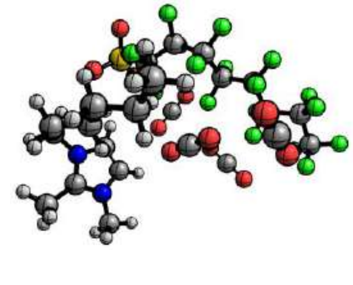	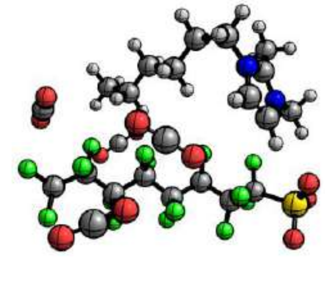	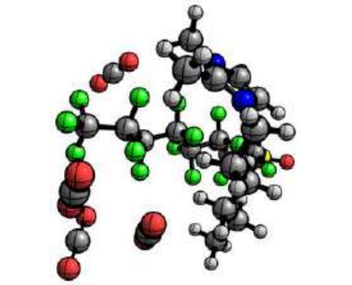
3.13	3.45	3.59	3.64	3.95
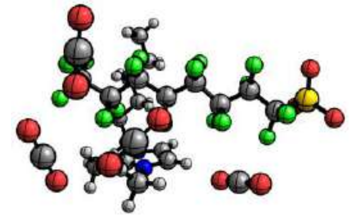	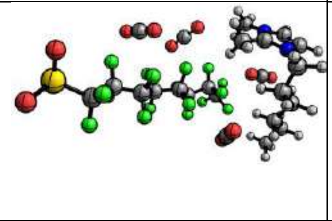	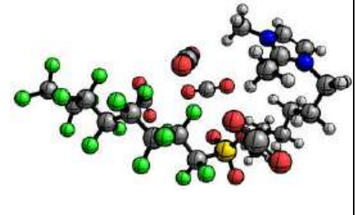	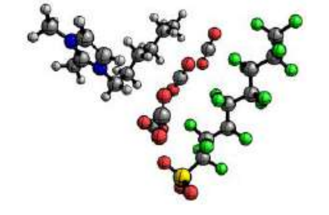	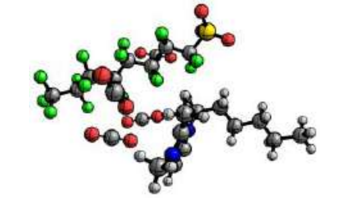
4.30	4.71	5.33	5.56	6.36
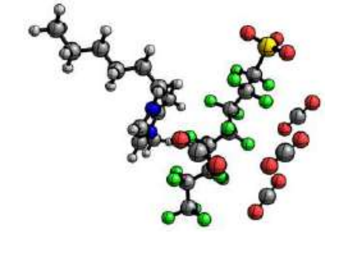	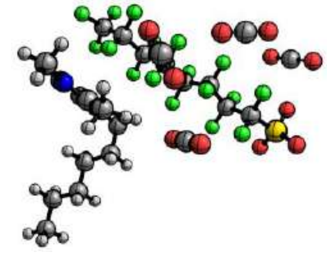	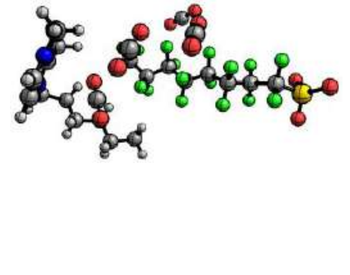	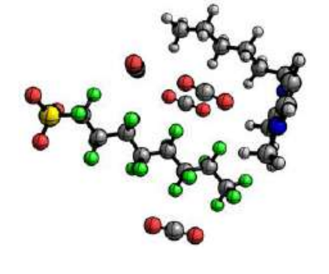	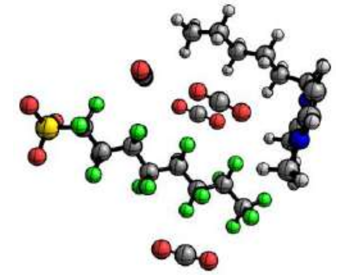
6.46	6.49	6.79	6.88	7.01

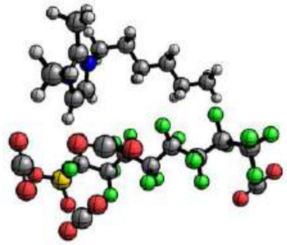	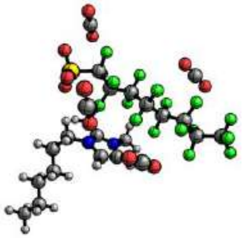	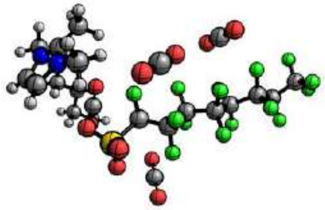	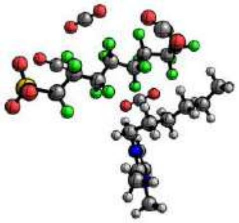	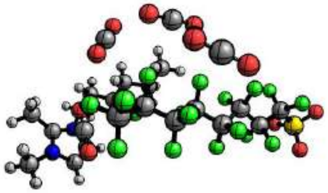
7.02	7.09	7.27	7.43	7.61
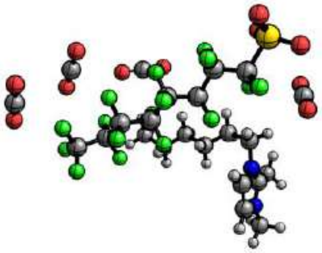	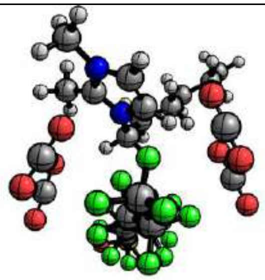	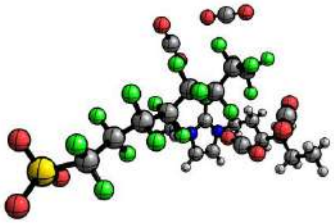	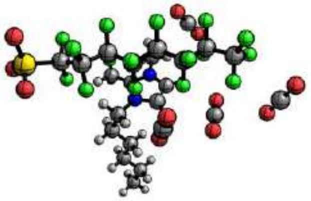	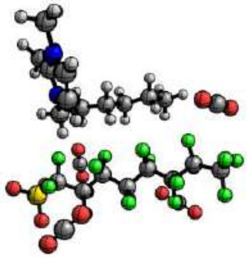
7.83	8.08	8.21	8.34	8.42
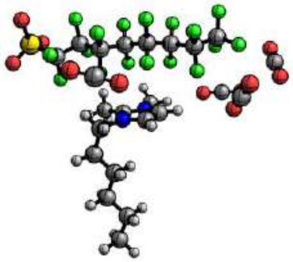	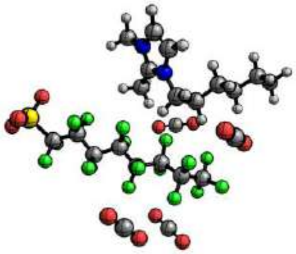	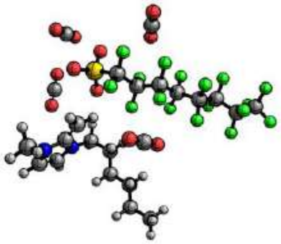		
8.88	9.04	9.27		

Table S47. Representations of lowest energy forms for $5CO_2[Hmim]^+[(PFOc)SO_3]^-$ at the M06-2X-D3/6-31G(d,p) level with an implicit solvent model PCM. Relative energies are listed in kcal/mol.

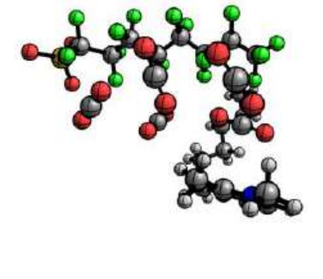	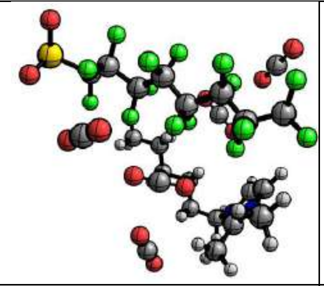	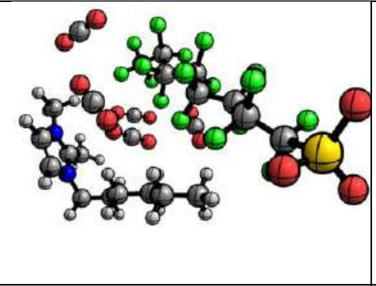	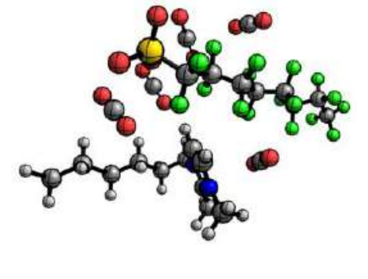	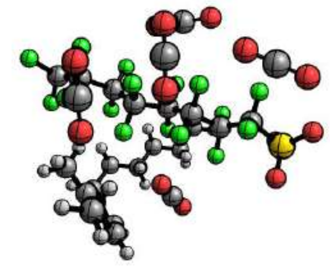
0.00	0.79	1.08	1.73	1.83
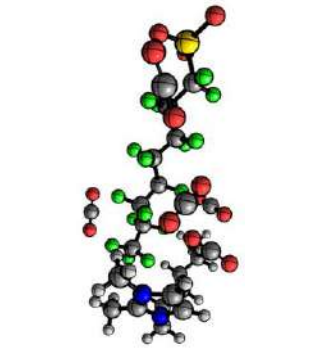	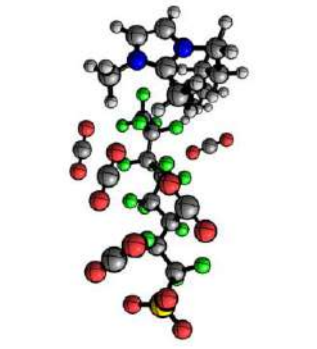	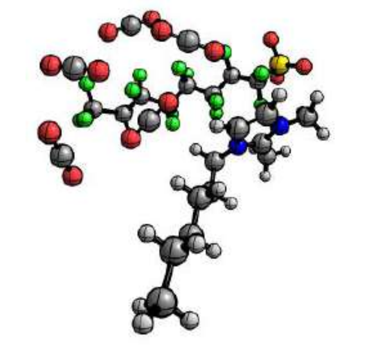	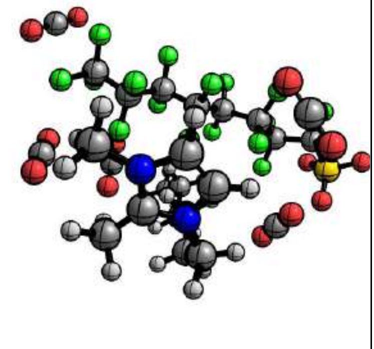	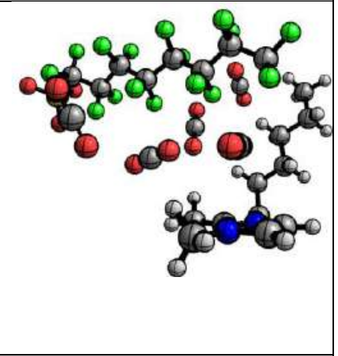
2.61	2.85	3.13	3.44	3.63
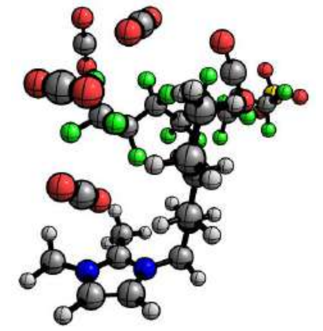	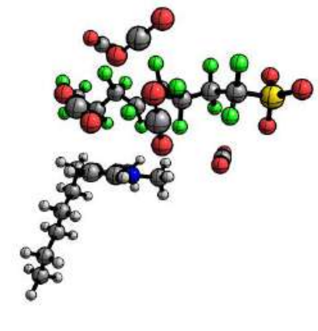	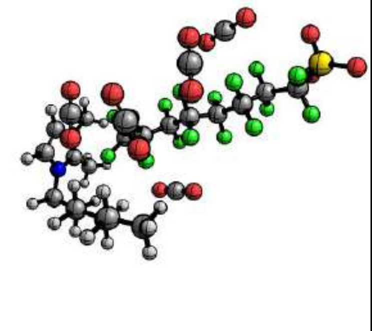	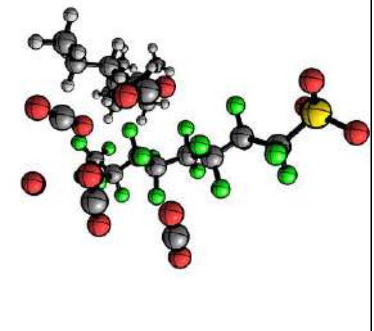	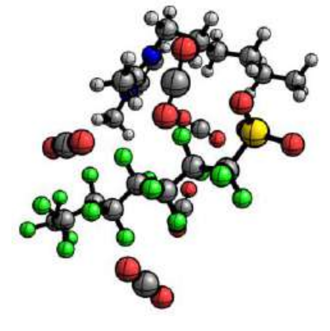
4.68	5.26	5.32	5.72	5.98

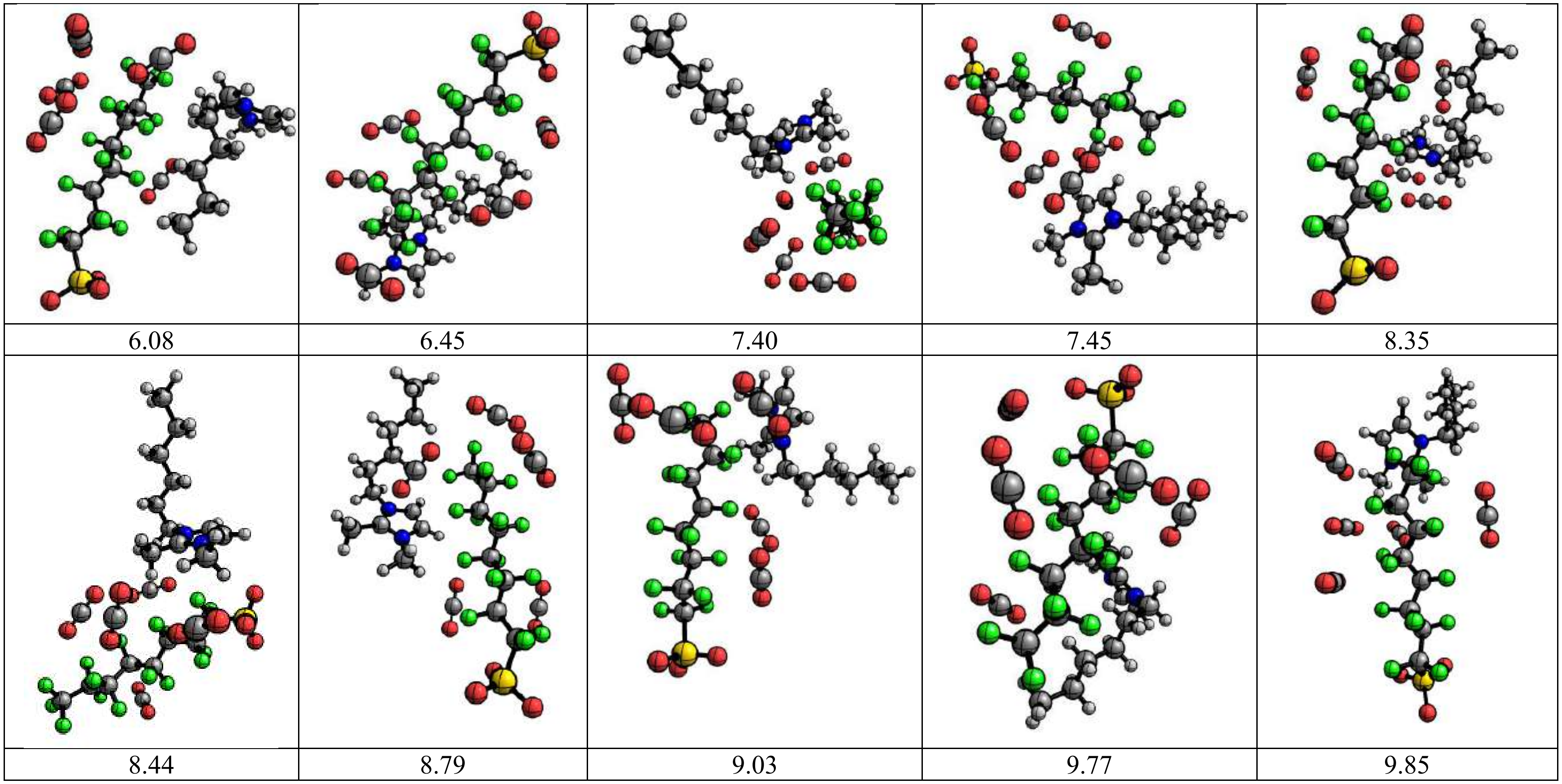
6.08
6.45
7.40
7.45
8.35
8.44
8.79
9.03
9.77
9.85

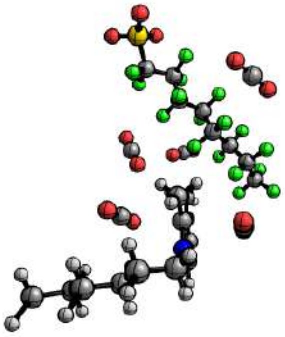	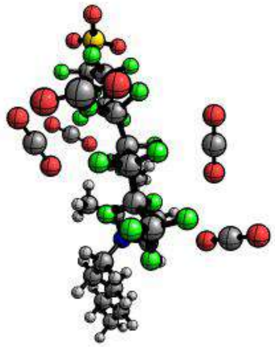	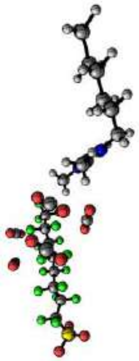		
9.85	11.20	15.72		

Table S48. Representations of lowest energy forms for $1CO_2[Omim]^+[(PFOc)SO_3]^-$at the M06-2X-D3/6-31G(d,p) level with an implicit solvent model PCM. Relative energies are listed in kcal/mol.

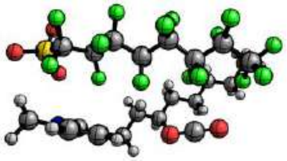	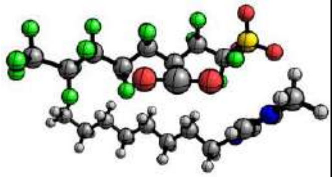	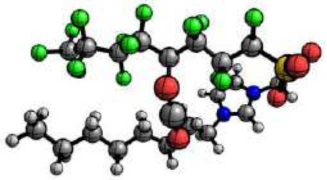	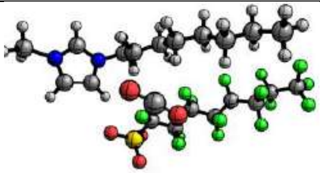	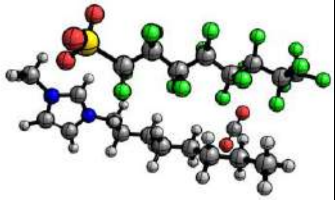
0.00	1.78	2.50	2.94	3.07
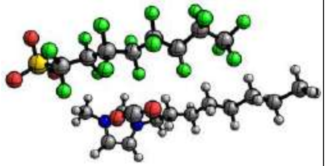	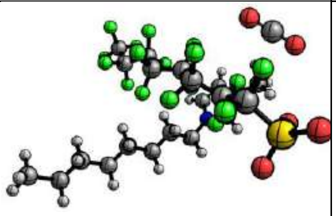	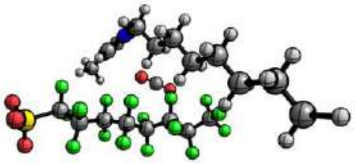	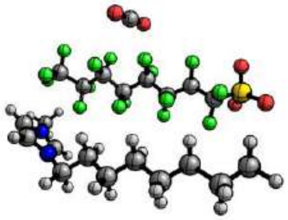	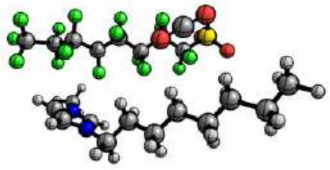
3.25	3.47	3.49	3.57	3.67

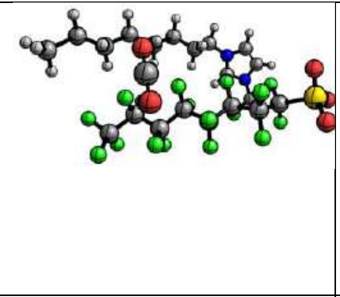	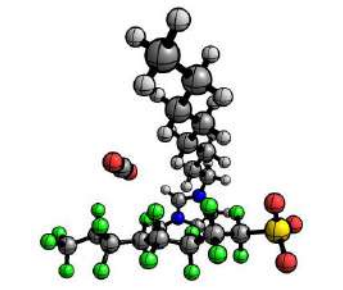	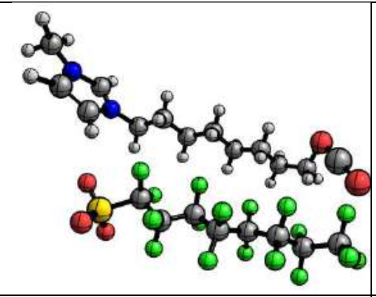	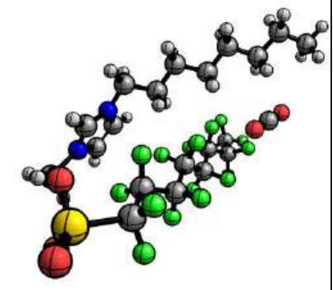	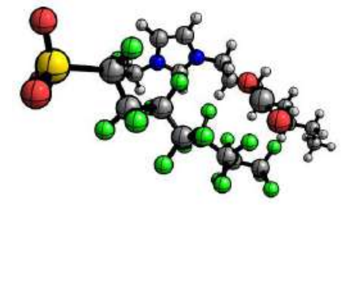
3.94	4.05	4.18	4.68	4.72
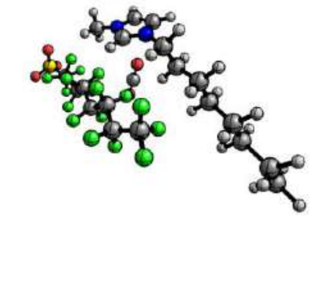	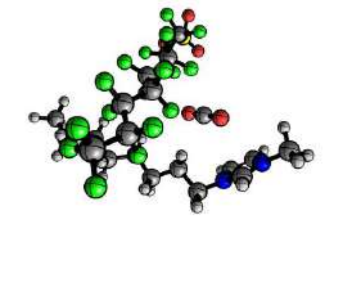	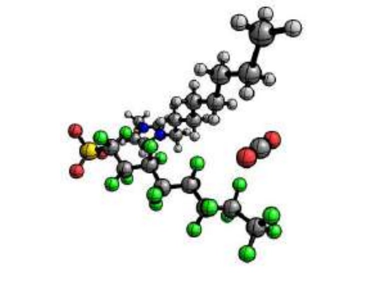	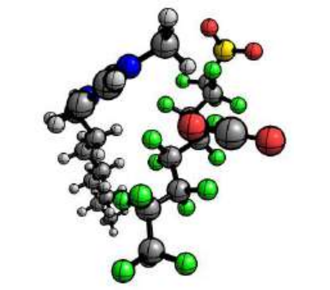	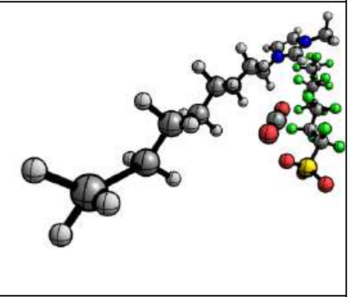
4.88	5.03	5.08	5.37	5.53
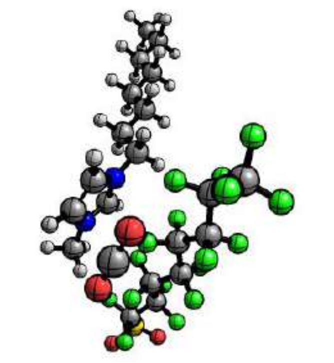	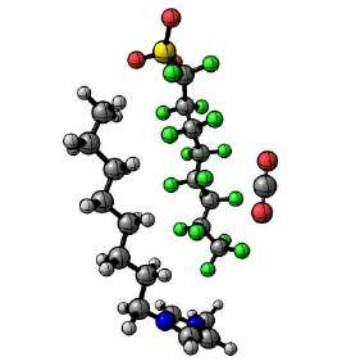	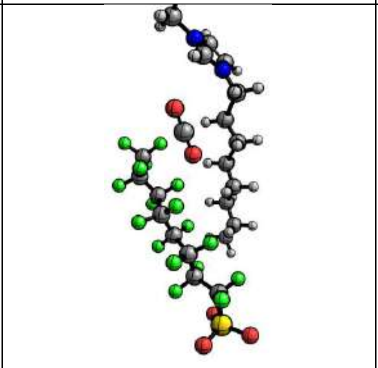	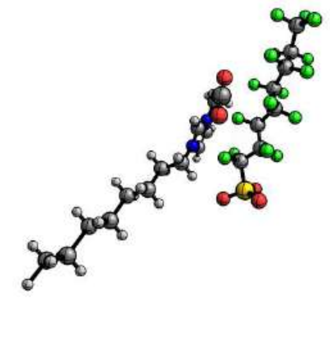	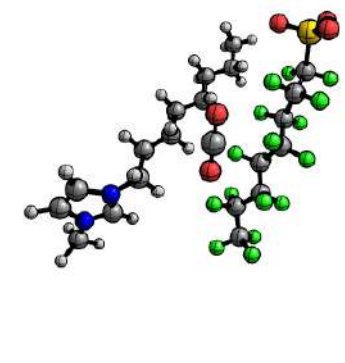
5.60	5.99	6.22	6.77	7.38

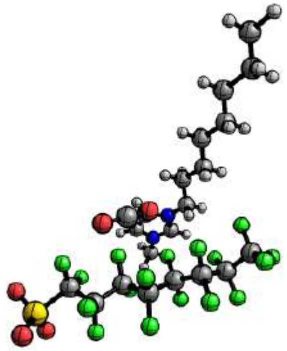	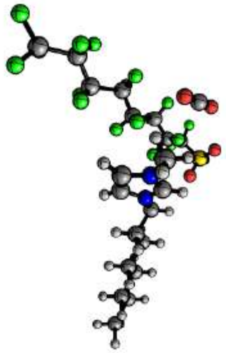	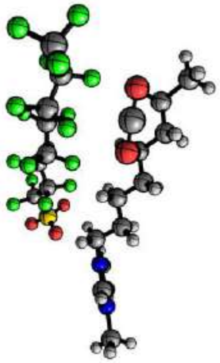	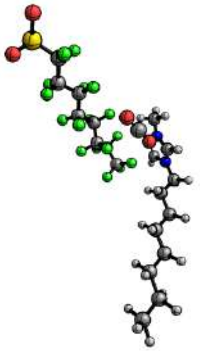	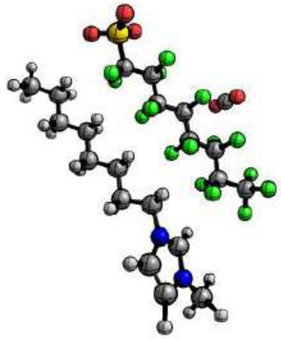
7.39	7.43	7.51	7.55	8.32
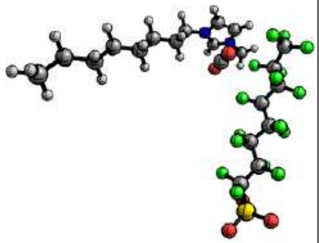	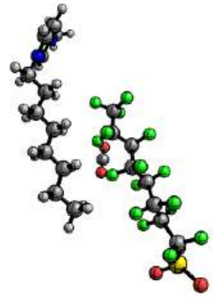	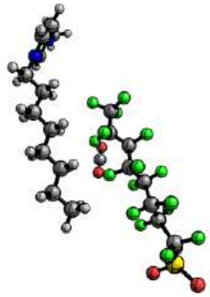		
8.90	9.89	10.02		

Table S49. Representations of lowest energy forms for $2CO_2[Omim]^+[(PFOc)SO_3]^-$at the M06-2X-D3/6-31G(d,p) level with an implicit solvent model PCM. Relative energies are listed in kcal/mol.

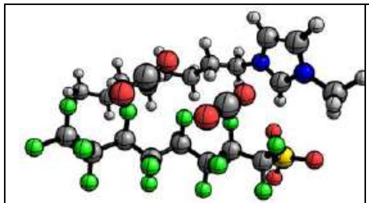	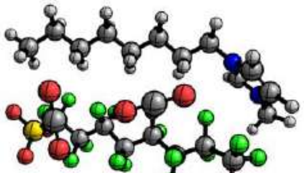	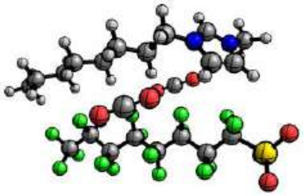	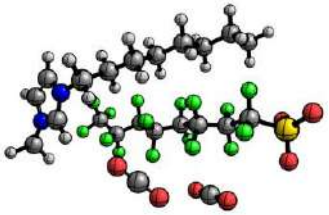	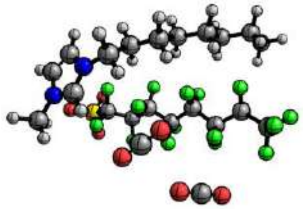
0.00	0.74	1.24	1.97	2.05

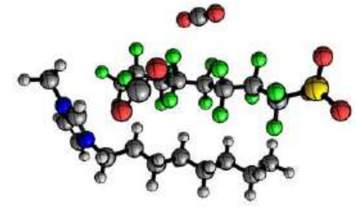	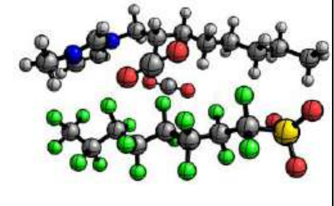	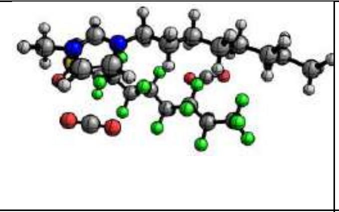	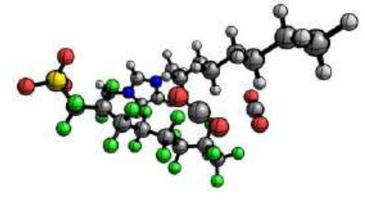	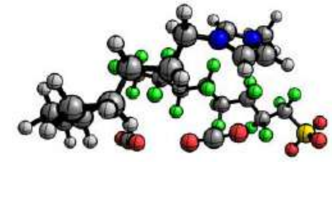
2.16	2.48	3.28	3.31	3.81
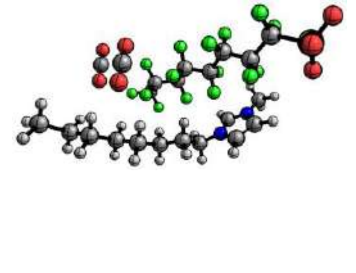	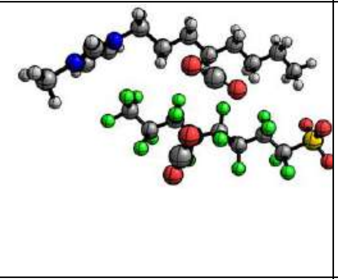	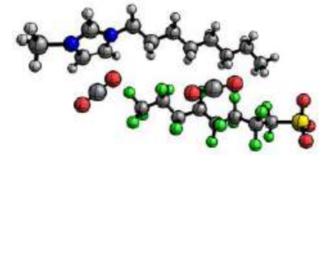	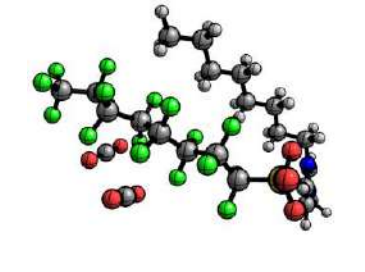	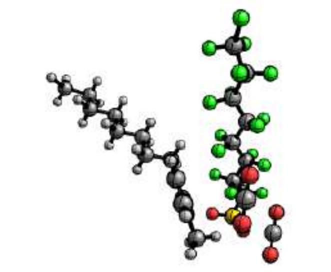
3.89	4.03	4.87	5.31	5.46
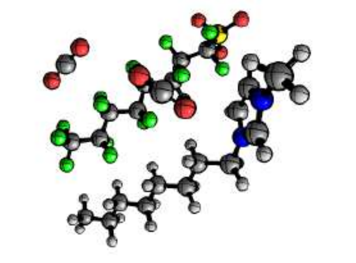	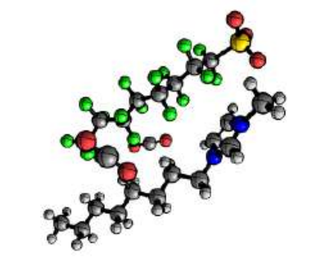	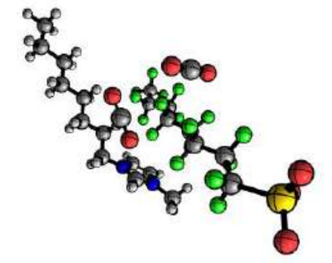	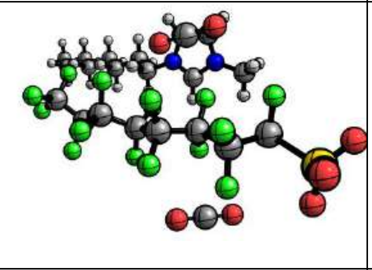	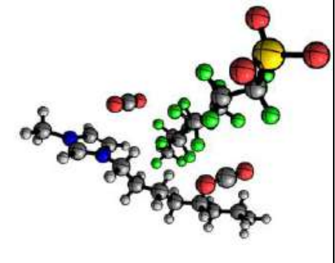
5.54	6.42	6.49	6.97	7.24
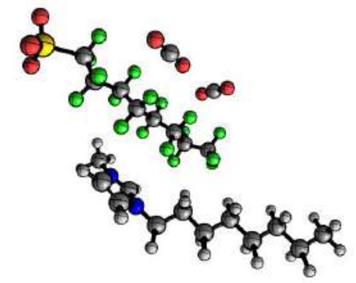	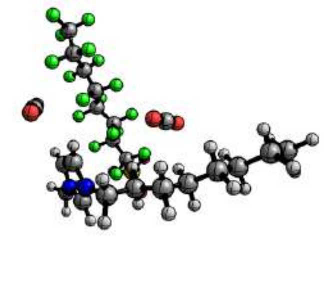	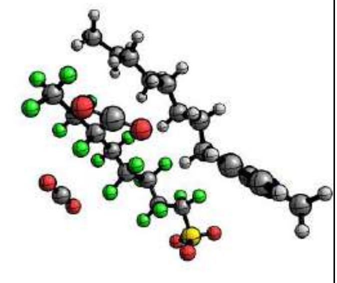	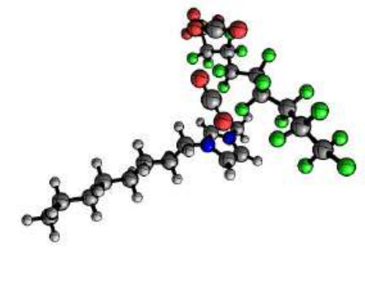	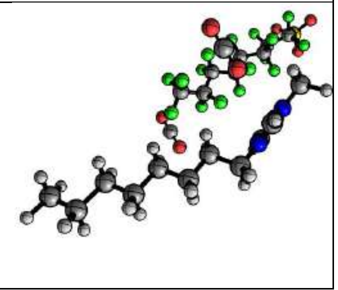

7.25	7.34	7.72	7.78	8.39
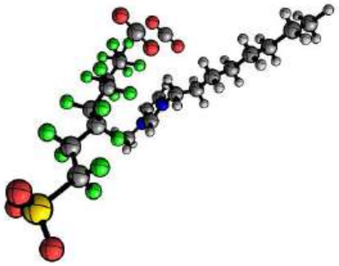	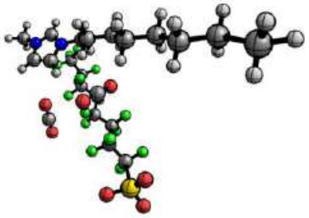	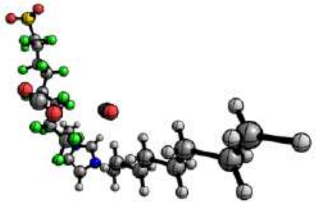	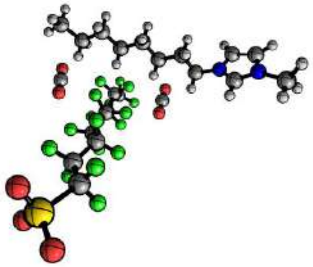	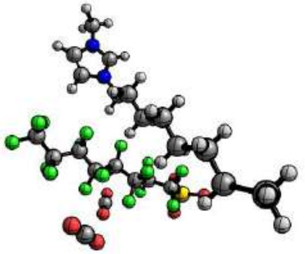
9.20	9.47	10.99	11.82	12.07
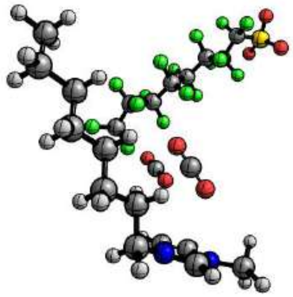	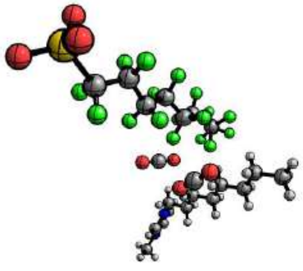	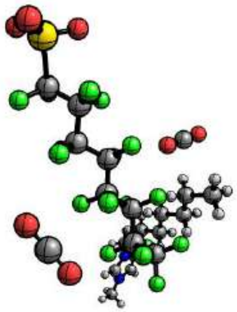	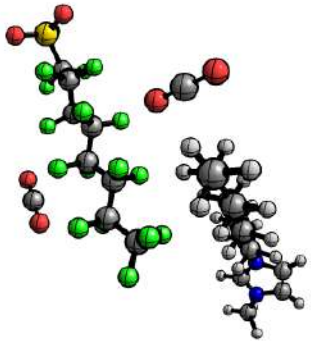	
12.48	13.20	13.44	16.61	

Table S50. Representations of lowest energy forms for $3CO_2[Omim]^+[(PFOc)SO_3]^-$at the M06-2X-D3/6-31G(d,p) level with an implicit solvent model PCM. Relative energies are listed in kcal/mol.

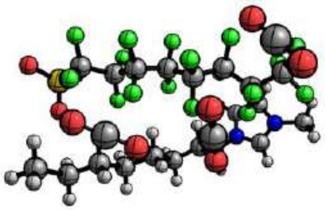	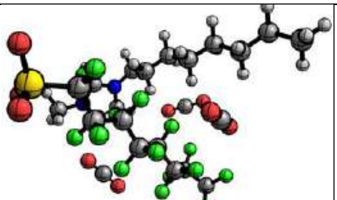	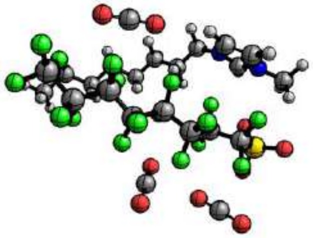	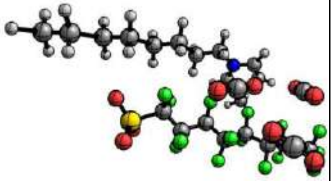	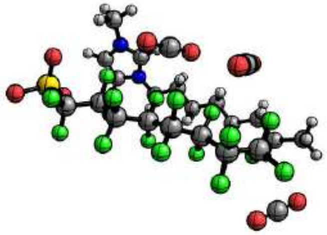
0.00	0.65	0.70	1.54	1.63

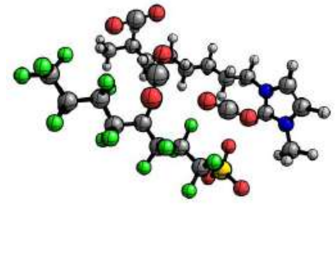	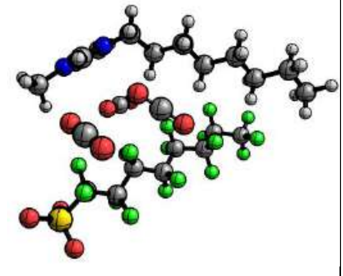	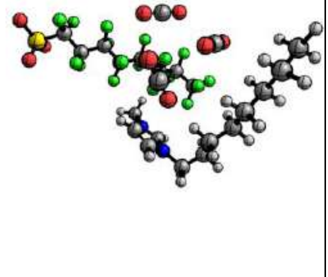	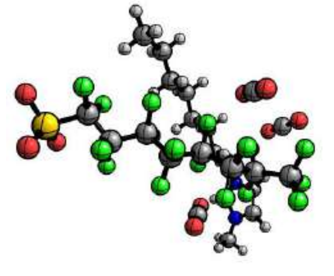	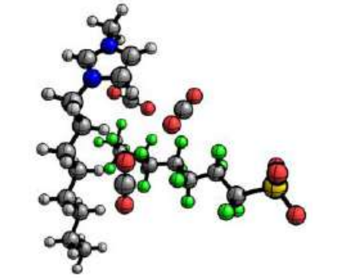
1.69	2.62	2.76	2.93	3.03
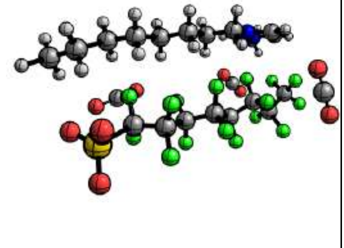	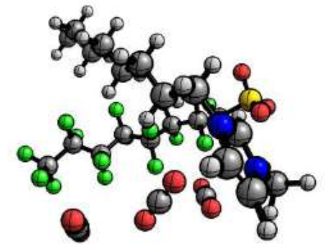	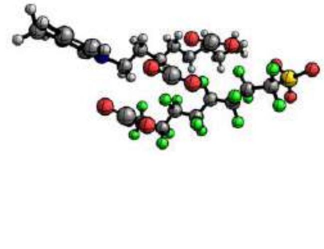	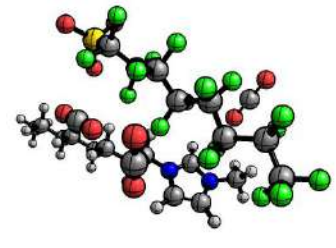	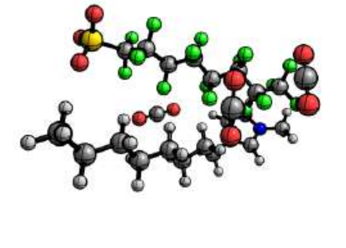
3.46	3.47	3.50	3.64	4.54
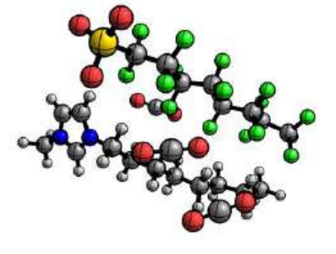	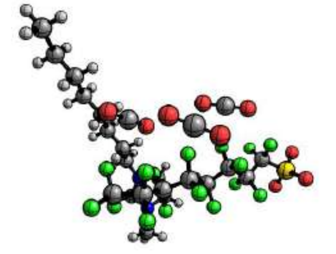	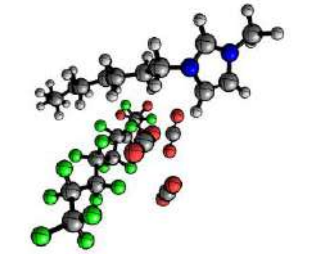	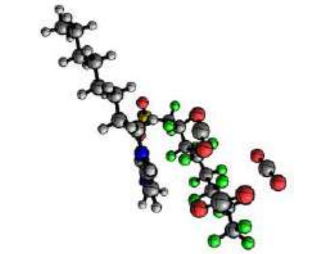	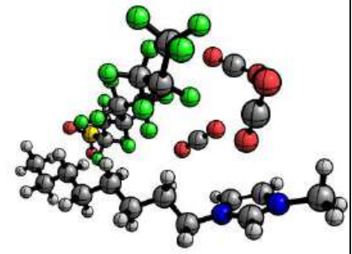
4.75	4.96	5.63	5.64	5.82

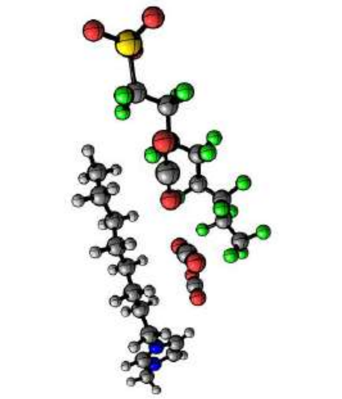	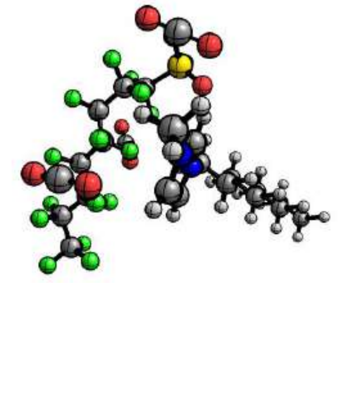	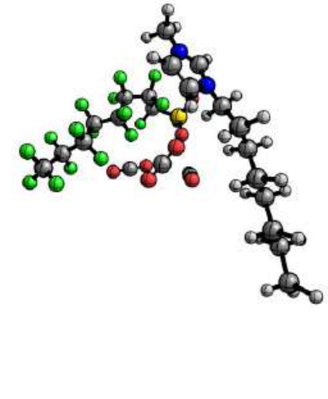	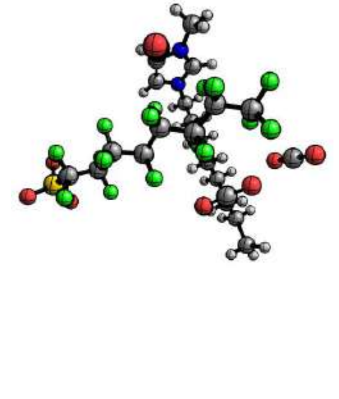	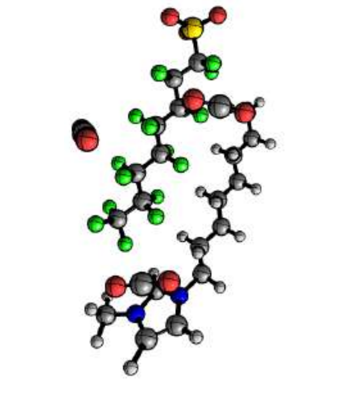
6.58	6.61	6.67	6.85	7.24
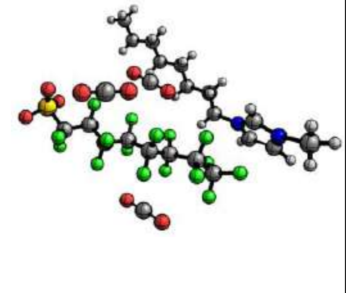	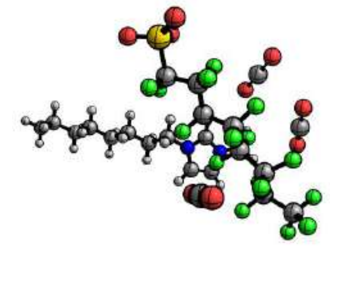	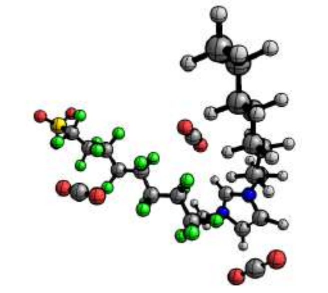	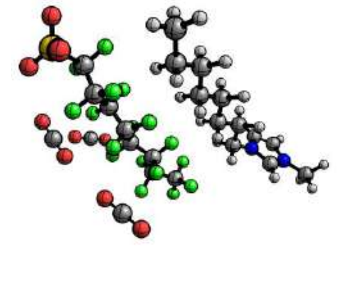	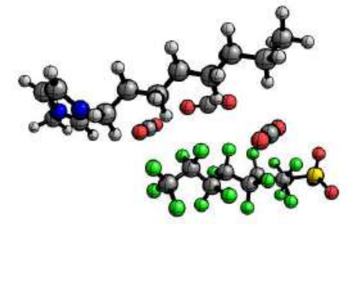
8.50	8.57	8.61	9.29	9.47
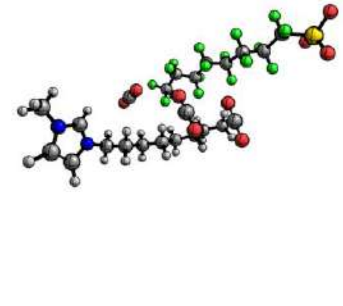	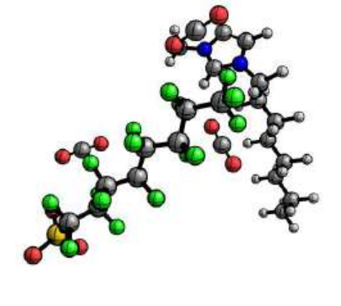	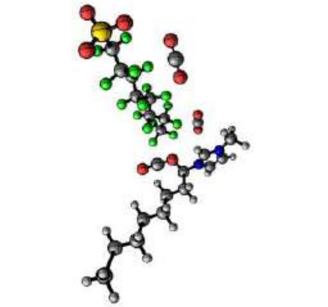		
9.79	10.53	13.21		

Table S51. Representations of lowest energy forms for $4CO_2[Omim]^+[(PFOc)SO_3]^-$ at the M06-2X-D3/6-31G(d,p) level with an implicit solvent model PCM. Relative energies are listed in kcal/mol.

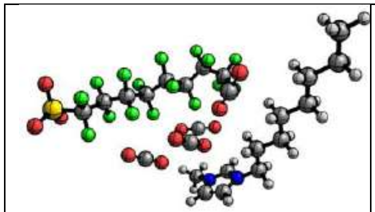	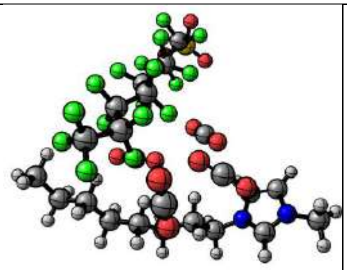	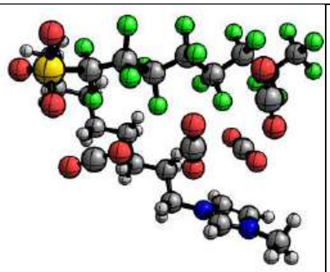	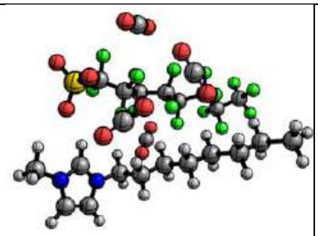	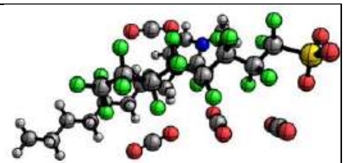
0.00	1.34	1.34	3.05	4.75
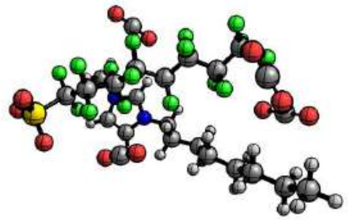	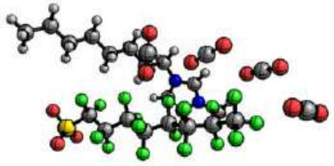	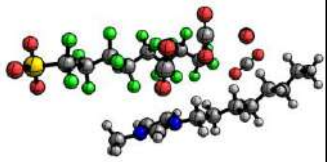	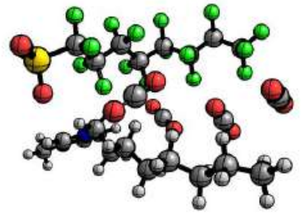	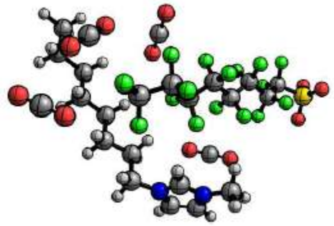
4.86	5.89	6.02	6.04	6.15
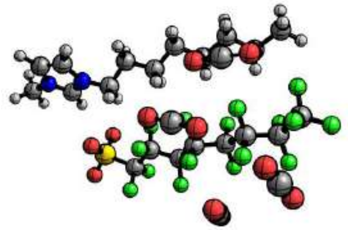	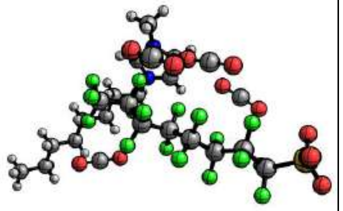	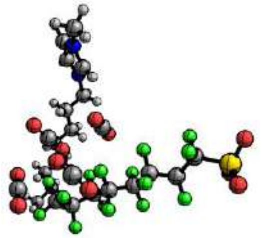	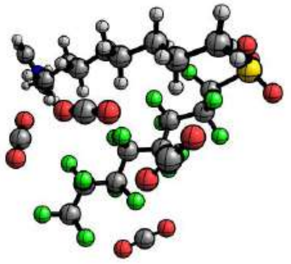	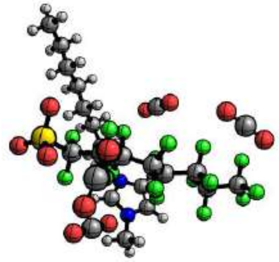
6.85	8.05	8.71	8.90	9.00

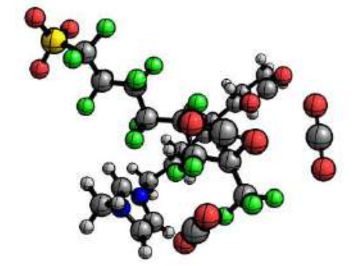				
9.15				

Table S52. Representations of lowest energy forms for $5CO_2[Omim]^+[(PFOc)SO_3]^-$ at the M06-2X-D3/6-31G(d,p) level with an implicit solvent model PCM. Relative energies are listed in kcal/mol.

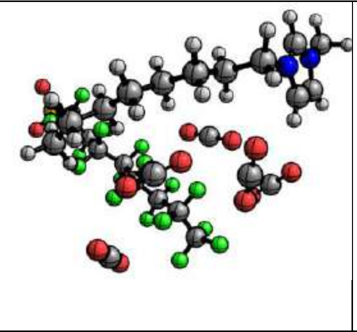	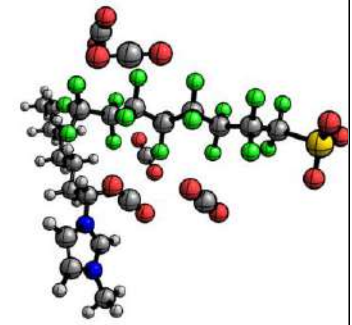	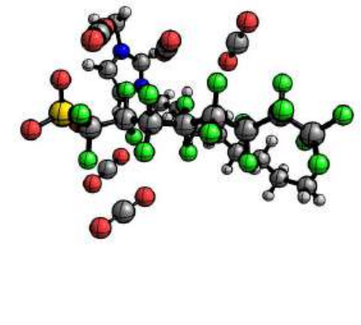	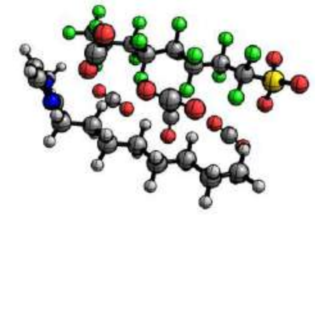	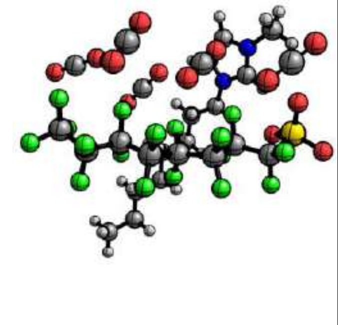
0.00	0.01	0.82	1.66	1.96
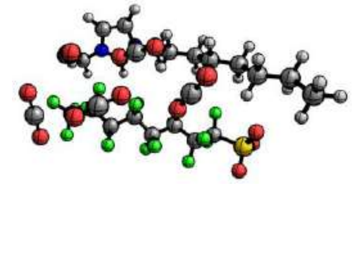	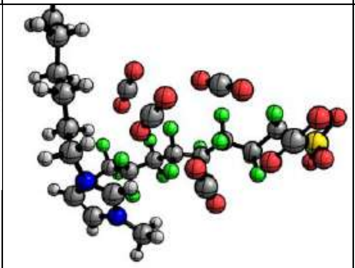	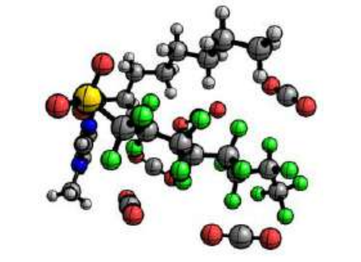	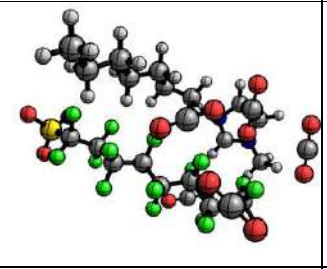	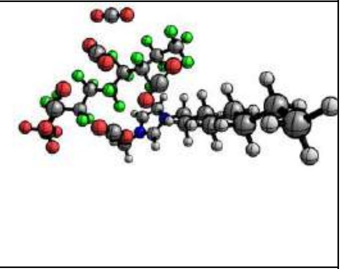
2.31	2.63	3.41	3.74	4.69

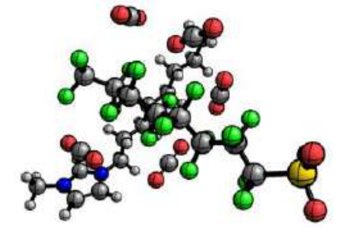	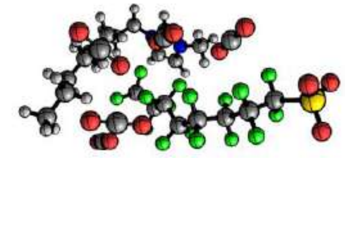	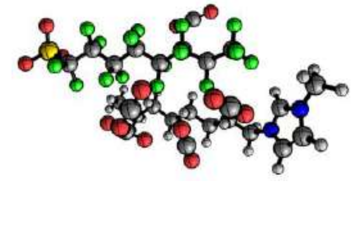	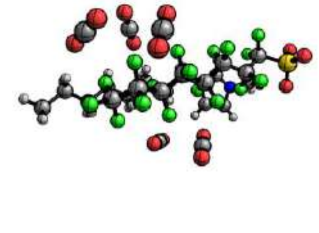	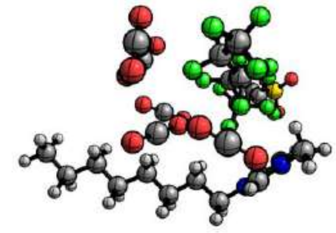
4.72	4.95	5.10	5.46	
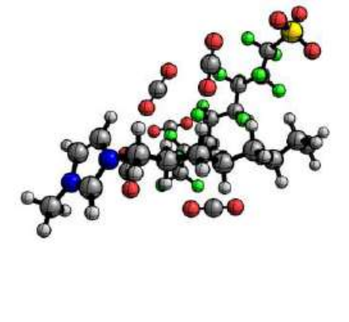	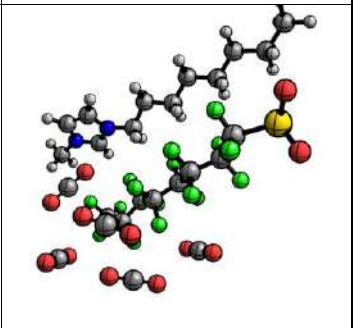	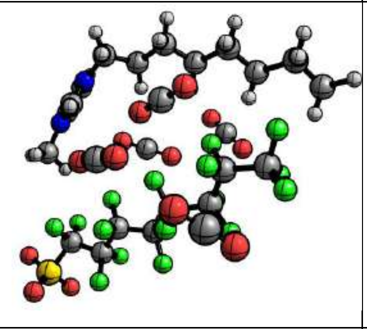	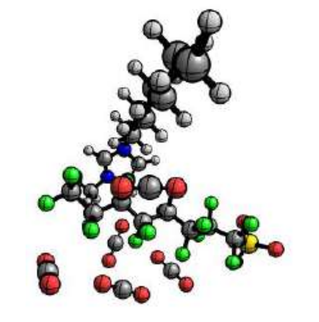	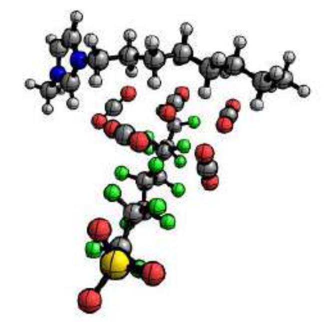
5.77	6.01	6.09	6.67	7.08
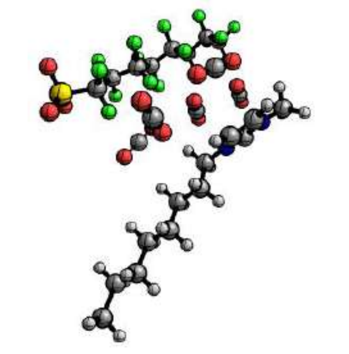	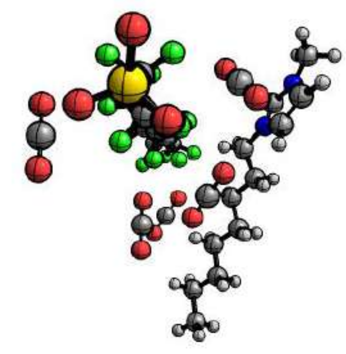	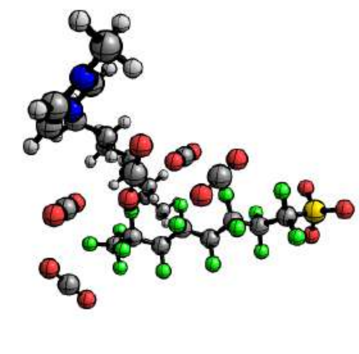	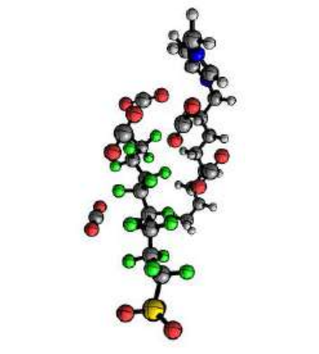	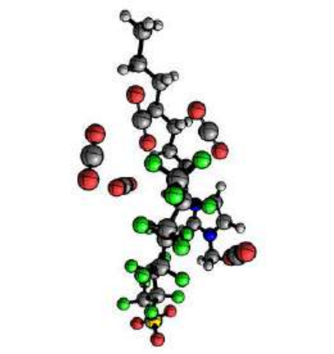
8.10	8.11	8.56	8.89	9.53

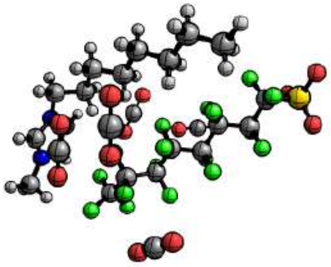	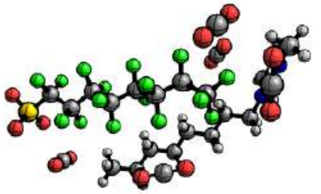	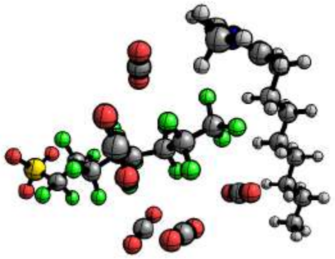	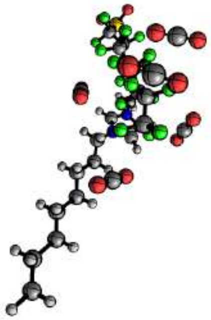	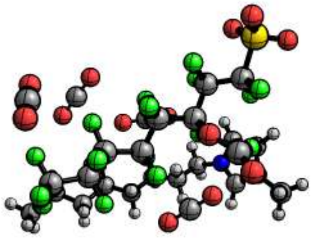
10.48	12.99	13.03	14.66	16.18
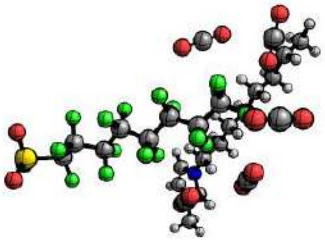	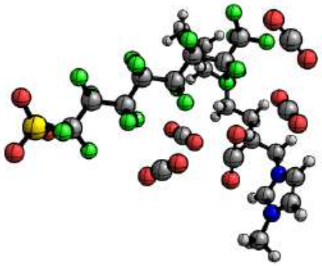			
19.00	19.15			

Table S53. Representations of lowest energy forms for $1CO_2[Hmim]^+[(PFBu)SO_3]^-$ at the M06-2X-D3/6-31G(d,p) level with an implicit solvent model PCM. Relative energies are listed in kcal/mol.

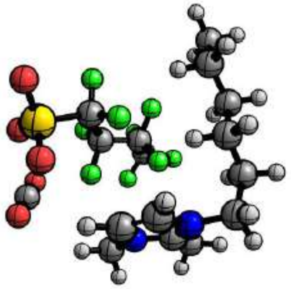	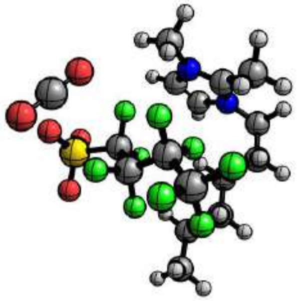	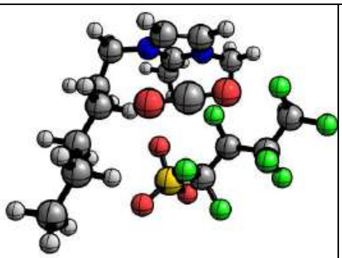	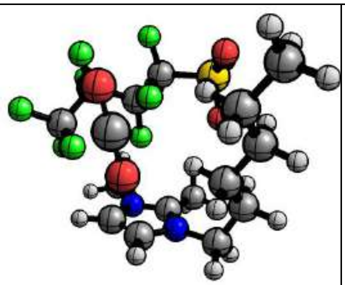	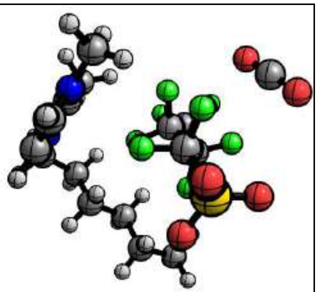
0.00	0.46	0.55	1.30	1.84

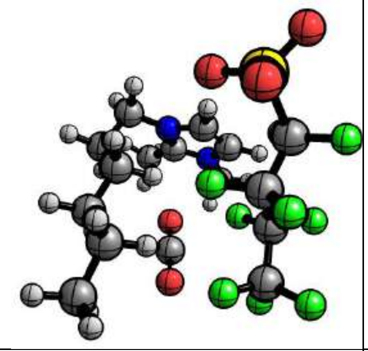	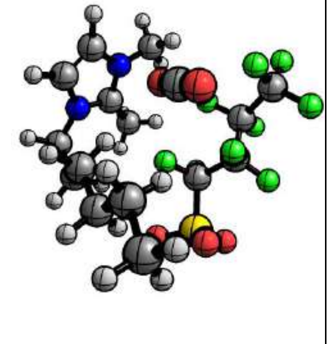	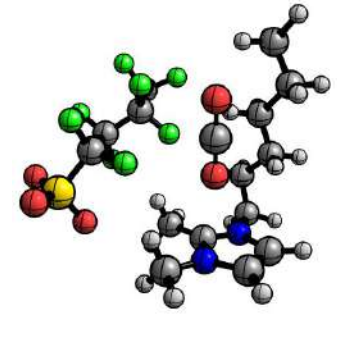	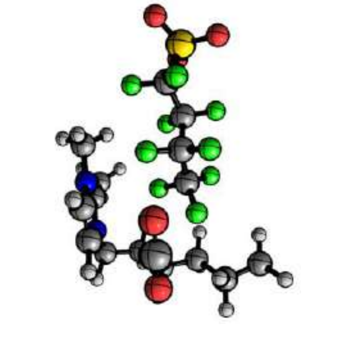	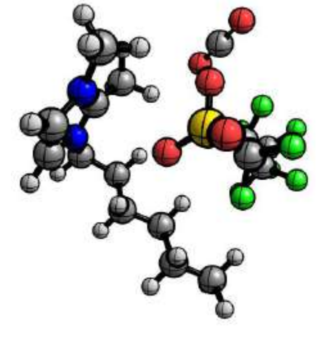
2.01	2.82	2.83	2.90	2.98
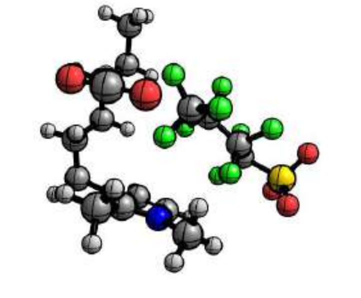	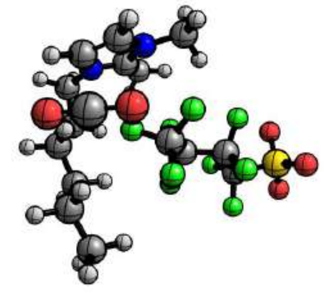	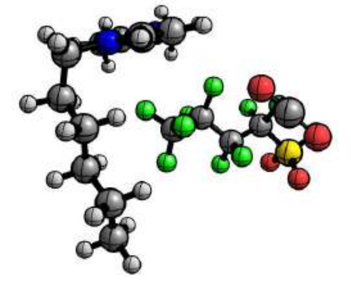	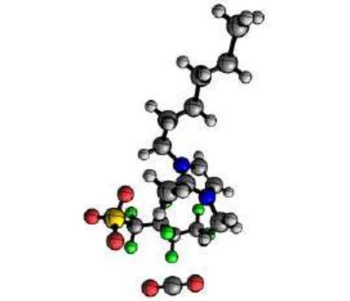	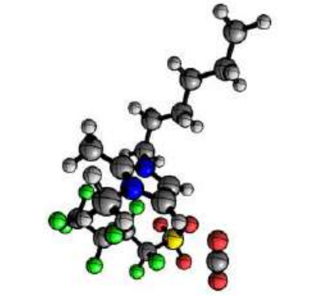
3.25	3.63	3.67	4.83	5.45
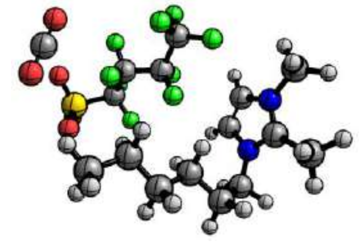	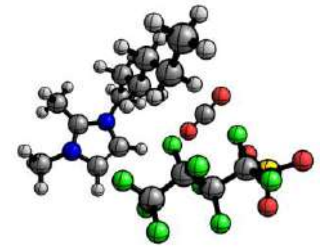	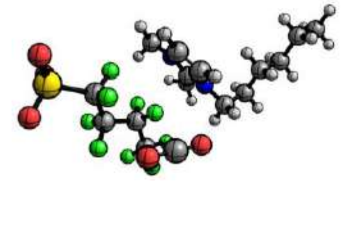	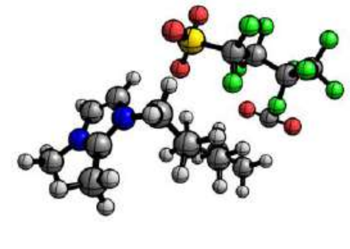	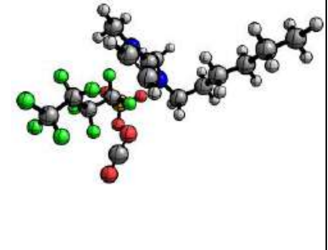
5.46	5.50	5.73	5.77	5.85

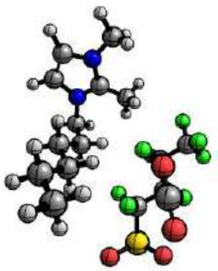	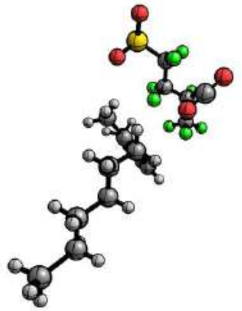	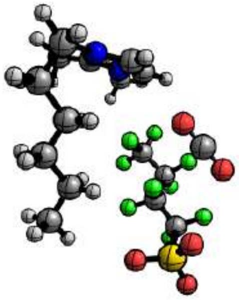	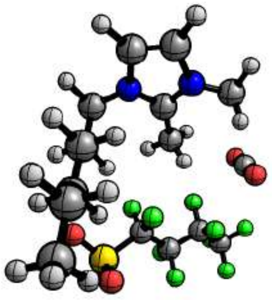	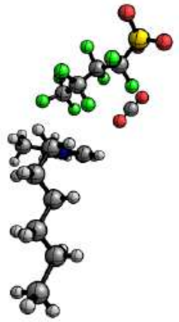
6.15	6.34	6.59	6.80	7.25
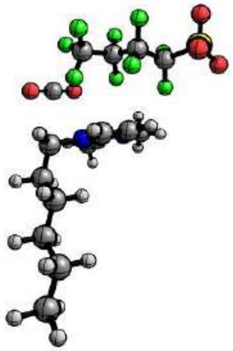	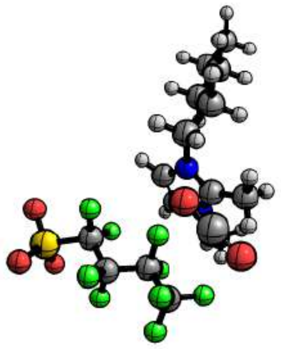	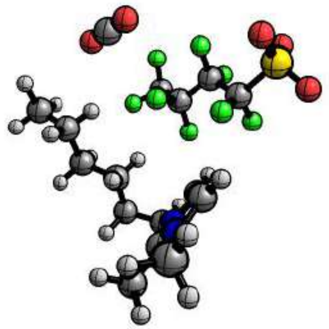	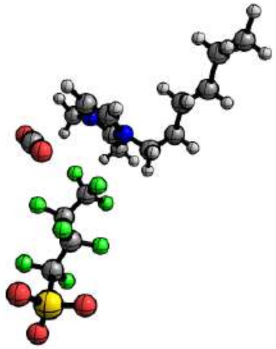	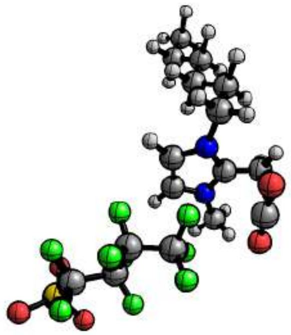
7.41	7.58	8.30	8.43	9.02

Table S54. Representations of lowest energy forms for $2CO_2[Hmim]^+[(PFBu)SO_3]^-$ at the M06-2X-D3/6-31G(d,p) level with an implicit solvent model PCM. Relative energies are listed in kcal/mol.

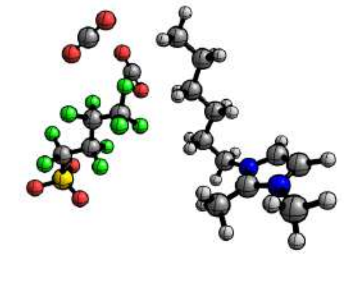	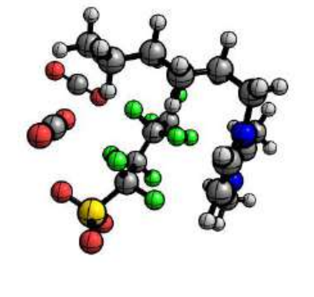	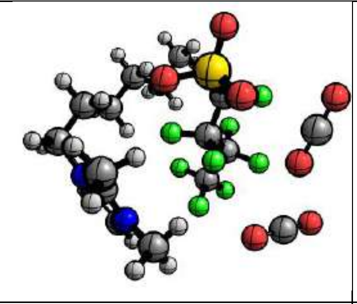	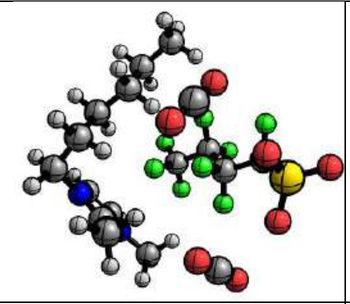	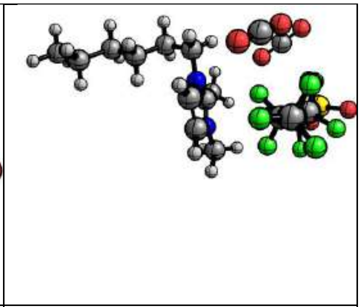
0.00	0.35	2.80	3.64	5.22
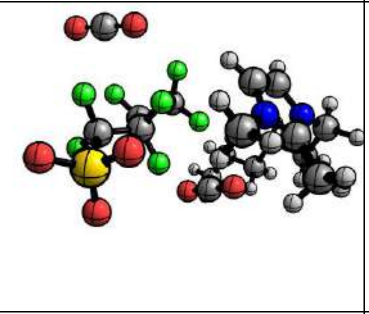	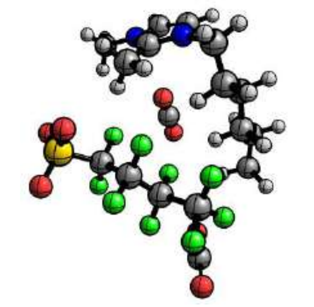	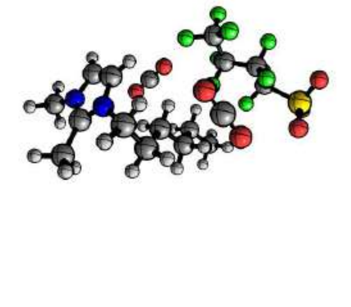	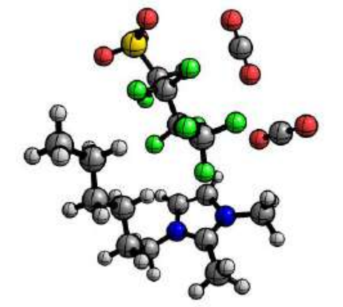	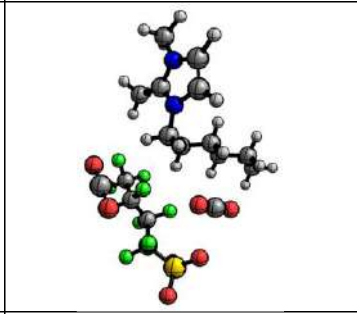
5.96	6.17	6.94	7.73	8.47
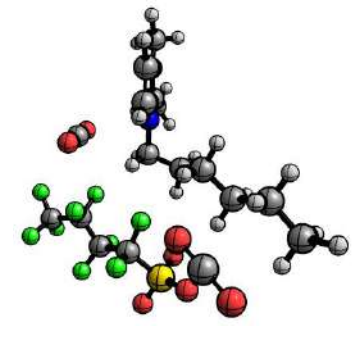	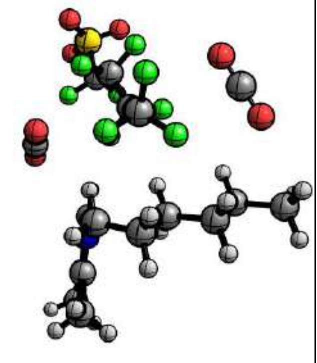	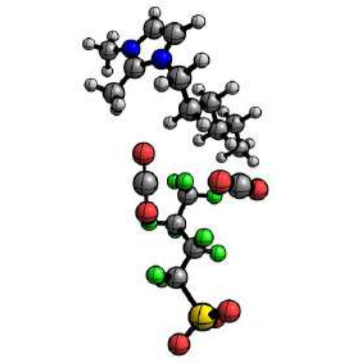	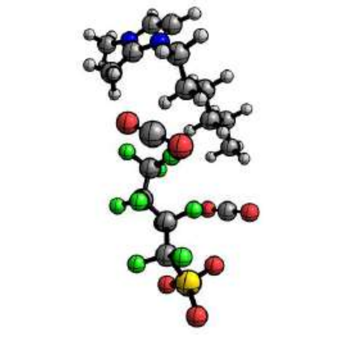	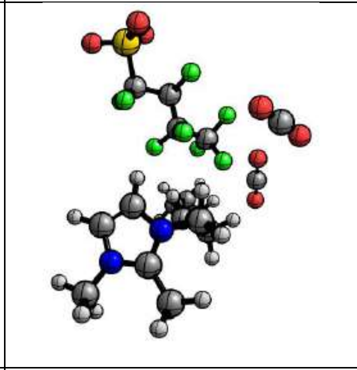
9.42	10.59	10.90	11.74	11.96

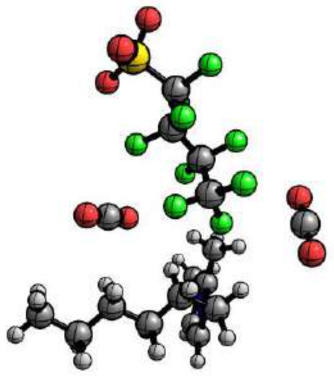				
12.58				

Table S55. Representations of lowest energy forms for $3CO_2[Hmim]^+[(PFBu)SO_3]^-$ at the M06-2X-D3/6-31G(d,p) level with an implicit solvent model PCM. Relative energies are listed in kcal/mol.

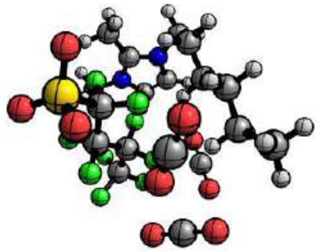	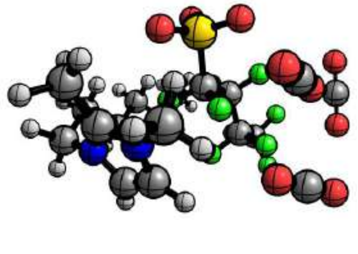	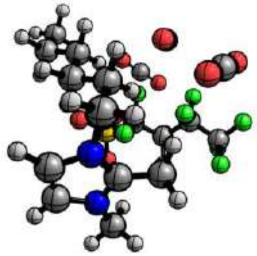	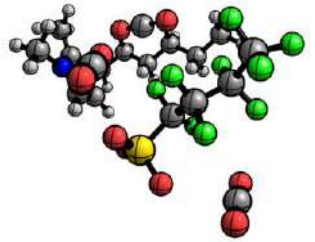	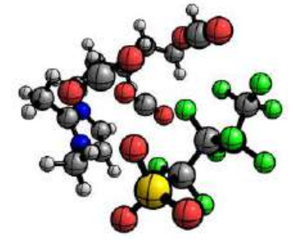
0.00	1.14	2.11	3.10	3.40
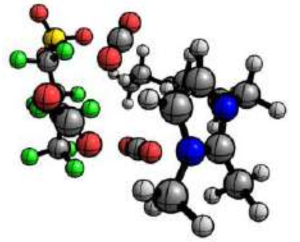	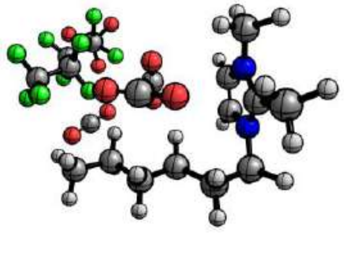	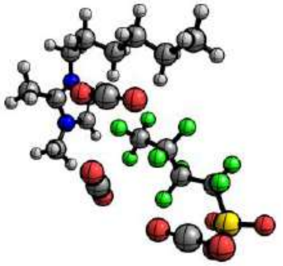	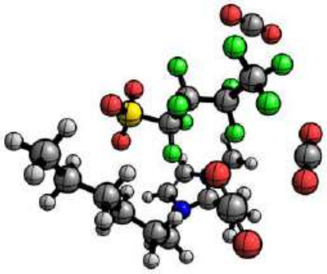	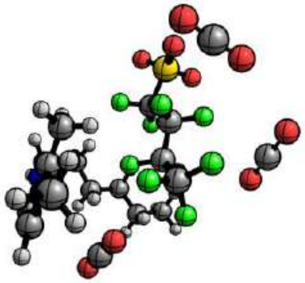

3.91	4.38	5.34	5.90	5.91
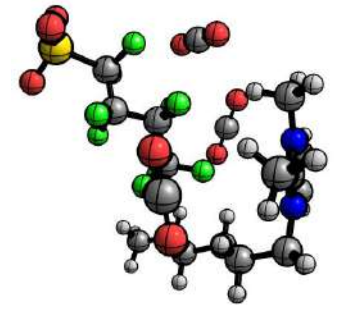	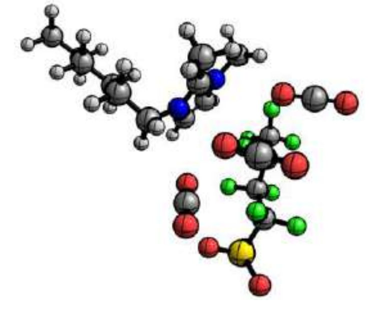	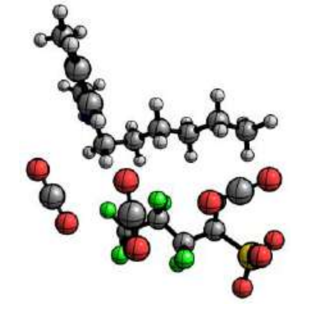	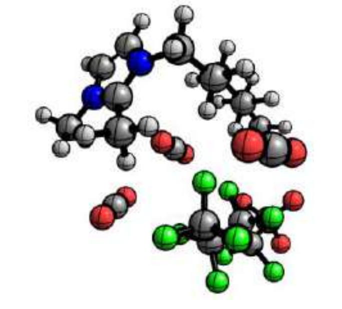	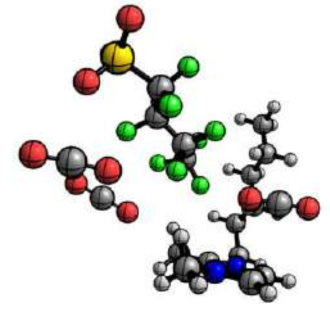
6.25	6.26	6.64	6.67	6.74
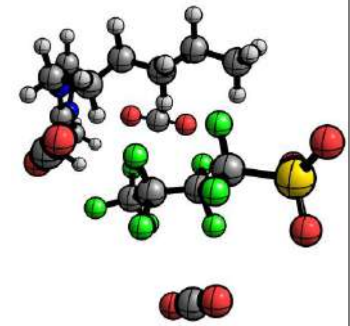	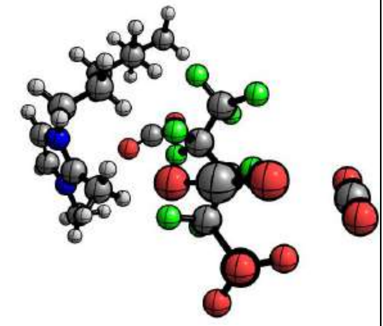	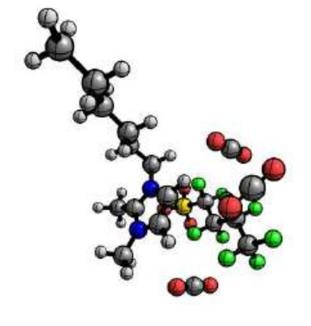	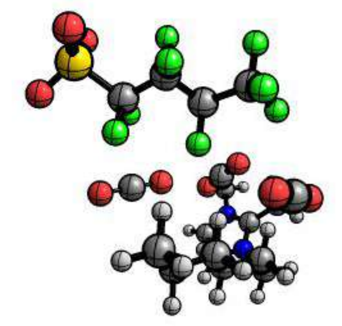	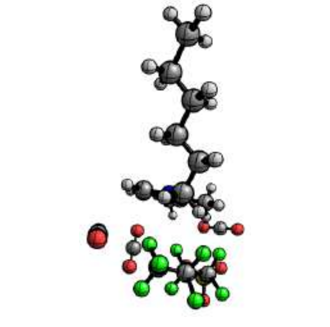
6.76	6.77	7.75	8.03	8.22
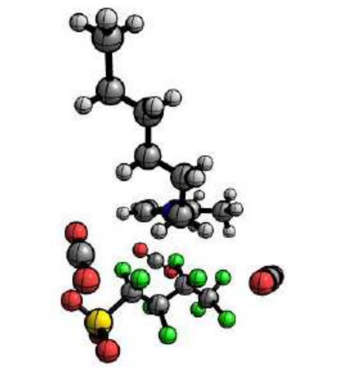	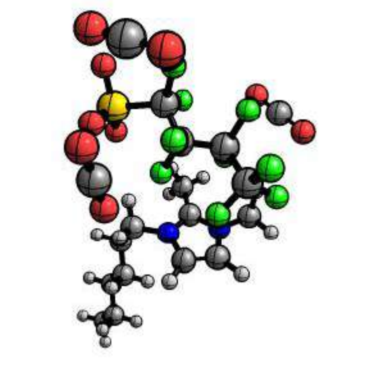	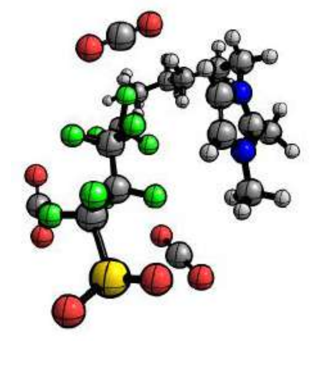	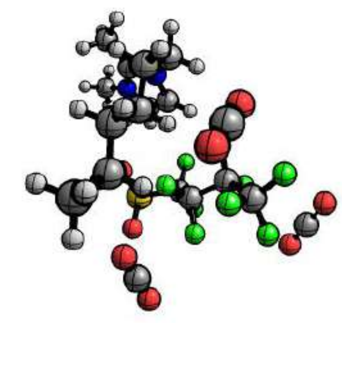	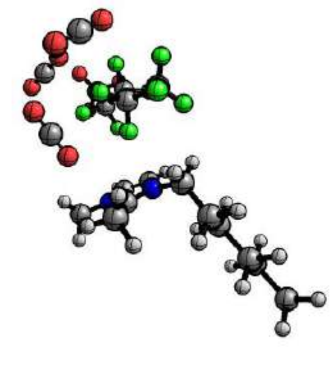
8.34	9.08	9.44	9.45	9.66

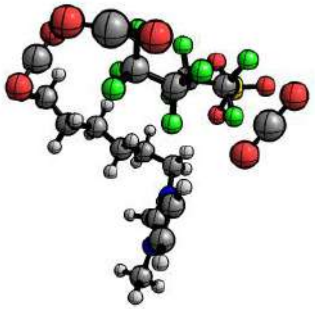	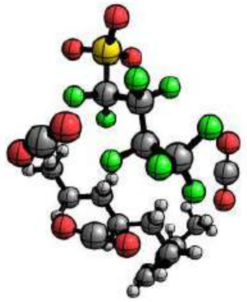	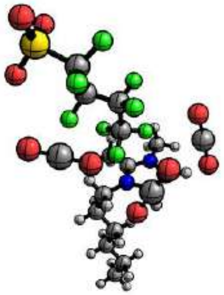	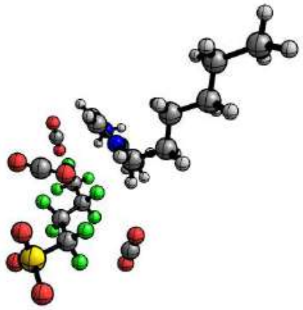	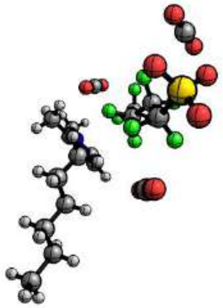
9.69	10.06	10.21	10.91	11.18
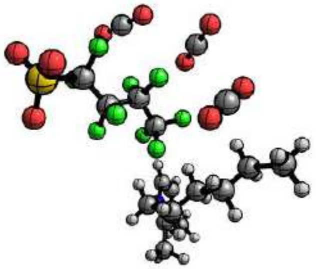	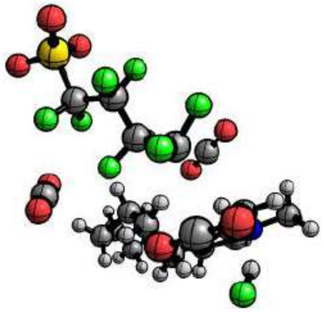	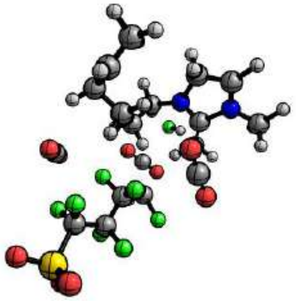		
11.25	11.86	14.27		

Table S56. Representations of lowest energy forms for $4CO_2[Hmim]^+[(PFBu)SO_3]^-$at the M06-2X-D3/6-31G(d,p) level with an implicit solvent model PCM. Relative energies are listed in kcal/mol.

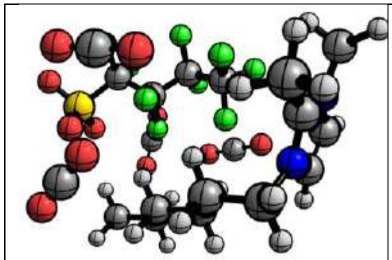	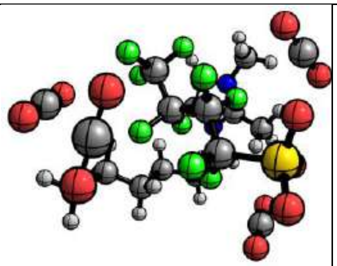	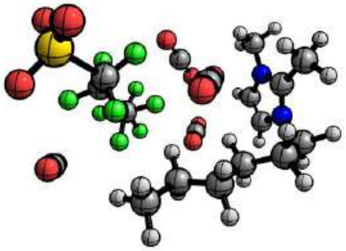	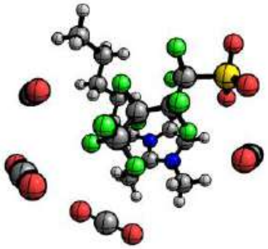	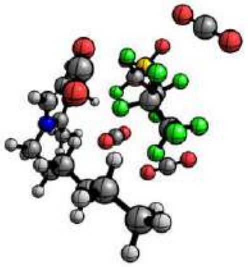
0.00	0.80	2.21	2.52	3.02

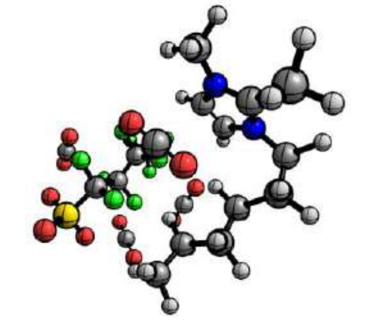	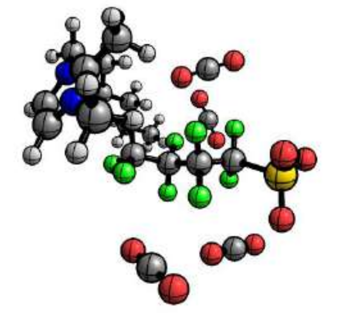	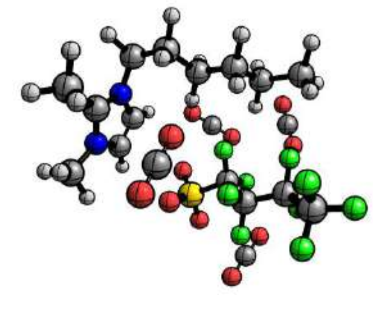	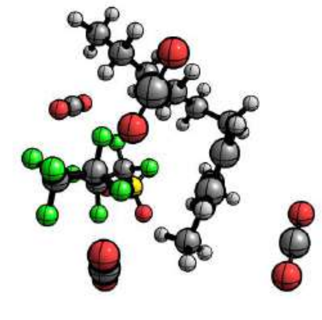	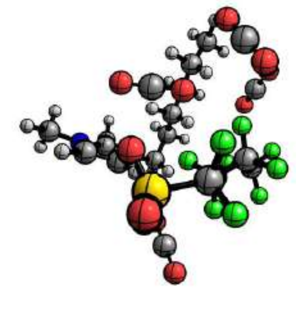
3.10	3.42	3.98	4.01	4.34
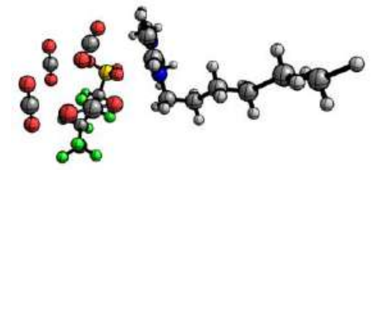	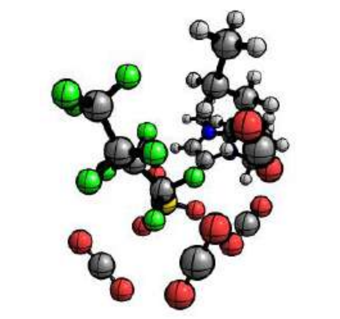	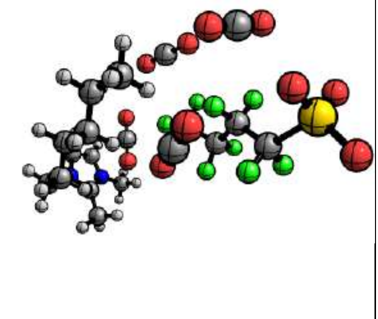	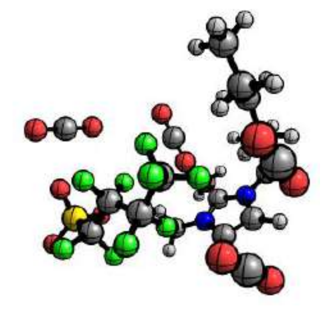	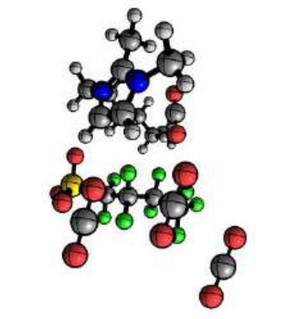
4.50	4.56	4.73	4.76	5.40
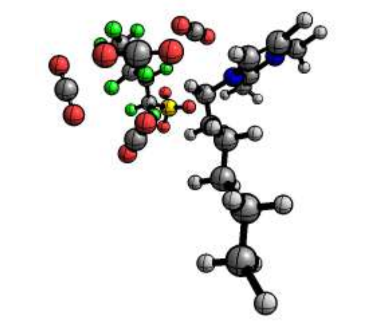	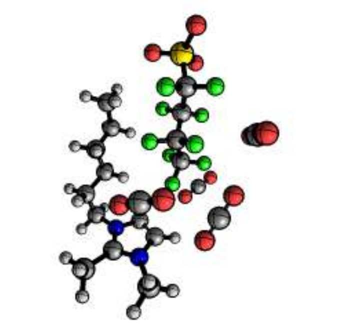	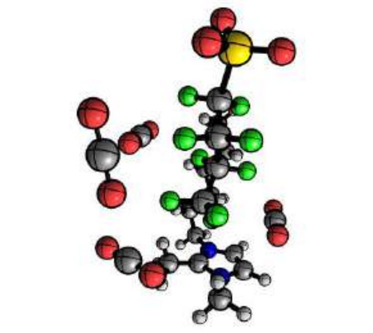	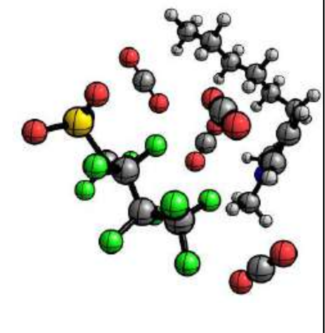	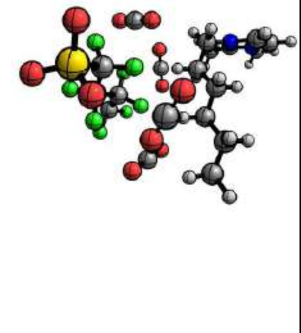
5.60	5.73	5.97	6.03	6.31

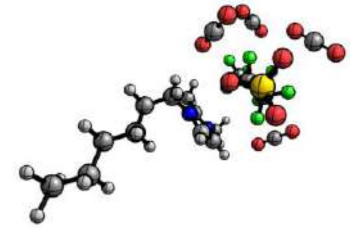	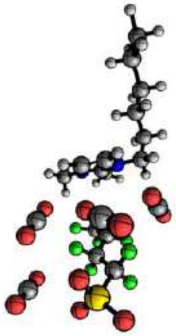	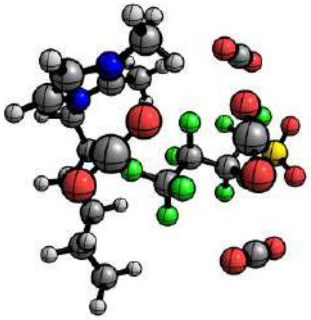	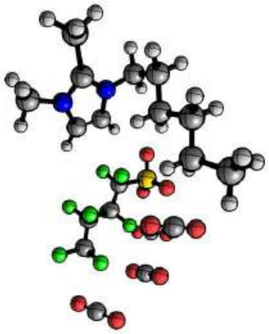	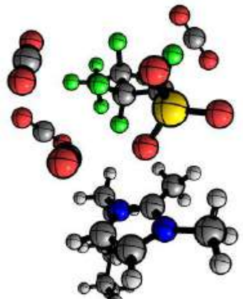
6.35	6.44	6.82	6.91	7.59
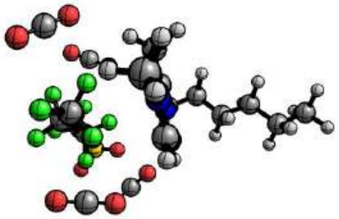	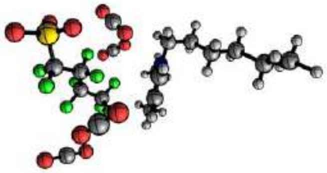	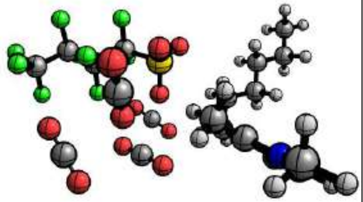	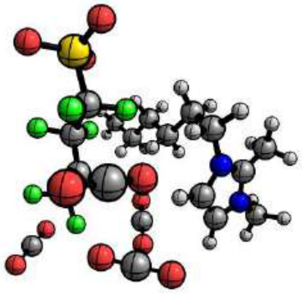	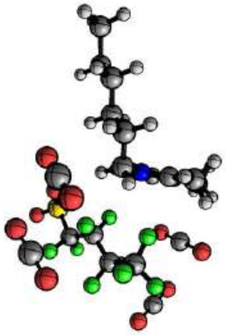
7.89	8.26	8.38	8.47	8.48
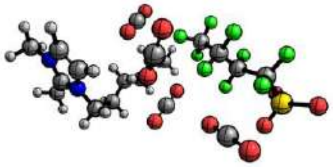	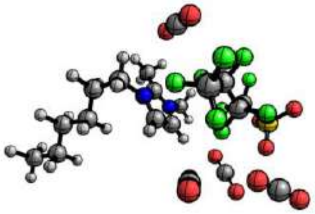	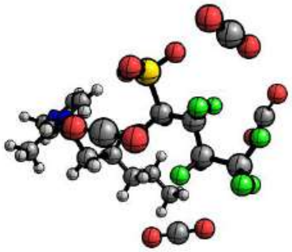		
8.86	8.87	9.78		

Table S57. Representations of lowest energy forms for $5CO_2[Hmim]^+[(PFBu)SO_3]^-$ at the M06-2X-D3/6-31G(d,p) level with an implicit solvent model PCM. Relative energies are listed in kcal/mol.

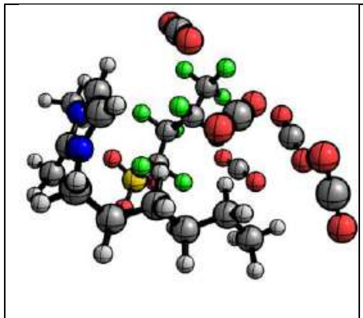	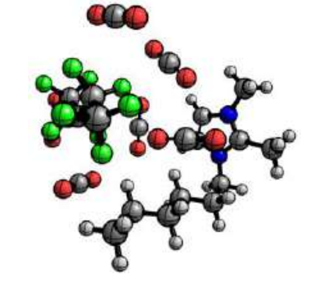	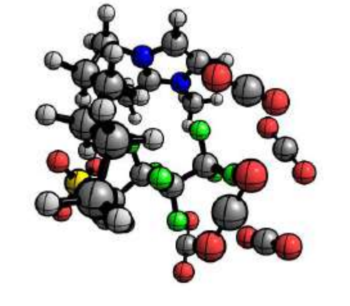	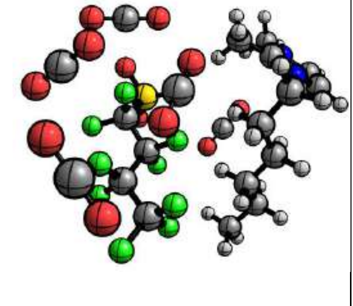	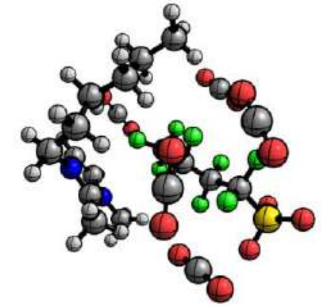
0.00	0.08	0.39	0.75	0.85
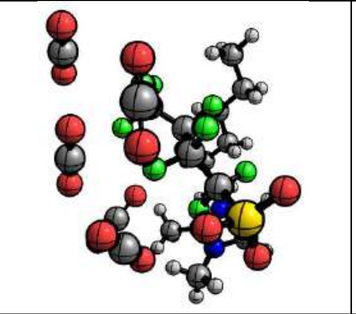	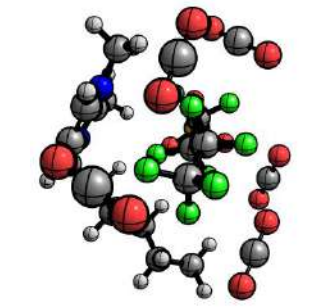	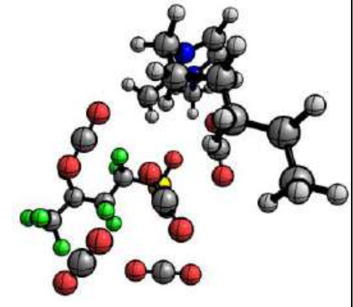	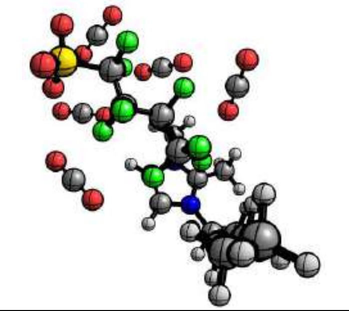	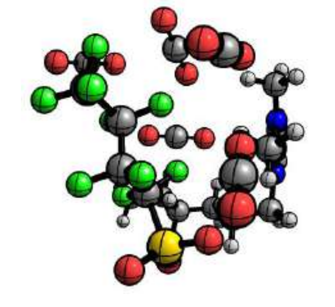
1.21	1.54	2.38	3.09	3.22
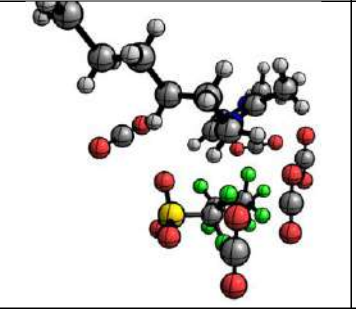	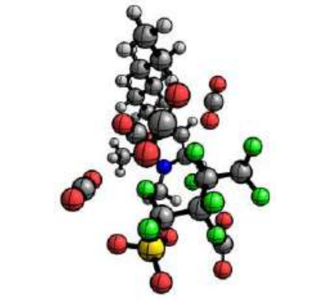	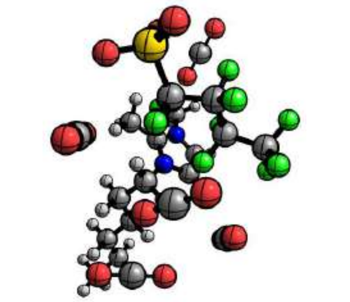	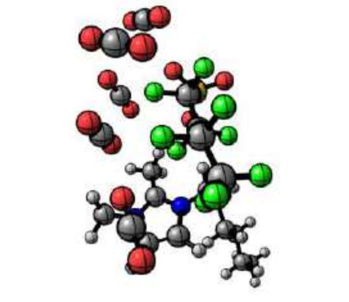	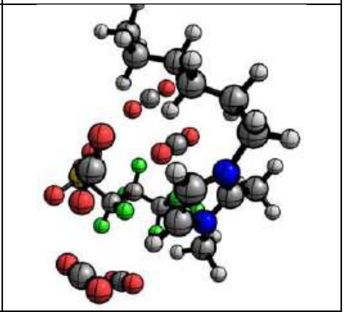
3.50	4.03	4.46	4.57	4.65

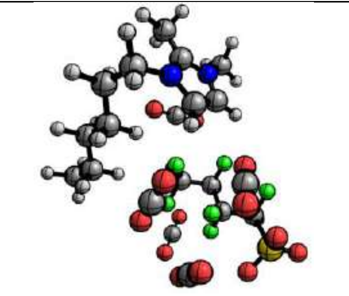	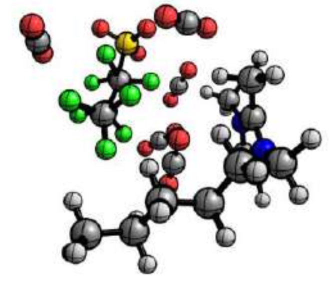	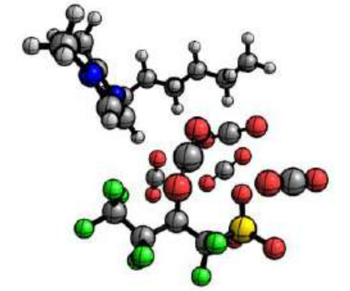	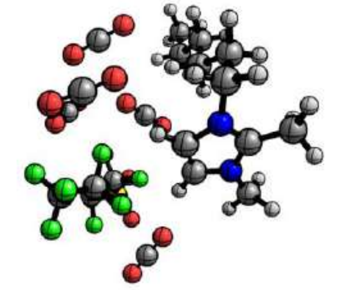	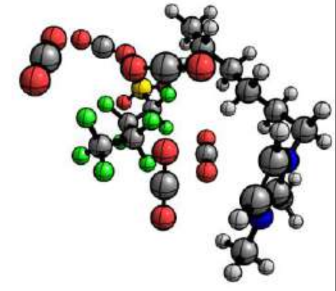
4.80	5.10	5.26	5.32	5.59
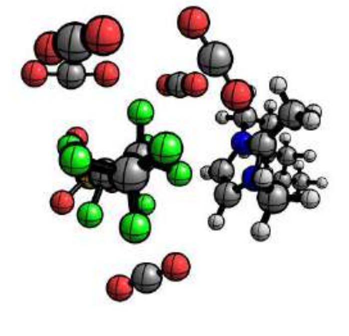	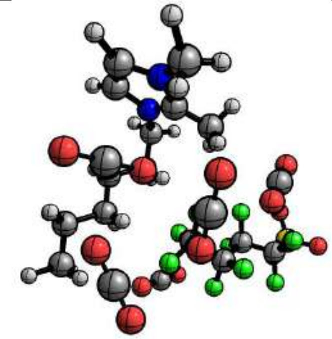	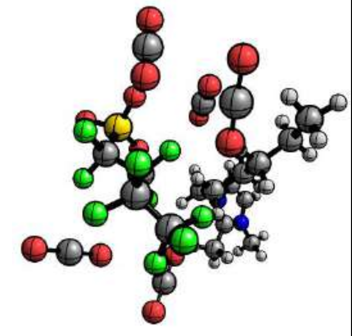	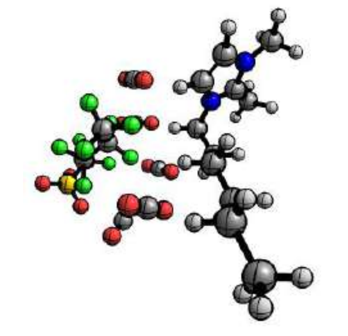	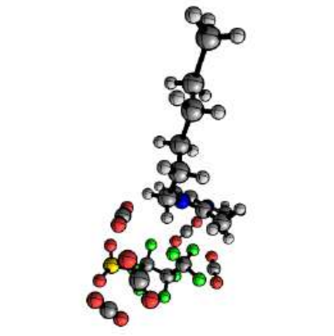
6.55	6.56	6.71	7.55	7.81
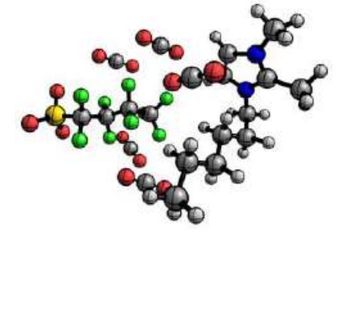	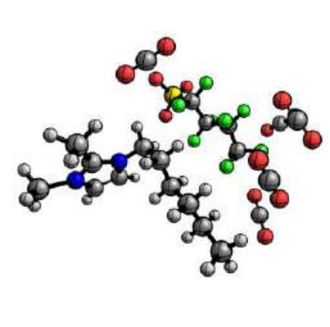	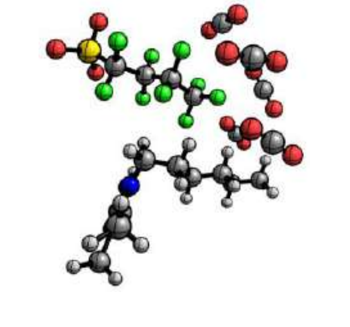	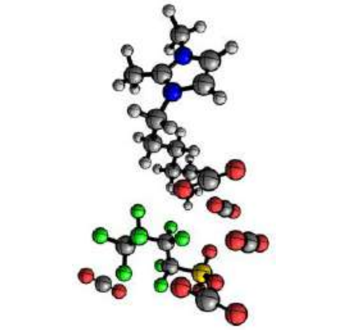	
9.31	9.64	9.76	9.88	

Table S58. Representations of lowest energy forms for $1CO_2[Omim]^+[(PFBu)SO_3]^-$ at the M06-2X-D3/6-31G(d,p) level with an implicit solvent model PCM. Relative energies are listed in kcal/mol.

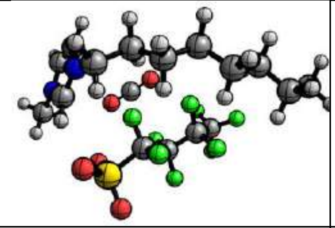	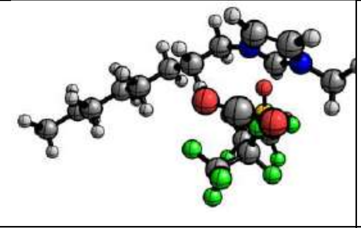	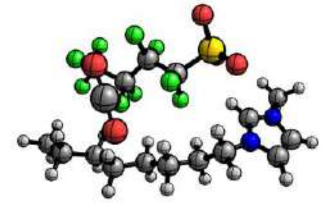	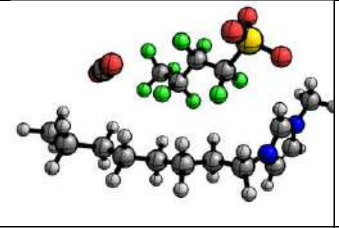	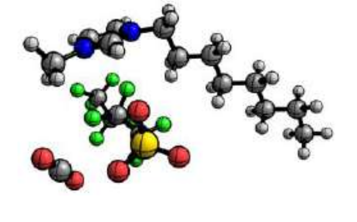
0.00	0.98	1.49	1.65	1.88
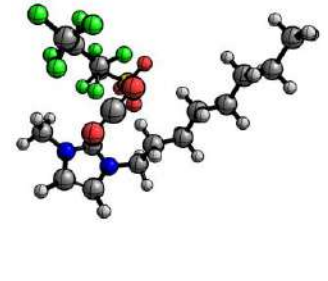	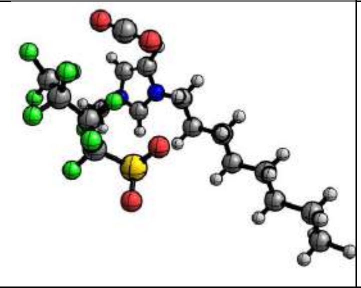	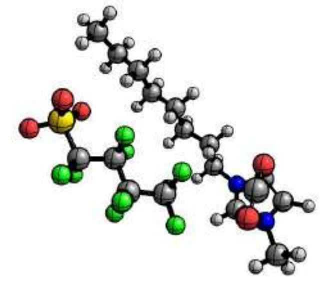	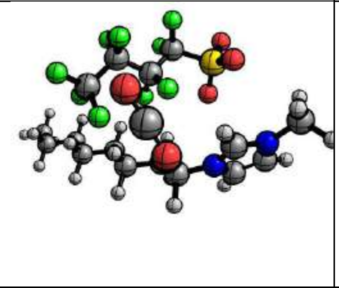	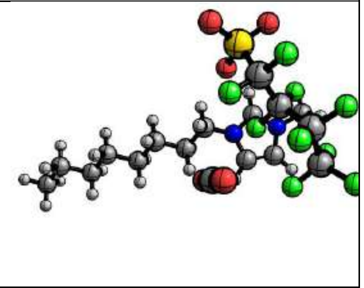
2.06	2.23	3.08	3.35	3.46
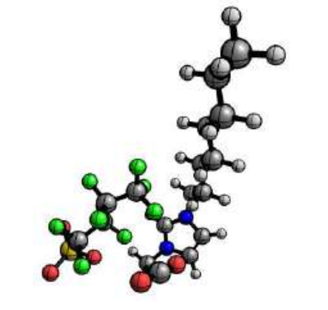	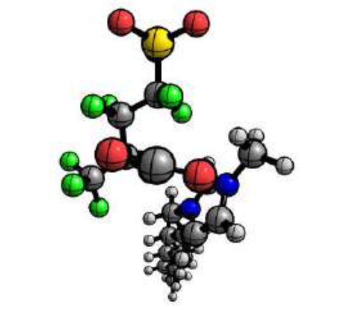	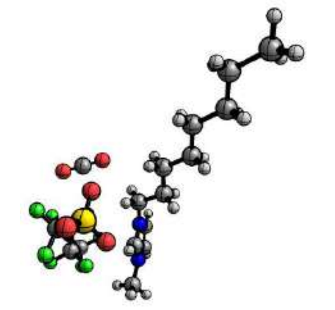	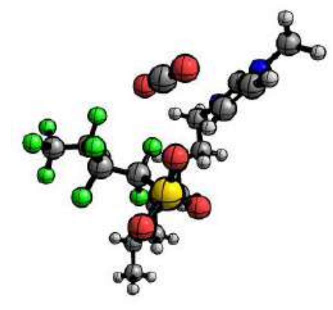	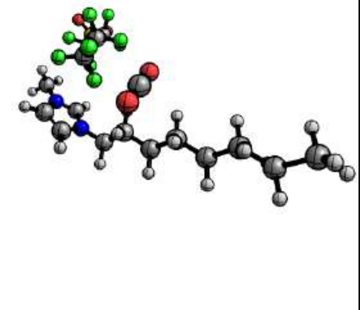
3.69	3.82	3.98	4.09	4.16

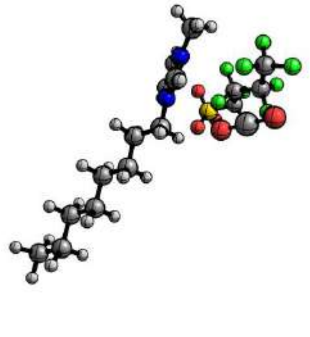	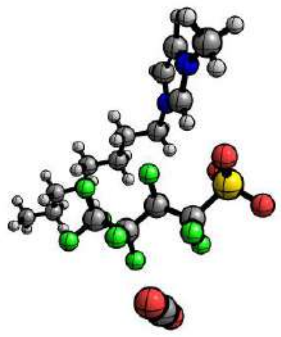	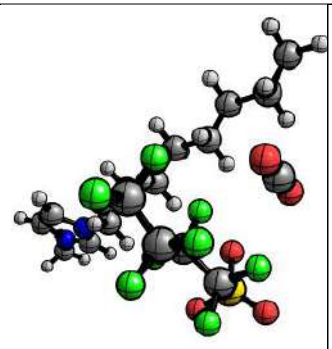	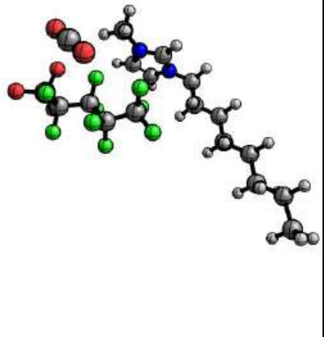	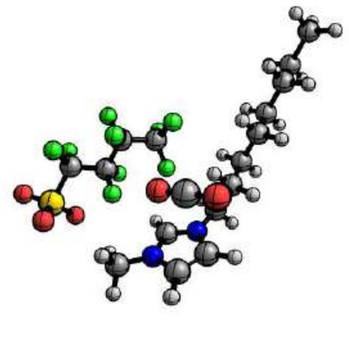
4.32	4.53	4.75	4.80	4.81
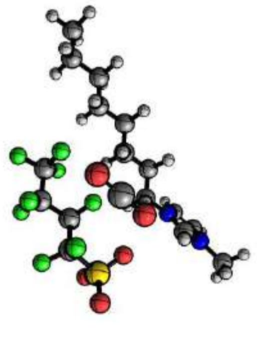	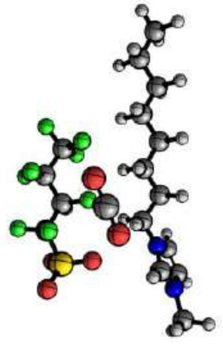	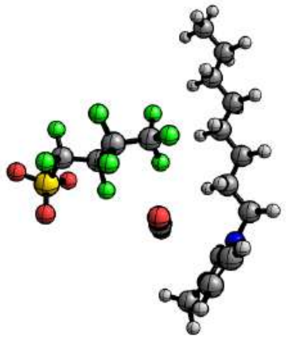	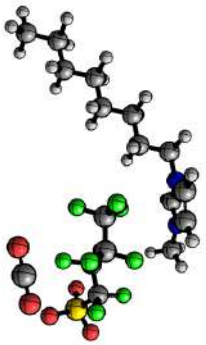	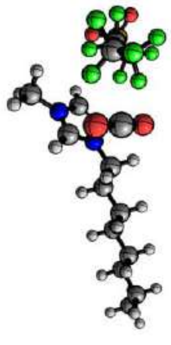
5.16	5.63	5.86	6.37	6.56
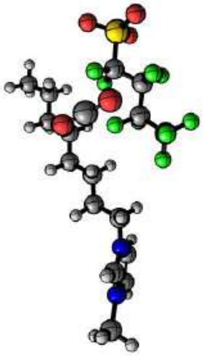	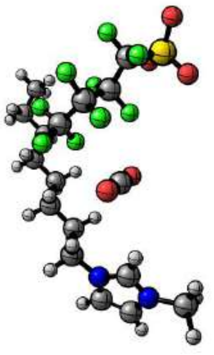	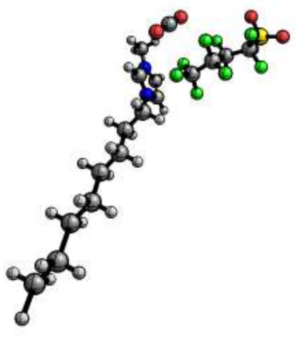	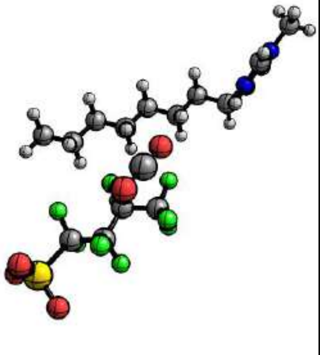	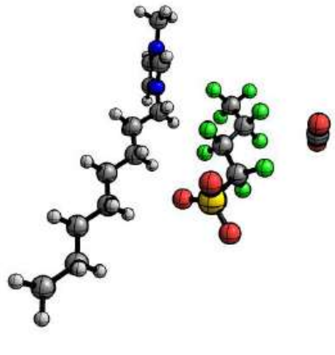
7.00	7.37	8.51	8.60	8.89

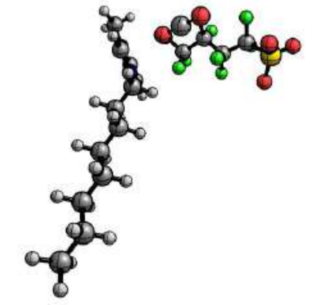				
8.89				

Table S59. Representations of lowest energy forms for $2CO_2[Omim]^+[(PFBu)SO_3]^-$at the M06-2X-D3/6-31G(d,p) level with an implicit solvent model PCM. Relative energies are listed in kcal/mol.

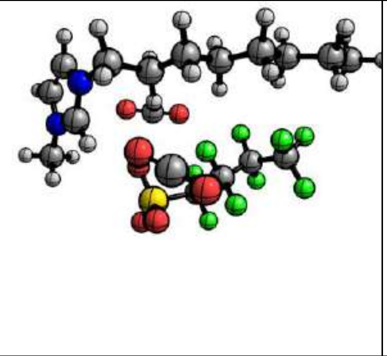	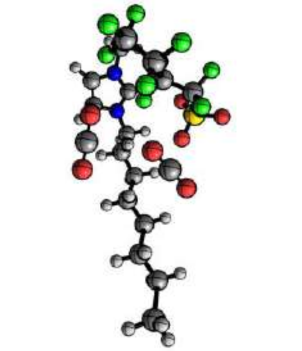	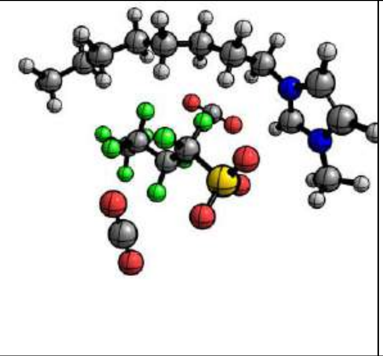	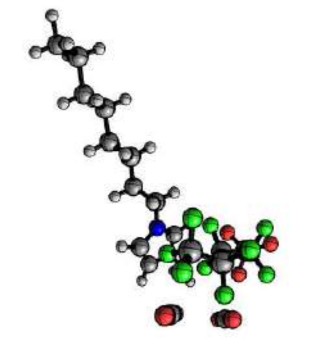	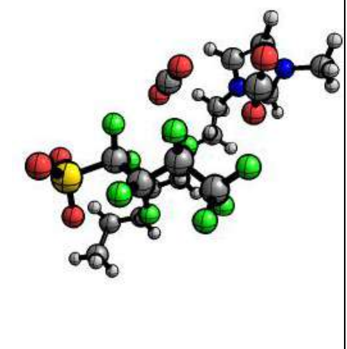
0.00	0.46	2.99	3.24	3.32

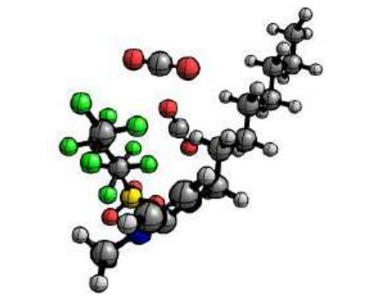	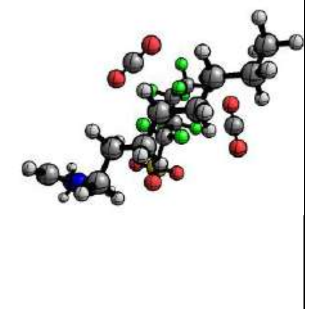	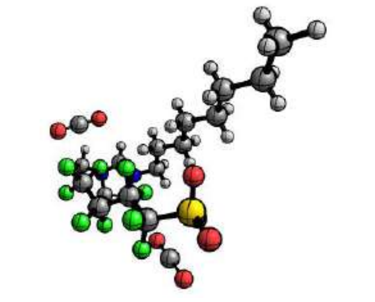	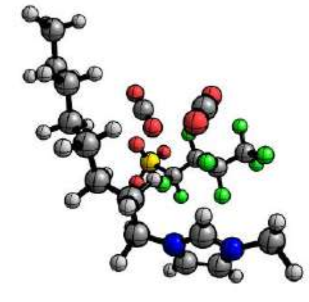	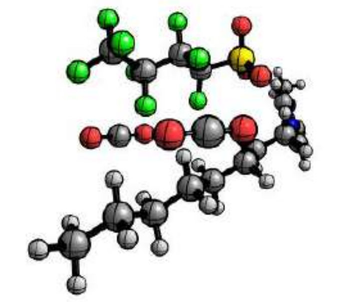
3.42	3.52	4.06	4.09	4.61
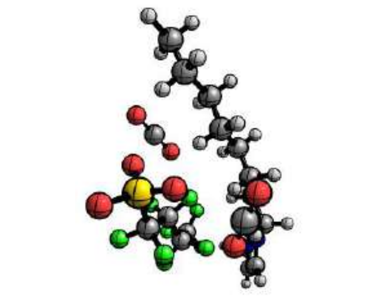	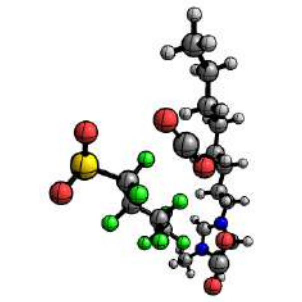	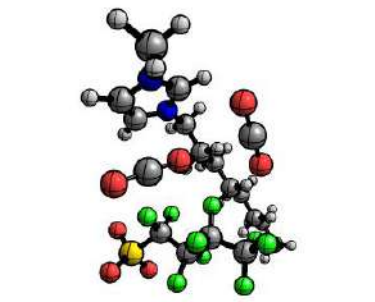	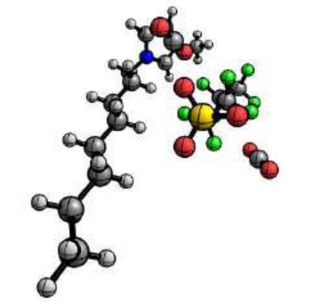	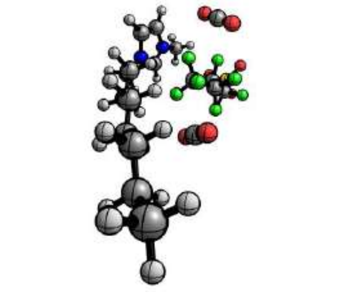
4.77	4.91	5.22	5.47	6.11
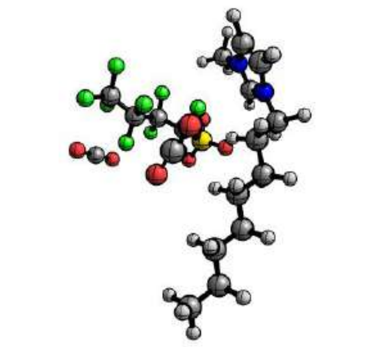	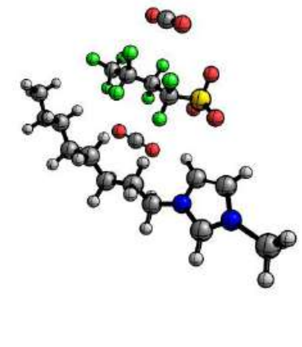	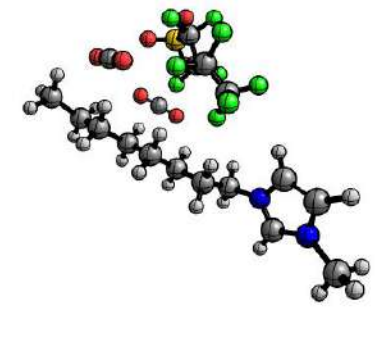	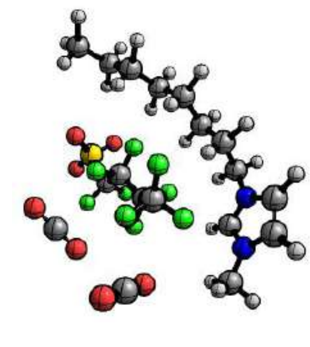	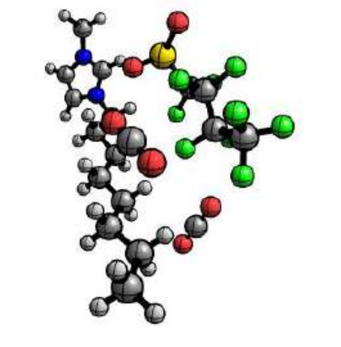
6.20	6.37	6.61	6.78	6.81

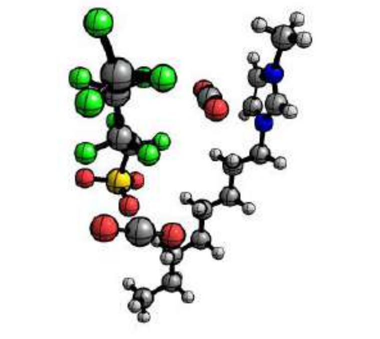	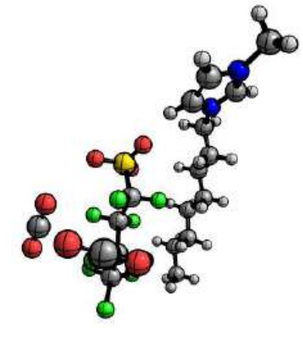	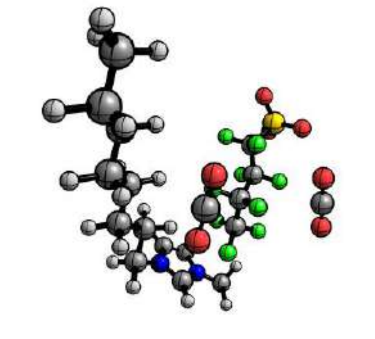	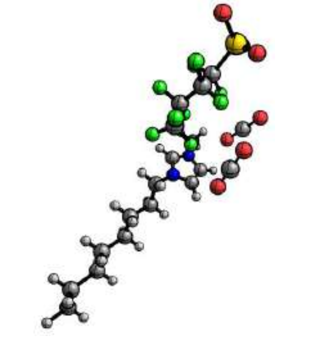	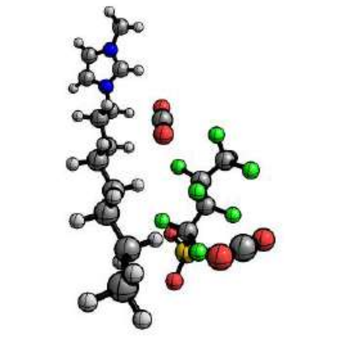
7.24	7.29	7.54	7.84	8.06
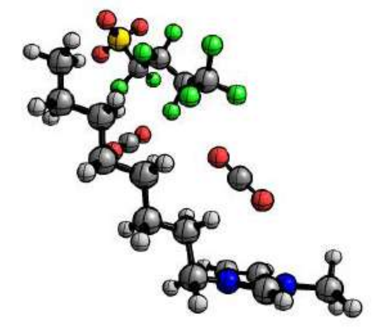	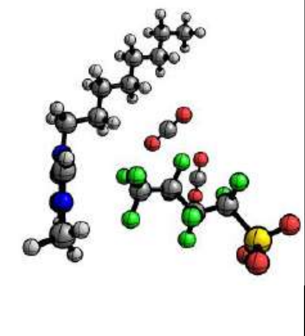	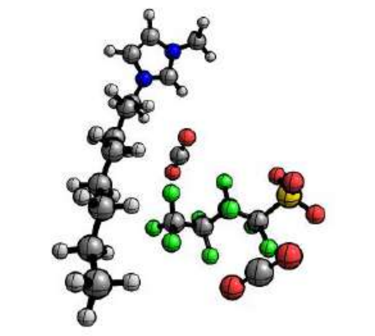	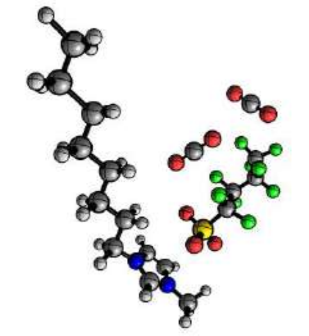	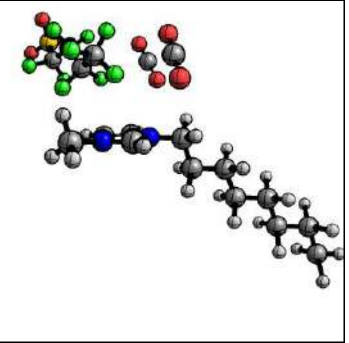
8.15	8.48	9.29	9.31	9.85
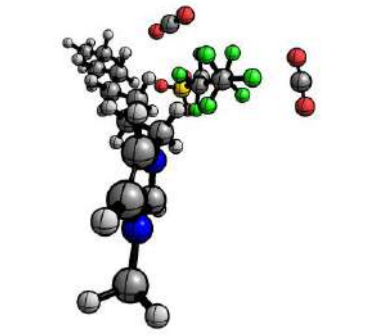	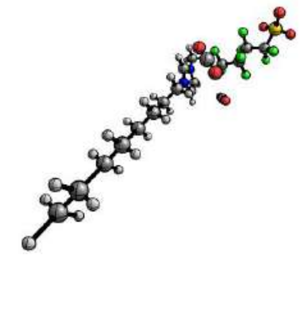	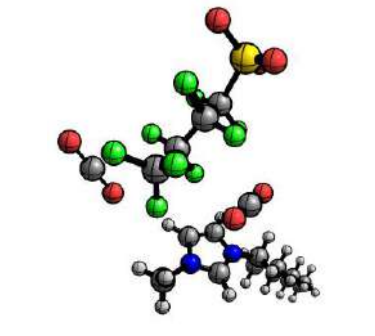	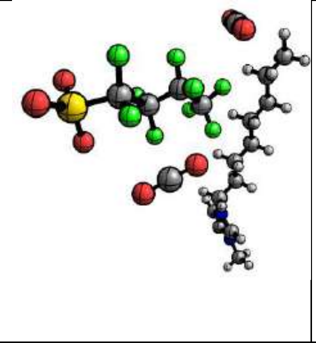	
10.22	10.81	12.67	13.41	

Table S60. Representations of lowest energy forms for $3CO_2[Omim]^+[(PFBu)SO_3]^-$ at the M06-2X-D3/6-31G(d,p) level with an implicit solvent model PCM. Relative energies are listed in kcal/mol.

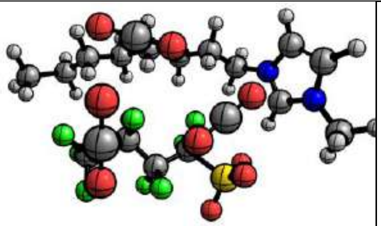	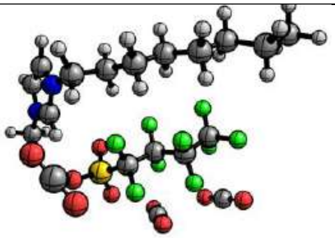	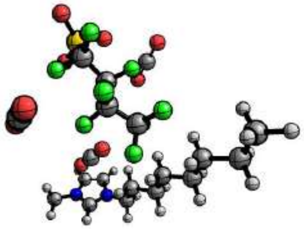	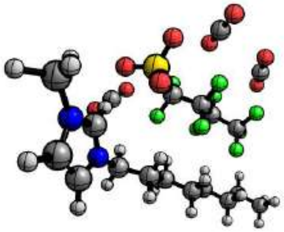	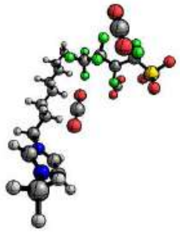
0.00	3.72	4.34	4.89	5.43
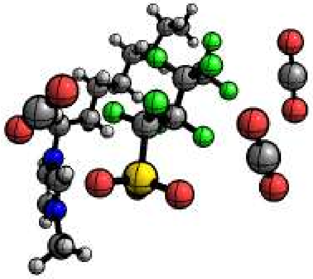	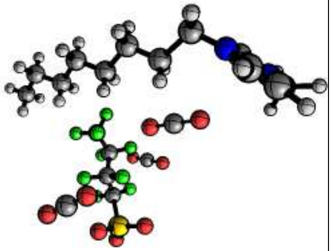	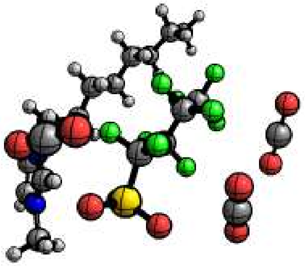	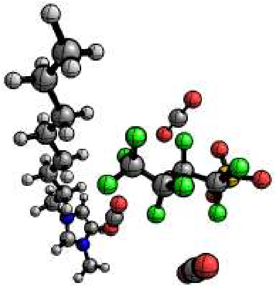	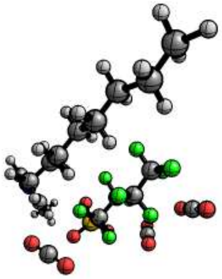
5.49	5.58	5.71	6.00	6.08
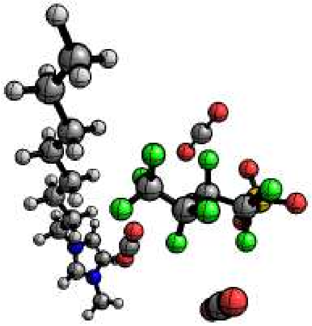	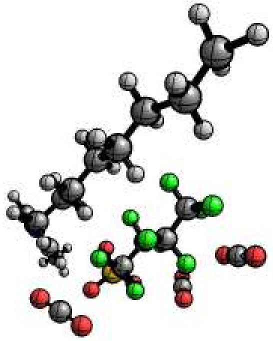	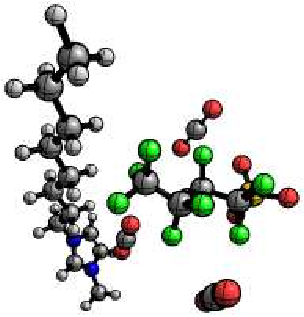	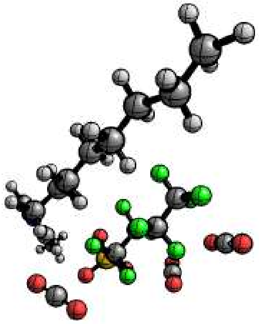	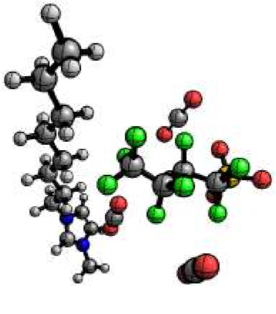
6.48	6.55	6.96	7.12	7.28

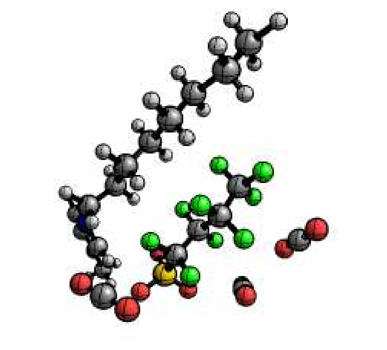	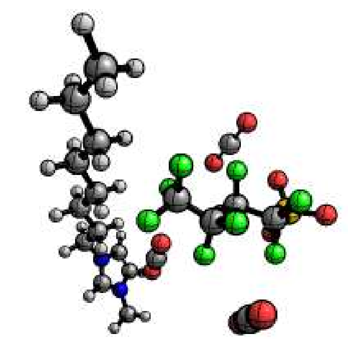	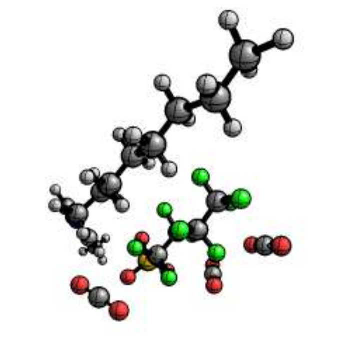	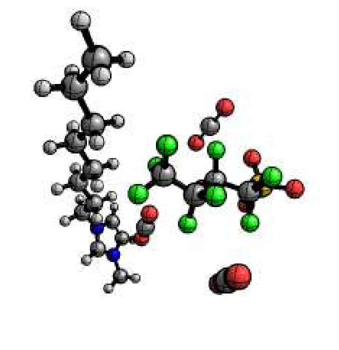	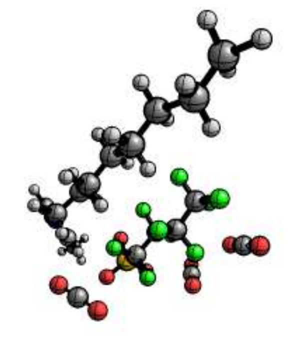
7.29	7.40	7.42	7.61	8.12
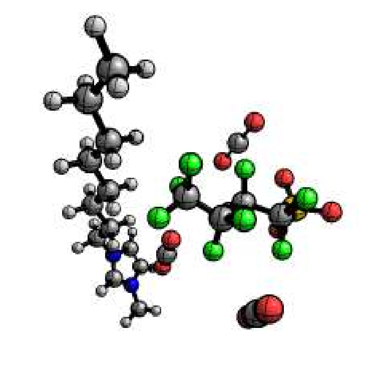	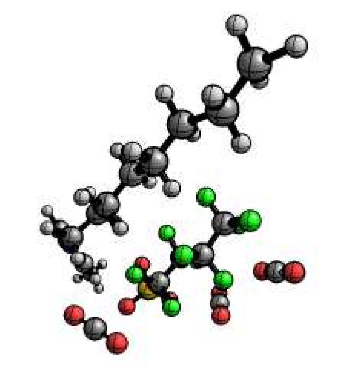	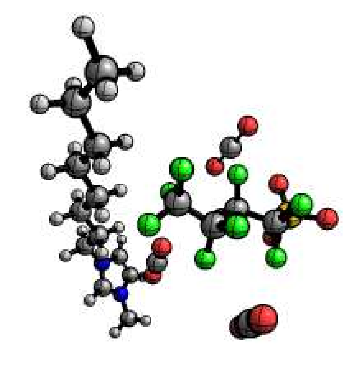	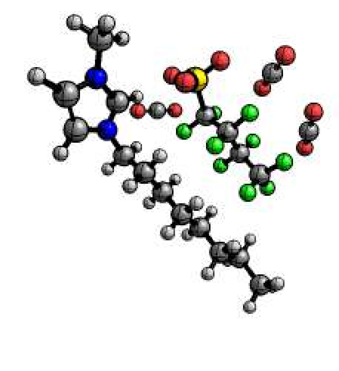	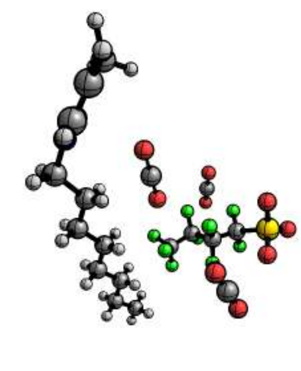
8.27	8.39	8.95	9.49	9.67
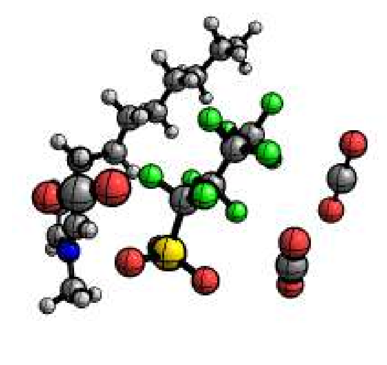	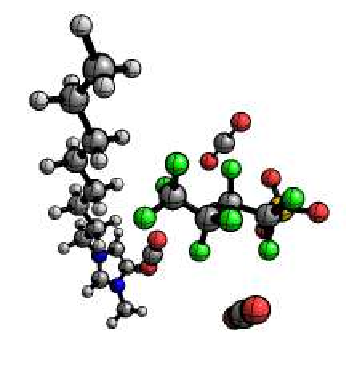	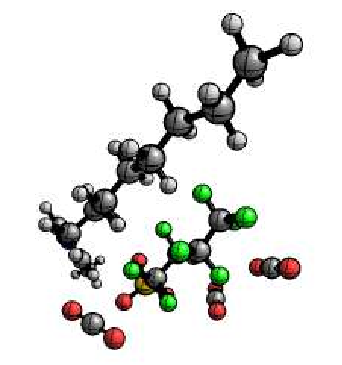	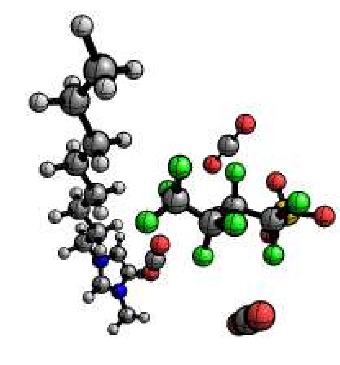	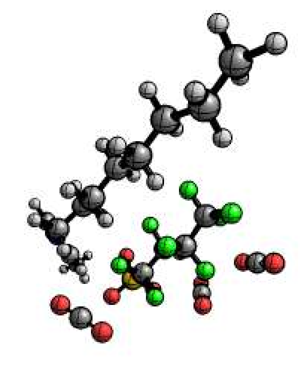

10.22	10.34	11.18	11.52	12.67
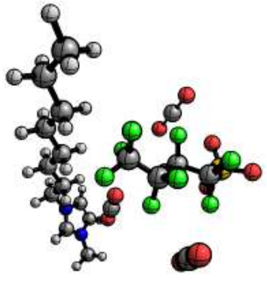	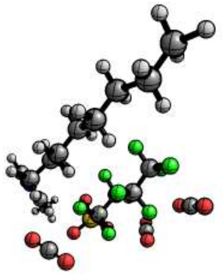	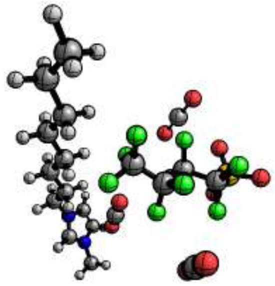		
13.08	13.23	14.72		

Table S61. Representations of lowest energy forms for $4CO_2[Omim]^+[(PFBu)SO_3]^-$ at the M06-2X-D3/6-31G(d,p) level with an implicit solvent model PCM. Relative energies are listed in kcal/mol.

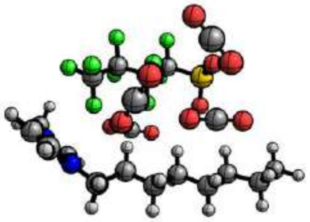	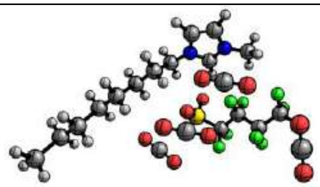	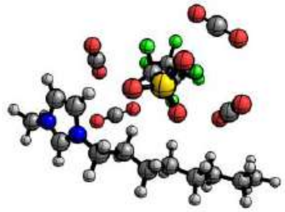	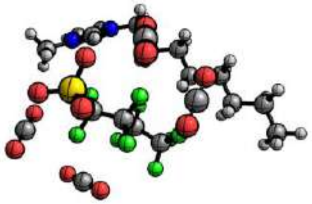	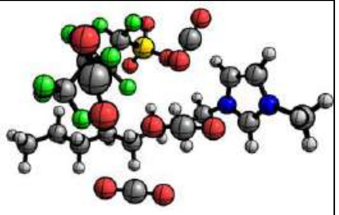
0.00	1.54	1.73	1.74	1.98
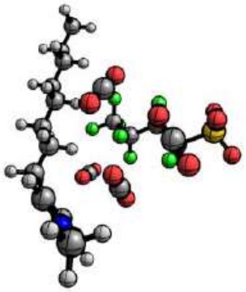	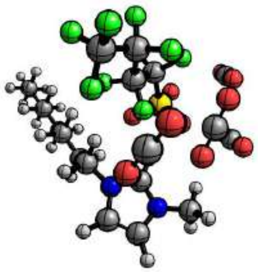	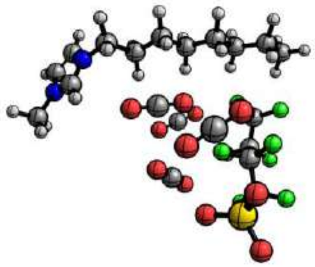	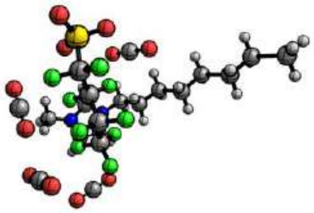	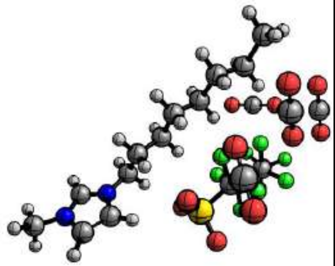
2.81	2.82	3.18	3.66	3.83

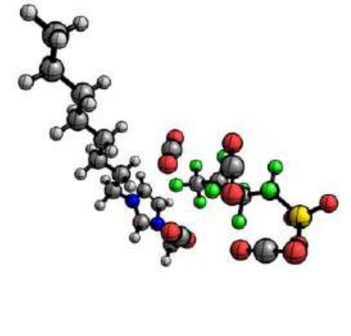	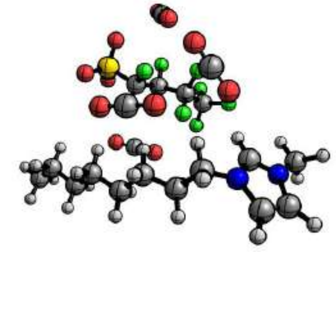	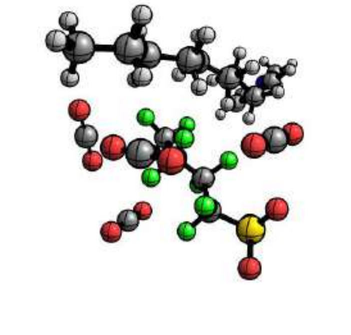	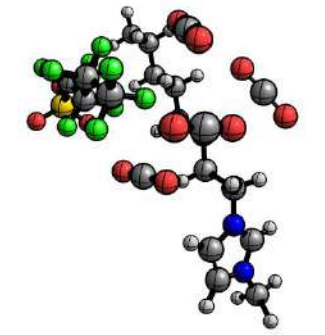	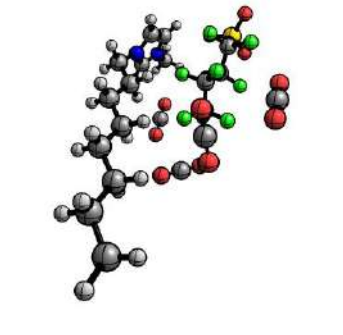
3.88	4.39	4.59	4.73	4.92
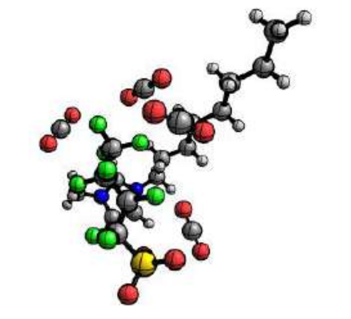	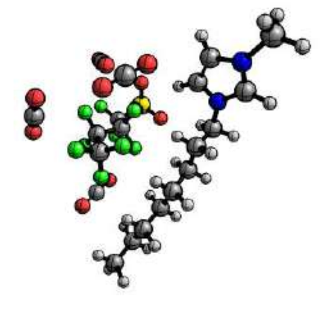	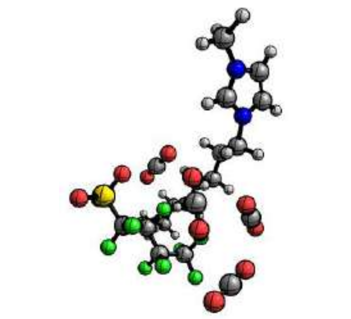	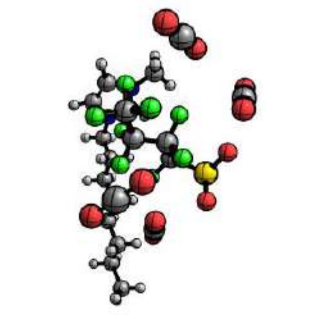	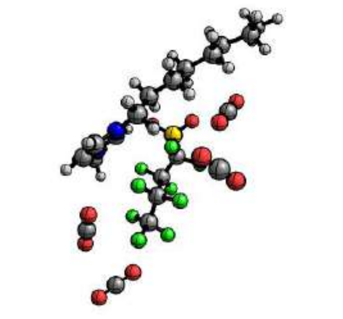
5.18	5.23	5.69	5.98	6.08
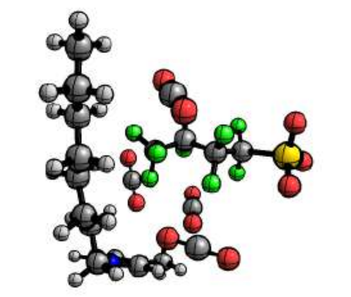	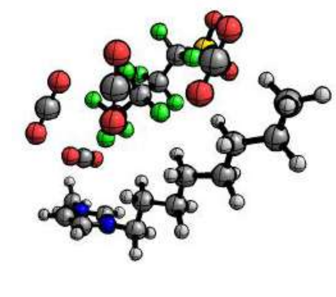	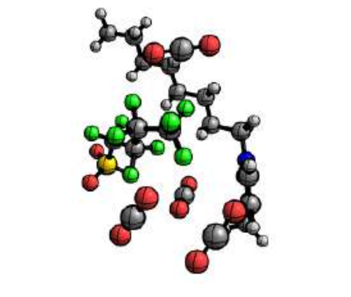	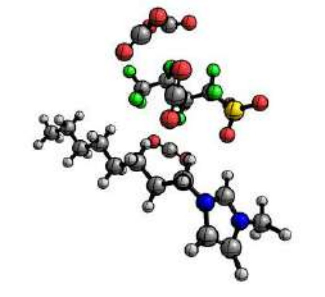	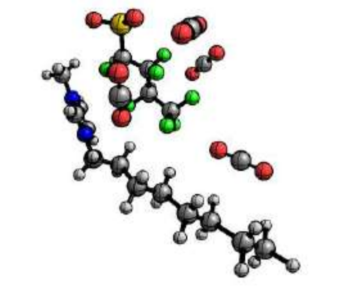
6.18	6.57	6.62	7.01	7.45

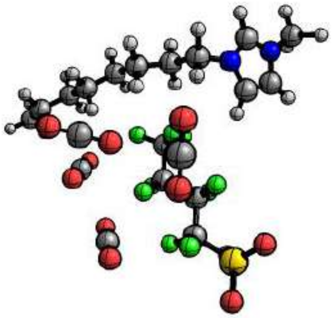	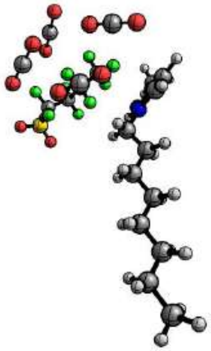	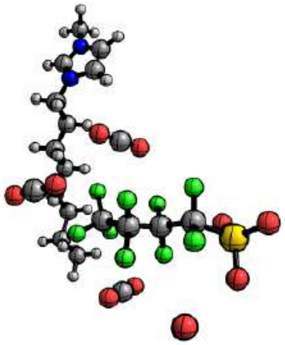	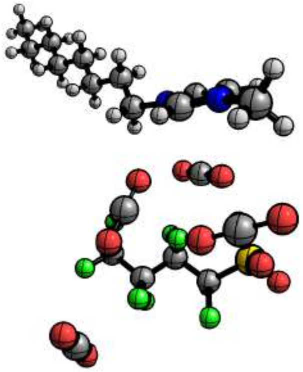	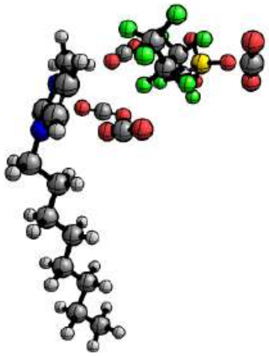
7.72	8.26	8.37	9.65	9.95
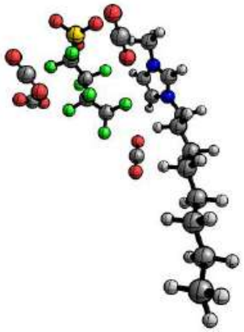	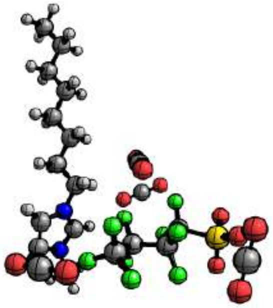	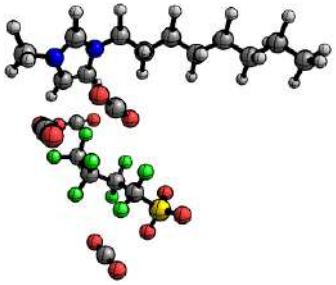		
9.99	10.08	11.32		

Table S62. Representations of lowest energy forms for $5CO_2[Omim]^+[(PFBu)SO_3]^-$ at the M06-2X-D3/6-31G(d,p) level with an implicit solvent model PCM. Relative energies are listed in kcal/mol.

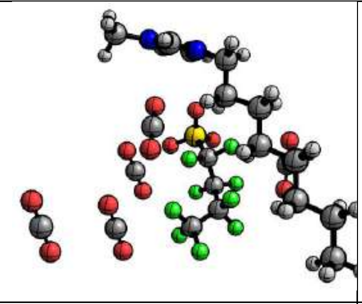	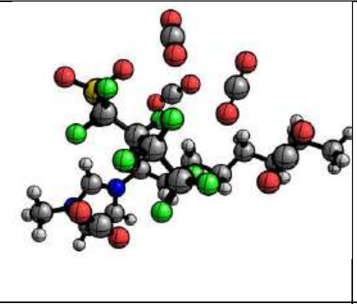	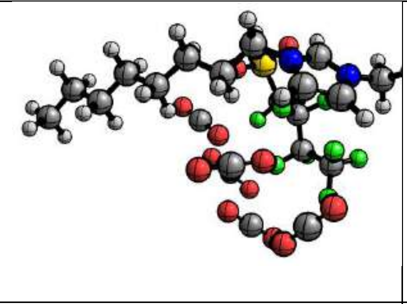	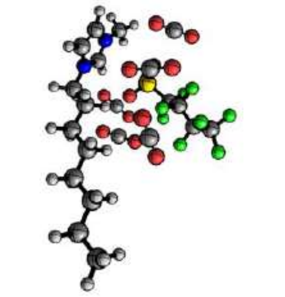	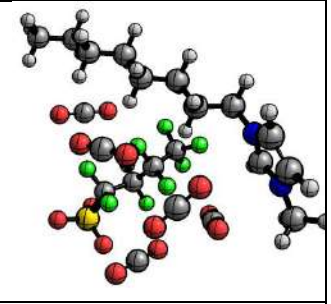
0.00	0.20	0.84	1.08	1.35
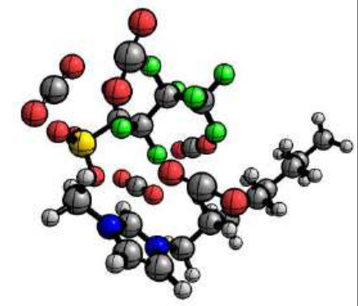	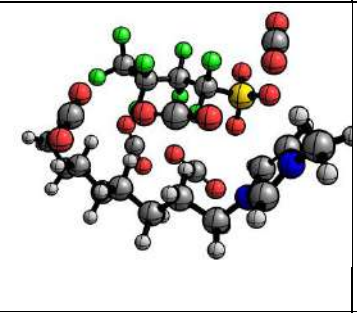	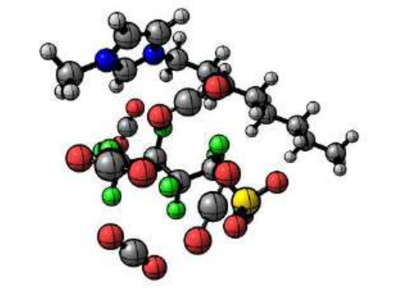	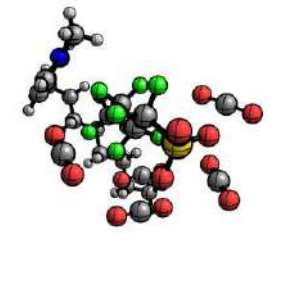	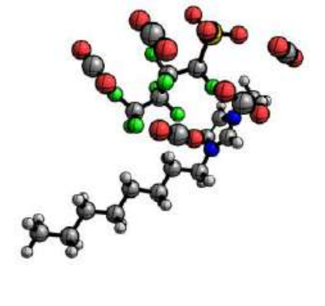
1.56	1.72	2.04	2.73	2.77
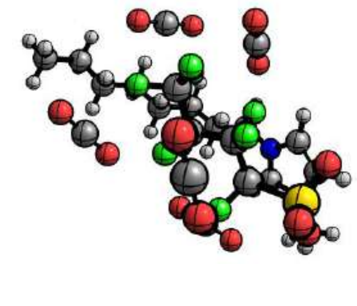	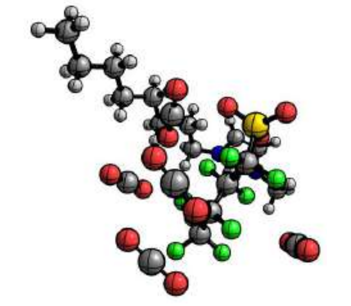	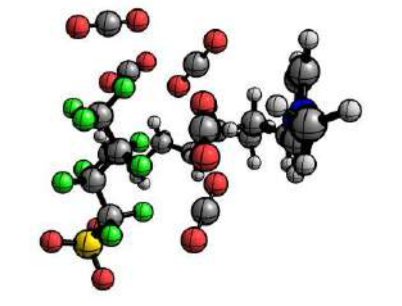	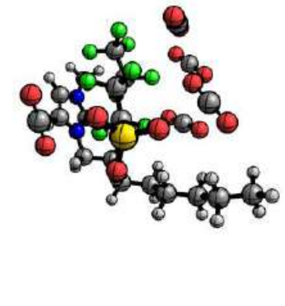	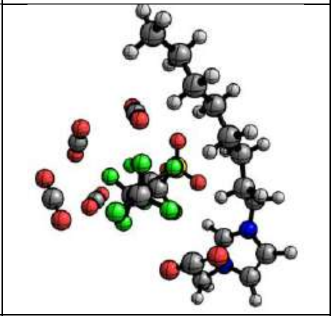
3.63	3.93	4.30	4.33	4.53

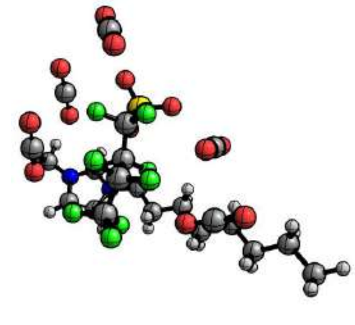	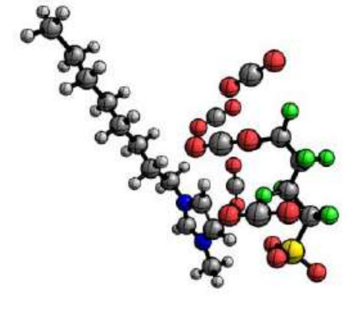	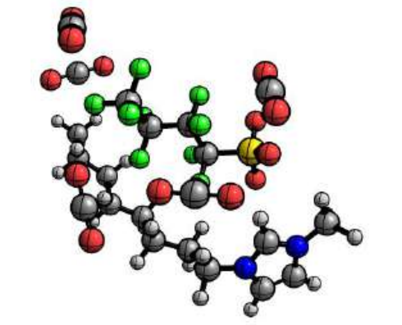	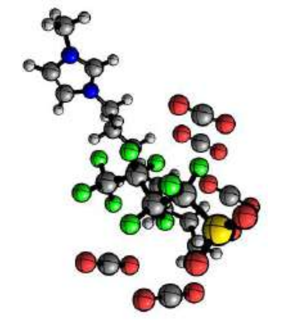	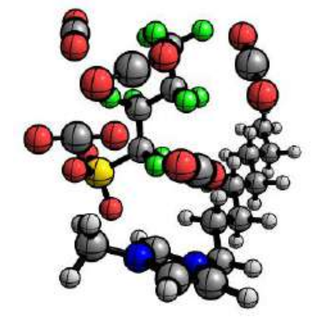
5.55	5.78	6.10	6.26	6.37
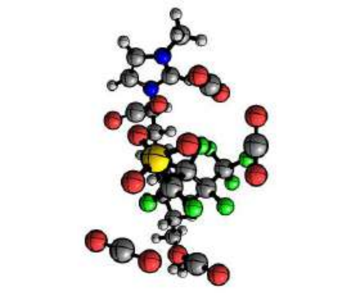	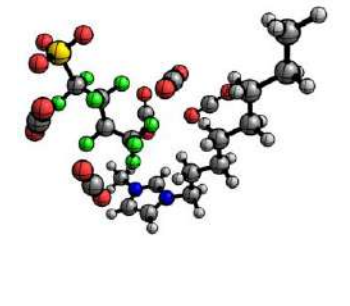	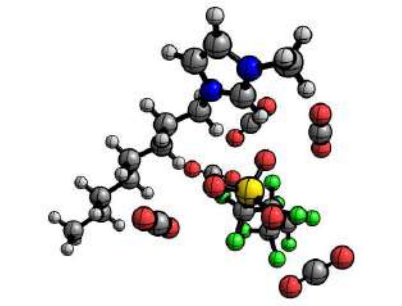	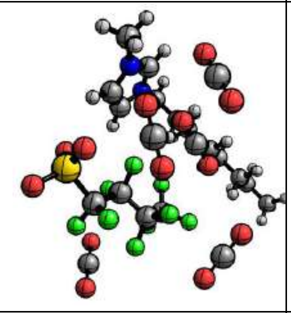	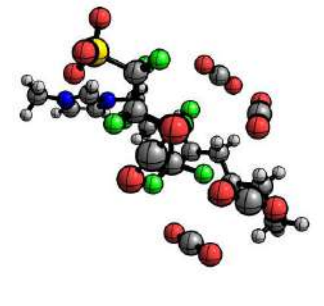
6.50	6.86	7.11	7.12	8.61
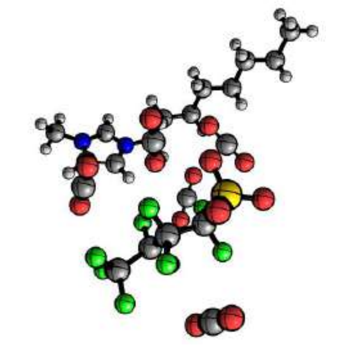	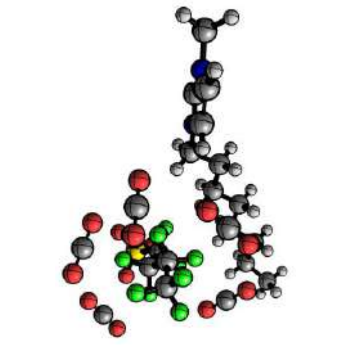	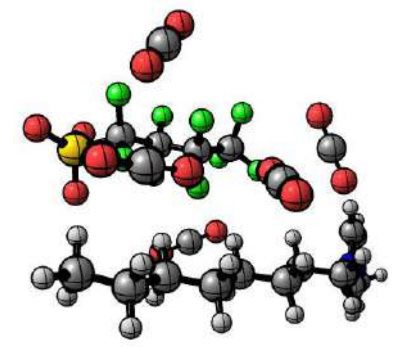	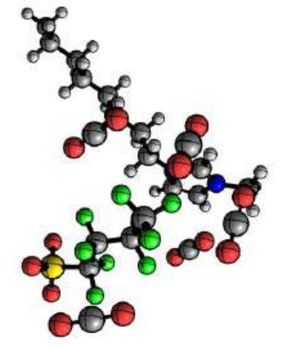	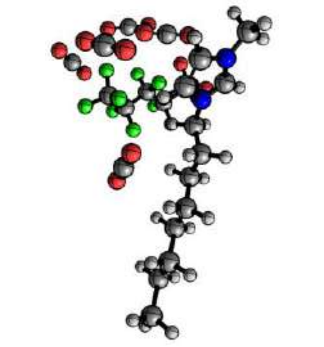
9.19	9.68	10.05	10.14	11.20

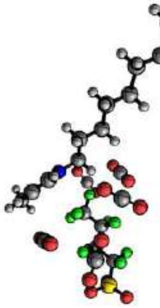	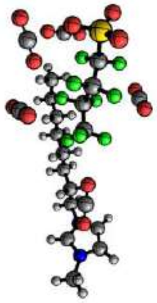	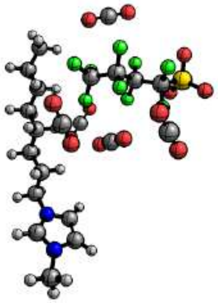	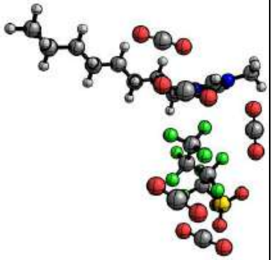	
12.84	12.85	13.83	13.91	

Table S63. Stabilization order of the clusters by number of CO_2 molecules.

ΔE	ΔH
**1CO$_2$**	**1CO$_2$**
[Dbim]$^+$[FAP]$^-$>[C$_8$H$_4$F$_{13}$mim]$^+$[TFO]$^-$> [Dbim]$^+$[Methide]$^-$ >[Hmim]$^+$[FAP]$^-$>[Hmim]$^+$[(PFOc)SO$_3$]$^-$>[Omim]$^+$[(PFOc)SO$_3$]$^-$> [Hmim]$^+$[Methide]$^-$>[Omim]$^+$[(PFBu)SO$_3$]$^-$>[Dmim]$^+$[TFO]$^-$ > [Dmim]$^+$[BF$_4$]$^-$>[C$_8$H$_4$F$_{13}$mim]$^+$[BF$_4$]$^-$>[Hmim]$^+$[(PFBu)SO$_3$]$^-$	[Dbim]$^+$[FAP]$^-$> [Dbim]$^+$[Methide]$^-$>[C$_8$H$_4$F$_{13}$mim]$^+$[TFO]$^-$> [Hmim]$^+$[Methide]$^-$>[Hmim]$^+$[FAP]$^-$>[Hmim]$^+$[(PFOc)SO$_3$]$^-$ >[Omim]$^+$[(PFOc)SO$_3$]$^-$> [Omim]$^+$[(PFBu)SO$_3$]$^-$>[Dmim]$^+$[TFO]$^-$ > [Dmim]$^+$[BF$_4$]$^-$>[C$_8$H$_4$F$_{13}$mim]$^+$[BF$_4$]$^-$>[Hmim]$^+$[(PFBu)SO$_3$]$^-$
**2CO$_2$**	**2CO$_2$**
[Hmim]$^+$[FAP]$^-$>[Dbim]$^+$[FAP]$^-$>[C$_8$H$_4$F$_{13}$mim]$^+$[TFO]$^-$>[Omim]$^+$[(PFBu)SO$_3$]$^-$>[Hmim]$^+$[(PFOc)SO$_3$]$^-$>[Omim]$^+$[(PFOc)SO$_3$]$^-$> [Dbim]$^+$[Methide]$^-$> [C$_8$H$_4$F$_{13}$mim]$^+$[BF$_4$]$^-$> [Dmim]$^+$[TFO]$^-$> [Dmim]$^+$[BF$_4$]$^-$> [Hmim]$^+$[Methide]$^-$> [Hmim]$^+$[(PFBu)SO$_3$]$^-$	[Hmim]$^+$[FAP]$^-$>[Dbim]$^+$[FAP]$^-$>[C$_8$H$_4$F$_{13}$mim]$^+$[TFO]$^-$>[Omim]$^+$[(PFBu)SO$_3$]$^-$>[Omim]$^+$[(PFOc)SO$_3$]$^-$>[Hmim]$^+$[(PFOc)SO$_3$]$^-$> [Dbim]$^+$[Methide]$^-$> [C$_8$H$_4$F$_{13}$mim]$^+$[BF$_4$]$^-$> [Dmim]$^+$[TFO]$^-$> [Dmim]$^+$[BF$_4$]$^-$> [Hmim]$^+$[Methide]$^-$> [Hmim]$^+$[(PFBu)SO$_3$]$^-$
**3CO$_2$**	**3CO$_2$**
[C$_8$H$_4$F$_{13}$mim]$^+$[BF$_4$]$^-$> [Dbim]$^+$[FAP]$^-$> [Omim]$^+$[(PFBu)SO$_3$]$^-$> [C$_8$H$_4$F$_{13}$mim]$^+$[TFO]$^-$> [Dbim]$^+$[Methide]$^-$> [Dmim]$^+$[BF$_4$]$^-$> [Omim]$^+$-[(PFOc)SO$_3$]$^-$> [Hmim]$^+$[(PFBu)SO$_3$]$^-$> [Hmim]$^+$[Methide]$^-$> [Dmim]$^+$[TFO]$^-$>[Hmim]$^+$[(PFOc)SO$_3$]$^-$> [Hmim]$^+$[FAP]$^-$	[C$_8$H$_4$F$_{13}$mim]$^+$[BF$_4$]$^-$> [Dbim]$^+$[FAP]$^-$> [Omim]$^+$[(PFBu)SO$_3$]$^-$> [C$_8$H$_4$F$_{13}$mim]$^+$[TFO]$^-$> [Dbim]$^+$[Methide]$^-$> [Dmim]$^+$[BF$_4$]$^-$> [Omim]$^+$[(PFOc)SO$_3$]$^-$> [Hmim]$^+$[(PFBu)SO$_3$]$^-$> [Hmim]$^+$[Methide]$^-$> [Dmim]$^+$[TFO]$^-$>[Hmim]$^+$[(PFOc)SO$_3$]$^-$> [Hmim]$^+$[FAP]$^-$
**4CO$_2$**	**4CO$_2$**
[Dbim]$^+$[FAP]$^-$> [Hmim]$^+$[FAP]$^-$> [C$_8$H$_4$F$_{13}$mim]$^+$[BF$_4$]$^-$> [C$_8$H$_4$F$_{13}$mim]$^+$[TFO]$^-$> [Dbim]$^+$[Methide]$^-$> [Dmim]$^+$[BF$_4$]$^-$> [Hmim]$^+$[Methide]$^-$> [Omim]$^+$[(PFBu)SO$_3$]$^-$> [Dmim]$^+$[TFO]$^-$>	[Dbim]$^+$[FAP]$^-$> [Hmim]$^+$[FAP]$^-$> [C$_8$H$_4$F$_{13}$mim]$^+$[BF$_4$]$^-$> [C$_8$H$_4$F$_{13}$mim]$^+$[TFO]$^-$> [Dbim]$^+$ [Methide]$^-$> [Dmim]$^+$[BF$_4$]$^-$>

[Hmim]$^+$[(PFBu)SO$_3$]$^-$> [Hmim]$^+$[(PFOc)SO$_3$]$^-$ >[Omim]$^+$[(PFOc)SO$_3$]$^-$	[Hmim]$^+$[Methide]$^-$> [Omim]$^+$[(PFBu)SO$_3$]$^-$> [Dmim]$^+$[TFO]$^-$> [Hmim]$^+$[(PFBu)SO$_3$]$^-$> [Hmim]$^+$[(PFOc)SO$_3$]$^-$>[Omim]$^+$[(PFOc)SO$_3$]$^-$
**5CO_2**	**5CO_2**
[Dbim]$^+$[FAP]$^-$>[Hmim]$^+$[FAP]$^-$> [C$_8$H$_4$F$_{13}$mim]$^+$[BF$_4$]$^-$> [Hmim]$^+$[Methide]$^-$ > [C$_8$H$_4$F$_{13}$mim]$^+$[TFO]$^-$> [Dmim]$^+$[BF$_4$]$^-$> [Dmim]$^+$[TFO]$^-$> [Hmim]$^+$[(PFOc)SO$_3$]$^-$> [Hmim]$^+$[(PFBu)SO$_3$]$^-$> [Dbim]$^+$[Methide]$^-$> [Omim]$^+$[(PFOc)SO$_3$]$^-$> [Omim]$^+$[(PFBu)SO$_3$]$^-$	[Dbim]$^+$[FAP]$^-$> [Hmim]$^+$[FAP]$^-$> [C$_8$H$_4$F$_{13}$mim]$^+$[BF$_4$]$^-$> [Hmim]$^+$[Methide]$^-$ > [C$_8$H$_4$F$_{13}$mim]$^+$[TFO]$^-$> [Dmim]$^+$[BF$_4$]$^-$> [Dmim]$^+$[TFO]$^-$> [Hmim]$^+$[(PFOc)SO$_3$]$^-$> [Hmim]$^+$[(PFBu)SO$_3$]$^-$> [Dbim]$^+$[Methide]$^-$> [Omim]$^+$[(PFOc)SO$_3$]$^-$> [Omim]$^+$ [(PFBu)SO$_3$]$^-$

Table S64. Topological parameters, where BT = bond type, IN = interaction number, $\rho(r_{cp})$ and $\nabla^2\rho(r_{cp})$ in a.u..

N° CO_2	Compound	BT	IT	IN	$\rho(r_{cp})$ 10^{-2}	10^{-2}	H 10^{-3}	V/G	IE kcal/mol
1CO_2	[Dmim]$^+$[BF$_4$]$^-$	Anion-Cation	C-H---F	2	1.05	4.14	1.64	0.81	-2.22
1CO_2	[Dmim]$^+$[BF$_4$]$^-$	Anion-Cation	C---F	3	0.56	2.30	1.02	0.78	-1.17
1CO_2	[Dmim]$^+$[BF$_4$]$^-$	Anion-CO_2	C---F	2	1.15	4.87	1.45	0.86	-2.91
1CO_2	[Dmim]$^+$[BF$_4$]$^-$	Cation-CO_2	N---O	1	0.74	3.33	1.91	0.70	-1.41
1CO_2	[Dmim]$^+$[TFO]$^-$	Anion-Cation	C---F	2	0.55	2.26	1.07	0.77	-1.10
1CO_2	[Dmim]$^+$[TFO]$^-$	Anion-Cation	C---O	1	0.54	2.24	1.12	0.75	-1.05
1CO_2	[Dmim]$^+$[TFO]$^-$	Anion-Cation	N---O	1	0.54	1.82	0.89	0.76	-0.87
1CO_2	[Dmim]$^+$[TFO]$^-$	Anion-Cation	F---N	1	0.94	4.29	2.15	0.75	-2.02
1CO_2	[Dmim]$^+$[TFO]$^-$	Anion-CO_2	C---O	1	0.63	2.36	1.22	0.74	-1.09
1CO_2	[Dmim]$^+$[TFO]$^-$	Anion-CO_2	O---O	1	0.54	2.01	0.94	0.77	-0.99
1CO_2	[Dmim]$^+$[TFO]$^-$	Anion-CO_2	F---O	1	0.77	3.57	1.81	0.75	-1.66
1CO_2	[Dmim]$^+$[TFO]$^-$	Cation-CO_2	C-H---O	1	0.69	2.69	1.45	0.73	-1.20

1CO_2	$[Dmim]^+[TFO]^-$	Cation-CO_2	C---O	1	0.92	3.46	1.23	0.83	-1.94
1CO_2	$[C_8H_4F_{13}mim]^+[BF_4]^-$	Anion-Cation	C-H---F	5	0.90	3.52	1.44	0.80	-1.86
1CO_2	$[C_8H_4F_{13}mim]^+[BF_4]^-$	Anion-Cation	C---F	2	1.03	4.23	1.63	0.82	-2.29
1CO_2	$[C_8H_4F_{13}mim]^+[BF_4]^-$	Anion-Cation	F---F	10	0.82	3.99	1.84	0.77	-1.98
1CO_2	$[C_8H_4F_{13}mim]^+[BF_4]^-$	Anion-CO_2	C---F	1	1.36	5.95	1.83	0.86	-3.52
1CO_2	$[C_8H_4F_{13}mim]^+[BF_4]^-$	Cation-CO_2	C-H---O	1	0.82	3.21	1.68	0.74	-1.46
1CO_2	$[C_8H_4F_{13}mim]^+[BF_4]^-$	Cation-CO_2	C---O	2	0.51	2.05	1.10	0.73	-0.92
1CO_2	$[C_8H_4F_{13}mim]^+[BF_4]^-$	Cation-CO_2	N---C	1	0.67	2.65	1.27	0.76	-1.28
1CO_2	$[C_8H_4F_{13}mim]^+[TFO]^-$	Anion-Cation	C-H---F	3	0.72	2.79	1.21	0.79	-1.43
1CO_2	$[C_8H_4F_{13}mim]^+[TFO]^-$	Anion-Cation	C-H---O	1	0.82	2.79	1.25	0.78	-1.40
1CO_2	$[C_8H_4F_{13}mim]^+[TFO]^-$	Anion-Cation	C---F	2	0.85	3.78	1.68	0.78	-1.91
1CO_2	$[C_8H_4F_{13}mim]^+[TFO]^-$	Anion-Cation	C---O	2	0.70	2.53	1.10	0.79	-1.29
1CO_2	$[C_8H_4F_{13}mim]^+[TFO]^-$	Anion-Cation	F---F	7	0.94	4.56	2.11	0.77	-2.25
1CO_2	$[C_8H_4F_{13}mim]^+[TFO]^-$	Anion-CO_2	C---O	1	1.13	4.39	1.42	0.85	-2.55
1CO_2	$[C_8H_4F_{13}mim]^+[TFO]^-$	Anion-CO_2	F---O	1	0.89	3.94	1.53	0.82	-2.14
1CO_2	$[C_8H_4F_{13}mim]^+[TFO]^-$	Cation-CO_2	C-H---O	3	0.71	2.80	1.47	0.73	-1.27
1CO_2	$[C_8H_4F_{13}mim]^+[TFO]^-$	Cation-CO_2	F---O	1	0.83	4.24	2.12	0.75	-1.99
1CO_2	$[Dbim]^+[FAP]^-$	Anion-Cation	C-H---F	9	0.52	2.09	0.95	0.77	-1.04
1CO_2	$[Dbim]^+[FAP]^-$	Anion-Cation	C---F	1	0.42	1.59	0.77	0.76	-0.76

$1CO_2$	$[Dbim]^+[FAP]^-$	Anion-Cation	F---F	8	1.32	6.97	3.22	0.78	-3.45
$1CO_2$	$[Dbim]^+[FAP]^-$	Anion-Cation	F---N	1	1.04	4.94	2.40	0.76	-2.37
$1CO_2$	$[Dbim]^+[FAP]^-$	Anion-CO_2	C---F	1	0.82	3.68	1.34	0.83	-2.05
$1CO_2$	$[Dbim]^+[FAP]^-$	Anion-CO_2	F---O	4	0.63	2.76	1.23	0.78	-1.39
$1CO_2$	$[Dbim]^+[FAP]^-$	Cation-CO_2	C-H---O	3	0.58	2.24	1.19	0.73	-1.01
$1CO_2$	$[Dbim]^+[Methide]^-$	Anion-Cation	C-H---F	9	0.52	2.08	0.95	0.77	-1.03
$1CO_2$	$[Dbim]^+[Methide]^-$	Anion-Cation	C-H---O	2	0.51	1.94	0.91	0.75	-0.95
$1CO_2$	$[Dbim]^+[Methide]^-$	Anion-Cation	O---O	1	1.20	5.06	2.26	0.78	-2.55
$1CO_2$	$[Dbim]^+[Methide]^-$	Anion-Cation	F---O	3	0.87	3.71	1.66	0.78	-1.87
$1CO_2$	$[Dbim]^+[Methide]^-$	Anion-Cation	C---F	3	0.70	2.89	1.27	0.78	-1.47
$1CO_2$	$[Dbim]^+[Methide]^-$	Anion-Cation	N---F	1	0.58	2.64	1.32	0.75	-1.25
$1CO_2$	$[Dbim]^+[Methide]^-$	Anion-Cation	F---F	2	0.71	3.36	1.50	0.79	-1.70
$1CO_2$	$[Dbim]^+[Methide]^-$	Anion-CO_2	O---O	1	0.31	1.17	0.56	0.76	-0.56
$1CO_2$	$[Dbim]^+[Methide]^-$	Anion-CO_2	F---O	2	0.53	2.28	1.08	0.77	-1.11
$1CO_2$	$[Dbim]^+[Methide]^-$	Anion-CO_2	C---F	1	0.82	3.94	1.62	0.80	-2.08
$1CO_2$	$[Dbim]^+[Methide]^-$	Anion-CO_2	C---O	1	1.07	4.55	1.69	0.83	-2.51
$1CO_2$	$[Dbim]^+[Methide]^-$	Cation-CO_2	C-H---O	1	0.23	0.80	0.43	0.73	-0.36
$1CO_2$	$[Hmim]^+[FAP]^-$	Anion-Cation	C-H---F	9	0.60	2.34	1.00	0.79	-1.20
$1CO_2$	$[Hmim]^+[FAP]^-$	Anion-Cation	C---F	5	0.67	2.93	1.30	0.76	-1.49

$1CO_2$	$[Hmim]^+[FAP]^-$	Anion-Cation	F---F	9	1.29	6.83	3.14	0.78	-3.39
$1CO_2$	$[Hmim]^+[FAP]^-$	Anion-Cation	F---N	1	0.71	3.26	1.68	0.74	-1.50
$1CO_2$	$[Hmim]^+[FAP]^-$	Anion-CO_2	F---O	5	0.51	2.26	1.05	0.77	-1.11
$1CO_2$	$[Hmim]^+[Methide]^-$	Anion-Cation	C-H---F	4	0.55	2.31	1.08	0.76	-1.13
$1CO_2$	$[Hmim]^+[Methide]^-$	Anion-Cation	C-H---S	1	0.23	0.69	0.43	0.67	-0.27
$1CO_2$	$[Hmim]^+[Methide]^-$	Anion-Cation	C---O	2	0.53	2.02	1.01	0.75	-0.95
$1CO_2$	$[Hmim]^+[Methide]^-$	Anion-Cation	F---O	1	0.55	2.35	1.14	0.76	-1.13
$1CO_2$	$[Hmim]^+[Methide]^-$	Anion-Cation	S---F	2	0.86	3.41	1.33	0.81	-1.84
$1CO_2$	$[Hmim]^+[Methide]^-$	Anion-Cation	C---F	1	0.77	2.90	1.19	0.80	-1.53
$1CO_2$	$[Hmim]^+[Methide]^-$	Anion-Cation	F---F	1	0.89	4.40	2.10	0.76	-2.13
$1CO_2$	$[Hmim]^+[Methide]^-$	Anion-Cation	N---F	1	0.92	4.40	2.18	0.75	-2.08
$1CO_2$	$[Hmim]^+[Methide]^-$	Anion-CO_2	F---O	2	0.71	3.07	1.45	0.77	-1.50
$1CO_2$	$[Hmim]^+[Methide]^-$	Anion-CO_2	O---O	1	0.73	2.87	1.23	0.79	-1.48
$1CO_2$	$[Hmim]^+[Methide]^-$	Anion-CO_2	C---F	1	0.91	4.05	1.47	0.83	-2.25
$1CO_2$	$[Hmim]^+[Methide]^-$	Anion-CO_2	S---O	1	0.93	3.56	1.74	0.76	-1.70
$1CO_2$	$[Hmim]^+[Methide]^-$	Cation-CO_2	C-H---O	1	0.37	1.29	0.67	0.74	-0.59
$1CO_2$	$[Hmim]^+[Methide]^-$	Cation-CO_2	C-H---C	1	0.48	1.81	0.89	0.76	-0.86
$1CO_2$	$[Hmim]^+[(PFOc)SO_3]^-$	Anion-Cation	C-H---F	6	0.62	2.49	1.08	0.79	-1.27
$1CO_2$	$[Hmim]^+[(PFOc)SO_3]^-$	Anion-Cation	C-H---O	3	0.45	1.54	0.71	0.77	-0.76

1CO_2	$[Hmim]^+[(PFOc)SO_3]^-$	Anion-Cation	C---F	3	0.79	3.61	1.69	0.77	-1.77
1CO_2	$[Hmim]^+[(PFOc)SO_3]^-$	Anion-CO_2	F---O	3	0.50	2.20	1.03	0.77	-1.08
1CO_2	$[Hmim]^+[(PFOc)SO_3]^-$	Anion-CO_2	C---F	1	0.85	4.02	1.64	0.81	-2.13
1CO_2	$[Hmim]^+[(PFOc)SO_3]^-$	Cation-CO_2	C-H---O	3	0.50	1.90	1.00	0.73	-0.86
1CO_2	$[Omim]^+[(PFOc)SO_3]^-$	Anion-Cation	C-H---F	12	0.59	2.34	1.05	0.78	-1.18
1CO_2	$[Omim]^+[(PFOc)SO_3]^-$	Anion-Cation	C-H---O	1	0.67	2.22	0.93	0.80	-1.16
1CO_2	$[Omim]^+[(PFOc)SO_3]^-$	Anion-Cation	C---F	3	0.54	2.21	1.05	0.76	-1.07
1CO_2	$[Omim]^+[(PFOc)SO_3]^-$	Anion-Cation	F---F	2	1.25	6.79	3.29	0.76	-3.26
1CO_2	$[Omim]^+[(PFOc)SO_3]^-$	Anion-Cation	C---O	1	0.65	2.48	1.15	0.77	-1.22
1CO_2	$[Omim]^+[(PFOc)SO_3]^-$	Anion-CO_2	F---O	3	0.62	2.79	1.31	0.77	-1.36
1CO_2	$[Omim]^+[(PFOc)SO_3]^-$	Anion-CO_2	C---F	1	0.72	3.24	1.30	0.81	-1.72
1CO_2	$[Omim]^+[(PFOc)SO_3]^-$	Cation-CO_2	C-H---O	4	0.54	2.05	1.06	0.74	-0.94
1CO_2	$[Hmim]^+[(PFBu)SO_3]^-$	Anion-Cation	C-H---F	8	0.58	2.31	1.04	0.78	-1.17
1CO_2	$[Hmim]^+[(PFBu)SO_3]^-$	Anion-Cation	C-H---O	1	0.67	2.45	1.18	0.76	-1.18
1CO_2	$[Hmim]^+[(PFBu)SO_3]^-$	Anion-Cation	C---F	4	0.65	2.82	1.28	0.77	-1.40
1CO_2	$[Hmim]^+[(PFBu)SO_3]^-$	Anion-CO_2	F---O	1	0.48	2.02	0.94	0.77	-1.00
1CO_2	$[Hmim]^+[(PFBu)SO_3]^-$	Anion-CO_2	C---F	1	0.93	4.24	1.64	0.82	-2.30
1CO_2	$[Hmim]^+[(PFBu)SO_3]^-$	Anion-CO_2	C---O	1	1.01	3.99	1.37	0.84	-2.27
1CO_2	$[Hmim]^+[(PFBu)SO_3]^-$	Anion-CO_2	O---O	1	0.57	2.09	0.94	0.78	-1.06
1CO_2	$[Hmim]^+[(PFBu)SO_3]^-$	Cation-CO_2	C-H---O	1	0.51	1.92	1.05	0.72	-0.85

$1CO_2$	$[Omim]^+[(PFBu)SO_3]^-$	Anion-Cation	C-H---F	10	0.57	2.41	1.10	0.77	-1.20
$1CO_2$	$[Omim]^+[(PFBu)SO_3]^-$	Anion-Cation	C-H---O	2	0.84	3.28	1.47	0.75	-1.65
$1CO_2$	$[Omim]^+[(PFBu)SO_3]^-$	Anion-Cation	F---O	1	1.06	4.81	2.11	0.79	-2.45
$1CO_2$	$[Omim]^+[(PFBu)SO_3]^-$	Anion-Cation	C---O	1	0.64	2.58	1.14	0.78	-1.31
$1CO_2$	$[Omim]^+[(PFBu)SO_3]^-$	Anion-Cation	F---F	1	1.24	6.75	3.20	0.77	-3.29
$1CO_2$	$[Omim]^+[(PFBu)SO_3]^-$	Anion-CO_2	F---O	2	0.43	1.82	0.85	0.77	-0.90
$1CO_2$	$[Omim]^+[(PFBu)SO_3]^-$	Anion-CO_2	C---F	2	0.82	3.74	1.44	0.82	-2.03
$1CO_2$	$[Omim]^+[(PFBu)SO_3]^-$	Anion-CO_2	O---O	1	0.42	1.50	0.67	0.78	-0.75
$1CO_2$	$[Omim]^+[(PFBu)SO_3]^-$	Cation-CO_2	C-H---O	1	0.69	2.49	1.24	0.75	-1.18
$1CO_2$	$[Omim]^+[(PFBu)SO_3]^-$	Cation-CO_2	N---O	1	0.80	3.54	2.04	0.70	-1.50
$2CO_2$	$[Dmim]^+[BF_4]^-$	Anion-Cation	C-H---F	4	0.87	3.43	1.41	0.80	-1.80
$2CO_2$	$[Dmim]^+[BF_4]^-$	Anion-CO_2	C---F	4	1.11	4.71	1.43	0.86	-2.80
$2CO_2$	$[Dmim]^+[BF_4]^-$	Cation-CO_2	N---O	2	0.51	1.99	1.12	0.71	-0.86
$2CO_2$	$[Dmim]^+[BF_4]^-$	Cation-CO_2	C---O	2	0.67	2.75	1.41	0.74	-1.27
$2CO_2$	$[Dmim]^+[BF_4]^-$	CO_2-CO_2	O---O	1	0.61	2.64	1.46	0.71	-1.15
$2CO_2$	$[Dmim]^+[TFO]^-$	Anion-Cation	C-H---F	1	0.54	2.17	0.98	0.78	-1.09
$2CO_2$	$[Dmim]^+[TFO]^-$	Anion-Cation	C---F	1	0.60	2.34	1.05	0.78	-1.17
$2CO_2$	$[Dmim]^+[TFO]^-$	Anion-Cation	C---O	2	0.63	2.29	1.03	0.78	-1.14
$2CO_2$	$[Dmim]^+[TFO]^-$	Anion-Cation	F---N	1	0.82	3.91	2.00	0.74	-1.81
$2CO_2$	$[Dmim]^+[TFO]^-$	Anion-CO_2	C---O	3	1.06	4.24	1.45	0.84	-2.41

$2CO_2$	$[Dmim]^+[TFO]^-$	Anion-CO_2	F---O	2	0.59	2.53	1.19	0.77	-1.24
$2CO_2$	$[Dmim]^+[TFO]^-$	Cation-CO_2	C---O	2	0.62	2.51	1.35	0.73	-1.12
$2CO_2$	$[Dmim]^+[TFO]^-$	CO_2-CO_2	O---O	1	0.93	4.11	2.07	0.75	-1.92
$2CO_2$	$[C_8H_4F_{13}mim]^+[BF_4]^-$	Anion-Cation	C-H---F	4	0.94	3.83	1.54	0.80	-2.04
$2CO_2$	$[C_8H_4F_{13}mim]^+[BF_4]^-$	Anion-Cation	F---F	6	1.04	5.13	2.42	0.77	-2.51
$2CO_2$	$[C_8H_4F_{13}mim]^+[BF_4]^-$	Anion-CO_2	C---F	2	1.14	4.84	1.50	0.86	-2.86
$2CO_2$	$[C_8H_4F_{13}mim]^+[BF_4]^-$	Anion-CO_2	F---O	2	0.80	3.46	1.27	0.81	-1.92
$2CO_2$	$[C_8H_4F_{13}mim]^+[BF_4]^-$	Cation-CO_2	C-H---O	1	0.99	3.96	2.01	0.75	-1.85
$2CO_2$	$[C_8H_4F_{13}mim]^+[BF_4]^-$	Cation-CO_2	C---O	2	0.80	3.38	1.67	0.75	-1.60
$2CO_2$	$[C_8H_4F_{13}mim]^+[BF_4]^-$	Cation-CO_2	F---O	6	0.58	2.65	1.32	0.76	-1.25
$2CO_2$	$[C_8H_4F_{13}mim]^+[BF_4]^-$	CO_2-CO_2	O---O	1	0.96	4.26	2.11	0.75	-2.02
$2CO_2$	$[C_8H_4F_{13}mim]^+[TFO]^-$	Anion-Cation	C-H---F	2	0.55	2.27	1.11	0.73	-1.09
$2CO_2$	$[C_8H_4F_{13}mim]^+[TFO]^-$	Anion-Cation	C-H---O	2	0.90	3.32	1.40	0.80	-1.72
$2CO_2$	$[C_8H_4F_{13}mim]^+[TFO]^-$	Anion-Cation	C---F	1	1.01	4.49	1.93	0.79	-2.31
$2CO_2$	$[C_8H_4F_{13}mim]^+[TFO]^-$	Anion-Cation	C---O	1	1.15	4.17	1.53	0.83	-2.31
$2CO_2$	$[C_8H_4F_{13}mim]^+[TFO]^-$	Anion-Cation	F---O	5	0.57	2.47	1.13	0.78	-1.22
$2CO_2$	$[C_8H_4F_{13}mim]^+[TFO]^-$	Anion-Cation	F---F	6	1.01	4.96	2.32	0.77	-2.44
$2CO_2$	$[C_8H_4F_{13}mim]^+[TFO]^-$	Anion-CO_2	C---O	1	1.38	5.86	1.99	0.84	-3.35
$2CO_2$	$[C_8H_4F_{13}mim]^+[TFO]^-$	Anion-CO_2	O---O	2	0.70	2.67	1.15	0.79	-1.37
$2CO_2$	$[C_8H_4F_{13}mim]^+[TFO]^-$	Anion-CO_2	F---O	3	0.55	2.43	1.08	0.78	-1.23
$2CO_2$	$[C_8H_4F_{13}mim]^+[TFO]^-$	Anion-CO_2	C-H---O	1	0.66	2.53	1.33	0.73	-1.15

$2CO_2$	$[C_8H_4F_{13}mim]^+[TFO]^-$	Anion-CO_2	C---O	2	0.51	1.98	1.06	0.73	-0.88
$2CO_2$	$[C_8H_4F_{13}mim]^+[TFO]^-$	Anion-CO_2	N---O	1	0.59	2.35	1.25	0.73	-1.06
$2CO_2$	$[C_8H_4F_{13}mim]^+[TFO]^-$	Anion-CO_2	F---O	3	0.52	2.40	1.22	0.75	-1.12
$2CO_2$	$[C_8H_4F_{13}mim]^+[TFO]^-$	CO_2-CO_2	C---O	1	0.69	3.17	1.55	0.76	-1.51
$2CO_2$	$[Dbim]^+[FAP]^-$	Anion-Cation	C-H---F	10	0.68	2.81	1.30	0.77	-1.39
$2CO_2$	$[Dbim]^+[FAP]^-$	Anion-Cation	F---F	8	1.35	7.20	3.37	0.77	-3.53
$2CO_2$	$[Dbim]^+[FAP]^-$	Anion-CO_2	C---F	3	0.79	3.62	1.41	0.81	-1.95
$2CO_2$	$[Dbim]^+[FAP]^-$	Anion-CO_2	F---O	7	0.55	2.39	1.15	0.76	-1.15
$2CO_2$	$[Dbim]^+[FAP]^-$	Cation-CO_2	C-H---O	6	0.56	2.13	1.10	0.74	-0.98
$2CO_2$	$[Dbim]^+[FAP]^-$	Cation-CO_2	C---O	2	0.60	2.44	1.32	0.72	-1.09
$2CO_2$	$[Dbim]^+[Methide]^-$	Anion-Cation	C-H---F	3	0.60	2.37	1.07	0.77	-1.19
$2CO_2$	$[Dbim]^+[Methide]^-$	Anion-Cation	C-H---O	5	0.79	2.91	1.30	0.78	-1.46
$2CO_2$	$[Dbim]^+[Methide]^-$	Anion-Cation	C-H---C	1	0.82	3.33	1.61	0.76	-1.60
$2CO_2$	$[Dbim]^+[Methide]^-$	Anion-Cation	F---O	3	0.65	2.64	1.14	0.79	-1.36
$2CO_2$	$[Dbim]^+[Methide]^-$	Anion-Cation	C---O	1	0.63	2.23	1.03	0.77	-1.10
$2CO_2$	$[Dbim]^+[Methide]^-$	Anion-Cation	F---F	1	0.52	2.31	0.94	0.81	-1.22
$2CO_2$	$[Dbim]^+[Methide]^-$	Anion-Cation	S---F	1	0.64	2.23	0.93	0.80	-1.17
$2CO_2$	$[Dbim]^+[Methide]^-$	Anion-CO_2	F---O	4	0.54	2.34	1.10	0.77	-1.15
$2CO_2$	$[Dbim]^+[Methide]^-$	Anion-CO_2	C---F	1	0.62	2.82	1.18	0.80	-1.47
$2CO_2$	$[Dbim]^+[Methide]^-$	Anion-CO_2	S---O	1	0.76	3.36	1.78	0.73	-1.52

$2CO_2$	$[Dbim]^+[Methide]^-$	Cation-CO_2	C-H---O	7	0.55	2.14	1.10	0.74	-0.99
$2CO_2$	$[Dbim]^+[Methide]^-$	Cation-CO_2	C-H---C	1	0.53	2.05	1.01	0.75	-0.98
$2CO_2$	$[Dbim]^+[Methide]^-$	Cation-CO_2	N---F	1	0.74	3.52	1.81	0.74	-1.63
$2CO_2$	$[Dbim]^+[Methide]^-$	CO2-CO2	O---O	1	0.60	2.48	1.34	0.72	-1.10
$2CO_2$	$[Hmim]^+[FAP]^-$	Anion-Cation	C-H---F	3	0.76	3.08	1.36	0.79	-1.56
$2CO_2$	$[Hmim]^+[FAP]^-$	Anion-Cation	C---F	5	0.58	2.41	1.14	0.76	-1.18
$2CO_2$	$[Hmim]^+[FAP]^-$	Anion-Cation	F---F	8	1.29	6.84	3.22	0.77	-3.35
$2CO_2$	$[Hmim]^+[FAP]^-$	Anion-Cation	F---N	1	0.54	2.09	1.02	0.76	-1.00
$2CO_2$	$[Hmim]^+[FAP]^-$	Anion-CO_2	C---F	2	1.09	4.91	1.65	0.84	-2.82
$2CO_2$	$[Hmim]^+[FAP]^-$	Anion-CO_2	F---O	7	0.50	2.17	1.03	0.77	-1.06
$2CO_2$	$[Hmim]^+[FAP]^-$	Cation-CO_2	C-H---O	2	0.67	2.58	1.33	0.74	-1.19
$2CO_2$	$[Hmim]^+[FAP]^-$	CO_2-CO_2	C---O	1	0.82	3.53	1.58	0.78	-1.78
$2CO_2$	$[Hmim]^+[Methide]^-$	Anion-Cation	C-H---O	1	0.71	2.26	0.96	0.80	-1.17
$2CO_2$	$[Hmim]^+[Methide]^-$	Anion-Cation	C---O	2	0.69	2.87	1.19	0.78	-1.50
$2CO_2$	$[Hmim]^+[Methide]^-$	Anion-Cation	F---O	3	0.96	4.15	1.86	0.78	-2.08
$2CO_2$	$[Hmim]^+[Methide]^-$	Anion-Cation	C---F	3	0.70	3.01	1.41	0.77	-1.48
$2CO_2$	$[Hmim]^+[Methide]^-$	Anion-Cation	F---F	2	0.63	2.93	1.27	0.79	-1.50
$2CO_2$	$[Hmim]^+[Methide]^-$	Anion-Cation	N---F	1	0.80	3.81	1.89	0.75	-1.80
$2CO_2$	$[Hmim]^+[Methide]^-$	Anion-Cation	O---O	1	1.21	5.34	2.52	0.77	-2.60

$2CO_2$	$[Hmim]^+[Methide]^-$	Anion-CO_2	C---O	1	1.06	4.35	1.58	0.83	-2.42
$2CO_2$	$[Hmim]^+[Methide]^-$	Anion-CO_2	F---O	3	0.37	1.61	0.78	0.76	-0.78
$2CO_2$	$[Hmim]^+[Methide]^-$	Anion-CO_2	C---F	2	0.89	4.05	1.54	0.82	-2.21
$2CO_2$	$[Hmim]^+[Methide]^-$	Anion-CO_2	S---O	1	0.57	2.16	1.15	0.73	-0.97
$2CO_2$	$[Hmim]^+[Methide]^-$	Cation-CO_2	C-H---O	4	0.61	2.21	1.11	0.75	-1.04
$2CO_2$	$[Hmim]^+[(PFOc)SO_3]^-$	Anion-Cation	C-H---F	7	0.64	2.56	1.13	0.78	-1.30
$2CO_2$	$[Hmim]^+[(PFOc)SO_3]^-$	Anion-Cation	C-H---O	2	0.34	1.13	0.54	0.76	-0.54
$2CO_2$	$[Hmim]^+[(PFOc)SO_3]^-$	Anion-Cation	C---F	3	0.74	3.20	1.44	0.78	-1.60
$2CO_2$	$[Hmim]^+[(PFOc)SO_3]^-$	Anion-Cation	F---F	1	1.20	6.58	3.09	0.77	-3.22
$2CO_2$	$[Hmim]^+[(PFOc)SO_3]^-$	Anion-CO_2	F---O	7	0.48	2.17	1.04	0.75	-1.05
$2CO_2$	$[Hmim]^+[(PFOc)SO_3]^-$	Anion-CO_2	C---F	1	0.91	4.21	1.64	0.82	-2.28
$2CO_2$	$[Hmim]^+[(PFOc)SO_3]^-$	Cation-CO_2	C-H---O	4	0.62	2.55	1.29	0.74	-1.19
$2CO_2$	$[Hmim]^+[(PFOc)SO_3]^-$	CO_2-CO_2	C---O	1	0.81	3.54	1.59	0.78	-1.78
$2CO_2$	$[Omim]^+[(PFOc)SO_3]^-$	Anion-Cation	C-H---F	17	0.55	2.15	0.95	0.78	-1.09
$2CO_2$	$[Omim]^+[(PFOc)SO_3]^-$	Anion-Cation	C-H---O	2	0.86	3.32	1.40	0.77	-1.73
$2CO_2$	$[Omim]^+[(PFOc)SO_3]^-$	Anion-Cation	C---F	2	0.46	1.95	0.99	0.74	-0.91
$2CO_2$	$[Omim]^+[(PFOc)SO_3]^-$	Anion-Cation	F---F	5	1.27	6.96	3.39	0.76	-3.33
$2CO_2$	$[Omim]^+[(PFOc)SO_3]^-$	Anion-CO_2	F---O	7	0.59	2.69	1.29	0.76	-1.30
$2CO_2$	$[Omim]^+[(PFOc)SO_3]^-$	Anion-CO_2	C---F	1	0.79	3.59	1.39	0.82	-1.94
$2CO_2$	$[Omim]^+[(PFOc)SO_3]^-$	Cation-CO_2	C-H---O	3	0.40	1.36	0.66	0.76	-0.65
$2CO_2$	$[Omim]^+[(PFOc)SO_3]^-$	Cation-CO_2	N---O	1	0.73	3.49	2.00	0.70	-1.48

$2CO_2$	$[Omim]^+[(PFOc)SO_3]^-$	CO_2-CO_2	O---O	1	0.95	4.18	1.96	0.77	-2.04
$2CO_2$	$[Hmim]^+[(PFBu)SO_3]^-$	Anion-Cation	C-H---F	3	0.55	2.22	1.04	0.77	-1.09
$2CO_2$	$[Hmim]^+[(PFBu)SO_3]^-$	Anion-Cation	C---F	1	0.43	1.84	0.95	0.74	-0.85
$2CO_2$	$[Hmim]^+[(PFBu)SO_3]^-$	Anion-CO_2	F---O	4	0.56	2.40	1.11	0.77	-1.18
$2CO_2$	$[Hmim]^+[(PFBu)SO_3]^-$	Anion-CO_2	C---F	2	0.73	3.34	1.34	0.81	-1.78
$2CO_2$	$[Hmim]^+[(PFBu)SO_3]^-$	Cation-CO_2	C-H---O	3	0.44	1.61	0.86	0.73	-0.72
$2CO_2$	$[Hmim]^+[(PFBu)SO_3]^-$	CO_2-CO_2	C---O	1	0.85	3.81	1.70	0.78	-1.92
$2CO_2$	$[Omim]^+[(PFBu)SO_3]^-$	Anion-Cation	C-H---F	9	0.52	2.02	0.89	0.79	-1.03
$2CO_2$	$[Omim]^+[(PFBu)SO_3]^-$	Anion-Cation	C-H---O	2	0.62	2.09	0.93	0.79	-1.06
$2CO_2$	$[Omim]^+[(PFBu)SO_3]^-$	Anion-CO_2	F---O	5	0.62	2.78	1.31	0.76	-1.36
$2CO_2$	$[Omim]^+[(PFBu)SO_3]^-$	Anion-CO_2	C---O	1	1.11	4.67	1.73	0.83	-2.58
$2CO_2$	$[Omim]^+[(PFBu)SO_3]^-$	Anion-CO_2	O---O	1	0.46	1.71	0.77	0.78	-0.86
$2CO_2$	$[Omim]^+[(PFBu)SO_3]^-$	Cation-CO_2	C-H---O	6	0.66	2.58	1.30	0.75	-1.21
$2CO_2$	$[Omim]^+[(PFBu)SO_3]^-$	Cation-CO_2	C---O	1	0.68	2.97	1.57	0.73	-1.35
$2CO_2$	$[Omim]^+[(PFBu)SO_3]^-$	Cation-CO_2	N---O	1	0.71	3.24	1.83	0.71	-1.39
$2CO_2$	$[Omim]^+[(PFBu)SO_3]^-$	CO_2-CO_2	O---O	1	0.97	3.77	1.42	0.82	-2.07
$3CO_2$	$[Dmim]^+[BF_4]^-$	Anion-Cation	C---F	2	0.92	3.75	1.36	0.83	-2.09
$3CO_2$	$[Dmim]^+[BF_4]^-$	Anion-CO_2	C---F	4	1.13	4.91	1.56	0.85	-2.87
$3CO_2$	$[Dmim]^+[BF_4]^-$	Anion-CO_2	F---O	1	1.11	4.93	1.87	0.82	-2.69
$3CO_2$	$[Dmim]^+[BF_4]^-$	Cation-CO_2	C-H---O	2	0.48	1.77	0.94	0.73	-0.80
$3CO_2$	$[Dmim]^+[BF_4]^-$	Cation-CO_2	N---O	2	0.80	3.34	1.76	0.73	-1.51
$3CO_2$	$[Dmim]^+[BF_4]^-$	Cation-CO_2	C---O	2	0.39	1.55	0.90	0.70	-0.65

$3CO_2$	$[Dmim]^+[BF_4]^-$	CO_2-CO_2	O---O	2	0.55	2.39	1.20	0.74	-1.12
$3CO_2$	$[Dmim]^+[BF_4]^-$	CO_2-CO_2	C---O	1	0.97	4.29	1.86	0.79	-2.20
$3CO_2$	$[Dmim]^+[TFO]^-$	Anion-Cation	C-H---O	1	0.83	2.86	1.27	0.78	-1.45
$3CO_2$	$[Dmim]^+[TFO]^-$	Anion-Cation	C---O	1	0.54	1.86	0.89	0.76	-0.90
$3CO_2$	$[Dmim]^+[TFO]^-$	Anion-Cation	F---N	1	0.89	4.28	2.18	0.74	-1.99
$3CO_2$	$[Dmim]^+[TFO]^-$	Anion-CO_2	C---F	2	0.94	4.10	1.47	0.83	-2.29
$3CO_2$	$[Dmim]^+[TFO]^-$	Anion-CO_2	C---O	1	0.96	3.76	1.35	0.83	-2.10
$3CO_2$	$[Dmim]^+[TFO]^-$	Anion-CO_2	F---O	4	0.58	2.51	1.21	0.76	-1.21
$3CO_2$	$[Dmim]^+[TFO]^-$	Cation-CO_2	C-H---O	3	0.63	2.41	1.27	0.73	-1.10
$3CO_2$	$[Dmim]^+[TFO]^-$	Cation-CO_2	C---O	2	0.57	2.32	1.25	0.73	-1.04
$3CO_2$	$[Dmim]^+[TFO]^-$	Cation-CO_2	O---O	1	0.93	4.05	1.92	0.77	-1.97
$3CO_2$	$[Dmim]^+[TFO]^-$	Cation-CO_2	N---O	1	0.47	1.81	1.01	0.71	-0.79
$3CO_2$	$[Dmim]^+[TFO]^-$	CO_2-CO_2	O---O	1	0.83	3.97	2.02	0.74	-1.84
$3CO_2$	$[C_8H_4F_{13}mim]^+[BF_4]^-$	Anion-Cation	C-H---F	4	0.90	3.66	1.62	0.78	-1.85
$3CO_2$	$[C_8H_4F_{13}mim]^+[BF_4]^-$	Anion-Cation	C---F	2	0.90	3.77	1.54	0.81	-1.99
$3CO_2$	$[C_8H_4F_{13}mim]^+[BF_4]^-$	Anion-Cation	F---F	7	1.00	4.94	2.31	0.77	-2.42
$3CO_2$	$[C_8H_4F_{13}mim]^+[BF_4]^-$	Anion-CO_2	C---F	5	1.11	4.76	1.49	0.86	-2.80
$3CO_2$	$[C_8H_4F_{13}mim]^+[BF_4]^-$	Anion-CO_2	F---O	1	1.13	4.99	1.79	0.83	-2.79
$3CO_2$	$[C_8H_4F_{13}mim]^+[BF_4]^-$	Cation-CO_2	C-H---O	1	0.49	1.71	0.86	0.75	-0.81
$3CO_2$	$[C_8H_4F_{13}mim]^+[BF_4]^-$	Cation-CO_2	C-H---C	1	0.39	1.59	0.89	0.71	-0.69
$3CO_2$	$[C_8H_4F_{13}mim]^+[BF_4]^-$	Cation-CO_2	N---O	1	0.71	3.31	1.94	0.69	-1.38

$3CO_2$	$[C_8H_4F_{13}mim]^+[BF_4]^-$	Cation-CO_2	F---O	9	0.52	2.38	1.19	0.75	-1.12
$3CO_2$	$[C_8H_4F_{13}mim]^+[BF_4]^-$	CO_2-CO_2	O---O	2	0.29	1.17	0.61	0.74	-0.54
$3CO_2$	$[C_8H_4F_{13}mim]^+[TFO]^-$	Anion-Cation	C-H---F	3	0.64	2.54	1.14	0.78	-1.28
$3CO_2$	$[C_8H_4F_{13}mim]^+[TFO]^-$	Anion-Cation	C-H---O	3	0.94	3.34	1.46	0.79	-1.70
$3CO_2$	$[C_8H_4F_{13}mim]^+[TFO]^-$	Anion-Cation	C---F	2	0.71	2.98	1.33	0.78	-1.50
$3CO_2$	$[C_8H_4F_{13}mim]^+[TFO]^-$	Anion-Cation	F---F	11	0.86	4.23	1.97	0.77	-2.08
$3CO_2$	$[C_8H_4F_{13}mim]^+[TFO]^-$	Anion-CO_2	C---F	1	0.94	4.16	1.47	0.84	-2.34
$3CO_2$	$[C_8H_4F_{13}mim]^+[TFO]^-$	Anion-CO_2	C---O	1	1.21	4.98	1.70	0.84	-2.84
$3CO_2$	$[C_8H_4F_{13}mim]^+[TFO]^-$	Anion-CO_2	O---O	1	0.46	1.69	0.81	0.76	-0.82
$3CO_2$	$[C_8H_4F_{13}mim]^+[TFO]^-$	Anion-CO_2	F---O	4	0.48	2.07	1.00	0.76	-1.00
$3CO_2$	$[C_8H_4F_{13}mim]^+[TFO]^-$	Cation-CO_2	C-H---O	1	0.32	1.21	0.66	0.72	-0.53
$3CO_2$	$[C_8H_4F_{13}mim]^+[TFO]^-$	Cation-CO_2	C---F	2	0.93	4.25	1.57	0.83	-2.35
$3CO_2$	$[C_8H_4F_{13}mim]^+[TFO]^-$	Cation-CO_2	C---O	1	0.44	1.92	1.10	0.70	-0.82
$3CO_2$	$[C_8H_4F_{13}mim]^+[TFO]^-$	Cation-CO_2	N---O	1	0.78	3.26	1.75	0.73	-1.46
$3CO_2$	$[C_8H_4F_{13}mim]^+[TFO]^-$	Cation-CO_2	F---O	3	0.58	2.49	1.19	0.76	-1.21
$3CO_2$	$[C_8H_4F_{13}mim]^+[TFO]^-$	Cation-CO_2	O---O	1	1.03	4.54	1.98	0.79	-2.32
$3CO_2$	$[Dbim]^+[FAP]^-$	Anion-Cation	C-H---F	10	0.78	3.26	1.31	0.79	-1.74
$3CO_2$	$[Dbim]^+[FAP]^-$	Anion-Cation	C---F	1	0.51	2.21	1.10	0.75	-1.04
$3CO_2$	$[Dbim]^+[FAP]^-$	Anion-Cation	F---F	9	1.32	6.93	3.19	0.78	-3.43
$3CO_2$	$[Dbim]^+[FAP]^-$	Anion-CO_2	C---F	2	0.62	2.83	1.18	0.80	-1.48
$3CO_2$	$[Dbim]^+[FAP]^-$	Anion-CO_2	F---O	11	0.52	2.22	1.02	0.77	-1.10

$3CO_2$	$[Dbim]^+[FAP]^-$	Cation-CO_2	C-H---O	6	0.53	2.10	1.12	0.73	-0.94
$3CO_2$	$[Dbim]^+[FAP]^-$	Cation-CO_2	C-H---C	1	0.45	1.77	0.90	0.74	-0.82
$3CO_2$	$[Dbim]^+[FAP]^-$	Cation-CO_2	C---O	1	0.76	2.91	1.42	0.76	-1.39
$3CO_2$	$[Dbim]^+[FAP]^-$	CO_2-CO_2	C---O	1	0.88	3.92	1.76	0.78	-1.97
$3CO_2$	$[Dbim]^+[Methide]^-$	Anion-Cation	C-H---F	1	0.72	2.93	1.30	0.78	-1.48
$3CO_2$	$[Dbim]^+[Methide]^-$	Anion-Cation	C-H---O	4	0.59	2.15	0.99	0.77	-1.07
$3CO_2$	$[Dbim]^+[Methide]^-$	Anion-Cation	C-H---C	1	0.79	2.57	1.01	0.81	-1.38
$3CO_2$	$[Dbim]^+[Methide]^-$	Anion-Cation	F---O	3	0.67	2.74	1.19	0.79	-1.41
$3CO_2$	$[Dbim]^+[Methide]^-$	Anion-Cation	C---F	2	0.75	3.16	1.41	0.78	-1.59
$3CO_2$	$[Dbim]^+[Methide]^-$	Anion-Cation	N---F	1	0.79	3.89	1.95	0.75	-1.82
$3CO_2$	$[Dbim]^+[Methide]^-$	Anion-Cation	F---F	1	0.47	2.08	0.86	0.80	-1.09
$3CO_2$	$[Dbim]^+[Methide]^-$	Anion-CO_2	O---O	3	0.51	1.92	0.93	0.76	-0.93
$3CO_2$	$[Dbim]^+[Methide]^-$	Anion-CO_2	F---O	4	0.50	2.16	1.03	0.76	-1.05
$3CO_2$	$[Dbim]^+[Methide]^-$	Anion-CO_2	C---F	4	0.85	3.91	1.54	0.81	-2.10
$3CO_2$	$[Dbim]^+[Methide]^-$	Anion-CO_2	C---O	1	1.14	4.72	1.66	0.84	-2.66
$3CO_2$	$[Dbim]^+[Methide]^-$	Anion-CO_2	S---O	1	0.81	3.47	1.78	0.74	-1.61
$3CO_2$	$[Dbim]^+[Methide]^-$	Cation-CO_2	C-H---O	1	0.44	1.65	0.88	0.73	-0.74
$3CO_2$	$[Dbim]^+[Methide]^-$	Cation-CO_2	C---O	2	0.41	1.62	0.95	0.69	-0.68
$3CO_2$	$[Hmim]^+[FAP]^-$	Anion-Cation	C-H---F	2	0.61	2.56	1.17	0.78	-1.28
$3CO_2$	$[Hmim]^+[FAP]^-$	Anion-Cation	C---F	3	0.78	3.13	1.32	0.80	-1.62

$3CO_2$	$[Hmim]^+[FAP]^-$	Anion-Cation	F---F	8	1.32	7.02	3.29	0.77	-3.44
$3CO_2$	$[Hmim]^+[FAP]^-$	Anion-CO_2	C---F	4	0.96	4.30	1.54	0.83	-2.41
$3CO_2$	$[Hmim]^+[FAP]^-$	Anion-CO_2	F---O	8	0.50	2.17	1.03	0.76	-1.05
$3CO_2$	$[Hmim]^+[FAP]^-$	Cation-CO_2	C-H---O	5	0.55	2.11	1.13	0.72	-0.94
$3CO_2$	$[Hmim]^+[FAP]^-$	Cation-CO_2	C---O	1	0.59	2.49	1.40	0.71	-1.07
$3CO_2$	$[Hmim]^+[Methide]^-$	Anion-Cation	C-H---F	4	0.64	2.61	1.17	0.78	-1.31
$3CO_2$	$[Hmim]^+[Methide]^-$	Anion-Cation	C-H---O	1	0.71	2.68	1.32	0.75	-1.27
$3CO_2$	$[Hmim]^+[Methide]^-$	Anion-Cation	C---O	2	0.69	2.69	1.29	0.76	-1.30
$3CO_2$	$[Hmim]^+[Methide]^-$	Anion-Cation	F---O	3	0.81	3.42	1.50	0.79	-1.74
$3CO_2$	$[Hmim]^+[Methide]^-$	Anion-Cation	C---F	2	0.59	2.26	1.02	0.78	-1.13
$3CO_2$	$[Hmim]^+[Methide]^-$	Anion-Cation	F---F	3	0.53	2.40	1.01	0.80	-1.25
$3CO_2$	$[Hmim]^+[Methide]^-$	Anion-CO_2	C---O	2	1.03	4.28	1.59	0.83	-2.36
$3CO_2$	$[Hmim]^+[Methide]^-$	Anion-CO_2	F---O	7	0.56	2.51	1.24	0.75	-1.19
$3CO_2$	$[Hmim]^+[Methide]^-$	Anion-CO_2	C---F	1	0.87	3.85	1.41	0.83	-2.13
$3CO_2$	$[Hmim]^+[Methide]^-$	Cation-CO_2	C-H---O	5	0.47	1.83	0.98	0.73	-0.82
$3CO_2$	$[Hmim]^+[Methide]^-$	CO_2-CO_2	O---O	2	0.98	4.30	2.03	0.77	-2.10
$3CO_2$	$[Hmim]^+[(PFOc)SO_3]^-$	Anion-Cation	C-H---F	12	0.53	2.18	1.01	0.77	-1.08
$3CO_2$	$[Hmim]^+[(PFOc)SO_3]^-$	Anion-Cation	C-H---O	2	0.32	1.00	0.46	0.76	-0.50
$3CO_2$	$[Hmim]^+[(PFOc)SO_3]^-$	Anion-Cation	C---F	2	0.66	2.80	1.35	0.76	-1.35
$3CO_2$	$[Hmim]^+[(PFOc)SO_3]^-$	Anion-CO_2	F---O	10	0.41	1.80	0.87	0.75	-0.87

$3CO_2$	$[Hmim]^+[(PFOc)SO_3]^-$	Anion-CO_2	C---F	3	0.82	3.79	1.50	0.81	-2.04
$3CO_2$	$[Hmim]^+[(PFOc)SO_3]^-$	Cation-CO_2	C-H---O	5	0.49	1.81	0.94	0.73	-0.83
$3CO_2$	$[Hmim]^+[(PFOc)SO_3]^-$	Cation-CO_2	C---O	1	0.39	1.31	0.75	0.70	-0.56
$3CO_2$	$[Hmim]^+[(PFOc)SO_3]^-$	CO_2-CO_2	O---O	1	0.90	4.00	2.04	0.74	-1.85
$3CO_2$	$[Omim]^+[(PFOc)SO_3]^-$	Anion-Cation	C-H---O	2	0.55	1.95	0.86	0.79	-0.99
$3CO_2$	$[Omim]^+[(PFOc)SO_3]^-$	Anion-Cation	C---F	3	0.58	2.38	1.11	0.77	-1.17
$3CO_2$	$[Omim]^+[(PFOc)SO_3]^-$	Anion-CO_2	F---O	9	0.56	2.57	1.21	0.76	-1.26
$3CO_2$	$[Omim]^+[(PFOc)SO_3]^-$	Anion-CO_2	C---F	1	0.95	4.41	1.68	0.82	-2.41
$3CO_2$	$[Omim]^+[(PFOc)SO_3]^-$	Cation-CO_2	C-H---O	5	0.39	1.41	0.65	0.75	-0.70
$3CO_2$	$[Omim]^+[(PFOc)SO_3]^-$	Cation-CO_2	C---O	1	0.39	1.48	0.85	0.70	-0.63
$3CO_2$	$[Omim]^+[(PFOc)SO_3]^-$	CO_2-CO_2	C---O	1	0.95	4.41	2.00	0.78	-2.21
$3CO_2$	$[Omim]^+[(PFOc)SO_3]^-$	CO_2-CO_2	O---O	1	0.50	2.17	1.14	0.73	-0.98
$3CO_2$	$[Hmim]^+[(PFBu)SO_3]^-$	Anion-Cation	C-H---F	8	0.47	1.87	0.87	0.76	-0.92
$3CO_2$	$[Hmim]^+[(PFBu)SO_3]^-$	Anion-Cation	C---F	3	0.83	3.97	1.87	0.77	-1.94
$3CO_2$	$[Hmim]^+[(PFBu)SO_3]^-$	Anion-CO_2	F---O	6	0.63	2.76	1.28	0.77	-1.36
$3CO_2$	$[Hmim]^+[(PFBu)SO_3]^-$	Anion-CO_2	C---F	1	0.91	4.09	1.50	0.83	-2.26
$3CO_2$	$[Hmim]^+[(PFBu)SO_3]^-$	Anion-CO_2	C---O	2	0.73	2.90	1.15	0.77	-1.55
$3CO_2$	$[Hmim]^+[(PFBu)SO_3]^-$	Cation-CO_2	C-H---O	7	0.54	1.97	0.95	0.76	-0.94
$3CO_2$	$[Hmim]^+[(PFBu)SO_3]^-$	CO_2-CO_2	O---O	2	0.78	3.55	1.81	0.74	-1.65
$3CO_2$	$[Omim]^+[(PFBu)SO_3]^-$	Anion-Cation	C-H---F	8	0.63	2.55	1.14	0.78	-1.28
$3CO_2$	$[Omim]^+[(PFBu)SO_3]^-$	Anion-Cation	C-H---O	2	0.73	2.62	1.21	0.78	-1.29

$3CO_2$	$[Omim]^+[(PFBu)SO_3]^-$	Anion-Cation	C---O	1	0.46	1.69	0.85	0.75	-0.80
$3CO_2$	$[Omim]^+[(PFBu)SO_3]^-$	Anion-CO_2	C-H---O	2	0.57	2.02	0.99	0.76	-0.97
$3CO_2$	$[Omim]^+[(PFBu)SO_3]^-$	Anion-CO_2	F---O	7	0.55	2.43	1.19	0.76	-1.16
$3CO_2$	$[Omim]^+[(PFBu)SO_3]^-$	Anion-CO_2	C---O	1	0.86	3.53	1.38	0.81	-1.90
$3CO_2$	$[Omim]^+[(PFBu)SO_3]^-$	Anion-CO_2	C---F	1	0.82	3.77	1.48	0.81	-2.03
$3CO_2$	$[Omim]^+[(PFBu)SO_3]^-$	Cation-CO_2	C-H---O	2	0.60	2.20	0.98	0.77	-1.11
$3CO_2$	$[Omim]^+[(PFBu)SO_3]^-$	Cation-CO_2	C---O	2	0.76	3.32	1.66	0.75	-1.57
$3CO_2$	$[Omim]^+[(PFBu)SO_3]^-$	CO_2-CO_2	C---O	2	0.87	4.05	1.88	0.77	-2.00
$3CO_2$	$[Omim]^+[(PFBu)SO_3]^-$	CO_2-CO_2	O---O	1	0.72	3.29	1.62	0.75	-1.56
$4CO_2$	$[Dmim]^+[BF_4]^-$	Anion-Cation	C-H---F	3	0.66	2.55	1.10	0.79	-1.31
$4CO_2$	$[Dmim]^+[BF_4]^-$	Anion-Cation	C---F	3	0.76	3.01	1.21	0.80	-1.60
$4CO_2$	$[Dmim]^+[BF_4]^-$	Anion-CO_2	C---F	6	1.13	4.86	1.51	0.86	-2.87
$4CO_2$	$[Dmim]^+[BF_4]^-$	Anion-CO_2	F---O	1	0.59	2.46	1.07	0.79	-1.26
$4CO_2$	$[Dmim]^+[BF_4]^-$	Cation-CO_2	C-H---O	4	0.55	2.04	1.09	0.73	-0.92
$4CO_2$	$[Dmim]^+[BF_4]^-$	Cation-CO_2	N---O	1	0.71	3.12	1.81	0.70	-1.31
$4CO_2$	$[Dmim]^+[BF_4]^-$	CO_2-CO_2	O---O	1	0.27	1.09	0.56	0.74	-0.50
$4CO_2$	$[Dmim]^+[BF_4]^-$	CO_2-CO_2	C---O	4	0.84	3.81	1.75	0.77	-1.89
$4CO_2$	$[Dmim]^+[TFO]^-$	Anion-Cation	C-H---O	1	0.59	1.94	0.90	0.77	-0.96
$4CO_2$	$[Dmim]^+[TFO]^-$	Anion-Cation	C---O	2	0.65	2.32	1.03	0.78	-1.18
$4CO_2$	$[Dmim]^+[TFO]^-$	Anion-CO_2	C---F	1	0.93	4.20	1.57	0.82	-2.31
$4CO_2$	$[Dmim]^+[TFO]^-$	Anion-CO_2	C---O	3	1.07	4.37	1.57	0.83	-2.44
$4CO_2$	$[Dmim]^+[TFO]^-$	Anion-CO_2	O---O	1	0.52	1.91	0.86	0.78	-0.96

4CO_2	[Dmim]$^+$[TFO]$^-$	Anion-CO_2	F---O	1	0.56	2.42	1.14	0.77	-1.18
4CO_2	[Dmim]$^+$[TFO]$^-$	Cation-CO_2	C-H---O	2	0.36	1.38	0.74	0.72	-0.62
4CO_2	[Dmim]$^+$[TFO]$^-$	Cation-CO_2	C---O	3	0.71	2.88	1.48	0.74	-1.33
4CO_2	[Dmim]$^+$[TFO]$^-$	Cation-CO_2	C---C	1	0.80	2.94	1.11	0.82	-1.61
4CO_2	[Dmim]$^+$[TFO]$^-$	CO_2-CO_2	C---O	5	0.86	3.87	1.74	0.78	-1.95
4CO_2	[Dmim]$^+$[TFO]$^-$	CO_2-CO_2	O---O	1	0.93	4.05	2.00	0.75	-1.92
4CO_2	[$C_8H_4F_{13}$mim]$^+$[BF_4]$^-$	Anion-Cation	C-H---F	4	0.84	3.31	1.38	0.80	-1.73
4CO_2	[$C_8H_4F_{13}$mim]$^+$[BF_4]$^-$	Anion-Cation	C---F	2	0.89	3.62	1.43	0.81	-1.94
4CO_2	[$C_8H_4F_{13}$mim]$^+$[BF_4]$^-$	Anion-Cation	N---F	1	0.90	3.90	1.78	0.78	-1.94
4CO_2	[$C_8H_4F_{13}$mim]$^+$[BF_4]$^-$	Anion-Cation	F---F	9	0.85	4.14	1.91	0.78	-2.05
4CO_2	[$C_8H_4F_{13}$mim]$^+$[BF_4]$^-$	Anion-CO_2	C---F	4	1.17	5.10	1.62	0.85	-2.98
4CO_2	[$C_8H_4F_{13}$mim]$^+$[BF_4]$^-$	Anion-CO_2	F---O	2	0.72	3.04	1.21	0.81	-1.62
4CO_2	[$C_8H_4F_{13}$mim]$^+$[BF_4]$^-$	Cation-CO_2	C-H---O	3	0.70	2.80	1.45	0.73	-1.29
4CO_2	[$C_8H_4F_{13}$mim]$^+$[BF_4]$^-$	Cation-CO_2	F---O	9	0.61	2.75	1.36	0.75	-1.31
4CO_2	[$C_8H_4F_{13}$mim]$^+$[BF_4]$^-$	CO_2-CO_2	C---O	3	0.90	4.01	1.78	0.78	-2.03
4CO_2	[$C_8H_4F_{13}$mim]$^+$[BF_4]$^-$	CO_2-CO_2	O---O	1	0.28	1.17	0.62	0.73	-0.53
4CO_2	[$C_8H_4F_{13}$mim]$^+$[TFO]$^-$	Anion-Cation	C-H---F	4	0.70	2.89	1.29	0.78	-1.46
4CO_2	[$C_8H_4F_{13}$mim]$^+$[TFO]$^-$	Anion-Cation	C---F	1	0.56	2.25	1.07	0.77	-1.09
4CO_2	[$C_8H_4F_{13}$mim]$^+$[TFO]$^-$	Anion-Cation	F---F	5	1.01	4.89	2.29	0.77	-2.40
4CO_2	[$C_8H_4F_{13}$mim]$^+$[TFO]$^-$	Anion-CO_2	C---F	1	0.66	2.87	1.14	0.81	-1.53
4CO_2	[$C_8H_4F_{13}$mim]$^+$[TFO]$^-$	Anion-CO2	C---O	2	0.97	4.02	1.52	0.82	-2.20

$4CO_2$	$[C_8H_4F_{13}mim]^+[TFO]^-$	Anion-CO_2	O---O	3	0.88	3.47	1.36	0.80	-1.87
$4CO_2$	$[C_8H_4F_{13}mim]^+[TFO]^-$	Anion-CO_2	F---O	4	0.61	2.66	1.21	0.78	-1.33
$4CO_2$	$[C_8H_4F_{13}mim]^+[TFO]^-$	Cation-CO_2	C-H---O	6	0.63	2.45	1.30	0.73	-1.11
$4CO_2$	$[C_8H_4F_{13}mim]^+[TFO]^-$	Cation-CO_2	C---F	1	0.69	3.09	1.24	0.81	-1.64
$4CO_2$	$[C_8H_4F_{13}mim]^+[TFO]^-$	Cation-CO_2	C---O	1	0.79	2.85	1.18	0.80	-1.49
$4CO_2$	$[C_8H_4F_{13}mim]^+[TFO]^-$	Cation-CO_2	F---O	4	0.79	3.71	1.88	0.74	-1.73
$4CO_2$	$[C_8H_4F_{13}mim]^+[TFO]^-$	CO_2-CO_2	C---O	2	0.91	4.14	1.86	0.78	-2.08
$4CO_2$	$[C_8H_4F_{13}mim]^+[TFO]^-$	CO_2-CO_2	O---O	2	0.86	3.81	1.95	0.74	-1.76
$4CO_2$	$[Dbim]^+[FAP]^-$	Anion-Cation	C-H---F	13	0.66	2.66	1.17	0.79	-1.35
$4CO_2$	$[Dbim]^+[FAP]^-$	Anion-Cation	C---F	1	0.30	1.28	0.73	0.70	-0.55
$4CO_2$	$[Dbim]^+[FAP]^-$	Anion-Cation	F---F	8	1.33	7.10	3.34	0.77	-3.47
$4CO_2$	$[Dbim]^+[FAP]^-$	Anion-CO_2	C---F	4	1.01	4.56	1.61	0.83	-2.57
$4CO_2$	$[Dbim]^+[FAP]^-$	Anion-CO_2	F---O	11	0.54	2.37	1.11	0.77	-1.16
$4CO_2$	$[Dbim]^+[FAP]^-$	Cation-CO_2	C-H---O	6	0.49	1.87	1.00	0.72	-0.84
$4CO_2$	$[Dbim]^+[FAP]^-$	Cation-CO_2	C---O	1	0.43	1.77	1.01	0.70	-0.75
$4CO_2$	$[Dbim]^+[FAP]^-$	Cation-CO_2	N---O	1	0.81	3.59	2.10	0.69	-1.50
$4CO_2$	$[Dbim]^+[FAP]^-$	CO_2-CO_2	C---O	1	0.81	3.62	1.67	0.77	-1.79
$4CO_2$	$[Dbim]^+[FAP]^-$	CO_2-CO_2	O---O	1	0.87	3.79	1.88	0.75	-1.79
$4CO_2$	$[Dbim]^+[Methide]^-$	Anion-Cation	C-H---F	3	0.44	1.72	0.81	0.77	-0.85
$4CO_2$	$[Dbim]^+[Methide]^-$	Anion-Cation	C-H---O	6	0.67	2.46	1.10	0.78	-1.24
$4CO_2$	$[Dbim]^+[Methide]^-$	Anion-Cation	F---O	4	0.74	3.06	1.31	0.79	-1.58

$4CO_2$	$[Dbim]^+[Methide]^-$	Anion-Cation	C---F	1	0.71	2.68	1.30	0.76	-1.28
$4CO_2$	$[Dbim]^+[Methide]^-$	Anion-Cation	C---O	2	0.46	1.77	0.83	0.76	-0.87
$4CO_2$	$[Dbim]^+[Methide]^-$	Anion-Cation	F---F	2	0.68	3.25	1.48	0.78	-1.62
$4CO_2$	$[Dbim]^+[Methide]^-$	Anion-Cation	N---O	1	0.73	2.86	1.48	0.74	-1.32
$4CO_2$	$[Dbim]^+[Methide]^-$	Anion-CO_2	O---O	1	1.22	5.22	2.30	0.79	-2.65
$4CO_2$	$[Dbim]^+[Methide]^-$	Anion-CO_2	F---O	11	0.49	2.16	1.04	0.76	-1.04
$4CO_2$	$[Dbim]^+[Methide]^-$	Anion-CO_2	C---F	1	0.92	4.20	1.61	0.82	-2.28
$4CO_2$	$[Dbim]^+[Methide]^-$	Anion-CO_2	C---O	2	1.22	5.24	1.88	0.83	-2.93
$4CO_2$	$[Dbim]^+[Methide]^-$	Cation-CO_2	C-H---O	5	0.40	1.48	0.77	0.73	-0.67
$4CO_2$	$[Dbim]^+[Methide]^-$	CO_2-CO_2	O---O	2	0.93	4.04	1.84	0.78	-2.02
$4CO_2$	$[Dbim]^+[Methide]^-$	CO_2-CO_2	C---O	1	0.89	3.92	1.71	0.79	-2.00
$4CO_2$	$[Hmim]^+[FAP]^-$	Anion-Cation	C-H---F	8	0.59	2.32	1.03	0.78	-1.17
$4CO_2$	$[Hmim]^+[FAP]^-$	Anion-Cation	C---F	3	0.59	2.56	1.16	0.77	-1.28
$4CO_2$	$[Hmim]^+[FAP]^-$	Anion-Cation	F---F	9	1.31	6.98	3.27	0.77	-3.42
$4CO_2$	$[Hmim]^+[FAP]^-$	Anion-CO_2	C---F	3	0.96	4.27	1.52	0.83	-2.39
$4CO_2$	$[Hmim]^+[FAP]^-$	Anion-CO_2	F---O	10	0.65	2.93	1.36	0.77	-1.44
$4CO_2$	$[Hmim]^+[FAP]^-$	Cation-CO_2	C-H---O	6	0.60	2.30	1.17	0.73	-1.07
$4CO_2$	$[Hmim]^+[FAP]^-$	CO_2-CO_2	C---O	2	0.93	4.18	1.85	0.79	-2.12
$4CO_2$	$[Hmim]^+[FAP]^-$	CO_2-CO_2	O---O	1	1.03	4.51	2.03	0.78	-2.26
$4CO_2$	$[Hmim]^+[Methide]^-$	Anion-Cation	C-H---O	9	0.59	2.02	0.87	0.79	-1.04

$4CO_2$	$[Hmim]^+[Methide]^-$	Anion-Cation	C-H---C	1	0.74	2.25	0.90	0.81	-1.21
$4CO_2$	$[Hmim]^+[Methide]^-$	Anion-Cation	F---O	3	0.86	3.79	1.73	0.78	-1.89
$4CO_2$	$[Hmim]^+[Methide]^-$	Anion-Cation	S---F	1	0.91	3.75	1.60	0.79	-1.93
$4CO_2$	$[Hmim]^+[Methide]^-$	Anion-Cation	O---O	1	1.18	5.04	2.33	0.77	-2.49
$4CO_2$	$[Hmim]^+[Methide]^-$	Anion-Cation	C---F	1	0.58	2.04	0.96	0.77	-1.00
$4CO_2$	$[Hmim]^+[Methide]^-$	Anion-Cation	F---F	1	0.94	4.74	2.33	0.76	-2.26
$4CO_2$	$[Hmim]^+[Methide]^-$	Anion-Cation	N---F	1	0.96	4.59	2.25	0.76	-2.19
$4CO_2$	$[Hmim]^+[Methide]^-$	Anion-CO_2	C---O	2	1.03	4.28	1.59	0.82	-2.36
$4CO_2$	$[Hmim]^+[Methide]^-$	Anion-CO_2	F---O	6	0.70	3.15	1.46	0.77	-1.55
$4CO_2$	$[Hmim]^+[Methide]^-$	Anion-CO_2	O---O	4	0.67	2.58	1.14	0.78	-1.31
$4CO_2$	$[Hmim]^+[Methide]^-$	Anion-CO_2	C---F	1	0.81	3.69	1.44	0.81	-1.99
$4CO_2$	$[Hmim]^+[Methide]^-$	Anion-CO_2	S---O	2	0.69	2.55	1.25	0.74	-1.22
$4CO_2$	$[Hmim]^+[Methide]^-$	Cation-CO_2	C-H---O	5	0.64	2.51	1.36	0.72	-1.12
$4CO_2$	$[Hmim]^+[Methide]^-$	Cation-CO_2	C---O	1	0.44	1.74	0.97	0.71	-0.76
$4CO_2$	$[Hmim]^+[Methide]^-$	CO_2-CO_2	C---O	2	0.82	3.79	1.75	0.77	-1.87
$4CO_2$	$[Hmim]^+[(PFOc)SO_3]^-$	Anion-Cation	C-H---F	7	0.29	1.13	0.55	0.74	-0.54
$4CO_2$	$[Hmim]^+[(PFOc)SO_3]^-$	Anion-Cation	F---F	3	1.36	7.65	3.73	0.76	-3.66
$4CO_2$	$[Hmim]^+[(PFOc)SO_3]^-$	Anion-CO_2	F---O	9	0.61	2.71	1.30	0.76	-1.31
$4CO_2$	$[Hmim]^+[(PFOc)SO_3]^-$	Anion-CO_2	C---F	5	0.77	3.62	1.48	0.80	-1.91
$4CO_2$	$[Hmim]^+[(PFOc)SO_3]^-$	Cation-CO_2	C-H---O	8	0.55	2.09	1.04	0.74	-0.99

$4CO_2$	$[Hmim]^+[(PFOc)SO_3]^-$	Cation-CO_2	C-H---C	1	0.84	3.16	1.20	0.82	-1.73
$4CO_2$	$[Hmim]^+[(PFOc)SO_3]^-$	Cation-CO_2	C---O	3	0.69	2.88	1.44	0.74	-1.35
$4CO_2$	$[Hmim]^+[(PFOc)SO_3]^-$	Cation-CO_2	C---C	1	0.88	3.06	1.20	0.81	-1.64
$4CO_2$	$[Hmim]^+[(PFOc)SO_3]^-$	CO_2-CO_2	O---O	2	0.79	3.47	1.80	0.73	-1.60
$4CO_2$	$[Omim]^+[(PFOc)SO_3]^-$	Anion-Cation	C-H---F	4	0.41	1.54	0.69	0.78	-0.77
$4CO_2$	$[Omim]^+[(PFOc)SO_3]^-$	Anion-Cation	C---O	1	0.76	3.53	1.70	0.76	-1.71
$4CO_2$	$[Omim]^+[(PFOc)SO_3]^-$	Anion-CO_2	F---O	6	0.56	2.46	1.15	0.77	-1.21
$4CO_2$	$[Omim]^+[(PFOc)SO_3]^-$	Anion-CO_2	C---F	1	0.89	4.16	1.65	0.81	-2.23
$4CO_2$	$[Omim]^+[(PFOc)SO_3]^-$	Cation-CO_2	C-H---O	8	0.53	2.00	1.01	0.74	-0.94
$4CO_2$	$[Omim]^+[(PFOc)SO_3]^-$	Cation-CO_2	C---O	2	0.58	2.33	1.16	0.74	-1.10
$4CO_2$	$[Omim]^+[(PFOc)SO_3]^-$	Cation-CO_2	C---C	1	0.81	2.81	1.16	0.80	-1.48
$4CO_2$	$[Omim]^+[(PFOc)SO_3]^-$	CO_2-CO_2	C---O	1	0.85	3.94	1.81	0.78	-1.96
$4CO_2$	$[Omim]^+[(PFOc)SO_3]^-$	CO_2-CO_2	O---O	2	0.65	2.79	1.28	0.76	-1.39
$4CO_2$	$[Hmim]^+[(PFBu)SO_3]^-$	Anion-Cation	C-H---F	7	0.53	2.15	0.98	0.77	-1.07
$4CO_2$	$[Hmim]^+[(PFBu)SO_3]^-$	Anion-Cation	C-H---O	3	0.45	1.40	0.57	0.81	-0.74
$4CO_2$	$[Hmim]^+[(PFBu)SO_3]^-$	Anion-Cation	C---F	3	0.68	2.82	1.26	0.78	-1.42
$4CO_2$	$[Hmim]^+[(PFBu)SO_3]^-$	Anion-Cation	F---N	2	0.57	2.45	1.17	0.76	-1.19
$4CO_2$	$[Hmim]^+[(PFBu)SO_3]^-$	Anion-CO_2	C-H---O	1	0.52	1.86	0.91	0.76	-0.89
$4CO_2$	$[Hmim]^+[(PFBu)SO_3]^-$	Anion-CO_2	F---O	8	0.58	2.57	1.21	0.77	-1.25
$4CO_2$	$[Hmim]^+[(PFBu)SO_3]^-$	Anion-CO_2	C---F	2	0.79	3.60	1.43	0.81	-1.93
$4CO_2$	$[Hmim]^+[(PFBu)SO_3]^-$	Anion-CO_2	C---O	2	0.99	4.26	1.66	0.82	-2.30

$4CO_2$	$[Hmim]^+[(PFBu)SO_3]^-$	Anion-CO_2	O---O	1	0.30	1.09	0.52	0.76	-0.52
$4CO_2$	$[Hmim]^+[(PFBu)SO_3]^-$	Cation-CO_2	C-H---O	6	0.47	1.73	0.86	0.74	-0.81
$4CO_2$	$[Hmim]^+[(PFBu)SO_3]^-$	Cation-CO_2	C---O	3	0.49	1.94	1.08	0.71	-0.85
$4CO_2$	$[Hmim]^+[(PFBu)SO_3]^-$	CO_2-CO_2	C---O	2	0.86	3.92	1.80	0.77	-1.95
$4CO_2$	$[Omim]^+[(PFBu)SO_3]^-$	Anion-Cation	C-H---F	3	0.65	2.54	1.14	0.78	-1.28
$4CO_2$	$[Omim]^+[(PFBu)SO_3]^-$	Anion-Cation	C-H---O	6	0.60	2.13	0.95	0.78	-1.08
$4CO_2$	$[Omim]^+[(PFBu)SO_3]^-$	Anion-CO_2	F---O	8	0.59	2.66	1.26	0.76	-1.29
$4CO_2$	$[Omim]^+[(PFBu)SO_3]^-$	Anion-CO_2	C---O	2	1.15	4.68	1.60	0.84	-2.66
$4CO_2$	$[Omim]^+[(PFBu)SO_3]^-$	Anion-CO_2	C---F	1	1.04	4.98	1.93	0.82	-2.69
$4CO_2$	$[Omim]^+[(PFBu)SO_3]^-$	Cation-CO_2	C-H---O	4	0.56	2.14	1.08	0.74	-1.00
$4CO_2$	$[Omim]^+[(PFBu)SO_3]^-$	Cation-CO_2	C---O	2	0.71	2.95	1.49	0.74	-1.38
$4CO_2$	$[Omim]^+[(PFBu)SO_3]^-$	CO_2-CO_2	C---O	2	0.82	3.67	1.69	0.77	-1.82
$5CO_2$	$[Dmim]^+[BF_4]^-$	Anion-Cation	C-H---F	4	0.87	3.38	1.36	0.81	-1.80
$5CO_2$	$[Dmim]^+[BF_4]^-$	Anion-Cation	C---F	1	0.65	2.45	0.98	0.81	-1.30
$5CO_2$	$[Dmim]^+[BF_4]^-$	Anion-CO_2	C---F	7	1.12	4.89	1.54	0.86	-2.87
$5CO_2$	$[Dmim]^+[BF_4]^-$	Anion-CO_2	F---O	1	0.65	2.70	1.18	0.79	-1.38
$5CO_2$	$[Dmim]^+[BF_4]^-$	Cation-CO_2	C-H---O	6	0.58	2.19	1.17	0.73	-0.99
$5CO_2$	$[Dmim]^+[BF_4]^-$	Cation-CO2	N---O	1	0.72	3.20	1.86	0.70	-1.34
$5CO_2$	$[Dmim]^+[BF_4]^-$	CO_2-CO_2	O---O	3	0.65	2.86	1.42	0.75	-1.35
$5CO_2$	$[Dmim]^+[BF_4]^-$	CO_2-CO_2	C---O	3	0.87	3.88	1.74	0.78	-1.95
$5CO_2$	$[Dmim]^+[TFO]^-$	Anion-Cation	C-H---O	1	0.67	2.22	1.00	0.78	-1.11

$5CO_2$	$[Dmim]^+[TFO]^-$	Anion-Cation	C---O	1	1.04	4.60	1.87	0.81	-2.44
$5CO_2$	$[Dmim]^+[TFO]^-$	Anion-Cation	F---N	1	0.80	3.61	1.85	0.74	-1.67
$5CO_2$	$[Dmim]^+[TFO]^-$	Anion-CO_2	C---O	3	1.06	4.15	1.42	0.84	-0.81
$5CO_2$	$[Dmim]^+[TFO]^-$	Cation-CO2	C-H---O	2	0.47	1.83	1.00	0.72	-0.81
$5CO_2$	$[Dmim]^+[TFO]^-$	Cation-CO2	C---O	2	0.62	2.55	1.31	0.74	-1.18
$5CO_2$	$[Dmim]^+[TFO]^-$	Cation-CO2	N---O	1	0.73	3.02	1.67	0.71	-1.32
$5CO_2$	$[Dmim]^+[TFO]^-$	CO2-CO2	C---O	1	0.78	3.42	1.54	0.78	-1.71
$5CO_2$	$[Dmim]^+[TFO]^-$	CO2-CO2	O---O	6	0.71	3.17	1.66	0.73	-1.44
$5CO_2$	$[C_8H_4F_{13}mim]^+[BF_4]^-$	Anion-Cation	C-H---F	4	0.77	3.05	1.26	0.80	-1.60
$5CO_2$	$[C_8H_4F_{13}mim]^+[BF_4]^-$	Anion-Cation	C---F	5	0.84	3.44	1.31	0.82	-1.87
$5CO_2$	$[C_8H_4F_{13}mim]^+[BF_4]^-$	Anion-Cation	F---F	7	0.85	4.16	1.92	0.78	-2.06
$5CO_2$	$[C_8H_4F_{13}mim]^+[BF_4]^-$	Anion-CO_2	C---F	2	1.17	5.28	1.77	0.85	-3.03
$5CO_2$	$[C_8H_4F_{13}mim]^+[BF_4]^-$	Anion-CO_2	F---O	3	1.01	4.42	1.61	0.83	-2.45
$5CO_2$	$[C_8H_4F_{13}mim]^+[BF_4]^-$	Cation-CO_2	C---F	3	0.77	3.66	1.51	0.80	-1.92
$5CO_2$	$[C_8H_4F_{13}mim]^+[BF_4]^-$	Cation-CO_2	F---O	13	0.52	2.36	1.18	0.75	-1.11
$5CO_2$	$[C_8H_4F_{13}mim]^+[BF_4]^-$	CO_2-CO_2	C---O	2	0.80	3.52	1.62	0.77	-1.74
$5CO_2$	$[C_8H_4F_{13}mim]^+[BF_4]^-$	CO_2-CO_2	O---O	3	0.74	3.19	1.51	0.76	-1.56
$5CO_2$	$[C_8H_4F_{13}mim]^+[TFO]^-$	Anion-Cation	C-H---F	2	0.65	2.56	1.16	0.78	-1.28
$5CO_2$	$[C_8H_4F_{13}mim]^+[TFO]^-$	Anion-Cation	C-H---O	1	0.83	2.83	1.28	0.78	-1.42

$5CO_2$	$[C_8H_4F_{13}mim]^+[TFO]^-$	Anion-Cation	C---F	4	0.75	3.38	1.50	0.78	-1.71
$5CO_2$	$[C_8H_4F_{13}mim]^+[TFO]^-$	Anion-Cation	F---F	7	0.91	4.49	2.11	0.76	-2.20
$5CO_2$	$[C_8H_4F_{13}mim]^+[TFO]^-$	Anion-Cation	N---F	1	0.70	2.81	1.33	0.77	-1.37
$5CO_2$	$[C_8H_4F_{13}mim]^+[TFO]^-$	Anion-CO_2	C---O	2	1.13	4.59	1.61	0.84	-2.59
$5CO_2$	$[C_8H_4F_{13}mim]^+[TFO]^-$	Anion-CO_2	F---O	10	0.64	2.84	1.34	0.77	-1.39
$5CO_2$	$[C_8H_4F_{13}mim]^+[TFO]^-$	Cation-CO_2	C-H---O	2	0.70	2.66	1.30	0.76	-1.27
$5CO_2$	$[C_8H_4F_{13}mim]^+[TFO]^-$	Cation-CO_2	C---F	1	0.83	3.91	1.57	0.81	-2.08
$5CO_2$	$[C_8H_4F_{13}mim]^+[TFO]^-$	Cation-CO_2	C---O	1	0.59	2.50	1.40	0.71	-1.08
$5CO_2$	$[C_8H_4F_{13}mim]^+[TFO]^-$	Cation-CO_2	F---O	7	0.66	2.90	1.36	0.77	-1.42
$5CO_2$	$[C_8H_4F_{13}mim]^+[TFO]^-$	CO_2-CO_2	C---O	3	0.89	3.97	1.78	0.78	-1.99
$5CO_2$	$[C_8H_4F_{13}mim]^+[TFO]^-$	CO_2-CO_2	O---O	4	0.65	2.78	1.36	0.75	-1.33
$5CO_2$	$[Dbim]^+[FAP]^-$	Anion-Cation	C-H---F	4	0.56	2.17	0.96	0.78	-1.10
$5CO_2$	$[Dbim]^+[FAP]^-$	Anion-Cation	C-H---C	1	0.81	2.82	1.27	0.78	-1.42
$5CO_2$	$[Dbim]^+[FAP]^-$	Anion-Cation	C---F	3	0.66	2.70	1.20	0.78	-1.36
$5CO_2$	$[Dbim]^+[FAP]^-$	Anion-Cation	F---F	8	1.31	6.99	3.31	0.77	-3.41
$5CO_2$	$[Dbim]^+[FAP]^-$	Anion-CO_2	C---F	3	1.01	4.53	1.58	0.84	-2.57
$5CO_2$	$[Dbim]^+[FAP]^-$	Anion-CO_2	F---O	15	0.63	2.79	1.28	0.77	-1.39
$5CO_2$	$[Dbim]^+[FAP]^-$	Cation-CO_2	C-H---O	10	0.52	1.97	0.96	0.74	-0.94
$5CO_2$	$[Dbim]^+[FAP]^-$	Cation-CO_2	C---O	3	0.71	2.87	1.42	0.75	-1.36
$5CO_2$	$[Dbim]^+[FAP]^-$	CO_2-CO_2	C---O	2	0.91	4.07	1.78	0.79	-2.07
$5CO_2$	$[Dbim]^+[FAP]^-$	CO_2-CO_2	O---O	1	0.96	4.26	2.04	0.76	-2.06

5CO2	$[Dbim]^+[Methide]^-$	Anion-Cation	C-H---F	1	0.51	2.20	1.11	0.75	-1.03
5CO2	$[Dbim]^+[Methide]^-$	Anion-Cation	C-H---O	3	0.82	2.96	1.35	0.78	-1.47
$5CO_2$	$[Dbim]^+[Methide]^-$	Anion-Cation	C-H---C	1	1.00	3.75	1.49	0.81	-2.01
$5CO_2$	$[Dbim]^+[Methide]^-$	Anion-Cation	F---O	3	0.68	2.79	1.20	0.79	-1.44
$5CO_2$	$[Dbim]^+[Methide]^-$	Anion-Cation	N---O	1	0.70	2.74	1.42	0.74	-1.26
$5CO_2$	$[Dbim]^+[Methide]^-$	Anion-Cation	F---F	1	0.61	2.70	1.12	0.80	-1.42
$5CO_2$	$[Dbim]^+[Methide]^-$	Anion-CO_2	O---O	3	0.54	2.02	0.96	0.77	-0.98
$5CO_2$	$[Dbim]^+[Methide]^-$	Anion-CO_2	F---O	6	0.57	2.52	1.23	0.76	-1.21
$5CO_2$	$[Dbim]^+[Methide]^-$	Anion-CO_2	C---F	2	0.65	2.96	1.23	0.80	-1.55
$5CO_2$	$[Dbim]^+[Methide]^-$	Anion-CO_2	C---O	4	1.00	4.13	1.53	0.83	-2.28
$5CO_2$	$[Dbim]^+[Methide]^-$	Cation-CO_2	C-H---O	4	0.50	1.88	0.98	0.74	-0.86
$5CO_2$	$[Dbim]^+[Methide]^-$	Cation-CO_2	N---O	1	0.59	2.42	1.34	0.71	-1.05
$5CO_2$	$[Dbim]^+[Methide]^-$	Cation-CO_2	C---O	1	1.39	5.12	1.77	0.84	-2.91
$5CO_2$	$[Dbim]^+[Methide]^-$	CO_2-CO_2	O---O	2	0.93	4.12	2.10	0.74	-1.92
$5CO_2$	$[Hmim]^+[FAP]^-$	Anion-Cation	C-H---F	8	0.59	2.32	1.03	0.77	-1.17
$5CO_2$	$[Hmim]^+[FAP]^-$	Anion-Cation	C---F	2	0.62	2.73	1.25	0.77	-1.35
$5CO_2$	$[Hmim]^+[FAP]^-$	Anion-Cation	C---O	1	0.92	4.01	1.75	0.79	-2.05
$5CO_2$	$[Hmim]^+[FAP]^-$	Anion-Cation	F---F	9	1.29	6.84	3.16	0.78	-3.38
$5CO_2$	$[Hmim]^+[FAP]^-$	Anion-Cation	F---N	2	0.59	2.47	1.20	0.76	-1.19
$5CO_2$	$[Hmim]^+[FAP]^-$	Anion-CO_2	C---F	6	0.91	4.19	1.56	0.82	-2.31

5CO_2	$[Hmim]^+[FAP]^-$	Anion-CO_2	F---O	8	0.62	2.80	1.32	0.76	-1.37
5CO_2	$[Hmim]^+[FAP]^-$	Cation-CO_2	C-H---O	2	0.41	1.50	0.78	0.72	-0.68
5CO_2	$[Hmim]^+[FAP]^-$	Cation-CO2	C-H---C	1	0.83	2.98	1.03	0.84	-1.69
5CO_2	$[Hmim]^+[FAP]^-$	Cation-CO_2	C---O	2	0.64	2.32	1.24	0.73	-1.05
5CO_2	$[Hmim]^+[FAP]^-$	CO_2-CO_2	C-H---O	2	0.40	1.49	0.77	0.74	-0.69
5CO_2	$[Hmim]^+[FAP]^-$	CO_2-CO_2	C---O	1	0.79	3.68	1.76	0.76	-1.79
5CO_2	$[Hmim]^+[FAP]^-$	CO_2-CO_2	O---O	4	0.46	1.98	1.02	0.74	-0.91
5CO_2	$[Hmim]^+[Methide]^-$	Anion-Cation	C-H---F	8	0.48	1.92	0.89	0.76	-0.95
5CO_2	$[Hmim]^+[Methide]^-$	Anion-Cation	C---O	1	0.77	2.75	1.20	0.79	-1.40
5CO_2	$[Hmim]^+[Methide]^-$	Anion-Cation	F---O	4	0.65	2.70	1.19	0.78	-1.37
5CO_2	$[Hmim]^+[Methide]^-$	Anion-Cation	F---F	2	0.90	4.41	2.08	0.77	-2.15
5CO_2	$[Hmim]^+[Methide]^-$	Anion-CO_2	C---O	2	1.06	4.48	1.71	0.82	-2.44
5CO_2	$[Hmim]^+[Methide]^-$	Anion-CO_2	F---O	11	0.63	2.79	1.35	0.76	-1.34
5CO_2	$[Hmim]^+[Methide]^-$	Anion-CO_2	C---F	2	0.97	4.39	1.63	0.83	-2.42
5CO_2	$[Hmim]^+[Methide]^-$	Anion-CO_2	O---O	1	0.41	1.63	0.86	0.73	-0.74
5CO_2	$[Hmim]^+[Methide]^-$	Cation-CO_2	C-H---O	7	0.56	2.00	1.00	0.75	-0.94
5CO_2	$[Hmim]^+[Methide]^-$	Cation-CO_2	C---O	1	0.75	2.82	1.46	0.74	-1.30
5CO_2	$[Hmim]^+[Methide]^-$	CO_2-CO_2	C---O	1	0.80	3.46	1.60	0.77	-1.71
5CO_2	$[Hmim]^+[Methide]^-$	CO_2-CO_2	O---O	4	0.67	2.97	1.48	0.74	-1.40
5CO_2	$[Hmim]^+[(PFOc)SO_3]^-$	Anion-Cation	C-H---F	5	0.48	1.92	0.87	0.78	-0.95
5CO_2	$[Hmim]^+[(PFOc)SO_3]^-$	Anion-Cation	F---F	2	1.18	6.36	2.98	0.77	-3.12
5CO_2	$[Hmim]^+[(PFOc)SO_3]^-$	Anion-CO_2	F---O	13	0.59	2.63	1.25	0.76	-1.28

$5CO_2$	$[Hmim]^+[(PFOc)SO_3]^-$	Anion-CO_2	C---F	4	0.72	3.33	1.38	0.80	-1.75
$5CO_2$	$[Hmim]^+[(PFOc)SO_3]^-$	Cation-CO_2	C-H---O	8	0.58	2.16	1.09	0.74	-1.01
$5CO_2$	$[Hmim]^+[(PFOc)SO_3]^-$	Cation-CO_2	C-H---C	1	0.78	2.88	1.14	0.81	-1.54
$5CO_2$	$[Hmim]^+[(PFOc)SO_3]^-$	Cation-CO_2	N---O	1	0.60	2.44	1.38	0.71	-1.05
$5CO_2$	$[Hmim]^+[(PFOc)SO_3]^-$	CO_2-CO_2	C---O	3	0.84	3.79	1.71	0.78	-1.90
$5CO_2$	$[Hmim]^+[(PFOc)SO_3]^-$	CO_2-CO_2	O---O	4	0.52	2.16	1.09	0.74	-1.01
$5CO_2$	$[Omim]^+[(PFOc)SO_3]^-$	Anion-Cation	C-H---F	5	0.52	2.03	0.93	0.77	-1.01
$5CO_2$	$[Omim]^+[(PFOc)SO_3]^-$	Anion-Cation	F---F	3	1.41	7.89	3.91	0.75	-3.74
$5CO_2$	$[Omim]^+[(PFOc)SO_3]^-$	Anion-CO_2	F---O	12	0.51	2.27	1.08	0.76	-1.10
$5CO_2$	$[Omim]^+[(PFOc)SO_3]^-$	Anion-CO_2	C---F	4	0.75	3.49	1.43	0.80	-1.84
$5CO_2$	$[Omim]^+[(PFOc)SO_3]^-$	Cation-CO_2	C-H---O	11	0.62	2.37	1.18	0.75	-1.11
$5CO_2$	$[Omim]^+[(PFOc)SO_3]^-$	Cation-CO_2	C---O	1	0.39	1.41	0.80	0.71	-0.61
$5CO_2$	$[Omim]^+[(PFOc)SO_3]^-$	CO_2-CO_2	C---O	3	0.91	4.15	1.84	0.78	-2.10
$5CO_2$	$[Omim]^+[(PFOc)SO_3]^-$	CO_2-CO_2	O---O	3	0.52	2.23	1.14	0.73	-1.04
$5CO_2$	$[Hmim]^+[(PFBu)SO_3]^-$	Anion-Cation	C-H---F	5	0.66	2.67	1.19	0.78	-1.35
$5CO_2$	$[Hmim]^+[(PFBu)SO_3]^-$	Anion-Cation	C-H---O	3	0.71	2.49	1.11	0.79	-1.26
$5CO_2$	$[Hmim]^+[(PFBu)SO_3]^-$	Anion-Cation	F---O	1	1.00	4.44	1.90	0.79	-2.28
$5CO_2$	$[Hmim]^+[(PFBu)SO_3]^-$	Anion-Cation	C---F	3	0.68	2.91	1.32	0.78	-1.46
$5CO_2$	$[Hmim]^+[(PFBu)SO_3]^-$	Anion-Cation	F---F	1	1.58	9.14	4.60	0.75	-4.28
$5CO_2$	$[Hmim]^+[(PFBu)SO_3]^-$	Anion-CO_2	F---O	11	0.61	2.68	1.27	0.77	-1.31
$5CO_2$	$[Hmim]^+[(PFBu)SO_3]^-$	Anion-CO_2	C---F	2	0.71	3.24	1.34	0.80	-1.71

5CO_2	$[Hmim]^+[(PFBu)SO_3]^-$	Cation-CO_2	C-H---O	8	0.43	1.62	0.87	0.72	-0.73
5CO2	$[Hmim]^+[(PFBu)SO_3]^-$	Cation-CO_2	C-H---C	1	0.67	2.48	1.02	0.80	-1.30
5CO_2	$[Hmim]^+[(PFBu)SO_3]^-$	Cation-CO_2	C---O	1	0.58	2.46	1.36	0.71	-1.07
5CO_2	$[Hmim]^+[(PFBu)SO_3]^-$	CO_2-CO_2	C---O	5	0.89	3.99	1.79	0.78	-2.00
5CO_2	$[Omim]^+[(PFBu)SO_3]^-$	Anion-Cation	C-H---F	4	0.65	2.52	1.08	0.79	-1.30
5CO_2	$[Omim]^+[(PFBu)SO_3]^-$	Anion-Cation	C-H---O	1	0.26	0.82	0.41	0.75	-0.38
5CO_2	$[Omim]^+[(PFBu)SO_3]^-$	Anion-Cation	C---O	1	0.50	1.89	1.00	0.73	-0.86
5CO_2	$[Omim]^+[(PFBu)SO_3]^-$	Anion-Cation	F---F	2	1.29	6.90	3.42	0.75	-3.27
5CO_2	$[Omim]^+[(PFBu)SO_3]^-$	Anion-CO_2	F---O	8	0.66	3.00	1.41	0.77	-1.47
5CO_2	$[Omim]^+[(PFBu)SO_3]^-$	Anion-CO_2	C---O	1	1.11	4.55	1.59	0.84	-2.57
5CO_2	$[Omim]^+[(PFBu)SO_3]^-$	Anion-CO_2	C---F	1	0.96	4.36	1.62	0.83	-2.41
5CO_2	$[Omim]^+[(PFBu)SO_3]^-$	Anion-CO_2	O---O	1	0.38	1.35	0.60	0.78	-0.68
5CO_2	$[Omim]^+[(PFBu)SO_3]^-$	Cation-CO_2	C-H---O	4	0.61	2.25	1.06	0.76	-1.10
5CO_2	$[Omim]^+[(PFBu)SO_3]^-$	Cation-CO_2	C---O	1	0.85	3.59	1.72	0.76	-1.74
5CO_2	$[Omim]^+[(PFBu)SO_3]^-$	CO_2-CO_2	O---O	4	0.77	3.42	1.66	0.76	-1.64

FIGURES

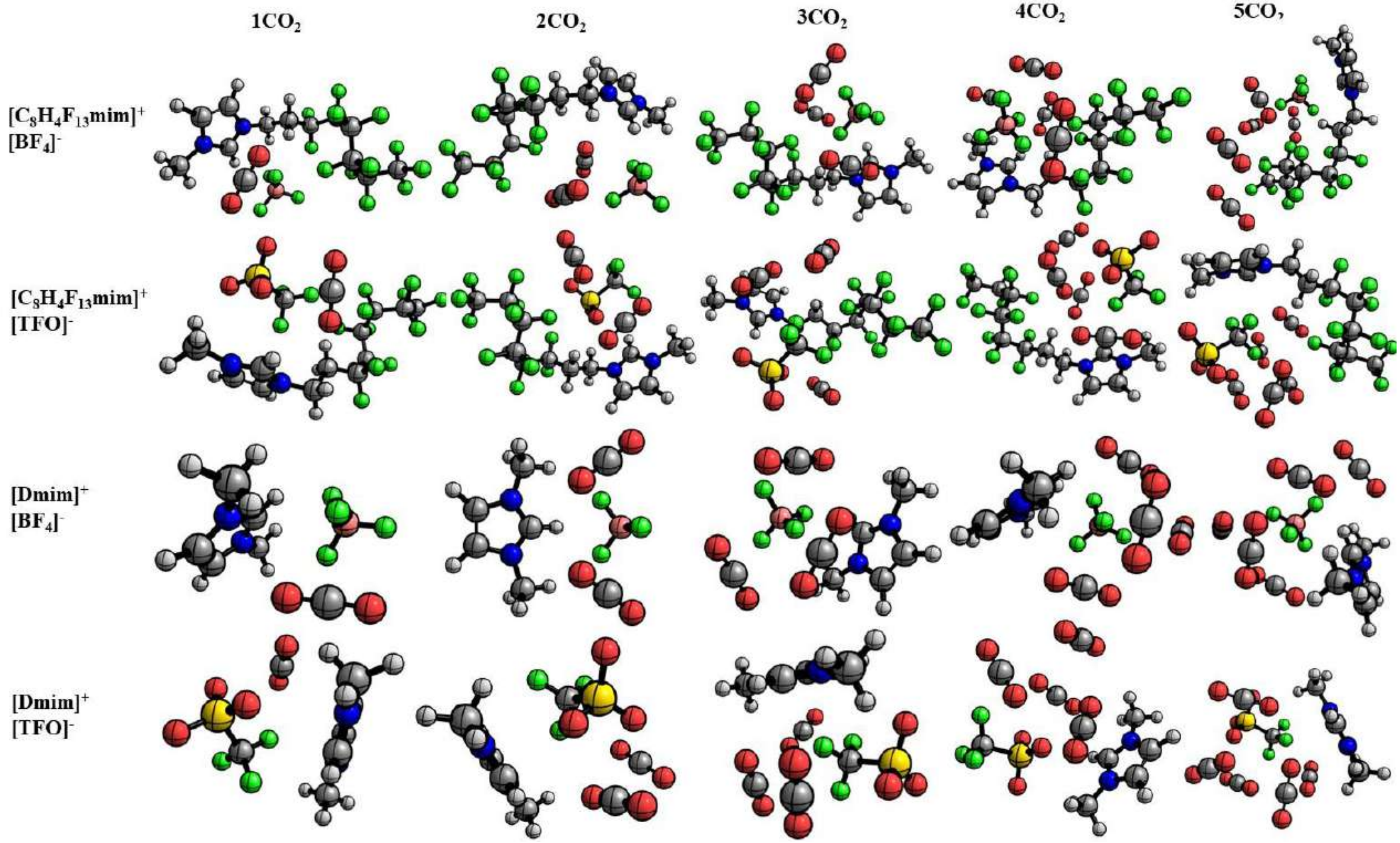

Figure S1. Putative global minimum-energy structures for the $nCO_2[C_8H_4F_{13}mim]^+[BF_4]^-$, $nCO_2[C_8H_4F_{13}mim]^+[TFO]^-$, $nCO_2[Dmim]^+[BF_4]^-$, $nCO_2[Dmim]^+[TFO]^-$ molecular clusters. Atoms color scheme: carbon (grey), nitrogen (blue), sulphur (yellow), phosphorus (orange), fluorine (green), and hydrogen (white) at a level of theory M06-2X/cc-pVTZ (D3, SMD).

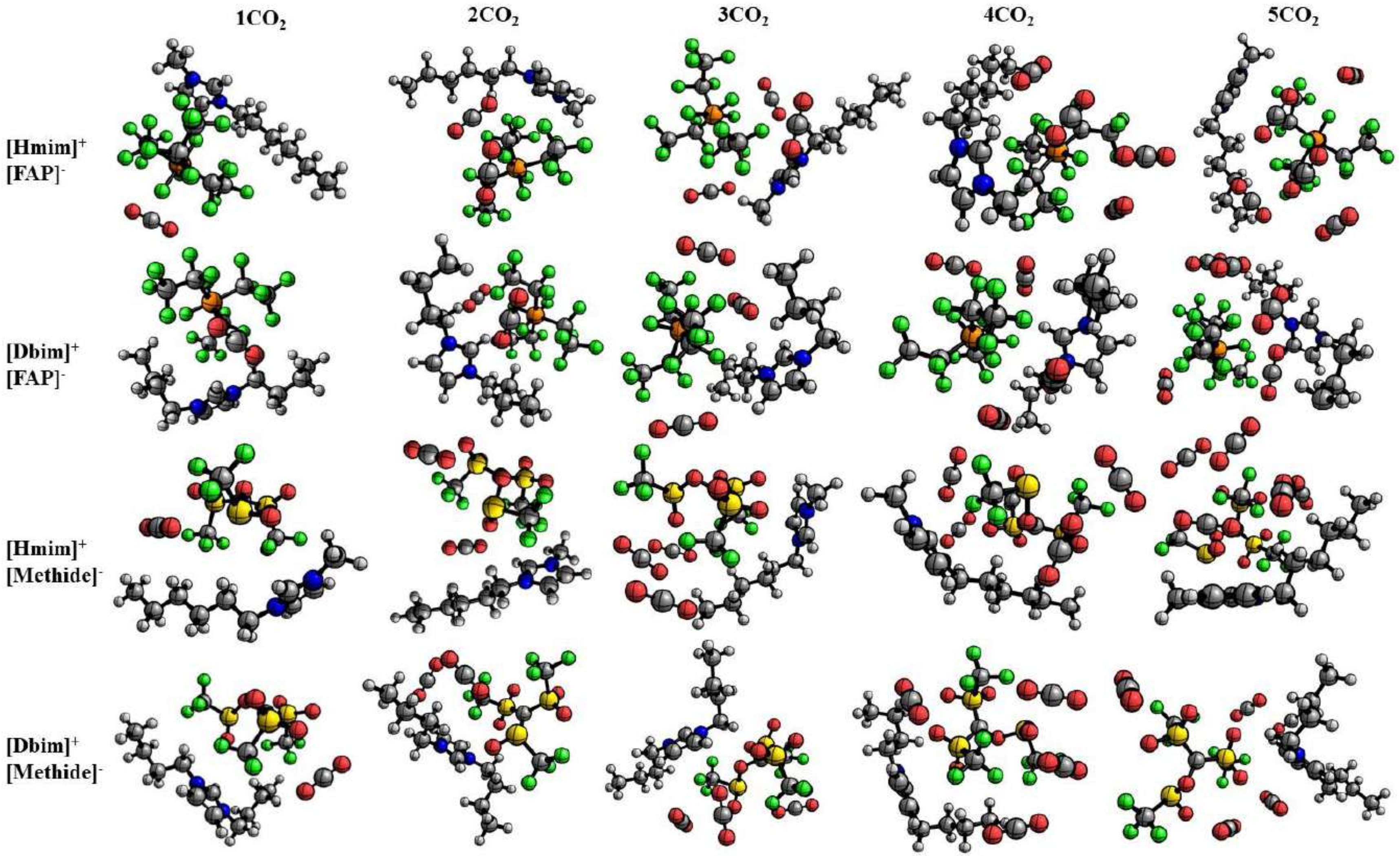

Figure S2. Putative global minimum-energy structures for the $nCO_2[Hmim]^+[FAP]^-$, $nCO_2[Dbim]^+[FAP]^-$, $nCO_2[Hmim]^+[Methide]^-$, $nCO_2[Dbim]^+[Methide]^-$ molecular clusters. Atoms color scheme: carbon (grey), nitrogen (blue), sulphur (yellow), phosphorus (orange), fluorine (green), and hydrogen (white) at a level of theory M06-2X/cc-pVTZ (D3, SMD).

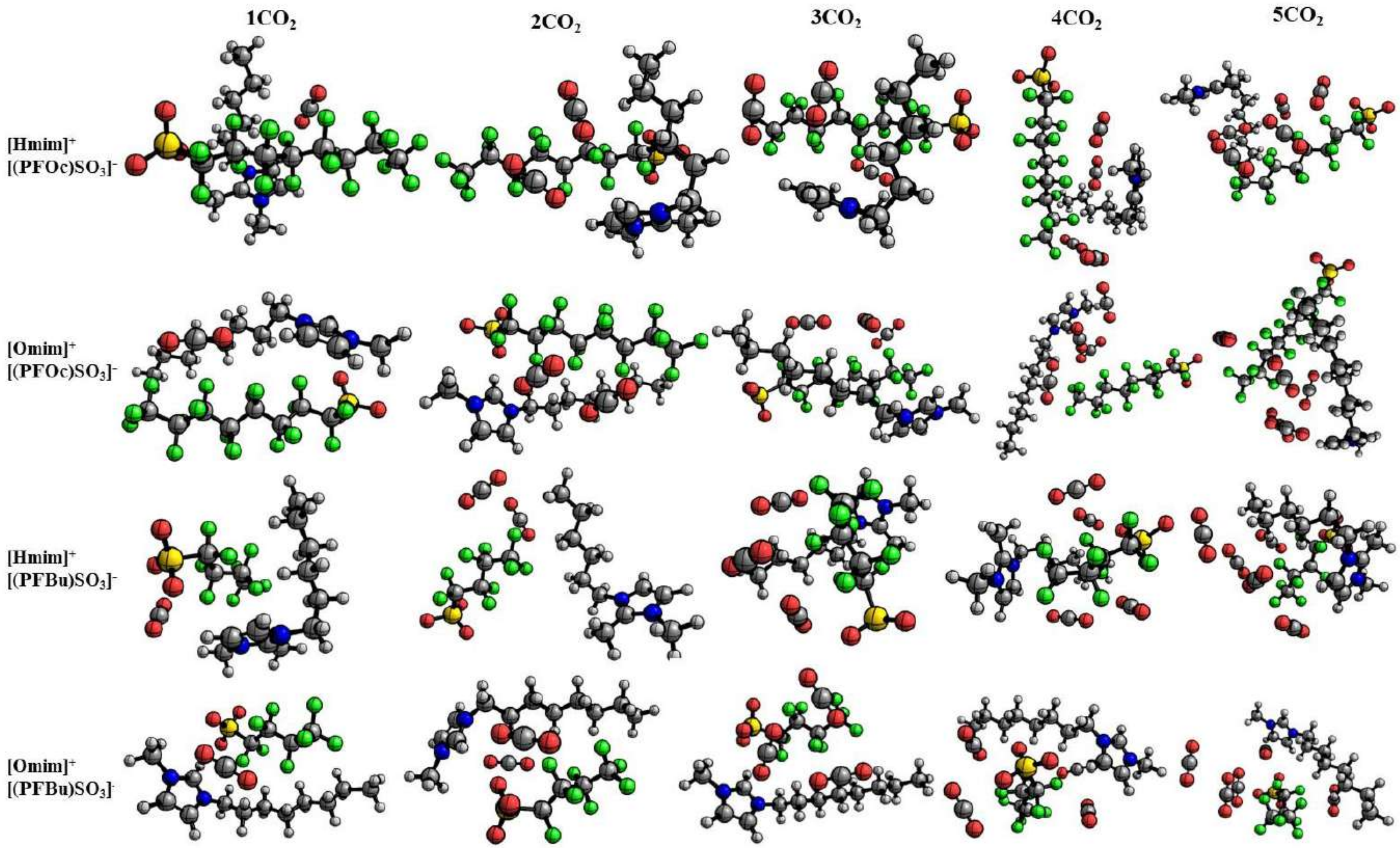

Figure S3. Putative global minimum-energy structures for the $nCO_2[Hmim]^+[(PFOc)SO_3]^-$, $nCO_2[Omim]^+[(PFOc)SO_3]^-$, nCO_2 $[Hmim]^+[(PFBu)SO_3]^-$, $nCO_2[Omim]^+[(PFBu)SO_3]^-$ molecular clusters. Atoms color scheme: carbon (grey), nitrogen (blue), sulphur (yellow), phosphorus (orange), fluorine (green), and hydrogen (white) at a level of theory M06-2X/cc-pVTZ (D3, SMD).

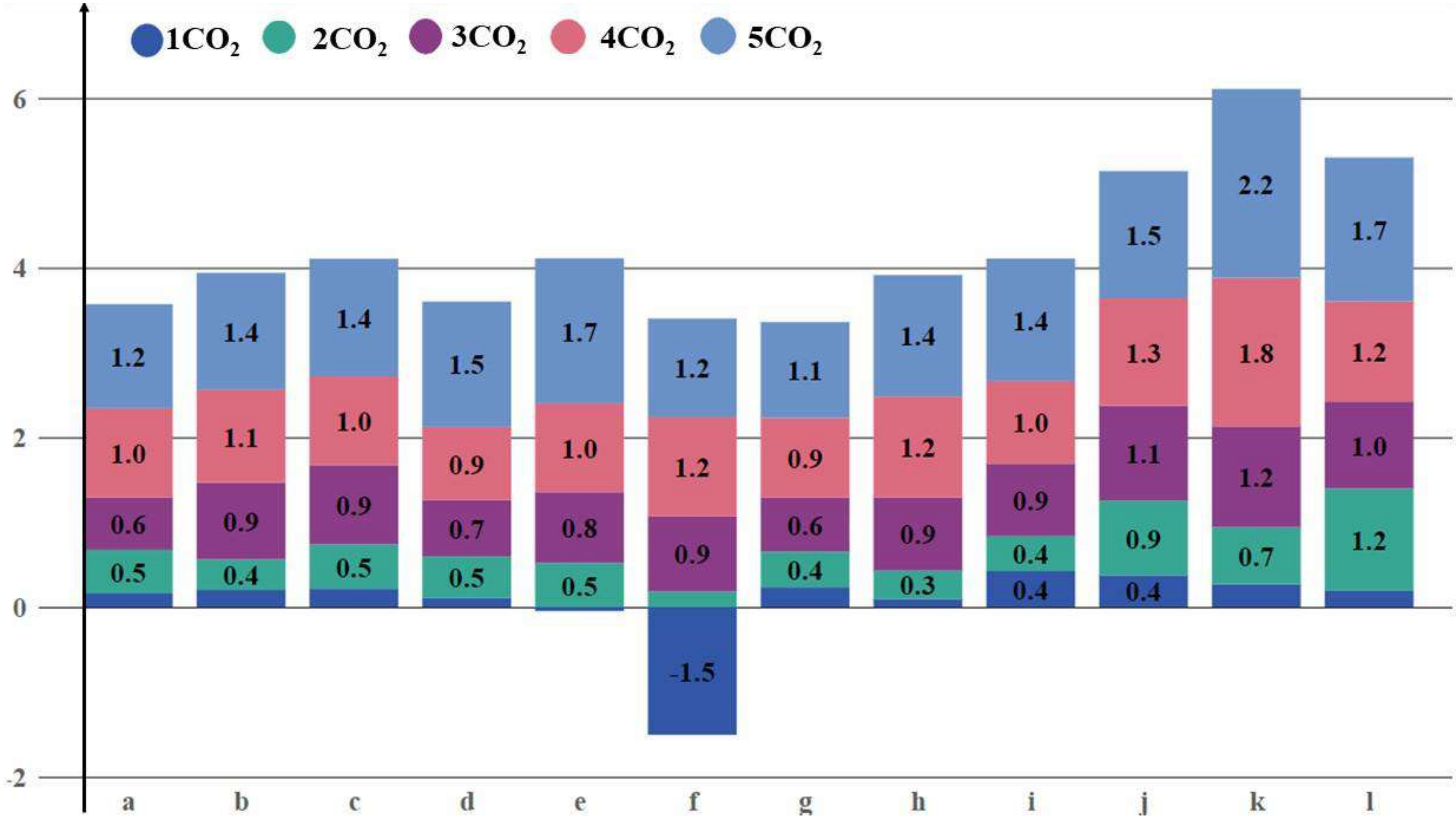

Figure S4. Difference between ΔH-ΔE of the clusters nCO_2IL; n = 1 a 5; IL = a: $[Dbim]^+[FAP]^-$, b: $[C_8H_4F_{13}mim]^+[TFO]^-$, c: $[Hmim]^+[FAP]^-$, d: $[C_8H_4F_{13}mim]^+[BF4]^-$, e: $[Dbim]^+[Methide]^-$, f: $[Hmim]^+[Methide]^-$, g: $[Dmim]^+[BF_4]^-$, h: $[Omim]^+[(PFBu)SO_3]^-$, i: $[Dmim]^+[TFO]^-$, j: $[Hmim]^+[(PFOc)SO_3]^-$, k: $[Omim]^+[(PFOc)SO_3]^-$, l: $[Hmim]^+[(PFBu)SO_3]^-$. ΔE and ΔH in kcal/mol.

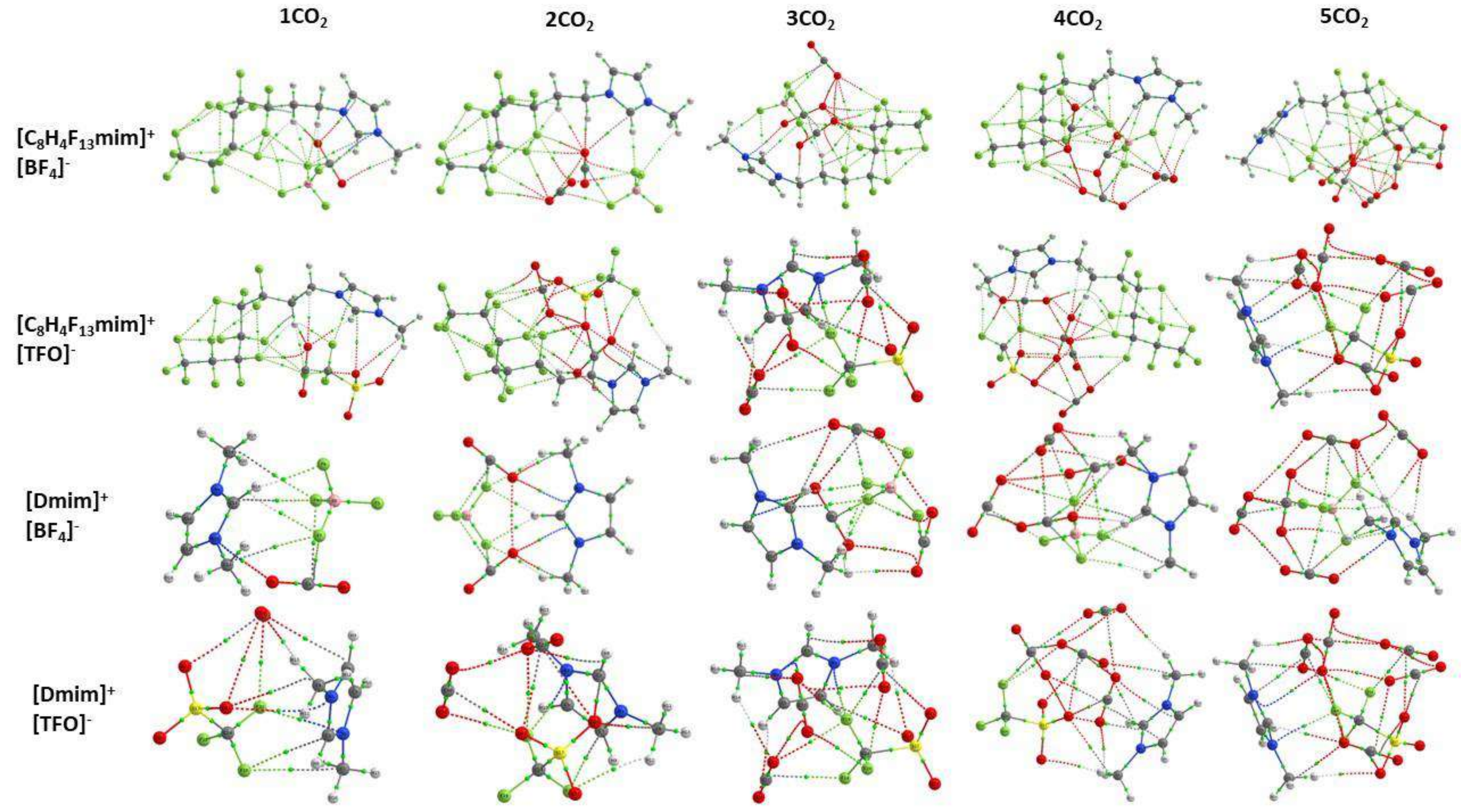

Figure S5. Molecular graphs and Bond critical points (BCP) for the $nCO_2[C_8H_4F_{13}mim]^+[BF4]^-$, $nCO_2[C_8H_4F_{13}mim]^+[TFO]^-$, $nCO_2[Dmim]^+[BF_4]^-$, $nCO_2[Dmim]^+[TFO]^-$ molecular clusters. Atoms color scheme: carbon (grey), nitrogen (blue), sulphur (yellow), phosphorus (orange), fluorine (green), and hydrogen (white). BCP are shown as green spheres. Bond paths are drawn as dashed lines paths at a level of theory M06-2X/cc-pVTZ (D3, SMD).

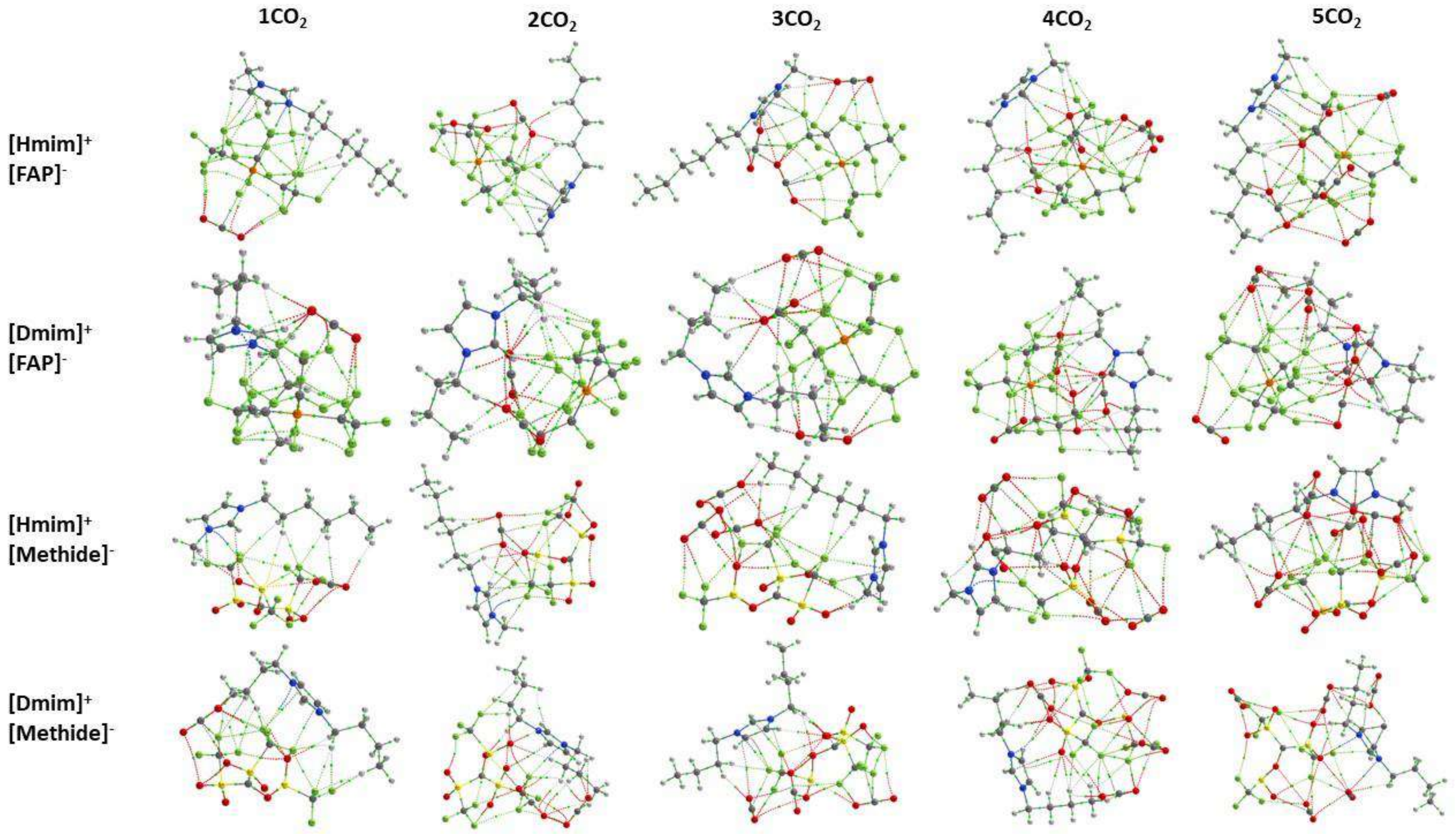

Figure S6. Molecular graphs and Bond critical points (BCP) for the nCO_2[Hmim]$^+$[FAP]$^-$, nCO_2[Dbim]$^+$[FAP]$^-$, nCO_2[Hmim]$^+$[Methide]$^-$, nCO_2[Dbim]$^+$[Methide]$^-$ molecular clusters. Atoms color scheme: carbon (grey), nitrogen (blue), sulphur (yellow), phosphorus (orange), fluorine (green), and hydrogen (white). BCP are shown as green spheres. Bond paths are drawn as dashed lines paths at a level of theory M06-2X/cc-pVTZ (D3, SMD).

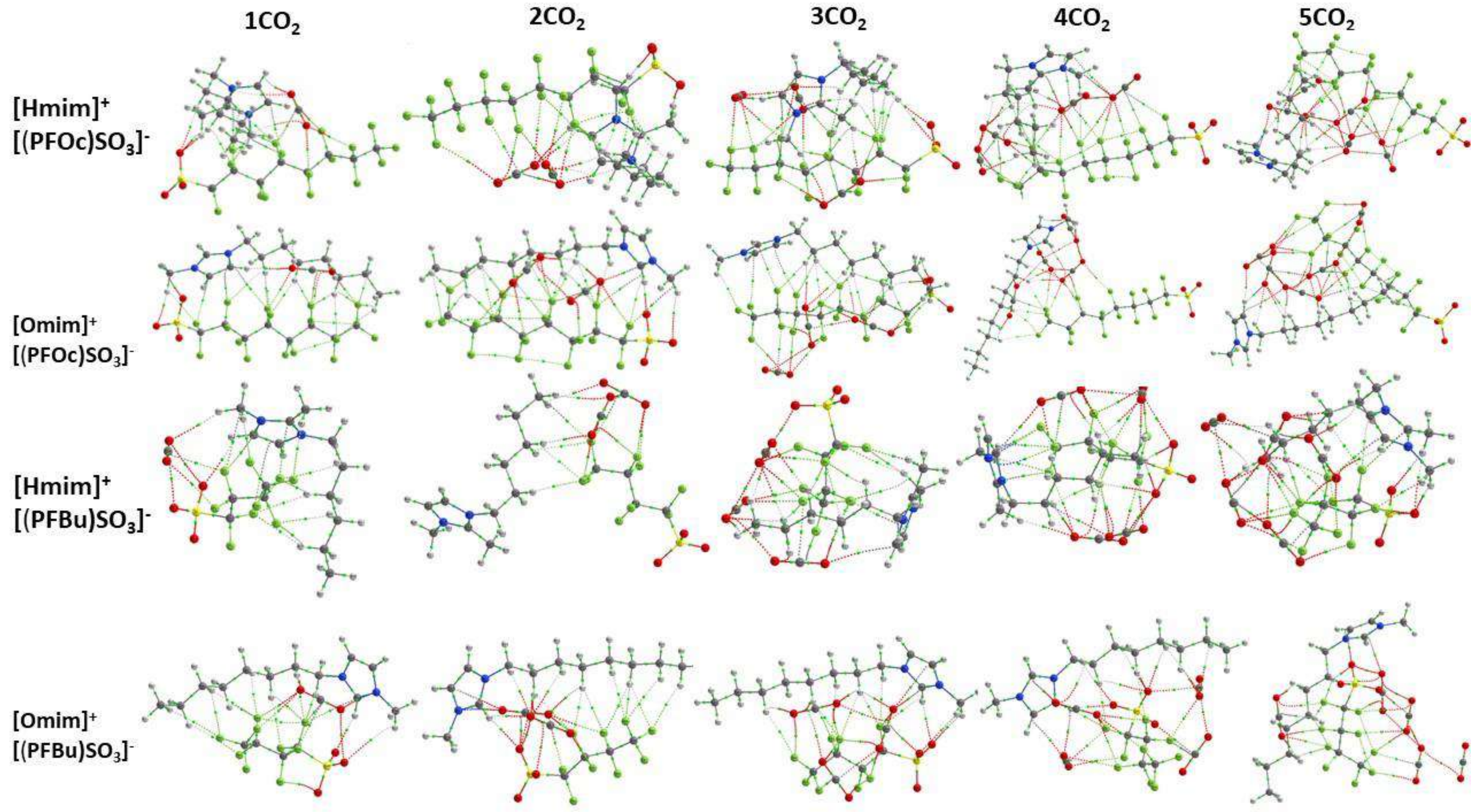

Figure S7. Molecular graphs and Bond critical points (BCP) for the $nCO_2[Hmim]^+[(PFOc)SO_3]^-$, $nCO_2[Omim]^+[(PFOc)SO_3]^-$, nCO_2 $[Hmim]^+[(PFBu)SO_3]^-$, $nCO_2[Omim]^+[(PFBu)SO_3]^-$molecular clusters. Atoms color scheme: carbon (grey), nitrogen (blue), sulphur (yellow), phosphorus (orange), fluorine (green), and hydrogen (white). BCP are shown as green spheres. Bond paths are drawn as dashed lines paths at a level of theory M06-2X/cc-pVTZ (D3, SMD).

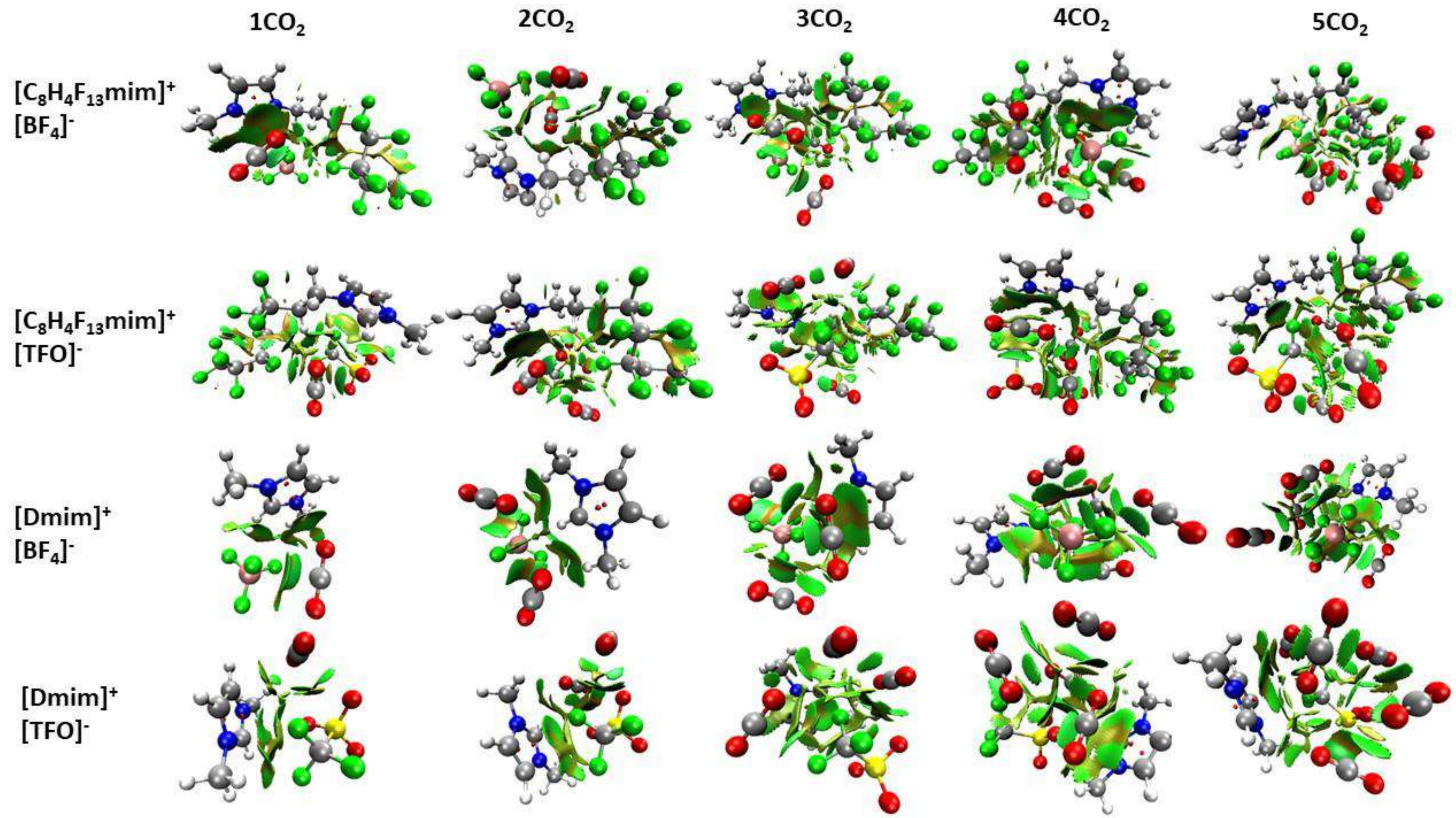

Figure S8. NCI plot isosurfaces (0.6 a.u) of the non-covalent interactions for $nCO_2[C_8H_4F_{13}mim]^+[BF4]^-$, $nCO_2[C_8H_4F_{13}mim]^+[TFO]^-$, $nCO_2[Dmim]^+[BF_4]^-$, $nCO_2[Dmim]^+[TFO]^-$ molecular clusters. Atoms color scheme: carbon (grey), nitrogen (blue), sulphur (yellow), phosphorus (orange), fluorine (green), and hydrogen (white). Green areas correspond to dispersive weak interactions.

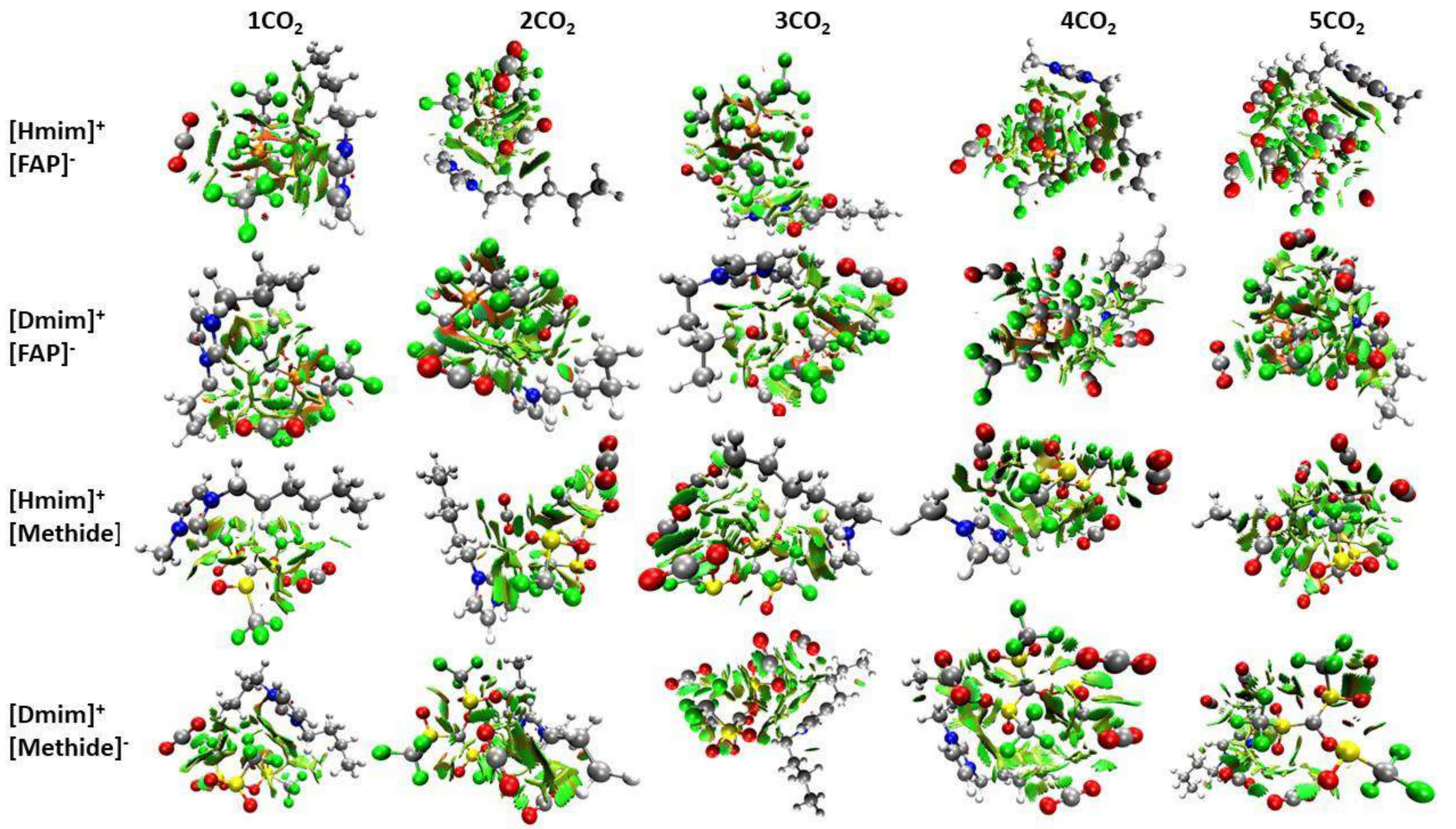

Figure S9. NCI plot isosurfaces (0.6 a.u) of the non-covalent interactions for the $nCO_2[Hmim]^+[FAP]^-$, $nCO_2[Dbim]^+[FAP]^-$, $nCO_2[Hmim]^+[Methide]^-$, $nCO_2[Dbim]^+[Methide]^-$ molecular clusters. Atoms color scheme: carbon (grey), nitrogen (blue), sulphur (yellow), phosphorus (orange), fluorine (green areas), and hydrogen (white). Green areas correspond to dispersive weak interactions.

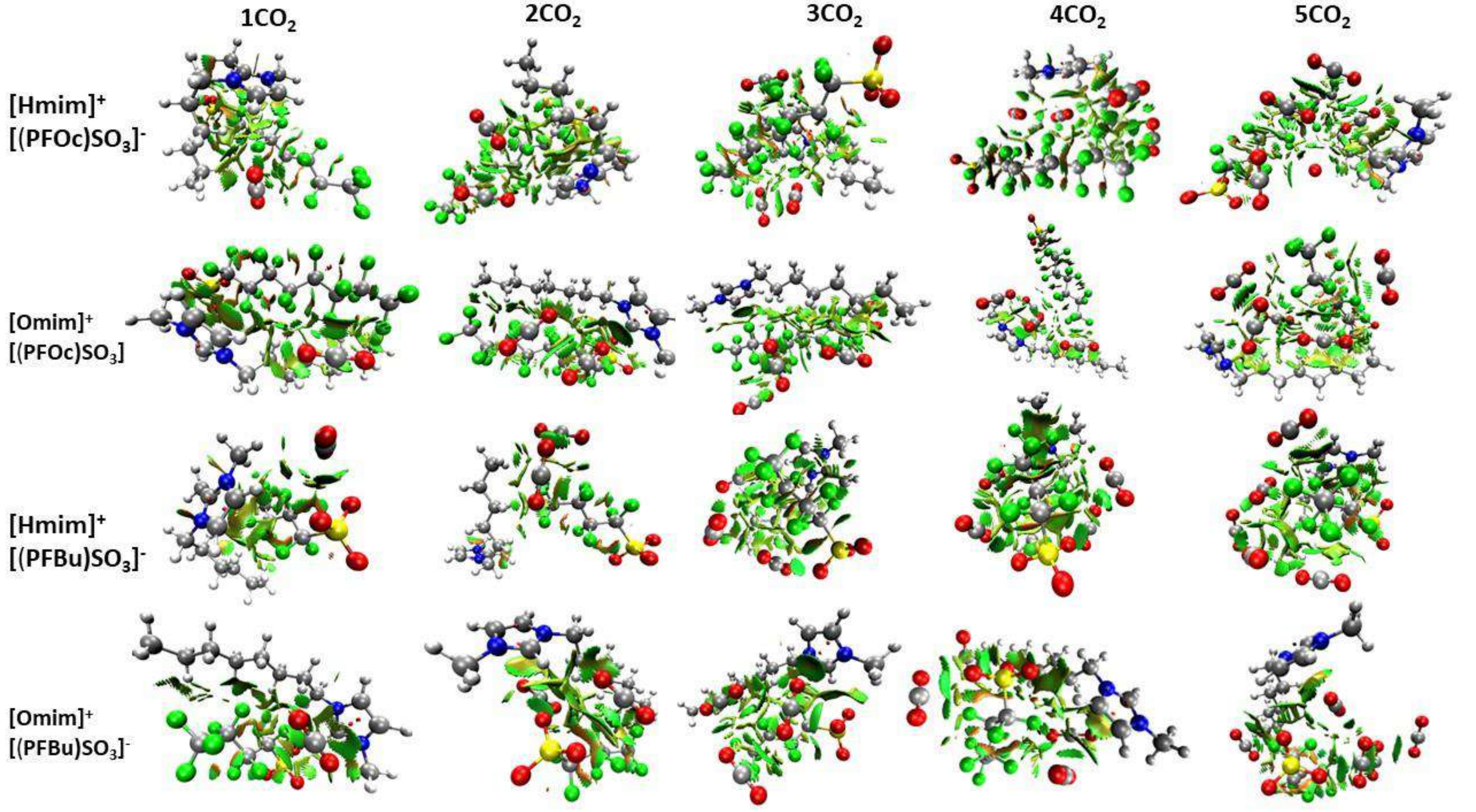

Figure S10. NCI plot isosurfaces (0.6 a.u) of the non-covalent interactions for the $nCO_2[Hmim]^+[(PFOc)SO_3]^-$, nCO_2 $[Hmim]^+[(PFBu)SO_3]^-$, $nCO_2[Omim]^+[(PFBu)SO_3]^-$, molecular clusters. Atoms color scheme: carbon (grey), nitrogen (blue), sulphur (yellow), phosphorus (orange), fluorine (green), and hydrogen (white). BCP are shown as green spheres. Bond paths are drawn as dashed lines paths at a level of theory M06-2X/cc-pVTZ (D3, SMD). Green areas correspond to dispersive weak interactions.

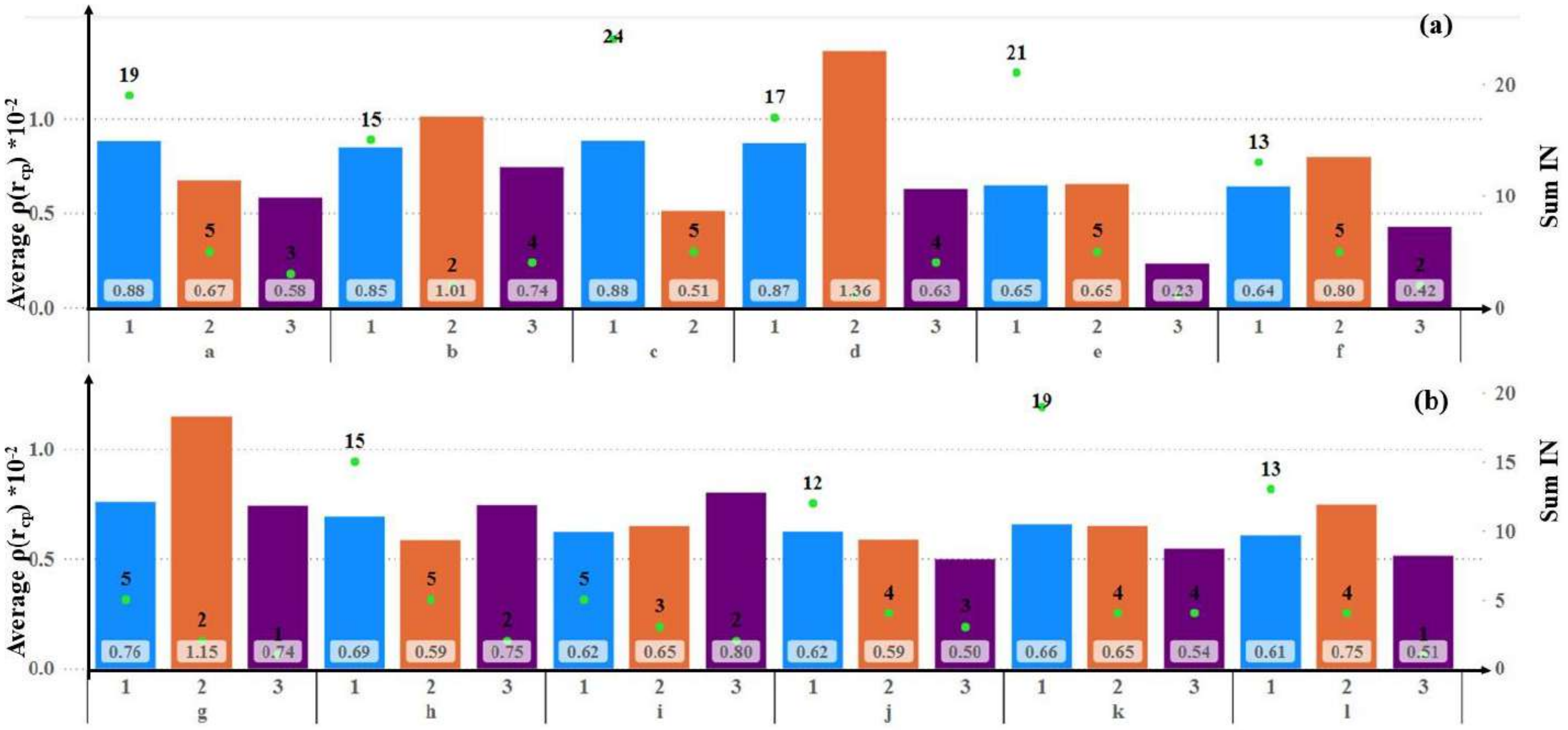

Figure S11. Average values of electron density $\rho(r_{cp})$ and number of interactions (IN) for the different types of interactions: 1 = Anion-Cation, 2 = Anion-CO_2, 3 = Cation-CO_2, 4 = CO_2-CO_2 for the clusters with 1CO2. (a) Plot for the clusters with IL a: $[Dbim]^+[FAP]^-$, b: $[C_8H_4F_{13}mim]^+[TFO]^-$, c: $[Hmim]^+[FAP]^-$, d: $[C_8H_4F_{13}mim]^+[BF_4]^-$, e: $[Dbim]^+[Methide]^-$, f: $[Hmim]^+[Methide]^-$. (b) Plot for the clusters with g: $[Dmim]^+[BF_4]^-$, h: $[Omim]^+[(PFBu)SO_3]^-$, i: $[Dmim]^+[TFO]^-$, j: $[Hmim]^+[(PFOc)SO_3]^-$, k: $[Omim]^+[(PFOc)SO_3]^-$, l: $[Hmim]^+[(PFBu)SO_3]^-$.

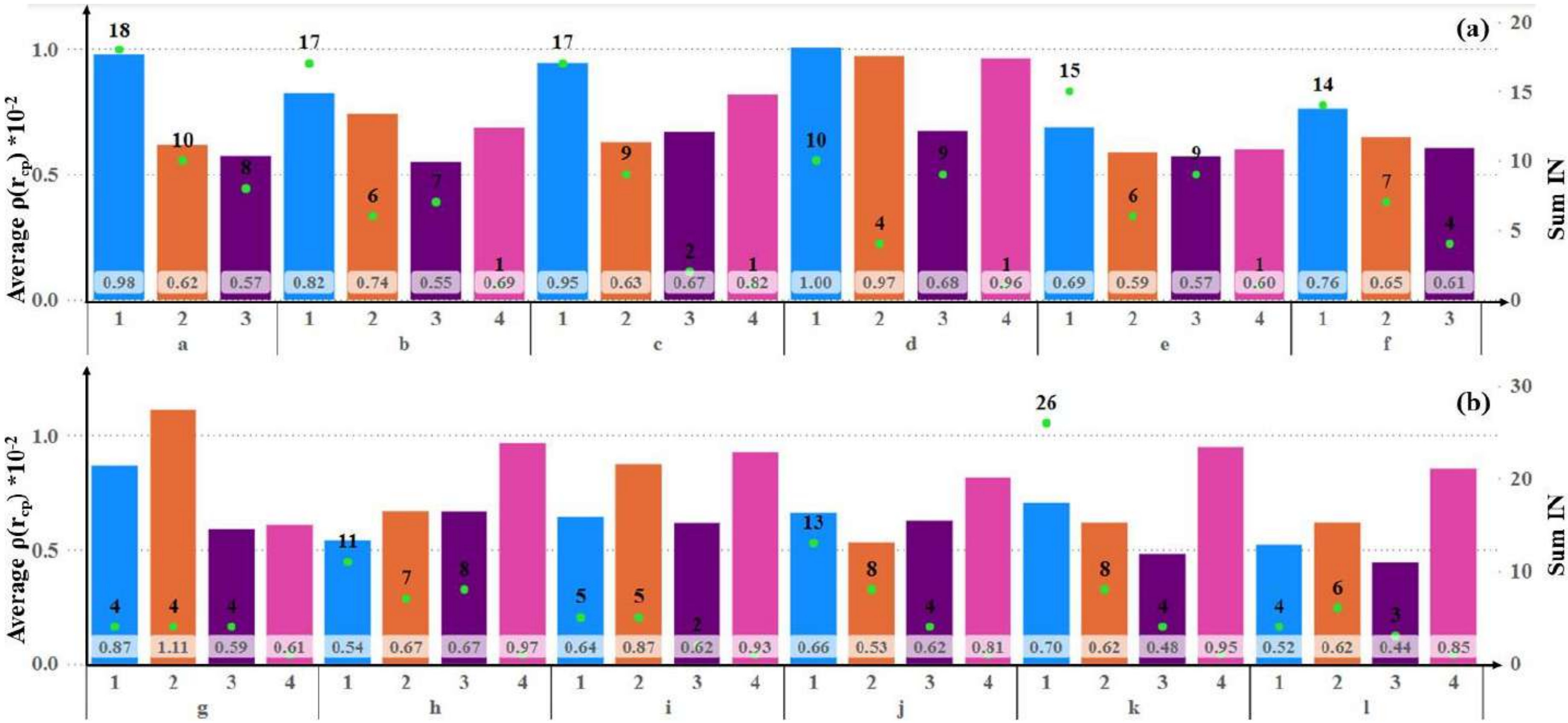

Figure S12. Average values of electron density $\rho(r_{cp})$ and number of interactions (IN) for the different types of interactions: 1 = Anion-Cation, 2 = Anion-CO_2, 3 = Cation-CO_2, 4 = CO_2-CO_2 for the clusters with 2CO2. (a) Plot for the clusters with IL a: $[Dbim]^+[FAP]^-$, b: $[C_8H_4F_{13}mim]^+[TFO]^-$, c: $[Hmim]^+[FAP]^-$, d: $[C_8H_4F_{13}mim]^+[BF_4]^-$, e: $[Dbim]^+[Methide]^-$, f: $[Hmim]^+[Methide]^-$. (b) Plot for the clusters with IL g: $[Dmim]^+[BF_4]^-$, h: $[Omim]^+[(PFBu)SO_3]^-$, i: $[Dmim]^+[TFO]^-$, j: $[Hmim]^+[(PFOc)SO_3]^-$, k: $[Omim]^+[(PFOc)SO_3]^-$, l: $[Hmim]^+[(PFBu)SO_3]^-$.

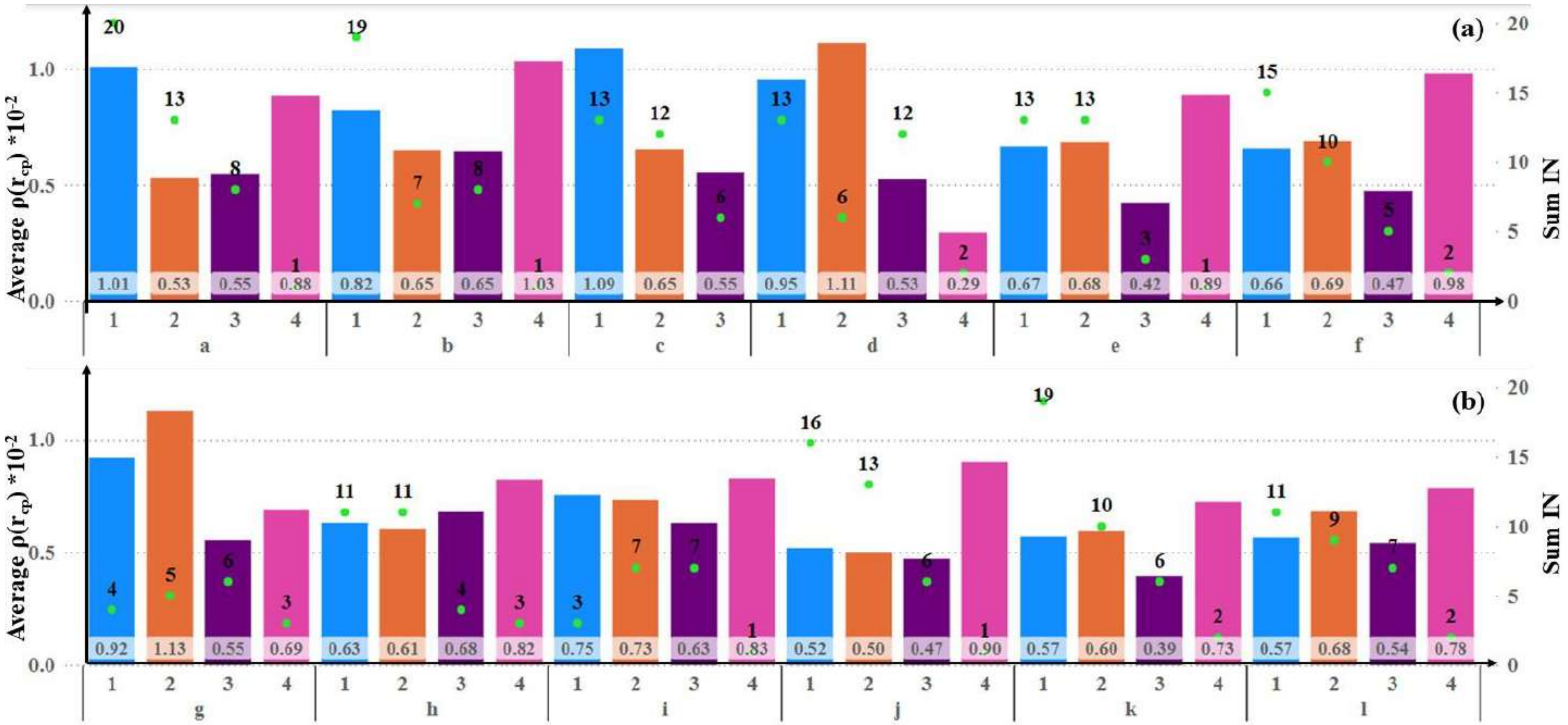

Figure S13. Average values of electron density $\rho(r_{cp})$ and number of interactions (IN) for the different types of interactions: 1 = Anion-Cation, 2 = Anion-CO_2, 3 = Cation-CO_2, 4 = CO_2-CO_2 for the clusters with 3CO2. (a) Plot for the clusters with IL a: $[Dbim]^+[FAP]^-$, b: $[C_8H_4F_{13}mim]^+[TFO]^-$, c: $[Hmim]^+[FAP]^-$, d: $[C_8H_4F_{13}mim]^+[BF_4]^-$, e: $[Dbim]^+[Methide]^-$, f: $[Hmim]^+[Methide]^-$. (b) Plot for the clusters with IL g: $[Dmim]^+[BF_4]^-$, h: $[Omim]^+[(PFBu)SO_3]^-$, i: $[Dmim]^+[TFO]^-$, j: $[Hmim]^+[(PFOc)SO_3]^-$, k: $[Omim]^+[(PFOc)SO_3]^-$, l: $[Hmim]^+[(PFBu)SO_3]^-$.

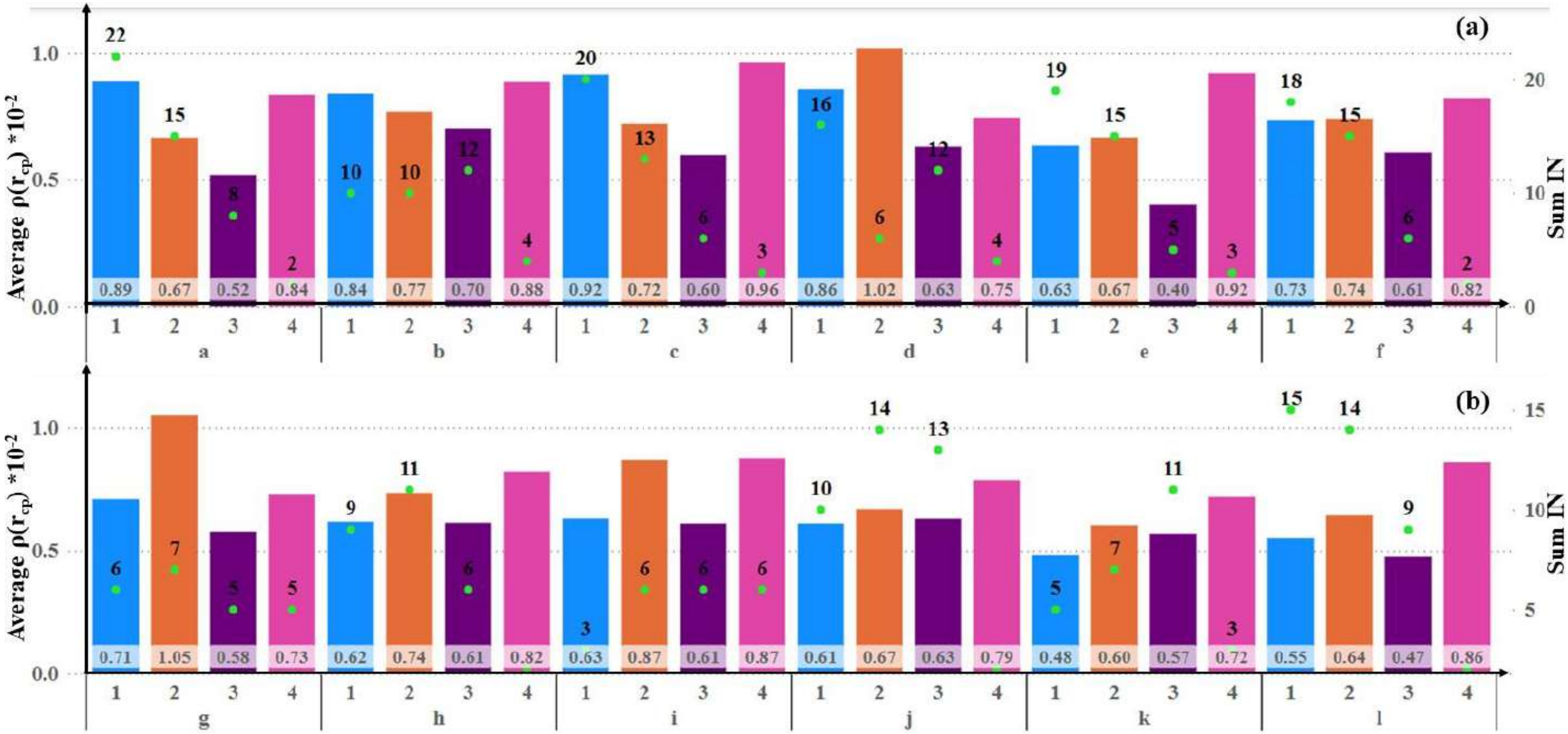

Figure S14. Average values of electron density $\rho(r_{cp})$ and number of interactions (IN) for the different types of interactions: 1 = Anion-Cation, 2 = Anion-CO_2, 3 = Cation-CO_2, 4 = CO_2-CO_2 for the clusters with 4CO2. (a) Plot for the clusters with IL a: $[Dbim]^+[FAP]^-$, b: $[C_8H_4F_{13}mim]^+[TFO]^-$, c: $[Hmim]^+[FAP]^-$, d: $[C_8H_4F_{13}mim]^+[BF_4]^-$, e: $[Dbim]^+[Methide]^-$, f: $[Hmim]^+[Methide]^-$. (b) Plot for the clusters with IL g: $[Dmim]^+[BF_4]^-$, h: $[Omim]^+[(PFBu)SO_3]^-$, i: $[Dmim]^+[TFO]^-$, j: $[Hmim]^+[(PFOc)SO_3]^-$, k: $[Omim]^+[(PFOc)SO_3]^-$, l: $[Hmim]^+[(PFBu)SO_3]^-$.

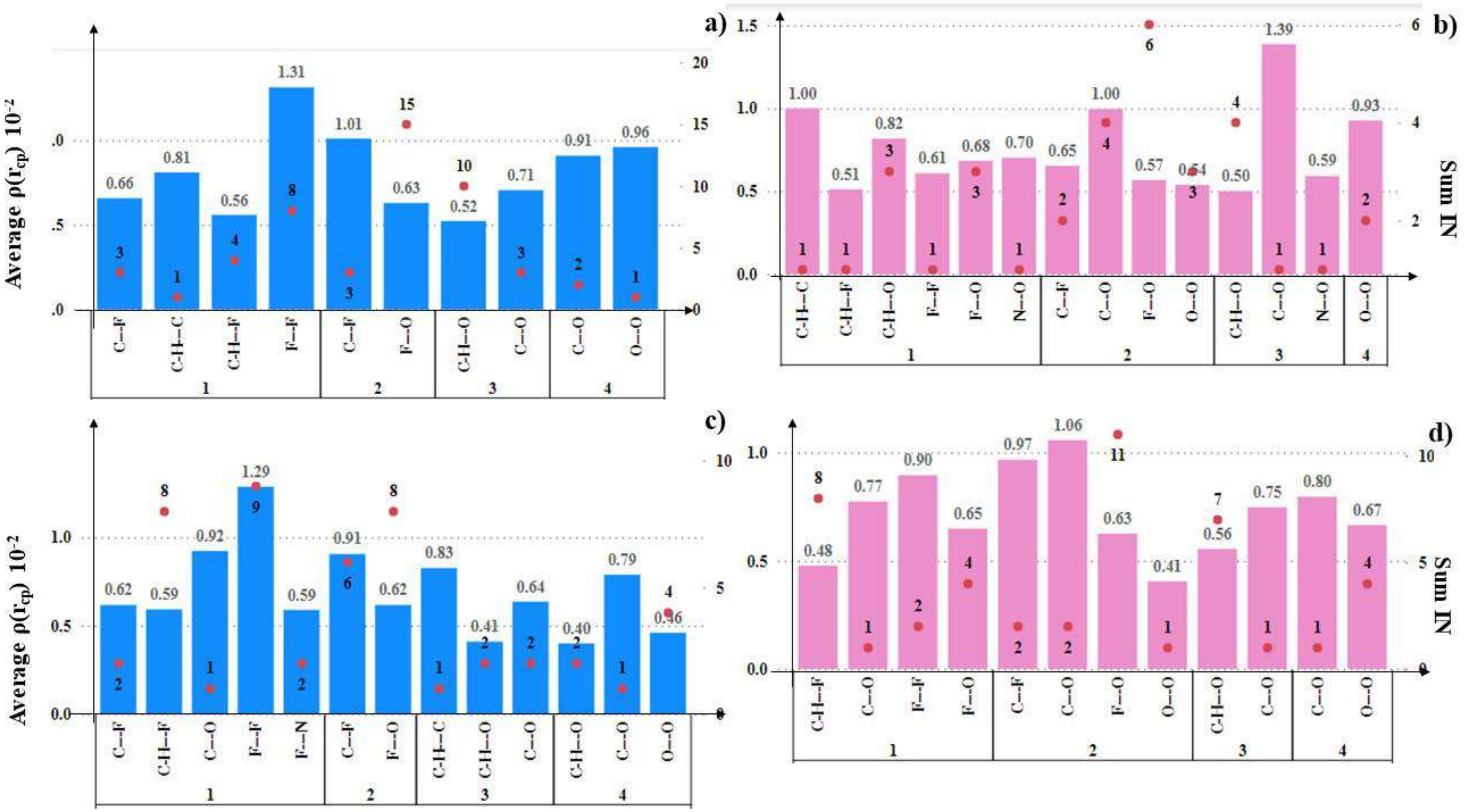

Figure S15. Average values of electron density ρ(rcp) and number of interactions (IN) for the different types of interactions: 1 = Anion-Cation, 2 = Anion-CO_2, 3 = Cation-CO_2, 4 = CO_2-CO_2 per interaction class for 5CO2. a) [Dbim]$^+$[FAP]$^-$, b) [Dbim]$^+$[Methide]$^-$, c) [Hmim]$^+$[FAP]$^-$, d) [Hmim]$^+$[Methide]$^-$ at a level of theory M06-2X/cc-pVTZ (D3, SMD).

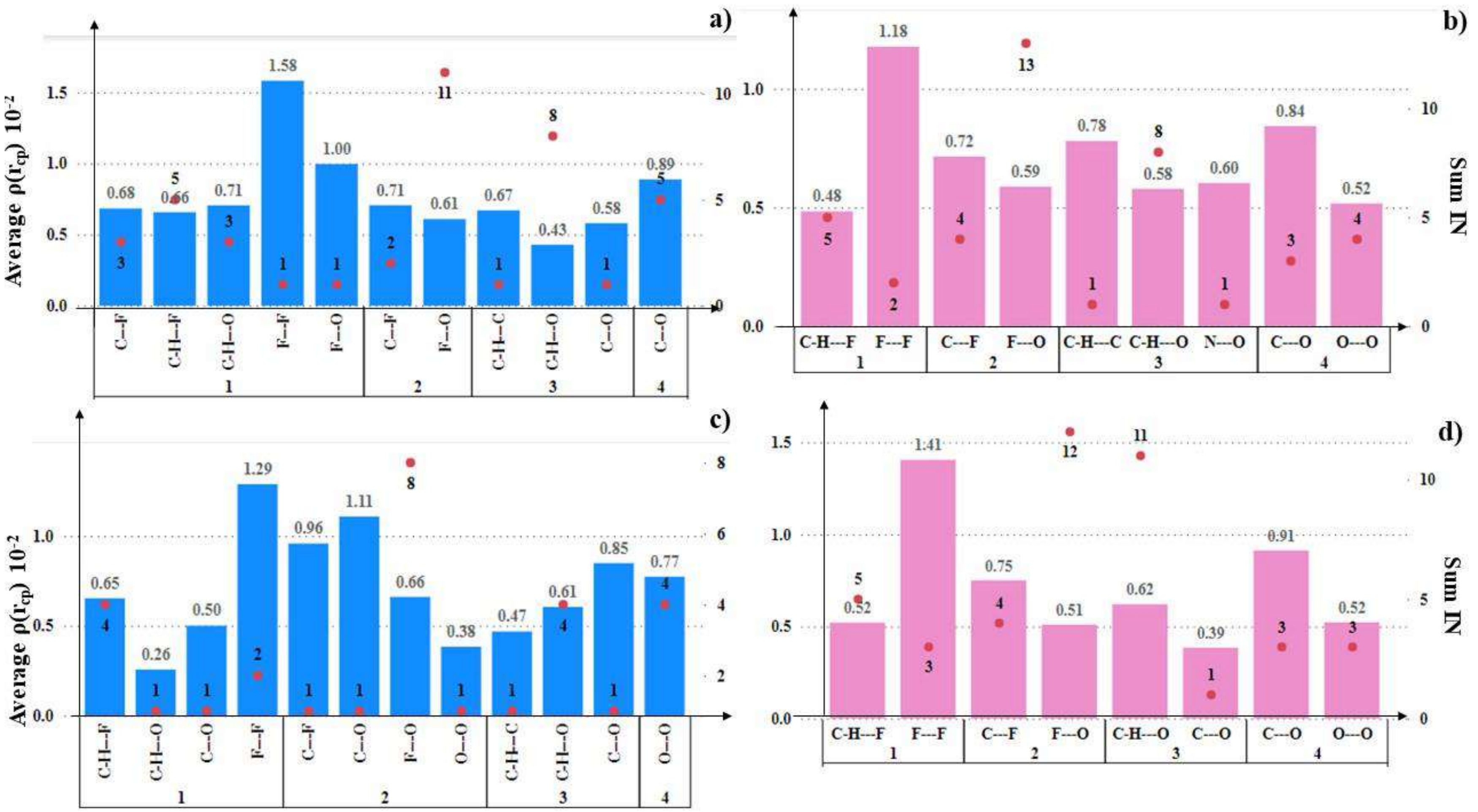

Figure S16. Average values of electron density ρ(rcp) and number of interactions (IN) for the different types of interactions: 1 = Anion-Cation, 2 = Anion-CO_2, 3 = Cation-CO_2, 4 = CO_2-CO_2 per interaction class for 5CO2. a) $[Hmim]^+[(PFOc)SO_3]^-$, b) $[Hmim]^+[(PFBu)SO_3]^-$,, c) $[Omim]^+[(PFOc)SO_3]^-$, d) $[Omim]^+[(PFBu)SO_3]^-$ at a level of theory M06-2X/cc-pVTZ (D3, SMD).

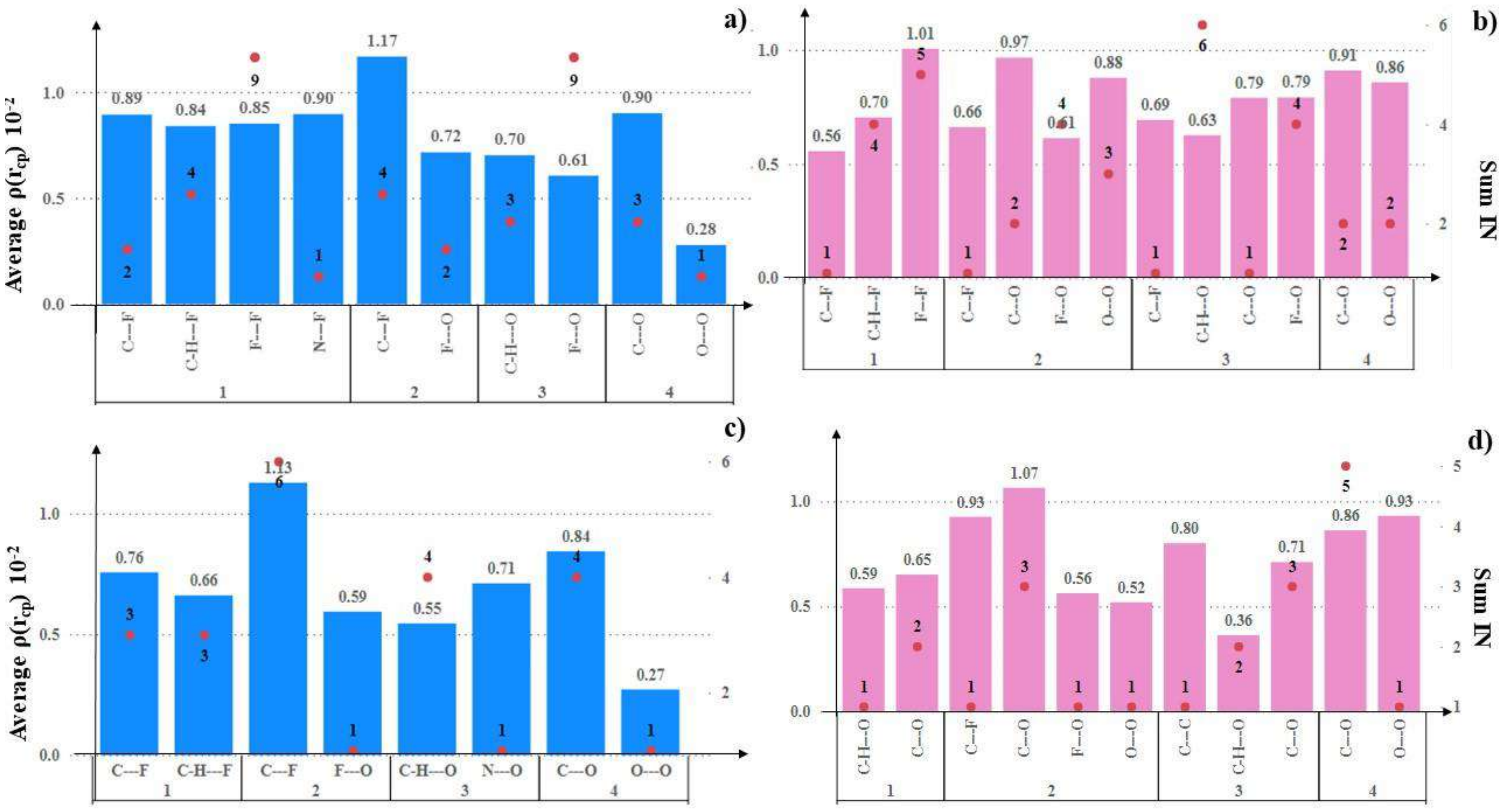

Figure S17. Average values of electron density ρ(rcp) and number of interactions (IN) for the different types of interactions: 1 = Anion-Cation, 2 = Anion-CO_2, 3 = Cation-CO_2, 4 = CO_2-CO_2 per interaction class for 4CO2. a) $[C_8H_4F_{13}mim]^+[BF_4]^-$, b) $[C_8H_4F_{13}mim]^+$ $[TFO]^-$, c) $[Dmim]^+[BF_4]^-$, d) $[Dmim]^+[TFO]^-$ at a level of theory M06-2X/cc-pVTZ (D3, SMD).

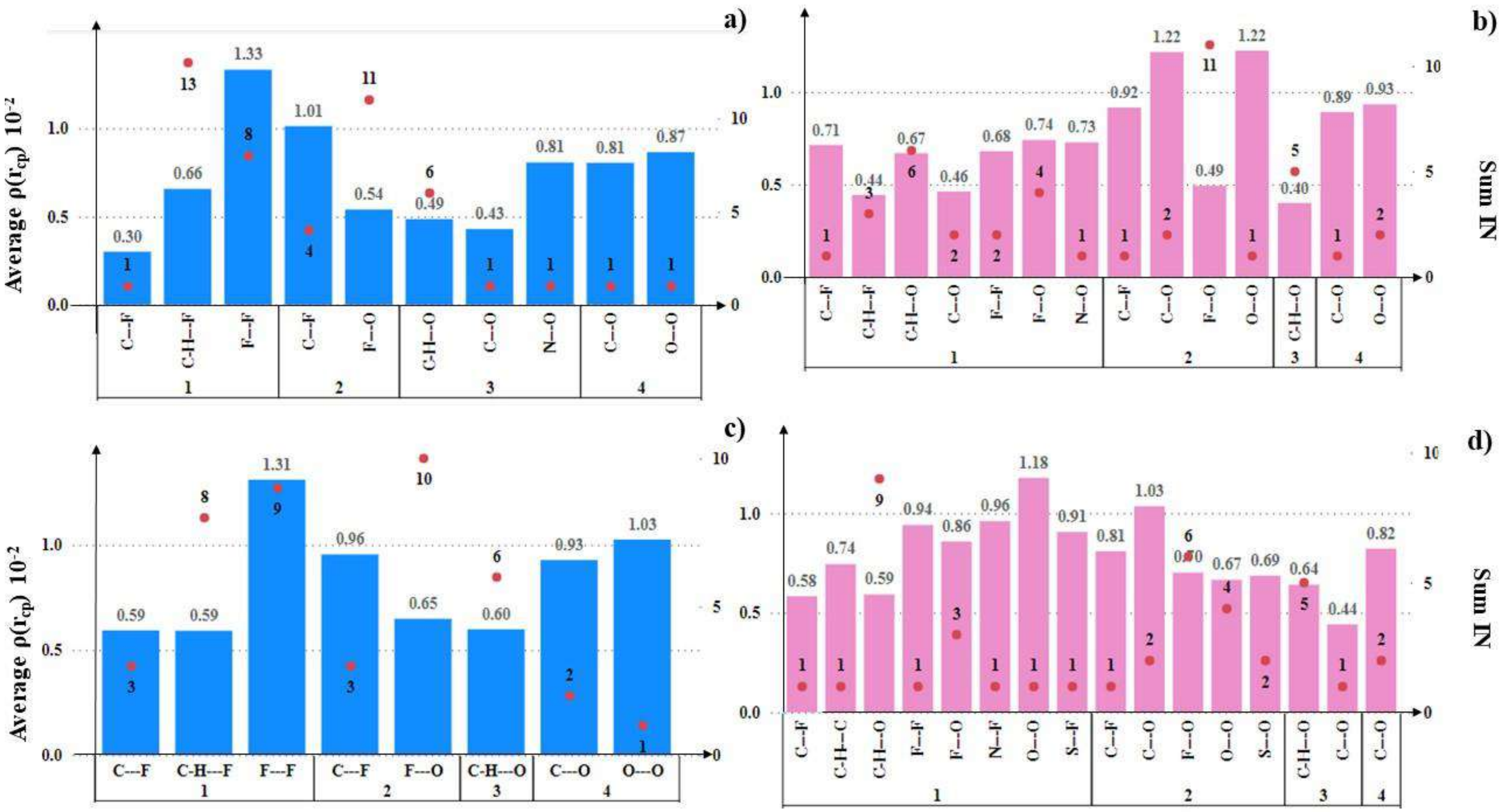

Figure S18. Average values of electron density ρ(rcp) and number of interactions (IN) for the different types of interactions: 1 = Anion-Cation, 2 = Anion-CO2, 3 = Cation-CO2, 4 = CO2-CO2 per interaction class for 4CO2. a) $[Dbim]^+[FAP]^-$, b) $[Dbim]^+[Methide]^-$, c) $[Hmim]^+[FAP]^-$, d) $[Hmim]^+[Methide]^-$ at a level of theory M06-2X/cc-pVTZ (D3, SMD).

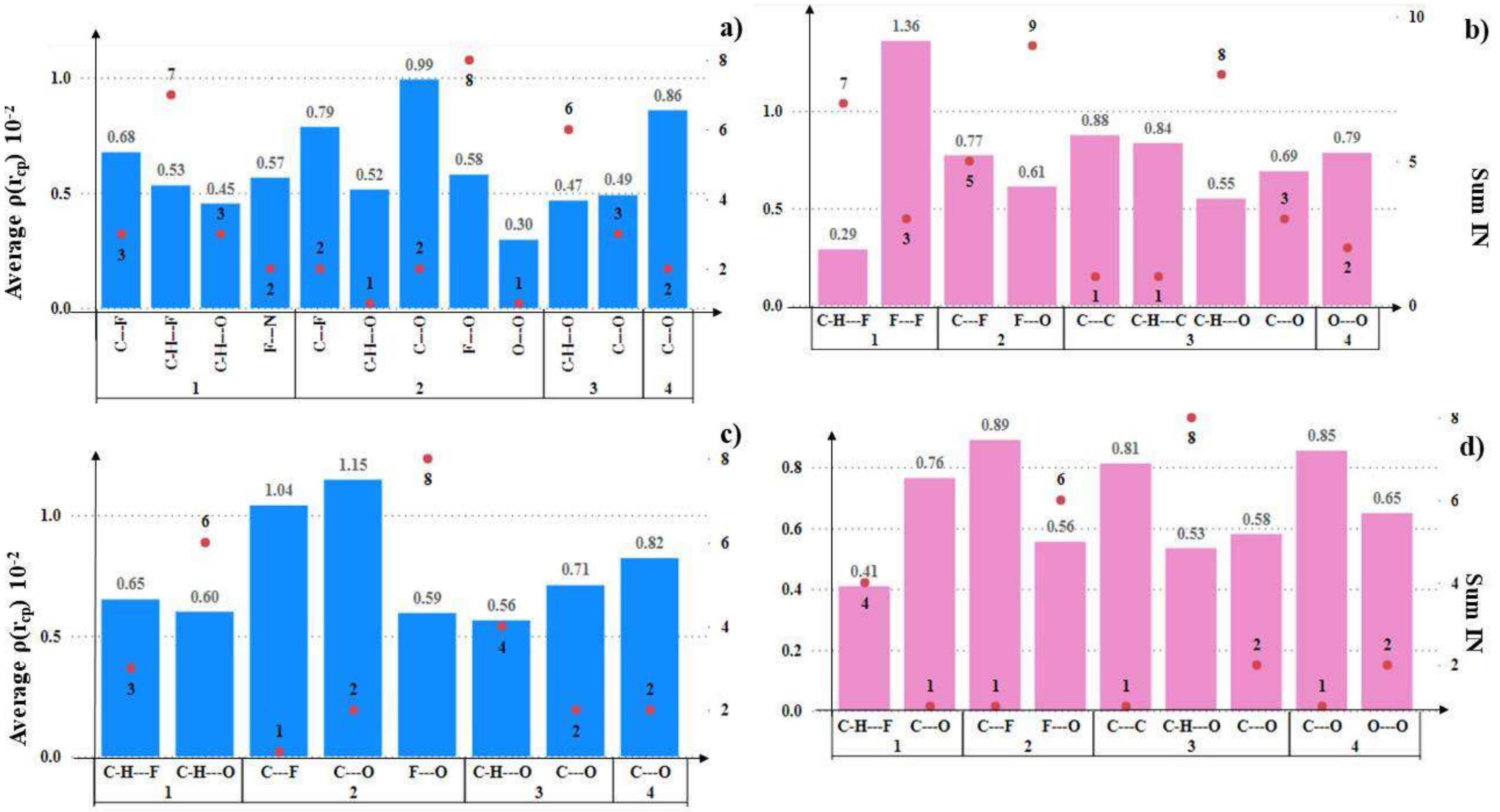

Figure S19. Average values of electron density ρ(rcp) and number of interactions (IN) for the different types of interactions: 1 = Anion-Cation, 2 = Anion-CO_2, 3 = Cation-CO_2, 4 = CO_2-CO_2 per interaction class for 4CO2. a) $[Hmim]^+[(PFOc)SO_3]^-$, b) $[Hmim]^+[(PFBu)SO_3]^-$, c) $[Omim]^+[(PFOc)SO_3]^-$, d) $[Omim]^+[(PFBu)SO_3]^-$ at a level of theory M06-2X/cc-pVTZ (D3, SMD).

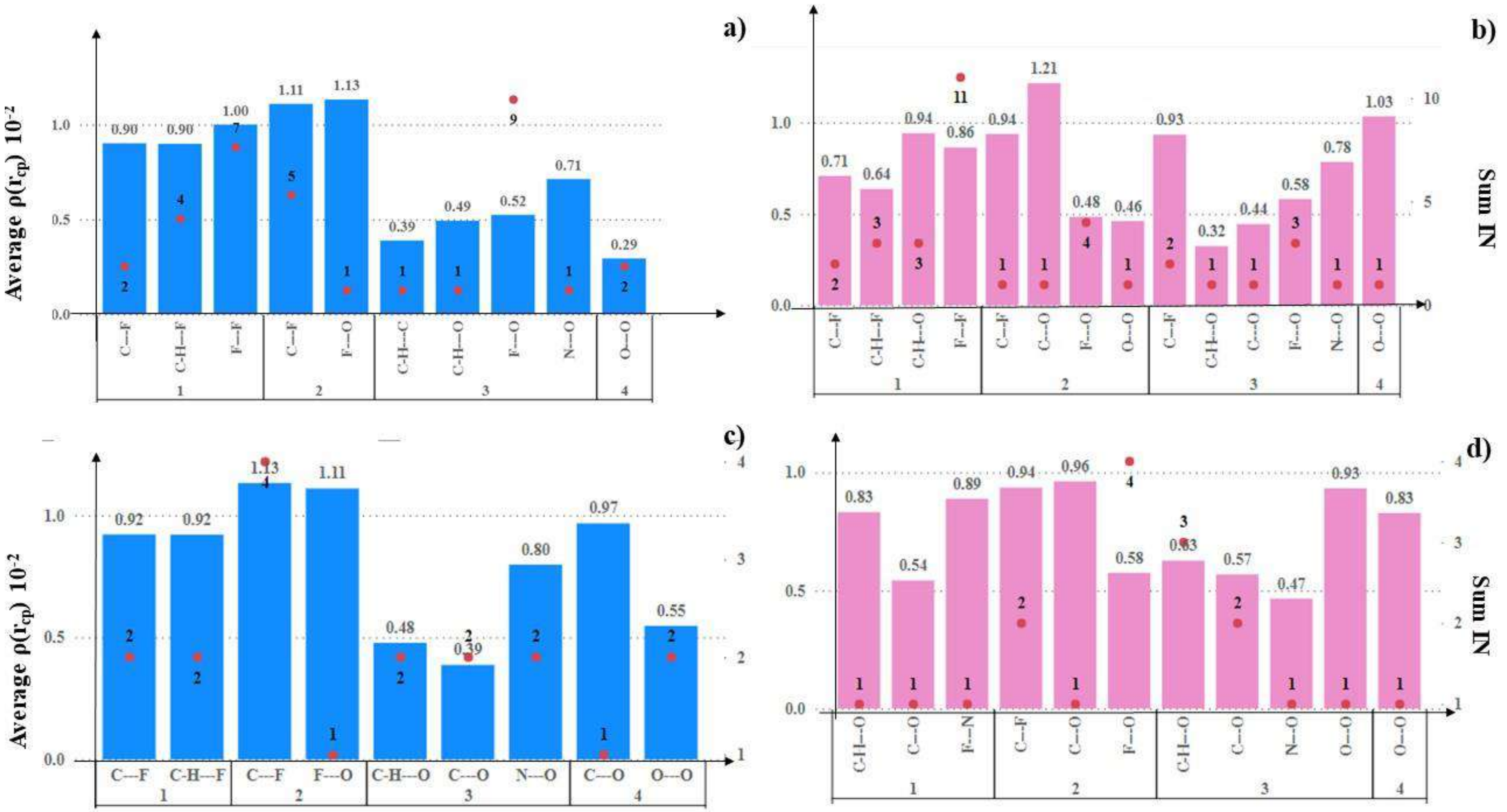

Figure S20. Average values of electron density ρ(rcp) and number of interactions (IN) for the different types of interactions: 1 = Anion-Cation, 2 = Anion-CO_2, 3 = Cation-CO_2, 4 = CO_2-CO_2 per interaction class for 3CO2. a) $[C_8H_4F_{13}mim]^+[BF_4]^-$, b) $[C_8H_4F_{13}mim]^+$ $[TFO]^-$, c) $[Dmim]^+[BF_4]^-$, d) $[Dmim]^+[TFO]^-$ at a level of theory M06-2X/cc-pVTZ (D3, SMD).

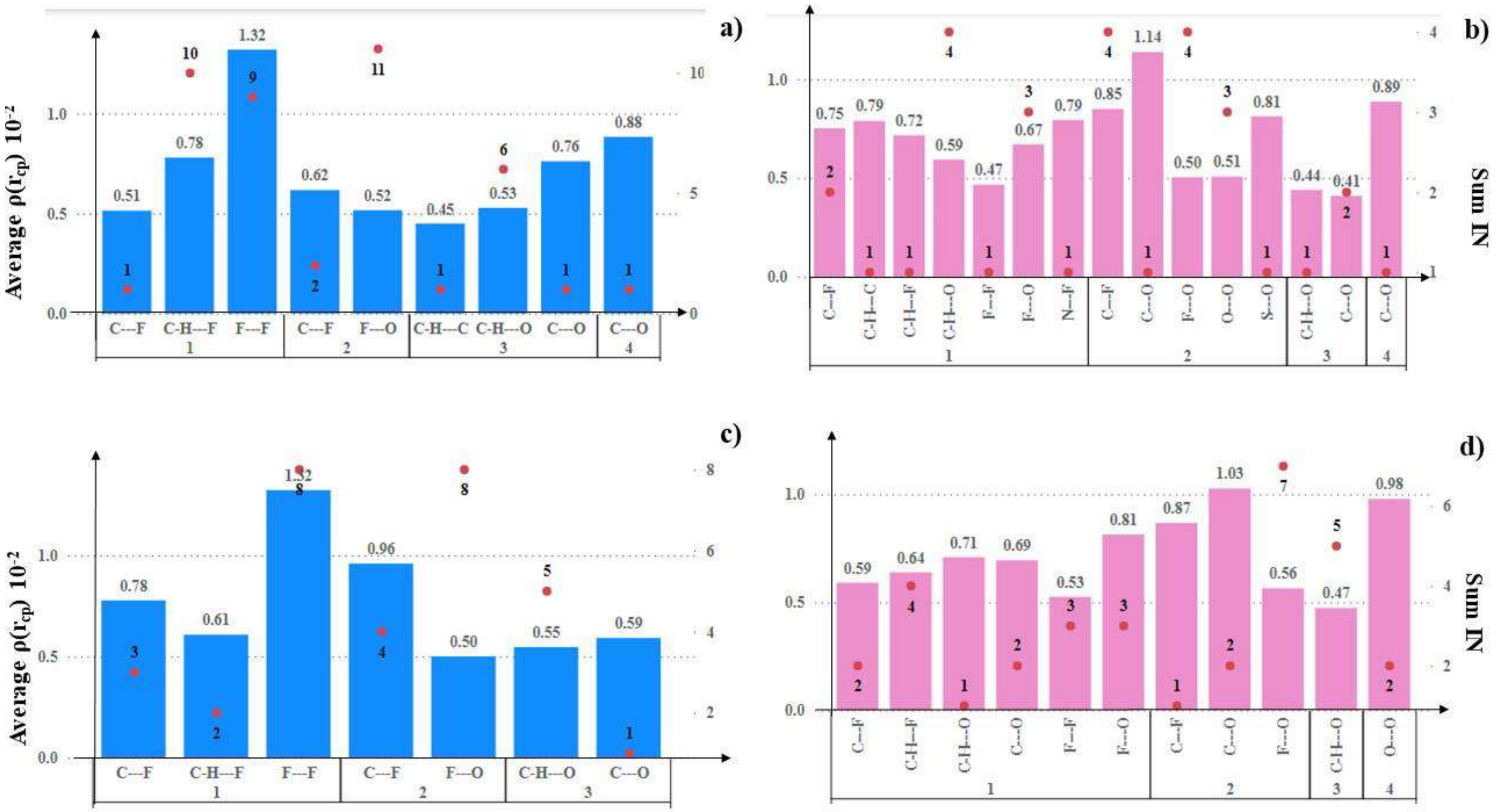

Figure S21. Average values of electron density ρ(rcp) and number of interactions (IN) for the different types of interactions: 1 = Anion-Cation, 2 = Anion-CO_2, 3 = Cation-CO_2, 4 = CO_2-CO_2 per interaction class for 3CO2. a) $[Dbim]^+[FAP]^-$, b) $[Dbim]^+[Methide]^-$, c) $[Hmim]^+[FAP]^-$, d) $[Hmim]^+[Methide]^-$ at a level of theory M06-2X/cc-pVTZ (D3, SMD).

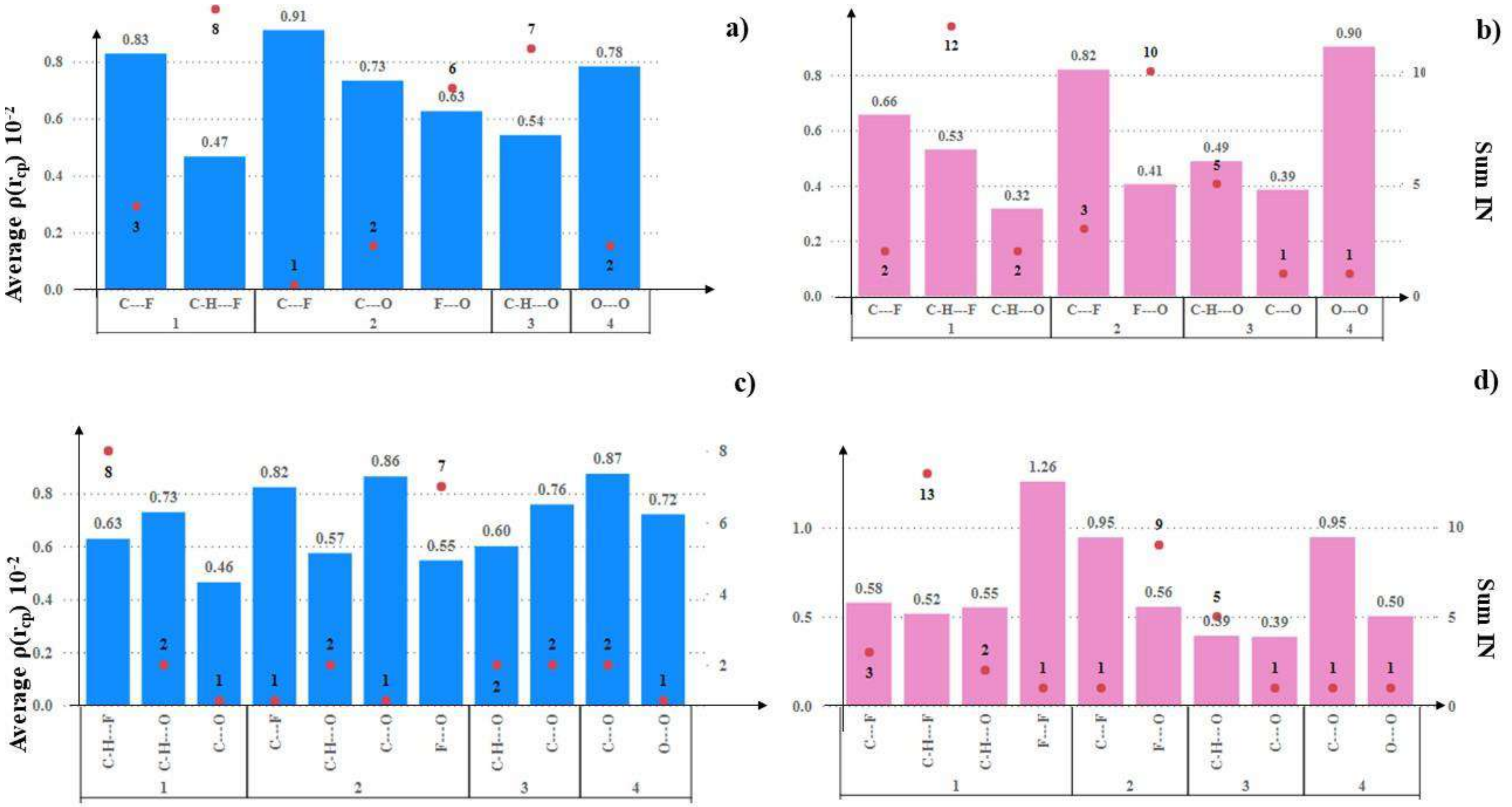

Figure S22. Average values of electron density ρ(rcp) and number of interactions (IN) for the different types of interactions: 1 = Anion-Cation, 2 = Anion-CO_2, 3 = Cation-CO_2, 4 = CO_2-CO_2 per interaction class for 3CO2. a) $[Hmim]^+[(PFOc)SO_3]^-$, b) $[Hmim]^+[(PFBu)SO_3]^-$, c) $[Omim]^+[(PFOc)SO_3]^-$, d) $[Omim]^+[(PFBu)SO_3]^-$ at a level of theory M06-2X/cc-pVTZ (D3, SMD).

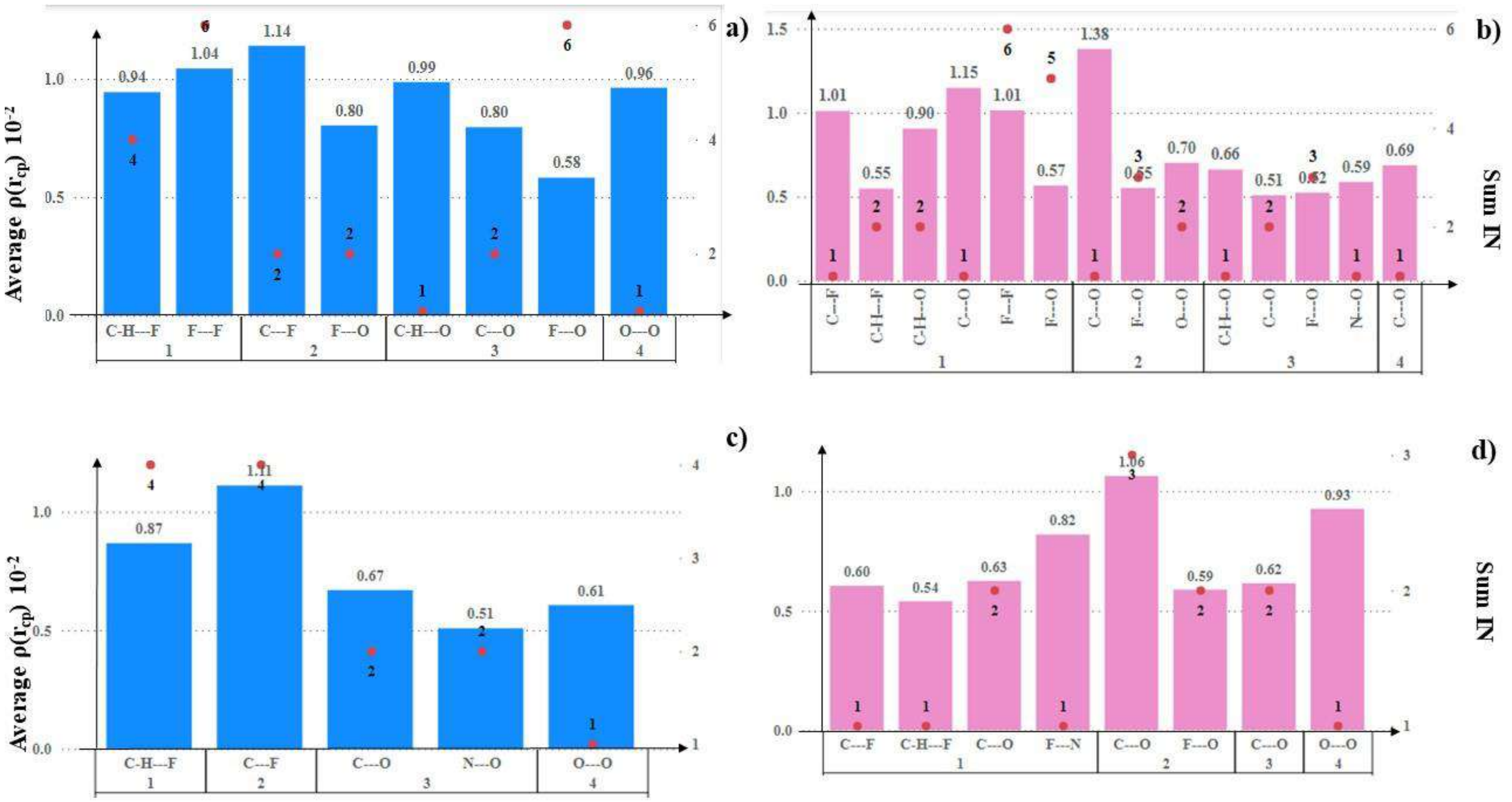

Figure S23. Average values of electron density ρ(rcp) and number of interactions (IN) for the different types of interactions: 1 = Anion-Cation, 2 = Anion-CO_2, 3 = Cation-CO_2, 4 = CO_2-CO_2 per interaction class for 2CO2. a) $[C_8H_4F_{13}mim]^+[BF_4]^-$, b) $[C_8H_4F_{13}mim]^+$ $[TFO]^-$, c) $[Dmim]^+[BF_4]^-$, d) $[Dmim]^+[TFO]^-$ at a level of theory M06-2X/cc-pVTZ (D3, SMD).

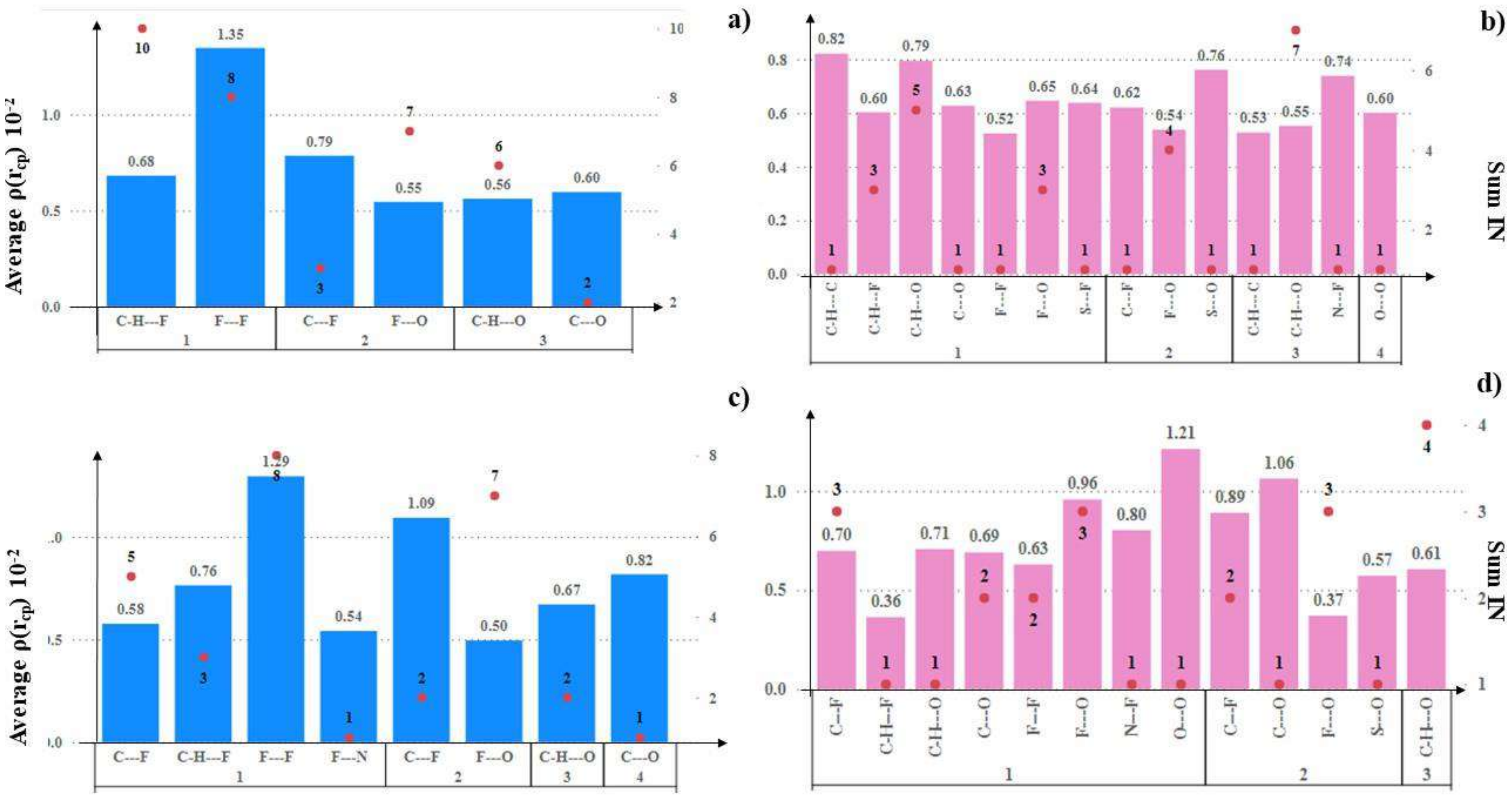

Figure S24. Average values of electron density ρ(rcp) and number of interactions (IN) for the different types of interactions: 1 = Anion-Cation, 2 = Anion-CO_2, 3 = Cation-CO_2, 4 = CO_2-CO_2 per interaction class for 2CO2. a) $[Dbim]^+[FAP]^-$, b) $[Dbim]^+[Methide]^-$, c) $[Hmim]^+[FAP]^-$, d) $[Hmim]^+[Methide]^-$ at a level of theory M06-2X/cc-pVTZ (D3, SMD).

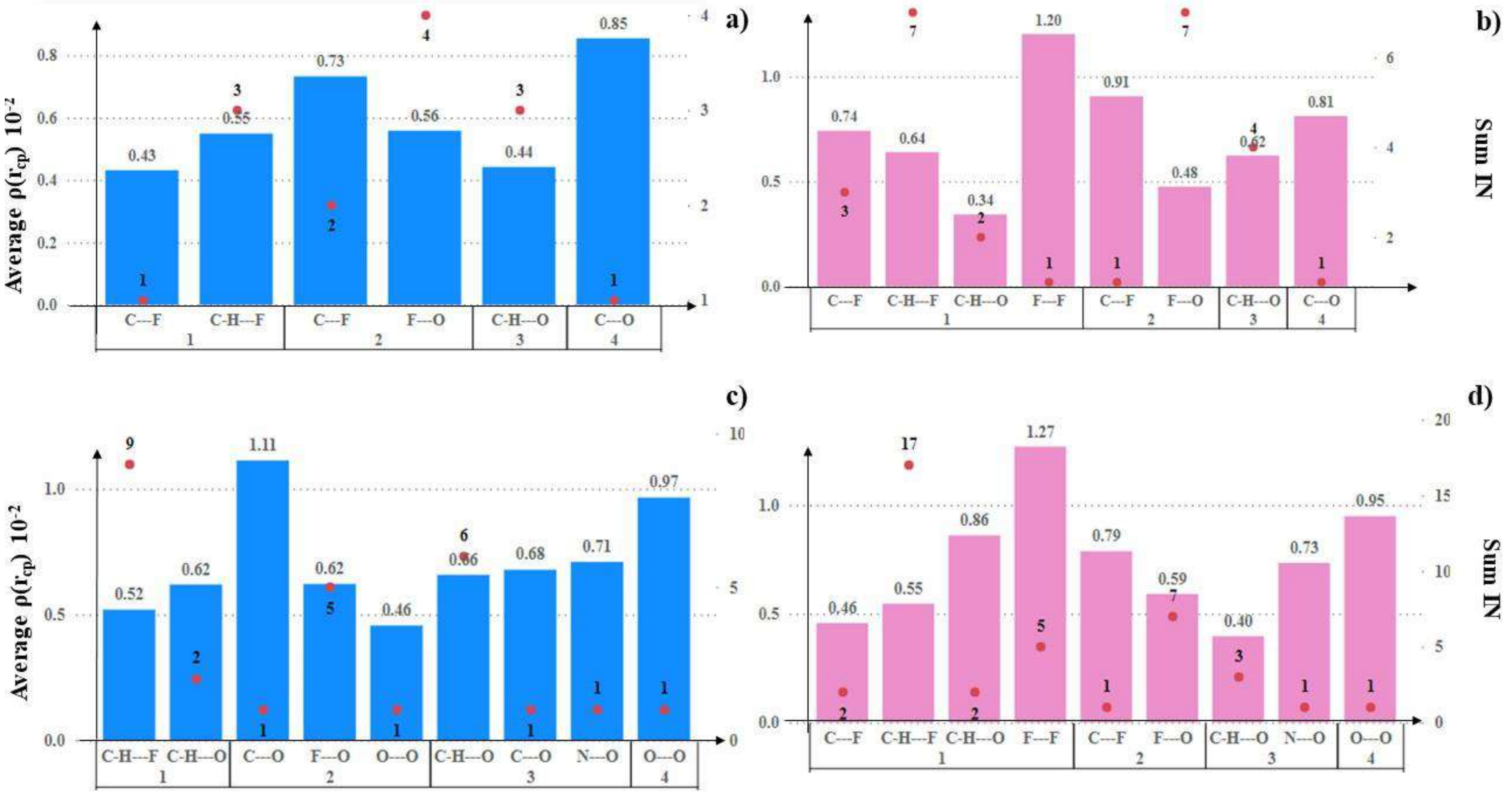

Figure S25. Average values of electron density ρ(rcp) and number of interactions (IN) for the different types of interactions: 1 = Anion-Cation, 2 = Anion-CO_2, 3 = Cation-CO_2, 4 = CO_2-CO_2 per interaction class for 2CO2. a) $[Hmim]^+[(PFOc)SO_3]^-$, b) $[Hmim]^+[(PFBu)SO_3]^-$, c) $[Omim]^+[(PFOc)SO_3]^-$, d) $[Omim]^+[(PFBu)SO_3]^-$ at a level of theory M06-2X/cc-pVTZ (D3, SMD).

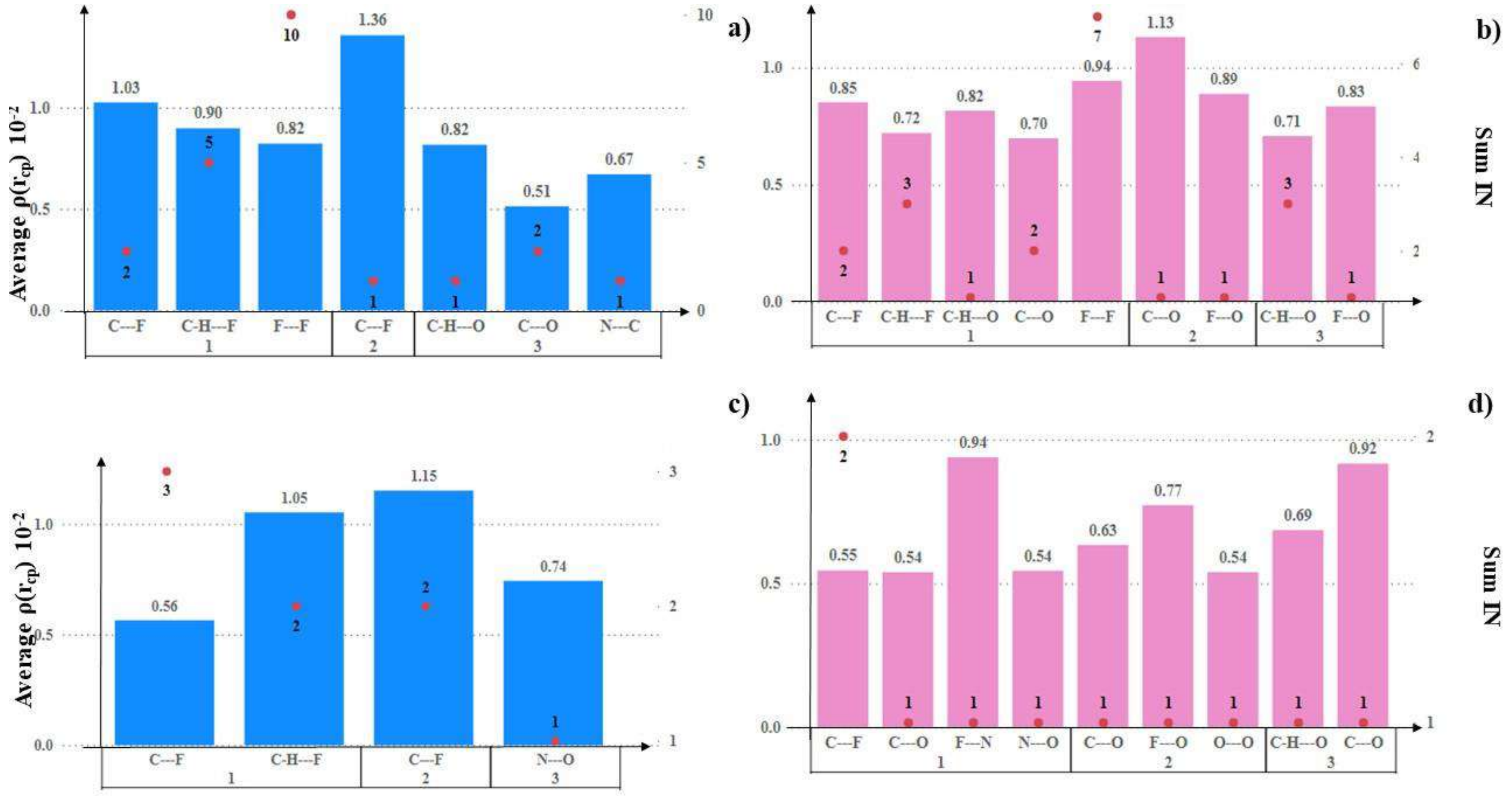

Figure S26. Average values of electron density ρ(rcp) and number of interactions (IN) for the different types of interactions: 1 = Anion-Cation, 2 = Anion-CO_2, 3 = Cation-CO_2, 4 = CO_2-CO_2 per interaction class for 1CO2. a) $[C_8H_4F_{13}mim]^+[BF_4]^-$, b) $[C_8H_4F_{13}mim]^+$ $[TFO]^-$, c) $[Dmim]^+[BF_4]^-$, d) $[Dmim]^+[TFO]^-$ at a level of theory M06-2X/cc-pVTZ (D3, SMD).

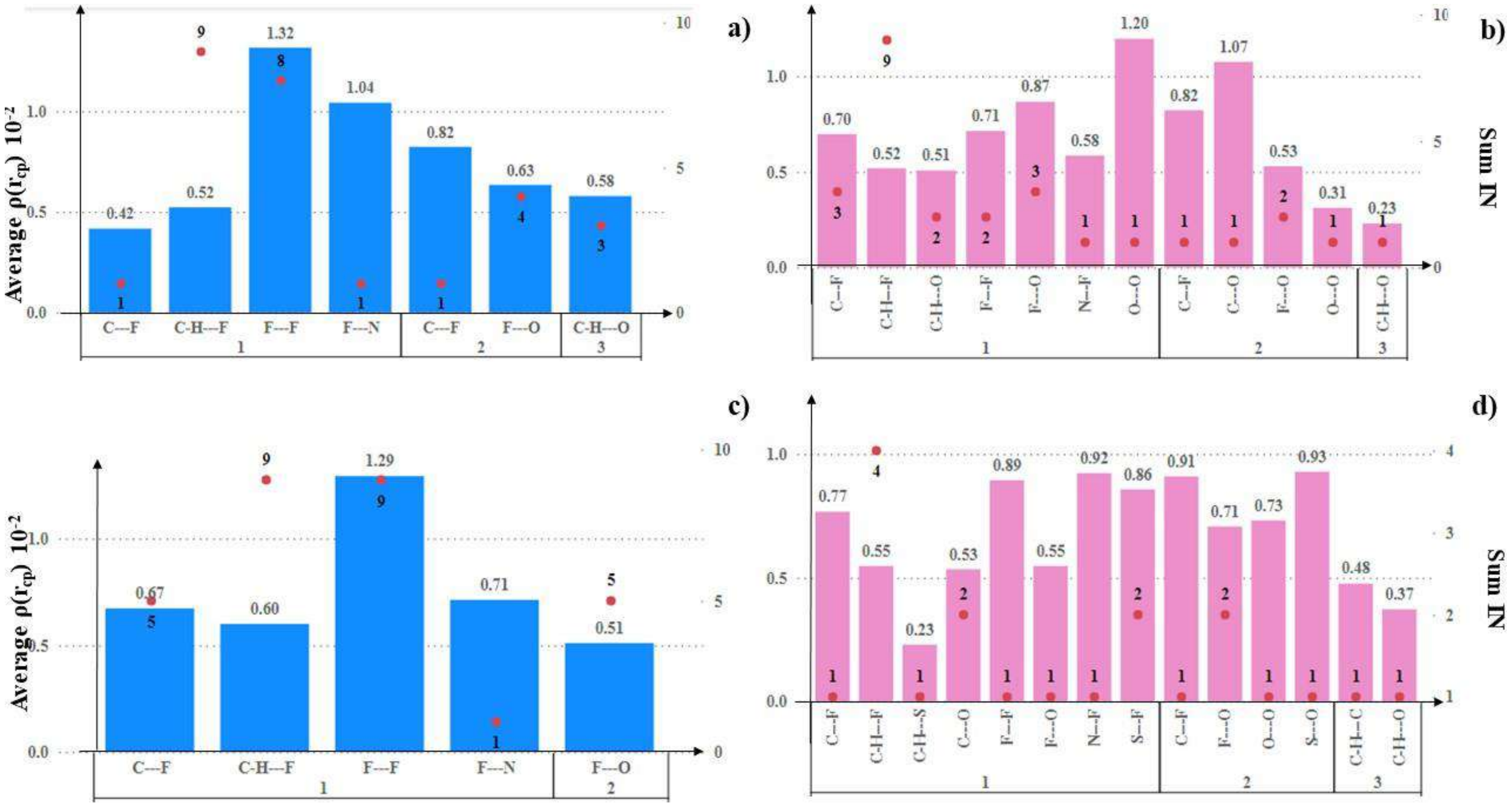

Figure S27. Average values of electron density ρ(rcp) and number of interactions (IN) for the different types of interactions: 1 = Anion-Cation, 2 = Anion-CO_2, 3 = Cation-CO_2, 4 = CO_2-CO_2 per interaction class for 1CO2. a) [Dbim]$^+$[FAP]$^-$, b) [Dbim]$^+$[Methide]$^-$, c) [Hmim]$^+$[FAP]$^-$, d) [Hmim]$^+$[Methide]$^-$ at a level of theory M06-2X/cc-pVTZ (D3, SMD).

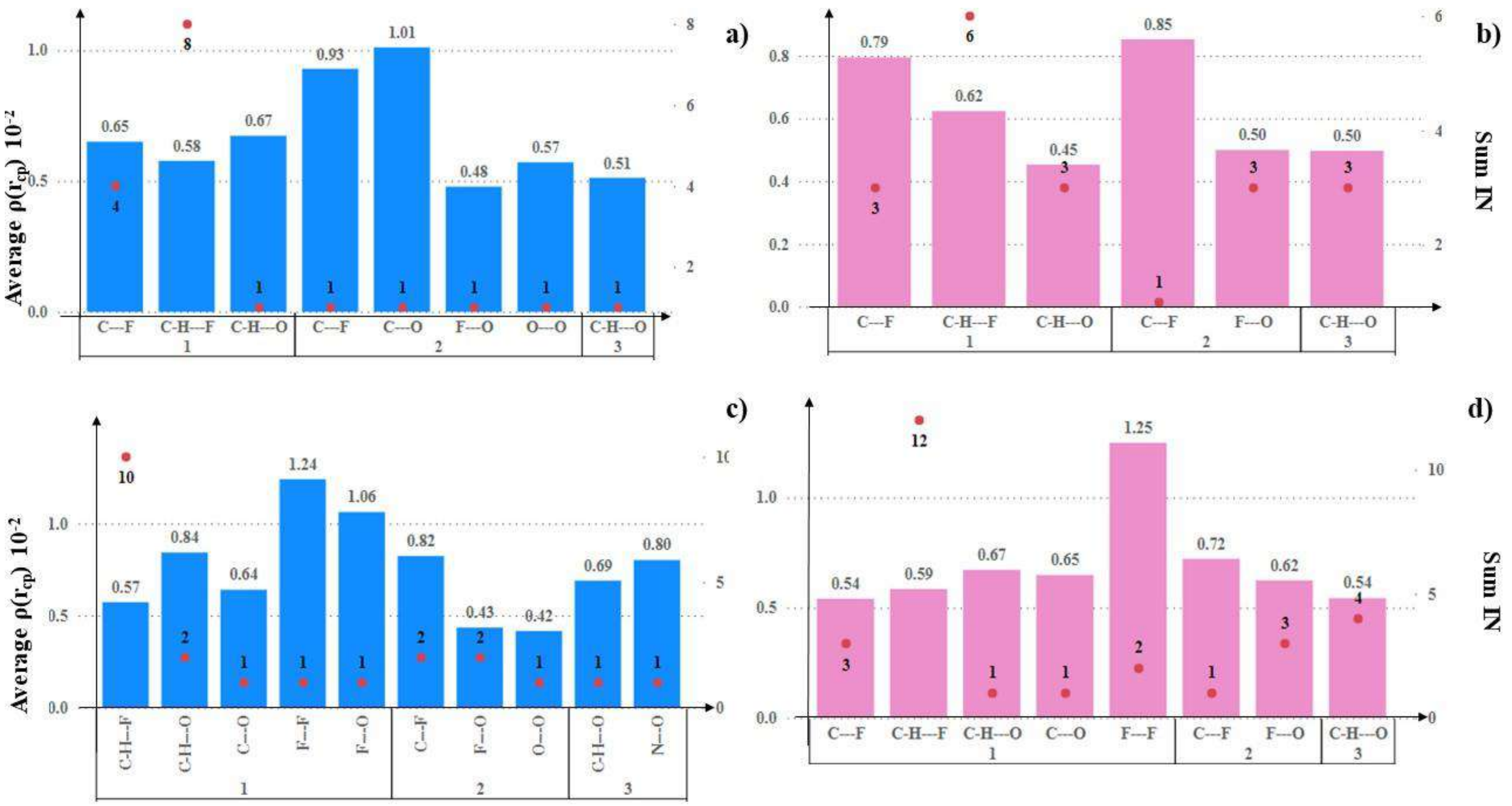

Figure S28. Average values of electron density ρ(rcp) and number of interactions (IN) for the different types of interactions: 1 = Anion-Cation, 2 = Anion-CO_2, 3 = Cation-CO_2, 4 = CO_2-CO_2 per interaction class for 1CO2. a) $[Hmim]^+[(PFOc)SO_3]^-$, b) $[Hmim]^+[(PFBu)SO_3]^-$, c) $[Omim]^+[(PFOc)SO_3]^-$, d) $[Omim]^+[(PFBu)SO_3]^-$ at a level of theory M06-2X/cc-pVTZ (D3, SMD).

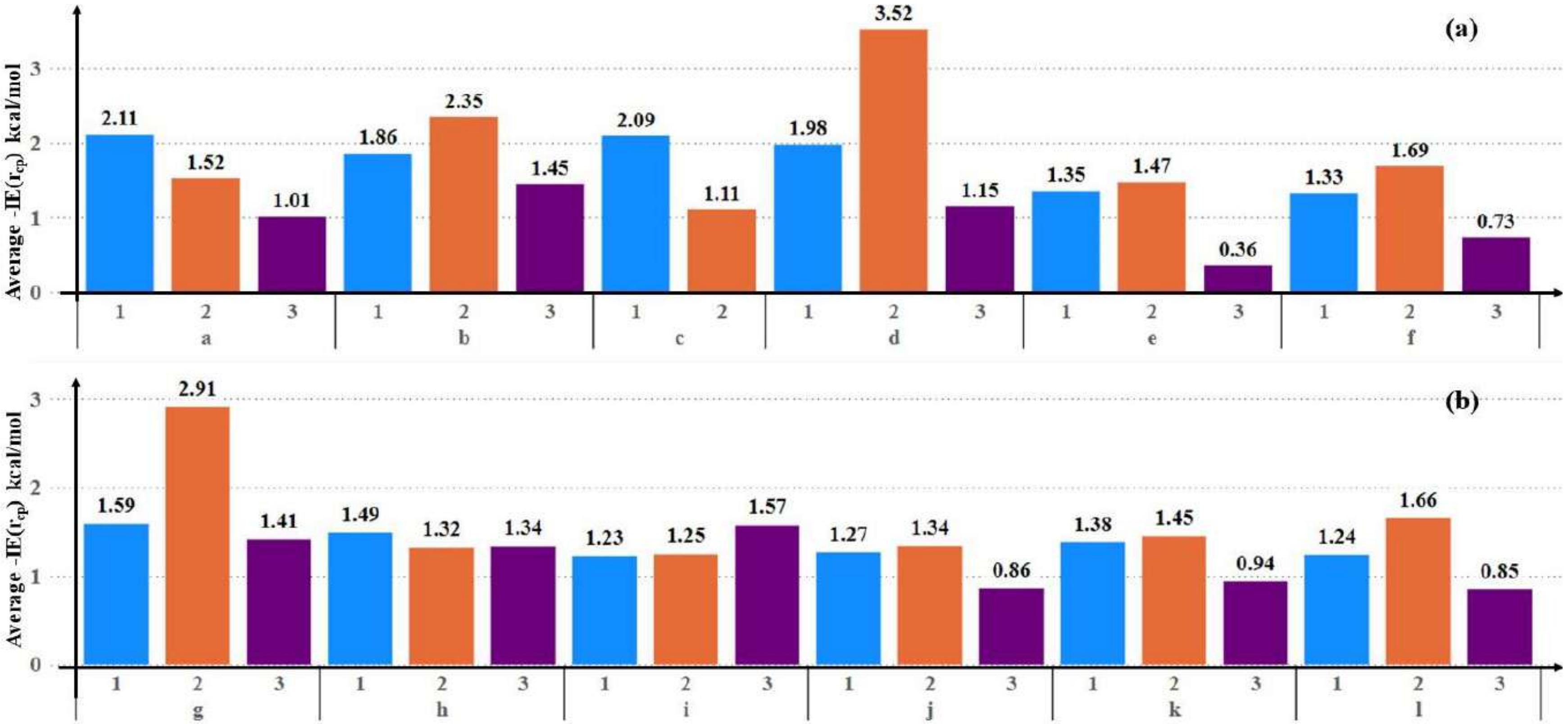

Figure S29. Interaction Energy values -IE(rcp) for the different types of interactions: 1 = Anion-Cation, 2 = Anion-CO_2, 3 = Cation-CO_2, 4 = CO_2-CO_2 for the clusters with 1CO2. (a) Values for the clusters formed with ILs a to f. a: $[Dbim]^+[FAP]^-$, b: $[C_8H_4F_{13}mim]^+[TFO]^-$, c: $[Hmim]^+[FAP]^-$, d: $[C_8H_4F_{13}mim]^+[BF_4]^-$, e: $[Dbim]^+[Methide]^-$, f: $[Hmim]^+[Methide]^-$. (b) Values for clusters formed with ILs g to l. g: $[Dmim]^+[BF_4]^-$, h: $[Omim]^+[(PFBu)SO_3]^-$, i: $[Dmim]^+[TFO]^-$, j: $[Hmim]^+[(PFOc)SO_3]^-$, k: $[Omim]^+[(PFOc)SO_3]^-$, l: $[Hmim]^+[(PFBu)SO_3]^-$.

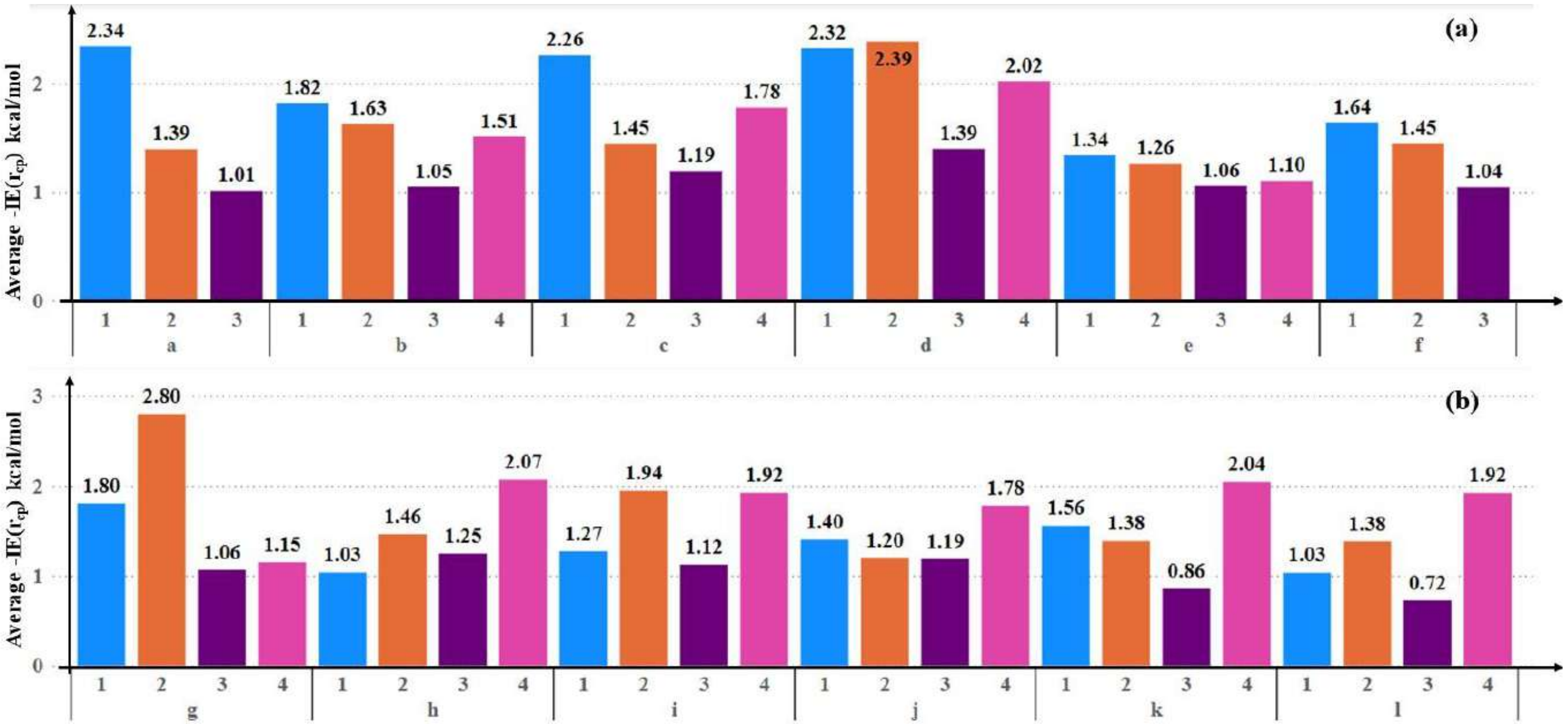

Figure S30. Interaction Energy values -IE(rcp) for the different types of interactions: 1 = Anion-Cation, 2 = Anion-CO_2, 3 = Cation-CO_2, 4 = CO_2-CO_2 for the clusters with 2CO2. (a) Values for the clusters formed with ILs a to f. a: $[Dbim]^+[FAP]^-$, b: $[C_8H_4F_{13}mim]^+[TFO]^-$, c: $[Hmim]^+[FAP]^-$, d: $[C_8H_4F_{13}mim]^+[BF_4]^-$, e: $[Dbim]^+[Methide]^-$, f: $[Hmim]^+[Methide]^-$. (b) Values for clusters formed with ILs g to l. g: $[Dmim]^+[BF_4]^-$, h: $[Omim]^+[(PFBu)SO_3]^-$, i: $[Dmim]^+[TFO]^-$, j: $[Hmim]^+[(PFOc)SO_3]^-$, k: $[Omim]^+[(PFOc)SO_3]^-$, l: $[Hmim]^+[(PFBu)SO_3]^-$.

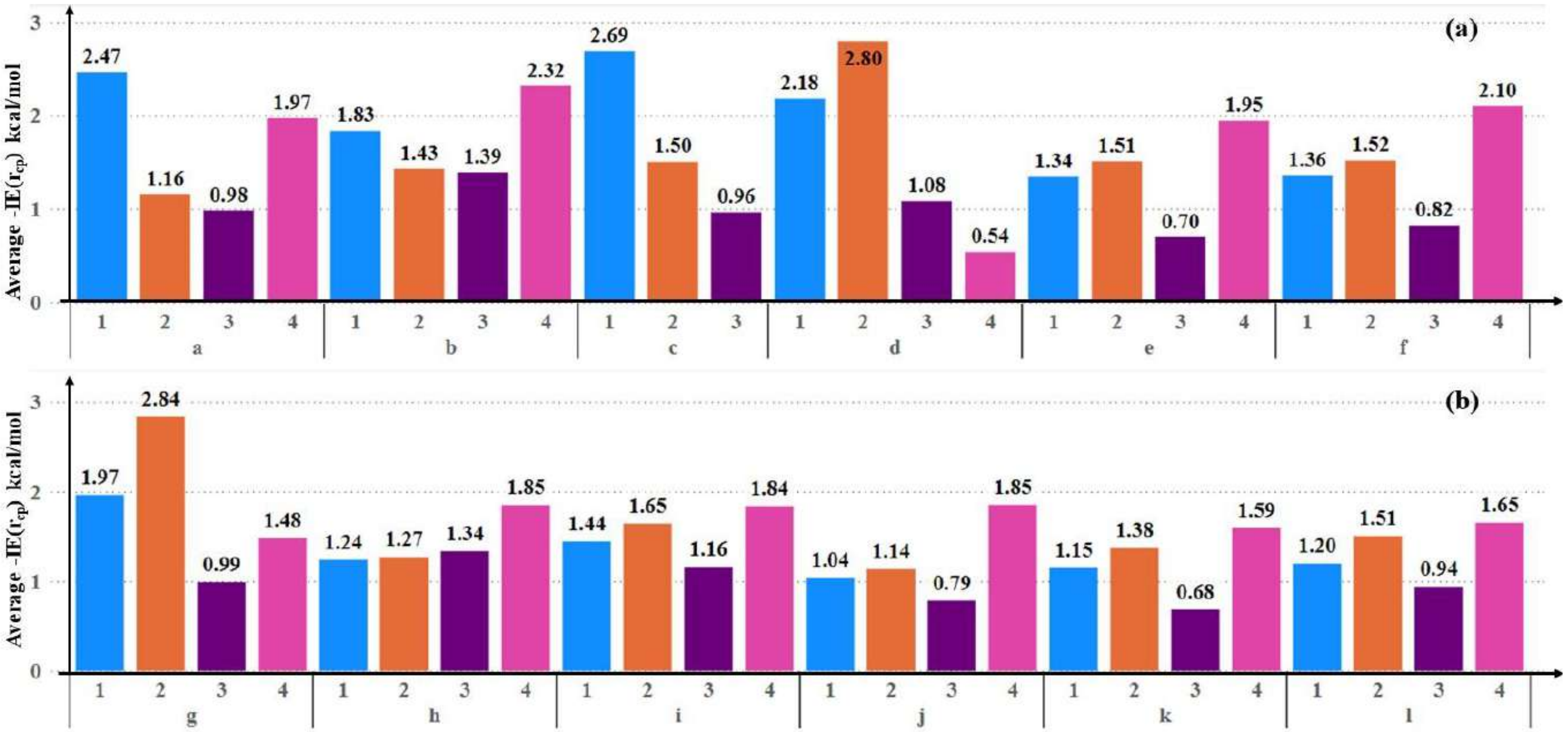

Figure S31. Interaction Energy values -IE(rcp) for the different types of interactions: 1 = Anion-Cation, 2 = Anion-CO_2, 3 = Cation-CO_2, 4 = CO_2-CO_2 for the clusters with 3CO2. (a) Values for the clusters formed with ILs a to f. a: $[Dbim]^+[FAP]^-$, b: $[C_8H_4F_{13}mim]^+[TFO]^-$, c: $[Hmim]^+[FAP]^-$, d: $[C_8H_4F_{13}mim]^+[BF_4]^-$, e: $[Dbim]^+[Methide]^-$, f: $[Hmim]^+[Methide]^-$. (b) Values for clusters formed with ILs g to l. g: $[Dmim]^+[BF_4]^-$, h: $[Omim]^+[(PFBu)SO_3]^-$, i: $[Dmim]^+[TFO]^-$, j: $[Hmim]^+[(PFOc)SO_3]^-$, k: $[Omim]^+[(PFOc)SO_3]^-$, l: $[Hmim]^+[(PFBu)SO_3]^-$.

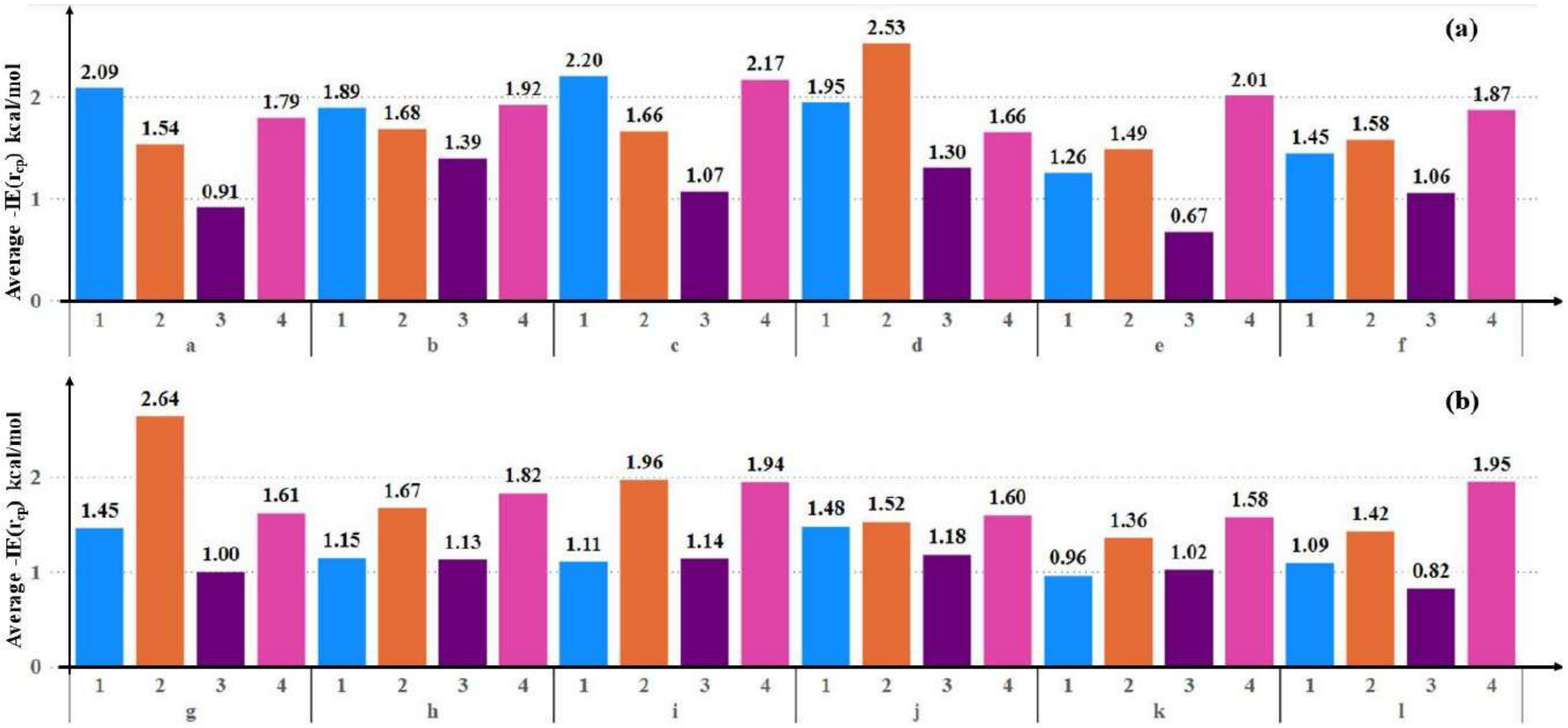

Figure S32. Interaction Energy values -IE(rcp) for the different types of interactions: 1 = Anion-Cation, 2 = Anion-CO_2, 3 = Cation-CO_2, 4 = CO_2-CO_2 for the clusters with 4CO2. (a) Values for the clusters formed with ILs a to f. a: $[Dbim]^+[FAP]^-$, b: $[C_8H_4F_{13}mim]^+[TFO]^-$, c: $[Hmim]^+[FAP]^-$, d: $[C_8H_4F_{13}mim]^+[BF_4]^-$, e: $[Dbim]^+[Methide]^-$, f: $[Hmim]^+[Methide]^-$. (b) Values for clusters formed with ILs g to l. g: $[Dmim]^+[BF_4]^-$, h: $[Omim]^+[(PFBu)SO_3]^-$, i: $[Dmim]^+[TFO]^-$, j: $[Hmim]^+[(PFOc)SO_3]^-$, k: $[Omim]^+[(PFOc)SO_3]^-$, l: $[Hmim]^+[(PFBu)SO_3]^-$.

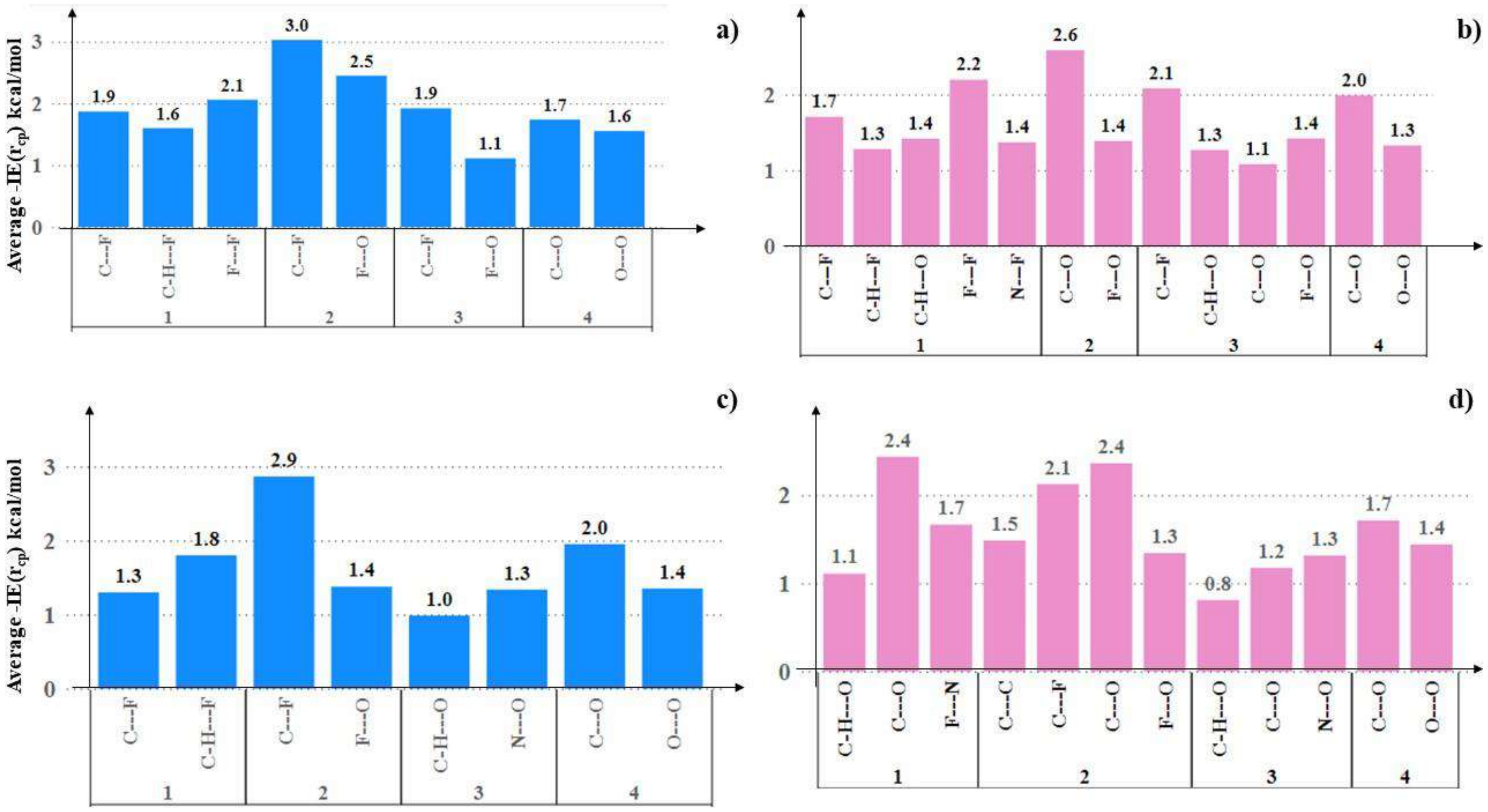

Figure S33. Average values of Interaction Energy values -IE(rcp) for the different types of interactions: 1 = Anion-Cation, 2 = Anion-CO_2, 3 = Cation-CO_2, 4 = CO_2-CO_2 per interaction class for 5CO2. a) $[C_8H_4F_{13}mim]^+[BF_4]^-$, b) $[C_8H_4F_{13}mim]^+[TFO]^-$, c) $[Dmim]^+[BF_4]^-$, d) $[Dmim]^+[TFO]^-$ at a level of theory M06-2X/cc-pVTZ (D3, SMD).

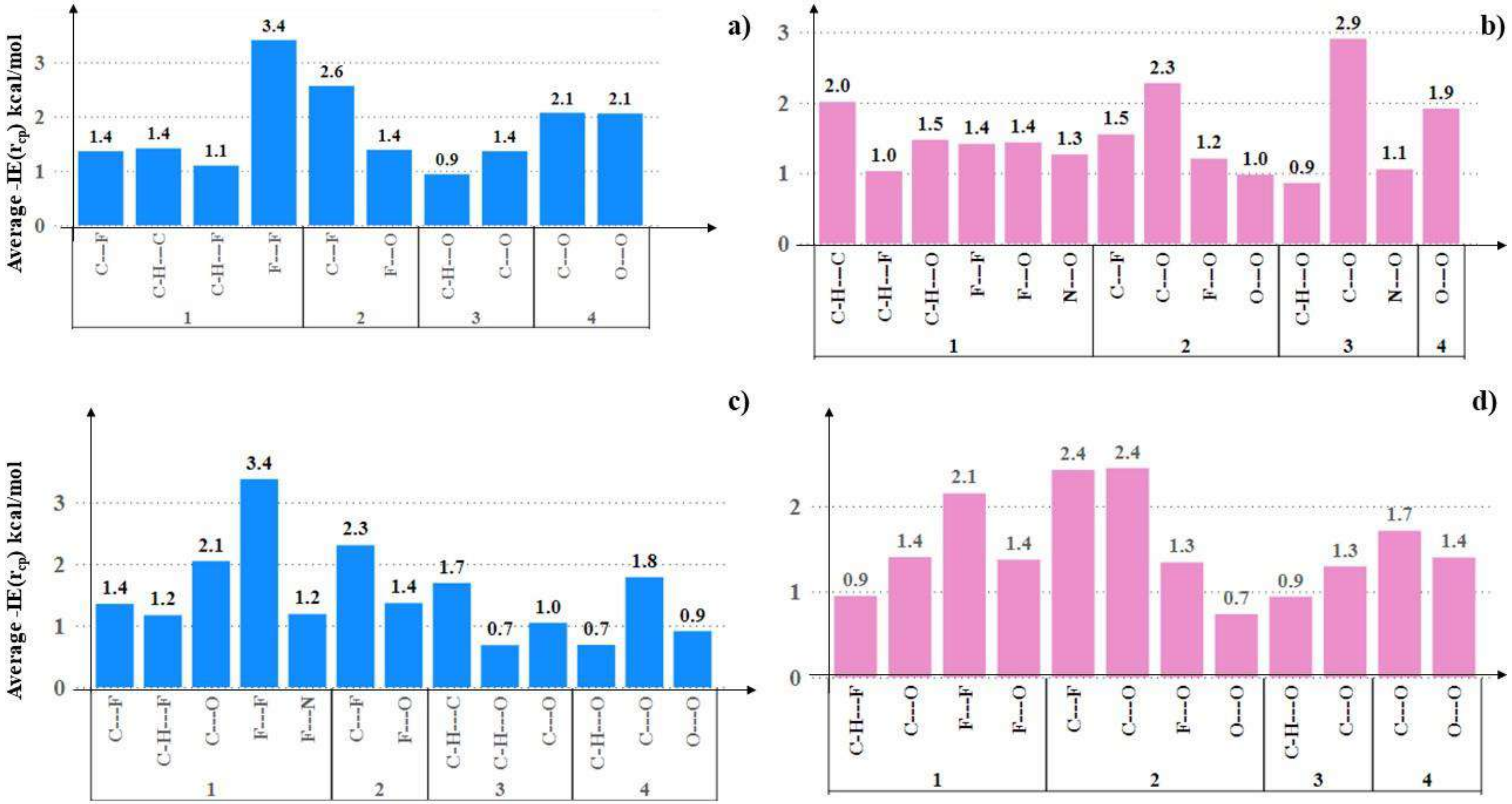

Figure S34. Average values of Interaction Energy values -IE(rcp) for the different types of interactions: 1 = Anion-Cation, 2 = Anion-CO_2, 3 = Cation-CO_2, 4 = CO_2-CO_2 per interaction class for 5CO2. a) $[Dbim]^+[FAP]^-$, b) $[Dbim]^+[Methide]^-$, c) $[Hmim]^+[FAP]^-$, d) $[Hmim]^+[Methide]^-$ at a level of theory M06-2X/cc-pVTZ (D3, SMD).

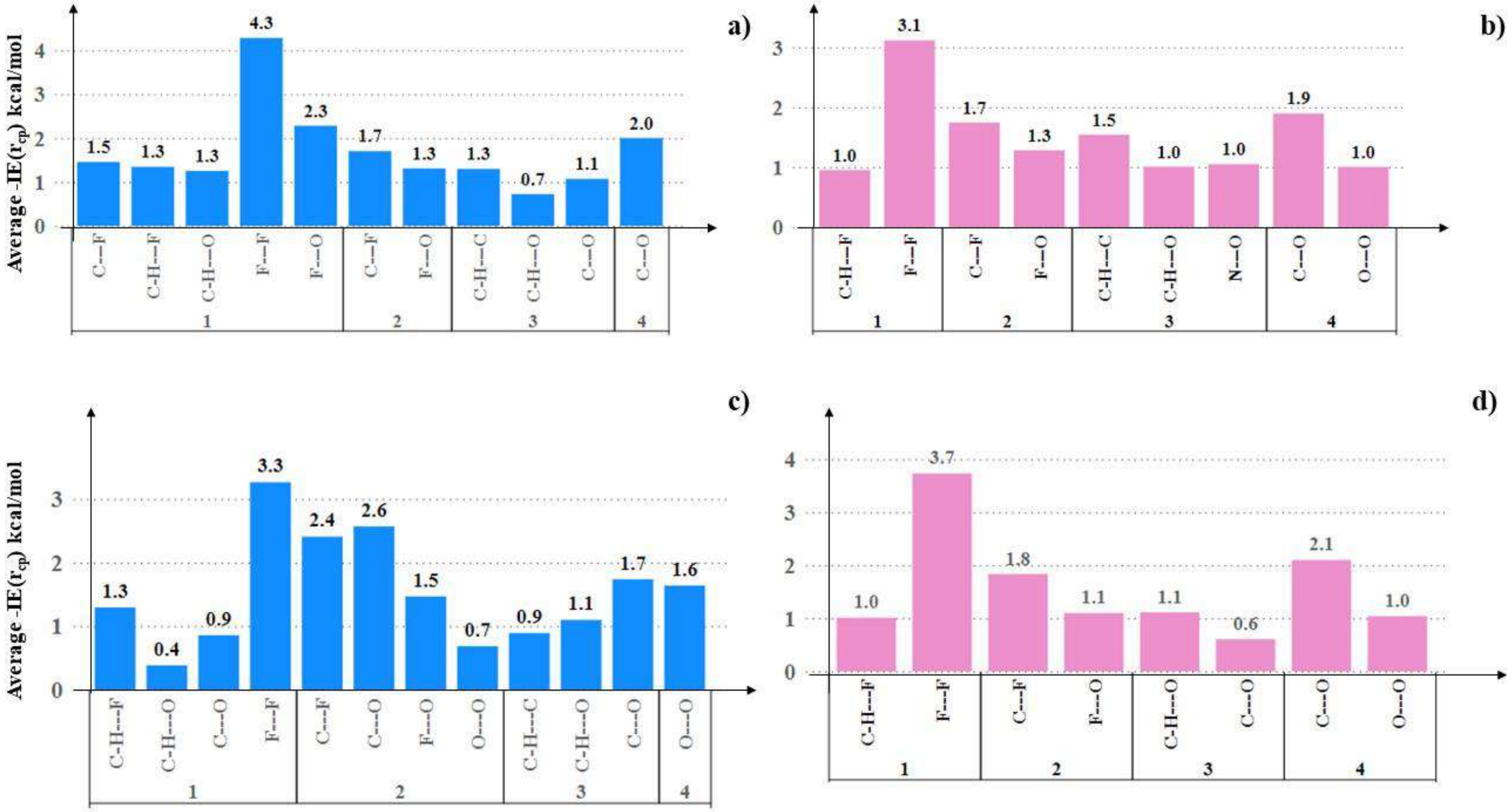

Figure S35. Average values of Interaction Energy values -IE(rcp) for the different types of interactions: 1 = Anion-Cation, 2 = Anion-CO_2, 3 = Cation-CO_2, 4 = CO_2-CO_2 per interaction class for 5CO2. a) $[Hmim]^+[(PFOc)SO_3]^-$, b) $[Hmim]^+[(PFBu)SO_3]^-$, c) $[Omim]^+[(PFOc)SO_3]^-$, d) $[Omim]^+[(PFBu)SO_3]^-$ at a level of theory M06-2X/cc-pVTZ (D3, SMD).

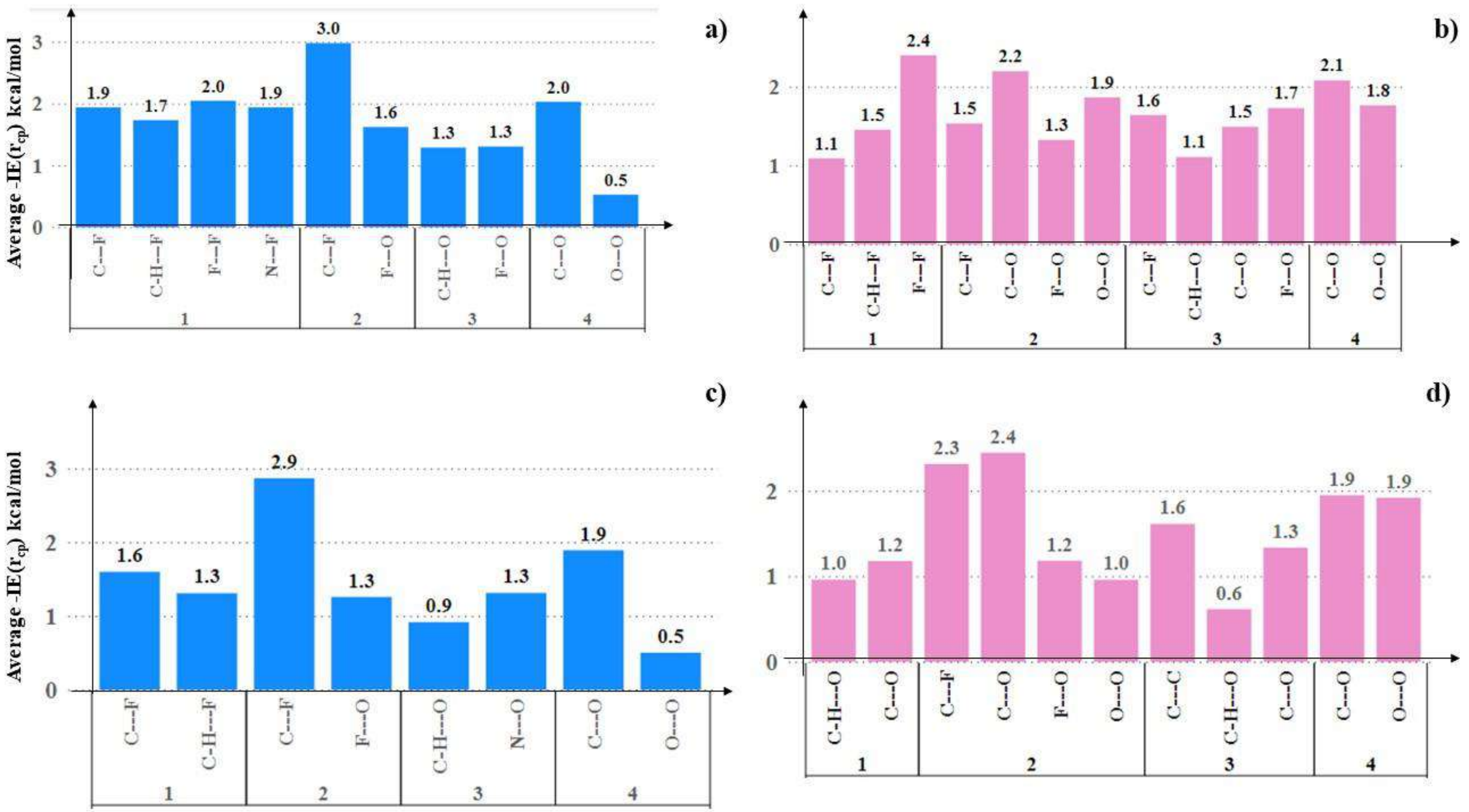

Figure S36. Average values of Interaction Energy values -IE(rcp) for the different types of interactions: 1 = Anion-Cation, 2 = Anion-CO_2, 3 = Cation-CO_2, 4 = CO_2-CO_2 per interaction class for 4CO2. a) $[C_8H_4F_{13}mim]^+[BF_4]^-$, b) $[C_8H_4F_{13}mim]^+[TFO]^-$, c) $[Dmim]^+[BF_4]^-$, d) $[Dmim]^+[TFO]^-$ at a level of theory M06-2X/cc-pVTZ (D3, SMD).

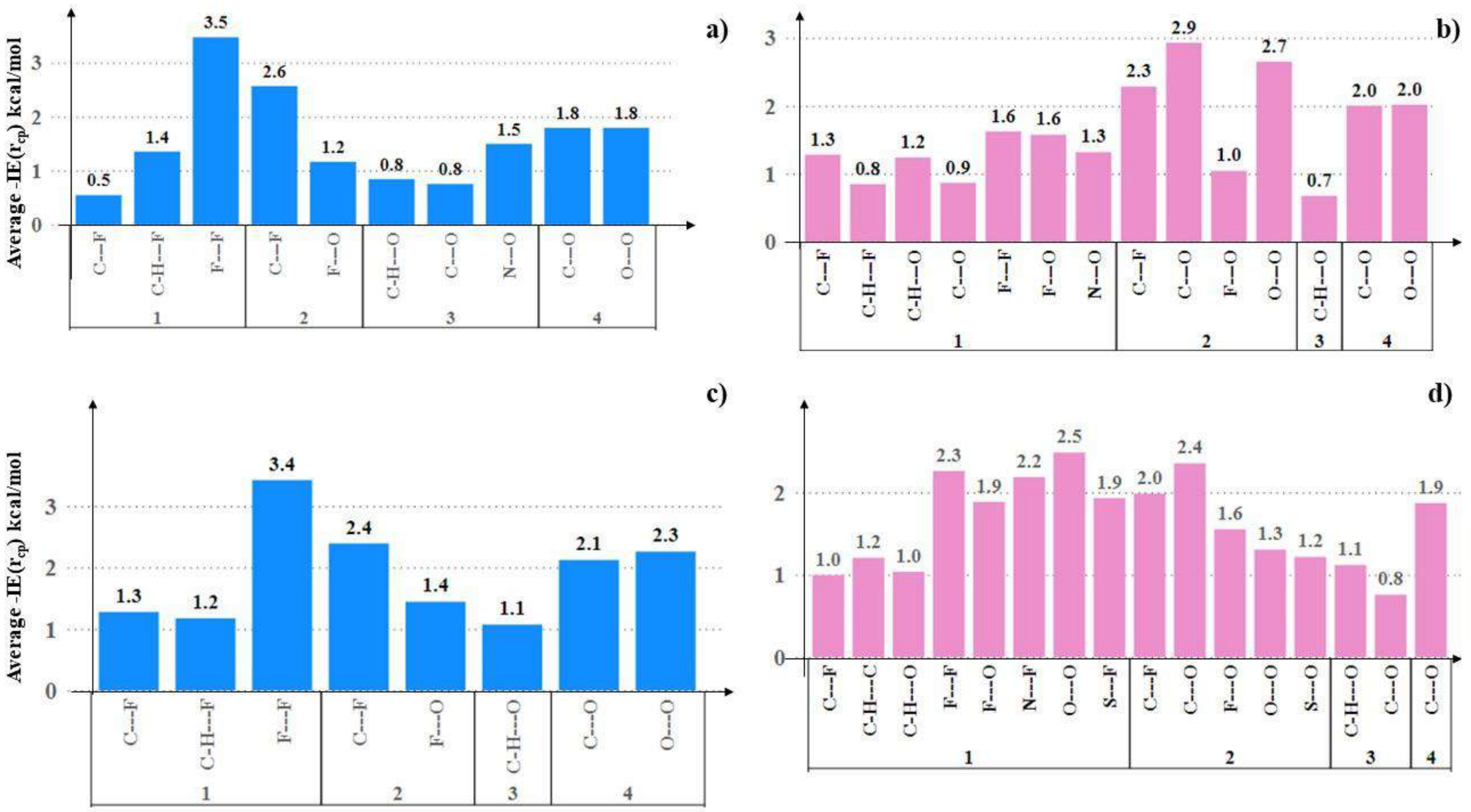

Figure S37. Average values of Interaction Energy values -IE(rcp) for the different types of interactions: 1 = Anion-Cation, 2 = Anion-CO_2, 3 = Cation-CO_2, 4 = CO_2-CO_2 per interaction class for 4CO2. a) [Dbim]$^+$[FAP]$^-$, b) [Dbim]$^+$[Methide]$^-$, c) [Hmim]$^+$[FAP]$^-$, d) [Hmim]$^+$[Methide]$^-$ at a level of theory M06-2X/cc-pVTZ (D3, SMD).

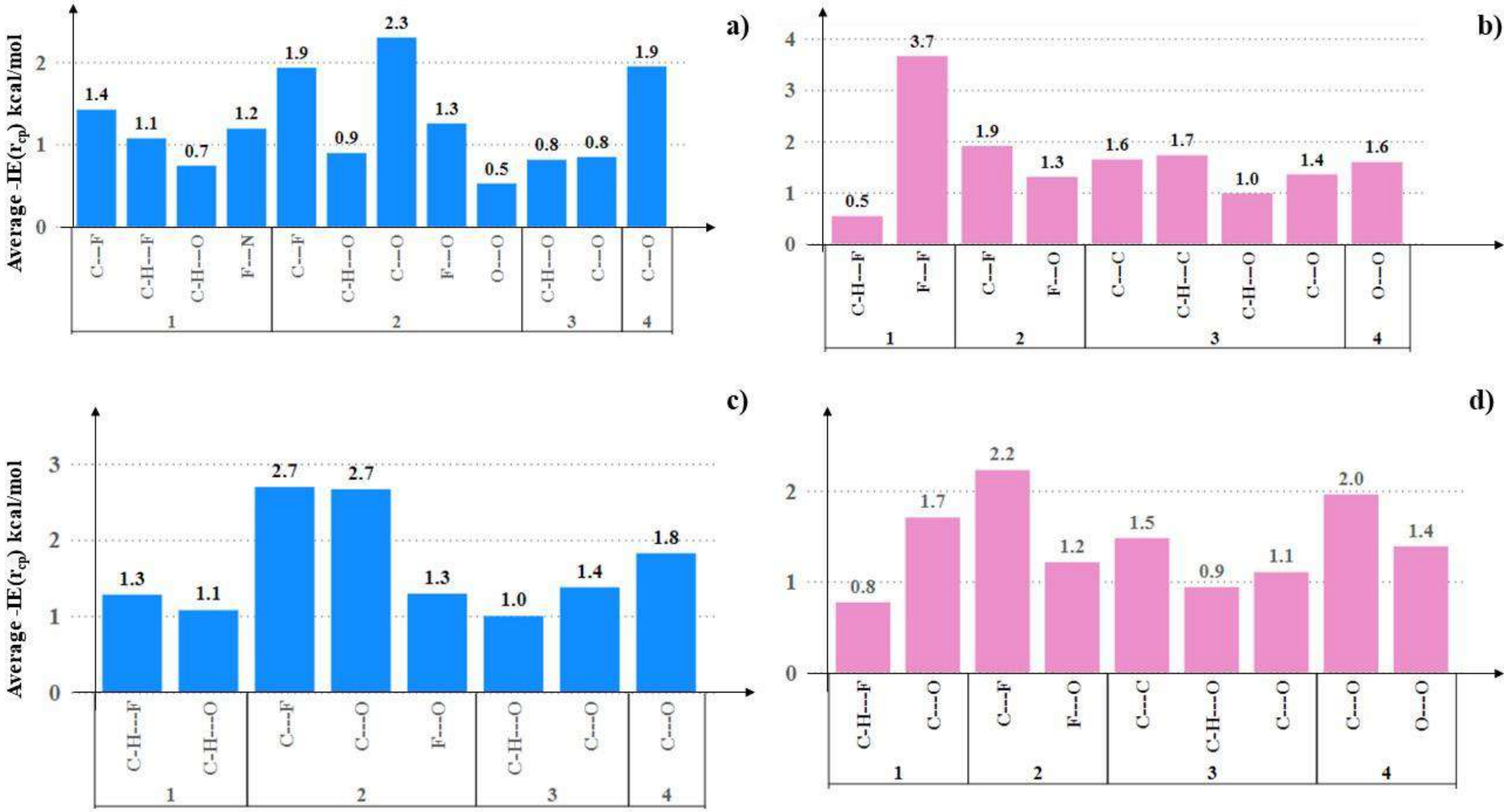

Figure S38. Average values of Interaction Energy values -IE(rcp) for the different types of interactions: 1 = Anion-Cation, 2 = Anion-CO_2, 3 = Cation-CO_2, 4 = CO_2-CO_2 per interaction class for 4CO2. a) $[Hmim]^+[(PFOc)SO_3]^-$, b) $[Hmim]^+[(PFBu)SO_3]^-$, c) $[Omim]^+[(PFOc)SO_3]^-$, d) $[Omim]^+[(PFBu)SO_3]^-$ at a level of theory M06-2X/cc-pVTZ (D3, SMD).

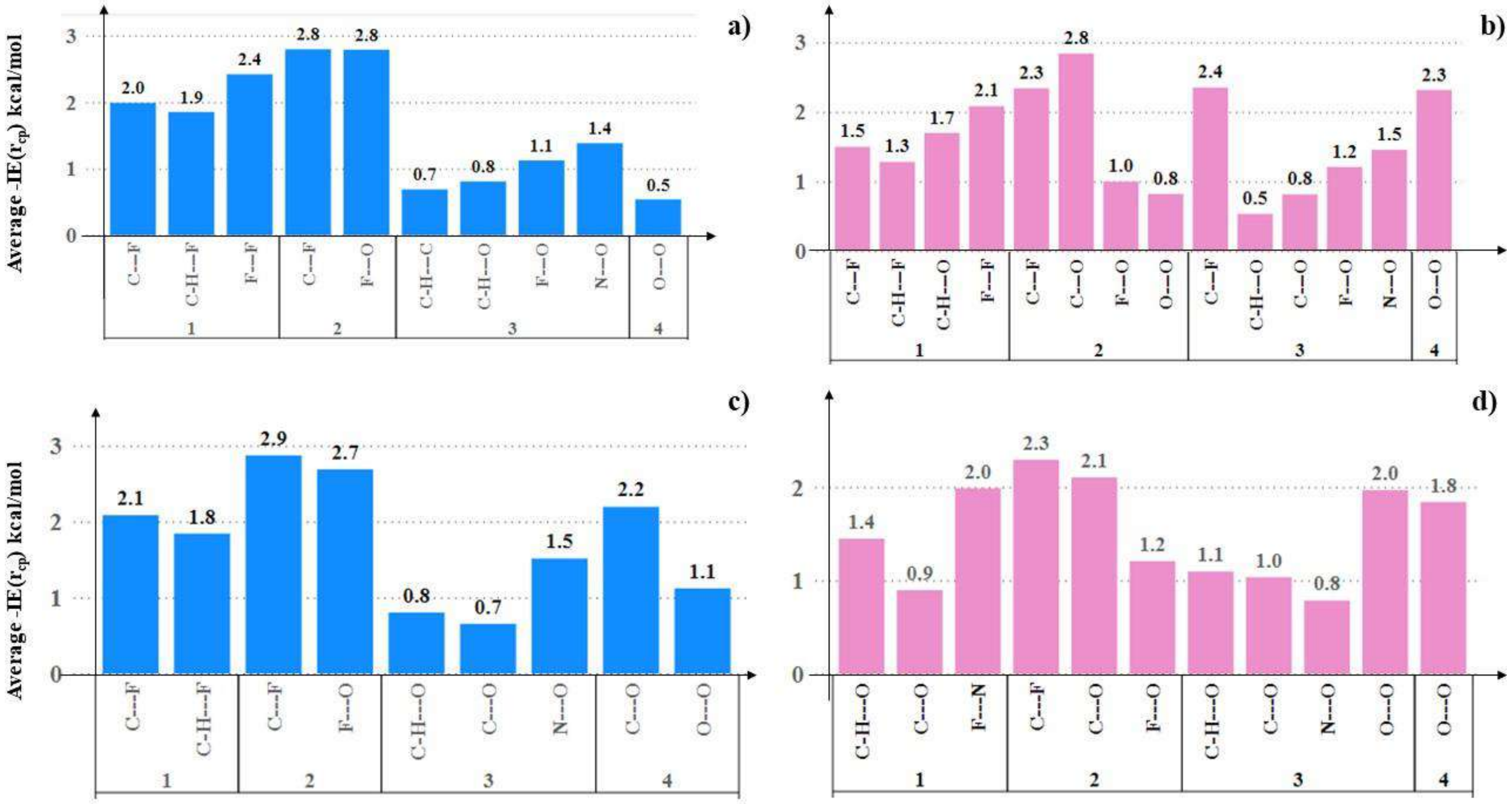

Figure S39. Average values of Interaction Energy values -IE(rcp) for the different types of interactions: 1 = Anion-Cation, 2 = Anion-CO_2, 3 = Cation-CO_2, 4 = CO_2-CO_2 per interaction class for 3CO2. a) $[C_8H_4F_{13}mim]^+[BF_4]^-$, b) $[C_8H_4F_{13}mim]^+[TFO]^-$, c) $[Dmim]^+[BF_4]^-$, d) $[Dmim]^+[TFO]^-$ at a level of theory M06-2X/cc-pVTZ (D3, SMD).

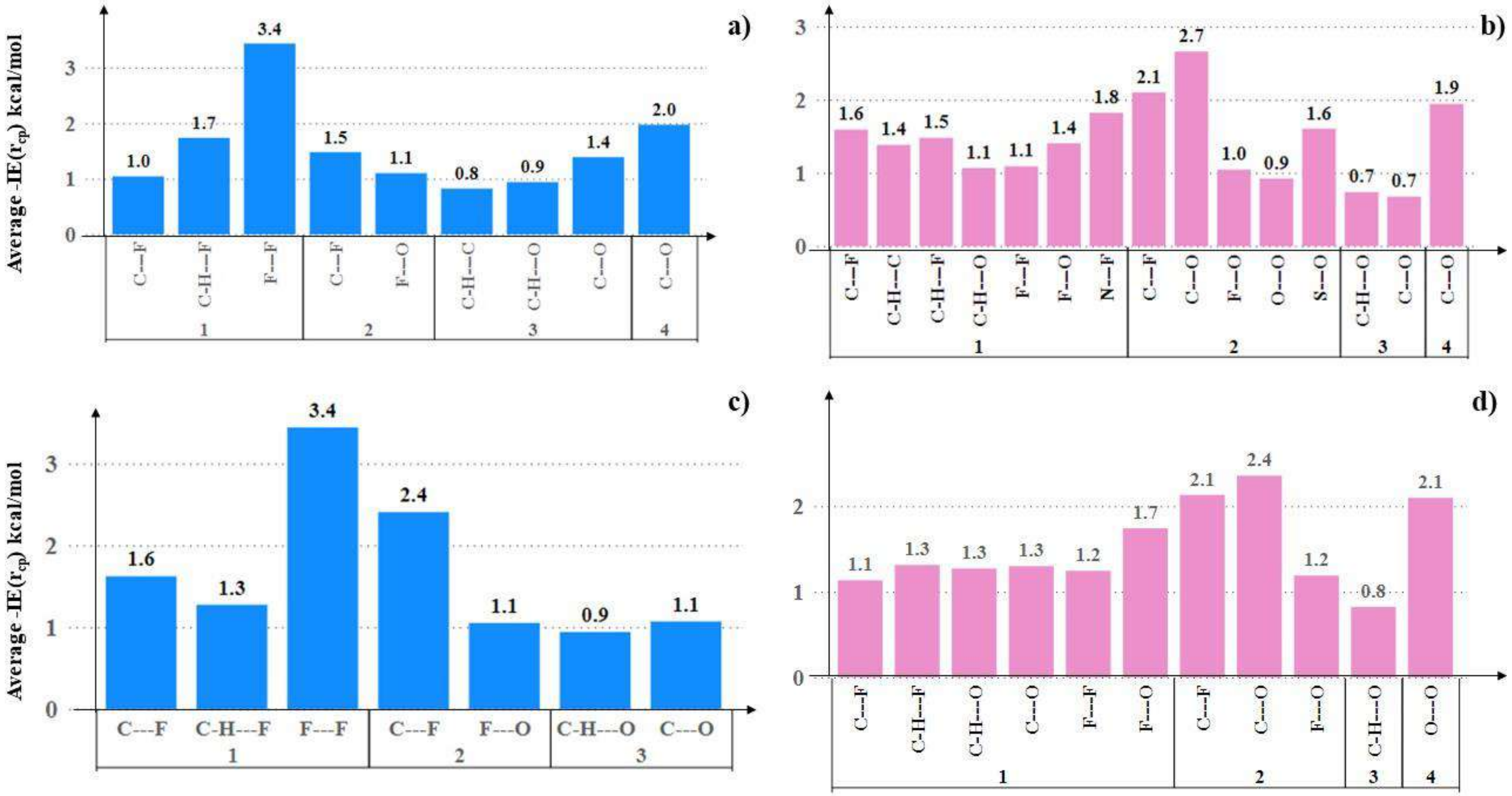

Figure S40. Average values of Interaction Energy values -IE(rcp) for the different types of interactions: 1 = Anion-Cation, 2 = Anion-CO_2, 3 = Cation-CO_2, 4 = CO_2-CO_2 per interaction class for 3CO2. a) $[Dbim]^+[FAP]^-$, b) $[Dbim]^+[Methide]^-$, c) $[Hmim]^+[FAP]^-$, d) $[Hmim]^+[Methide]^-$ at a level of theory M06-2X/cc-pVTZ (D3, SMD).

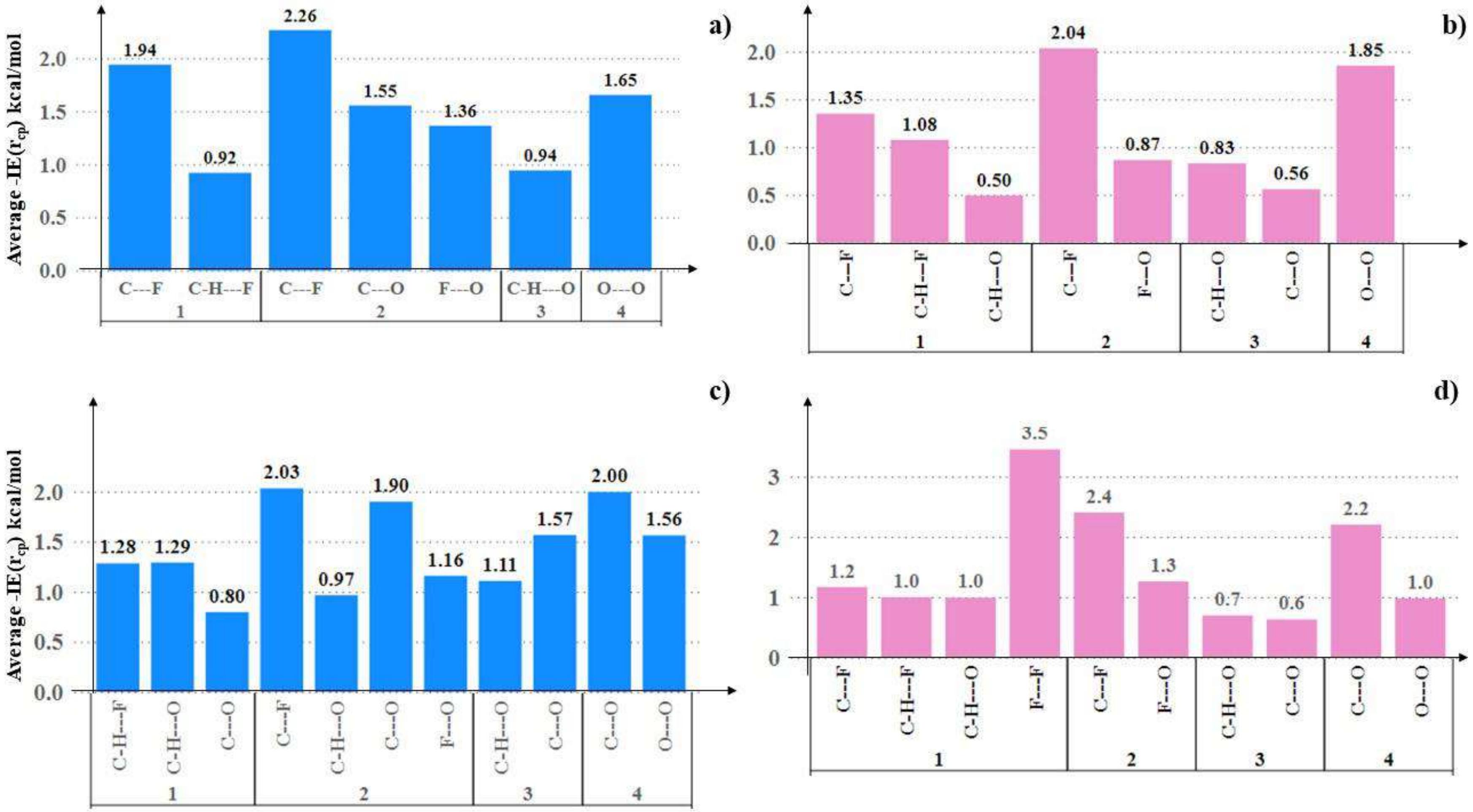

Figure S41. Average values of Interaction Energy values -IE(rcp) for the different types of interactions: 1 = Anion-Cation, 2 = Anion-CO_2, 3 = Cation-CO_2, 4 = CO_2-CO_2 per interaction class for 3CO2. a) $[Hmim]^+[(PFOc)SO_3]^-$, b) $[Hmim]^+[(PFBu)SO_3]^-$, c) $[Omim]^+[(PFOc)SO_3]^-$, d) $[Omim]^+[(PFBu)SO_3]^-$ at a level of theory M06-2X/cc-pVTZ (D3, SMD).

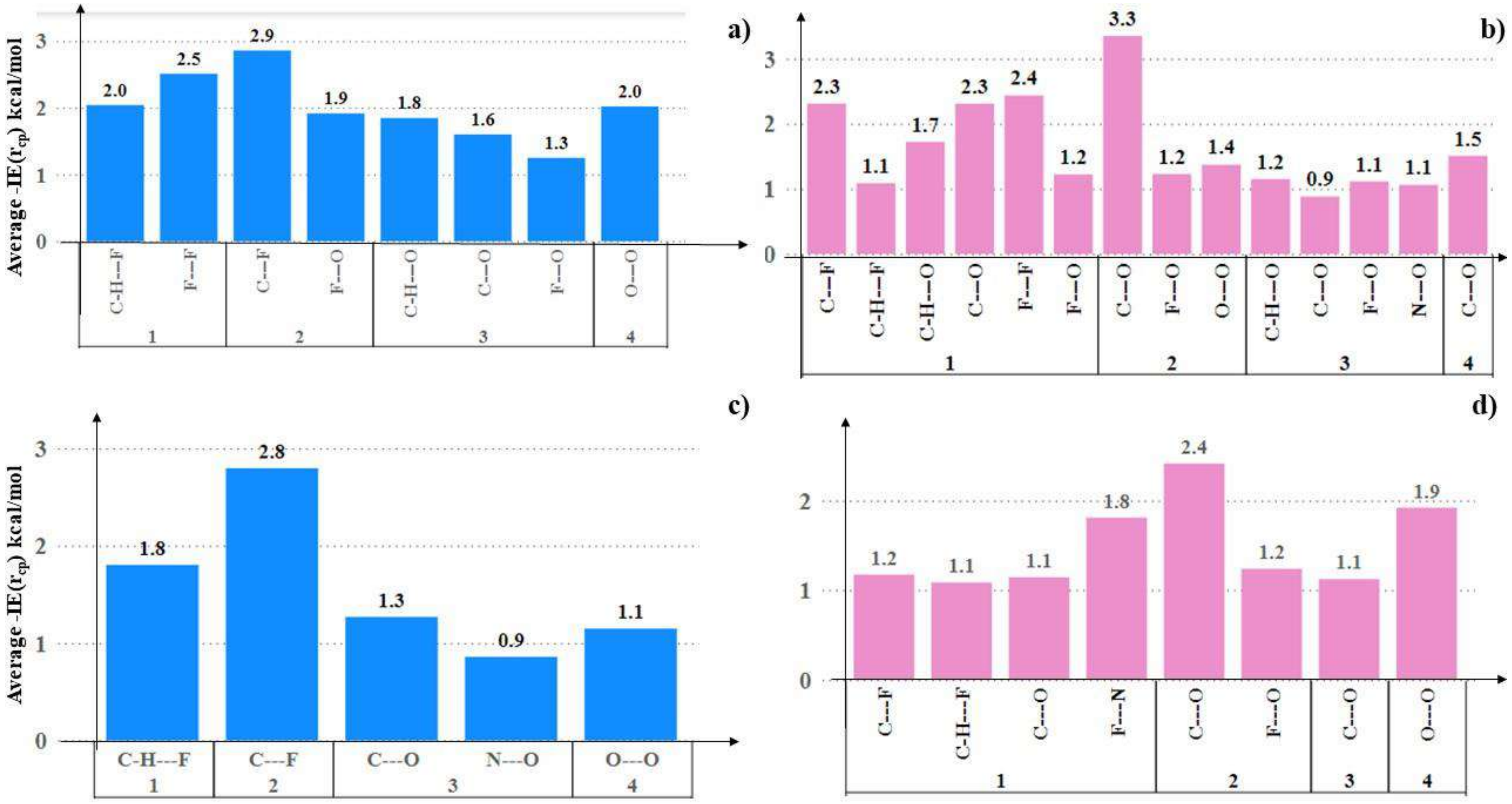

Figure S42. Average values of Interaction Energy values -IE(rcp) for the different types of interactions: 1 = Anion-Cation, 2 = Anion-CO_2, 3 = Cation-CO_2, 4 = CO_2-CO_2 per interaction class for 2CO2. a) $[C_8H_4F_{13}mim]^+[BF_4]^-$, b) $[C_8H_4F_{13}mim]^+[TFO]^-$, c) $[Dmim]^+[BF_4]^-$, d) $[Dmim]^+[TFO]^-$ at a level of theory M06-2X/cc-pVTZ (D3, SMD).

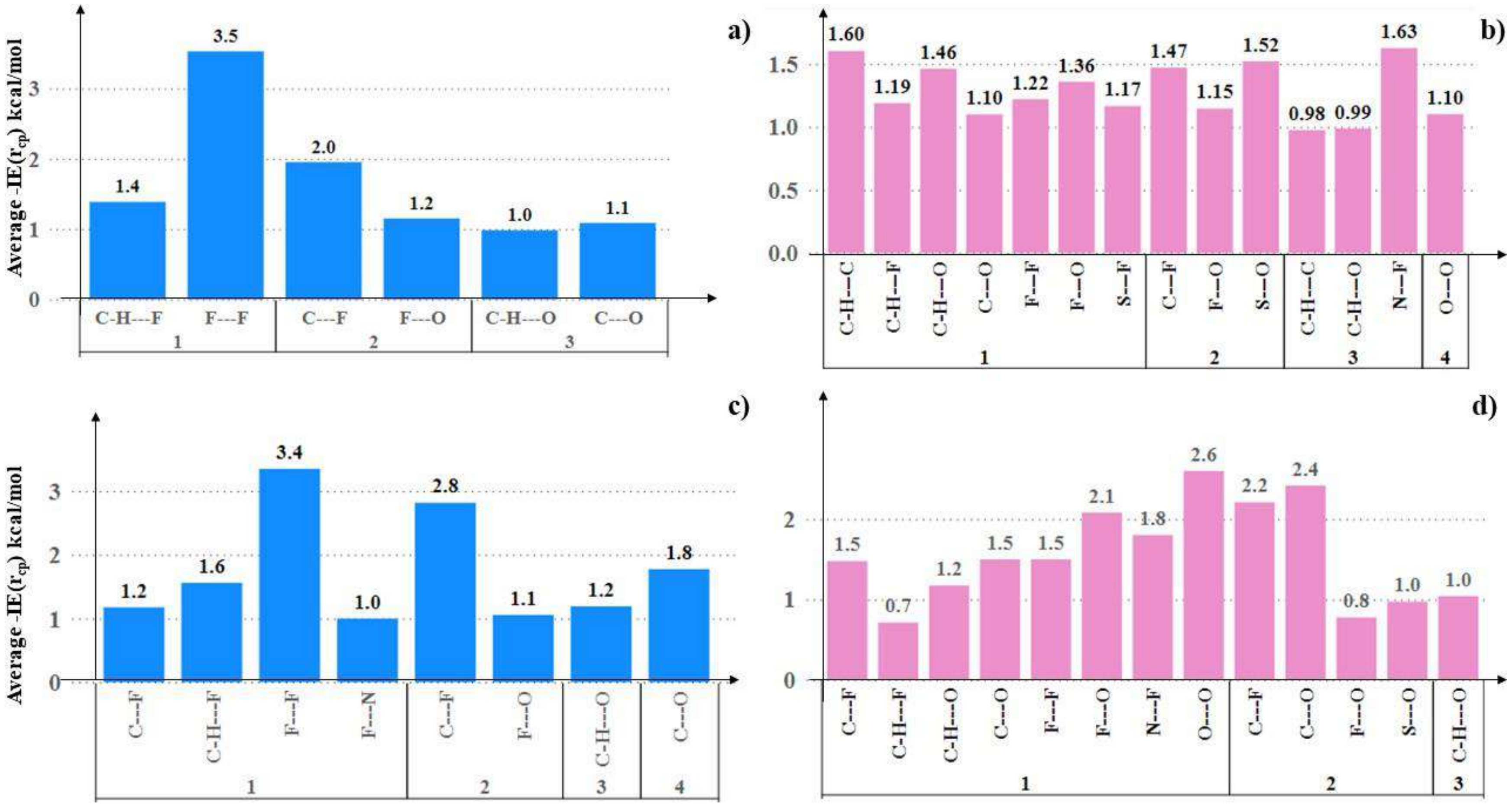

Figure S43. Average values of Interaction Energy values -IE(rcp) for the different types of interactions: 1 = Anion-Cation, 2 = Anion-CO_2, 3 = Cation-CO_2, 4 = CO_2-CO_2 per interaction class for 2CO2. a) $[Dbim]^+[FAP]^-$, b) $[Dbim]^+[Methide]^-$, c) $[Hmim]^+[FAP]^-$, d) $[Hmim]^+[Methide]^-$ at a level of theory M06-2X/cc-pVTZ (D3, SMD).

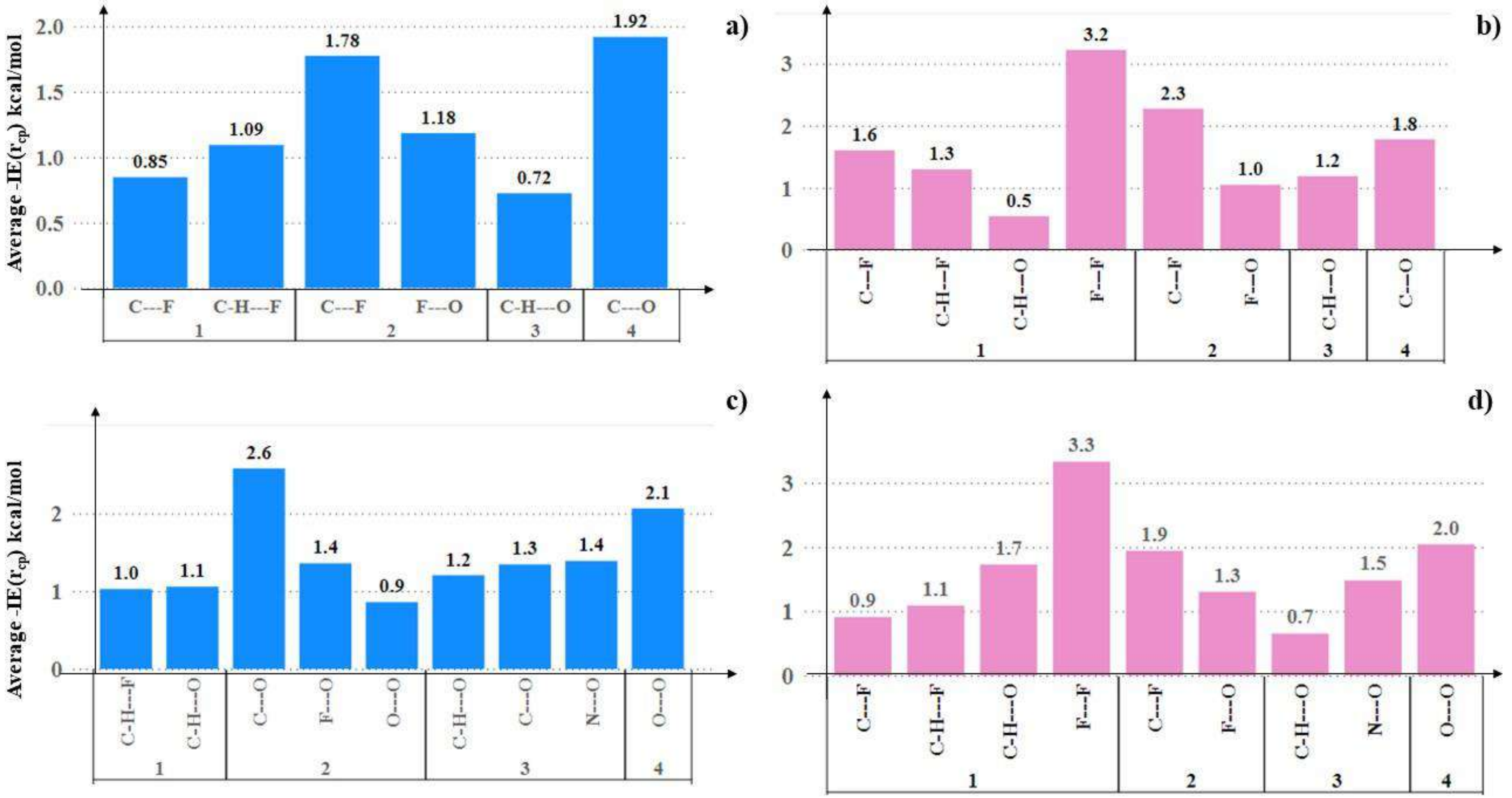

Figure S44. Average values of Interaction Energy values -IE(rcp) for the different types of interactions: 1 = Anion-Cation, 2 = Anion-CO_2, 3 = Cation-CO_2, 4 = CO_2-CO_2 per interaction class for 2CO2. a) $[Hmim]^+[(PFOc)SO_3]^-$, b) $[Hmim]^+[(PFBu)SO_3]^-$, c) $[Omim]^+[(PFOc)SO_3]^-$, d) $[Omim]^+[(PFBu)SO_3]^-$ at a level of theory M06-2X/cc-pVTZ (D3, SMD).

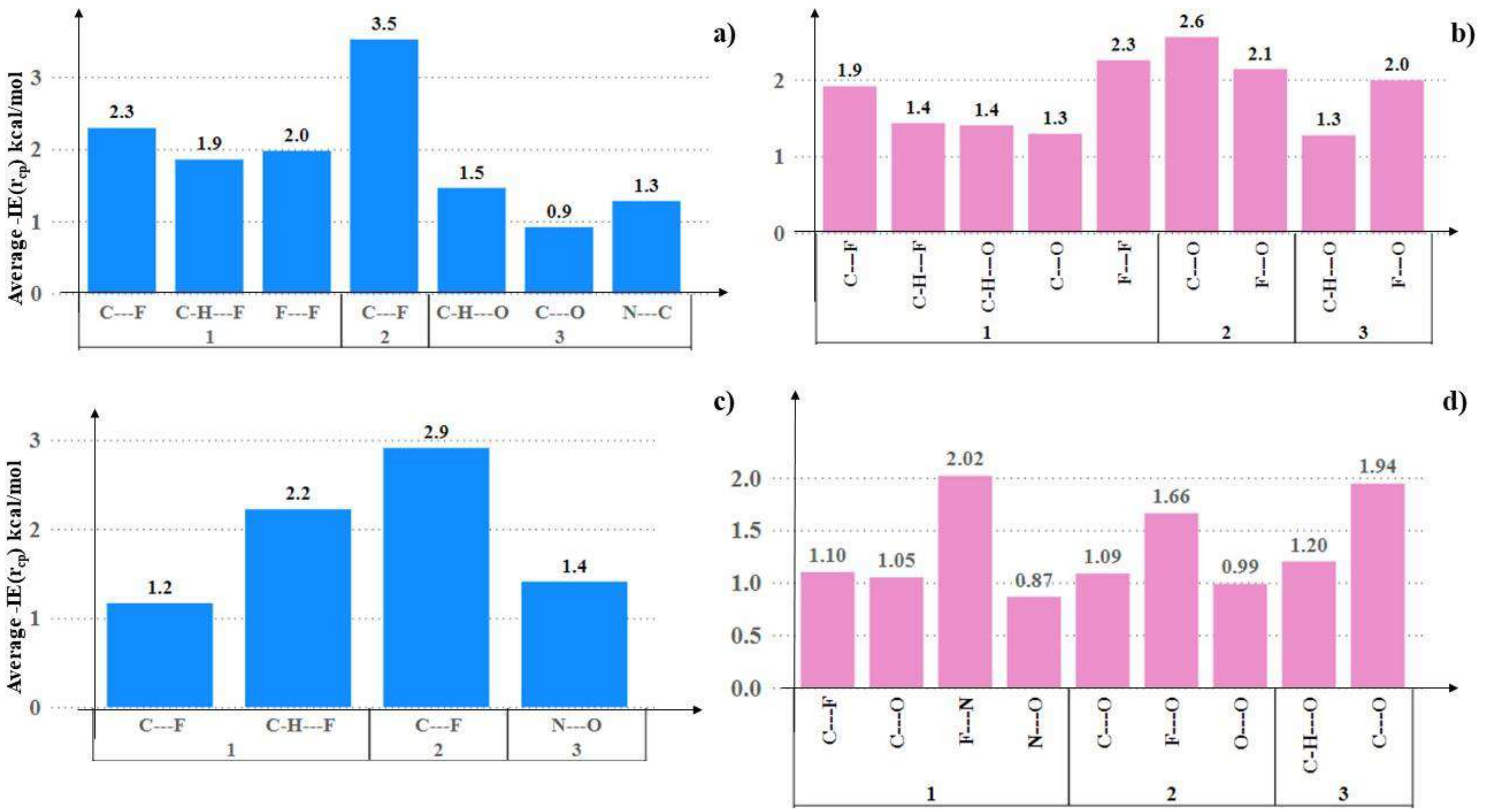

Figure S45. Average values of Interaction Energy values -IE(rcp) for the different types of interactions: 1 = Anion-Cation, 2 = Anion-CO_2, 3 = Cation-CO_2, 4 = CO_2-CO_2 per interaction class for n = 1. a) $[C_8H_4F_{13}mim]^+[BF_4]^-$, b) $[C_8H_4F_{13}mim]^+[TFO]^-$, c) $[Dmim]^+[BF_4]^-$, d) $[Dmim]^+[TFO]^-$ at a level of theory M06-2X/cc-pVTZ (D3, SMD).

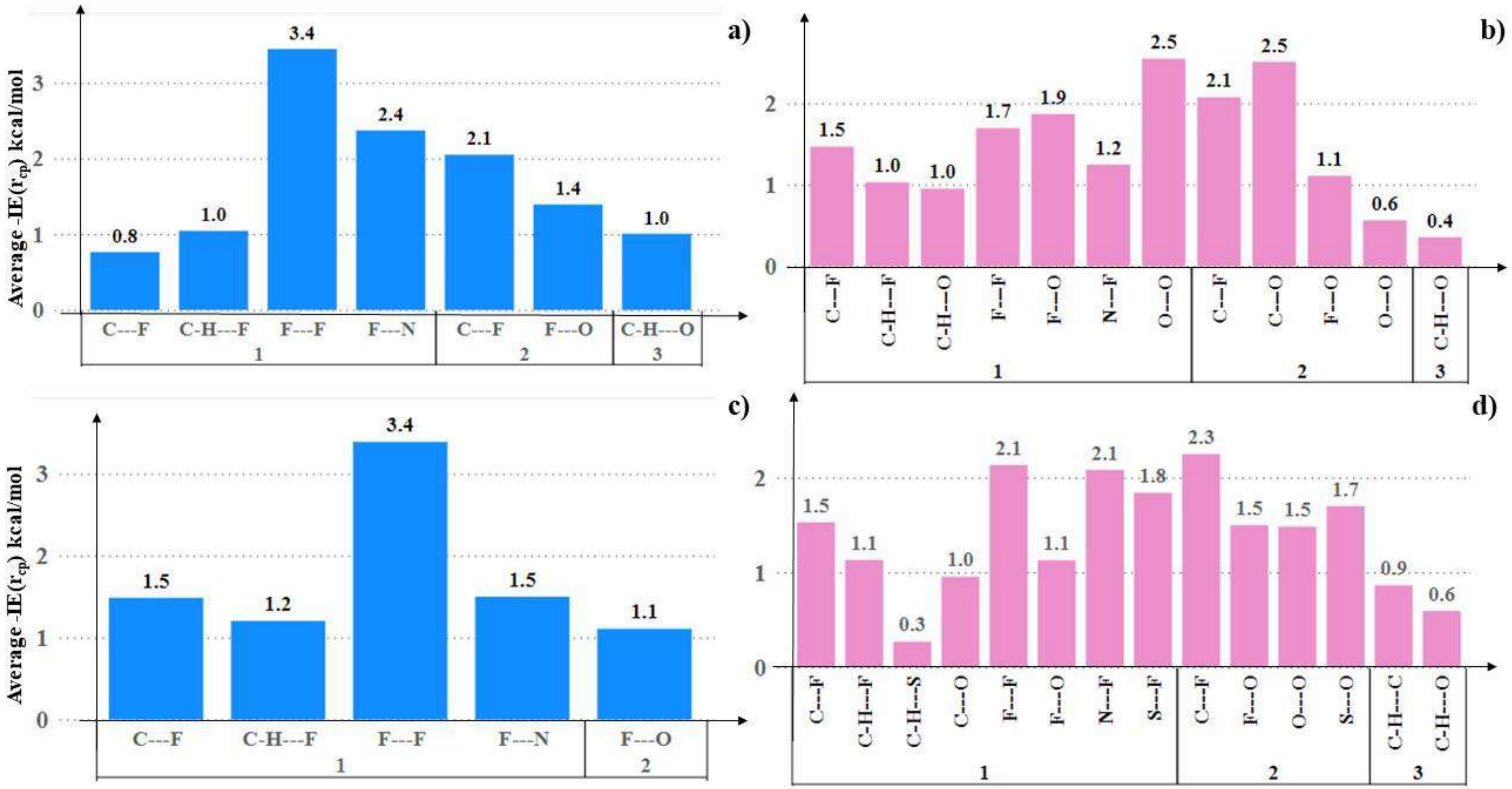

Figure S46. Average values of Interaction Energy values -IE(rcp) for the different types of interactions: 1 = Anion-Cation, 2 = Anion-CO_2, 3 = Cation-CO_2, 4 = CO_2-CO_2 per interaction class for 1CO2. a) $[Dbim]^+[FAP]^-$, b) $[Dbim]^+[Methide]^-$, c) $[Hmim]^+[FAP]^-$, d) $[Hmim]^+[Methide]^-$ at a level of theory M06-2X/cc-pVTZ (D3, SMD).

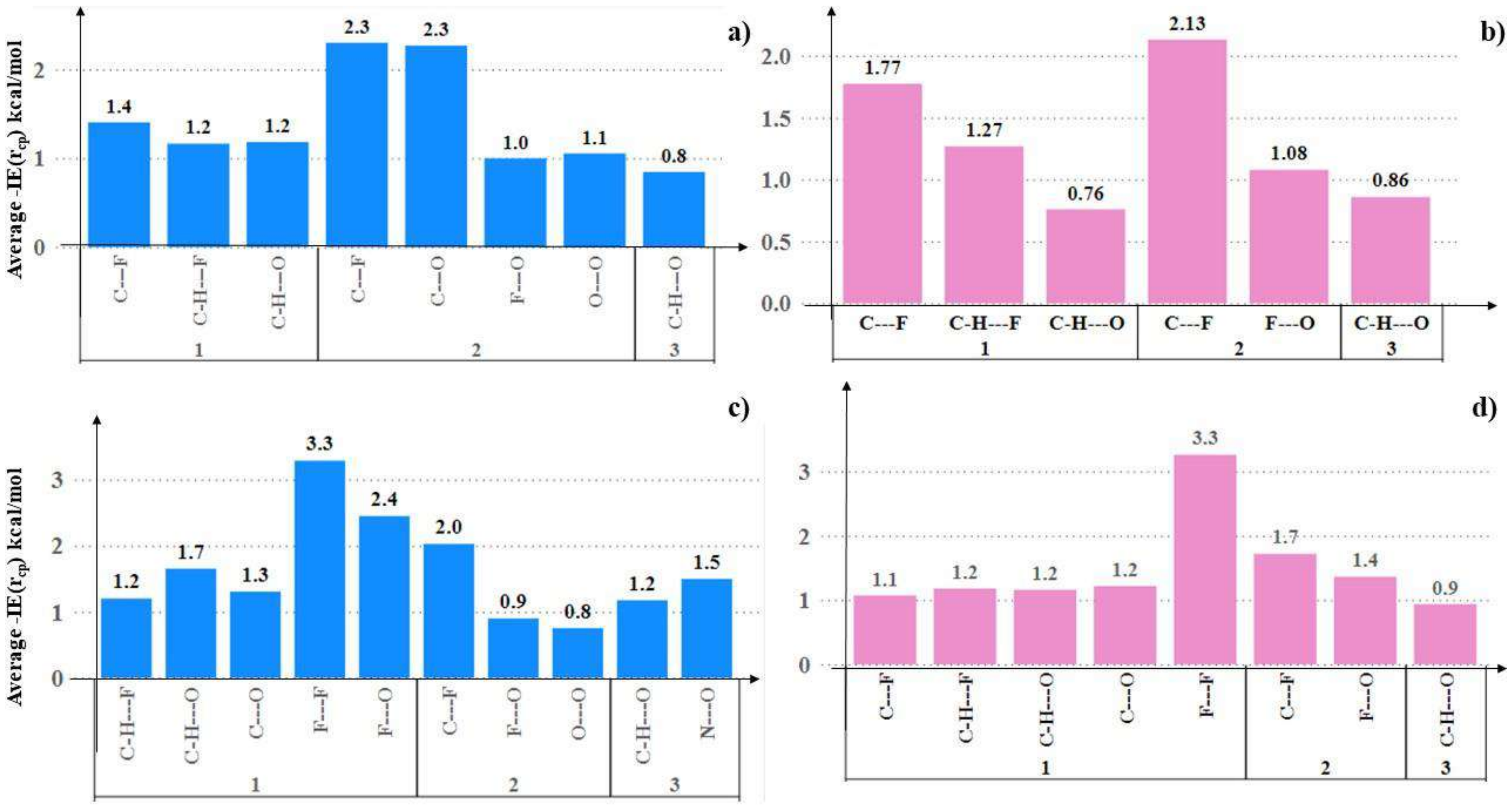

Figure S47. Average values of Interaction Energy values -IE(rcp) for the different types of interactions: 1 = Anion-Cation, 2 = Anion-CO_2, 3 = Cation-CO_2, 4 = CO_2-CO_2 per interaction class for n = 1. a) $[Hmim]^+[(PFOc)SO_3]^-$, b) $[Hmim]^+[(PFBu)SO_3]^-$, c) $[Omim]^+[(PFOc)SO_3]^-$, d) $[Omim]^+[(PFBu)SO_3]^-$ at a level of theory M06-2X/cc-pVTZ (D3, SMD).

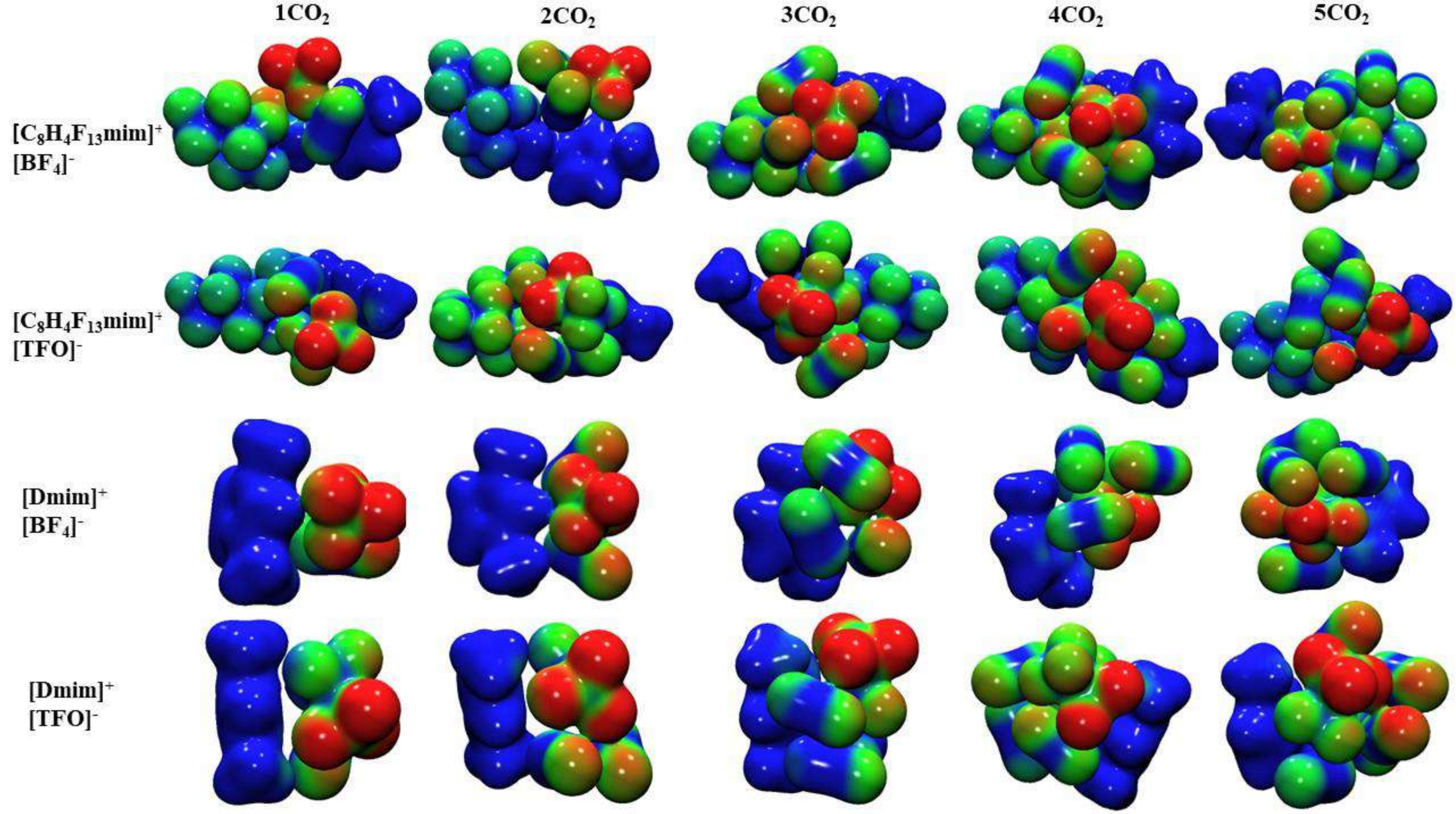

Figure S48. Electrostatic potential maps for $nCO_2[C_8H_4F_{13}mim]^+[BF4]^-$, $nCO_2[C_8H_4F_{13}mim]^+[TFO]^-$, $nCO_2[Dmim]^+[BF_4]^-$, $nCO_2[Dmim]^+[TFO]^-$ molecular clusters. All isosurfaces were generated at 0.01 a.u.

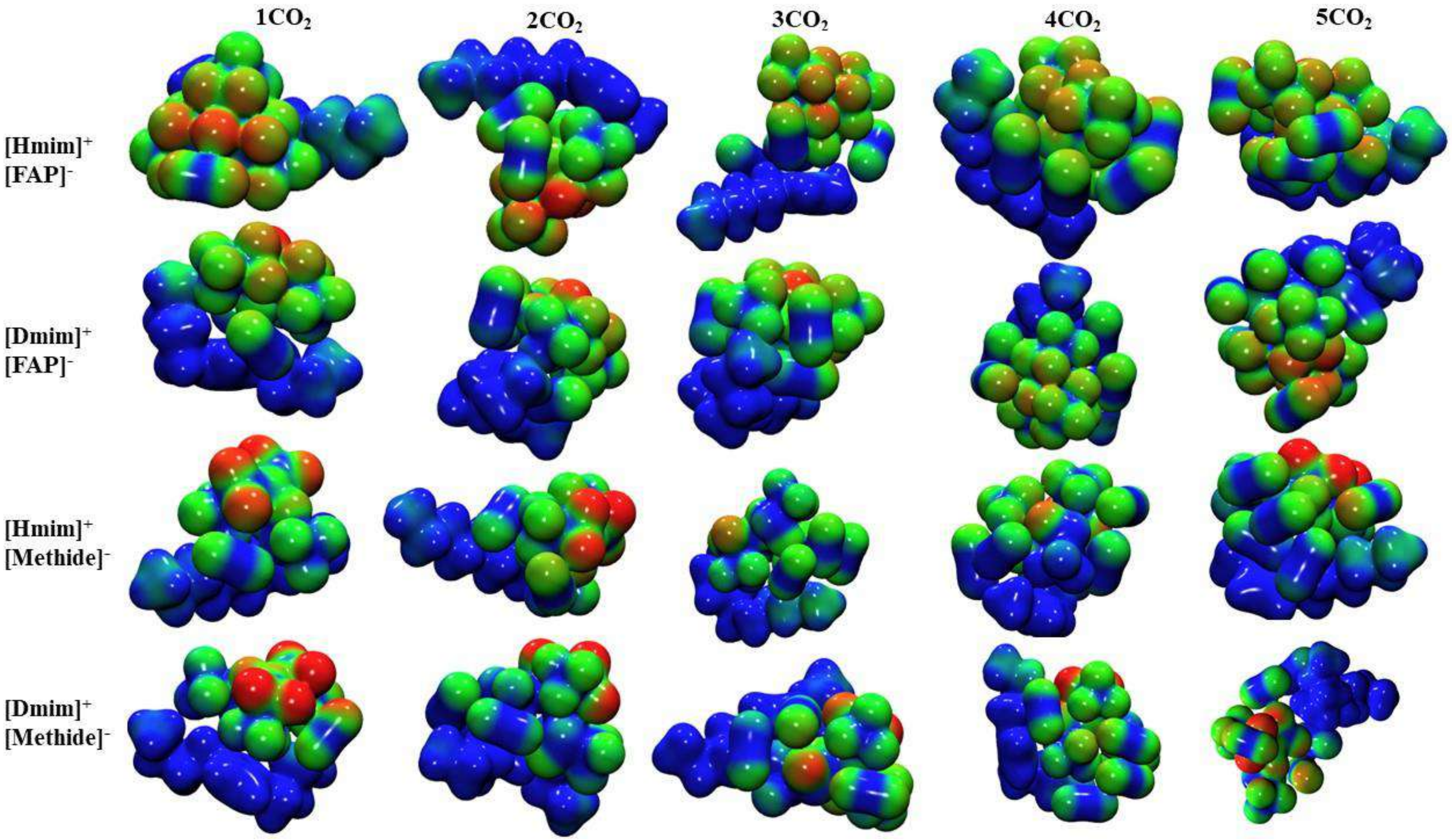

Figure S49. Electrostatic potential maps for $nCO_2[Dbim]^+[FAP]^-$, $nCO_2[Hmim]^+[FAP]^-$, $nCO_2[Dbim]^+[Methide]^-$, $nCO_2[Hmim]^+[Methide]^-$ molecular clusters with n = 1 a 5. All isosurfaces were generated at 0.01 a.u.

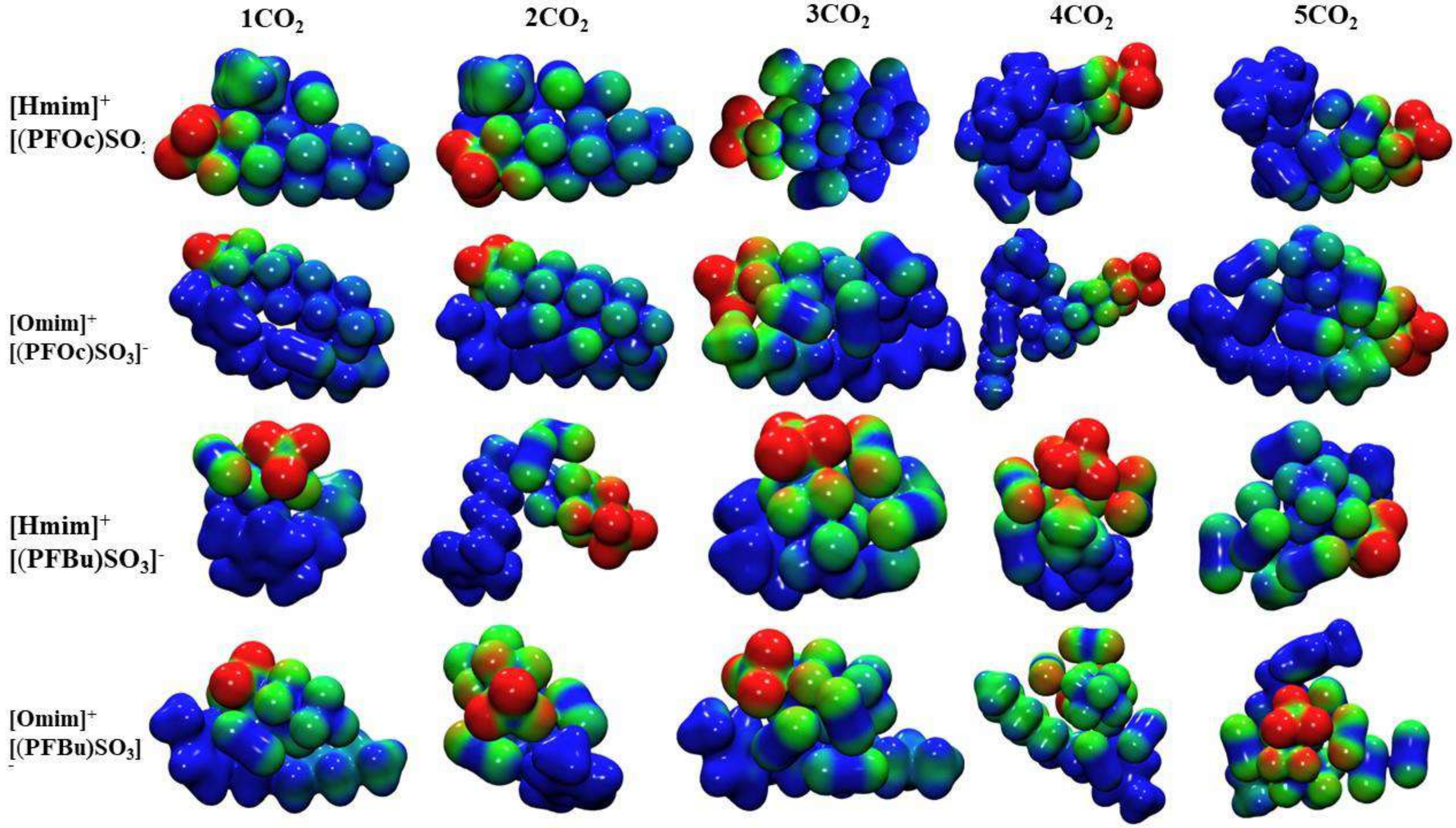

Figure S50. Electrostatic potential maps for $nCO_2[Hmim]^+[(PFOc)SO_3]^-$, nCO_2 $[Hmim]^+[(PFBu)SO_3]^-$, $nCO_2[Omim]^+[(PFBu)SO_3]^-$ molecular clusters with n = 1 a 5. All isosurfaces were generated at 0.01 a.u.
